# The mining and construction of a knowledge base for gene-disease association in mitochondrial diseases

**DOI:** 10.1038/s41598-021-03249-0

**Published:** 2021-12-13

**Authors:** Wei Wang, Junying Song, Yunhai Chuai, Fu Chen, Chunlan Song, Mingming Shu, Yayun Wang, Yunfei Li, Xinyu Zhai, Shujie Han, Shun Yao, Kexin Shen, Wei Shang, Lei Zhang

**Affiliations:** 1 Department of Obstetrics and Gynecology, The Seventh Medical Center of Chinese PLA General Hospital, No. 5, Nanmencang Hutong, Dongsishitiao, Dongcheng District, Beijing, 100027 China; 2 Department of Obstetrics and Gynecology, Chinese PLA General Hospital, No. 28, Fuxing Road, Haidian District, Beijing, 100853 China; 3 Harrison International Peace Hospital, Hengshui, China; 4 Department of Histology and Embryology, Hebei Medical University, No. 361, Zhongshan East Road, Shijiazhuang, 050017 Hebei China; 5 Navy Clinical Medical School, Anhui Medical University, No. 81, Meishan Road, Hefei, 230032 Anhui China; 6 South China University of Technology, Guangzhou, China; 7 Beijing Geneworks Technology Co., Ltd., Beijing, China

**Keywords:** Genetics, Medical genetics, Genetic counselling

## Abstract

Mitochondrial diseases are a group of heterogeneous genetic metabolic diseases caused by mitochondrial DNA (mtDNA) or nuclear DNA (nDNA) gene mutations. Mining the gene-disease association of mitochondrial diseases is helpful for understanding the pathogenesis of mitochondrial diseases, for carrying out early clinical diagnosis for related diseases, and for formulating better treatment strategies for mitochondrial diseases. This project researched the relationship between genes and mitochondrial diseases, combined the Malacards, Genecards, and MITOMAP disease databases to mine the knowledge on mitochondrial diseases and genes, used database integration and the sequencing method of the phenolyzer tool to integrate disease-related genes from different databases, and sorted the disease-related candidate genes. Finally, we screened 531 mitochondrial related diseases, extracted 26,723 genes directly or indirectly related to mitochondria, collected 24,602 variant sites on 1474 genes, and established a mitochondrial disease knowledge base (MitDisease) with a core of genes, diseases, and variants. This knowledge base is helpful for clinicians who want to combine the results of gene testing for diagnosis, to understand the occurrence and development of mitochondrial diseases, and to develop corresponding treatment methods.

## Introduction

Mitochondrial diseases are caused by mitochondrial genome and (or) nuclear genome mutation, which leads to mitochondrial structural and functional defects and cell oxidative phosphorylation disorder. They mainly affect high energy consuming organs such as the brain, muscles, and the heart, and thus these diseases have a high mortality rate. The mitochondrial proteome contains about 1500 kinds of proteins, and only 13 of them are encoded by mitochondrial genes, while most of them are encoded by nuclear genes. The percentage of mitochondrial diseases caused by mutations is about 75–95%^1^. Mitochondrial genetics is characterized by maternal inheritance, heterogeneity, and mutation load, the threshold effect, bottlenecks, and random distribution^2^. It was found that mutations in mtDNA loci 11,778, 3460 and 14,484 could lead to Leber hereditary optic neuropathy (LHON)^3,4^. Tseng et al. found that breast cancer patients carry mtDNA mutations which are mainly located in the D loop region, coding region, tRNA, or rRNA gene region^5^. In POI patients' mt-tRNA, there are five mutations, namely tRNA C3303T, tRNA A4435G, tRNA T4363C, tRNA G5821A, and tRNAA15951G. These mutations occur in the highly conserved nucleotides of the corresponding mt-tRNA and may lead to the failure of mt-tRNA metabolism, which then leads to a disorder in mitochondrial protein synthesis^6^. The 12SrRNA gene mutation is the most important cause of hearing loss, among which the A1555G and C1494T mutations are associated with nonsyndromic hearing loss caused by aminoglycosides^7^. At present, 600 disease-related mitochondrial DNA (mitochondrial DNA, mtDNA) mutations have been identified, with a diverse range in the ages of onset, disease types, and degrees^2^.

The existing databases on human mitochondrial diseases such as MITOMAP^8^, HmtDB^9^, MSeqDR^10^, The UCLA Life Sciences Core Mitochondrial DNA Project, Human Mitochondrial Genome Polymorphism Database^11^, MitoTool^12^, mainly collect information on the structure and function of pathogenic mutations and their clinical characteristics, population-related variations, and gene–gene interactions of the mitochondrial genome. However, there is not yet a mitochondrial disease database with a core of genes, diseases and variants, that effectively integrates the correlation between mitochondrial diseases and mitochondrial gene mutations.

In this study, to research the relationship between genes and mitochondrial diseases, mining the knowledge on mitochondrial diseases and genes was carried out by combining the Malacards, Genecards, and MITOMAP disease databases, the phenolyzer tool was used to integrate disease-related genes from different databases and sort the disease-related candidate genes, and a knowledge base for gene-disease association in mitochondrial diseases was established, so as to systematically collect the information on mitochondrial disease, analyze the pathogenesis of related diseases, and provide a theoretical basis for the prevention and clinical treatment of mitochondrial diseases.

## Results

### Summary of the mitochondrial disease-gene knowledge base

**Figure 1:**
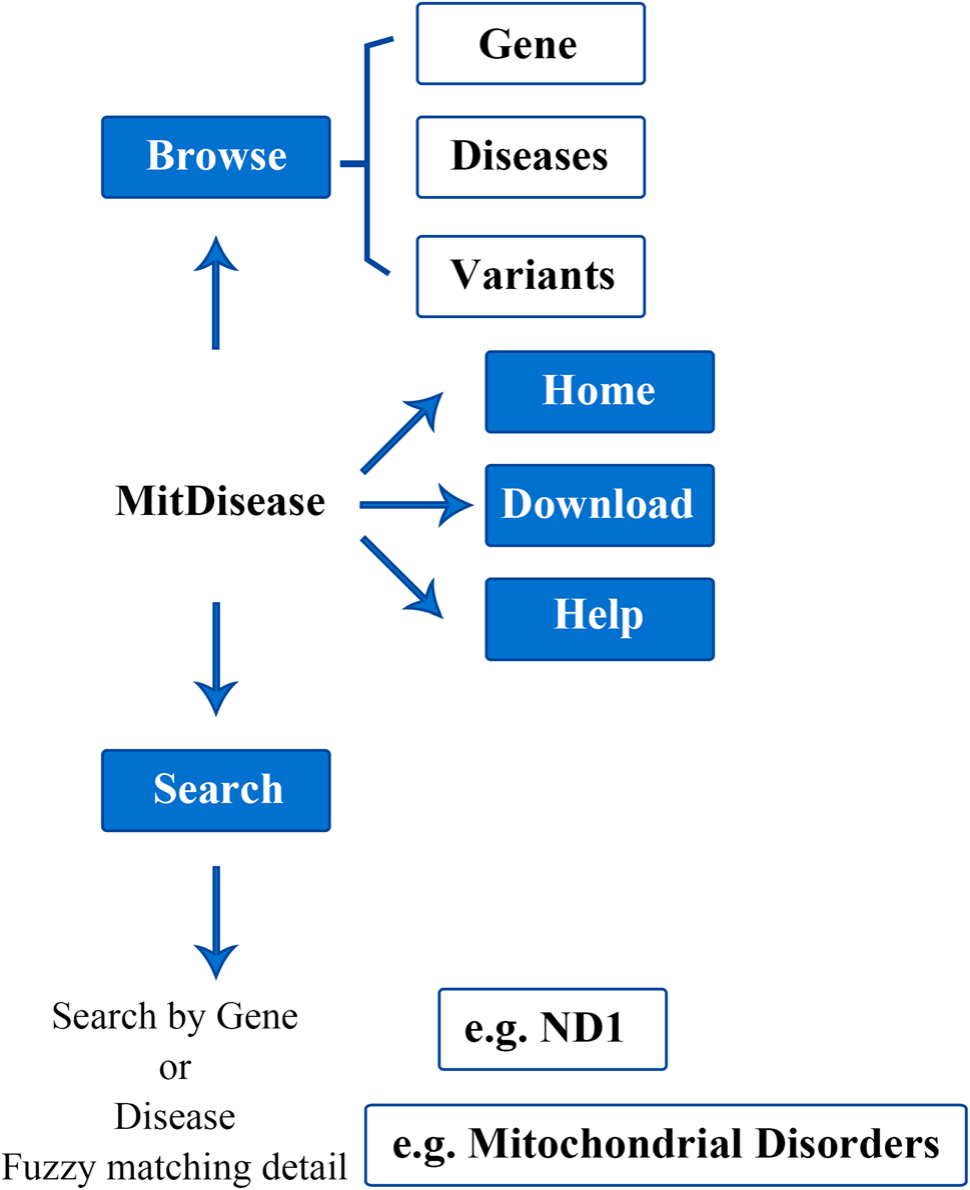
Web interface functions.

A mitochondrial disease knowledge base (MitDisease) with a core of genes, diseases, and variants was established. In the MitDisease knowledge base, 531 mitochondrial diseases were screened by combining the MalaCards, GeneCards, and MITOMAP databases (see Table 1). Using the names of the mitochondrial diseases as the key words, we extracted the gene information of mitochondrial diseases in the MalaCards and GeneCards databases in batches respectively, and finally 26,723 genes directly or indirectly related to mitochondria were extracted, 24,602 variant sites on 1474 genes were collected (see Table 2). The web interface of the MitDisease website (http://md.geneworks.cn/home) consists of five modules in the upper right corner of the page: "Home", "Browser", "Download", "Help", and "Search" (see Fig. 1). MitDisease provides multi-dimensional browsing and query functions with a core of diseases and genes.

**Table 1:** Statistical table of mitochondrial disease name screening.

Databases	Search term	No. of disease after initial screening	No. of disease after screening the disease names that contain mitochondr*	No. of disease after manual checking	No. of disease after integration
GeneCards	Mitochondrial genes	366	499	512	531
	Mitochondr*	1891			
MalaCards	Mitochondr*	3426	206		
MITOMAP	–	54	54	54

**Table 2:** Statistical table of mitochondrial genes and variations screening.

Collection types	Databases	No. of diseases containing gene or variation	No. of gene or variation	No. of gene or variation after integration
Genes	GeneCards	531	26,723	26,723
	MalaCards	498	7097	
Variations	MalaCards	316	1474 genes/24,602 variant sites	1474 genes/24,602 variant sites

### Introduction to the function of the browser module

**Figure 2:**
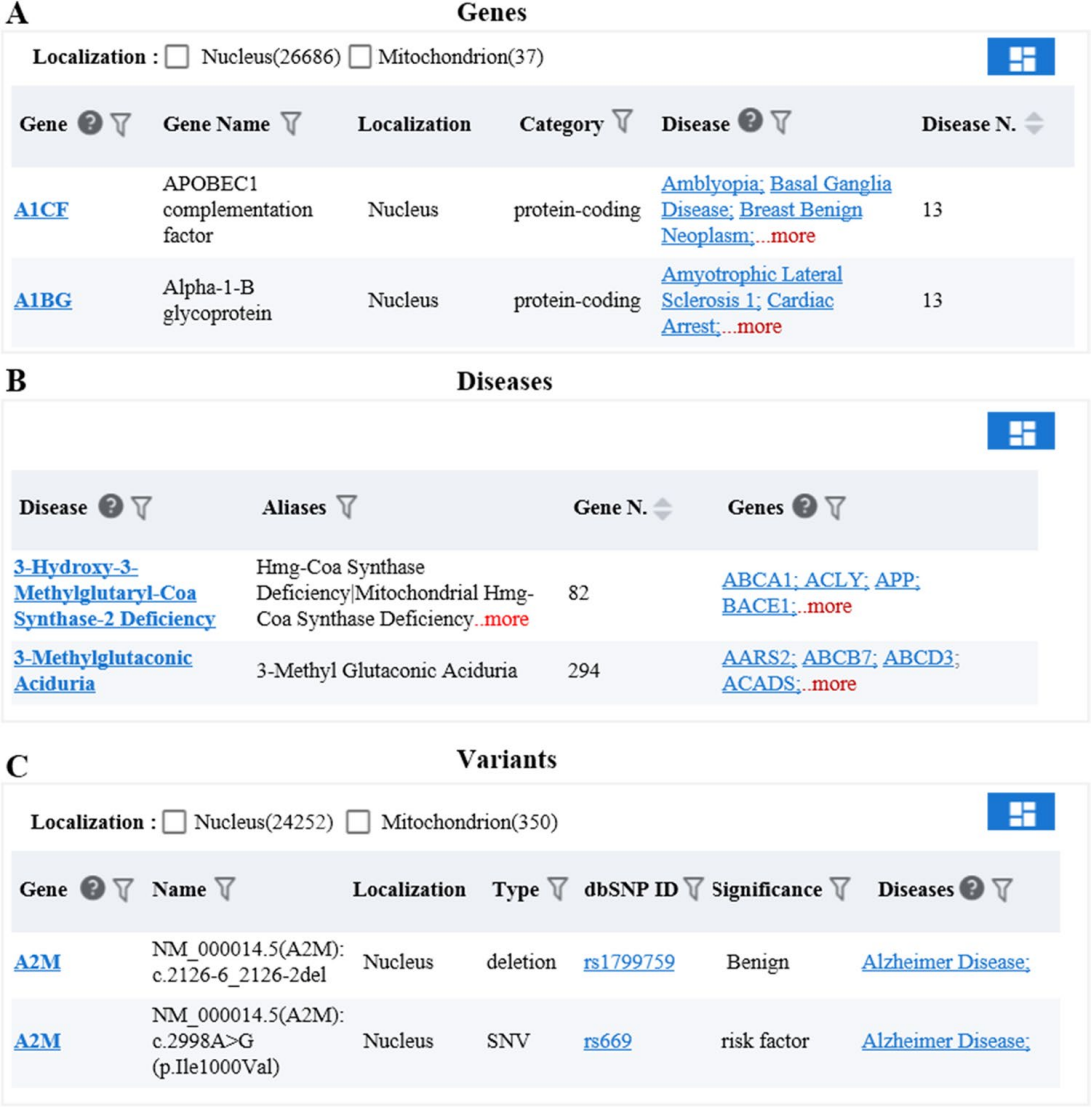
Introduction to the browser module function. (**A**) Browsing interface for the collection results of the genes associated with mitochondrial diseases. (**B**) Browsing interface for the collection results of mitochondrial related diseases. (**C**) Browsing interface for the collection results of the variant sites of genes associated with mitochondrial diseases.

The browser module provides browsing queries with "Gene", "Diseases", and "Variants" as the core, and displays all the information on gene, disease, and variant sites collected by the knowledge base. The gene module collected 26,723 genes, including 7097 seed genes directly related to mitochondrial diseases, which expanded to 19,626 predicted genes. In addition, 37 mitochondrial genes, 26,686 nuclear genes, and 18,977 coding proteins, with 42 rRNA, 32 tRNA, and 3933 ncRNA genes classified as well. The user can easily browse all the gene-related diseases and disease statistics (see Fig. 2A). The disease module collected 531 mitochondrial diseases, including diabetes, deafness, tumors, cardiovascular diseases, neurodegenerative diseases, and other diseases related to mitochondrial dysfunction. The user can easily browse the genes related to all the diseases and their statistical information (see Fig. 2B). The variants module has a core of variant sites, and we collected a total of 24,602 variant sites on 1474 genes. We then recorded the variation ID, clinical significance, mutation type, dbSNP ID, and other information on the reported variant sites of seed genes associated with mitochondrial diseases (see Fig. 2C).

### Introduction to the function of the search module

**Figure 3:**
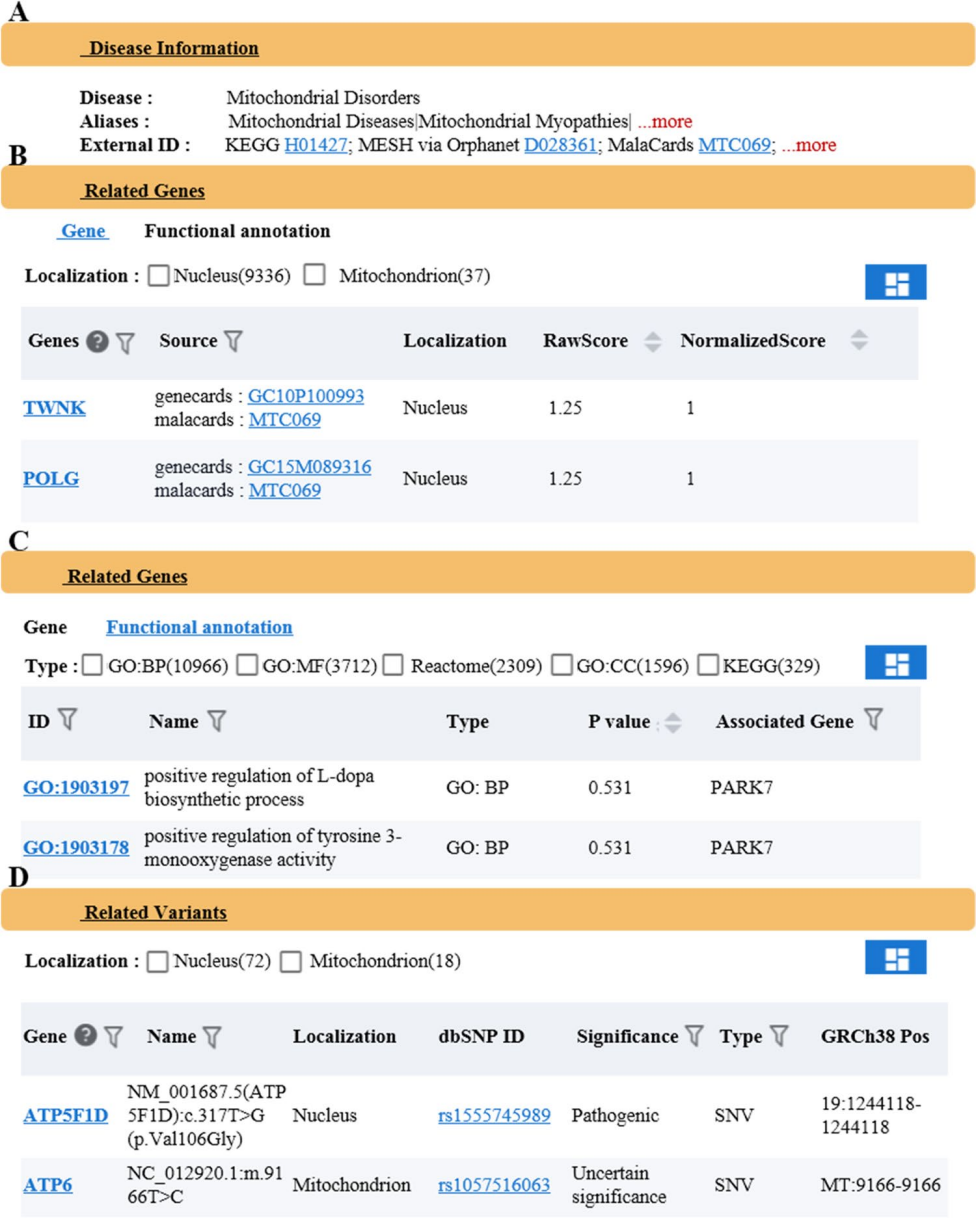
Introduction to the function of the search module with a single disease as the core. (**A**) Basic information of the searched disease, providing aliases for the disease and external links to other databases. (**B**) Disease related genes and the list of their correlation score. (**C**) Results from the functional annotation of disease-related genes in the KEGG/GO(MF/CC/BP)/Reactome databases. (**D**) Results from disease-related gene variant sites.

**Figure 4:**
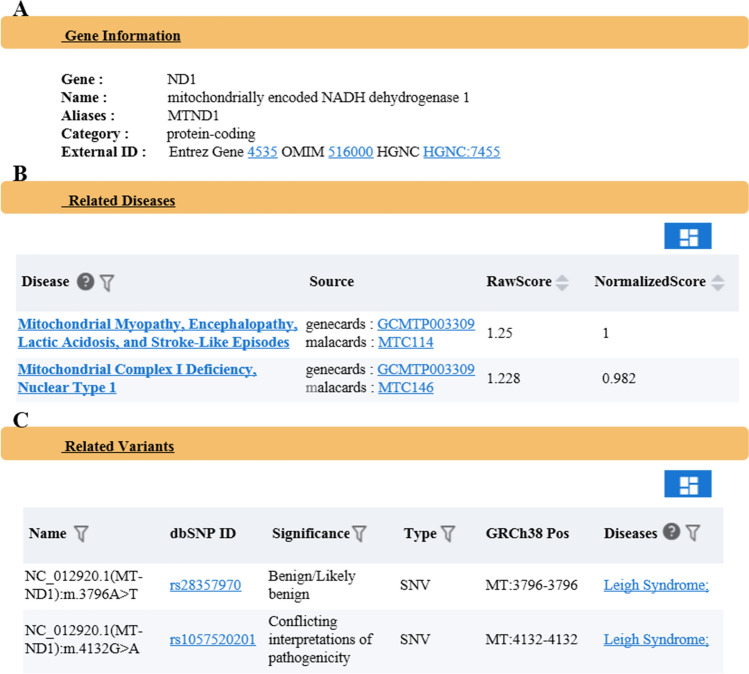
Introduction to the function of the search module with a gene as the core. (**A**) Basic function information of the searched gene. (**B**) Gene-related diseases and correlation. (**C**) Results from the gene-related variant sites.

The search module provides a search and query function with a single disease or gene as the main parameters. The user can select "Gene" or "Disease" in the drop-down box, and enter the gene or disease of interest in the text box. Detailed information for the search term will be displayed on the results page. When the user selects "Disease" and fills in "Mitochondrial Disorders", three aspects of the searched disease information will be displayed on the results page: (1) Introduction to the basic information of diseases, including aliases of diseases and external links to disease databases (such as DO, OMIM, Malacards, Mesh, etc.) (see Fig. 3A). (2) Disease-related gene information: on the one hand, information on "Mitochondrial Disorders" related genes, original scores, normalized scores, etc., is displayed, and these results are sorted from high to low values (see Fig. 3B), and on the other hand, KEGG, Reactome, GO-MF, GO-CC, and GO-BP functional enrichment analysis are performed for disease-related genes (see Fig. 3C). (3) Information from disease-related variant sites (see Fig. 3D). When the user selects "Gene" and fills in "ND1", three aspects of the searched gene information will be displayed on the results page: (1) Introduction to the basic information of the gene, including aliases for the gene and external links to other databases (such as NCBI, OMIM, HGNC, etc.) (see Fig. 4A). (2) Gene-related diseases, including associated diseases, evidence source, original scores, normalized scores, etc. (see Fig. 4B). (3) Results of all related variant sites of the ND1 gene (see Fig. 4C).

### Interactive features of the web interface

**Figure 5:**
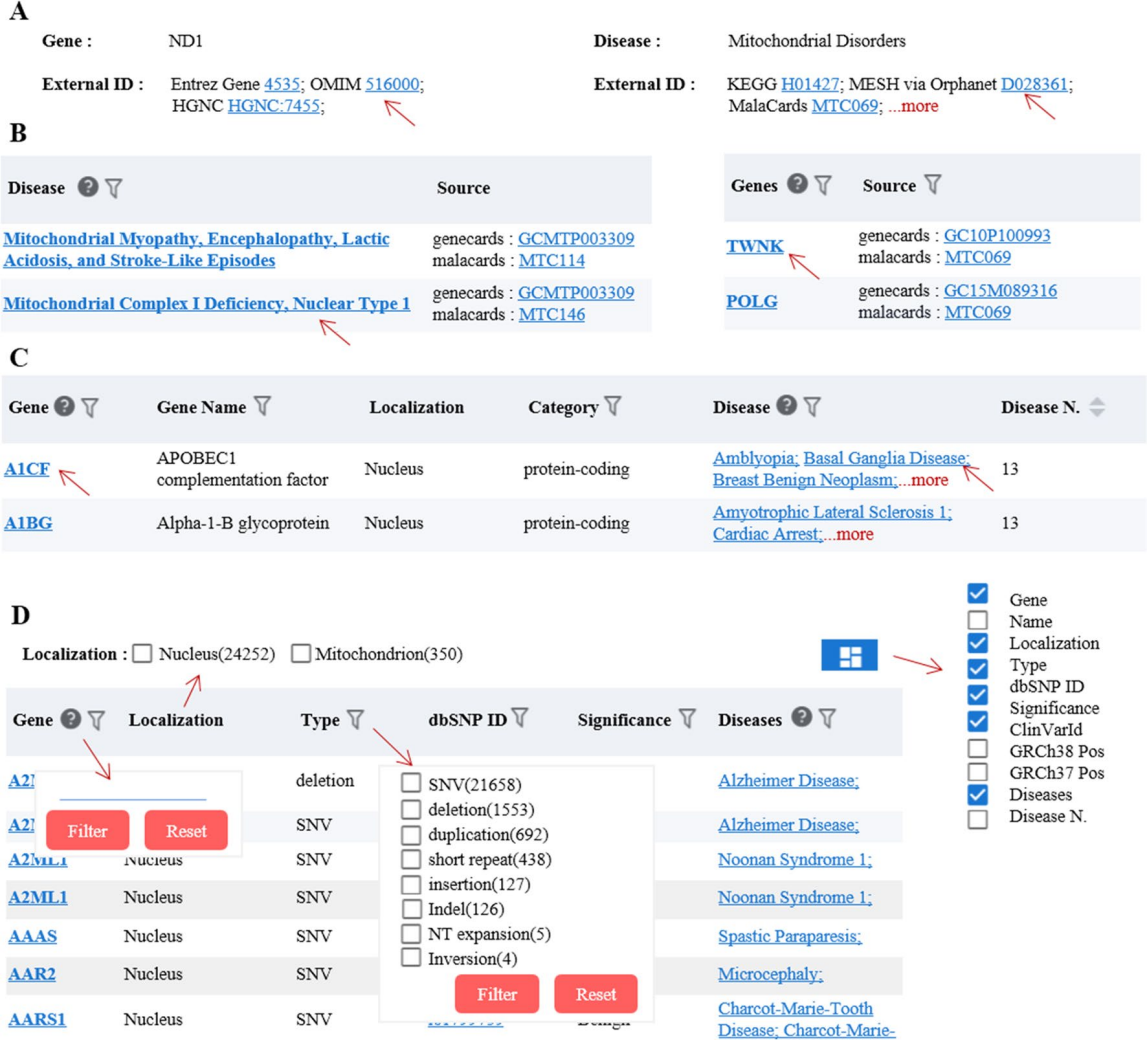
Interactive features of the web interface. (**A**) Connection of external database ID to basic information of the gene or disease. (**B**) The jump effect can be set by clicking the gene or disease on the search results page. (**C**) The jump effect is set by clicking the gene or disease on the browser results page. (**D**) The results table can be displayed with the option to hide columns, and the columns have a filtering effect (A/B/C red arrows indicate that the user can click to achieve the jump effect, while D indicates that the column filtering function can be displayed).

Hyperlinks are set in multiple positions on the website to facilitate the user to understand relevant information in multiple dimensions. The corresponding database can be jumped to by clicking the corresponding ID of the database in the "External ID" column (see Fig. 5A) corresponding to the gene information or disease information. The corresponding database results page (see Fig. 5B) can be jumped to by clicking the corresponding ID in the source column of the disease-related gene scoring results. The detailed information related to the gene or disease (see Fig. 5C) can be obtained by clicking the single disease or gene, whether it is in the browser module or on the results page of the search module. A control for displaying/hiding columns was added for all the results tables of the website, and the user can display or hide some columns according to their needs. When there are too many columns displayed, a scroll bar is available.

Different filter types have been set according to the properties of the different columns in the results table (see Fig. 5D). For example, in the variant results page of the browser, the gene column is set to the user's free input to filter the rows containing this information. The type column also has limited classification information which is displayed in drop-down form, and the user can check one or more types of interest. In order to highlight whether the gene is a mitochondrial gene, we put the classification information of the localization column at the top of the table. The disease column N is numerical and can be arranged in ascending or descending order.

## Discussion

Mitochondrial diseases are a group of heterogeneous genetic metabolic diseases caused by mitochondrial DNA (mtDNA) or nuclear DNA (nDNA) gene mutations^13^. The research on mitochondrial diseases plays an important role in human genetics. At present, the research on mitochondrial diseases and related gene mutations has become the focus of current genetic research^14,15^. In this study, a knowledge base for gene-disease association in mitochondrial diseases (MitDisease) was established.

The MitDisease knowledge base used the MalaCards^16^, GeneCards^17^, and MITOMAP^8^ databases to expand the names of mitochondrial diseases, and extract disease related genes. The MalaCards human disease database is an integrated compendium of annotated diseases consolidated from 73 data sources, and has a web card for each of 21,753 disease entries^16^. GeneCards is a searchable, integrative database that provides comprehensive, user-friendly information on all annotated and predicted human genes. The knowledgebase automatically integrates gene-centric data from about 150 web sources, including genomic, transcriptomic, proteomic, genetic, clinical and functional information^17^. Malacards and GeneCards databases both integrate a large number of databases, such as ClinVar, HMDB, MeSH, OMIM, Orphanet, UniProtKB. In addition, MITOMAP is a compendium of polymorphisms and mutations in human mitochondrial DNA. MITOMAP uses the mtDNA sequence as the unifying element for bringing together information on mitochondrial genome structure and function, pathogenic mutations and their clinical characteristics, population associated variation and gene–gene interactions^18^. Therefore, the GeneCards, MalaCards and MITOMAP databases were mainly used for the construction of the mitochondrial disease-gene knowledge base in this study.

Different from other human mitochondrial diseases databases such as MITOMAP^8^, HmtDB^9^, MSeqDR^10^, Human Mitochondrial Genome Polymorphism Database^11^, MitoTool^12^, the MitDisease knowledge base is a specialized disease knowledge base for mitochondrial diseases, which researched the relationship between genes and mitochondrial diseases, and used Python web crawler technology to mine gene-disease association of mitochondrial diseases in the MalaCards, GeneCards, and MITOMAP databases. It references the data integration and ranking algorithm of the phenolyzer tool, integrates disease-related genes from different sources, and ranked disease-related candidate genes. At the same time, it used MongoDB non-relational database for data storage and management. MitDisease provides a user-friendly web interface with a core of genes, diseases, and variants, as well as displaying detailed information about mitochondrial diseases from multiple dimensions and completing the visualization of database page. The number of mitochondrial diseases is far greater, related genes and variant sites collected by the MitDisease knowledge base are more comprehensive. A total of 531 mitochondrial related diseases were screened, 26,723 genes directly or indirectly related to mitochondria were extracted, and 24,602 variant sites on 1474 genes were sorted and collected. The MitDisease knowledge base provides a score for disease-gene association, and the user can preliminarily judge the degree of disease-gene association according to the ranking score.

The web interface of the MitDisease website consists of five modules: "Home", "Browser", "Download", "Help", and "Search". MitDisease provides multi-dimensional browsing and query functions with a core of diseases and genes. The browser module provides browsing queries with "Gene", "Diseases", and "Variants" as the core, and displays all the information on genes, diseases, variant sites and relevant statistics. The search module not only provides a search and query function with a single disease or gene as the main parameters, but also realizes the correlation analysis and functional enrichment analysis of genes, diseases and mutation sites. MitDisease realizes the interactive features of Web interface through "External ID" bar corresponding to Gene Information or Disease Information.

Although some achievements have been made in this study on the relationship between mitochondrial diseases and genes, there are still some deficiencies which need to be further improved. First, this study was limited by the scopes of the databases and thesaurus, and only carried out entity recognition for diseases in Malacards, Genecards, and MITOMAP. If the database is extended to PubMed or any other related database, it may increase the extraction of candidate gene-disease information. In addition, this study focused on genes and diseases, but didn't discuss other biomedical concepts (such as pathways, drugs, metabolism, etc.), so in the future we will consider using more relationship type entities to strengthen the discovery of connections and construct heterogeneous networks, in order to provide an important reference for clarifying the pathogenesis of mitochondrial diseases and expanding ideas for diagnosis and treatment.

## Methods

### Construction of the mitochondrial disease-gene knowledge base

**Figure 6:**
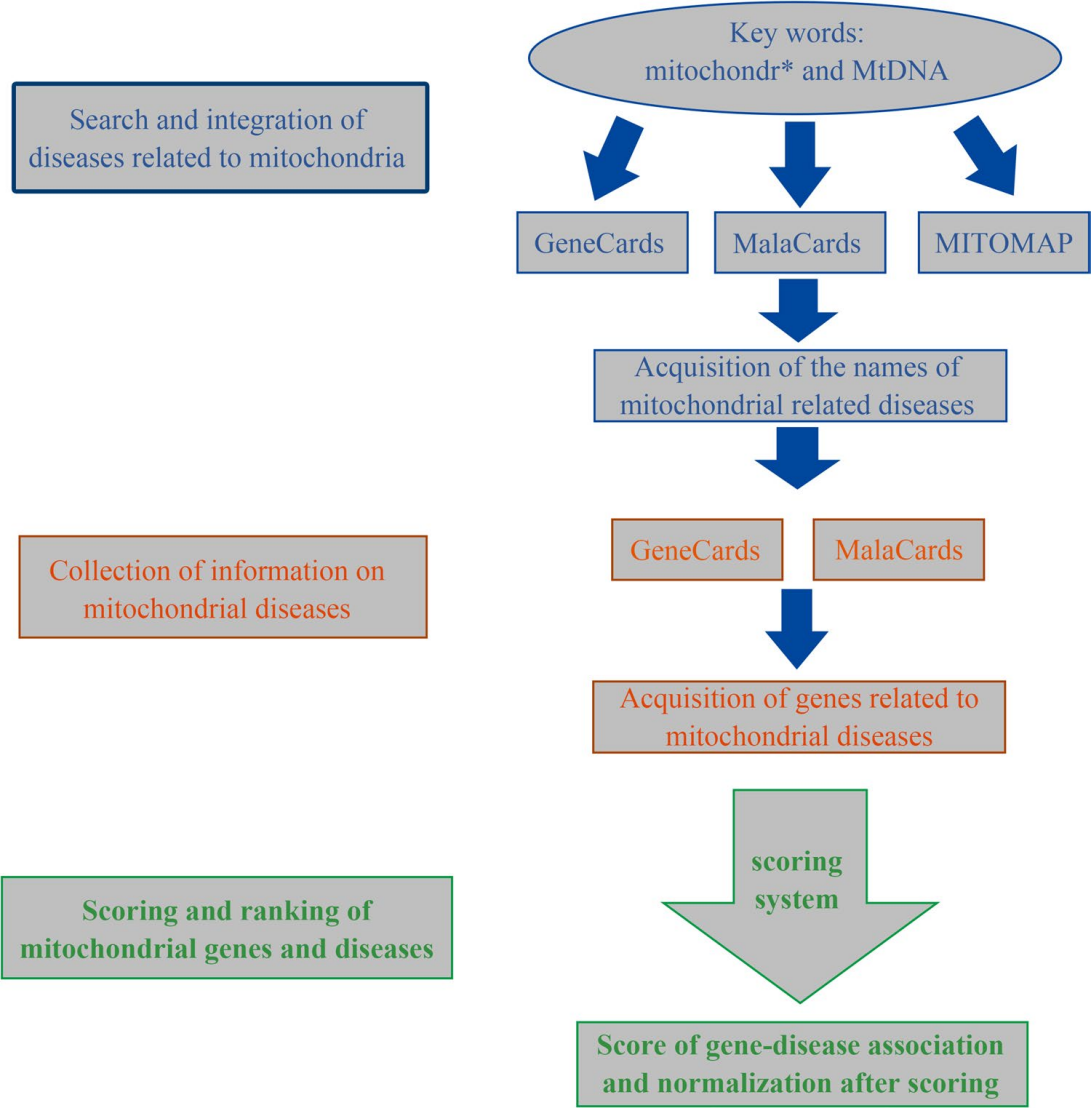
Construction process for the mitochondrial disease-gene knowledge base.

The MitDisease knowledge base used the MalaCards (http://www.malacards.org/)^16^, GeneCards (https://www.genecards.org/)^17^, and MITOMAP (https://mitomap.org/)^8^ databases to expand the names of mitochondrial diseases, and extract disease related genes. In addition, it referred to the rules of the phenolyzer^19^ tool in scoring and ranking genes and diseases from different databases. The detailed construction of the mitochondrial disease-gene knowledge base was described in the following three aspects (see Fig. 6).

### Acquisition of the names of mitochondrial related diseases

First, in the MalaCards^16^ human disease database, we used the key words such as mitochondr* and MtDNA to search the names of diseases related to mitochondria. In the GeneCards^17^ database, we used 37 genes (13 polypeptide coding genes, 22 tRNA, and 2 rRNA) encoded by mitochondria as the key words to obtain the names of diseases related to genes. In addition, the keyword mitochondr* was used in the GeneCards database to obtain the ranked TOP2000 genes, and then the gene-associated disease names were extracted in batches to retrieve the disease names related to mitochondria. In the MITOMAP^8^, a comprehensive human mitochondrial DNA database, we collected the information on the mitochondrial related diseases provided by the MITOMAP^8^ website. The disease names obtained from the different databases were integrated as preliminary candidate mitochondrial diseases. Second, crawler programs were used to capture the alias information of candidate diseases in batches, and diseases or diseases with mitochondrial related words (mitochondria, mitochondrial, Mtdna) in the alias were retained. Finally, we manually checked whether the candidate diseases were mitochondrial diseases by referring to the Mesh, Malacrads, OMIM, and other disease databases such as HmtDB^9^, MSeqDR^10^, Human Mitochondrial Genome Polymorphism Database^11^, combining this with the literature reports on related diseases from NCBI and mitochondrial disease criteria^20–22^, thereby obtaining the final names of mitochondrial diseases (see Supplementary material 1, mitoDiseaseAlias.txt). In order to ensure the reliability and quality of mitochondrial related disease data obtained, a total of 5 curators worked in checking the results, at least 4 out of 5 curators gave the same results before we reached a conclusion.

### Collection of information on mitochondrial diseases

Using the names of the final mitochondrial diseases as the key words, we extracted the gene information of mitochondrial diseases in the MalaCards and GeneCards databases in batches, standardized all the genes to Entrez ID, and then filtered out the genes that could not correspond to Entrez ID through symbol or alias (see Supplementary material 2, Homo_sapiens.gene_split). In the GeneCards database, disease-related genes, gene description information, disease-gene relevance scores can be obtained. In addition, we extracted information on “Aliases & Classifications and Variations” in batches from the MalaCards database (see Supplementary material 3, Variations_GENE_DISEASE).

### Scoring and ranking of mitochondrial genes and diseases

The disease-related genes obtained from the Malacards and Genecards databases were accompanied with gene classification and scores, and the scores indicated the reliability of the gene-disease association. First, in order to merge the related genes from different databases for the same disease, the scores in the different databases were normalized from 0–1 (see Supplementary material 4, DB_GENECARDS_GENE_DISEASE_SCORE; see Supplementary material 5, DB_MALACARDS_GENE_DISEASE_SCORE). According to the evidence of the gene-disease relationship and the extent of which such relationship is confirmed, we set the score of 100 as the threshold for each gene-disease pair in the gene-disease databases (GeneCards and MalaCards). If the gene-disease score is greater than 100, it is normalized to 1; if the score is less than 100, the score is divided by 100 as the normalized value. Second, according to the rules of the Phenotype Based Gene Analyzer (phenolyzer)^19^, a tool focusing on discovering genes based on user-specific disease/phenotype terms, the gene-disease scores from different databases were normalized again and ranked, the specific algorithms used were as follows:

Score of gene-disease association Eq. (1):

1 $$ S\left( {{\text{Gene}},\;{\text{Disease}}} \right) = \sum {{\text{Score}}\left( {{\text{Gene}},\;{\text{Disease}}} \right) \times {\text{Realiability}}} $$

Score(Gene, Disease) in the Eq. (1) represented the normalized score of the retrieved gene-disease association in one data source calculated in the first step. Reliability in the Eq. (1) represented the weight value of the data source scoring, which was determined according to the reliability of the data source. The reliability of the GeneCards databases and the MalaCards databases was 0.25 and 1, respectively. S(Gene, Disease) represented the sum of the scores of the retrieved gene-disease association in all data sources.

Normalization after scoring Eq. (2):

2 $$ \tilde{S}\left( {{\text{Gene}},\;{\text{Disease}}} \right) = \frac{{S\left( {{\text{Gene}},\;{\text{Disease}}} \right)}}{{\max \left\{ {S\left( {{\text{Gene}},\;{\text{Disease}}} \right)} \right\}}} $$

The Eq. (2) normalized the score by dividing the actual score by the maximum score, and the normalized gene-disease association score value is between 0 and 1.

### Gene function annotation

**Figure 7:**
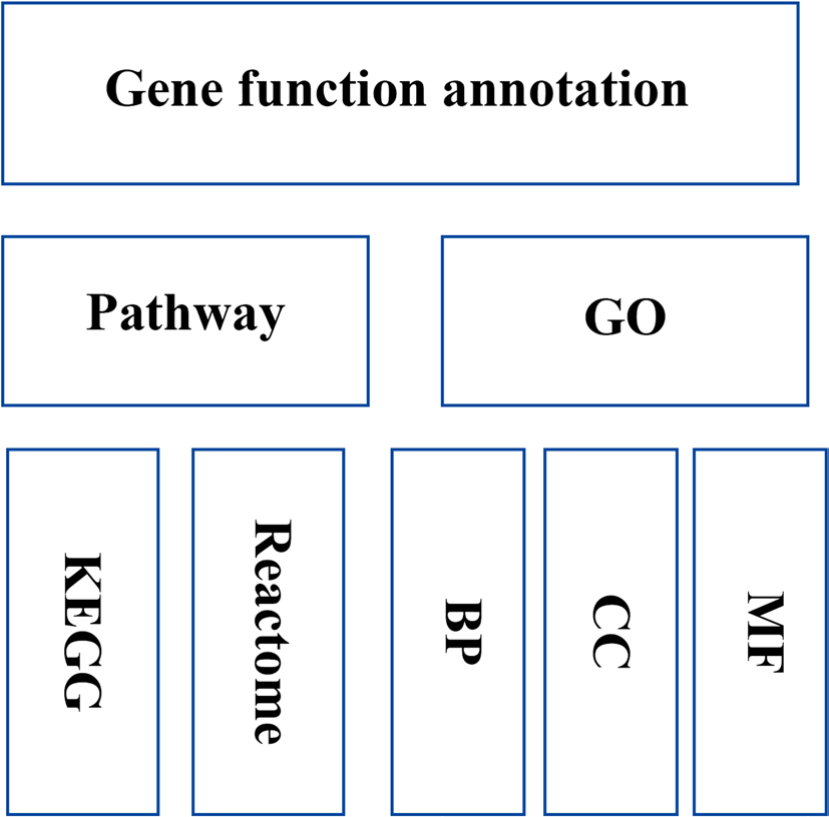
Gene function annotation.

We downloaded the annotation information of pathways and gene ontology from the Reactome (https://reactome.org/, version 1)^23^, KEGG PATHWAY (https://www.kegg.jp/kegg/pathway.html, the KEGG API in September 2020)^24,25^, and GO (http://geneontology.org/, version 1.4)^26^ databases (see Supplementary material 6, Reactome_enrichment; see Supplementary material 7, KEGG_enrichment; see Supplementary material 8, GOBP_enrichment/GOCC_enrichment/ GOMF_enrichment), and then extracted the database ID and the corresponding gene information to complete the collation of the gene function annotation library (see Fig. 7). Based on the gene function annotation library, we used the scipy package (scipy.stats. hypergeom for Fisher Test) in Python to enrich and analyze the disease-related genes and calculate the p-value by multiple testing-FDR, using the python package (statsmodels. stats. multitest. fdrcorrection).

### Web realization

The MitDisease website provided a user-friendly web interface. HTML 5.5.31 and JavaScript were used for the front-end development of the MitDisease knowledge base, and the MongoDB non-relational database was used for data storage and management. In this website, the crawling of web pages, the calling of calculation programs, and the API interface were all completed by Python and its dependent packages.

## Supplementary Information

Supplementary Information 1.

Supplementary Information 2.

Supplementary Information 3.

Supplementary Information 4.

Supplementary Information 5.

Supplementary Information 6.

Supplementary Information 7.

Supplementary Information 8.

Supplementary Information 9.

Supplementary Information 10.

Supplementary Information 11.
