# Supplementary material for: The mining and construction of a knowledge base for gene-disease association in mitochondrial diseases
Source: Sci Rep. 2021 Dec 13;11:23909. doi: 10.1038/s41598-021-03249-0 (PMC8668972; doi:10.1038/s41598-021-03249-0)
Supplement: Supplementary file 1 — Supplementary Information 1. [file 41598_2021_3249_MOESM1_ESM.pdf]

The Mining and Construction of a Knowledge Base for Gene-Disease Association in Mitochondrial Diseases

Wei Wang[a,b,c,d], Junying Song[c], Yunhai Chuai[a], Fu Chen[a], Chunlan Song[a], Mingming Shu[a], Yayun Wang[g], Yunfei Li[d], Xinyu Zhai[e], Shujie Han[e], Shun Yao[e], Kexin Shen[f], Wei Shang[a,b,e]*, Lei Zhang[d,]*

Supplementary material

Supplementary material 1, mitoDiseaseAlias;

Supplementary material 2, Homo_sapiens.gene_split;

Supplementary material 3, Variations_GENE_DISEASE;

Supplementary material 4, DB_GENECARDS_GENE_DISEASE_SCORE;

Supplementary material 5, DB_MALACARDS_GENE_DISEASE_SCORE;

Supplementary material 6, Reactome_enrichment;

Supplementary material 7, KEGG_enrichment;

Supplementary material 8, GOBP_enrichment, GOCC_enrichment, GOMF_enrichment.
