# Supplementary material for: The mining and construction of a knowledge base for gene-disease association in mitochondrial diseases
Source: Sci Rep. 2021 Dec 13;11:23909. doi: 10.1038/s41598-021-03249-0 (PMC8668972; doi:10.1038/s41598-021-03249-0)
Supplement: Supplementary file 7 — Supplementary Information 7. [file 41598_2021_3249_MOESM7_ESM.docx]

ID,geneSymbol,name,geneN

R-HSA-1059683,"IL6,IL6R,IL6ST,IL6ST,JAK1,JAK1,JAK2,JAK2,PTPN11,STAT1,STAT3,TYK2,TYK2,CBL,SOCS3",Interleukin-6 signaling,15

R-HSA-109581,"AKT3,BCL2L11,TMED7-TICAM2,OCLN,DNM1L,BCAP31,PSME3,PSMD14,CDKN2A,YWHAQ,OMA1,ADD1,PSMB11,DYNLL2,PSMA8,TICAM1,CTNNB1,DAPK1,DAPK3,DCC,DFFA,DFFB,DSG1,DSG2,DSG3,DSP,E2F1,AKT1,AKT2,UNC5B,CARD8,ACIN1,PSME4,PPP1R13B,FNTA,DAPK2,LY96,APPL1,GAS2,BBC3,SFN,DBNL,GSN,GZMB,H1-0,H1-2,H1-3,H1-4,H1-5,H1-1,HMGB1,HMGB2,APAF1,APAF1,APC,BIRC2,XIAP,XIAP,TICAM2,FAS,FASLG,KPNA1,KPNB1,LMNA,LMNB1,MAPT,NMT1,OPA1,PAK2,APIP,STK26,PKP1,PLEC,PMAIP1,SEPTIN4,CYCS,CYCS,UACA,PPP3CC,PPP3R1,PRKCD,PRKCQ,MAPK1,MAPK3,MAPK8,DIABLO,DIABLO,PSMA1,PSMA2,PSMA3,PSMA4,PSMA5,PSMA6,PSMA7,PSMB1,PSMB2,PSMB3,PSMB4,PSMB5,PSMB6,PSMB7,PSMB8,PSMB9,PSMB10,PSMC1,PSMC2,PSMC3,PSMC4,PSMC5,PSMC6,PSMD1,PSMD2,PSMD3,AVEN,PSMD4,PSMD5,PSMD7,PSMD8,PSMD9,PSMD10,PSMD11,PSMD12,PSMD13,BAD,BAD,PSME1,PSME2,PTK2,BAK1,BAX,BCL2,BCL2L1,ROCK1,RPS27A,SATB1,BID,CLSPN,BMX,SPTAN1,STAT3,TFDP1,TFDP2,C1QBP,TJP1,TLR4,TP53,TP53BP2,TP73,TRAF2,UBA52,UBB,UBC,VIM,YWHAB,YWHAE,YWHAG,YWHAH,YWHAZ,ARHGAP10,SEM1,CASP3,CASP3,CASP6,CASP7,CASP8,CASP9,CASP9,STK24,TP63,DYNLL1,TRADD,RIPK1,TNFSF10,FADD,TNFRSF10B,TNFRSF10A,CFLAR,UNC5A,BMF,CD14,TJP2,PSMF1,MAGED1,PSMD6,CDH1",Apoptosis,185

R-HSA-109582,"A1BG,SH2B3,KIF20A,RASGRP1,RASGRP1,CDK2,LHFPL2,CDK5,RASGRP2,RASGRP2,KCNMB2,ABCC4,MRVI1,HMG20B,TUBA1B,TUBB3,TUBB4A,TUBB4B,RAPGEF3,FAM3C,VAV3,VAV3,MERTK,CEACAM5,CAP1,VTI1B,PROCR,SH2B2,CENPE,PDPN,GNA13,GNA13,GNB5,GNB5,CFL1,KIF1C,CEACAM3,PDE10A,CEACAM8,MAGED2,EHD1,KIF2C,RAPGEF4,KIF3A,KIF3A,SLC7A9,AKAP10,TUBA3E,VPS45,KIF12,MGLL,TUBA3D,CLU,SERPINA3,JAML,KIF19,H3C14,COL1A1,COL1A1,COL1A2,COL1A2,CD109,DGKK,APOOL,CRK,DOCK11,AAMP,GATA5,SIRPA,MAPK14,CSK,PIK3R6,KIF18B,KLC3,ADRA2A,ADRA2B,ADRA2C,CTSW,CXADR,DGKA,DGKB,DGKG,DGKH,DGKQ,ZFPM1,CFD,DOCK1,DOCK2,DOCK3,ECM1,EGF,AHSG,A2M,AKT1,ALB,F2,F2R,F2R,F2RL2,F2RL2,F3,F5,F7,F8,F9,F10,F11,F12,F13A1,F13B,FCER1G,JMJD1C,KIF6,DAGLB,FGA,FGB,ALDOA,FGG,FGR,VEGFD,MMRN1,KIFAP3,KIFAP3,HABP4,P2RX2,KDM1A,KIF21B,ENDOD1,KIF1B,FLNA,RCOR1,KIF13B,DOCK9,FN1,ZFPM2,SLC7A8,CBX5,PIK3R5,SLC16A8,SLC7A11,PPIL2,MAFF,MAFF,KIF4A,ABL1,FYN,LY6G6F,SIN3A,SH2B1,ABHD12,KIF26A,GAS6,GATA1,GATA2,GATA3,GATA4,GATA6,LAT,KCNMB3,BRPF3,GLG1,KCNMB4,GNA11,GNA11,GNA12,GNA12,GNA15,GNA15,GNAI1,GNAI2,GNAI3,GNAQ,GNAQ,GNAS,GNAS,GNB1,GNB1,GNB2,GNB2,GNB3,GNB3,GNG3,GNG3,GNG4,GNG4,GNG5,GNG5,GNG7,GNG7,GNG10,GNG10,GNG11,GNG11,GNGT1,GNGT1,GNGT2,GNGT2,GP1BA,GP1BB,GP5,GP9,GPC1,ANGPT1,ANGPT1,ANGPT2,KIF4B,GRB2,GRB7,GRB14,SCG3,RACGAP1,GUCY1A2,OLA1,VPREB3,GUCY1A1,GUCY1B1,GYPA,GYPB,GYPC,ANXA2,H3-3A,H3-3B,HBB,HBD,HBE1,HBG1,HBG2,SERPIND1,HDAC1,HDAC2,ANXA5,HGF,EHD3,EHD2,HRAS,HRG,HSPA5,H3C15,APLP2,APOA1,APOB,IFNA1,IFNA2,IFNA4,IFNA5,IFNA6,IFNA7,IFNA8,IFNA10,IFNA13,IFNA14,IFNA16,IFNA17,IFNA21,IFNB1,GNAT3,TUBB8,TUBB2B,IGF1,IGF2,APOH,APP,JCHAIN,IGLL1,INPP5D,ITGA6,IRF1,IRF2,ISLR,ITGA1,ITGA2,ITGA2B,ITGA3,ITGA4,ITGA5,ITGAL,ITGAM,ITGAV,ITGAX,ITGB1,ITGB1,ITGB2,ITGB3,ITIH3,ITIH4,ITPK1,ITPR1,ITPR2,ITPR3,JAK2,NHLRC2,KCNMA1,KCNMB1,KIF2A,KIF3C,KIF3C,KIF5A,KIF5B,KLKB1,KNG1,KLC1,KIF11,KIFC1,KIFC1,KIF25,KIF22,KRAS,RHOA,RHOB,L1CAM,RHOG,LAMP2,LCK,LCP2,LGALS3BP,LYN,EPCAM,ARRB1,ARRB2,MAFG,MAFG,MAG,CD99,MIF,MMP1,MPL,PSG8,MYB,SERPINC1,CEACAM6,NFE2,NFE2,ATP1B1,ATP1B2,ATP1B3,NOS1,NOS2,NOS3,ATP2A1,ATP2A2,ATP2A3,NRAS,ATP2B1,ATP2B2,ATP2B3,ATP2B4,OLR1,ORM1,ORM2,P2RX1,P2RX3,P2RX4,P2RX5,P2RX7,P2RY1,P2RY1,PAFAH2,SERPINE1,SERPINB2,AK3,F11R,PDE11A,PCDH7,SERPINA5,SCCPDH,GP6,CLEC1B,PHF21A,PDE1A,TEX264,ANGPT4,PDE2A,PDE9A,PDE1B,PDGFA,PDGFB,FAM49B,PDPK1,PDPK1,CYB5R1,CD244,PECAM1,GNG13,GNG13,TUBA8,PF4,PF4V1,PFN1,SERPINA1,SERPINA4,SERPINB6,SERPINE2,SERPINB8,PIK3CA,PIK3CB,PIK3CG,PIK3R1,PIK3R2,PLA2G4A,PLAT,PLAU,PLAUR,PLCG1,PLCG2,PLG,PLEK,SERPINF2,TREM1,GNG2,GNG2,TMX3,APBB1IP,GTPBP2,KIF1A,PPBP,PPIA,PPIA,TOR4A,KIF26B,PPP2CA,PPP2CB,PPP2R1A,PPP2R1B,PPP2R5A,PPP2R5B,PPP2R5C,PPP2R5D,PPP2R5E,SIRPG,PRCP,SRGN,KIF27,CARMIL1,KIF21A,KIF16B,DOCK10,PRKACA,CDC37L1,MFN1,PRKACB,PRKACG,PRKAR1A,PRKAR1B,PRKAR2A,PRKAR2B,PRKCA,PRKCB,PRKCD,PRKCE,PRKCG,PRKCH,PRKCQ,PRKCZ,PRKG1,PRKG1,PRKG2,PRKG2,MAPK1,MAPK3,GNG12,GNG12,PROC,PROS1,SLC7A10,PRTN3,PSAP,PSG1,PSG2,PSG3,PSG4,PSG5,PSG6,PSG7,PSG9,PSG11,KIF15,KIF15,TRPC7,CD177,PTGIR,PTGIR,ABHD6,PTK2,DOCK6,QSOX1,PTPN1,PTPN6,PTPN11,JAM2,RAB5A,RAB27B,RAC1,RAC2,RAD51C,RAD51B,RAF1,RAP1A,RAP1B,RARRES2,GNB4,GNB4,ACTB,S100A10,CEACAM1,SDC1,SDC2,SDC4,SELE,SELL,SELP,SELPLG,SELENOP,RBSN,KIF9,KIF9,SHC1,MICAL1,P2RY12,KLC2,SLC3A2,H3C13,SLC8A2,SLC8A1,SLC8A3,SLC16A1,CHID1,SOD1,SOS1,SPARC,SPN,SPP2,SRC,SRI,STIM1,STX4,STXBP2,STXBP3,BSG,SYK,SYK,TBXA2R,TEK,TEK,TF,TFPI,TGFB1,TGFB2,TGFB3,LEFTY2,THBD,THBS1,THPO,TIMP1,TIMP3,C1QBP,TLN1,SERPING1,TSPAN7,TMSB4X,CLEC3B,TP53,TRPC3,TRPC6,TTN,TUBA4A,TUBA3C,TUBB2A,VAV1,VAV1,VAV2,VAV2,VCL,VEGFA,VEGFB,VEGFC,VPREB1,VWF,WEE1,DAGLA,YES1,YWHAZ,LRP8,TUBA1A,MANF,PCYOX1L,MAFK,MAFK,TUBAL3,DOCK5,CALM1,ORAI2,CALM2,MPIG6B,CALM3,ACTN4,TUBB1,CALU,SLC7A5,AKAP1,DOCK8,CABLES2,KIF18A,KIF18A,CAPZA1,CAPZA1,CAPZA2,CAPZA2,CAPZB,CAPZB,H3C1,H3C4,H3C3,H3C6,H3C11,H3C8,H3C12,H3C10,H3C2,CD99L2,JAM3,FERMT3,FCAMR,TAGLN2,TUBB6,KIF2B,TUBA1C,ORAI1,PIK3R3,ITGA10,DGKZ,DGKE,DGKD,DOCK7,CAV1,PDE5A,ACTN1,TNFRSF10D,TNFRSF10B,TNFRSF10A,ACTN2,CD84,H3C7,KLC4,F2RL3,F2RL3,DOK2,SLC7A7,SLC7A6,ESAM,KIFC2,SLC16A3,P2RX6,CD2,DGKI,CABLES1,CD9,KIF3B,KIF3B,SYTL4,GNG8,GNG8,PICK1,CD36,KIF23,BCAR1,KIF20B,CD44,CD47,CD48,GNA14,GNA14,CD58,CD63,SDC3,CD74,DOCK4,PHACTR2,MFN2,WDR1,CDC42",Hemostasis,680

R-HSA-109606,"AKT3,BCL2L11,CDKN2A,YWHAQ,DYNLL2,E2F1,AKT1,AKT2,CARD8,PPP1R13B,BBC3,SFN,GZMB,APAF1,APAF1,XIAP,XIAP,NMT1,APIP,PMAIP1,SEPTIN4,CYCS,CYCS,UACA,PPP3CC,PPP3R1,MAPK1,MAPK3,MAPK8,DIABLO,DIABLO,AVEN,BAD,BAD,BAK1,BAX,BCL2,BCL2L1,BID,STAT3,TFDP1,TFDP2,C1QBP,TP53,TP53BP2,TP73,YWHAB,YWHAE,YWHAG,YWHAH,YWHAZ,CASP3,CASP3,CASP7,CASP8,CASP9,CASP9,TP63,DYNLL1,BMF",Intrinsic Pathway for Apoptosis,60

R-HSA-109703,"AKT2,PDE3B,PDE3B",PKB-mediated events,3

R-HSA-109704,"FRS2,THEM4,KLB,AKT2,AKT2,FGF1,FGF2,FGF3,FGF4,FGF5,FGF6,FGF7,FGF8,FGF9,FGF10,FGFR1,FGFR3,FGFR2,FGFR4,FLT3,FLT3LG,GAB1,FGF20,FGF22,GRB2,PIK3R4,IRS1,PDE3B,PDE3B,PDPK1,PIK3C3,PIK3CA,PIK3CB,PIK3R1,PIK3R2,TLR9,TRIB3,PTPN11,FGF23,IRS2,FGF18,FGF17,FGF16,KL,GAB2,FGF19",PI3K Cascade,46

R-HSA-110056,"IL6,IL6R,IL6ST,JAK1,JAK2,MAPK3,MAP2K1,PTPN11,TYK2,CDK1",MAPK3 (ERK1) activation,10

R-HSA-110312,"MAD2L2,PCNA,REV1,REV3L,RFC1,RFC2,RFC3,RFC4,RFC5,RPA1,RPA2,RPA3,RPS27A,UBA52,UBB,UBC",Translesion synthesis by REV1,16

R-HSA-110313,"MAD2L2,POLD3,POLI,USP43,RCHY1,PCNA,POLK,REV1,POLE3,POLD1,POLD2,POLE,POLE2,POLH,NPLOC4,POLE4,POLD4,REV3L,RFC1,RFC2,RFC3,RFC4,RFC5,RPA1,RPA2,RPA3,RPS27A,UBA52,UBB,UBC,UBA7,UFD1,VCP,TRIM25,SPRTN,USP10,UBE2L6,ISG15,PCLAF",Translesion synthesis by Y family DNA polymerases bypasses lesions on DNA template,39

R-HSA-110314,"POLD3,DDB1,PCNA,DTL,POLE3,POLD1,POLD2,POLE,POLE2,POLE4,RAD18,WDR48,POLD4,RFC1,RFC2,RFC3,RFC4,RFC5,RPA1,RPA2,RPA3,RPS27A,UBA52,UBB,UBC,UBE2B,USP1,CUL4B,CUL4A,RBX1",Recognition of DNA damage by PCNA-containing replication complex,30

R-HSA-110320,"RCHY1,PCNA,POLH,NPLOC4,RFC1,RFC2,RFC3,RFC4,RFC5,RPA1,RPA2,RPA3,RPS27A,UBA52,UBB,UBC,UFD1,VCP,SPRTN",Translesion Synthesis by POLH,19

R-HSA-110328,"LOC102724334,H4-16,H2BU1,SMUG1,NEIL2,H2BC1,POT1,TINF2,H2AC8,H2AC7,H2AX,H2AZ1,H2BC5,H2BC3,H2AB1,NTHL1,OGG1,TERF2IP,NEIL3,H4C15,H2AJ,ACD,TDG,TERF1,TERF2,H2AC19,UNG,NEIL1,H3-4,H4C9,H2AC14,H2AC6,H2AC4,H2AC18,H2AC20,H2BC8,H2BC13,H2BC15,H2BC14,H2BC7,H2BC6,H2BC9,H2BC10,H2BC4,H2BC17,H2BC21,H4C1,H4C4,H4C6,H4C12,H4C11,H4C3,H4C8,H4C2,H4C5,H4C13,H4C14,H2BC12,MBD4,H2BC11,H2AZ2",Recognition and association of DNA glycosylase with site containing an affected pyrimidine,61

R-HSA-110329,"LOC102724334,H4-16,H2BU1,SMUG1,NEIL2,H2BC1,POT1,TINF2,H2AC8,H2AC7,H2AX,H2AZ1,H2BC5,H2BC3,H2AB1,NTHL1,OGG1,TERF2IP,NEIL3,H4C15,H2AJ,ACD,TDG,TERF1,TERF2,H2AC19,UNG,NEIL1,H3-4,H4C9,H2AC14,H2AC6,H2AC4,H2AC18,H2AC20,H2BC8,H2BC13,H2BC15,H2BC14,H2BC7,H2BC6,H2BC9,H2BC10,H2BC4,H2BC17,H2BC21,H4C1,H4C4,H4C6,H4C12,H4C11,H4C3,H4C8,H4C2,H4C5,H4C13,H4C14,H2BC12,MBD4,H2BC11,H2AZ2",Cleavage of the damaged pyrimidine ,61

R-HSA-110330,"LOC102724334,LOC102724334,H4-16,H4-16,H2BU1,H2BU1,H2BC1,H2BC1,POT1,POT1,TINF2,TINF2,H2AC8,H2AC8,H2AC7,H2AC7,H2AX,H2AX,H2AZ1,H2AZ1,H2BC5,H2BC5,H2BC3,H2BC3,MPG,MUTYH,H2AB1,H2AB1,OGG1,TERF2IP,TERF2IP,NEIL3,NEIL3,H4C15,H4C15,H2AJ,H2AJ,ACD,ACD,TERF1,TERF1,TERF2,TERF2,H2AC19,H2AC19,H3-4,H3-4,H4C9,H4C9,H2AC14,H2AC14,H2AC6,H2AC6,H2AC4,H2AC4,H2AC18,H2AC18,H2AC20,H2AC20,H2BC8,H2BC8,H2BC13,H2BC13,H2BC15,H2BC15,H2BC14,H2BC14,H2BC7,H2BC7,H2BC6,H2BC6,H2BC9,H2BC9,H2BC10,H2BC10,H2BC4,H2BC4,H2BC17,H2BC17,H2BC21,H2BC21,H4C1,H4C1,H4C4,H4C4,H4C6,H4C6,H4C12,H4C12,H4C11,H4C11,H4C3,H4C3,H4C8,H4C8,H4C2,H4C2,H4C5,H4C5,H4C13,H4C13,H4C14,H4C14,H2BC12,H2BC12,H2BC11,H2BC11,H2AZ2,H2AZ2",Recognition and association of DNA glycosylase with site containing an affected purine,109

R-HSA-110331,"LOC102724334,LOC102724334,H4-16,H4-16,H2BU1,H2BU1,H2BC1,H2BC1,POT1,POT1,TINF2,TINF2,H2AC8,H2AC8,H2AC7,H2AC7,H2AX,H2AX,H2AZ1,H2AZ1,H2BC5,H2BC5,H2BC3,H2BC3,MPG,MUTYH,H2AB1,H2AB1,OGG1,TERF2IP,TERF2IP,NEIL3,NEIL3,H4C15,H4C15,H2AJ,H2AJ,ACD,ACD,TERF1,TERF1,TERF2,TERF2,H2AC19,H2AC19,H3-4,H3-4,H4C9,H4C9,H2AC14,H2AC14,H2AC6,H2AC6,H2AC4,H2AC4,H2AC18,H2AC18,H2AC20,H2AC20,H2BC8,H2BC8,H2BC13,H2BC13,H2BC15,H2BC15,H2BC14,H2BC14,H2BC7,H2BC7,H2BC6,H2BC6,H2BC9,H2BC9,H2BC10,H2BC10,H2BC4,H2BC4,H2BC17,H2BC17,H2BC21,H2BC21,H4C1,H4C1,H4C4,H4C4,H4C6,H4C6,H4C12,H4C12,H4C11,H4C11,H4C3,H4C3,H4C8,H4C8,H4C2,H4C2,H4C5,H4C5,H4C13,H4C13,H4C14,H4C14,H2BC12,H2BC12,H2BC11,H2BC11,H2AZ2,H2AZ2",Cleavage of the damaged purine,109

R-HSA-110357,"SMUG1,APEX1,MPG,MUTYH,NTHL1,OGG1,TDG,UNG,MBD4",Displacement of DNA glycosylase by APEX1,9

R-HSA-110362,"PARP2,PARP1,FEN1,APEX1,LIG1,POLB,ADPRHL2,BPHL,PARG",POLB-Dependent Long Patch Base Excision Repair,9

R-HSA-110373,"PARP2,POLD3,PARP1,FEN1,APEX1,LIG1,PCNA,POLE3,POLB,POLD1,POLD2,POLE,POLE2,ADPRHL2,POLE4,POLD4,RFC1,RFC2,RFC3,RFC4,RFC5,RPA1,RPA2,RPA3,BPHL,PARG",Resolution of AP sites via the multiple-nucleotide patch replacement pathway,26

R-HSA-110381,"APEX1,LIG3,POLB,XRCC1",Resolution of AP sites via the single-nucleotide replacement pathway,4

R-HSA-111367,"LSM11,NCBP2,ZNF473,NCBP1,SNRPB,SNRPD3,SNRPE,SNRPF,SNRPG,LSM10",SLBP independent Processing of Histone Pre-mRNAs,10

R-HSA-111446,"BCL2L11,MAPK8,DYNLL1",Activation of BIM and translocation to mitochondria ,3

R-HSA-111447,"AKT3,YWHAQ,AKT1,AKT2,SFN,PPP3CC,PPP3R1,BAD,BAD,BCL2,BID,YWHAB,YWHAE,YWHAG,YWHAH,YWHAZ",Activation of BAD and translocation to mitochondria ,16

R-HSA-111448,"E2F1,PMAIP1,TFDP1,TFDP2,TP53",Activation of NOXA and translocation to mitochondria,5

R-HSA-111452,"BAK1,BID",Activation and oligomerization of BAK protein,2

R-HSA-111453,"BCL2L11,BBC3,PMAIP1,BAD,BCL2,BCL2L1,BID,STAT3,BMF",BH3-only proteins associate with and inactivate anti-apoptotic BCL-2 members,9

R-HSA-111457,"SEPTIN4,CYCS,DIABLO,BAK1,BAX",Release of apoptotic factors from the mitochondria,5

R-HSA-111458,"CARD8,APAF1,APAF1,XIAP,APIP,CYCS,CYCS,UACA,MAPK1,MAPK3,DIABLO,AVEN,CASP9,CASP9",Formation of apoptosome,14

R-HSA-111459,"APAF1,XIAP,CYCS,CASP3,CASP7,CASP9",Activation of caspases through apoptosome-mediated cleavage,6

R-HSA-111461,"CARD8,APAF1,APAF1,XIAP,APIP,CYCS,CYCS,UACA,MAPK1,MAPK3,DIABLO,AVEN,CASP3,CASP7,CASP9,CASP9",Cytochrome c-mediated apoptotic response,16

R-HSA-111463,"APAF1,XIAP,XIAP,CYCS,DIABLO,DIABLO,CASP3,CASP7,CASP9",SMAC (DIABLO) binds to IAPs ,9

R-HSA-111464,"APAF1,XIAP,XIAP,CYCS,DIABLO,DIABLO,CASP3,CASP7,CASP9",SMAC(DIABLO)-mediated dissociation of IAP:caspase complexes ,9

R-HSA-111465,"OCLN,BCAP31,ADD1,CTNNB1,DSG1,DSG2,DSG3,DSP,ACIN1,FNTA,GAS2,DBNL,GSN,APC,BIRC2,LMNA,LMNB1,MAPT,STK26,PKP1,PLEC,PRKCD,PRKCQ,PTK2,ROCK1,SATB1,CLSPN,BMX,SPTAN1,TJP1,VIM,CASP3,CASP6,CASP7,CASP8,STK24,TJP2,CDH1",Apoptotic cleavage of cellular proteins,38

R-HSA-111469,"APAF1,XIAP,XIAP,SEPTIN4,CYCS,DIABLO,DIABLO,CASP3,CASP3,CASP7,CASP9","SMAC, XIAP-regulated apoptotic response",11

R-HSA-111471,"CDKN2A,CARD8,APAF1,APAF1,XIAP,XIAP,APIP,SEPTIN4,CYCS,CYCS,UACA,MAPK1,MAPK3,DIABLO,DIABLO,AVEN,BAK1,BAX,C1QBP,CASP3,CASP3,CASP7,CASP9,CASP9",Apoptotic factor-mediated response,24

R-HSA-111885,"CDK5,CAMKK2,CAMKK2,GNB5,ADCY1,ADCY1,AHCYL1,ADCY2,ADCY2,ADCY3,ADCY3,ADCY5,ADCY5,ADCY6,ADCY6,ADCY7,ADCY7,ADCY8,ADCY8,ADCY9,ADCY9,CREB1,GRK2,ADCY4,ADCY4,PLCB1,PLCB1,NBEA,GNAI1,GNAI1,GNAI2,GNAI2,GNAI3,GNAI3,GNAL,GNAO1,GNAO1,GNAT1,GNAT1,GNAT2,GNAT2,GNAZ,GNAZ,GNB1,GNB2,GNB3,GNG3,GNG4,GNG5,GNG7,GNG10,GNG11,GNGT1,GNGT2,GNAT3,GNAT3,ITPR1,ITPR2,ITPR3,KPNA2,OPRM1,PDE1A,PDE1C,PDE4A,PDE4B,PDE4C,PDE4D,PDE1B,PDYN,GNG13,PLA2G4A,PLCB2,PLCB2,PLCB3,PLCB3,PLCB4,PLCB4,GNG2,POMC,PPP1CA,PPP2CA,PPP2CB,PPP2R1A,PPP2R1B,PPP2R5D,PPP3CA,PPP3CB,PPP3CC,PPP3R1,PRKACA,PRKACA,PRKACB,PRKACB,PRKACG,PRKACG,PRKAR1A,PRKAR1B,PRKAR2A,PRKAR2A,PRKAR2B,PRKCA,PRKCD,PRKCG,MAPK1,GNG12,PRKX,GNB4,CALM1,CALM1,CALM2,CALM2,CALM3,CALM3,CAMK4,CAMK4,CAMK2A,CAMK2B,CAMK2D,CAMK2G,PPP1R1B,PPP1R1B,CAMKK1,CAMKK1,GNG8",Opioid Signalling,124

R-HSA-111931,"ADCY1,ADCY2,ADCY3,ADCY5,ADCY6,ADCY7,ADCY8,ADCY9,CREB1,ADCY4,NBEA,PRKACA,PRKACA,PRKACB,PRKACB,PRKACG,PRKACG,PRKAR1A,PRKAR1B,PRKAR2A,PRKAR2A,PRKAR2B,PRKX,CALM1,CALM2,CALM3",PKA-mediated phosphorylation of CREB,26

R-HSA-111932,"CAMKK2,CAMKK2,CREB1,KPNA2,CALM1,CALM1,CALM2,CALM2,CALM3,CALM3,CAMK4,CAMK4,CAMK2A,CAMK2B,CAMK2D,CAMK2G,CAMKK1,CAMKK1",CaMK IV-mediated phosphorylation of CREB,18

R-HSA-111933,"CAMKK2,CAMKK2,ADCY1,ADCY2,ADCY3,ADCY5,ADCY6,ADCY7,ADCY8,ADCY9,CREB1,GRK2,ADCY4,NBEA,KPNA2,PDE1A,PDE1C,PDE1B,PRKACA,PRKACA,PRKACB,PRKACB,PRKACG,PRKACG,PRKAR1A,PRKAR1B,PRKAR2A,PRKAR2A,PRKAR2B,PRKCA,PRKCD,PRKCG,PRKX,CALM1,CALM1,CALM2,CALM2,CALM3,CALM3,CAMK4,CAMK4,CAMK2A,CAMK2B,CAMK2D,CAMK2G,CAMKK1,CAMKK1",Calmodulin induced events,47

R-HSA-111957,"PDE1A,PDE1C,PDE1B,CALM1,CALM2,CALM3",Cam-PDE 1 activation,6

R-HSA-111995,"PLA2G4A,MAPK1",phospho-PLA2 pathway,2

R-HSA-111996,"CAMKK2,CAMKK2,ADCY1,ADCY2,ADCY3,ADCY5,ADCY6,ADCY7,ADCY8,ADCY9,CREB1,GRK2,ADCY4,NBEA,KPNA2,PDE1A,PDE1C,PDE1B,PLA2G4A,PRKACA,PRKACA,PRKACB,PRKACB,PRKACG,PRKACG,PRKAR1A,PRKAR1B,PRKAR2A,PRKAR2A,PRKAR2B,PRKCA,PRKCD,PRKCG,MAPK1,PRKX,CALM1,CALM1,CALM2,CALM2,CALM3,CALM3,CAMK4,CAMK4,CAMK2A,CAMK2B,CAMK2D,CAMK2G,CAMKK1,CAMKK1",Ca-dependent events,49

R-HSA-111997,"CAMKK2,CAMKK2,ADCY1,ADCY2,ADCY3,ADCY5,ADCY6,ADCY7,ADCY8,ADCY9,CREB1,GRK2,ADCY4,NBEA,KPNA2,PDE1A,PDE1C,PDE1B,PRKACA,PRKACA,PRKACB,PRKACB,PRKACG,PRKACG,PRKAR1A,PRKAR1B,PRKAR2A,PRKAR2A,PRKAR2B,PRKCA,PRKCD,PRKCG,PRKX,CALM1,CALM1,CALM2,CALM2,CALM3,CALM3,CAMK4,CAMK4,CAMK2A,CAMK2B,CAMK2D,CAMK2G,CAMKK1,CAMKK1",CaM pathway,47

R-HSA-112040,"CAMKK2,CAMKK2,ADCY1,ADCY1,AHCYL1,ADCY2,ADCY2,ADCY3,ADCY3,ADCY5,ADCY5,ADCY6,ADCY6,ADCY7,ADCY7,ADCY8,ADCY8,ADCY9,ADCY9,CREB1,GRK2,ADCY4,ADCY4,PLCB1,PLCB1,NBEA,GNAI1,GNAI1,GNAI2,GNAI2,GNAI3,GNAI3,GNAL,GNAO1,GNAO1,GNAT1,GNAT1,GNAT2,GNAT2,GNAZ,GNAZ,GNAT3,GNAT3,ITPR1,ITPR2,ITPR3,KPNA2,PDE1A,PDE1C,PDE1B,PLA2G4A,PLCB2,PLCB2,PLCB3,PLCB3,PLCB4,PLCB4,PRKACA,PRKACA,PRKACB,PRKACB,PRKACG,PRKACG,PRKAR1A,PRKAR1B,PRKAR2A,PRKAR2A,PRKAR2B,PRKCA,PRKCD,PRKCG,MAPK1,PRKX,CALM1,CALM1,CALM2,CALM2,CALM3,CALM3,CAMK4,CAMK4,CAMK2A,CAMK2B,CAMK2D,CAMK2G,CAMKK1,CAMKK1",G-protein mediated events,87

R-HSA-112043,"CAMKK2,CAMKK2,ADCY1,AHCYL1,ADCY2,ADCY3,ADCY5,ADCY6,ADCY7,ADCY8,ADCY9,CREB1,GRK2,ADCY4,PLCB1,PLCB1,NBEA,GNAI1,GNAI1,GNAI2,GNAI2,GNAI3,GNAI3,GNAO1,GNAO1,GNAT1,GNAT1,GNAT2,GNAT2,GNAZ,GNAZ,GNAT3,GNAT3,ITPR1,ITPR2,ITPR3,KPNA2,PDE1A,PDE1C,PDE1B,PLA2G4A,PLCB2,PLCB2,PLCB3,PLCB3,PLCB4,PLCB4,PRKACA,PRKACA,PRKACB,PRKACB,PRKACG,PRKACG,PRKAR1A,PRKAR1B,PRKAR2A,PRKAR2A,PRKAR2B,PRKCA,PRKCD,PRKCG,MAPK1,PRKX,CALM1,CALM1,CALM2,CALM2,CALM3,CALM3,CAMK4,CAMK4,CAMK2A,CAMK2B,CAMK2D,CAMK2G,CAMKK1,CAMKK1",PLC beta mediated events,77

R-HSA-112122,ALKBH2,ALKBH2 mediated reversal of alkylation damage,1

R-HSA-112126,"ASCC3,ALKBH3,ASCC1,ASCC2",ALKBH3 mediated reversal of alkylation damage,4

R-HSA-112303,"GJC1,PANX1,PANX2,GJD2,GJA10",Electric Transmission Across Gap Junctions,5

R-HSA-112307,"GJC1,PANX1,PANX2,GJD2,GJA10",Transmission across Electrical Synapses ,5

R-HSA-112308,"CACNG2,CACNG4,CACNA2D3,CACNA1A,CACNA1B,CACNA1E,CACNA2D1,CACNB1,CACNB2,CACNB3,CACNB4,CACNA2D2",Presynaptic depolarization and calcium channel opening,12

R-HSA-112310,"UNC13B,ARL6IP5,CPLX1,CHAT,SLC32A1,ABAT,RIMS1,GAD1,GAD2,NAAA,GLS2,GLS,APBA1,HSPA8,MAOA,SLC38A2,LIN7C,SLC17A7,RAB3A,SLC5A7,LIN7B,SLC1A1,SLC1A2,SLC1A3,SLC1A6,SLC1A7,SLC6A1,SLC6A11,SLC6A12,SLC6A13,SLC18A2,SLC18A3,SLC22A1,SLC22A2,SNAP25,STX1A,STXBP1,VAMP2,SYN1,SYN2,SYT1,ALDH5A1,DNAJC5,SYN3,PPFIA4,PPFIA2,PPFIA1,PPFIA3,CASK,LIN7A,TSPOAP1",Neurotransmitter release cycle,51

R-HSA-112311,"COMT,ALDH2,LRTOMT,MAOA,ACHE,BCHE,SLC6A3,SLC6A4,SLC22A1,SLC22A2",Neurotransmitter clearance,10

R-HSA-112313,"GLUL,SLC1A2,SLC1A3,SLC38A1",Neurotransmitter uptake and metabolism In glial cells,4

R-HSA-112314,"CACNG3,CACNG2,TUBA1B,TUBB3,TUBB4A,TUBB4B,CAMKK2,CAMKK2,GNB5,ADCY1,ADCY1,ADCY2,ADCY2,ADCY3,ADCY3,ADCY5,ADCY5,ADCY6,ADCY6,TUBA3E,ADCY7,ADCY7,CHRNA1,TUBA3D,CHRNA2,CHRNA3,CHRNA4,CHRNA5,CHRNA7,ADCY8,ADCY8,CHRNB2,CHRNB3,CHRNB4,CHRND,CHRNE,CHRNG,ADCY9,ADCY9,GRIN3A,GRIN3A,GRIN3B,AP2M1,AP2S1,CREB1,CREB1,AP2A1,AP2A2,AP2B1,HTR3C,DLG1,DLG1,DLG2,DLG2,DLG3,DLG3,DLG4,DLG4,ADCY4,ADCY4,HTR3D,GABRR3,EPB41L1,ERBB4,PPM1E,ARHGEF9,ARHGEF9,PLCB1,GRIP1,GABBR1,GABRA1,GABRA1,GABRA2,GABRA2,GABRA3,GABRA3,GABRA4,GABRA4,GABRA5,GABRA5,GABRA6,GABRA6,GABRB1,GABRB1,GABRB2,GABRB2,GABRB3,GABRB3,GABRG2,GABRG2,GABRG3,GABRG3,GABRR1,GABRR2,NBEA,NPTN,CACNG4,RPS6KA6,RPS6KA6,GLRA1,GLRA1,GLRA2,GLRA2,GLRB,GLRB,GNAI1,GNAI1,GNAI2,GNAI2,GNAI3,GNAI3,GNAL,GNB1,GNB2,GNB3,GNG3,GNG4,GNG5,GNG7,GNG10,GNG11,GNGT1,GNGT2,HTR3E,GRIA1,GRIA1,GRIA2,GRIA2,GRIA3,GRIA3,GRIA4,GRIA4,GIT1,GRIK1,GRIK2,GRIK3,GRIK4,GRIK5,GRIN1,GRIN1,GRIN2A,GRIN2A,GRIN2B,GRIN2B,GRIN2C,GRIN2C,GRIN2D,GRIN2D,NRG1,APBA1,HRAS,HTR3A,GNAT3,GNAT3,TUBB8,TUBB2B,KCNJ2,KCNJ3,KCNJ4,KCNJ5,KCNJ6,KCNJ9,KCNJ10,KCNJ12,KCNJ15,KCNJ16,KPNA2,KRAS,MAPT,MDM2,MYO6,NEFL,NEFL,NRAS,NRGN,NRGN,NSF,PRKAG2,PDPK1,GNG13,TUBA8,PLCB2,PLCB3,PRKAG3,GNG2,LIN7C,CHRNA9,PRKAA1,PRKAA2,PRKAB1,PRKAB2,PRKACA,PRKACA,PRKACB,PRKACB,PRKACG,PRKACG,PRKAG1,PRKAR1A,PRKAR1B,PRKAR2A,PRKAR2B,PRKCA,PRKCB,PRKCG,GABRQ,GABRQ,MAPK1,MAPK3,GNG12,PRKX,KIF17,RAC1,RASGRF1,RASGRF2,CACNG8,GNB4,RPS6KA1,RPS6KA1,RPS6KA2,RPS6KA2,RPS6KA3,RPS6KA3,LIN7B,SRC,TSPAN7,TUBA4A,TUBA3C,TUBB2A,TUBA1A,TUBAL3,GLRA3,GLRA3,CALM1,CALM1,CALM2,CALM2,CALM3,CALM3,GRIP2,TUBB1,CAMK4,CAMK4,CAMK2A,CAMK2A,CAMK2B,CAMK2B,CAMK2D,CAMK2D,CAMK2G,CAMK2G,NCALD,CAMKK1,CAMKK1,TUBB6,TUBA1C,CAMK1,CAMK1,CASK,ACTN2,ACTN2,LIN7A,ARHGEF7,CHRNA6,CHRFAM7A,HTR3B,GNG8,PICK1,AKAP5,GABBR2,PPM1F",Neurotransmitter receptors and postsynaptic signal transmission,271

R-HSA-112315,"CACNG3,CACNG2,TUBA1B,TUBB3,TUBB4A,TUBB4B,UNC13B,ARL6IP5,CAMKK2,CAMKK2,GNB5,ADCY1,ADCY1,ADCY2,ADCY2,CPLX1,ADCY3,ADCY3,CHAT,ADCY5,ADCY5,ADCY6,ADCY6,TUBA3E,ADCY7,ADCY7,CHRNA1,TUBA3D,CHRNA2,CHRNA3,CHRNA4,CHRNA5,CHRNA7,ADCY8,ADCY8,CHRNB2,CHRNB3,CHRNB4,CHRND,CHRNE,CHRNG,ADCY9,ADCY9,GRIN3A,GRIN3A,GRIN3B,AP2M1,AP2S1,COMT,CREB1,CREB1,SLC32A1,AP2A1,AP2A2,AP2B1,HTR3C,DLG1,DLG1,DLG2,DLG2,DLG3,DLG3,DLG4,DLG4,ABAT,ADCY4,ADCY4,HTR3D,GABRR3,EPB41L1,ERBB4,ALDH2,LRTOMT,PPM1E,RIMS1,ARHGEF9,ARHGEF9,PLCB1,GRIP1,GABBR1,GABRA1,GABRA1,GABRA2,GABRA2,GABRA3,GABRA3,GABRA4,GABRA4,GABRA5,GABRA5,GABRA6,GABRA6,GABRB1,GABRB1,GABRB2,GABRB2,GABRB3,GABRB3,GABRG2,GABRG2,GABRG3,GABRG3,GABRR1,GABRR2,GAD1,GAD2,NBEA,NPTN,CACNG4,NAAA,GLS2,RPS6KA6,RPS6KA6,GLRA1,GLRA1,GLRA2,GLRA2,GLRB,GLRB,GLS,GLUL,GNAI1,GNAI1,GNAI2,GNAI2,GNAI3,GNAI3,GNAL,GNB1,GNB2,GNB3,GNG3,GNG4,GNG5,GNG7,GNG10,GNG11,GNGT1,GNGT2,HTR3E,GRIA1,GRIA1,GRIA2,GRIA2,GRIA3,GRIA3,GRIA4,GRIA4,GIT1,GRIK1,GRIK2,GRIK3,GRIK4,GRIK5,GRIN1,GRIN1,GRIN2A,GRIN2A,GRIN2B,GRIN2B,GRIN2C,GRIN2C,GRIN2D,GRIN2D,NRG1,APBA1,APBA1,HRAS,HSPA8,HTR3A,GNAT3,GNAT3,TUBB8,TUBB2B,KCNJ2,KCNJ3,KCNJ4,KCNJ5,KCNJ6,KCNJ9,KCNJ10,KCNJ12,KCNJ15,KCNJ16,KPNA2,KRAS,MAOA,MAPT,MDM2,ACHE,MYO6,NEFL,NEFL,NRAS,NRGN,NRGN,NSF,PRKAG2,PDPK1,GNG13,TUBA8,PLCB2,PLCB3,PRKAG3,GNG2,SLC38A2,LIN7C,LIN7C,CHRNA9,PRKAA1,PRKAA2,PRKAB1,PRKAB2,PRKACA,PRKACA,PRKACB,PRKACB,PRKACG,PRKACG,PRKAG1,PRKAR1A,PRKAR1B,PRKAR2A,PRKAR2B,PRKCA,PRKCB,CACNA2D3,PRKCG,GABRQ,GABRQ,MAPK1,MAPK3,GNG12,PRKX,SLC17A7,KIF17,RAB3A,RAC1,BCHE,RASGRF1,RASGRF2,CACNG8,GNB4,SLC5A7,RPS6KA1,RPS6KA1,RPS6KA2,RPS6KA2,RPS6KA3,RPS6KA3,LIN7B,LIN7B,SLC1A1,SLC1A2,SLC1A3,SLC1A6,SLC1A7,SLC6A1,SLC6A3,SLC6A4,SLC6A11,SLC6A12,SLC6A13,SLC18A2,SLC18A3,SLC22A1,SLC22A2,SNAP25,SRC,STX1A,STXBP1,VAMP2,SYN1,SYN2,SYT1,TSPAN7,TUBA4A,TUBA3C,TUBB2A,CACNA1A,CACNA1B,CACNA1E,CACNA2D1,CACNB1,CACNB2,CACNB3,TUBA1A,CACNB4,ALDH5A1,TUBAL3,GLRA3,GLRA3,CALM1,CALM1,DNAJC5,CALM2,CALM2,CALM3,CALM3,GRIP2,TUBB1,CAMK4,CAMK4,CAMK2A,CAMK2A,SLC38A1,CAMK2B,CAMK2B,CAMK2D,CAMK2D,CAMK2G,CAMK2G,SYN3,NCALD,CAMKK1,CAMKK1,TUBB6,TUBA1C,PPFIA4,PPFIA2,PPFIA1,CAMK1,CAMK1,PPFIA3,CASK,CASK,ACTN2,ACTN2,LIN7A,LIN7A,ARHGEF7,CHRNA6,CHRFAM7A,HTR3B,CACNA2D2,TSPOAP1,GNG8,PICK1,AKAP5,GABBR2,PPM1F",Transmission across Chemical Synapses,341

R-HSA-112316,"HCN4,GJC1,ABCC9,KCNK7,FLOT1,KCNMB2,RTN3,CACNG3,CACNG2,TUBA1B,TUBB3,TUBB4A,TUBB4B,UNC13B,ARL6IP5,PDLIM5,CAMKK2,CAMKK2,GNB5,ADCY1,ADCY1,ADCY2,ADCY2,CPLX1,ADCY3,ADCY3,CHAT,ADCY5,ADCY5,IL1RAPL1,ADCY6,ADCY6,TUBA3E,ADCY7,ADCY7,CHRNA1,TUBA3D,CHRNA2,CHRNA3,CHRNA4,CHRNA5,CHRNA7,ADCY8,ADCY8,CHRNB2,CHRNB3,CHRNB4,CHRND,CHRNE,CHRNG,SLITRK1,ADCY9,ADCY9,GRIN3A,GRIN3A,GRIN3B,AP2M1,AP2S1,SYT2,KCNH8,COMT,CREB1,CREB1,SLITRK4,SLC32A1,SYT9,AP2A1,AP2A2,AP2B1,KCNV2,HTR3C,KCNG3,DLG1,DLG1,DLG2,DLG2,DLG3,DLG3,DLG4,DLG4,ABAT,ADCY4,ADCY4,HTR3D,GABRR3,EPB41,EPB41L1,EPB41L2,ERBB4,ALDH2,LRTOMT,NLGN4Y,NLGN4Y,DLGAP4,DLGAP4,PPM1E,SLITRK3,NLGN1,NLGN1,SHANK2,SHANK2,RIMS1,EPB41L3,FLOT2,ARHGEF9,ARHGEF9,PLCB1,KCNH4,KCNH3,GRIP1,PANX1,GABBR1,GABRA1,GABRA1,GABRA2,GABRA2,GABRA3,GABRA3,GABRA4,GABRA4,GABRA5,GABRA5,GABRA6,GABRA6,GABRB1,GABRB1,GABRB2,GABRB2,GABRB3,GABRB3,GABRG2,GABRG2,GABRG3,GABRG3,GABRR1,GABRR2,GAD1,GAD2,SIPA1L1,LRRTM2,SLITRK5,KCNG2,IL1RAPL2,NBEA,KCNV1,NPTN,CACNG4,KCNMB3,KCNH5,NAAA,GLS2,RPS6KA6,RPS6KA6,KCNMB4,GLRA1,GLRA1,GLRA2,GLRA2,GLRB,GLRB,GLS,GLUL,GNAI1,GNAI1,GNAI2,GNAI2,GNAI3,GNAI3,GNAL,GNB1,GNB2,GNB3,GNG3,GNG4,GNG5,GNG7,GNG10,GNG11,GNGT1,GNGT2,HTR3E,GRIA1,GRIA1,GRIA2,GRIA2,GRIA3,GRIA3,GRIA4,GRIA4,GIT1,GRIK1,GRIK2,DBNL,GRIK3,GRIK4,GRIK5,GRIN1,GRIN1,GRIN2A,GRIN2A,GRIN2B,GRIN2B,GRIN2C,GRIN2C,GRIN2D,GRIN2D,GRM1,GRM1,GRM5,GRM5,NRG1,APBA1,APBA1,APBA2,HRAS,HSPA8,HTR3A,KCNK18,SYT10,GNAT3,GNAT3,TUBB8,LRRTM1,LRRTM3,TUBB2B,HCN1,IL1RAP,KCNA1,KCNA2,KCNA3,KCNA4,KCNA5,KCNA6,KCNA7,KCNA10,KCNB1,KCNC1,KCNC2,KCNC3,KCNC4,KCND1,KCND2,KCND3,KCNF1,KCNG1,KCNH1,KCNH2,KCNJ1,KCNJ2,KCNJ3,KCNJ4,KCNJ5,KCNJ6,KCNJ8,KCNJ9,KCNJ10,KCNJ11,KCNJ12,KCNJ14,KCNJ15,KCNJ16,KCNK1,KCNK2,KCNK3,KCNMA1,KCNMB1,KCNN1,KCNN2,KCNN3,KCNN4,KCNQ1,KCNQ2,KCNQ3,KCNS1,KCNS2,KCNS3,KPNA2,KRAS,MAOA,MAPT,MDM2,ACHE,MYO6,NEFL,NEFL,NRAS,NRGN,NRGN,NSF,NTRK3,KCNK4,SHANK1,SHANK1,KCNK9,PRKAG2,PDPK1,GNG13,TUBA8,PLCB2,PLCB3,PRKAG3,KCNK10,GNG2,SLC38A2,NLGN3,NLGN3,LIN7C,LIN7C,CHRNA9,PRKAA1,PRKAA2,PRKAB1,PRKAB2,PRKACA,PRKACA,PRKACB,PRKACB,PRKACG,PRKACG,PRKAG1,PRKAR1A,PRKAR1B,PRKAR2A,PRKAR2B,PRKCA,PRKCB,CACNA2D3,PRKCG,GABRQ,GABRQ,MAPK1,MAPK3,GNG12,PRKX,KCNQ5,KCNK13,PANX2,SLC17A7,GJD2,LRFN2,NLGN4X,NLGN4X,NLGN2,NLGN2,KIF17,BEGAIN,LRFN1,HCN3,EPB41L5,PTPRD,PTPRF,PTPRS,DLGAP3,DLGAP3,RAB3A,RAC1,BCHE,RASGRF1,RASGRF2,CACNG8,GNB4,SLC5A7,HCN2,RPS6KA1,RPS6KA1,RPS6KA2,RPS6KA2,RPS6KA3,RPS6KA3,LIN7B,LIN7B,SLC1A1,SLC1A2,SLC1A3,SLC1A6,SLC1A7,SLC6A1,SLC6A3,SLC6A4,SLC6A11,SLC6A12,SLC6A13,SLC18A2,SLC18A3,SLC22A1,SLC22A2,SNAP25,SRC,STX1A,STX1A,STXBP1,STXBP1,ABCC8,VAMP2,SYN1,SYN2,SYT1,SYT1,TSPAN7,TUBA4A,TUBA3C,TUBB2A,CACNA1A,CACNA1B,CACNA1E,CACNA2D1,CACNB1,CACNB2,CACNB3,TUBA1A,CACNB4,KCNAB1,LRFN4,ALDH5A1,LRFN3,TUBAL3,GLRA3,GLRA3,LRRTM4,CALM1,CALM1,DNAJC5,CALM2,CALM2,CALM3,CALM3,GRIP2,TUBB1,KCNH6,CAMK4,CAMK4,CAMK2A,CAMK2A,SLC38A1,CAMK2B,CAMK2B,CAMK2D,CAMK2D,CAMK2G,CAMK2G,SHARPIN,SYN3,KCNK16,NCALD,SLITRK6,CAMKK1,CAMKK1,TUBB6,SLITRK2,GJA10,TUBA1C,PPFIBP2,PPFIBP1,PPFIA4,PPFIA2,PPFIA1,KCNAB2,CAMK1,CAMK1,PPFIA3,CASK,CASK,ACTN2,ACTN2,LIN7A,LIN7A,ARHGEF7,CHRNA6,KCNK17,CHRFAM7A,KCNH7,SYT7,KCNQ4,SYT12,HTR3B,KCNAB3,DLGAP1,DLGAP1,CACNA2D2,TSPOAP1,KCNG4,KCNB2,NRXN3,NRXN3,NRXN1,NRXN1,NRXN2,NRXN2,LRRC4B,GNG8,KCNK6,HOMER3,HOMER3,HOMER2,HOMER2,HOMER1,HOMER1,PICK1,AKAP5,APBA3,GABBR2,PPM1F",Neuronal System,502

R-HSA-112382,"ELOA3D,CDK7,CDK9,SUPT16H,LEO1,ELOA3,ERCC2,ERCC3,NCBP2,RTF1,NELFB,AFF4,GTF2F1,GTF2F2,GTF2H1,GTF2H2,GTF2H3,GTF2H4,GTF2H5,MLLT1,MLLT3,MNAT1,NCBP1,ELOA2,NELFCD,POLR2A,POLR2B,POLR2C,POLR2D,POLR2E,POLR2F,POLR2G,POLR2H,POLR2I,POLR2J,POLR2K,POLR2L,PAF1,IWS1,EAF2,SSRP1,SUPT4H1,SUPT5H,SUPT6H,TCEA1,ELOC,ELOB,ELOA,ELOA3B,NELFA,NELFE,CDC73,WDR61,ELL,EAF1,CCNK,CCNH,CCNT1,CCNT2,CTDP1,CTR9",Formation of RNA Pol II elongation complex ,61

R-HSA-112399,"FRS2,THEM4,KLB,AKT2,AKT2,FGF1,FGF2,FGF3,FGF4,FGF5,FGF6,FGF7,FGF8,FGF9,FGF10,FGFR1,FGFR3,FGFR2,FGFR4,FLT3,FLT3LG,GAB1,FGF20,FGF22,GRB2,GRB2,PIK3R4,HRAS,IRS1,IRS1,KRAS,NRAS,PDE3B,PDE3B,PDPK1,PIK3C3,PIK3CA,PIK3CB,PIK3R1,PIK3R2,TLR9,TRIB3,PTPN11,SOS1,SOS1,FGF23,IRS2,IRS2,FGF18,FGF17,FGF16,KL,GAB2,FGF19",IRS-mediated signalling,54

R-HSA-112409,"DUSP10,DUSP1,DUSP2,DUSP4,DUSP5,DUSP6,DUSP7,DUSP8,DUSP9,IL6,IL6R,IL6ST,JAK1,JAK2,MAPK1,MAPK3,MAP2K1,MAP2K2,PTPN11,TYK2,DUSP16,PEA15,CDK1",RAF-independent MAPK1/3 activation,23

R-HSA-112411,"IL6,IL6R,IL6ST,JAK1,JAK2,MAPK1,MAP2K2,PTPN11,TYK2",MAPK1 (ERK2) activation,9

R-HSA-112412,"GRB2,GRB2,HRAS,IRS1,IRS1,KRAS,NRAS,SOS1,SOS1,IRS2,IRS2",SOS-mediated signalling,11

R-HSA-113418,"CDK7,ERCC2,ERCC3,NCBP2,NELFB,GTF2F1,GTF2F2,GTF2H1,GTF2H2,GTF2H3,GTF2H4,GTF2H5,MNAT1,NCBP1,NELFCD,POLR2A,POLR2B,POLR2C,POLR2D,POLR2E,POLR2F,POLR2G,POLR2H,POLR2I,POLR2J,POLR2K,POLR2L,SUPT4H1,SUPT5H,NELFA,NELFE,CCNH,CTDP1",Formation of the Early Elongation Complex,33

R-HSA-113501,"E2F1,POLA2,PPP2R3B,POLA1,PPP2CA,PPP2CB,PPP2R1A,PPP2R1B,PRIM1,PRIM2,RB1,RB1,TFDP1,TFDP2",Inhibition of replication initiation of damaged DNA by RB1/E2F1,14

R-HSA-113507,"ORC6,ORC3,ORC1,ORC2,ORC4,ORC5,MCM8,CCNB1,CDK1",E2F-enabled inhibition of pre-replication complex formation,9

R-HSA-113510,"E2F1,ORC6,ORC3,POLA2,PPP2R3B,ORC1,ORC2,ORC4,ORC5,POLA1,PPP2CA,PPP2CB,PPP2R1A,PPP2R1B,PRIM1,PRIM2,RB1,RB1,TFDP1,TFDP2,MCM8,CCNB1,CDK1",E2F mediated regulation of DNA replication,23

R-HSA-114294,"BAX,BID","Activation, translocation and oligomerization of BAX",2

R-HSA-114452,"AKT3,BCL2L11,YWHAQ,DYNLL2,E2F1,AKT1,AKT2,PPP1R13B,BBC3,SFN,PMAIP1,PPP3CC,PPP3R1,MAPK8,BAD,BAD,BCL2,BID,TFDP1,TFDP2,TP53,TP53BP2,TP73,YWHAB,YWHAE,YWHAG,YWHAH,YWHAZ,TP63,DYNLL1,BMF",Activation of BH3-only proteins,31

R-HSA-114508,"RASGRP1,RASGRP2,MGLL,DGKK,DGKA,DGKB,DGKG,DGKH,DGKQ,DAGLB,ABHD12,ITPR1,ITPR2,ITPR3,PRKCD,PRKCE,PRKCH,PRKCQ,TRPC7,ABHD6,TRPC3,TRPC6,DAGLA,DGKZ,DGKE,DGKD,DGKI",Effects of PIP2 hydrolysis,27

R-HSA-114516,"PRKCA,PRKCB,PRKCG,STX4,STXBP3",Disinhibition of SNARE formation,5

R-HSA-114604,"VAV3,VAV3,PDPN,COL1A1,COL1A2,PIK3R6,FCER1G,PIK3R5,FYN,LAT,RHOA,RHOB,RHOG,LCK,LCP2,LYN,GP6,CLEC1B,PDPK1,PDPK1,PIK3CA,PIK3CB,PIK3CG,PIK3R1,PIK3R2,PLCG2,PRKCZ,PTPN6,PTPN11,RAC1,RAC2,SYK,SYK,VAV1,VAV1,VAV2,VAV2,MPIG6B,PIK3R3,CDC42",GPVI-mediated activation cascade,40

R-HSA-114608,"A1BG,LHFPL2,ABCC4,FAM3C,CAP1,VTI1B,CFL1,MAGED2,CLU,SERPINA3,CD109,APOOL,CTSW,CFD,ECM1,EGF,AHSG,A2M,ALB,F5,F8,F13A1,FGA,FGB,ALDOA,FGG,VEGFD,MMRN1,HABP4,ENDOD1,FLNA,FN1,LY6G6F,GAS6,BRPF3,SCG3,OLA1,ANXA5,HGF,HRG,HSPA5,APLP2,APOA1,IGF1,IGF2,APOH,APP,ISLR,ITGA2B,ITGB3,ITIH3,ITIH4,NHLRC2,KNG1,LAMP2,LGALS3BP,ORM1,ORM2,SERPINE1,PCDH7,SCCPDH,TEX264,PDGFA,PDGFB,FAM49B,CYB5R1,PECAM1,PF4,PFN1,SERPINA1,SERPINA4,PLG,PLEK,SERPINF2,TMX3,GTPBP2,PPBP,PPIA,TOR4A,SRGN,CDC37L1,PROS1,PSAP,QSOX1,RAB27B,RARRES2,SELP,SELENOP,CHID1,SOD1,SPARC,SPP2,STXBP2,TF,TGFB1,TGFB2,TGFB3,LEFTY2,THBS1,TIMP1,TIMP3,TLN1,SERPING1,TMSB4X,CLEC3B,TTN,TUBA4A,VCL,VEGFA,VEGFB,VEGFC,VWF,MANF,PCYOX1L,CALM1,CALM2,CALM3,ACTN4,CALU,FERMT3,TAGLN2,ACTN1,ACTN2,CD9,SYTL4,CD36,CD63,PHACTR2,WDR1",Platelet degranulation ,129

R-HSA-1168372,"RASGRP1,RASGRP1,PSME3,PSMD14,MALT1,MALT1,CHUK,CHUK,PSMB11,PSMA8,FKBP1A,PSME4,FBXW11,RASGRP3,RASGRP3,HRAS,IKBKB,IKBKB,KRAS,NFATC1,NFATC1,NFATC2,NFATC2,NFATC3,NFATC3,NFKB1,NFKBIA,NFKBIB,NFKBIE,NRAS,PPIA,PPP3CA,PPP3CA,PPP3CB,PPP3CB,PPP3R1,PPP3R1,PRKCB,PSMA1,PSMA2,PSMA3,PSMA4,PSMA5,PSMA6,PSMA7,PSMB1,PSMB2,PSMB3,PSMB4,PSMB5,PSMB6,PSMB7,PSMB8,PSMB9,PSMB10,PSMC1,PSMC2,PSMC3,PSMC4,PSMC5,PSMC6,PSMD1,PSMD2,PSMD3,PSMD4,PSMD5,PSMD7,PSMD8,PSMD9,PSMD10,PSMD11,PSMD12,PSMD13,PSME1,PSME2,REL,RELA,RPS27A,SKP1,MAP3K7,UBA52,UBB,UBC,SEM1,CALM1,CALM1,CALM2,CALM2,CALM3,CALM3,CARD11,CARD11,CUL1,IKBKG,IKBKG,BCL10,BCL10,BTRC,PSMF1,PSMD6",Downstream signaling events of B Cell Receptor (BCR),100

R-HSA-1169091,"PSME3,PSMD14,MALT1,MALT1,CHUK,CHUK,PSMB11,PSMA8,PSME4,FBXW11,IKBKB,IKBKB,NFKB1,NFKBIA,NFKBIB,NFKBIE,PRKCB,PSMA1,PSMA2,PSMA3,PSMA4,PSMA5,PSMA6,PSMA7,PSMB1,PSMB2,PSMB3,PSMB4,PSMB5,PSMB6,PSMB7,PSMB8,PSMB9,PSMB10,PSMC1,PSMC2,PSMC3,PSMC4,PSMC5,PSMC6,PSMD1,PSMD2,PSMD3,PSMD4,PSMD5,PSMD7,PSMD8,PSMD9,PSMD10,PSMD11,PSMD12,PSMD13,PSME1,PSME2,REL,RELA,RPS27A,SKP1,MAP3K7,UBA52,UBB,UBC,SEM1,CARD11,CARD11,CUL1,IKBKG,IKBKG,BCL10,BCL10,BTRC,PSMF1,PSMD6",Activation of NF-kappaB in B cells,73

R-HSA-1169092,"RASGRP1,RASGRP1,RASGRP3,RASGRP3,HRAS,KRAS,NRAS",Activation of RAS in B cells,7

R-HSA-1169408,"NUP50,NUP42,USP18,NUP35,EIF4A1,EIF4A2,EIF4E,EIF4G1,EIF4G2,NUP205,FLNB,NUP210,NUP160,NUP188,DDX58,NUP62,ARIH1,EIF4E3,IFIT1,NUP43,IRF3,JAK1,KPNA1,KPNB1,KPNA2,KPNA3,KPNA4,KPNA5,KPNA7,MX1,MX2,NEDD4,NUP88,NUP98,HERC5,PIN1,PLCG1,NUP54,PPM1B,NDC1,NUP133,MAPK3,EIF2AK2,NUP107,RANBP2,RPS27A,SEC13,STAT1,TPR,UBA52,UBB,UBC,UBA7,UBE2E1,UBE2N,TRIM25,NUP37,NUP85,NUP214,AAAS,SEH1L,RAE1,EIF4G3,UBE2L6,EIF4E2,NUP155,ISG15,NUP93,EIF4A3,NUP58,POM121,NUP153",ISG15 antiviral mechanism,72

R-HSA-1169410,"NUP50,NUP42,USP18,NUP35,EIF4A1,EIF4A2,EIF4E,EIF4G1,EIF4G2,PDE12,FLNA,NUP205,FLNB,NUP210,NUP160,NUP188,DDX58,NUP62,ARIH1,EIF4E3,IFIT1,NUP43,IRF3,JAK1,KPNA1,KPNB1,KPNA2,KPNA3,KPNA4,KPNA5,KPNA7,MX1,MX2,NEDD4,NUP88,NUP98,OAS1,OAS2,OAS3,HERC5,PIN1,PLCG1,NUP54,PPM1B,NDC1,NUP133,MAPK3,EIF2AK2,NUP107,RANBP2,RNASEL,ABCE1,RPS27A,SEC13,STAT1,TPR,UBA52,UBB,UBC,UBA7,UBE2E1,UBE2N,TRIM25,NUP37,NUP85,NUP214,AAAS,SEH1L,RAE1,OASL,EIF4G3,UBE2L6,EIF4E2,NUP155,ISG15,NUP93,EIF4A3,NUP58,POM121,NUP153",Antiviral mechanism by IFN-stimulated genes,80

R-HSA-1170546,"CSH1,CSH1,SH2B1,GH1,GH1,GH2,GH2,GHR,JAK2,JAK2,PRL,PRL,PRLR,PRLR,PTPN11,SKP1,STAT5A,STAT5B,CUL1,BTRC,RBX1",Prolactin receptor signaling,21

R-HSA-1181150,"DRAP1,LEFTY1,ACVR1C,DAND5,FOXO3,GDF1,SMAD2,SMAD2,SMAD3,SMAD3,SMAD4,NODAL,FURIN,PCSK6,CFC1,TDGF1,LEFTY2,FOXH1,ACVR1B,ACVR2A,ACVR2B,CER1",Signaling by NODAL,22

R-HSA-1187000,"ADAM30,IZUMO4,CATSPER1,CATSPER2,IZUMO2,KCNU1,ZP1,ADAM2,CATSPERD,B4GALT1,IZUMO1,CATSPER3,CATSPER4,ACR,OVGP1,CATSPERG,ZP4,SPAM1,ZP2,ZP3,CATSPERB,HVCN1,ADAM21,ADAM20,CD9",Fertilization,25

R-HSA-1221632,"LOC102724334,STAG1,SYCP2,STAG3,STAG2,DIDO1,H4-16,H2BU1,SYNE2,SYNE1,SUN1,H2BC1,SYCE2,SUN2,POT1,TINF2,SMC1B,H2AC8,H2AC7,H2AX,H2AZ1,H2BC5,H2BC3,HSPA2,LMNA,LMNB1,H2AB1,SYCP3,TERF2IP,ATR,H4C15,H2AJ,TEX12,RAD21,SYCE3,ACD,BRCA1,SYCP1,TERF1,TERF2,H2AC19,UBE2I,SMC1A,H3-4,H4C9,H2AC14,H2AC6,H2AC4,H2AC18,H2AC20,H2BC8,H2BC13,H2BC15,H2BC14,H2BC7,H2BC6,H2BC9,H2BC10,H2BC4,H2BC17,H2BC21,H4C1,H4C4,H4C6,H4C12,H4C11,H4C3,H4C8,H4C2,H4C5,H4C13,H4C14,FKBP6,H2BC12,H2BC11,SMC3,SYCE1,H2AZ2,REC8",Meiotic synapsis,79

R-HSA-1222449,LTF,Mtb iron assimilation by chelation,1

R-HSA-1222499,LTF,Latent infection - Other responses of Mtb to phagocytosis,1

R-HSA-1222556,"TCIRG1,ATP6V1G3,CYBA,CYBB,ATP6V0E2,ATP6V0A2,ATP6V0D2,ATP6V1C2,LPO,MPO,NCF2,NCF4,NOS1,NOS2,NOS3,ATP6V0A4,ATP6V1D,ATP6V1H,ATP6V1A,ATP6V1B1,ATP6V1B2,ATP6V0C,ATP6V1C1,ATP6V1E1,ATP6V0B,ATP6V1G2,ATP6V0A1,RAC2,NCF1,SLC11A1,HVCN1,ATP6V0E1,ATP6V1E2,ATP6V0D1,ATP6V1F,ATP6V1G1",ROS and RNS production in phagocytes,36

R-HSA-1226099,"FRS2,CPSF6,CNTRL,FGFR1OP,ERLIN2,CUX1,FGF1,FGF1,FGF2,FGF2,FGF3,FGF4,FGF5,FGF6,FGF7,FGF8,FGF9,FGF10,FGFR1,FGFR3,FGFR2,FGFR2,FGFR4,NCBP2,GAB1,FGFR1OP2,FGF20,FGF22,GRB2,GTF2F1,GTF2F2,HRAS,KRAS,MYO18A,NCBP1,NRAS,PIK3CA,PIK3R1,PLCG1,POLR2A,POLR2B,POLR2C,POLR2D,POLR2E,POLR2F,POLR2G,POLR2H,POLR2I,POLR2J,POLR2K,POLR2L,BCR,SOS1,STAT1,STAT3,STAT5A,STAT5B,ZMYM2,FGF23,TRIM24,FGF18,FGF17,FGF16,LRRFIP1,BAG4,GAB2",Signaling by FGFR in disease,66

R-HSA-1227986,"AKT3,RNF41,STUB1,NRG3,NRG3,CDC37,NRG4,NRG4,DIAPH1,HBEGF,HBEGF,EGF,EGF,EGFR,EGFR,ERBB2,ERBB2,ERBB3,ERBB3,ERBB4,ERBB4,EREG,EREG,AKT1,AKT2,FYN,GAB1,PTPN18,GRB2,GRB2,GRB7,NRG1,NRG1,HRAS,HRAS,HSP90AA1,KRAS,KRAS,RHOA,MATK,NRAS,NRAS,MEMO1,PIK3CA,PIK3R1,PLCG1,PRKCA,PRKCD,PRKCE,ERBIN,PTK6,PTPN12,PTPN12,RPS27A,SHC1,SHC1,SOS1,SOS1,SRC,BTC,BTC,UBA52,UBB,UBC,YES1,CUL5,USP8,NRG2,NRG2",Signaling by ERBB2,69

R-HSA-1227990,"NRG3,CDC37,NRG4,HBEGF,EGF,EGFR,ERBB2,ERBB3,ERBB4,EREG,GAB1,GRB2,NRG1,HRAS,HSP90AA1,KRAS,NRAS,PIK3CA,PIK3R1,PLCG1,ERBIN,PTPN12,SHC1,SOS1,BTC,NRG2",Signaling by ERBB2 in Cancer,26

R-HSA-1234158,"CITED2,CREBBP,EP300,EPAS1,EPO,HIGD1A,HIF1A,ARNT,HIF3A,VEGFA,CA9",Regulation of gene expression by Hypoxia-inducible Factor,11

R-HSA-1234174,"PSME3,PSMD14,CITED2,EGLN2,EGLN2,EGLN3,EGLN3,PSMB11,WTIP,WTIP,CREBBP,PSMA8,EP300,EPAS1,EPAS1,EPO,PSME4,HIGD1A,HIF1A,ARNT,EGLN1,EGLN1,HIF1AN,PSMA1,PSMA2,PSMA3,PSMA4,PSMA5,PSMA6,PSMA7,PSMB1,PSMB2,PSMB3,PSMB4,PSMB5,PSMB6,PSMB7,PSMB8,PSMB9,PSMB10,PSMC1,PSMC2,PSMC3,PSMC4,PSMC5,PSMC6,PSMD1,PSMD2,PSMD3,PSMD4,PSMD5,PSMD7,PSMD8,PSMD9,PSMD10,PSMD11,PSMD12,PSMD13,PSME1,PSME2,RPS27A,HIF3A,ELOC,ELOC,ELOB,ELOB,UBA52,UBB,UBC,UBE2D1,UBE2D2,UBE2D3,VEGFA,VHL,VHL,CA9,SEM1,CUL2,CUL2,AJUBA,AJUBA,LIMD1,LIMD1,PSMF1,PSMD6,RBX1,RBX1",Cellular response to hypoxia,87

R-HSA-1234176,"PSME3,PSMD14,EGLN2,EGLN2,EGLN3,EGLN3,PSMB11,WTIP,WTIP,PSMA8,EPAS1,EPAS1,PSME4,HIF1A,EGLN1,EGLN1,PSMA1,PSMA2,PSMA3,PSMA4,PSMA5,PSMA6,PSMA7,PSMB1,PSMB2,PSMB3,PSMB4,PSMB5,PSMB6,PSMB7,PSMB8,PSMB9,PSMB10,PSMC1,PSMC2,PSMC3,PSMC4,PSMC5,PSMC6,PSMD1,PSMD2,PSMD3,PSMD4,PSMD5,PSMD7,PSMD8,PSMD9,PSMD10,PSMD11,PSMD12,PSMD13,PSME1,PSME2,RPS27A,HIF3A,ELOC,ELOC,ELOB,ELOB,UBA52,UBB,UBC,UBE2D1,UBE2D2,UBE2D3,VHL,VHL,SEM1,CUL2,CUL2,AJUBA,AJUBA,LIMD1,LIMD1,PSMF1,PSMD6,RBX1,RBX1",Oxygen-dependent proline hydroxylation of Hypoxia-inducible Factor Alpha,78

R-HSA-1236382,"CDC37,EGF,EGFR,GAB1,GRB2,HRAS,HSP90AA1,KRAS,NRAS,PIK3CA,PIK3R1,PLCG1,RPS27A,SHC1,SOS1,UBA52,UBB,UBC,CBL",Constitutive Signaling by Ligand-Responsive EGFR Cancer Variants,19

R-HSA-1236394,"YAP1,MXD4,NRG3,NRG3,ADAP1,WWP1,CSN2,NRG4,NRG4,DLG4,HBEGF,HBEGF,EGF,EGFR,ERBB3,ERBB4,ERBB4,EREG,EREG,ESR1,TAB2,TAB2,NCSTN,GABRA1,GABRB1,GABRB2,GABRB3,GABRG2,GABRG3,GFAP,GRB2,NRG1,NRG1,HRAS,APOE,KRAS,STMN1,NEDD4,NRAS,APH1A,WWOX,PGR,PIK3CA,PIK3R1,PSENEN,GABRQ,PSEN1,PSEN2,RPS27A,S100B,CXCL12,SHC1,SOS1,SPARC,SRC,STAT5A,BTC,BTC,ADAM17,UBA52,UBB,UBC,APH1B,ITCH,NRG2,NRG2,NCOR1,NCOR1",Signaling by ERBB4,68

R-HSA-1236973,"CYBA,CYBB,ITGAV,ITGB5,NCF2,NCF4,NCF1,CD36",Cross-presentation of particulate exogenous antigens (phagosomes),8

R-HSA-1236974,"PSME3,PSMD14,TLR6,SEC61B,TIRAP,CHUK,PSMB11,PSMA8,PSME4,SEC61G,LY96,PDIA3,PDIA3,SEC61A1,HLA-A,HLA-A,HLA-B,HLA-B,HLA-C,HLA-C,HLA-E,HLA-E,HLA-F,HLA-F,HLA-G,HLA-G,IKBKB,MYD88,SEC61A2,B2M,B2M,PSMA1,PSMA2,PSMA3,PSMA4,PSMA5,PSMA6,PSMA7,PSMB1,PSMB2,PSMB3,PSMB4,PSMB5,PSMB6,PSMB7,PSMB8,PSMB9,PSMB10,PSMC1,PSMC2,PSMC3,PSMC4,PSMC5,PSMC6,PSMD1,PSMD2,PSMD3,PSMD4,PSMD5,PSMD7,PSMD8,PSMD9,PSMD10,PSMD11,PSMD12,PSMD13,PSME1,PSME2,RPS27A,STX4,TAP1,TAP1,TAP2,TAP2,TAPBP,BTK,TLR1,TLR2,TLR4,UBA52,UBB,UBC,SEM1,CALR,IKBKG,VAMP8,SNAP23,CD14,VAMP3,CD36,PSMF1,SEC22B,PSMD6",ER-Phagosome pathway,93

R-HSA-1236975,"PSME3,PSME3,PSMD14,PSMD14,TLR6,SEC61B,TIRAP,CHUK,PSMB11,PSMB11,PSMA8,PSMA8,CTSL,CTSV,CTSS,CYBA,CYBB,FCGR1A,FCGR1A,PSME4,PSME4,SEC61G,LY96,PDIA3,PDIA3,SEC61A1,HLA-A,HLA-A,HLA-B,HLA-B,HLA-C,HLA-C,HLA-E,HLA-E,HLA-F,HLA-F,HLA-G,HLA-G,IKBKB,ITGAV,ITGB5,LNPEP,MRC1,MRC1,MYD88,NCF2,NCF4,CD207,CD207,SEC61A2,B2M,B2M,PSMA1,PSMA1,PSMA2,PSMA2,PSMA3,PSMA3,PSMA4,PSMA4,PSMA5,PSMA5,PSMA6,PSMA6,PSMA7,PSMA7,PSMB1,PSMB1,PSMB2,PSMB2,PSMB3,PSMB3,PSMB4,PSMB4,PSMB5,PSMB5,PSMB6,PSMB6,PSMB7,PSMB7,PSMB8,PSMB8,PSMB9,PSMB9,PSMB10,PSMB10,PSMC1,PSMC1,PSMC2,PSMC2,PSMC3,PSMC3,PSMC4,PSMC4,PSMC5,PSMC5,PSMC6,PSMC6,PSMD1,PSMD1,PSMD2,PSMD2,PSMD3,PSMD3,PSMD4,PSMD4,PSMD5,PSMD5,PSMD7,PSMD7,PSMD8,PSMD8,PSMD9,PSMD9,PSMD10,PSMD10,PSMD11,PSMD11,PSMD12,PSMD12,PSMD13,PSMD13,PSME1,PSME1,PSME2,PSME2,RPS27A,NCF1,STX4,TAP1,TAP1,TAP2,TAP2,TAPBP,BTK,TLR1,TLR2,TLR4,UBA52,UBB,UBC,SEM1,SEM1,CALR,IKBKG,VAMP8,SNAP23,CD14,VAMP3,CD36,PSMF1,PSMF1,SEC22B,PSMD6,PSMD6,MRC2,MRC2",Antigen processing-Cross presentation,157

R-HSA-1236977,"CTSL,CTSV,CTSS,HLA-A,HLA-B,HLA-C,HLA-E,HLA-F,HLA-G,LNPEP,B2M",Endosomal/Vacuolar pathway,11

R-HSA-1236978,"PSME3,PSMD14,PSMB11,PSMA8,FCGR1A,FCGR1A,PSME4,MRC1,MRC1,CD207,CD207,PSMA1,PSMA2,PSMA3,PSMA4,PSMA5,PSMA6,PSMA7,PSMB1,PSMB2,PSMB3,PSMB4,PSMB5,PSMB6,PSMB7,PSMB8,PSMB9,PSMB10,PSMC1,PSMC2,PSMC3,PSMC4,PSMC5,PSMC6,PSMD1,PSMD2,PSMD3,PSMD4,PSMD5,PSMD7,PSMD8,PSMD9,PSMD10,PSMD11,PSMD12,PSMD13,PSME1,PSME2,SEM1,PSMF1,PSMD6,MRC2,MRC2",Cross-presentation of soluble exogenous antigens (endosomes),53

R-HSA-1237044,"HBA1,HBA2,HBB,AQP1,CYB5R4,CYB5R2,CYB5R1,RHAG,CYB5RL,SLC4A1,CA1,CA2,CA4",Erythrocytes take up carbon dioxide and release oxygen,13

R-HSA-1237112,"GOT1,MTAP,APIP,ADI1,ENOPH1,MRI1",Methionine salvage pathway,6

R-HSA-1247673,"HBA1,HBA2,HBB,AQP1,RHAG,SLC4A1,CA1,CA2,CA4",Erythrocytes take up oxygen and release carbon dioxide,9

R-HSA-1250196,"NRG3,NRG3,NRG4,NRG4,HBEGF,HBEGF,EGF,EGF,EGFR,EGFR,ERBB2,ERBB2,ERBB3,ERBB3,ERBB4,ERBB4,EREG,EREG,GRB2,NRG1,NRG1,HRAS,KRAS,NRAS,PRKCA,PRKCD,PRKCE,PTPN12,PTPN12,SHC1,SHC1,SOS1,BTC,BTC,NRG2,NRG2",SHC1 events in ERBB2 signaling,36

R-HSA-1250342,"NRG3,NRG4,HBEGF,ERBB4,EREG,NRG1,PIK3CA,PIK3R1,BTC,NRG2",PI3K events in ERBB4 signaling,10

R-HSA-1250347,"NRG3,NRG4,HBEGF,ERBB4,EREG,GRB2,NRG1,HRAS,KRAS,NRAS,SHC1,SOS1,BTC,NRG2",SHC1 events in ERBB4 signaling,14

R-HSA-1251932,"EGF,EGFR,ERBB2,PLCG1",PLCG1 events in ERBB2 signaling,4

R-HSA-1251985,"YAP1,MXD4,NRG3,ADAP1,CSN2,NRG4,HBEGF,ERBB4,ERBB4,EREG,ESR1,TAB2,TAB2,NCSTN,GFAP,NRG1,APOE,STMN1,APH1A,WWOX,PGR,PSENEN,PSEN1,PSEN2,S100B,CXCL12,SPARC,SRC,STAT5A,BTC,ADAM17,APH1B,NRG2,NCOR1,NCOR1",Nuclear signaling by ERBB4,35

R-HSA-1253288,"WWP1,ERBB4,NEDD4,RPS27A,SRC,UBA52,UBB,UBC,ITCH",Downregulation of ERBB4 signaling,9

R-HSA-1257604,"AKT3,AKT3,HDAC5,COMMD3-BMI1,COMMD3-BMI1,PSME3,PSMD14,CDKN1A,CDKN1B,STUB1,RRAGB,LAMTOR5,RRAGA,NRG3,FRS2,WWP2,CHD3,CHD3,CHD4,CHD4,CHUK,THEM4,PIK3AP1,PSMB11,CREB1,ATF2,ATF2,PSMA8,CSNK2A1,CSNK2A2,NRG4,CSNK2B,KLB,SLC38A9,ATN1,HBEGF,PHC1,PHC1,PHC2,PHC2,AGO3,AGO4,EGF,EGFR,EGR1,EGR1,ERBB2,ERBB3,ERBB4,EREG,AKT1,AKT1,AKT2,AKT2,ESR1,ESR2,MECOM,MECOM,EZH2,EZH2,FGF1,FGF2,FGF3,FGF4,FGF5,FGF6,FGF7,FGF8,FGF9,FGF10,FGFR1,FGFR3,FGFR2,FGFR4,SCMH1,SCMH1,KDM1A,PHLPP2,FOXO1,FOXO3,TNRC6B,RCOR1,PSME4,PHLPP1,OTUD3,PIP5K1C,CBX6,CBX6,SUZ12,SUZ12,MKRN1,FRK,MTOR,RICTOR,FYN,GAB1,EPGN,FGF20,AGO1,FGF22,AGO2,TNRC6A,GRB2,LAMTOR2,GSK3A,GSK3B,ICOS,HDAC1,HDAC1,HDAC2,HDAC2,HGF,NRG1,NR4A1,XIAP,IL1RAP,INS,INSR,IRAK1,IRS1,JUN,JUN,AREG,KIT,LAMTOR4,RHOG,LCK,MDM2,MET,KITLG,FOXO4,MOV10,MYD88,NEDD4,TRAT1,IRAK4,PDGFA,PDGFB,PDGFRA,HDAC7,PDGFRB,PDPK1,PIK3CA,PIK3CB,PIK3CD,PIK3R1,PIK3R2,PIP4K2A,MBD3,MBD3,PML,PPARG,PPARG,GATAD2A,GATAD2A,LAMTOR1,PPP2CA,PPP2CB,PPP2R1A,PPP2R1B,PPP2R5A,PPP2R5B,PPP2R5C,PPP2R5D,PPP2R5E,PRR5,MAPK1,MAPK3,PSMA1,PSMA2,PSMA3,PSMA4,PSMA5,PSMA6,PSMA7,PSMB1,PSMB2,PSMB3,PSMB4,PSMB5,PSMB6,PSMB7,PSMB8,PSMB9,PSMB10,PSMC1,PSMC2,PSMC3,PSMC4,PSMC5,PSMC6,PSMD1,PSMD2,PSMD3,PSMD4,PSMD5,PSMD7,PSMD8,PSMD9,PSMD10,SALL4,SALL4,PSMD11,PSMD12,PSMD13,BAD,PSME1,PSME2,PTEN,CBX8,CBX8,GATAD2B,GATAD2B,MTA3,MTA3,RPTOR,TNRC6C,TRIB3,PTPN11,RRAGD,RAC1,RAC2,RBBP4,RBBP4,RBBP7,RBBP7,REST,TRIM27,RHEB,RING1,RING1,RNF2,RNF2,RPS6KB2,RPS27A,RRAGC,MLST8,BMI1,BMI1,SNAI2,SNAI2,SNAI1,SNAI1,SRC,STRN,BTC,TGFA,NR2E1,TP53,TP53,TRAF6,TSC2,UBA52,UBB,UBC,VAV1,USP7,MAPKAP1,SEM1,PIP4K2C,PHC3,PHC3,PREX2,TNKS2,FGF23,RNF146,PIP5K1A,PIP5K1B,PIP4K2B,CASP9,MAF1,MAF1,AKT1S1,CBX2,CBX2,PIK3R3,CBX4,CBX4,LAMTOR3,TNKS,IRS2,EED,EED,FGF18,FGF17,FGF16,HDAC3,IER3,USP13,IL33,MTA1,MTA1,IL1RL1,MTA2,MTA2,CD19,KL,CD28,CD80,CD86,PSMF1,NRG2,PSMD6,FGF19",PIP3 activates AKT signaling,304

R-HSA-1266695,"CISH,H3C14,H3C14,HGF,H3C15,H3C15,IL2RG,IL2RG,IL7,IL7,IL7R,IL7R,IRS1,JAK1,JAK1,JAK3,JAK3,PIK3R1,PIK3R1,PIK3R2,PIK3R2,BRWD1,BRWD1,RAG1,RAG2,CRLF2,CRLF2,H3C13,H3C13,SMARCA4,STAT3,STAT3,STAT5A,STAT5A,STAT5B,STAT5B,H3C1,H3C1,H3C4,H3C4,H3C3,H3C3,H3C6,H3C6,H3C11,H3C11,H3C8,H3C8,H3C12,H3C12,H3C10,H3C10,H3C2,H3C2,PIK3R3,PIK3R3,TSLP,TSLP,SOCS1,IRS2,SOCS2,H3C7,H3C7",Interleukin-7 signaling,63

R-HSA-1266738,"CDH2,CDH2,AKT3,MED6,MED6,KRTAP25-1,KRTAP4-9,CD24,CDH4,CDH4,MED16,MED16,MEF2B,KRTAP21-3,MAMLD1,RANBP9,KRTAP9-7,KRTAP16-1,KRTAP9-6,KRTAP29-1,LOC100653049,ARPC5,ARPC4,ARPC3,ARPC1B,ACTR3,ACTR2,ARPC2,CDH15,CDH15,PLXNC1,CDK2,CDK4,PSME3,ADAM10,CDK5,CDK5,PSMD14,CDK8,CDK8,CDKN1A,LOC102724334,PAK4,CACNG3,CACNG2,SEMA3A,SEMA3A,TUBA1B,TUBA1B,TUBB3,TUBB3,TUBB4A,TUBB4A,TUBB4B,TUBB4B,MYL12B,MYL9,CDSN,YAP1,VAV3,CAP2,CAP1,CARM1,NCOA2,NCOA2,CEBPA,CEBPA,SEMA4D,CEBPB,CEBPB,CEBPD,CEBPE,ARPC1A,DPYSL4,DRAP1,MYL12A,MYL12A,LEFTY1,CTCF,BUB1B-PAK6,CFL1,CHL1,FRS2,PPARGC1A,PPARGC1A,RNPS1,ADIRF,SPINK5,WDR5,PKP3,KLK8,RPL35,TUBA3E,TUBA3E,STX1B,SCN11A,KRT71,MED8,MED8,LYPLA2,TUBA3D,TUBA3D,RPL39L,AGAP2,AP2M1,AP2M1,AP2S1,AP2S1,CLTA,CLTA,CLTB,CLTC,CLTC,KRT74,H4-16,PSMB11,KRT40,RPTN,H3C14,CNTN1,COL2A1,COL3A1,COL4A1,H2BU1,COL4A2,COL4A3,COL4A4,COL4A5,COL4A5,COL5A1,COL5A2,COL6A1,COL6A2,COL6A3,COL9A1,COL9A2,COL9A3,ACVR1C,COL6A6,UNC5D,CREB1,CREB1,CREBBP,CREBBP,CRMP1,RPS4Y2,KRTAP13-1,RPL10L,KRT72,LIPJ,MAPK14,MAPK14,PSMA8,CSF3R,KRT80,CSNK2A1,CSNK2A2,CSNK2B,NCAN,KRT25,DSG4,CSTA,HJV,LELP1,CTNNA1,CTNNA1,CTNNA2,CTNNA2,CTNNB1,CTNNB1,CYP51A1,AP2A1,AP2A1,DAB1,DAG1,DAG1,AP2A2,AP2A2,KRT28,AP2B1,AP2B1,DCC,DCC,LGI4,DCX,ZNF467,DLG1,DLG3,DLG4,DLG4,DNM1,DNM2,DOCK1,DOCK1,DOK1,DPYSL2,DPYSL3,DRP2,DSC1,DSC2,DSC3,DSCAM,DSCAM,DSG1,DSG2,DSG3,DSP,E2F1,E2F1,EBF1,KRT24,EFNA1,EFNA1,EFNA2,EFNA2,EFNA3,EFNA3,EFNA4,EFNA4,EFNA5,EFNA5,EFNB1,EFNB1,EFNB2,EFNB2,EFNB3,EFNB3,EGFR,EGR2,EGR2,KRT78,EPHA2,EPHA2,EIF4G1,DAND5,LCE4A,RPL22L1,LGI3,EP300,EP300,EPAS1,EPHA1,EPHA1,EPHA3,EPHA3,EPHA4,EPHA4,EPHA5,EPHA5,EPHA7,EPHA7,EPHA8,EPHA8,EPHB1,EPHB1,EPHB2,EPHB2,EPHB3,EPHB3,EPHB4,EPHB4,EPHB6,EPHB6,ERBB2,AKT1,AKT2,ETF1,EVPL,ALCAM,EZH2,FABP4,FABP4,MED19,MED19,UNC5B,DOK6,RFX6,FES,FES,FGF2,FGF10,FGFR1,FGFR1,CASC3,ABLIM3,NCBP2,SNW1,PAXIP1,USP33,ZSWIM8,NCOA6,NCOA6,FOXO1,FOXO1,FOXO3,CDK19,CDK19,NFASC,NFASC,FLG,CLASP2,PLXND1,FLI1,PSME4,KAZN,CLASP1,ARHGEF12,SRGAP2,NCSTN,MED13L,MED13L,PIP5K1C,SUZ12,RPL13A,CASP14,GSPT2,FLRT3,KIF4A,KIF4A,NR5A2,ABL1,ABL1,NR5A1,FYN,FYN,GAB1,LCE5A,KRTAP15-1,H2BC1,COL6A5,PTF1A,NGEF,NGEF,KLK5,KLK5,RPL36,WWTR1,GAP43,KRT23,UPF2,DNM3,KLK13,LCE2B,GATA2,GCK,KAT2A,NR6A1,GDF1,GDNF,GDNF,GFI1,GFRA1,GFRA1,GFRA2,GFRA2,GFRA3,PABPC1,ABL2,ABL2,FOXD3,FOXP1,CACNG4,CNTN6,RND1,RND1,RPS6KA6,ZNF638,GPC1,LAMA1,EPHA10,EPHA10,KRTAP13-4,EPHA6,EPHA6,KIF4B,KIF4B,RGMB,ANK1,ANK1,KRT6C,ANK2,ANK2,ANK3,ANK3,GRB2,GRB7,GRB10,GIT1,GIT1,GRIN1,GRIN1,GRIN2B,GRIN2B,MED4,MED4,ARHGAP35,GSK3B,GSPT1,SH3KBP1,H2AC8,H2AC7,H2AX,H2AZ1,H2BC5,H2BC3,H3-3A,H3-3B,HDAC2,HDAC2,HMGCR,FOXA2,FOXA3,HNF4A,HNF4G,ONECUT1,KRT73,HOXA1,HOXA2,HOXA3,HOXA4,HOXB1,HOXB2,HOXB3,HOXB4,HOXC4,HOXD1,HOXD3,HOXD4,HRAS,HES1,HSPA8,HSP90AA1,HSP90AB1,H3C15,IAPP,KRTAP8-1,KRTAP11-1,KRTAP19-1,KRTAP13-2,KRTAP13-3,KRTAP23-1,KRTAP6-1,KRTAP6-2,KRTAP19-2,KRTAP19-3,KRTAP19-4,KRTAP19-5,KRTAP19-6,KRTAP19-7,KRTAP20-1,KRTAP20-2,KRTAP21-1,KRTAP21-2,KRTAP22-1,KRT79,LIPM,KRT27,TUBB8,TUBB8,TUBB2B,TUBB2B,RBPJ,LCE1A,LCE1B,LCE1C,LCE1D,LCE1E,LCE1F,LCE2A,LCE2C,LCE2D,LCE3A,LCE3B,LCE3C,LCE3E,KRT26,KRTAP12-2,KRTAP12-1,KRTAP10-10,IL6R,INS,INSM1,PDX1,ISL1,ITGA1,ITGA2,ITGA2B,ITGA5,ITGA9,ITGAV,ITGB1,ITGB1,ITGB3,IVL,JUN,JUP,KRT77,AGRN,KCNQ2,KCNQ3,KRAS,KRTAP5-9,KRT1,KRT2,KRT3,KRT4,KRT5,KRT6A,KRT6B,KRT7,KRT8,KRT9,KRT10,KRT12,KRT13,KRT14,KRT15,KRTAP10-6,KRTAP10-7,KRTAP10-9,KRTAP10-1,KRTAP10-11,KRTAP10-2,KRTAP10-8,KRTAP10-3,KRTAP12-3,KRTAP12-4,KRTAP10-12,KRT16,RHOA,RHOA,KRT17,KRTAP5-1,KRTAP5-3,KRTAP5-4,KRTAP5-10,KRT18,RHOB,RHOB,KRT19,KRT31,KRT32,KRT33A,KRT33B,KRT34,KRT35,KRT81,KRT82,KRTAP26-1,KRT83,RHOC,RHOC,KRT84,KRT85,KRT86,MAFA,L1CAM,L1CAM,KRT39,LAMA2,ONECUT3,LAMB1,LAMC1,RPSA,LEP,ABLIM1,LIMK1,LIMK1,LIMK2,MED11,MED11,LORICRIN,LPL,SPINK6,LYN,LYN,SMAD2,SMAD2,SMAD3,SMAD3,SMAD4,MAG,MAGOH,MBP,ADAM11,MEF2A,BORCS8-MEF2B,MEF2C,MEF2D,MEIS1,MET,KMT2A,MMP2,MMP9,MPZ,KLK14,KLK12,KRTAP5-5,KRTAP5-2,KRTAP5-6,KRTAP5-7,KRTAP5-11,MSI1,MSN,MSN,LCE6A,MYB,MYC,MYF5,MYF5,MYF6,MYF6,MYH9,MYH10,MYH11,MYL6,MYO9B,MYO10,MYOD1,MYOD1,MYOG,MYOG,NAB1,NAB2,NCAM1,NCAM1,NCBP1,NCK1,NCK1,RPL10A,H2AB1,NELL2,NEO1,NEUROD1,NEUROD1,NFKB1,NKX2-2,NKX2-2,NKX6-1,NODAL,NOTCH1,NRAS,NRCAM,NRCAM,NRTN,NRTN,NTN3,FURIN,FURIN,PCSK6,PCSK6,COL5A3,PAK1,PAK1,PAK2,PAK2,PAK3,PAK3,NEUROG3,NEUROG3,PAX6,PBX1,PBX1,CDON,CDON,MED31,MED31,PCK1,RPS27L,APH1A,RPL26L1,ANGPTL4,LEF1,LEF1,SPTBN5,SPTBN5,KRT76,EVL,EVL,MED15,MED15,TUBA8,TUBA8,PFN1,PFN2,PI3,PIK3CA,PIK3CB,PIK3CD,PIK3R1,PIK3R2,PITPNA,PKLR,PKNOX1,PKP1,PKP2,PLCG1,PLCG1,SHC3,PLIN1,PLXNA1,PLXNA1,ADAM22,PLXNA2,PLXNA2,PLXNB1,PLXNB3,PML,PML,PMP22,TREM2,POLR2A,POLR2B,POLR2C,POLR2D,POLR2E,POLR2F,POLR2G,POLR2H,POLR2I,POLR2J,POLR2K,POLR2L,KRT20,POU3F1,POU3F2,POU5F1,PPARA,PPARG,PPARG,MED1,MED1,MED18,MED18,PPL,MED9,MED9,MAGOHB,LGI2,DPPA4,PPP3CB,H4C15,MAML3,PLXNA3,PLXNA3,MED29,MED29,PRKACA,PRKACA,PRKACB,PRKACB,PRKACG,PRKACG,DOK4,ENAH,ENAH,PRKAR2A,H2AJ,PRKCA,PRKCA,SCN3B,DOK5,PSENEN,PRKCQ,MAPK1,MAPK3,MAPK7,MAPK8,CFC1,MAPK11,MAPK11,MAPK13,MAP2K1,MAP2K2,MAP2K6,PRNP,PSPN,PSPN,RELN,PRSS8,PSEN1,PSEN2,PSMA1,PSMA2,PSMA3,PSMA4,PSMA5,PSMA6,PSMA7,PSMB1,DPYSL5,PSMB2,PSMB3,PSMB4,PAK6,PSMB5,PSMB6,PSMB7,LHX9,PSMB8,RGMA,PSMB9,PSMB10,PSMC1,PSMC2,PSMC3,PSMC4,PSMC5,PSMC6,PSMD1,PSMD2,PSMD3,PSMD4,PSMD5,TRPC7,PSMD7,PSMD8,PAK5,PSMD9,PSMD10,SALL4,PSMD11,PSMD12,PSMD13,PSME1,PSME2,ADGRG6,DSCAML1,PTK2,PTK2,SRGAP1,SEMA6A,SHTN1,PRX,PRX,SPTBN4,SPTBN4,PTPN11,PTPN11,KRTAP5-8,PTPRA,PTPRA,PTPRC,KMT2C,RAC1,RAC1,RAP1GAP,RARA,RARA,RARB,RARG,RASA1,RASA1,NTN4,RBBP4,CACNG8,RBBP5,RBBP7,RDX,RDX,RELA,RET,ACTB,ACTB,ROBO1,ROBO1,ROBO2,ROBO2,ROCK1,ROCK1,RPL3,RPL3L,RPL4,RPL5,RPL6,RPL7,RPL7A,RPL8,RPL9,RPL10,RPL11,RPL12,RPL13,RPL15,RPL17,RPL18,RPL18A,RPL19,RPL21,RPL22,RPL23A,RPL24,RPL26,RPL27,RPL30,RPL27A,RPL28,RPL29,RPL31,RPL32,RPL34,RPL35A,RPL36AL,RPL37,RPL37A,RPL38,RPL39,RPL41,RPL36A,RPLP0,RPLP1,RPLP2,RPS2,RPS3,RPS3A,RPS4X,RPS4Y1,RPS5,RPS6,RPS6KA1,RPS6KA2,RPS6KA3,RPS7,RPS8,RPS9,RPS10,RPS11,RPS12,RPS13,RPS14,RPS15,RPS15A,RPS16,RPS17,RPS18,RPS19,RPS20,RPS21,RPS23,RPS24,RPS25,RPS26,RPS27,RPS27A,RPS27A,RPS28,RPS29,RRAS,RRAS,RXRA,RXRA,SALL1,MAPK12,MAPK12,CELA2A,SCN1A,SCN1B,SCN2A,SCN2B,SCN3A,SCN4A,SCN4B,SCN5A,SCN7A,SCN8A,SCN9A,SCN10A,SDC2,SDCBP,CXCL12,ZNF335,PRDM14,PERP,GFRA4,GFRA4,SEMA4A,ROBO3,ROBO3,ARHGEF28,SPINK9,LIPK,LIPN,HIF3A,KRTAP24-1,KRTAP27-1,ITSN1,ITSN1,SH3GL2,SH3GL2,SHC1,SIAH1,SIAH2,UPF3B,UPF3A,SLC2A2,SLC2A4,KRTAP4-11,H3C13,SLIT1,SLIT3,SLIT3,SMARCA4,SMARCA4,SMARCD3,BNIP2,BNIP2,SOS1,SOS1,SOS2,SOX2,SOX10,SOX10,SPI1,SPI1,SPRR1A,SPRR1B,SPRR2A,SPRR2B,SPRR2D,SPRR2E,SPRR2F,SPRR2G,SPRR3,SPTA1,SPTA1,SPTAN1,SPTAN1,SPTB,SPTB,SPTBN1,SPTBN1,SPTBN2,SPTBN2,SRC,SRC,SREBF1,SREBF2,SREBF2,ST14,ST14,STAT3,STAT3,STX1A,MED22,MED22,KLF5,TAL1,CNTN2,TBL1X,ELOC,ELOB,TCF4,TCF4,HNF1A,HNF1B,TCF3,TCF3,TCF12,TCF12,TDGF1,TDGF1,TEAD1,NR2F2,TFDP1,TFDP1,TFDP2,TFDP2,TGFB1,LEFTY2,TGM1,TCHH,TIAM1,TLN1,ACTG1,ACTG1,TNF,TRIO,TRIO,TRPC1,TRPC3,TRPC4,TRPC5,TRPC6,H2AC19,TUBA4A,TUBA4A,TUBA3C,TUBA3C,TUBB2A,TUBB2A,KRTAP4-8,KRTAP1-4,KRTAP2-2,KRTAP19-8,KRTAP9-1,TYROBP,KRTAP2-3,UBA52,UBA52,UBB,UBB,UBC,UBC,UTRN,KDM6A,VASP,VASP,VAV2,EZR,EZR,VLDLR,WNT1,WNT10B,YES1,YES1,YY1,ZIC3,PCGF2,CACNA1C,CACNA1D,CACNA1S,CACNB1,CACNB2,CACNB3,TUBA1A,TUBA1A,CACNB4,CXCR4,ST8SIA4,DEK,DEK,PAGR1,TBL1XR1,LIN28A,MYH14,SEM1,TUBAL3,TUBAL3,NANOG,SCD5,SEMA6D,CHD9,LHX3,MED28,MED28,ARHGAP39,KMT2D,TUBB1,TUBB1,ST8SIA2,KRTAP1-3,KRTAP1-1,MED25,MED25,KRTAP9-9,KRTAP4-6,KRTAP2-1,NCOA3,NCOA3,CLTCL1,CAPN1,CAPNS1,H4C9,H2AC14,H2AC6,H2AC4,H2AC18,H2AC20,H2BC8,H2BC13,H2BC15,H2BC14,H2BC7,H2BC6,H2BC9,H2BC10,APH1B,H2BC4,H2BC17,H2BC21,H3C1,H3C4,H3C3,H3C6,H3C11,H3C8,H3C12,H3C10,H3C2,H4C1,H4C4,H4C6,H4C12,H4C11,H4C3,H4C8,H4C2,H4C5,H4C13,H4C14,KRTAP1-5,KRTAP3-1,KRTAP3-2,KRTAP9-2,KRTAP9-3,KRTAP9-8,KRTAP17-1,ADGRV1,MED10,MED10,NCK2,NCK2,MAML2,ABLIM2,FAM120B,FAM120B,CUL2,KRTAP4-4,TUBB6,TUBB6,LCE3D,TUBA1C,TUBA1C,SEMA7A,SEMA7A,ZSCAN10,AJUBA,PKP4,PIK3R3,CNTNAP1,ITGA10,H2BC12,KRTAP9-4,KRTAP4-1,KRTAP4-5,KRTAP4-3,KRTAP4-2,KRTAP3-3,KRTAP2-4,HELZ2,HELZ2,RUNX1,UNC5C,NCOA1,NCOA1,CBFB,NUMB,IRS2,KRT38,KRT37,KRT36,EED,ADAM23,NRP2,NRP1,NRP1,HDAC3,HDAC3,KAT2B,CDK5R1,CDK5R1,LDB1,ARHGEF7,CACNA1I,CACNA1H,CACNA1G,CCNC,CCNC,FOXH1,CCND3,H3C7,H2BC11,WASL,WASL,RPS6KA4,LHX4,KALRN,UNC5A,UNC5A,SEMA5A,MED30,MED30,SPAG9,SPAG9,RPL14,DOK2,ARTN,ARTN,PIAS2,ASH2L,ACVR1B,KRT75,CNOT9,PLXNA4,PLXNA4,BOC,BOC,ACVR2A,LGI1,RPS6KA5,RPS6KA5,PDLIM7,MED14,MED14,ACVR2B,KLF4,KLF4,TGM5,RPL23,CER1,SLIT2,SLIT2,LHX2,ADIPOQ,MED21,MED21,NTN1,NTN1,H2AZ2,MED23,MED23,MED17,MED17,MED26,MED26,MED27,MED27,MED7,MED7,ROCK2,ROCK2,MED20,MED20,CD36,PSMF1,AKAP5,AKAP5,NCOR1,NCOR1,NCOR2,NCOR2,TGS1,CD72,SEMA3E,EIF4A3,MAML1,ARHGEF11,GAB2,FARP2,FARP2,PSMD6,MED24,MED24,SRGAP3,MAFB,RBM8A,THRAP3,THRAP3,MED12,MED12,MED13,MED13,RBX1,CDC42,CDC42",Developmental Biology,1365

R-HSA-1268020,"TOMM6,CMC4,TIMM23,TIMM23,COX17,SLC25A13,TIMM17B,TIMM17B,TIMM17A,TIMM17A,TOMM40,TIMM44,PITRM1,MTX2,DNAJC19,CHCHD4,GRPEL2,CS,HSCB,HSCB,CYC1,TIMM8A,LDHD,LDHD,PMPCA,FXN,FXN,SAMM50,TIMM10B,TIMM13,TIMM10,TIMM10,TIMM9,TIMM9,TIMM8B,GFER,TIMM21,SLC25A4,SLC25A4,SLC25A6,SLC25A6,TIMM22,TIMM22,HSPA9,HSPD1,HSPD1,IDH3G,COA6,CHCHD10,TOMM5,MTX1,NDUFB8,NDUFB8,ATP5F1A,ACO2,OTC,OTC,ATP5F1B,ATP5F1B,PAM16,CHCHD2,COA4,ATP5MC1,ATP5MC1,TOMM7,CHCHD3,CMC2,TOMM22,BCS1L,TAZ,TAZ,VDAC1,CHCHD7,GRPEL1,CHCHD5,SLC25A12,COX19,TIMM50,PMPCB,TOMM20,TOMM70",Mitochondrial protein import,81

R-HSA-1280215,"RANBP9,CHURC1-FNTB,IL18BP,TRIM10,RASGRP1,EBI3,RASA4,PSME3,PSMD14,CNKSR1,CDKN1A,ADAR,TRIM22,IRF9,NOD1,IFITM3,LYPLA1,IFI30,TAB1,TRIM38,CEBPD,BATF,IFITM2,TRIM3,TNFSF13B,NRG3,CFL1,NUP50,FRS3,FRS2,EDAR,IL24,TRIM31,NUP42,IL1RAPL1,IRAK3,DUSP10,USP18,IL17F,CHUK,GBP4,GBP5,CISH,CISH,TNFRSF13C,RASGRP4,IL22RA2,THEM4,TRIM6,PSMB11,CCR1,CCR5,CNN2,H3C14,H3C14,CNTF,CNTFR,COL1A2,EDARADD,NUP35,IL17RE,MAP3K8,MAP3K8,IL31RA,CREB1,ATF2,CRK,CRKL,MAPK14,PSMA8,CSF1,CSF1R,CSF2,CSF2RA,CSF2RB,CSF2RB,CSF3,CSF3R,CSH1,CSH1,CSK,NRG4,IL34,CTF1,IL23R,IL23R,CTSG,PAQR3,KLB,DAB2IP,SPRED1,GBP6,IFNLR1,DLG1,DLG2,DLG3,DLG4,AGER,HBEGF,DUSP1,DUSP2,DUSP3,DUSP4,DUSP5,DUSP6,DUSP7,DUSP8,DUSP9,EDA,S1PR1,EGF,EGFR,EGR1,EIF4A1,EIF4A2,EIF4E,EIF4G1,EIF4G2,TXLNA,ELK1,SPRED2,PDE12,ERBB2,ERBB3,ERBB4,EREG,AKT1,AKT2,AKT2,F13A1,PTK2B,FCER2,FCGR1A,RASGEF1A,FGA,FGB,FGF1,FGF2,FGF3,FGF4,FGF5,FGF6,FGF7,FGF8,FGF9,FGF10,FGFR1,FGFR3,FGFR2,FGFR4,FGG,MRAS,RASA3,CNKSR2,FOXO1,TRIM35,FOXO3,TAB2,FLNA,NUP205,FLNB,RGL1,PSME4,FLT3,FLT3,NUP210,FLT3LG,FLT3LG,NUP160,FBXW11,TRIM2,FN1,FNTA,FNTB,ICMT,TNFRSF13B,NUP188,CLCF1,FOS,FPR1,DDX58,NUP62,TRIM29,IL17RA,ALOX5,IFIT5,ALOX15,PELI3,IL27,IL27,FYN,FYN,IFI6,GAB1,EPGN,TAB3,SHC2,RASGRP3,ARIH1,PTPN23,SAMHD1,SH2B1,PTPN20,GATA3,FGF20,GBP1,GBP2,GBP3,PTPN18,IL36RN,GDNF,GFRA1,GFRA2,GFRA3,GH1,GH1,GH2,GH2,GHR,GHR,FGF22,LAT,IL36B,IL37,IL36A,IL17C,PDCD4,IFNL2,IFNL3,IFNL1,KSR2,ANGPT1,NKIRAS2,NKIRAS1,GRB2,GRB2,RAPGEF1,LAMTOR2,GRIN1,GRIN2B,GRIN2D,TBK1,CXCL1,CXCL2,GSTA2,BLNK,IL19,ANXA1,ANXA2,HCK,HCK,HGF,HGF,NRG1,PIK3R4,HIF1A,HLA-A,HLA-B,HLA-C,HLA-DPA1,HLA-DPB1,HLA-DQA1,HLA-DQA2,HLA-DQB1,HLA-DQB2,HLA-DRA,HLA-DRB1,HLA-DRB3,HLA-DRB4,HLA-DRB5,HLA-E,HLA-F,HLA-G,HMGB1,HMOX1,EIF4E3,HNRNPA2B1,HNRNPF,HRAS,BIRC2,BIRC3,HSPA8,HSPA9,BIRC5,HSP90AA1,H3C15,H3C15,ICAM1,IRF8,IFI27,IFI35,IFIT2,IFIT1,IFIT3,IFNA1,IFNA1,IFNA2,IFNA2,IFNA4,IFNA4,IFNA5,IFNA5,IFNA6,IFNA6,IFNA7,IFNA7,IFNA8,IFNA8,IFNA10,IFNA10,IFNA13,IFNA13,IFNA14,IFNA14,IFNA16,IFNA16,IFNA17,IFNA17,IFNA21,IFNA21,IFNAR1,IFNAR1,IFNAR2,IFNAR2,IFNB1,IFNB1,IFNG,IFNG,IFNGR1,IFNGR1,IFNGR2,IFNGR2,NUP43,APP,IKBKB,IL1A,IL1B,IL1R1,IL1RAP,IL1RN,IL2,IL2RA,FASLG,IL2RB,IL2RB,IL2RG,IL2RG,IL3,IL3RA,IL4,IL4R,IL5,IL5RA,IL6,IL6R,IL6ST,IL6ST,IL7,IL7,IL7R,IL7R,CXCL8,IL9,IL9R,IL10,IL10RA,IL10RB,IL11,IL11RA,IL12A,IL12A,IL12B,IL12B,IL12RB1,IL12RB1,IL12RB2,IL12RB2,IL13,IL13RA1,IL13RA2,IL15,IL15,IL15RA,IL15RA,IL16,TNFRSF9,IL17A,IL18,CXCL10,INPP5D,INPPL1,IRAK1,IRAK2,IRF1,IRF2,IRF3,IRF4,IRF5,IRF6,IRF7,IRS1,IRS1,ISG20,ITGA2B,ITGAM,ITGAX,ITGB1,ITGB2,ARAF,ITGB3,JAK1,JAK1,JAK2,JAK2,JAK3,JAK3,JUN,JUNB,AREG,ARF1,USP17L2,KIT,KPNA1,KPNB1,KPNA2,KPNA3,KPNA4,KPNA5,KRAS,IL31,TMEM189-UBE2V1,CCL4L1,GBP7,LAMA5,LBP,LCK,LCN2,LCP1,LGALS9,LIF,LIFR,SPRED3,LMNB1,ARL2,KPNA7,LTA,LTA,LTB,LTB,LTBR,LTBR,LYN,LYN,TNFSF12-TNFSF13,ARRB1,SMAD3,ARRB2,MAOA,MARK3,CCL3L3,MCL1,MEF2A,MEF2C,MAP3K3,MET,KITLG,CIITA,MID1,MIF,MAP3K11,MMP1,MMP2,MMP3,MMP9,MSN,MT2A,MTAP,MUC1,MX1,MX2,MYC,MYD88,ATF1,NCAM1,NDN,NEDD4,NEFL,NF1,NFKB1,NFKB2,NFKBIA,NFKBIB,NOS2,NRAS,NRTN,NUP88,NUP98,OAS1,OAS2,OAS3,TNFRSF11B,OPRD1,OPRM1,OSM,P4HB,PEBP1,SERPINB2,IL20,IL21R,IL22,PAK2,ABHD17B,ZDHHC9,GOLGA7,TRIM17,IRAK4,HERC5,VRK3,TNFRSF12A,SPTBN5,PDE3B,PDE3B,IP6K2,PDE6D,PDGFA,PDGFB,PDGFRA,IL23A,IL23A,PDGFRB,PDPK1,PHB,PIK3C3,PIK3CA,PIK3CA,PIK3CB,PIK3CB,PIM1,PIK3CD,PIK3CD,PIK3R1,PIK3R1,PIK3R2,PIK3R2,PIN1,PITPNA,PLCG1,SHC3,NUP54,PML,IL20RA,IL20RB,TRIM34,BRWD1,BRWD1,TLR9,POMC,TOLLIP,POU2F1,APBB1IP,XAF1,IL17RD,PPIA,PPM1B,PPP1CB,PPP1CC,TRIM68,PPP2CA,PPP2CB,PPP2R1A,PPP2R1B,TRIM62,PPP2R5A,PPP2R5B,PPP2R5C,PPP2R5D,PPP2R5E,BOLA2,PPP5C,IL17RB,PRKACA,NDC1,NUP133,PRKCD,IL26,PRKCQ,PRKG2,MAPK1,MAPK1,MAPK3,MAPK3,MAPK7,MAPK7,MAPK8,MAPK11,MAPK9,MAPK10,MAP2K1,MAP2K1,MAP2K2,MAP2K3,MAP2K6,MAP2K7,EIF2AK2,PRL,PRL,PRLR,PRLR,PSPN,IL36G,PRTN3,B2M,PSMA1,PSMA2,PSMA3,PSMA4,PSMA5,PSMA6,PSMA7,PSMB1,PSMB2,PSMB3,PSMB4,PSMB5,PSMB6,PSMB7,PSMB8,PSMB9,PSMB10,PSMC1,PSMC2,PSMC3,PSMC4,PSMC5,PSMC6,PSMD1,PSMD2,PSMD3,PSMD4,PSMD5,NUP107,PSMD7,RGL3,PSMD8,PSMD9,PSMD10,PELI2,PELI1,PSMD11,PSMD12,PSMD13,PSME1,PSME2,PTAFR,PTGS2,PTK2,PTPN1,PTPN1,PTPN2,SPTBN4,PTPN3,PTPN4,TRIB3,PTPN6,PTPN6,PTPN7,PTPN9,PTPN11,PTPN11,PTPN12,PTPN13,PTPN14,PTPRA,PTPRZ1,RHOU,ABHD17C,RGL2,RAF1,RAG1,RAG2,RALA,IL22RA1,RALGDS,RANBP2,RAP1A,IL21,RAP1B,RASA1,RASA2,RASGRF1,RASGRF2,SIGIRR,CCND1,BCL2,RELA,RELB,RET,BCL2L1,ACTB,BCL6,EDA2R,RNASEL,ABCE1,TNFRSF17,RORA,RORC,RPLP0,RPS6KA1,RPS6KA2,RPS6KA3,RPS27A,S100A12,S100B,SAA1,MAPK12,CCL2,CCL3,CCL3L1,CCL4,CCL5,CCL11,CCL19,CCL20,CCL22,SDC1,SEC13,GFRA4,CRLF2,CRLF2,NOD2,MAP2K4,MAP2K4,SHC1,SHC1,IL25,RASAL3,SKP1,H3C13,H3C13,BOLA2B,SMARCA4,SNAP25,FSCN1,SNRPA1,SOD1,SOD2,SOS1,SOS2,SOX2,SP100,SPTA1,SPTAN1,SPTB,SPTBN1,SPTBN2,SRC,BRAF,TRIM21,STAT1,STAT1,STAT2,STAT2,STAT3,STAT3,STAT4,STAT4,STAT5A,STAT5A,STAT5B,STAT5B,STAT6,STX1A,STX3,STX4,STXBP2,BST2,VAMP2,VAMP7,BTC,SYK,SYK,ADAM17,MAP3K7,TALDO1,ZEB1,TCP1,TEC,TEK,TGFA,TGFB1,TIMP1,TLN1,ACTG1,TNF,TNFRSF1A,TNFRSF1B,TP53,TPR,HSP90B1,TRAF2,TRAF3,TRAF6,TWIST1,TNFSF4,CCR2,TNFRSF4,TYK2,TYK2,UBA52,UBB,UBC,UBA7,UBE2E1,UBE2N,UBE2V1,SUMO1,VAV1,VCAM1,VCL,VEGFA,VIM,VWF,YES1,YES1,YWHAB,YWHAZ,CA1,TRIM25,TRIM26,IL1R2,MAPKAPK3,NUP37,TRIM48,TNIP2,SEM1,NUP85,NANOG,CALM1,TRIM46,NUP214,TRIM45,SHOC2,CALM2,FGF23,CALM3,DUSP16,AAAS,CAMK2A,CAMK2B,TRIM8,CAMK2D,CAMK2G,ABHD17A,SEH1L,CANX,CAPZA1,BRAP,CASP1,H3C1,H3C1,H3C4,H3C4,H3C3,H3C3,H3C6,H3C6,H3C11,H3C11,H3C8,H3C8,H3C12,H3C12,H3C10,H3C10,H3C2,H3C2,CASP3,KBTBD7,WDR83,RASAL1,CUL3,CUL1,IL1F10,RAE1,IL17RC,PTPN5,HAVCR2,PIK3R3,PIK3R3,IKBKG,IFITM1,TRIM5,TSLP,TSLP,PIAS1,TNFSF11,OASL,LAMTOR3,SOCS1,SOCS1,IRS2,IRS2,CBL,CBL,EIF4G3,PEA15,TNFRSF25,TNFSF14,TNFSF14,TNFSF13,TNFSF12,TNFSF9,TNFRSF14,RIPK2,TNFRSF6B,TNFRSF18,TNFRSF11A,ACTN2,IL18RAP,IL1RL2,IL18R1,FGF18,FGF17,FGF16,IQGAP1,SYNGAP1,SOCS2,SOCS2,KSR1,SQSTM1,BTRC,H3C7,H3C7,TNFSF18,MAP3K14,SOCS3,SOCS3,UBA3,UBE2M,ARTN,AIP,IL33,RSAD2,IL1RL1,OSMR,CD4,IL32,CRLF1,UBE2L6,RPS6KA5,MAPKAPK2,KL,CD27,CD80,CD86,TNFRSF8,TNFSF8,GSTO1,RASAL2,IL27RA,IL27RA,EIF4E2,CD36,PSMF1,NRG2,CD40,CD40LG,CD44,NUP155,ISG15,SOCS5,NUP93,RAPGEF2,CD70,EIF4A3,NUP58,TRIM14,GAB2,GAB2,PSMD6,POM121,FGF19,TNFSF15,NUP153,RBX1,CDC42,RCE1,HNRNPDL",Cytokine Signaling in Immune system,988

R-HSA-1280218,"AKT3,CD300LD,KIR2DS2,GPR75-ASB3,MICA,KLRC4-KLRK1,AP1M2,HUWE1,KIF20A,ACTR1B,ACTR1A,RASGRP1,RASGRP1,BCAP31,RNF41,PSME3,PSME3,PSMD14,PSMD14,KLRG1,CD96,RASGRP2,LOC102725035,STUB1,TRAIP,KLHL41,TLR6,TUBA1B,TUBB3,TUBB4A,TUBB4B,BTN3A3,BTN2A2,ANAPC10,RAPGEF3,UBAC1,ARIH2,SEC24B,IFI30,UBE2E3,SEC23A,LRRC41,FBXW10,ATG7,DCTN2,RBCK1,CENPE,CD226,DCTN6,CTSC,AHCYL1,LOC107987462,SEC24A,LILRB1,HCST,CD300C,MALT1,MALT1,SEC61B,LILRB5,KIF2C,LILRB4,LILRA1,LILRA3,LILRA2,WWP1,UBE2C,RAPGEF4,BTN3A2,BTN3A1,BTN2A1,CD160,KIF3A,GLMN,DCTN3,TUBA3E,KLHL2,CD300A,TUBA3D,TRIM9,SIGLEC11,TIRAP,CHUK,CHUK,SLAMF6,OSBPL1A,FBXO32,FBXO17,THEM4,SH2D1B,AP2M1,AP1S1,AP2S1,UBE2J2,PIK3AP1,PIK3AP1,JAML,CLTA,CLTC,PSMB11,PSMB11,LRR1,CD300LB,OSCAR,FBXO27,ASB17,RNF19B,COL1A1,COL1A2,COL2A1,COL3A1,AP1S3,COL17A1,TRIM71,CD200R1,MAP3K8,RAET1E,TRIM50,ASB10,ASB11,ASB5,ASB6,ASB7,ASB8,ASB9,TRIM69,DYNLL2,UBE2F,MIB2,ASB15,ASB14,ASB12,HECTD2,PSMA8,PSMA8,CSK,FBXL14,FBXL16,CD300LF,CD300LG,KLC3,UBE2U,CTLA4,FBXO41,CTSB,CTSD,CTSE,CTSH,CTSK,CTSL,CTSL,CTSV,CTSV,DTX3L,BTLA,BTLA,CTSO,CTSS,CTSS,CXADR,CYBA,BTNL9,CYBB,RNF217,KCTD7,CBLL2,AP2A1,AP2A2,AP1B1,AP2B1,DCTN1,AP1G1,DNM1,DYNC1H1,DYNC1I1,DYNC1I2,DYNC1LI2,DNM2,PIANP,UBR1,KCTD6,FBXO15,AKT1,AKT2,FCGR1A,FCGR1A,FCGR2B,FCGR3A,RNF182,FBXL13,ZNRF2,FKBP1A,SEC31A,UBOX5,KLRK1,KIFAP3,TRIM32,FBXO21,RAP1GAP2,TAB2,FBXL7,PSME4,PSME4,FBXW11,MGRN1,UBR2,ICOSLG,NEDD4L,UBR4,UFL1,SEC61G,LILRA4,MKRN1,LY96,KIF4A,CDC26,MTOR,RICTOR,FYB1,FYB1,FYN,FYN,RNF144B,RASGRP3,RASGRP3,FBXO7,HECTD1,ANAPC13,RNF19A,RCHY1,NCR3,LTN1,DNM3,HERC4,WSB1,KIF26A,FBXW2,PTPN22,FBXL3,FBXO2,FBXL5,FBXL4,KLHL3,FBXW8,FBXO22,FBXO10,FBXO9,FBXO6,FBXO4,SIGLEC7,LAT,DAPP1,SIGLEC9,SIGLEC8,RNF115,KLHL20,UBE2S,FBXL22,FBXW12,KIF4B,TREML4,GRB2,GRB2,MYLIP,CLEC2D,CD274,RACGAP1,PDIA3,PDIA3,BLNK,BLNK,ICOS,ANAPC2,SEC61A1,ANAPC4,PILRB,PILRA,SH3KBP1,SH3KBP1,CD209,UBE2K,HLA-A,HLA-A,HLA-B,HLA-B,HLA-C,HLA-C,HLA-DMB,HLA-DOA,HLA-DOB,HLA-DPA1,HLA-DPB1,HLA-DQA1,HLA-DQA2,HLA-DQB1,HLA-DQB2,HLA-DRA,HLA-DRB1,HLA-DRB3,HLA-DRB4,HLA-DRB5,HLA-E,HLA-E,HLA-F,HLA-F,HLA-G,HLA-G,HRAS,HSPA5,HSPA5,ICAM1,ICAM2,ICAM3,ICAM4,CLEC4G,TREML1,CD300E,TUBB8,TUBB2B,LILRA5,IKBKB,IKBKB,INPP5D,ITGA4,ITGAL,ITGAV,ITGB1,ITGB2,ITGB5,ITGB7,ITK,ITPR1,ITPR1,ITPR2,ITPR2,ITPR3,ITPR3,NCR3LG1,ARF1,KIF2A,KIF3C,KIF5A,KIF5B,KIR2DL1,KIR2DL2,KIR2DL3,KIR2DL4,KIR2DS1,KIR3DL1,KIR3DL2,KLRB1,KLRC1,KLRD1,KLC1,KIF11,KIF22,KRAS,TMEM189-UBE2V1,LAG3,LAIR1,LAIR2,KBTBD13,LCK,LCP2,LCP2,LMO7,ASB18,LNPEP,LYN,LYN,SH2D1A,CD99,MICB,CD200,MRC1,MRC1,TRIM37,MYD88,NCF2,NCF4,NCK1,NCK1,NEDD4,NFATC1,NFATC1,NFATC2,NFATC2,NFATC3,NFATC3,NFKB1,NFKBIA,NFKBIB,NFKBIE,NRAS,CD207,CD207,PAK1,PAK2,PAK3,PRKN,TRAT1,KLHL5,SAR1B,ASB3,RLIM,DYNC1LI1,DCTN4,HERC5,MEX3C,PDCD1,FZR1,KLRF1,ANAPC5,ANAPC7,RNF138,UBE2J1,EVL,ANAPC11,UBE2D4,ASB1,ASB4,ASB2,PDPK1,FBXO40,ERAP1,TUBA8,PIK3CA,PIK3CB,PIK3CD,PIK3CD,PIK3R1,PIK3R1,PIK3R2,BTBD1,PLCG1,PLCG2,PLCG2,TREM2,TREM1,FBXW5,FBXL19,CTSA,RNF111,PPIA,FBXL12,UBE2R2,PPL,HERC6,DET1,PPP2CA,PPP2CB,KLHL11,SEC61A2,PPP2R1A,RNF220,PPP2R1B,UBA6,PPP2R5A,PPP2R5B,PPP2R5C,PPP2R5D,UBE2W,PPP2R5E,FBXW7,PPP3CA,PPP3CA,PPP3CB,PPP3CB,FBXL8,PPP3R1,PPP3R1,TRIM36,UBE2Q1,PRR5,RNF126,PRKACA,PRKACB,PRKACG,ENAH,PRKCB,RNF130,PAG1,ACTR10,PRKCQ,RNF114,PRKG1,KLHL9,BTNL2,CRTAM,LGMN,NPDC1,TRIM39,B2M,B2M,PSMA1,PSMA1,PSMA2,PSMA2,PSMA3,PSMA3,PSMA4,PSMA4,PSMA5,PSMA5,PSMA6,PSMA6,PSMA7,PSMA7,PSMB1,PSMB1,PSMB2,PSMB2,PSMB3,PSMB3,PSMB4,PSMB4,PSMB5,PSMB5,PSMB6,PSMB6,PSMB7,PSMB7,PSMB8,PSMB8,PSMB9,PSMB9,PSMB10,PSMB10,KIF15,PSMC1,PSMC1,PSMC2,PSMC2,PSMC3,PSMC3,PSMC4,PSMC4,PSMC5,PSMC5,PSMC6,PSMC6,PSMD1,PSMD1,PSMD2,PSMD2,PSMD3,PSMD3,PSMD4,PSMD4,PSMD5,PSMD5,PSMD7,PSMD7,PSMD8,PSMD8,PSMD9,PSMD9,SMURF1,PSMD10,PSMD10,PSMD11,PSMD11,PSMD12,PSMD12,PSMD13,PSMD13,PSME1,PSME1,PSME2,PSME2,PTEN,HECW2,HACE1,KLHL42,SH3RF1,RNF213,TRIB3,PTPN6,PTPN6,PTPN11,SLAMF7,PTPRC,PTPRJ,PVR,NECTIN2,RAC1,RAF1,RAP1A,RAP1B,RAP1GAP,RBBP6,REL,RELA,RNF4,RNF6,RPS27A,RPS27A,RNF123,UBE2O,SEC13,BLK,SELL,ERAP2,BLMH,PJA1,MLST8,RNF25,SFTPD,KLHL25,SH3GL2,FBXW4,ANAPC1,UNKL,SMURF2,SIAH1,SIAH2,KLC2,SIPA1,SKP1,SKP2,UBE2Z,NCF1,SIGLEC1,SOS1,SOS1,SPTBN2,SRC,TRIM21,STIM1,STIM1,STX4,SYK,SYK,MAP3K7,MAP3K7,TAP1,TAP1,TAP2,TAP2,TAPBP,ELOC,ELOB,BTK,BTK,BTN1A1,THOP1,ICAM5,TLR1,TLR2,TLR4,TPP2,C3,TRAF6,TRPC1,TUBA4A,TUBA3C,TUBB2A,TYROBP,UBA52,UBA52,UBB,UBB,UBC,UBC,UBA1,UBA7,UBE2A,UBE2B,UBE2D1,UBE2D2,UBE2D3,UBE2E1,UBE2E2,UBE2G1,UBE2G2,UBE2H,UBE2L3,UBE2N,UBE2V1,UBE2V2,UBE3A,VASP,VAV1,VAV1,VCAM1,VHL,WAS,XDH,YES1,YWHAB,YWHAZ,ZAP70,ZBTB16,TUBA1A,RAB7A,MAPKAP1,FBXL15,ULBP3,HECTD3,ASB13,SEM1,SEM1,FBXO31,TUBAL3,TREML2,UBA5,BTNL8,CALM1,CALM1,SPSB1,RNF34,FBXO11,ORAI2,ULBP1,PDCD1LG2,CALM2,CALM2,CUL5,CALM3,CALM3,TUBB1,COLEC12,CALR,GAN,TRIM11,MADCAM1,KIF18A,CANX,CANX,CAPZA1,CAPZA2,CAPZB,RILP,ITCH,KBTBD7,FBXO30,TRAF7,FBXW9,CARD11,CARD11,DCTN5,CUL3,CUL2,CUL1,KBTBD8,TUBB6,KIF2B,TRIM63,LNX1,SPSB2,TUBA1C,KLHL22,ORAI1,ZNRF1,FBXL20,PIK3R3,IKBKG,IKBKG,IFITM1,SOCS1,DYNLL1,VAMP8,CBLB,CDC23,CTSF,TNFRSF14,TNFRSF14,RIPK2,SNAP23,SIGLEC5,CDC16,AP1S2,AP1M1,TRIM4,BCL10,BCL10,HERC3,HERC2,HERC1,BTRC,SIGLEC10,SIGLEC12,KBTBD6,CCNF,UBE3B,KLC4,UBE3D,BTBD6,MAP3K14,SOCS3,KLHL13,UBA3,UBE2M,LRSAM1,CD1A,TRIM41,CD1B,CD1C,CD1D,CD3D,CD3E,LONRF1,CD3G,CD247,CD4,SPSB4,UBE2L6,CD8A,ASB16,CD8B,CD14,UBE2Q2,CD19,CD19,TRIP12,CD22,CD22,VAMP3,UBE4A,FBXO44,CAPZA3,KIF3B,CD101,CD28,GRAP2,CD80,CD86,NCR2,NCR1,CD33,SIGLEC6,CD34,CD36,PSMF1,PSMF1,KIF23,NPEPPS,SEC22B,CD40,CD40LG,RNF14,RNF7,SEC24C,DZIP3,UBE3C,CD74,CD79A,CD79A,DCAF1,CD79B,CD79B,CD81,KEAP1,CUL7,PSMD6,PSMD6,PJA2,AREL1,SEC24D,MRC2,MRC2,KLHL21,CDC20,CDC27,CDC34,CLEC2B,RBX1,CDC42,CDH1",Adaptive Immune System,869

R-HSA-1295596,"SPRY2,GRB2,PPP2CA,PPP2CB,PPP2R1A,MAPK1,MAPK3,PTPN11,RPS27A,SRC,BRAF,UBA52,UBB,UBC,MKNK1,CBL",Spry regulation of FGF signaling,16

R-HSA-1296025,"ABCC9,KCNJ8,KCNJ11,ABCC8",ATP sensitive Potassium channels,4

R-HSA-1296041,"GNB5,GABBR1,GNB1,GNB2,GNB3,GNG3,GNG4,GNG5,GNG7,GNG10,GNG11,GNGT1,GNGT2,KCNJ2,KCNJ3,KCNJ4,KCNJ5,KCNJ6,KCNJ9,KCNJ10,KCNJ12,KCNJ15,KCNJ16,GNG13,GNG2,GNG12,GNB4,GNG8,GABBR2",Activation of G protein gated Potassium channels,29

R-HSA-1296052,"KCNMB2,KCNMB3,KCNMB4,KCNMA1,KCNMB1,KCNN1,KCNN2,KCNN3,KCNN4",Ca2+ activated K+ channels,9

R-HSA-1296053,"KCNJ2,KCNJ4,KCNJ12,KCNJ14",Classical Kir channels,4

R-HSA-1296059,"GNB5,GABBR1,GNB1,GNB2,GNB3,GNG3,GNG4,GNG5,GNG7,GNG10,GNG11,GNGT1,GNGT2,KCNJ2,KCNJ3,KCNJ4,KCNJ5,KCNJ6,KCNJ9,KCNJ10,KCNJ12,KCNJ15,KCNJ16,GNG13,GNG2,GNG12,GNB4,GNG8,GABBR2",G protein gated Potassium channels,29

R-HSA-1296061,"HCN4,HCN1,HCN3,HCN2",HCN channels,4

R-HSA-1296065,"ABCC9,GNB5,GABBR1,GNB1,GNB2,GNB3,GNG3,GNG4,GNG5,GNG7,GNG10,GNG11,GNGT1,GNGT2,KCNJ1,KCNJ2,KCNJ3,KCNJ4,KCNJ5,KCNJ6,KCNJ8,KCNJ9,KCNJ10,KCNJ11,KCNJ12,KCNJ14,KCNJ15,KCNJ16,GNG13,GNG2,GNG12,GNB4,ABCC8,GNG8,GABBR2",Inwardly rectifying K+ channels,35

R-HSA-1296067,"KCNJ1,KCNJ10,KCNJ16",Potassium transport channels,3

R-HSA-1296071,"HCN4,ABCC9,KCNK7,KCNMB2,GNB5,KCNH8,KCNV2,KCNG3,KCNH4,KCNH3,GABBR1,KCNG2,KCNV1,KCNMB3,KCNH5,KCNMB4,GNB1,GNB2,GNB3,GNG3,GNG4,GNG5,GNG7,GNG10,GNG11,GNGT1,GNGT2,KCNK18,HCN1,KCNA1,KCNA2,KCNA3,KCNA4,KCNA5,KCNA6,KCNA7,KCNA10,KCNB1,KCNC1,KCNC2,KCNC3,KCNC4,KCND1,KCND2,KCND3,KCNF1,KCNG1,KCNH1,KCNH2,KCNJ1,KCNJ2,KCNJ3,KCNJ4,KCNJ5,KCNJ6,KCNJ8,KCNJ9,KCNJ10,KCNJ11,KCNJ12,KCNJ14,KCNJ15,KCNJ16,KCNK1,KCNK2,KCNK3,KCNMA1,KCNMB1,KCNN1,KCNN2,KCNN3,KCNN4,KCNQ1,KCNQ2,KCNQ3,KCNS1,KCNS2,KCNS3,KCNK4,KCNK9,GNG13,KCNK10,GNG2,GNG12,KCNQ5,KCNK13,HCN3,GNB4,HCN2,ABCC8,KCNAB1,KCNH6,KCNK16,KCNAB2,KCNK17,KCNH7,KCNQ4,KCNAB3,KCNG4,KCNB2,GNG8,KCNK6,GABBR2",Potassium Channels,103

R-HSA-1296072,"KCNH8,KCNV2,KCNG3,KCNH4,KCNH3,KCNG2,KCNV1,KCNH5,KCNA1,KCNA2,KCNA3,KCNA4,KCNA5,KCNA6,KCNA7,KCNA10,KCNB1,KCNC1,KCNC2,KCNC3,KCNC4,KCND1,KCND2,KCND3,KCNF1,KCNG1,KCNH1,KCNH2,KCNQ1,KCNQ2,KCNQ3,KCNS1,KCNS2,KCNS3,KCNQ5,KCNAB1,KCNH6,KCNAB2,KCNH7,KCNQ4,KCNAB3,KCNG4,KCNB2",Voltage gated Potassium channels,43

R-HSA-1296346,"KCNK7,KCNK18,KCNK1,KCNK2,KCNK3,KCNK4,KCNK9,KCNK10,KCNK13,KCNK16,KCNK17,KCNK6",Tandem pore domain potassium channels,12

R-HSA-1299287,KCNK13,Tandem pore domain halothane-inhibited K+ channel (THIK),1

R-HSA-1299308,"KCNK7,KCNK1,KCNK6",Tandem of pore domain in a weak inwardly rectifying K+ channels (TWIK),3

R-HSA-1299316,"KCNK3,KCNK9",TWIK-releated acid-sensitive K+ channel (TASK),2

R-HSA-1299344,KCNK18,TWIK-related spinal cord K+ channel (TRESK),1

R-HSA-1299361,"KCNK16,KCNK17",TWIK-related alkaline pH activated K+ channel (TALK),2

R-HSA-1299503,"KCNK2,KCNK4,KCNK10",TWIK related potassium channel (TREK),3

R-HSA-1300642,"CATSPER1,CATSPER2,KCNU1,CATSPERD,CATSPER3,CATSPER4,CATSPERG,CATSPERB,HVCN1",Sperm Motility And Taxes,9

R-HSA-1300645,"IZUMO4,IZUMO2,IZUMO1,ACR,CD9",Acrosome Reaction and Sperm:Oocyte Membrane Binding,5

R-HSA-1306955,"ERBB2,ERBB3,GRB7,NRG1,NRG2",GRB7 events in ERBB2 signaling,5

R-HSA-1307965,"KLB,FGFR4,FGF19",betaKlotho-mediated ligand binding,3

R-HSA-1358803,"AKT3,RNF41,ERBB2,ERBB3,AKT1,AKT2,NRG1,RPS27A,UBA52,UBB,UBC,USP8,NRG2",Downregulation of ERBB2:ERBB3 signaling,13

R-HSA-1362277,"LIN54,LIN54,E2F1,E2F1,E2F4,E2F4,E2F5,E2F5,LIN9,LIN9,HDAC1,MAX,MYC,PCNA,LIN37,LIN37,RBBP4,RBBP4,RBL1,RBL1,RBL2,RBL2,TFDP1,TFDP1,TFDP2,TFDP2,TOP2A,LIN52,LIN52,CDC6,CDC25A",Transcription of E2F targets under negative control by DREAM complex,31

R-HSA-1362300,"LIN54,E2F1,E2F4,E2F5,LIN9,HDAC1,MYBL2,LIN37,RBBP4,RBL1,RBL2,TFDP1,TFDP2,CCNA2,LIN52,CDK1",Transcription of E2F targets under negative control by p107 (RBL1) and p130 (RBL2) in complex with HDAC1,16

R-HSA-1362409,"FDX2,ISCA2,HSCB,FDX1,FDXR,ISCU,FXN,GLRX5,SLC25A37,LYRM4,ISCA1,SLC25A28,NFS1",Mitochondrial iron-sulfur cluster biogenesis,13

R-HSA-1368071,"HDAC3,NR1D1,NCOR1",NR1D1 (REV-ERBA) represses gene expression,3

R-HSA-1368082,"CARM1,NCOA2,CPT1A,CREBBP,EP300,EP300,NCOA6,PPARA,MED1,RORA,RORA,RXRA,SMARCD3,SREBF1,TBL1X,TBL1XR1,CHD9,HELZ2,NCOA1,TGS1",RORA activates gene expression,20

R-HSA-1368108,"NAMPT,CARM1,NCOA2,CREBBP,DBP,F7,NCOA6,NOCT,KLF15,ARNTL,ARNTL,NPAS2,NPAS2,SERPINE1,PPARA,MED1,AVP,ARNTL2,RXRA,SMARCD3,TBL1X,BHLHE41,TBL1XR1,CHD9,HELZ2,BHLHE40,NCOA1,CLOCK,CLOCK,TGS1","BMAL1:CLOCK,NPAS2 activates circadian gene expression",30

R-HSA-1369007,"ABCB6,ABCB8,ABCB7,ABCB10",Mitochondrial ABC transporters,4

R-HSA-1369062,"ABCA7,ABCA10,ABCA9,ABCA2,ABCA3,ABCD1,ABCD2,ABCA6,ABCA5,ABCA12,APOA1,PEX19,ABCD3,ABCG4,ABCG5,ABCG8,PEX3,ABCG1",ABC transporters in lipid homeostasis,18

R-HSA-139853,"P2RX2,ITPR1,ITPR2,ITPR3,P2RX1,P2RX3,P2RX4,P2RX5,P2RX7,TRPC7,STIM1,TRPC3,TRPC6,ORAI2,ORAI1,P2RX6",Elevation of cytosolic Ca2+ levels,16

R-HSA-139910,"DYNLL2,MAPK8,BMF",Activation of BMF and translocation to mitochondria,3

R-HSA-139915,"E2F1,PPP1R13B,BBC3,TFDP1,TFDP2,TP53,TP53BP2,TP73,TP63",Activation of PUMA and translocation to mitochondria,9

R-HSA-140179,"PAOX,MAOA,MAOB,SMOX",Amine Oxidase reactions,4

R-HSA-140180,PTGS1,COX reactions,1

R-HSA-140342,"DFFA,DFFB,H1-0,H1-2,H1-3,H1-4,H1-5,H1-1,HMGB1,HMGB2,KPNA1,KPNB1,CASP3",Apoptosis induced DNA fragmentation,13

R-HSA-140534,"TMED7-TICAM2,TICAM1,LY96,TICAM2,FAS,FASLG,TLR4,TRAF2,CASP8,TRADD,RIPK1,TNFSF10,FADD,TNFRSF10B,TNFRSF10A,CFLAR,CD14",Caspase activation via Death Receptors in the presence of ligand,17

R-HSA-140834,"F3,F7,F9,F10,TFPI",Extrinsic Pathway of Fibrin Clot Formation,5

R-HSA-140837,"A2M,F2,F8,F9,F10,F11,F12,GP1BA,GP1BB,GP5,GP9,SERPIND1,KLKB1,KNG1,SERPINC1,SERPINA5,SERPINE2,PRCP,PROC,PROS1,C1QBP,SERPING1,VWF",Intrinsic Pathway of Fibrin Clot Formation,23

R-HSA-140875,"PROCR,F2,F2R,F5,F8,F10,F13A1,F13B,FGA,FGB,FGG,SERPIND1,SERPINC1,SERPINA5,PF4,PF4V1,SERPINE2,PROC,PROS1,PRTN3,CD177,THBD",Common Pathway of Fibrin Clot Formation,22

R-HSA-140877,"PROCR,A2M,F2,F2R,F3,F5,F7,F8,F9,F10,F11,F12,F13A1,F13B,FGA,FGB,FGG,GP1BA,GP1BB,GP5,GP9,SERPIND1,KLKB1,KNG1,SERPINC1,SERPINA5,PF4,PF4V1,SERPINE2,PRCP,PROC,PROS1,PRTN3,CD177,TFPI,THBD,C1QBP,SERPING1,VWF",Formation of Fibrin Clot (Clotting Cascade),39

R-HSA-141333,"MAOA,MAOB",Biogenic amines are oxidatively deaminated to aldehydes by MAOA and MAOB,2

R-HSA-141334,"PAOX,SMOX",PAOs oxidise polyamines to amines,2

R-HSA-141405,"ANAPC10,UBE2C,ANAPC16,CDC26,ANAPC15,UBE2S,ANAPC2,ANAPC4,MAD2L1,ANAPC5,ANAPC7,ANAPC11,ANAPC1,BUB1B,UBE2D1,UBE2E1,CDC23,CDC16,BUB3,CDC20,CDC27",Inhibition of the proteolytic activity of APC/C required for the onset of anaphase by mitotic spindle checkpoint components,21

R-HSA-141424,"CENPS-CORT,PMF1-BGLAP,NDC80,CENPA,CENPC,CENPE,CENPF,NUDC,KIF2C,ZWINT,PMF1,DYNLL2,SPC24,SGO2,SGO1,DYNC1H1,DYNC1I1,DYNC1I2,DYNC1LI2,SKA1,MAPRE1,CLASP2,NUP160,CLASP1,ITGB3BP,CENPI,AHCTF1,NSL1,BIRC5,SKA2,NUP43,INCENP,CENPS,KIF2A,CENPP,MAD2L1,NUP98,PAFAH1B1,DYNC1LI1,PLK1,NDE1,ERCC6L,SPDL1,PPP1CC,ZWILCH,CDCA8,PPP2CA,PPP2CB,CENPQ,PPP2R1A,PPP2R1B,PPP2R5A,PPP2R5B,PPP2R5C,PPP2R5D,PPP2R5E,NUP133,CENPN,RCC2,KNL1,NUP107,SPC25,TAOK1,RANBP2,RANGAP1,RPS27,CLIP1,SEC13,CENPK,CENPH,BUB1,BUB1B,XPO1,MIS12,CENPM,NUP37,CENPO,CENPU,NUP85,DSN1,CENPT,B9D2,NDEL1,SEH1L,KIF18A,NUF2,MAD1L1,KIF2B,DYNLL1,CENPL,ZW10,BUB3,AURKB,KNTC1,CKAP5,CDC20",Amplification of signal from the kinetochores,96

R-HSA-141430,"ANAPC10,UBE2C,ANAPC16,CDC26,ANAPC15,UBE2S,ANAPC2,ANAPC4,MAD2L1,ANAPC5,ANAPC7,ANAPC11,ANAPC1,BUB1B,UBE2D1,UBE2E1,CDC23,CDC16,BUB3,CDC20,CDC27",Inactivation of APC/C via direct inhibition of the APC/C complex,21

R-HSA-141444,"CENPS-CORT,PMF1-BGLAP,NDC80,CENPA,CENPC,CENPE,CENPF,NUDC,KIF2C,ZWINT,PMF1,DYNLL2,SPC24,SGO2,SGO1,DYNC1H1,DYNC1I1,DYNC1I2,DYNC1LI2,SKA1,MAPRE1,CLASP2,NUP160,CLASP1,ITGB3BP,CENPI,AHCTF1,NSL1,BIRC5,SKA2,NUP43,INCENP,CENPS,KIF2A,CENPP,MAD2L1,NUP98,PAFAH1B1,DYNC1LI1,PLK1,NDE1,ERCC6L,SPDL1,PPP1CC,ZWILCH,CDCA8,PPP2CA,PPP2CB,CENPQ,PPP2R1A,PPP2R1B,PPP2R5A,PPP2R5B,PPP2R5C,PPP2R5D,PPP2R5E,NUP133,CENPN,RCC2,KNL1,NUP107,SPC25,TAOK1,RANBP2,RANGAP1,RPS27,CLIP1,SEC13,CENPK,CENPH,BUB1,BUB1B,XPO1,MIS12,CENPM,NUP37,CENPO,CENPU,NUP85,DSN1,CENPT,B9D2,NDEL1,SEH1L,KIF18A,NUF2,MAD1L1,KIF2B,DYNLL1,CENPL,ZW10,BUB3,AURKB,KNTC1,CKAP5,CDC20",Amplification of signal from unattached kinetochores via a MAD2 inhibitory signal,96

R-HSA-1428517,"LRPPRC,TRAP1,ATP5PD,ATP5MG,ME3,UQCR11,COX20,NDUFA11,COX4I1,COX5B,COX6A1,COX6B1,COX6C,COX7B,COX7C,COX8A,COX11,NDUFAF6,ADHFE1,CS,PM20D1,CYC1,LDHAL6A,DLAT,DLD,DLST,ETFA,ETFB,ETFDH,FH,NNT,SLC16A8,MPC2,NDUFAF3,UQCRQ,DMAC2L,GLO1,COX18,ACAD9,NDUFAF4,GSTZ1,UQCR10,HAGH,IDH2,IDH3A,IDH3B,IDH3G,NDUFS7,LDHA,LDHB,LDHC,MDH2,ME1,ME2,ATP6,ATP8,COX1,COX2,COX3,CYTB,ND1,ND2,ND3,ND4,ND5,ND6,NDUFA1,NDUFA2,NDUFA3,NDUFA4,NDUFA5,NDUFA6,NDUFA7,NDUFA8,NDUFA9,NDUFA10,NDUFAB1,NDUFB1,NDUFB2,NDUFB3,NDUFB4,NDUFB5,NDUFB6,NDUFB7,NDUFB8,NDUFB9,NDUFB10,NDUFC1,NDUFC2,NDUFS1,NDUFS2,NDUFS3,NDUFV1,NDUFS4,NDUFS5,NDUFS6,NDUFS8,NDUFV2,NDUFV3,OGDH,ATP5F1A,ACO2,ATP5F1B,ATP5F1C,NDUFA13,NDUFAF1,TACO1,COX16,ECSIT,ATP5F1D,TIMMDC1,ATP5F1E,ATP5PB,ATP5MC1,PDHA1,PDHA2,PDHB,PDK1,PDK2,PDK3,PDK4,MPC1,ATP5MC2,ATP5MC3,ATP5ME,ATP5PF,ATP5PO,CYCS,NDUFB11,PPARD,PDP1,PDPR,NDUFAF7,TMEM126B,NDUFA12,PDP2,RXRA,SCO1,SDHA,SDHA,SDHB,SDHB,SDHC,SDHC,SDHD,SDHD,SLC16A1,BSG,SURF1,D2HGDH,UCP1,UCP2,UCP3,UQCRB,UQCRC1,UQCRC2,UQCRFS1,UQCRH,VDAC1,NDUFAF5,L2HGDH,COQ10B,NUBPL,PDHX,FAHD1,COX14,SUCLG2,SUCLG1,SUCLA2,SLC25A14,SLC16A3,COX7A2L,NDUFAF2,LDHAL6B,COQ10A,COX5A,SLC25A27,ATP5MF,SCO2",The citric acid (TCA) cycle and respiratory electron transport,179

R-HSA-1430728,"NAT2,ADA,MED6,NAALAD2,ACOT8,GNPDA1,TSTD1,PLA2G4B,MED16,NT5C1B-RDH14,ABCC5,NR1H3,GPC6,GPC6,CYP3A7-CYP3A51P,CERT1,UST,NUBP2,LRPPRC,TRAP1,NAMPT,AASS,LPCAT3,SLC25A13,SLC25A15,PSME3,PSMD14,AK6,PLIN3,COQ7,CDK8,GPHN,RIDA,GLYAT,CBSL,CBSL,BCKDK,B3GALT5,AKR1A1,B3GNT3,CDO1,BPNT1,CEPT1,CDS1,PEMT,ST3GAL6,RAPGEF3,CDIPT,SEC24B,LYPLA1,ACAA2,ECI2,ATP5PD,SLC25A17,SEC23A,CARM1,CARM1,NCOA2,NCOA2,AGPAT1,AGPAT2,SPTLC1,SLC19A2,MTHFS,DNPH1,SLCO1B1,PAICS,ATP5MG,PMVK,CES1,CSPG5,B3GNT2,GNB5,GNB5,EBP,FUT9,ADCY1,CERS1,UGT2B11,PTGES3,NUP50,AHCYL1,MTHFD2,ADCY2,SEC24A,STARD10,CGA,NEU3,ALDH1L1,FTCD,HPSE,CYP46A1,SLC26A1,ME3,PPARGC1A,LYVE1,ADCY3,PNPLA6,UGT2A1,STARD3,SERINC3,ACOT2,HTD2,UQCR11,SDS,SLC27A5,SLC27A3,SLC27A2,GLIPR1,LIAS,CHAT,B4GAT1,SLC35D2,RAPGEF4,KERA,AKR1C4,NUP42,ADCY5,HIBADH,PLAAT3,NUDT4,NUDT5,NUDT3,INMT,CHKA,ADCY6,CHKB,RPL35,CA5B,CHP1,FDX2,HOGA1,CYP4F8,B4GALT7,MED8,ADCY7,PLCD3,SLCO2B1,CHRM3,CHST14,SLC46A1,SLC52A3,CTRC,ACOT7,MGLL,MGLL,AZIN2,CYP2U1,SDSL,ADCY8,CYGB,OSBPL1A,OSBPL5,OSBPL6,OSBPL7,OSBPL8,OSBPL9,OSBPL10,CIDEA,PTPMT1,ADCY9,CKB,NOSTRIN,CKM,CKMT1B,CKMT2,COX20,MOGAT1,ACSM1,APOA5,RPL39L,THEM4,PLAAT5,IP6K3,PSTK,GSTO2,SLC36A4,CYP2R1,CLPS,TPH2,AK7,PLD4,PLD4,ADSS1,PSMB11,ISCA2,ACOT4,PLIN2,DEGS2,HYKK,PLA2G4E,ACSM2A,ADH1A,ABCC2,B4GALNT2,GGT6,ADH1B,AFMID,ACER1,ADH1C,TNFAIP8L1,NDUFA11,CYP4F22,B3GALT6,SLC44A3,ADH4,PRXL2B,ADH5,NAXE,FITM2,NUP35,MBOAT2,NEU4,AADAC,ADH6,ACMSD,SGPP2,ADH7,COMT,UROC1,NUDT16,ADK,GLYCTK,COX4I1,GNPDA2,COX5B,ENPP6,PPARGC1B,NADK2,UGT3A1,COX6A1,COX6B1,CMBL,STARD4,COX6C,ACOT12,COX7B,COX7C,COX8A,B3GAT2,COX10,COX11,COX15,CPOX,CPS1,CPT1A,CPT1B,CPT2,NDUFAF6,ADHFE1,GPAT4,HGSNAT,CARNMT1,CRAT,CREBBP,CREBBP,RPS4Y2,RPL10L,CRYM,ACSM6,SAMD8,PARP4,CS,PSMA8,AMDHD1,PTGR2,CSNK1G2,CSNK2A1,CSNK2A2,CSNK2B,VCAN,NCAN,CSPG4,SLC25A10,PIK3R6,STARD6,PM20D1,CTH,CTH,LIPI,AANAT,ADRA2A,HSCB,CTPS1,PNPLA5,CTRB1,GPAT2,PLB1,UPP2,PPM1L,ADRA2C,CYB5A,PPM1K,ARSK,CYC1,MBOAT1,CYP1A1,CYP1A2,CYP1B1,CYP2A6,VKORC1L1,CYP2A7,CYP3A7,CYP2A13,CYP2B6,CYP2C19,CYP2C8,CYP2C9,CYP2C18,CYP2D6,CYP2E1,CYP2F1,CYP2J2,CYP3A4,CYP3A5,CYP4A11,ADSL,CYP4B1,AK8,CYP7A1,CYP8B1,CYP11A1,CYP11B1,CYP11B2,FAAH2,CYP17A1,CYP19A1,AWAT1,AWAT2,CYP21A2,ADSS2,CYP24A1,CYP26A1,CYP27A1,CYP27B1,CYP51A1,LDHAL6A,ALDH1L2,SLC5A8,DAO,FITM1,DARS1,ADAL,DBH,DBI,NAGS,PHOSPHO1,DBT,ECI1,DCK,DCN,DCTD,DCT,DDC,AKR1C1,AKR1C2,SPTSSB,CHST13,DECR1,MMAA,SGMS2,UGT3A2,IDO2,FUT11,NUDT10,SPTSSA,ABHD3,DGUOK,DHCR7,DHCR24,DHFR,DHFR,DHODH,CYB5R3,NQO1,DIO1,DIO2,DIO3,DLAT,DLD,DLST,SARDH,ACAN,AGL,DNM2,DPEP1,DPYD,DPYS,AGT,SLC26A2,DTYMK,DUT,AGXT,TYMP,ECHS1,ABCA1,AHCY,AHCY,AHR,EHHADH,PAOX,ADCY4,ACSF3,PIKFYVE,PIKFYVE,LIPH,DHFR2,RPL22L1,PLD6,PLD6,HACD2,ENO1,ENO2,ENO3,AK1,EP300,AK2,CERS3,SLC44A5,AK4,EPHX1,EPHX2,EPRS1,ERCC2,AKT1,ESD,ALAD,ESRRA,ETFA,ETFB,ALAS1,ETFDH,ALAS2,ALB,EXT1,EXT2,ABCD1,ALDH1A1,FAAH,FABP4,FABP4,FABP1,FABP2,ALDH2,FABP3,FABP5,FABP6,FABP7,ALDH3A1,ACSL1,ACSL3,ACSL4,FAH,ALDH1B1,FASN,FASN,MED19,FOLH1B,SLC37A2,GLYATL2,ABCB7,FBP1,ALDH3B1,AK9,GSTA5,PRPS1L1,GPC2,GPC2,FDFT1,FDPS,HS3ST5,ALDH9A1,FDX1,FDXR,FECH,GPC4,GPC4,ALDH3A2,ALDOA,GPC5,GPC5,FH,FHL2,CHSY1,PLEKHA6,INPP5F,MORC2,ALDOB,ARSG,SACM1L,PLA2R1,SEPHS2,RPIA,SCAP,SCAP,PTGR1,AKR7A3,NT5C2,ALDOC,PLCH1,NCOA6,NCOA6,NMNAT2,CDK19,AKR1B1,NUP205,SLC35D1,GPD1L,LPIN1,RGL1,PSME4,ACSBG1,NUP210,PLCB1,PLCB1,DDHD2,FMO1,PPIP5K2,FMO2,NUP160,FMO3,ACSL6,SIN3B,FMOD,MAN2B2,ESYT1,MED13L,PIP5K1C,MLYCD,SLC44A1,FOLH1,GCAT,ETHE1,QPRT,ISCU,CES3,HAAO,FOLR2,NUP188,RPL13A,NNT,PIK3R5,SLC16A8,FPGS,CHST5,DDAH2,DDAH1,PDSS1,ACOT9,AMACR,CA14,NUP62,PLD3,PLA2G15,PRKD3,SHPK,BHMT2,PITPNB,PISD,ALOX12,FXN,ALOX5,ALOX5AP,ALOX12B,ALOX15,AKR7L,ALOX15B,ALPI,FUT1,FUT2,FUT3,FUT4,FUT5,FUT6,FUT7,TECRL,KDSR,IPMK,LCLAT1,CERS6,G6PC,G6PD,SLC37A4,LPCAT4,GAA,TMEM86B,PLA2G4F,SERINC5,PGLS,GALC,TNFAIP8,GALE,TXN2,B4GALNT1,SULT4A1,GALK1,PRKD2,SUMF2,RPL36,MPC2,GALNS,MTHFD1L,NDUFAF3,GALT,SGMS1,GAMT,IL4I1,SAMHD1,SIN3A,NUDT13,GAPDH,MMACHC,DHRS7B,AOC1,TKFC,LRP10,ACOT11,OSBPL3,GLCE,HACL1,DECR2,GART,AMD1,PHGDH,B3GAT3,TIAM2,KCNG2,HIBCH,PLA2G2D,GATM,AK5,GBA,GBE1,GAPDHS,GC,GCDH,GCG,GCG,GCH1,GCHFR,GCK,GCKR,GCSH,HS6ST3,GGT1,B4GALT1,GGT7,GGT5,OPLAH,CBLIF,AMPD1,TPK1,ACAD8,ANKRD1,PPA2,B3GAT1,UQCRQ,AMPD2,GK,DMAC2L,GK2,INPP5J,CPNE7,NDOR1,GLS2,GLA,GPC3,GPC3,AMPD3,GLB1,NMRK2,GNMT,GNMT,SULT1C4,TNFRSF21,MMADHC,SULT1B1,GCLC,GCLM,MOCS3,HPGDS,GLDC,MCAT,GLO1,GLP1R,GLP1R,MAT2B,GLS,GLRX,GLUD1,GLUD2,AMT,GLUL,GM2A,GMPR,GNA11,GNA11,GNA15,GNA15,GNAI1,GNAI1,GNAI2,GNAI2,GNAQ,GNAQ,GNAS,GNAS,GNB1,GNB1,GNB2,GNB2,GNB3,GNB3,GNG3,GNG3,GNG4,GNG4,GNG5,GNG5,GNG7,GNG7,GNG10,GNG10,GNG11,GNG11,GNGT1,GNGT1,GNGT2,GNGT2,GNS,ABO,GOT1,GOT1,GOT2,GOT2,GPC1,GPC1,GPD1,GPD2,GPI,SLCO1B3,PGM2L1,PLA2G4D,PGP,NUDT7,GDPD1,THEM5,CYP4A22,RIMKLA,SUMF1,CYP4V2,COX18,FFAR1,GPS2,GPT,GPX1,GPX1,GPX2,GPX2,GPX4,GPX4,ACAD9,FLVCR1,NDUFAF4,MED4,ORMDL2,N6AMT1,GSR,GSR,GSS,GSTA1,GSTA2,GSTA3,GSTA4,GSTM1,GSTM2,GSTM3,GSTM4,GSTM5,GSTP1,GSTT1,GSTT2,GSTZ1,CYP2S1,UQCR10,GRHL1,GUK1,GUK1,GUSB,UBIAD1,GYG1,GYG1,PYCR2,HILPDA,DSE,CERS2,DMGDH,LRP12,PSAT1,GYS1,GYS2,ACAA1,HSD17B10,HAGH,HADHA,HADHB,HADH,HAL,HAS1,HAS2,HAS3,HDC,HEXA,HEXB,HGD,PLA2G2E,ST6GALNAC6,NT5C,PIK3R4,HK1,HK2,ACACA,HK3,AOC2,HLCS,HMBS,HMGCL,HMGCR,HMGCS1,HMGCS2,AOX1,HMMR,HMOX1,HMOX2,HNMT,HNMT,ACACB,HPD,HPGD,HPGD,HPRT1,MMAB,HSD3B1,HSD3B2,HSD11B1,HSD11B2,HSD17B1,HSD17B3,HSD17B2,HSD17B4,ACADL,HSP90AA1,HSP90AB1,HSPG2,HSPG2,NDST1,APOA1,APOA1,APOA2,APOA2,HYAL1,IARS1,CHSY3,APOB,APOB,GPIHBP1,ENPP7,GADL1,NAT8L,ACADM,ARSI,ZDHHC21,ACER2,CYP26C1,ACSM4,IDH1,IDH2,IDH3A,IDH3B,IDH3G,IDI1,IDS,IDUA,APOC2,APOC2,APOC3,APOC3,HSD17B13,MOGAT3,DGAT2L6,ARSH,SLC35B2,SERINC2,APOE,APOE,ACSM2B,NUP43,NMNAT3,ACADS,APRT,ACADSB,IMPA1,IMPA2,IMPDH1,IMPDH2,IDO1,INPP1,INS,INPP4A,INPP5A,INPP5B,INPP5D,INPPL1,INSIG1,ACADVL,ITPA,ITPK1,ITPKA,ITPKB,ITPR1,ITPR1,ITPR2,ITPR2,ITPR3,ITPR3,IVD,GSTK1,KARS1,NDUFS7,KCNB1,ASPG,KCNC2,MIGA1,ARF1,PNPLA7,AGRN,AGRN,SLC27A1,KCNJ11,ARF3,CA13,ENTPD8,NHLRC1,KCNS3,KHK,ACAT1,ARG1,KPNB1,ARG2,LIPT2,TNFAIP8L3,GLYATL3,IYD,ACAT2,LALBA,NUDT19,SULT6B1,RPSA,AGMO,LBR,LDHA,LDHB,LDHC,LDLR,LHB,LIPE,FADS1,MED11,HACD4,LPL,PLPP6,LRP1,LRP2,LSS,LTA4H,ARNT,CYP4F3,LTC4S,ARNTL,LUM,MARCKS,ARSA,ARSB,STS,MAN2C1,MAN2B1,MANBA,MAOA,MAOB,ARSD,MARS1,ACBD7,MAT1A,MAT2A,ARSL,ARSF,CHST6,MDH1,MDH2,ME1,ME2,MGST1,MGST2,MGST3,ASAH1,ACHE,ALDH6A1,MOCS1,MOCS2,ASL,MPST,ABCC1,ASMT,ASNS,CTRB2,MTHFD2L,AKR1B15,ASPA,ASS1,SULT1A4,MTAP,ATP6,ATP8,COX1,COX2,COX3,CYTB,MTF1,NUDT1,MTHFD1,MTHFR,MTM1,ND1,ND2,ND3,ND4,ND5,ND6,MTR,MTRR,MMUT,MVD,MVK,NAGLU,NUBP1,NDUFA1,NDUFA2,NDUFA3,NDUFA4,NDUFA5,ACLY,NDUFA6,NDUFA7,NDUFA8,NDUFA9,NDUFA10,NDUFAB1,NDUFB1,NDUFB2,NDUFB3,ATIC,NDUFB4,NDUFB5,NDUFB6,NDUFB7,NDUFB8,NDUFB9,NDUFB10,NDUFC1,NDUFC2,NDUFS1,NDUFS2,NDUFS3,NDUFV1,NDUFS4,NDUFS5,NDUFS6,NDUFS8,NDUFV2,NDUFV3,RPL10A,NEU1,NEU2,NFYA,NFYA,NFYB,NFYB,NFYC,NFYC,NME1,NME2,NME3,NME4,NQO2,NNMT,NNMT,NOS3,PNP,NPAS2,NPAS2,NRF1,NT5E,NUP88,NUP98,CHAC2,OAT,OAZ1,OAZ2,OCA2,OCRL,ODC1,OMD,OGDH,OGN,ATP5F1A,ACO2,OSBP,OTC,ALDH7A1,OXCT1,RRM2B,G0S2,PLA2G3,DUOX2,PODXL2,CHST11,PAH,ATP5F1B,PNPLA8,NSDHL,ATP5F1C,PC,PCBD1,PCCA,PCCB,ACOX1,SLC35B3,MED31,COQ6,PCK1,PCK1,PCK2,RPS27L,NOSIP,DERA,APIP,NDUFA13,CRYL1,MLXIPL,MLXIPL,SEPSECS,ADIPOR1,ADIPOR1,ABHD5,MECR,NDUFAF1,RDH11,RPL26L1,SAR1B,ANGPTL4,INSIG2,HSD17B12,SLC45A2,AADAT,HSD17B11,HSD17B14,HAO2,DCXR,PLCE1,TACO1,ACP6,GLRX5,GLTP,COX16,PDZD11,NT5C3A,PIPOX,GMPR2,CD320,ECSIT,ATP5F1D,PCYT1A,TIMMDC1,CYP39A1,SLC25A37,MBTPS2,CHST15,PLA1A,CSAD,ATP5F1E,PRKAG2,PRKAG2,IP6K2,ISYNA1,HSD17B7,HACD3,ATP5PB,TRMT112,LARS1,SCLY,RAB14,GDE1,AZIN1,MED15,PIAS4,ATP5MC1,PDHA1,LIPT1,PDHA2,PDHB,PDK1,PDK2,CIAO2B,PDK3,PDK4,MPC1,ENPP1,ENPP2,OAZ3,ENPP3,ATP5MC2,ACSL5,CMPK1,UPB1,RTEL1,INPP5K,GNG13,GNG13,ATP5MC3,COQ3,PFAS,PFKFB1,PFKFB1,PFKFB2,PFKFB3,ATP5ME,PFKFB4,PFKL,PFKM,PFKP,ATP5PF,PGAM1,PGAM2,PGD,PGK1,PGK2,PGM1,ABCB1,ABCB4,PHKA1,PHKA1,PHKA2,PHKB,PHKB,PHKG1,PHKG1,PHKG2,PHYH,PIK3C2A,PIK3C2B,PIK3C2G,PIK3C3,PIK3CA,PIK3CB,PIK3CD,PIK3CG,PIK3R1,PIK3R2,PI4KA,PI4KB,PIP4K2A,PKLR,PKLR,PKM,PLA2G1B,PLA2G2A,PLA2G4A,PLA2G5,PLCB2,PLCB2,PLCB3,PLCB3,PLCB4,PLCD1,NUDT9,PLCG1,PANK1,PLCG2,PLD1,PLD1,NUP54,PLD2,PLD2,PLIN1,BCO1,ATP5PO,DUOX1,ACP5,SLC37A1,PNLIP,PNMT,CYCS,POLD1,GNG2,GNG2,HAO1,POMC,PON1,PON2,PON3,POR,PLEKHA5,CHPF2,UGT2B28,SMOX,HMGCLL1,NDUFB11,MTMR12,UGT1A10,UGT1A8,UGT1A7,UGT1A6,UGT1A5,UGT1A9,PARP14,PPA1,PPARA,PPARA,UGT1A4,UGT1A1,UGT1A3,PPARD,CRLS1,CROT,PPARG,MED1,MED1,PDP1,PPAT,CTSA,MED18,CKMT1A,RETSAT,MTMR10,ELOVL2,AUH,CYP2W1,IMPAD1,LPCAT2,PARP16,UCKL1,PLAAT2,PPOX,NMRK1,ACSM5,PPP1CA,MTARC2,PPP1CB,PPP1CC,MOCOS,SLC52A1,PDPR,PPP1R3C,MED9,PPP2CA,PPP2CB,PNPO,PPP2R1A,PPP2R1B,NUDT11,NADSYN1,TMLHE,ETNK2,PANK4,ADI1,NUDT15,PGM2,PPP2R5D,ACOXL,PI4K2B,OLAH,SPTLC3,RFK,AGPAT5,RNLS,ACER3,ABHD10,CHDH,PI4K2A,PPT1,CSGALNACT2,NDUFAF7,PRELP,ETNK1,CHST12,SMPD3,DHTKD1,STAB2,MIOX,MED29,MTMR8,SMPD4,PRKAA2,PRKAA2,PRKAB2,PRKAB2,PRKACA,PRKACA,PRKACB,PRKACB,PRKACG,PRKACG,VAC14,VAC14,NDC1,FAR2,PRKAR1A,PRKAR1A,NAXD,NUP133,CNDP2,PRKAR1B,PRKAR1B,AGK,PRKAR2A,PRKAR2A,PRKAR2B,PRKAR2B,PRKCA,LMBRD1,CSGALNACT1,PECR,ACOT13,TMEM126B,PRKD1,ACSS2,PRKG2,APOM,NDUFA12,GNG12,GNG12,PRODH,GPCPD1,KYAT3,PRPS1,PRPS2,LGMN,PRSS1,PRSS3,CTPS2,CHST7,PSAP,CYP26B1,INPP5E,ASAH2,PSMA1,PSMA2,PSMA3,SPHK2,PSMA4,PSMA5,PSMA6,PSMA7,PSMB1,AGPAT3,AGPAT4,BDH2,PSMB2,PSMB3,STARD7,STARD7,PSMB4,MCCC1,PSMB5,PSMB6,PSMB7,NT5M,PSMB8,PARP6,PSMB9,ADPRM,PSMB10,CHPT1,BAAT,PSMC1,PSMC2,AKR1B10,COQ9,CIAPIN1,PSMC3,PSMC4,PSMC5,PSMC6,PSMD1,PSMD2,ENTPD7,PSMD3,PSMD4,PNPLA2,CYSLTR2,PDSS2,PSMD5,PLAAT1,NUP107,LYRM4,PSMD7,PSMD8,PSMD9,SLC44A2,PSMD10,PSMD11,PSMD12,PSMD13,PSME1,PSME2,CEMIP,PSPH,PTEN,PTGDS,PTGIS,NMRAL1,AS3MT,PTGS1,PTGS2,UGT2A2,ESYT2,AHRR,RIMKLB,SERINC1,PDP2,CARNS1,PITPNM2,PLEKHA4,GPAM,GBA2,GBA3,TRIB3,G6PC2,PTPN13,CYP4F11,PTS,ABCD4,PXMP2,PYCR1,ALDH18A1,PCYT2,PYGB,PYGL,PYGM,PYGM,SQOR,SQOR,ENOPH1,PCTP,PRODH2,MID1IP1,QARS1,BCAT1,QDPR,RAB4A,RAB5A,BCAT2,BCHE,RAN,RANBP2,RAP1A,RARS1,PLAAT4,BCKDHA,PLEKHA1,PLEKHA2,ALOXE3,GNB4,GNB4,BCKDHB,RBP1,RBP2,RBP4,RHCE,RHD,SLC25A19,ELOVL5,PPCDC,HPSE2,AASDHPPT,EEFSEC,RORA,RPE,RPL3,RPL3L,RPL4,RPL5,RPL6,RPL7,RPL7A,RPL8,RPL9,RPL10,RPL11,RPL12,RPL13,RPL15,RPL17,RPL18,RPL18A,RPL19,RPL21,RPL22,RPL23A,RPL24,RPL26,RPL27,RPL30,RPL27A,RPL28,RPL29,RPL31,RPL32,RPL34,RPL35A,RPL36AL,RPL37,RPL37A,RPL38,RPL39,RPL41,RPL36A,RPLP0,RPLP1,RPLP2,RPS2,RPS3,RPS3A,RPS4X,SERINC4,RPS4Y1,RPS5,RPS6,RPS7,RPS8,RPS9,RPS10,RPS11,RPS12,RPS13,RPS14,RPS15,RPS15A,RPS16,RPS17,BDH1,RPS18,RPS19,RPS20,RPS21,RPS23,RPS24,RPS25,RPS26,RPS27,RPS27A,RPS27A,RPS28,RPS29,RRM1,RRM2,RXRA,RXRA,RXRB,ACSM3,SARS1,SAT1,SBF1,MSMO1,SC5D,SCD,BGN,SCO1,SCP2,BHMT,SDC1,SDC1,SRR,BCAN,SDC2,SDC2,SDC4,SDC4,ABHD4,SDHA,SDHA,SDHB,SDHB,SDHC,SDHC,SDHD,SDHD,CIDEC,SEC13,OXCT2,LHPP,RBKS,MCCC2,XYLT1,XYLT2,ACOT1,ACOT6,DPEP2,DPEP3,MMS19,BLVRA,MTMR14,CIAO3,SGSH,BLVRB,NDST4,PLA2G2F,FABP9,FABP12,SHMT1,HS3ST6,SHMT2,MTARC1,IPPK,CERK,ARV1,NMNAT1,CYP3A43,ST3GAL1,ST3GAL2,ELOVL1,ELOVL1,ST3GAL4,ETNPPL,ST3GAL3,LPIN3,AGXT2,AGXT2,SLC2A1,SLC2A2,SLC2A3,SLC3A2,NADK,PYCR3,SLC5A5,SLC6A7,SLC6A8,GSTT2B,SLC6A11,SLC6A12,NME1-NME2,SLC9A1,SLC10A1,SLC10A2,SLC16A1,SLC19A1,SLC25A1,SLCO1A2,SLC22A1,SLC22A3,SLC22A2,SLC22A5,PLEKHA3,AACS,BMX,CYP4F12,MTMR9,MTMR9,SMARCD3,SMARCD3,SMPD1,SMPD2,SMS,SUMO2,SNAP25,SORD,SP1,BPGM,SPR,BPHL,SQLE,SRD5A1,SRD5A2,AKR1D1,SREBF1,SREBF1,SREBF2,SREBF2,SRM,STAR,SULT1E1,ELOVL4,STK11,SULT1A2,STX1A,STXBP1,SULT1A1,SULT1A3,SULT1C2,BSG,SULT2B1,SUOX,SULT2A1,BST1,ABCC8,SURF1,MED22,VAMP2,BTD,SYT5,TALDO1,TAT,TAZ,TBL1X,TBL1X,TBXAS1,MLX,MLX,TCN1,TCN2,TDO2,TH,TSPO,THRSP,TK1,TK2,TKT,TM7SF2,TPH1,TPI1,TPMT,TPO,TPR,TPTE,TSHB,TST,TST,TTPA,TTR,D2HGDH,RPEL1,TXN,TXNRD1,TXNRD1,TYMS,TYR,TYRP1,UBA52,UBA52,UBB,UBC,UBE2I,UCP1,UCP2,UCP3,UGCG,UGDH,UGP2,UGT2B4,UGT2B7,UGT2B10,UGT2B15,UGT2B17,UGT8,UCK2,UMPS,NR1H2,UPP1,UQCRB,UQCRC1,UQCRC2,UQCRFS1,UQCRH,UROD,UROS,VDAC1,VDR,XDH,CA1,CA2,CA3,CA4,CA5A,CA6,CA7,CA9,CA12,CACNA1A,CACNA1C,CACNA1C,CACNA1D,CACNA1D,CACNA1E,LRP8,CACNB2,CACNB2,CACNB3,CACNB3,SLC25A20,CAD,VKORC1,GGCT,NUP37,SECISBP2,ELOVL6,DCTPP1,CHAC1,NDUFAF5,MBOAT7,FA2H,GDPD3,THTPA,HSD17B8,B3GNT4,GLB1L,EPM2A,SLC52A2,CHPF,ADIPOR2,ADIPOR2,CERS4,ACSS3,TNFAIP8L2,ARSJ,SRD5A3,PANK3,AIMP2,PARP8,PPCS,TBL1XR1,TBL1XR1,ACBD4,SEM1,UGT2A3,AGMAT,PIP4K2C,NUDT18,PLBD1,LPCAT1,NUP85,L2HGDH,SCD5,ELOVL7,PANK2,CALM1,CALM1,PTGES2,ASRGL1,MOGAT2,CHD9,CHD9,NUP214,COQ10B,ACSF2,NUBPL,RUFY1,HSD3B7,ITPKC,CUBN,MED28,FLAD1,PNPLA3,SLC25A16,COASY,CALM2,CALM2,PDHX,SLC19A3,ACAD10,SLC44A4,STARD5,CPTP,CYB5B,CALM3,CALM3,DDHD1,AAAS,SLC25A32,SLC7A5,PTDSS2,SGPP1,GDPD5,PLA2G12A,ACSBG2,ISCA1,AMN,SBF2,MED25,FAHD1,SLC25A28,SEH1L,NCOA3,PUDP,PNPLA4,ACOX2,ACOX3,PITPNM3,ELOVL3,ADPGK,CHST9,UCK1,NUDT12,PARP9,HYAL3,ESYT3,BCO2,SLC25A2,STARD3NL,PIP5K1A,PIP5K1B,PIP4K2B,PLA2G6,PLA2G6,PLA2G10,BRIP1,SLC25A11,ACAD11,FAR1,BBOX1,MRI1,MED10,COQ5,ACBD6,GNPAT,FAM120B,ACSS1,TPST2,TPST1,NT5C1A,DGAT2,MCEE,GPT2,PLEKHA8,FUT10,RAE1,GPAT3,PLCD4,ABHD14B,CBR4,PARP10,MFSD2A,ADO,MIGA2,COX14,PHYKPL,PIK3R3,NDST2,DDO,CYP4F2,CHST1,AGPS,HELZ2,HELZ2,SELENOI,DEGS1,KMO,PDXK,CAV1,CAV1,AKR7A2,SLC25A12,PLA2G4C,PLPP1,PLPP2,PLPP3,AOC3,AKR1C3,ABCB11,NCOA1,NCOA1,ALDH4A1,SERPINA6,JMJD7-PLA2G4B,HYAL2,DGAT1,B4GALT4,B4GALT3,B4GALT2,B3GALT4,B3GALNT1,B3GALT2,B3GALT1,ABCC3,MBTPS1,CBR1,CBR3,CBS,CBS,CDS2,MTMR1,FBP2,PEX11A,SUCLG2,SUCLG1,SUCLA2,INPP4B,CES2,IQGAP1,KYAT1,GMPS,HDAC3,SYNJ1,SYNJ2,VNN2,VNN1,SPHK1,SGPL1,SLC5A6,CPNE3,MTMR3,MTMR2,MTMR2,CPNE1,GYG2,CCNC,KYNU,WASL,PLCZ1,SLC25A21,NAT1,SLC25A14,HS6ST2,CH25H,MED30,RPL14,AIP,NFS1,PAPSS2,PAPSS2,PAPSS1,PAPSS1,PIP4P1,CERS5,MTMR6,MTMR7,MTMR7,MTMR4,SLC16A3,ACBD5,COX7A2L,ACY3,IDI2,NDUFAF2,HACD1,DSEL,VAPB,VAPA,LRAT,GLYATL1,LDHAL6B,CACNA2D2,CACNA2D2,AIMP1,TSPOAP1,G6PC3,MAPKAPK2,MED14,B3GNT7,NT5C1B,COQ10A,NAPRT,B4GALT6,B4GALT5,NDST3,RPL23,TPTE2,CPNE6,ADIPOQ,ADIPOQ,PPT2,COX5A,GRHPR,SLC22A13,CIAO1,HS6ST1,ORMDL1,ORMDL3,MED21,FADS2,FADS2,CYP7B1,GNG8,GNG8,ABCG2,ABCG2,CHST2,MED23,MED17,MED26,MED27,MED7,GSTO1,GGPS1,PCYT1B,CHST3,MED20,CD36,SLC25A27,PGS1,PSMF1,AKAP5,ACY1,SPTLC2,CD38,EEF1E1,TECR,ENTPD1,PTGES,ENTPD2,ENTPD6,ATP5MF,ENTPD3,MINPP1,ENTPD5,NR1D1,CLOCK,ENTPD4,CD44,PITPNM1,NCOR1,NCOR2,GDA,GNA14,GNA14,NUP155,SEC24C,PLCH2,HS2ST1,LPIN2,SDC3,SDC3,SLC25A44,TGS1,TGS1,PPIP5K1,NUP93,CDA,PTDSS1,IP6K1,NUP58,PSMD6,MED24,SEC24D,POM121,OSBPL2,FIG4,FIG4,ARNT2,LPGAT1,XYLB,HS3ST4,HS3ST3B1,HS3ST3A1,HS3ST2,HS3ST1,SLC23A2,SLC23A1,THRAP3,MED12,MED13,NR1H4,NUP153,SCO2",Metabolism,2263

R-HSA-1433557,"SH2B3,PTPRU,SH2B2,GRAP,CHEK1,CMA1,FER,FES,FYN,FYN,GRB2,GRB7,GRB10,HRAS,JAK2,KIT,KIT,KRAS,LCK,LCK,LYN,LYN,KITLG,KITLG,MMP9,NRAS,PIK3CA,PIK3R1,PIK3R2,PRKCA,PTPN6,PTPN11,RAC1,SOS1,SRC,SRC,STAT1,STAT3,STAT5A,STAT5B,TEC,VAV1,YES1,YES1,PIK3R3,SOCS1,CBL,SOCS6,GRAP2,GAB2",Signaling by SCF-KIT,50

R-HSA-1433559,"SH2B3,SH2B2,FYN,GRB2,KIT,KIT,LCK,LYN,KITLG,KITLG,PRKCA,PTPN6,SOS1,SRC,YES1,SOCS1,CBL,SOCS6",Regulation of KIT signaling,18

R-HSA-1433617,"LEFTY1,ACVR1C,DAND5,NODAL,CFC1,TDGF1,LEFTY2,ACVR1B,ACVR2A,ACVR2B,CER1",Regulation of signaling by NODAL,11

R-HSA-1442490,"ADAM10,COL1A1,COL1A1,COL1A2,COL1A2,COL2A1,COL2A1,COL3A1,COL3A1,COL4A1,COL4A1,COL4A2,COL4A2,COL4A3,COL4A3,COL4A4,COL4A4,COL4A5,COL4A5,COL4A6,COL4A6,COL5A1,COL5A2,COL6A1,COL6A2,COL6A3,COL7A1,COL7A1,COL8A1,COL8A1,COL8A2,COL8A2,COL9A1,COL9A2,COL9A3,COL10A1,COL10A1,COL11A1,COL11A1,COL11A2,COL11A2,COL12A1,COL13A1,COL15A1,COL16A1,COL17A1,COL19A1,COL6A6,COL26A1,CTSB,CTSD,CTSK,CTSL,TMPRSS6,TMPRSS6,ELANE,COL6A5,MMP1,MMP1,MMP2,MMP2,MMP3,MMP3,MMP7,MMP8,MMP9,MMP9,MMP10,MMP10,MMP11,MMP12,MMP13,MMP13,MMP14,MMP14,MMP15,MMP19,FURIN,FURIN,COL5A3,PRSS2,ADAM17,COL14A1,COL18A1,COL25A1,PHYKPL,ADAM9,COL23A1,MMP20",Collagen degradation,89

R-HSA-1445148,"TUBA1B,TUBB3,TUBB4A,TUBB4B,EXOC5,RAB10,YWHAQ,KIF3A,TUBA3E,EXOC3,TUBA3D,EXOC8,AKT1,AKT2,KIFAP3,TBC1D1,EXOC7,RHOQ,SFN,TUBB8,TUBB2B,LNPEP,RAB8A,MYH9,MYO1C,MYO5A,PRKAG2,RAB14,TUBA8,PRKAG3,EXOC6,PRKAA2,PRKAB1,PRKAB2,PRKAG1,EXOC1,EXOC2,RALGAPB,RALGAPA2,RAB4A,RAB13,RAC1,RALA,ACTB,EXOC4,SLC2A4,STX4,STXBP3,VAMP2,ACTG1,TUBA4A,TUBA3C,TUBB2A,YWHAB,YWHAE,YWHAG,YWHAH,YWHAZ,TUBA1A,ASPSCR1,TUBAL3,CALM1,CALM2,CALM3,TUBB1,TUBB6,TUBA1C,RAB11A,SNAP23,KIF3B,C2CD5,TBC1D4",Translocation of SLC2A4 (GLUT4) to the plasma membrane,72

R-HSA-1461957,"DEFB130B,DEFB4B,DEFB118,CCR6,DEFB104A,DEFB127,DEFB129,DEFB1,DEFB4A,DEFB105A,DEFB106A,DEFB107A,DEFB108B,DEFB110,DEFB113,DEFB114,DEFB115,DEFB116,DEFB119,DEFB121,DEFB123,DEFB124,DEFB125,DEFB128,DEFB130A,DEFB132,DEFB103A,DEFB107B,DEFB104B,DEFB106B,DEFB105B,DEFB103B,DEFB135,DEFB136,DEFB134,DEFB131A,TLR1,TLR2,CCR2,DEFB126",Beta defensins,40

R-HSA-1461973,"DEFB130B,DEFB4B,DEFB118,CCR6,DEFB104A,DEFB127,DEFB129,DEFA1,DEFA3,DEFA4,DEFA5,DEFA6,DEFB1,DEFB4A,DEFB105A,DEFB106A,DEFB107A,DEFB108B,DEFB110,DEFB113,DEFB114,DEFB115,DEFB116,DEFB119,DEFB121,DEFB123,DEFB124,DEFB125,DEFB128,DEFB130A,DEFB132,DEFB103A,ART1,DEFB107B,DEFB104B,DEFB106B,DEFB105B,DEFB103B,PRSS2,PRSS3,DEFB135,DEFB136,DEFB134,DEFB131A,TLR1,TLR2,DEFA1B,CCR2,DEFB126,CD4",Defensins,50

R-HSA-1462054,"DEFA1,DEFA3,DEFA4,DEFA5,DEFA6,ART1,PRSS2,PRSS3,DEFA1B,CD4",Alpha-defensins,10

R-HSA-1474151,"DHFR,AKT1,GCH1,GCHFR,HSP90AA1,NOS3,PRKG2,PTS,SPR,CALM1,CALM2,CALM3","Tetrahydrobiopterin (BH4) synthesis, recycling, salvage and regulation",12

R-HSA-1474165,"RAD50,CDK2,CDK4,LOC102724334,STAG1,SYCP2,STAG3,STAG2,DIDO1,ADAM30,DMC1,IZUMO4,CATSPER1,CATSPER2,H4-16,IZUMO2,H3C14,H2BU1,KCNU1,ZP1,SYNE2,SYNE1,SUN1,SPO11,ADAM2,H2BC1,SYCE2,CATSPERD,SUN2,POT1,TINF2,B4GALT1,MLH3,SMC1B,IZUMO1,PSMC3IP,H2AC8,H2AC7,H2AX,H2AZ1,H2BC5,H2BC3,H3-3A,H3-3B,HSPA2,H3C15,CATSPER3,CATSPER4,LMNA,LMNB1,MLH1,MRE11,MSH4,MSH5,NBN,ATM,H2AB1,ACR,OVGP1,SYCP3,TERF2IP,ATR,H4C15,H2AJ,TEX12,PRDM9,CATSPERG,ZP4,RAD21,RAD51,RAD51C,RBBP8,RPA1,RPA2,RPA3,BLM,SYCE3,ACD,H3C13,SPAM1,BRCA1,BRCA2,SYCP1,TERF1,TERF2,TOP3A,H2AC19,UBE2I,ZP2,ZP3,CATSPERB,SMC1A,H3-4,H4C9,H2AC14,H2AC6,H2AC4,H2AC18,H2AC20,H2BC8,H2BC13,H2BC15,H2BC14,H2BC7,H2BC6,H2BC9,H2BC10,H2BC4,H2BC17,H2BC21,H3C1,H3C4,H3C3,H3C6,H3C11,H3C8,H3C12,H3C10,H3C2,H4C1,H4C4,H4C6,H4C12,H4C11,H4C3,H4C8,H4C2,H4C5,H4C13,H4C14,MND1,HVCN1,FKBP6,H2BC12,ADAM21,ADAM20,H3C7,H2BC11,SMC3,CD9,SYCE1,H2AZ2,REC8",Reproduction,143

R-HSA-1474228,"ADAM8,ADAM10,ADAM10,CAPN9,MMP24,ADAMTS8,ADAMTS5,ADAMTS5,CAPN11,CAPN10,CMA1,COL1A1,COL1A1,COL1A2,COL1A2,COL2A1,COL2A1,COL3A1,COL3A1,COL4A1,COL4A1,COL4A2,COL4A2,COL4A3,COL4A3,COL4A4,COL4A4,COL4A5,COL4A5,COL4A6,COL4A6,COL5A1,COL5A2,COL6A1,COL6A2,COL6A3,COL7A1,COL7A1,COL8A1,COL8A1,COL8A2,COL8A2,COL9A1,COL9A2,COL9A3,COL10A1,COL10A1,COL11A1,COL11A1,COL11A2,COL11A2,COL12A1,COL13A1,COL15A1,COL16A1,COL17A1,COL19A1,COL6A6,COL26A1,CAPN12,CTRB1,CTSB,CTSD,CTSG,CTSK,CTSL,CTSV,CTSS,CTSS,DCN,TMPRSS6,TMPRSS6,ADAMTS16,ADAMTS18,ACAN,ACAN,ELANE,ELANE,A2M,ELN,FBN1,FBN1,FBN2,SCUBE3,FN1,FN1,NCSTN,CAPN7,COL6A5,OPTC,HSPG2,HSPG2,KLK2,KLKB1,CAPN8,LAMA3,LAMA3,LAMA5,LAMB1,LAMB3,LAMB3,LAMC1,LAMC2,LAMC2,MMP1,MMP1,MMP2,MMP2,MMP3,MMP3,MMP7,MMP7,MMP8,MMP9,MMP9,MMP10,MMP10,MMP11,MMP12,MMP12,MMP13,MMP13,MMP14,MMP14,MMP15,MMP15,MMP16,MMP17,MMP19,MMP19,CTRB2,CAPN14,NID1,NID1,FURIN,FURIN,COL5A3,SPOCK3,PLG,PLG,PRSS1,PRSS2,KLK7,HTRA1,PSEN1,ADAMTS9,BCAN,MMP25,BMP1,BMP1,CAPN15,SPP1,BSG,ADAM17,TIMP1,TIMP2,TLL1,TLL2,TPSAB1,CAPN5,COL14A1,SCUBE1,COL18A1,CAPN1,CAPN1,CAPN2,CAPN3,CAPNS1,CAPNS1,CAPN6,CAST,CASP3,CAPNS2,FBN3,COL25A1,PHYKPL,ADAM15,ADAM9,COL23A1,CAPN13,MMP20,MMP20,ADAMTS4,ADAMTS4,ADAMTS1,CD44,CDH1,CDH1",Degradation of the extracellular matrix,188

R-HSA-1474244,"ADAM8,ADAM10,ADAM10,LAMC3,LAMC3,CRTAP,FBLN5,FBLN5,P3H3,CAPN9,CEACAM8,MMP24,ADAMTS8,ADAMTS5,ADAMTS5,EMILIN1,EMILIN1,CAPN11,CAPN10,CMA1,COL1A1,COL1A1,COL1A2,COL1A2,COL2A1,COL2A1,COL3A1,COL3A1,COL4A1,COL4A1,COL4A2,COL4A2,COL4A3,COL4A3,COL4A4,COL4A4,COL4A5,COL4A5,COL4A6,COL4A6,COL5A1,COL5A1,COL5A2,COL5A2,COL6A1,COL6A1,COL6A2,COL6A2,COL6A3,COL6A3,COL7A1,COL7A1,COL8A1,COL8A1,COL8A2,COL8A2,COL9A1,COL9A1,COL9A2,COL9A2,COL9A3,COL9A3,COL10A1,COL10A1,COL11A1,COL11A1,COL11A2,COL11A2,COL12A1,COL13A1,COL15A1,COL16A1,COL16A1,COL17A1,COL17A1,COL19A1,COMP,COL6A6,COL6A6,COL26A1,HAPLN1,ADAMTS14,VCAN,NCAN,CAPN12,CTRB1,CTSB,CTSD,CTSG,CTSK,CTSL,CTSV,CTSS,CTSS,DAG1,DAG1,DCN,DCN,TMPRSS6,TMPRSS6,COL22A1,ADAMTS16,ADAMTS18,DMD,DMP1,ACAN,ACAN,DSPP,ELANE,ELANE,A2M,ELN,ELN,FBLN1,FBLN1,FBLN2,FBLN2,FBN1,FBN1,FBN2,FBN2,EFEMP1,EFEMP1,SCUBE3,FGA,FGB,FGF2,FGG,NID2,NID2,ITGA11,ITGA11,COLGALT2,FMOD,FN1,FN1,NCSTN,CAPN7,COL24A1,COL6A5,COL6A5,OPTC,PCOLCE2,BMP10,P4HA3,LAMA1,LAMA1,EFEMP2,EFEMP2,HSPG2,HSPG2,TNC,TNC,IBSP,IBSP,ICAM1,ICAM2,ICAM3,ICAM4,COL28A1,APP,ITGA6,ITGA6,ITGA1,ITGA1,ITGA2,ITGA2,ITGA2B,ITGA3,ITGA3,ITGA4,ITGA4,ITGA5,ITGA5,ITGA7,ITGA7,ITGA9,ITGA9,ITGAD,ITGAE,ITGAL,ITGAM,ITGAV,ITGAV,ITGAX,ITGB1,ITGB1,ITGB2,ITGB3,ITGB3,ITGB4,ITGB5,ITGB5,ITGB6,ITGB7,ITGB8,AGRN,AGRN,KDR,KLK2,KLKB1,CAPN8,LAMA2,LAMA2,LAMA3,LAMA3,LAMA4,LAMA4,LAMA5,LAMA5,LAMB1,LAMB1,LAMB2,LAMB2,LAMB3,LAMB3,LAMC1,LAMC1,LAMC2,LAMC2,LOX,LOXL1,LOXL2,LRP4,LTBP1,LTBP2,LTBP3,LUM,LUM,MATN1,MATN3,MFAP1,MFAP2,MFAP2,MFAP3,MFAP4,MMP1,MMP1,MMP2,MMP2,MMP3,MMP3,MMP7,MMP7,MMP8,MMP9,MMP9,MMP10,MMP10,MMP11,MMP12,MMP12,MMP13,MMP13,MMP14,MMP14,MMP15,MMP15,MMP16,MMP17,MMP19,MMP19,CTRB2,CAPN14,MUSK,CEACAM6,NCAM1,NID1,NID1,DDR2,P4HA1,P4HB,FURIN,FURIN,COL5A3,COL5A3,SERPINE1,F11R,SPOCK3,PCOLCE,TRAPPC4,PDGFA,PDGFB,PECAM1,PLEC,PLG,PLG,PLOD1,PLOD2,PPIB,ASPN,P3H2,PRKCA,PRSS1,PRSS2,KLK7,HTRA1,PSEN1,ADAMTS9,COL20A1,PTPRS,JAM2,NTN4,BGN,BGN,CEACAM1,SDC1,SDC1,BCAN,SDC2,SDC2,SDC4,SDC4,TNN,P3H1,MMP25,BMP1,BMP1,BMP2,BMP4,BMP7,CAPN15,DST,SPARC,SPP1,SPP1,BSG,ADAM17,TGFB1,TGFB1,TGFB2,TGFB2,TGFB3,TGFB3,THBS1,THBS1,TIMP1,TIMP2,ICAM5,TLL1,TLL2,TNR,TNXB,TPSAB1,CAPN5,TTR,COL14A1,COL14A1,VCAM1,VTN,VTN,VWF,DDR1,PXDN,COLGALT1,SCUBE1,ADAM12,MFAP5,MFAP5,COL18A1,COL21A1,MADCAM1,GDF5,CAPN1,CAPN1,CAPN2,CAPN3,CAPNS1,CAPNS1,CAPN6,CAST,CASP3,JAM3,EMILIN2,EMILIN2,LOXL4,LTBP4,CAPNS2,FBN3,FBN3,COL25A1,LOXL3,PHYKPL,ITGA10,ITGA10,ITGA8,ITGA8,COL27A1,CASK,ACTN1,SERPINH1,ADAM19,ADAM15,ADAM9,MATN4,P4HA2,PLOD3,EMILIN3,EMILIN3,COL23A1,COL23A1,CAPN13,MMP20,MMP20,NRXN1,ADAMTS4,ADAMTS4,ADAMTS3,ADAMTS2,ADAMTS1,CD44,CD47,SH3PXD2A,SDC3,SDC3,CD151,CDH1,CDH1",Extracellular matrix organization,418

R-HSA-1474290,"CRTAP,P3H3,COL1A1,COL1A2,COL2A1,COL3A1,COL4A1,COL4A2,COL4A3,COL4A4,COL4A5,COL4A6,COL5A1,COL5A2,COL6A1,COL6A2,COL6A3,COL7A1,COL8A1,COL8A2,COL9A1,COL9A2,COL9A3,COL10A1,COL11A1,COL11A2,COL12A1,COL13A1,COL15A1,COL16A1,COL17A1,COL19A1,COL6A6,COL26A1,ADAMTS14,CTSB,CTSL,CTSV,CTSS,COL22A1,COLGALT2,COL24A1,COL6A5,PCOLCE2,P4HA3,COL28A1,ITGA6,ITGB4,LAMA3,LAMB3,LAMC2,LOX,LOXL1,LOXL2,MMP3,MMP7,MMP9,MMP13,P4HA1,P4HB,COL5A3,PCOLCE,PLEC,PLOD1,PLOD2,PPIB,P3H2,COL20A1,P3H1,BMP1,DST,TLL1,TLL2,COL14A1,PXDN,COLGALT1,COL18A1,COL21A1,LOXL4,COL25A1,LOXL3,COL27A1,SERPINH1,P4HA2,PLOD3,COL23A1,MMP20,ADAMTS3,ADAMTS2,CD151",Collagen formation,90

R-HSA-1475029,"CA5B,CA14,CA13,CA1,CA2,CA3,CA4,CA5A,CA6,CA7,CA9,CA12",Reversible hydration of carbon dioxide,12

R-HSA-1480926,"HBA1,HBA2,HBB,AQP1,CYB5R4,CYB5R2,CYB5R1,RHAG,CYB5RL,SLC4A1,CA1,CA2,CA4",O2/CO2 exchange in erythrocytes,13

R-HSA-1482788,"PLA2G4B,LPCAT3,PLAAT3,PLA2G4E,MBOAT2,PLB1,PLA2R1,LPCAT4,TMEM86B,PLA2G4F,PLA2G2D,PLA2G4D,PLA2G2E,PLA2G3,PNPLA8,PLA2G1B,PLA2G2A,PLA2G4A,PLA2G5,LPCAT2,PLA2G2F,PLBD1,LPCAT1,PLA2G12A,PLA2G6,PLA2G10,PLA2G4C,JMJD7-PLA2G4B",Acyl chain remodelling of PC,28

R-HSA-1482798,"LCLAT1,HADHA,HADHB,PLA2G4A,TAZ,PLA2G6",Acyl chain remodeling of CL,6

R-HSA-1482801,"PLA2G4B,LPCAT3,PLAAT3,OSBPL5,OSBPL8,OSBPL10,PLA2G4E,MBOAT1,PLA2R1,LPCAT4,PLA2G4F,PLA2G2D,PLA2G4D,PLA2G2E,PLA1A,PLA2G1B,PLA2G2A,PLA2G4A,PLA2G5,PLA2G2F,PLA2G12A,PLA2G10,JMJD7-PLA2G4B",Acyl chain remodelling of PS,23

R-HSA-1482839,"PLA2G4B,LPCAT3,PLAAT3,PLAAT5,PLA2G4E,MBOAT2,MBOAT1,PLA2R1,LPCAT4,PLA2G4F,PLA2G2D,PLA2G4D,PLA2G2E,PLA2G3,PNPLA8,PLA2G1B,PLA2G2A,PLA2G4A,PLA2G5,PLAAT2,PLAAT1,PLAAT4,ABHD4,PLA2G2F,PLBD1,PLA2G12A,PLA2G6,PLA2G10,PLA2G4C,JMJD7-PLA2G4B",Acyl chain remodelling of PE,30

R-HSA-1482883,"MGLL,AWAT2,DGAT2L6,PNPLA2,PNPLA3,DGAT2,DGAT1",Acyl chain remodeling of DAG and TAG,7

R-HSA-1482922,"PLAAT3,PLA2G4E,PLA2R1,PLA2G4F,PLA2G2D,PLA2G4D,PLA2G2E,PLA2G1B,PLA2G2A,PLA2G4A,PLA2G5,PLA2G2F,MBOAT7,PLBD1,PLA2G12A,PLA2G10,PLA2G4C",Acyl chain remodelling of PI,17

R-HSA-1482925,"PLA2G4B,PLA2R1,LPCAT4,PLA2G4F,PLA2G2D,PLA2G4D,PLA2G2E,PLA2G3,PLA2G1B,PLA2G2A,PLA2G4A,PLA2G5,CRLS1,PLA2G2F,LPCAT1,PLA2G12A,PLA2G10,JMJD7-PLA2G4B,LPGAT1",Acyl chain remodelling of PG,19

R-HSA-1483076,CRLS1,Synthesis of CL,1

R-HSA-1483101,"PTDSS2,PTDSS1",Synthesis of PS,2

R-HSA-1483115,"PLA2G4B,PLA2G4E,PLA2G15,PLA2G4F,PLA2G4D,PLA2G4A,GPCPD1,PLBD1,PLA2G4C,JMJD7-PLA2G4B",Hydrolysis of LPC,10

R-HSA-1483148,"PTPMT1,PLD4,PLD6,PLD3,PLD1,PLD2,CDS2,PGS1",Synthesis of PG,8

R-HSA-1483152,"GPCPD1,PLA2G4C",Hydrolysis of LPE,2

R-HSA-1483166,"PLA2G4B,AGPAT1,AGPAT2,GPAT4,LIPI,GPAT2,LIPH,PLD6,PLA2R1,GPD1L,DDHD2,ALPI,LCLAT1,LPCAT4,PLA2G2D,GPD1,GPD2,PLA2G4D,PLA2G2E,MIGA1,ACP6,PLA2G1B,PLA2G2A,PLA2G4A,PLA2G5,PLD1,PLD2,AGPAT5,AGPAT3,AGPAT4,GPAM,PLA2G2F,LPCAT1,DDHD1,PLA2G12A,PLA2G10,GNPAT,GPAT3,MIGA2,JMJD7-PLA2G4B",Synthesis of PA,40

R-HSA-1483191,"CEPT1,PEMT,STARD10,CHAT,CHKA,CHKB,SLC44A3,CSNK2A1,CSNK2A2,CSNK2B,PHOSPHO1,ABHD3,SLC44A5,LPIN1,SLC44A1,ACHE,PCYT1A,STARD7,STARD7,CHPT1,SLC44A2,PCTP,BCHE,LPIN3,LPCAT1,SLC44A4,MFSD2A,PCYT1B,LPIN2",Synthesis of PC,29

R-HSA-1483196,PITPNB,PI and PC transport between ER and Golgi membranes,1

R-HSA-1483206,"PLA2G4B,LPCAT3,CEPT1,CDS1,PEMT,CDIPT,AGPAT1,AGPAT2,STARD10,CHAT,PLAAT3,CHKA,CHKB,MGLL,OSBPL5,OSBPL8,OSBPL10,PTPMT1,PLAAT5,PLD4,PLA2G4E,SLC44A3,MBOAT2,GPAT4,CSNK2A1,CSNK2A2,CSNK2B,LIPI,GPAT2,PLB1,MBOAT1,AWAT2,PHOSPHO1,ABHD3,LIPH,PLD6,PLD6,SLC44A5,PLA2R1,GPD1L,LPIN1,DDHD2,SLC44A1,PLD3,PLA2G15,PITPNB,PISD,ALPI,LCLAT1,LPCAT4,TMEM86B,PLA2G4F,PLA2G2D,CPNE7,GPD1,GPD2,PLA2G4D,HADHA,HADHB,PLA2G2E,DGAT2L6,MIGA1,ACHE,PLA2G3,PNPLA8,ACP6,PCYT1A,PLA1A,PLA2G1B,PLA2G2A,PLA2G4A,PLA2G5,PLD1,PLD1,PLD2,PLD2,CRLS1,LPCAT2,PLAAT2,ETNK2,AGPAT5,ETNK1,AGK,GPCPD1,AGPAT3,AGPAT4,STARD7,STARD7,CHPT1,PNPLA2,PLAAT1,SLC44A2,PITPNM2,GPAM,PCYT2,PCTP,BCHE,PLAAT4,ABHD4,PLA2G2F,ETNPPL,LPIN3,TAZ,MBOAT7,PLBD1,LPCAT1,PNPLA3,SLC44A4,DDHD1,PTDSS2,PLA2G12A,PITPNM3,PLA2G6,PLA2G6,PLA2G10,GNPAT,DGAT2,GPAT3,MFSD2A,MIGA2,SELENOI,PLA2G4C,JMJD7-PLA2G4B,DGAT1,CDS2,CPNE3,CPNE1,CPNE6,PCYT1B,PGS1,PITPNM1,LPIN2,PTDSS1,LPGAT1",Glycerophospholipid biosynthesis,134

R-HSA-1483213,"CEPT1,CHKA,CHKB,PHOSPHO1,LPIN1,PISD,ETNK2,ETNK1,PCYT2,ETNPPL,LPIN3,SELENOI,LPIN2",Synthesis of PE,13

R-HSA-1483226,"CDS1,CDIPT,PITPNM2,PITPNM3,PITPNM1",Synthesis of PI,5

R-HSA-1483248,"SACM1L,PI4KA,PI4K2B,SBF1,MTMR2",Synthesis of PIPs at the ER membrane,5

R-HSA-1483249,"NUDT4,NUDT3,PLCD3,IP6K3,PLD4,NUDT10,PLCH1,PLCB1,PPIP5K2,IPMK,INPP5J,IMPA1,IMPA2,INPP1,INPP4A,INPP5A,INPP5B,INPP5D,INPPL1,ITPK1,ITPKA,ITPKB,OCRL,PLCE1,IP6K2,ISYNA1,PLCB2,PLCB3,PLCB4,PLCD1,PLCG1,PLCG2,NUDT11,MIOX,PTEN,IPPK,MTMR9,CALM1,ITPKC,CALM2,CALM3,PLCD4,INPP4B,SYNJ1,PLCZ1,MTMR7,MINPP1,PLCH2,PPIP5K1,IP6K1",Inositol phosphate metabolism,50

R-HSA-1483255,"PNPLA6,TNFAIP8L1,ENPP6,PIK3R6,PIKFYVE,PIKFYVE,PLEKHA6,INPP5F,SACM1L,PIP5K1C,PIK3R5,PITPNB,TNFAIP8,INPP5J,GDPD1,PIK3R4,INPP4A,INPP5D,INPPL1,ARF1,PNPLA7,ARF3,TNFAIP8L3,MTM1,OCRL,RAB14,GDE1,INPP5K,PIK3C2A,PIK3C2B,PIK3C2G,PIK3C3,PIK3CA,PIK3CB,PIK3CD,PIK3CG,PIK3R1,PIK3R2,PI4KA,PI4KB,PIP4K2A,PLEKHA5,MTMR12,MTMR10,PI4K2B,PI4K2A,MTMR8,VAC14,VAC14,INPP5E,PTEN,PLEKHA4,PTPN13,RAB4A,RAB5A,PLEKHA1,PLEKHA2,SBF1,MTMR14,PLEKHA3,BMX,MTMR9,MTMR9,TPTE,GDPD3,TNFAIP8L2,PIP4K2C,RUFY1,GDPD5,SBF2,PIP5K1A,PIP5K1B,PIP4K2B,PLEKHA8,PIK3R3,MTMR1,INPP4B,SYNJ1,SYNJ2,MTMR3,MTMR2,MTMR2,PIP4P1,MTMR6,MTMR7,MTMR7,MTMR4,TPTE2,FIG4,FIG4",PI Metabolism,90

R-HSA-1483257,"PLA2G4B,LPCAT3,CEPT1,CDS1,PEMT,CDIPT,AGPAT1,AGPAT2,STARD10,PNPLA6,CHAT,PLAAT3,CHKA,CHKB,MGLL,OSBPL5,OSBPL8,OSBPL10,PTPMT1,PLAAT5,PLD4,PLA2G4E,TNFAIP8L1,SLC44A3,MBOAT2,ENPP6,GPAT4,CSNK2A1,CSNK2A2,CSNK2B,PIK3R6,LIPI,GPAT2,PLB1,MBOAT1,AWAT2,PHOSPHO1,ABHD3,PIKFYVE,PIKFYVE,LIPH,PLD6,PLD6,SLC44A5,PLEKHA6,INPP5F,SACM1L,PLA2R1,GPD1L,LPIN1,DDHD2,PIP5K1C,SLC44A1,PIK3R5,PLD3,PLA2G15,PITPNB,PISD,ALPI,LCLAT1,LPCAT4,TMEM86B,PLA2G4F,TNFAIP8,PLA2G2D,INPP5J,CPNE7,GPD1,GPD2,PLA2G4D,GDPD1,HADHA,HADHB,PLA2G2E,PIK3R4,DGAT2L6,INPP4A,INPP5D,INPPL1,MIGA1,ARF1,PNPLA7,ARF3,TNFAIP8L3,ACHE,MTM1,OCRL,PLA2G3,PNPLA8,ACP6,PCYT1A,PLA1A,RAB14,GDE1,INPP5K,PIK3C2A,PIK3C2B,PIK3C2G,PIK3C3,PIK3CA,PIK3CB,PIK3CD,PIK3CG,PIK3R1,PIK3R2,PI4KA,PI4KB,PIP4K2A,PLA2G1B,PLA2G2A,PLA2G4A,PLA2G5,PLD1,PLD1,PLD2,PLD2,PLEKHA5,MTMR12,CRLS1,MTMR10,LPCAT2,PLAAT2,ETNK2,PI4K2B,AGPAT5,PI4K2A,ETNK1,MTMR8,VAC14,VAC14,AGK,GPCPD1,INPP5E,AGPAT3,AGPAT4,STARD7,STARD7,CHPT1,PNPLA2,PLAAT1,SLC44A2,PTEN,PITPNM2,PLEKHA4,GPAM,PTPN13,PCYT2,PCTP,RAB4A,RAB5A,BCHE,PLAAT4,PLEKHA1,PLEKHA2,SBF1,ABHD4,MTMR14,PLA2G2F,ETNPPL,LPIN3,PLEKHA3,BMX,MTMR9,MTMR9,TAZ,TPTE,MBOAT7,GDPD3,TNFAIP8L2,PIP4K2C,PLBD1,LPCAT1,RUFY1,PNPLA3,SLC44A4,DDHD1,PTDSS2,GDPD5,PLA2G12A,SBF2,PITPNM3,PIP5K1A,PIP5K1B,PIP4K2B,PLA2G6,PLA2G6,PLA2G10,GNPAT,DGAT2,PLEKHA8,GPAT3,MFSD2A,MIGA2,PIK3R3,SELENOI,PLA2G4C,JMJD7-PLA2G4B,DGAT1,CDS2,MTMR1,INPP4B,SYNJ1,SYNJ2,CPNE3,MTMR3,MTMR2,MTMR2,CPNE1,PIP4P1,MTMR6,MTMR7,MTMR7,MTMR4,TPTE2,CPNE6,PCYT1B,PGS1,PITPNM1,LPIN2,PTDSS1,FIG4,FIG4,LPGAT1",Phospholipid metabolism,223

R-HSA-1489509,"CAMKK2,CAMKK2,ADCY1,AHCYL1,ADCY2,ADCY3,ADCY5,ADCY6,ADCY7,ADCY8,ADCY9,CREB1,GRK2,ADCY4,NBEA,ITPR1,ITPR2,ITPR3,KPNA2,PDE1A,PDE1C,PDE1B,PLCG1,PRKACA,PRKACA,PRKACB,PRKACB,PRKACG,PRKACG,PRKAR1A,PRKAR1B,PRKAR2A,PRKAR2A,PRKAR2B,PRKCA,PRKCD,PRKCE,PRKCG,PRKX,CALM1,CALM1,CALM2,CALM2,CALM3,CALM3,CAMK4,CAMK4,CAMK2A,CAMK2B,CAMK2D,CAMK2G,CAMKK1,CAMKK1",DAG and IP3 signaling,53

R-HSA-1500620,"RAD50,CDK2,CDK4,LOC102724334,STAG1,SYCP2,STAG3,STAG2,DIDO1,DMC1,H4-16,H3C14,H2BU1,SYNE2,SYNE1,SUN1,SPO11,H2BC1,SYCE2,SUN2,POT1,TINF2,MLH3,SMC1B,PSMC3IP,H2AC8,H2AC7,H2AX,H2AZ1,H2BC5,H2BC3,H3-3A,H3-3B,HSPA2,H3C15,LMNA,LMNB1,MLH1,MRE11,MSH4,MSH5,NBN,ATM,H2AB1,SYCP3,TERF2IP,ATR,H4C15,H2AJ,TEX12,PRDM9,RAD21,RAD51,RAD51C,RBBP8,RPA1,RPA2,RPA3,BLM,SYCE3,ACD,H3C13,BRCA1,BRCA2,SYCP1,TERF1,TERF2,TOP3A,H2AC19,UBE2I,SMC1A,H3-4,H4C9,H2AC14,H2AC6,H2AC4,H2AC18,H2AC20,H2BC8,H2BC13,H2BC15,H2BC14,H2BC7,H2BC6,H2BC9,H2BC10,H2BC4,H2BC17,H2BC21,H3C1,H3C4,H3C3,H3C6,H3C11,H3C8,H3C12,H3C10,H3C2,H4C1,H4C4,H4C6,H4C12,H4C11,H4C3,H4C8,H4C2,H4C5,H4C13,H4C14,MND1,FKBP6,H2BC12,H3C7,H2BC11,SMC3,SYCE1,H2AZ2,REC8",Meiosis,118

R-HSA-1500931,"CDH2,CDH3,CDH4,CDH5,CDH6,CDH7,CDH8,CDH9,CDH10,CDH11,CDH12,CDH13,CDH15,CDH17,CDH18,SIRPB1,CLDN16,FERMT2,COL17A1,CLDN4,CLDN3,CLDN7,CLDN23,SIRPA,SIRPA,CLDN19,CTNNA1,CTNNB1,CTNND1,PTK2B,SDK1,FLNA,FLNC,CLDN14,CD2AP,CADM1,CLDN15,FYB1,FYN,FYN,CADM2,NECTIN3,CLDN17,ANG,GRB2,PARVB,ILK,ITGA6,ITGB1,ITGB4,JUP,KRT5,KRT14,LAMA3,LAMB3,LAMC2,LIMS1,AFDN,AFDN,NCK1,NPHS1,NPHS1,CLDN20,CLDN11,F11R,PARD6A,CLDN18,PIK3CA,PIK3CB,PIK3R1,PIK3R2,PLEC,CLDN22,SDK2,FBLIM1,KIRREL1,KIRREL1,SIRPG,LIMS2,PARVA,PRKCI,PARD3,PTK2,PTPN6,PTPN11,CADM3,PVR,NECTIN1,NECTIN2,PXN,ACTB,ACTB,RSU1,MPP5,CDH24,SFTPD,SFTPA1,DST,SPTAN1,SPTBN1,SRC,TESK1,ACTG1,ACTG1,CLDN5,SFTPA2,TYROBP,VASP,NPHS2,ACTN4,NECTIN4,KIRREL2,NCK2,PARD6G,PARD6B,KIRREL3,CASK,ACTN1,ACTN1,ACTN2,IQGAP1,ACTN3,SKAP2,WASL,CLDN12,CLDN10,CLDN8,CLDN6,CLDN2,CLDN1,CLDN9,CRB3,ARHGEF6,CD47,CD47,CD151,MAGI2,CDH1",Cell-Cell communication,138

R-HSA-1502540,"FSTL3,FST,DRAP1,ACVR1C,ACVR1C,INHBA,INHBA,INHBB,INHBB,SMAD2,SMAD3,SMAD4,FOXH1,ACVR1B,ACVR2A,ACVR2A,ACVR2B,ACVR2B",Signaling by Activin,18

R-HSA-1538133,"CDK2,LIN54,LIN54,DYRK1A,E2F1,E2F1,E2F4,E2F4,E2F5,E2F5,LIN9,LIN9,HDAC1,HDAC1,MAX,MYBL2,MYC,PCNA,LIN37,LIN37,RBBP4,RBBP4,RBL1,RBL1,RBL2,RBL2,TFDP1,TFDP1,TFDP2,TFDP2,TOP2A,CCNA2,CCNA2,CCNA1,CCNE1,CCNE2,LIN52,LIN52,CDK1,CDC6,CDC25A",G0 and Early G1,41

R-HSA-156580,"NAT2,GLYAT,AKR1A1,BPNT1,UGT2B11,SLC26A1,UGT2A1,ACSM1,GSTO2,ACSM2A,GGT6,COMT,UGT3A1,CYP1A2,UGT3A2,SLC26A2,AHCY,ESD,GLYATL2,GSTA5,SLC35D1,SULT4A1,GGT1,GGT7,GGT5,OPLAH,SULT1C4,SULT1B1,GCLC,GCLM,HPGDS,MAT2B,N6AMT1,GSS,GSTA1,GSTA2,GSTA3,GSTA4,GSTM1,GSTM2,GSTM3,GSTM4,GSTM5,GSTP1,GSTT1,GSTT2,GSTZ1,ACSM4,SLC35B2,ACSM2B,GSTK1,GLYATL3,SULT6B1,MAT1A,MAT2A,MGST1,MGST2,MGST3,SULT1A4,MTR,MTRR,NNMT,CHAC2,PODXL2,SLC35B3,TRMT112,UGT2B28,UGT1A10,UGT1A8,UGT1A7,UGT1A6,UGT1A5,UGT1A9,UGT1A4,UGT1A1,UGT1A3,IMPAD1,ACSM5,ABHD10,CNDP2,AS3MT,UGT2A2,GSTT2B,SULT1E1,SULT1A2,SULT1A1,SULT1A3,SULT1C2,SULT2B1,SULT2A1,TPMT,UGDH,UGP2,UGT2B4,UGT2B7,UGT2B10,UGT2B15,UGT2B17,GGCT,CHAC1,UGT2A3,TPST2,TPST1,ABHD14B,NAT1,PAPSS2,PAPSS1,GLYATL1,GSTO1",Phase II - Conjugation of compounds,109

R-HSA-156581,"COMT,CYP1A2,AHCY,MAT2B,N6AMT1,MAT1A,MAT2A,MTR,MTRR,NNMT,TRMT112,AS3MT,TPMT,GSTO1",Methylation,14

R-HSA-156582,"NAT2,NAT1",Acetylation,2

R-HSA-156584,"BPNT1,SLC26A1,SLC26A2,SULT4A1,SULT1C4,SULT1B1,SLC35B2,SULT6B1,SULT1A4,PODXL2,SLC35B3,IMPAD1,SULT1E1,SULT1A2,SULT1A1,SULT1A3,SULT1C2,SULT2B1,SULT2A1,TPST2,TPST1,ABHD14B,PAPSS2,PAPSS1",Cytosolic sulfonation of small molecules,24

R-HSA-156587,"GLYAT,ACSM1,ACSM2A,GLYATL2,ACSM4,ACSM2B,GLYATL3,ACSM5,GLYATL1",Amino Acid conjugation,9

R-HSA-156588,"UGT2B11,UGT2A1,UGT3A1,UGT3A2,SLC35D1,UGT2B28,UGT1A10,UGT1A8,UGT1A7,UGT1A6,UGT1A5,UGT1A9,UGT1A4,UGT1A1,UGT1A3,ABHD10,UGT2A2,UGDH,UGP2,UGT2B4,UGT2B7,UGT2B10,UGT2B15,UGT2B17,UGT2A3",Glucuronidation,25

R-HSA-156590,"AKR1A1,GSTO2,GGT6,ESD,GSTA5,GGT1,GGT7,GGT5,OPLAH,GCLC,GCLM,HPGDS,GSS,GSTA1,GSTA2,GSTA3,GSTA4,GSTM1,GSTM2,GSTM3,GSTM4,GSTM5,GSTP1,GSTT1,GSTT2,GSTZ1,GSTK1,MGST1,MGST2,MGST3,CHAC2,CNDP2,GSTT2B,GGCT,CHAC1,GSTO1",Glutathione conjugation,36

R-HSA-1566948,"FBLN5,FBLN5,EMILIN1,EMILIN1,ELN,ELN,FBLN1,FBLN1,FBLN2,FBLN2,FBN1,FBN1,FBN2,FBN2,EFEMP1,EFEMP1,FN1,BMP10,EFEMP2,EFEMP2,ITGA5,ITGAV,ITGB1,ITGB3,ITGB5,ITGB6,ITGB8,LOX,LOXL1,LOXL2,LTBP1,LTBP2,LTBP3,MFAP1,MFAP2,MFAP2,MFAP3,MFAP4,FURIN,BMP2,BMP4,BMP7,TGFB1,TGFB2,TGFB3,VTN,MFAP5,MFAP5,GDF5,EMILIN2,EMILIN2,LOXL4,LTBP4,FBN3,FBN3,LOXL3,ITGA8,EMILIN3,EMILIN3",Elastic fibre formation,59

R-HSA-1566977,"CEACAM8,FN1,ITGA5,ITGB1,CEACAM6,CEACAM1",Fibronectin matrix formation,6

R-HSA-156711,"CENPF,LIN54,EP300,FOXM1,LIN9,MYBL2,PLK1,LIN37,RBBP4,WEE1,CCNB1,PKMYT1,CCNB2,LIN52,CDC25A,CDC25C",Polo-like kinase mediated events,16

R-HSA-156827,"EIF3M,RPL35,RPL39L,RPS4Y2,RPL10L,EIF1AX,EIF2S1,EIF2S3,EIF4A1,EIF4A2,EIF4B,EIF4E,EIF4G1,RPL22L1,RPL13A,RPL36,PABPC1,EIF3K,EIF3E,RPSA,RPL10A,RPS27L,RPL26L1,EIF3L,RPL3,RPL3L,RPL4,RPL5,RPL6,RPL7,RPL7A,RPL8,RPL9,RPL10,RPL11,RPL12,RPL13,RPL15,RPL17,RPL18,RPL18A,RPL19,RPL21,RPL22,RPL23A,RPL24,RPL26,RPL27,RPL30,RPL27A,RPL28,RPL29,RPL31,RPL32,RPL34,RPL35A,RPL36AL,RPL37,RPL37A,RPL38,RPL39,RPL41,RPL36A,RPLP0,RPLP1,RPLP2,RPS2,RPS3,RPS3A,RPS4X,RPS4Y1,RPS5,RPS6,RPS7,RPS8,RPS9,RPS10,RPS11,RPS12,RPS13,RPS14,RPS15,RPS15A,RPS16,RPS17,RPS18,RPS19,RPS20,RPS21,RPS23,RPS24,RPS25,RPS26,RPS27,RPS27A,RPS28,RPS29,UBA52,EIF4H,EIF3A,EIF3B,EIF3C,EIF3D,EIF3F,EIF3G,EIF3H,EIF3I,EIF3J,EIF2S2,RPL14,RPL23",L13a-mediated translational silencing of Ceruloplasmin expression,111

R-HSA-156842,"RPL35,RPL39L,RPS4Y2,RPL10L,EEF1A1,EEF1A1,EEF1A2,EEF1B2,EEF1D,EEF1G,EEF2,EEF2,RPL22L1,RPL13A,RPL36,RPSA,RPL10A,RPS27L,RPL26L1,RPL3,RPL3L,RPL4,RPL5,RPL6,RPL7,RPL7A,RPL8,RPL9,RPL10,RPL11,RPL12,RPL13,RPL15,RPL17,RPL18,RPL18A,RPL19,RPL21,RPL22,RPL23A,RPL24,RPL26,RPL27,RPL30,RPL27A,RPL28,RPL29,RPL31,RPL32,RPL34,RPL35A,RPL36AL,RPL37,RPL37A,RPL38,RPL39,RPL41,RPL36A,RPLP0,RPLP1,RPLP2,RPS2,RPS3,RPS3A,RPS4X,RPS4Y1,RPS5,RPS6,RPS7,RPS8,RPS9,RPS10,RPS11,RPS12,RPS13,RPS14,RPS15,RPS15A,RPS16,RPS17,RPS18,RPS19,RPS20,RPS21,RPS23,RPS24,RPS25,RPS26,RPS27,RPS27A,RPS28,RPS29,UBA52,RPL14,RPL23",Eukaryotic Translation Elongation,95

R-HSA-156902,"RPL35,RPL39L,RPS4Y2,RPL10L,EEF1A1,EEF2,EEF2,RPL22L1,RPL13A,RPL36,RPSA,RPL10A,RPS27L,RPL26L1,RPL3,RPL3L,RPL4,RPL5,RPL6,RPL7,RPL7A,RPL8,RPL9,RPL10,RPL11,RPL12,RPL13,RPL15,RPL17,RPL18,RPL18A,RPL19,RPL21,RPL22,RPL23A,RPL24,RPL26,RPL27,RPL30,RPL27A,RPL28,RPL29,RPL31,RPL32,RPL34,RPL35A,RPL36AL,RPL37,RPL37A,RPL38,RPL39,RPL41,RPL36A,RPLP0,RPLP1,RPLP2,RPS2,RPS3,RPS3A,RPS4X,RPS4Y1,RPS5,RPS6,RPS7,RPS8,RPS9,RPS10,RPS11,RPS12,RPS13,RPS14,RPS15,RPS15A,RPS16,RPS17,RPS18,RPS19,RPS20,RPS21,RPS23,RPS24,RPS25,RPS26,RPS27,RPS27A,RPS28,RPS29,UBA52,RPL14,RPL23",Peptide chain elongation,90

R-HSA-157118,"HDAC6,HDAC5,MAMLD1,MAMLD1,PSME3,ADAM10,ADAM10,PSMD14,CDK8,LOC102724334,IKZF1,ST3GAL6,TACC3,TMED2,WWP2,DTX2,DTX2,H4-16,H3C14,CNTN1,H2BU1,CREB1,CREBBP,CREBBP,MIB2,PTCRA,JAG1,JAG1,DTX1,DTX1,E2F1,E2F1,E2F3,E2F3,AGO3,AGO4,EGF,EGFR,ELF3,EP300,EP300,AKT1,FABP7,FCER2,SNW1,SNW1,TNRC6B,PLXND1,DTX4,DTX4,FLT4,WWC1,NCSTN,NCSTN,HEY1,HEY1,HEY2,HEY2,POFUT1,H2BC1,KAT2A,KAT2A,HEYL,HEYL,AGO1,B4GALT1,NBEA,AGO2,TNRC6A,DLL1,DLL1,GZMB,H2AC8,H2AC7,H2AX,H2AZ1,H2BC5,H2BC3,H3-3A,H3-3B,HDAC1,HDAC2,HIF1A,HES1,HES1,H3C15,RBPJ,RBPJ,JAG2,JAG2,JUN,HES5,HES5,LFNG,ARRB1,SMAD3,ARRB2,MDK,MFNG,MOV10,MYC,H2AB1,NOTCH1,NOTCH1,NOTCH2,NOTCH2,NOTCH3,NOTCH3,NOTCH4,NOTCH4,ATP2A1,ATP2A2,ATP2A3,YBX1,FURIN,PBX1,APH1A,APH1A,SIRT6,HDAC7,NEURL1B,DLL4,DLL4,FBXW7,H4C15,MAML3,MAML3,H2AJ,PRKCI,PSENEN,PSENEN,HDAC8,PSEN1,PSEN1,PSEN2,PSEN2,PSMA1,PSMA2,PSMA3,PSMA4,PSMA5,PSMA6,PSMA7,PSMB1,PSMB2,PSMB3,PSMB4,PSMB5,PSMB6,PSMB7,PSMB8,PSMB9,POGLUT1,PSMB10,PSMC1,PSMC2,PSMC3,PSMC4,PSMC5,PSMC6,PSMD1,PSMD2,PSMD3,PSMD4,PSMD5,PSMD7,PSMD8,PSMD9,PSMD10,PSMD11,PSMD12,PSMD13,PSME1,PSME2,MIB1,TNRC6C,RAB6A,ACTA2,CCND1,RFNG,RPS27A,RPS27A,SEL1L,ST3GAL4,ST3GAL3,SKP1,H3C13,STAT1,STAT1,ADAM17,TBL1X,TFDP1,TFDP1,TFDP2,TFDP2,TLE1,TLE2,TLE3,TLE4,TP53,H2AC19,UBA52,UBA52,UBB,UBB,UBC,UBC,YWHAZ,YWHAZ,TBL1XR1,SEM1,HDAC11,H4C9,H2AC14,H2AC6,H2AC4,H2AC18,H2AC20,H2BC8,H2BC13,H2BC15,H2BC14,H2BC7,H2BC6,H2BC9,H2BC10,APH1B,APH1B,H2BC4,H2BC17,H2BC21,H3C1,H3C4,H3C3,H3C6,H3C11,H3C8,H3C12,H3C10,H3C2,H4C1,H4C4,H4C6,H4C12,H4C11,H4C3,H4C8,H4C2,H4C5,H4C13,H4C14,ITCH,ITCH,HDAC10,MAML2,MAML2,CUL1,H2BC12,RUNX1,NUMB,DLK1,HDAC3,KAT2B,KAT2B,CCNC,H3C7,H2BC11,NEURL1,DNER,H2AZ2,PSMF1,NCOR1,NCOR2,HDAC9,HDAC4,DLGAP5,MAML1,MAML1,PSMD6,RBX1",Signaling by NOTCH,280

R-HSA-157579,"TEN1,CDK2,LOC102724334,POLD3,RUVBL2,H4-16,H2BU1,DAXX,DKC1,DNA2,FEN1,ANKRD28,POLA2,H2BC1,POT1,TINF2,H2AC8,H2AC7,H2AX,H2AZ1,H2BC5,H2BC3,H3-3A,H3-3B,LIG1,H2AB1,PCNA,RTEL1,POLA1,POLD1,POLD2,POLR2A,POLR2B,POLR2C,POLR2D,POLR2E,POLR2F,POLR2G,POLR2H,POLR2I,TERF2IP,POLR2J,POLR2K,POLR2L,GAR1,ATRX,CHTF8,WRAP53,SHQ1,PPP6R3,PPP6C,H4C15,NOP10,PRIM1,PRIM2,NHP2,H2AJ,POLD4,RFC1,RFC2,RFC3,RFC4,RFC5,RPA1,RPA2,RPA3,CHTF18,BLM,ACD,TERF1,TERF2,TERT,H2AC19,WRN,DSCC1,STN1,PIF1,CTC1,H3-4,H4C9,H2AC14,H2AC6,H2AC4,H2AC18,H2AC20,H2BC8,H2BC13,H2BC15,H2BC14,H2BC7,H2BC6,H2BC9,H2BC10,H2BC4,H2BC17,H2BC21,H4C1,H4C4,H4C6,H4C12,H4C11,H4C3,H4C8,H4C2,H4C5,H4C13,H4C14,H2BC12,RUVBL1,CCNA2,CCNA1,H2BC11,H2AZ2",Telomere Maintenance,113

R-HSA-157858,"GJC1,TUBA1B,TUBA1B,TUBB3,TUBB3,TUBB4A,TUBB4A,TUBB4B,TUBB4B,GJB6,TUBA3E,TUBA3E,TUBA3D,TUBA3D,AP2M1,CLTA,CLTB,CLTC,GJD3,GJB4,DAB2,DNM1,DNM2,GJD4,GJA1,GJA1,GJA3,GJA4,GJA5,GJA8,GJB1,GJB1,GJB2,GJB2,GJB3,GJB5,TUBB8,TUBB8,TUBB2B,TUBB2B,GJB7,MYO6,TUBA8,TUBA8,GJC2,GJD2,ACTB,SRC,TJP1,TJP1,ACTG1,TUBA4A,TUBA4A,TUBA3C,TUBA3C,TUBB2A,TUBB2A,TUBA1A,TUBA1A,TUBAL3,TUBAL3,GJA9,TUBB1,TUBB1,CLTCL1,TUBB6,TUBB6,GJA10,TUBA1C,TUBA1C",Gap junction trafficking and regulation,70

R-HSA-15869,"ADA,NT5C1B-RDH14,AK6,DNPH1,PAICS,NUDT5,AK7,ADSS1,NUDT16,ADK,CTPS1,UPP2,ADSL,AK8,ADSS2,ADAL,DCK,DCTD,DGUOK,DHODH,DPYD,DPYS,DTYMK,DUT,TYMP,AK1,AK2,AK4,AK9,NT5C2,SAMHD1,NUDT13,GART,AK5,AMPD1,AMPD2,AMPD3,GLRX,GMPR,GPX1,GSR,GUK1,NT5C,HPRT1,APRT,IMPDH1,IMPDH2,ITPA,ENTPD8,NUDT1,ATIC,NME1,NME2,NME3,NME4,PNP,NT5E,RRM2B,NT5C3A,GMPR2,CMPK1,UPB1,PFAS,NUDT9,PPAT,UCKL1,NUDT15,CTPS2,NT5M,ADPRM,ENTPD7,RRM1,RRM2,LHPP,AGXT2,NME1-NME2,TK1,TK2,TXN,TXNRD1,TYMS,UCK2,UMPS,UPP1,XDH,CAD,DCTPP1,NUDT18,PUDP,UCK1,NT5C1A,GMPS,NT5C1B,ENTPD1,ENTPD2,ENTPD6,ENTPD3,ENTPD5,ENTPD4,GDA,CDA",Metabolism of nucleotides,101

R-HSA-1592230,"ATP5PD,CARM1,NCOA2,ATP5MG,MTX2,PPARGC1A,PPARGC1A,IMMT,POLG2,MICOS13,PPARGC1B,PPARGC1B,CREB1,CREB1,ATF2,CREBBP,APOOL,MAPK14,CRTC2,ESRRA,ESRRA,ALAS1,NCOA6,PPRC1,CRTC1,SIRT5,SIRT4,SIRT3,GABPA,GABPB1,SAMM50,DMAC2L,GLUD1,GLUD2,HCFC1,HSPA9,IDH2,MEF2C,MEF2D,MICOS10,ATP6,ATP8,MTX1,NRF1,NRF1,ATP5F1A,ATP5F1B,ATP5F1C,TFB1M,ATP5F1D,ATP5F1E,PRKAG2,ATP5PB,ATP5MC1,ATP5MC2,ATP5MC3,ATP5ME,ATP5PF,PRKAG3,ATP5PO,CYCS,POLRMT,PPARA,MED1,CHCHD3,PRKAA2,PRKAB1,PRKAB2,PRKAG1,DNAJC11,ACSS2,MAPK11,TWNK,RXRA,MAPK12,TFB2M,CRTC3,SMARCD3,SOD2,SSBP1,TBL1X,TFAM,TFAM,APOO,TBL1XR1,MTERF1,CALM1,CHD9,CALM2,CALM3,CAMK4,CHCHD6,PERM1,HELZ2,NCOA1,TMEM11,HDAC3,ATP5MF,NR1D1,NCOR1,TGS1",Mitochondrial biogenesis,101

R-HSA-159227,"ALYREF,NXF1,NUP50,NUP42,NUP35,EIF4E,NCBP2,NUP205,NUP210,NUP160,NUP188,NUP62,NUP43,NCBP1,NUP88,NUP98,NUP54,NDC1,NUP133,NUP107,RANBP2,SEC13,TPR,NUP37,NUP85,NUP214,AAAS,SEH1L,RAE1,NUP155,NUP93,NUP58,POM121,NUP153",Transport of the SLBP independent Mature mRNA,34

R-HSA-159230,"ALYREF,NXF1,NUP50,NUP42,NUP35,EIF4E,NCBP2,NUP205,NUP210,NUP160,NUP188,NUP62,NUP43,NCBP1,NUP88,NUP98,NUP54,NDC1,NUP133,NUP107,RANBP2,SEC13,TPR,SLBP,NUP37,NUP85,NUP214,AAAS,SEH1L,RAE1,NUP155,NUP93,NUP58,POM121,NUP153",Transport of the SLBP Dependant Mature mRNA,35

R-HSA-159231,"ALYREF,NXF1,NUP50,CPSF4,NUP42,NUP35,EIF4E,NCBP2,NUP205,NUP210,NUP160,NUP188,NUP62,CPSF1,NUP43,NCBP1,NUP88,NUP98,CPSF3,NUP54,CPSF2,WDR33,NDC1,NUP133,NUP107,RANBP2,SEC13,TPR,NUP37,NUP85,NUP214,AAAS,FIP1L1,SYMPK,SEH1L,RAE1,NUP155,NUP93,NUP58,POM121,NUP153",Transport of Mature mRNA Derived from an Intronless Transcript,41

R-HSA-159234,"ALYREF,NXF1,NUP50,CPSF4,NUP42,NUP35,EIF4E,NCBP2,NUP205,NUP210,NUP160,NUP188,NUP62,CPSF1,NUP43,NCBP1,NUP88,NUP98,CPSF3,NUP54,CPSF2,WDR33,NDC1,NUP133,NUP107,RANBP2,SEC13,TPR,SLBP,NUP37,NUP85,NUP214,AAAS,FIP1L1,SYMPK,SEH1L,RAE1,NUP155,NUP93,NUP58,POM121,NUP153",Transport of Mature mRNAs Derived from Intronless Transcripts,42

R-HSA-159236,"ALYREF,DDX39A,SRRM1,U2AF1L5,NXF1,SLU7,NUP50,RNPS1,NUP42,U2AF2,NUP35,U2AF1L4,CASC3,NCBP2,NUP205,NUP210,NUP160,NUP188,NUP62,CHTOP,GLE1,NXT1,NUP43,MAGOH,NCBP1,NUP88,NUP98,LUZP4,CDC40,NUP54,MAGOHB,NDC1,NUP133,NXF2,NUP107,THOC2,RANBP2,SEC13,SRSF1,SRSF2,SRSF3,SRSF4,SRSF5,SRSF6,SRSF7,UPF3B,TPR,NXF2B,U2AF1,NUP37,DDX39B,THOC6,NUP85,THOC7,NUP214,AAAS,SEH1L,FYTTD1,POLDIP3,THOC3,SARNP,RAE1,THOC5,SRSF9,SRSF11,NUP155,NUP93,EIF4A3,DHX38,NUP58,ZC3H11A,POM121,RBM8A,NUP153,THOC1",Transport of Mature mRNA derived from an Intron-Containing Transcript,75

R-HSA-1592389,"MMP24,CMA1,CTRB1,CTSG,CTSK,CTSV,ELANE,KLK2,KLKB1,MMP1,MMP2,MMP3,MMP7,MMP8,MMP9,MMP10,MMP11,MMP13,MMP14,MMP15,MMP16,MMP17,CTRB2,FURIN,SPOCK3,PLG,PRSS1,PRSS2,MMP25,TIMP1,TIMP2,TPSAB1,COL18A1",Activation of Matrix Metalloproteinases,33

R-HSA-159418,"NCOA2,SLCO1B1,SLC27A5,ALB,FABP6,SLCO1B3,BAAT,RXRA,SLC10A1,SLC10A2,SLCO1A2,STARD5,ABCB11,NCOA1,ABCC3,NR1H4",Recycling of bile acids and salts,16

R-HSA-159424,"GLYAT,ACSM1,ACSM2A,GLYATL2,ACSM4,ACSM2B,GLYATL3,ACSM5,GLYATL1",Conjugation of carboxylic acids,9

R-HSA-159740,"F2,F7,F9,F10,GAS6,GGCX,GGCX,PROC,PROS1,BGLAP,PROZ",Gamma-carboxylation of protein precursors,11

R-HSA-159763,"F2,F7,F9,F10,GAS6,PROC,PROS1,BGLAP,PROZ",Transport of gamma-carboxylated protein precursors from the endoplasmic reticulum to the Golgi apparatus,9

R-HSA-159782,"F2,F7,F9,F10,GAS6,FURIN,PROC,PROS1,BGLAP,PROZ",Removal of aminoterminal propeptides from gamma-carboxylated proteins,10

R-HSA-159854,"F2,F7,F9,F10,GAS6,GAS6,GGCX,GGCX,FURIN,PROC,PROS1,BGLAP,PROZ","Gamma-carboxylation, transport, and amino-terminal cleavage of proteins",13

R-HSA-1606322,"RIPK3,CHUK,NLRP4,TICAM1,DHX9,DTX4,NKIRAS2,NKIRAS1,TBK1,TBK1,IKBKB,IRF3,IRF3,MYD88,NFKB1,NFKB2,NFKBIA,NFKBIB,RELA,TLR3,ZBP1,ZBP1,IKBKG,RIPK1",ZBP1(DAI) mediated induction of type I IFNs,24

R-HSA-1606341,"NLRP4,DTX4,TBK1,TBK1,IRF3,IRF3,ZBP1,ZBP1",IRF3 mediated activation of type 1 IFN,8

R-HSA-1614517,"TSTD1,SLC25A10,ETHE1,SQOR,SQOR,SUOX,TST",Sulfide oxidation to sulfate,7

R-HSA-1614558,"TSTD1,CDO1,SLC25A10,CTH,ETHE1,TXN2,GOT2,GADL1,MPST,CSAD,SQOR,SQOR,SUOX,TST,TST,ADO",Degradation of cysteine and homocysteine,16

R-HSA-1614603,"CBSL,CTH,CBS",Cysteine formation from homocysteine,3

R-HSA-1614635,"TSTD1,CBSL,CDO1,SLC25A10,CTH,AHCY,ETHE1,BHMT2,TXN2,GOT1,GOT2,GADL1,MAT1A,MPST,MTAP,MTR,MTRR,APIP,CSAD,ADI1,SQOR,SQOR,ENOPH1,BHMT,SUOX,TST,TST,MRI1,ADO,CBS",Sulfur amino acid metabolism,30

R-HSA-162582,"AKT3,AKT3,ABI1,ABI1,HDAC6,HDAC5,BCL2L11,SH2B3,INSL5,FRAT1,ARHGEF33,CDH5,MAMLD1,MAMLD1,RANBP9,CENPS-CORT,PMF1-BGLAP,CHURC1-FNTB,COMMD3-BMI1,COMMD3-BMI1,NR1H3,PTPRU,GPC6,ARPC5,ARPC5,ARPC4,ARPC4,ARPC3,ARPC3,ARPC1B,ARPC1B,ACTR3,ACTR3,ACTR2,ACTR2,NPY4R2,OPN1MW3,ARPC2,ARPC2,ARL4C,RASGRP1,RASGRP1,DNAL4,FAM13A,ABI2,ABI2,RASA4,LPAR6,WASF2,WASF2,CDK2,DHRS9,CDK4,RNF41,PSME3,PSME3,ADAM10,ADAM10,CDK5,CDK5,CALCRL,PSMD14,PSMD14,TRIB1,RASGRP2,RASGRP2,CDK8,CDK8,CDK9,SPRY1,SPRY2,STAM2,CNKSR1,CDKN1A,RAMP2,RAMP1,RAMP3,CDKN1B,CDKN1B,FSTL3,LOC102723532,LOC102724334,LOC102724334,STUB1,STAG1,NET1,TPTEP2-CSNK1E,RGS19,RGS19,CDKN2B,TCIRG1,NMUR1,LAMC3,IKZF1,RRAGB,TUBA1B,TUBB3,TUBB4A,TUBB4B,MYL12B,MYL12B,DLC1,MYL9,MYL9,RACK1,ST3GAL6,NDC80,RAPGEF3,YAP1,LYPLA1,CDC42EP2,VAV3,VAV3,TAB1,GPNMB,BAIAP2,BAIAP2,TACC3,FST,LINC02210-CRHR1,CARM1,NCOA2,KAT5,KAT5,LAMTOR5,ARPC1A,ARPC1A,CXCL13,CENPA,DRAP1,CENPC,CDC42EP3,SH2B2,MXD4,RBCK1,CENPE,MYL12A,MYL12A,CENPF,RGS14,RGS14,IGF2BP1,CAMKK2,CAMKK2,SPINT2,KHDRBS3,KHDRBS1,CXCR6,RRAGA,GNA13,GNB5,GNB5,RRH,ADCY1,ADCY1,CETP,NRG3,NRG3,CFL1,NUDC,PTGES3,STAG2,GRAP,AHCYL1,NCKAP1,NCKAP1,IQGAP2,OR5I1,ADCY2,ADCY2,CFTR,CYSLTR1,CCR9,CGA,WASF3,WASF3,NOXA1,FRS3,FRS3,FRS2,FRS2,PDE10A,CCL27,NMU,NPFFR2,PROKR1,GPR83,ADCY3,ADCY3,DHRS4,UTS2,RALBP1,OS9,TMED2,YWHAQ,METAP2,KIF2C,ADAP1,CHD1,WWP1,EEF1AKMT4-ECE2,WWP2,RAPGEF4,CHD3,CHD3,CHD4,CHD4,AKR1C4,ADCY5,ADCY5,PRDM4,CHEK1,CIT,CIT,KIF3A,KIF3A,ZWINT,CDC37,FSTL1,STRAP,STRAP,WIF1,ADCY6,ADCY6,FZD10,AKAP13,DUSP10,CHN1,CHN2,PMF1,GPR176,GPR45,PTGDR2,HRH3,TUBA3E,RDH13,CHRM1,CHRM2,ADCY7,ADCY7,PTH2,CHRM3,CHRM4,CHRM5,MGLL,TUBA3D,DTX2,DTX2,ADCY8,ADCY8,UCN3,CHUK,FMNL2,RHPN1,RHPN1,ADCY9,ADCY9,ARHGEF25,ARHGAP33,RASGRP4,ADCYAP1,ADCYAP1,ARAP2,ARAP1,ADCYAP1R1,ADCYAP1R1,THEM4,TAGAP,AP2M1,AP2M1,AP2S1,AP2S1,RLN3,PIK3AP1,OR52E2,OR52J3,OR51L1,OR51A7,OR51S1,OR51F2,OR52R1,OR4C46,OR4X2,OR4B1,OR52M1,OR52K2,OR5P2,OR5P3,OR8I2,OR2D3,OR2D2,OR52W1,OR56A4,OR56A1,CLPS,LRRK2,CLTA,CLTA,OR2AP1,OR10P1,CLTB,SDR9C7,OR10AD1,CLTC,CLTC,OR10A7,CMA1,H4-16,H4-16,FGD4,RXFP2,PSMB11,PSMB11,OR4K14,OR4L1,OR11H6,GPHB5,CCR1,LEO1,CCR3,CCR4,CCR5,CCR6,CCR7,CCR8,ACKR2,ADH1A,CMKLR1,NOXO1,LTB4R,OR4D2,CNGB1,CNGA1,OR7D4,OR7G1,OR1M1,ADH1C,PCP2,OR1I1,OR10H4,CNR1,CNR2,H3C14,H3C14,ADH4,OR2M5,OR2M3,OR2T12,OR14C36,OR2T34,OR2T10,OR2T4,OR2T11,ATP6V1G3,CNTN1,OR10J5,OR2B11,COL1A1,COL1A2,UHMK1,COL2A1,COL3A1,COL4A1,IQGAP3,ARHGEF19,H2BU1,H2BU1,OR10T2,OR6P1,OR10X1,OR10Z1,OR6K6,OR6N1,COL4A2,COL4A3,COL4A4,PROKR2,COL4A5,COL5A1,COL5A2,COL6A1,COL6A2,COL6A3,NMS,COL9A1,COL9A2,COL9A3,OR9A4,COL11A1,COL11A2,ACVR1C,ACVR1C,COL6A6,GRK7,RTP1,CORT,EVC2,EVC2,ADM,ADORA1,OR2Y1,TAAR9,TAAR1,ADORA2A,ADORA2A,OR9A2,OR2A14,OR6B1,OR2F2,ADORA2B,CPT1A,CPT1B,CRABP1,CRABP2,CREB1,CREB1,ATF2,ATF2,CREBBP,CREBBP,OR13C5,OR13C8,OR13C3,OR13C4,OR13F1,OR1L8,OR1N2,OR1N1,DGKK,CRH,AMER1,AMER1,CRHBP,CRHR1,CRHR2,CRK,CRK,CRKL,CRKL,AAMP,ADORA3,DYNLL2,PARP1,MIB2,MAPK14,MAPK14,FGFBP3,PSMA8,PSMA8,OR52I2,OR51E1,CSF2,CSF2RA,ARHGAP42,CSF2RB,OR10A5,OR2AG1,PRICKLE1,CSK,CSN2,CSNK1A1,CSNK1A1,RDH12,CSNK1E,CSNK1G2,CSNK2A1,CSNK2A2,NRG4,NRG4,ADRA1D,CSNK2B,PIK3R6,ADRA1B,NOTUM,WIPF2,WIPF2,KLC3,SPC24,ADRA1A,CDC42EP5,CTBP1,CTBP2,CTNNA1,CTNNB1,CTNNB1,ADRA2A,CTNND1,OR6B3,CTSD,ADRA2B,PLB1,SGO2,GPBAR1,SGO1,ADRA2C,CX3CR1,PAQR3,KLB,ADRB1,DAB2IP,SLC38A9,CYBA,CYBB,ADRB2,CYLD,AMOT,AMOTL1,ADRB3,ATP6V0E2,GRK2,GRK2,GRK3,RDH10,OR1Q1,ARHGAP36,AWAT2,CYP26A1,AP2A1,AP2A1,CD55,DGKA,DGKB,DGKG,DGKH,DGKQ,AP2A2,AP2A2,SPRED1,OR7D2,AP2B1,AP2B1,PLPPR5,AKR1C1,OXER1,DDX5,TLE5,GPHA2,RHOV,PTCRA,DIAPH1,DIAPH2,DLAT,DLD,DLG1,DLG2,DLG3,DLG4,DLG4,DNM1,DYNC1H1,DYNC1I1,DYNC1I2,DYNC1LI2,DNM2,DOCK1,DOCK3,DOCK3,DOK1,DRD1,DRD2,DRD3,DRD4,DRD5,JAG1,JAG1,ATN1,AGT,HBEGF,HBEGF,DTX1,DTX1,DUSP1,DUSP2,DUSP3,DUSP4,DUSP5,DUSP6,DUSP7,AGTR1,DUSP8,DUSP9,DVL1,DVL1,DVL2,DVL2,DVL3,DVL3,AGTR2,E2F1,E2F1,APLNR,E2F3,E2F3,E2F4,E2F5,GPR183,ECE1,ECT2,ABCA1,S1PR1,LPAR1,S1PR3,EDN1,EDN2,EDN3,EDNRA,EDNRB,PHC1,PHC1,PHC2,PHC2,AGO3,AGO4,EGF,EGF,EGFR,EGFR,EGR1,EGR1,SDR16C5,EGR2,EGR2,EGR3,EGR3,EGR4,OR56B4,ADCY4,ADCY4,EIF4B,EIF4E,EIF4EBP1,EIF4G1,ELF3,A2M,ELK1,ELK1,SPRED2,KCTD6,MUC20,ARHGAP27,ADGRE1,CTTN,KHDRBS2,EP300,EP300,EPAS1,EPO,EPO,EPOR,EPOR,EPS15,ERBB2,ERBB2,ERBB3,ERBB3,ERBB4,ERBB4,EREG,EREG,AKT1,AKT1,AKT2,AKT2,ESR1,ESR2,ETV4,EVC,EVC,MECOM,MECOM,EZH2,EZH2,F2,F2R,F2RL1,F2RL2,F3,ALDH1A1,FABP5,FABP6,FABP7,PTK2B,FASN,OR8U1,OR4C16,OR4C11,OR4S2,OR4C6,OR5D14,OR5L1,OR5D18,OR5AS1,OR8K5,OR5T2,OR8H1,OR8K3,OR8J1,OR5R1,OR5M3,OR5M8,OR5M11,OR5AR1,OR8B12,OR8G5,OR10G8,OR10G9,OR10S1,OR6T1,OR4D5,OR6Q1,OR9I1,OR9Q1,OR9Q2,OR1S2,OR1S1,OR10Q1,OR5B17,OR5B21,OR5A2,OR5A1,OR4D6,OR4D11,ALDH1A3,SKA1,FCER2,RASGEF1A,REEP3,OPN5,FGD2,KDM1B,GPC2,DAGLB,NAPEPLD,GPRC6A,GPC4,FER,FES,FGA,FGB,FGD1,FGF1,FGF2,FGF3,FGF4,FGF5,FGF6,FGF7,FGF8,FGF9,FGF10,FGFR1,FGFR3,GPC5,FGFR2,FGFR4,FGG,VEGFD,FKBP1A,FKBP1A,MRAS,RASA3,CNKSR2,DZIP1,FKBP4,FKBP5,ARHGEF15,NCBP2,MAPRE1,SNW1,SNW1,DKK1,DKK1,SCMH1,SCMH1,DAAM1,KDM1A,KDM1A,KDM4B,PHLPP2,HECW1,FOXO1,KDM4C,FOXO3,ARHGAP26,TNRC6B,TAB2,TAB2,CLASP2,PLXND1,FLNA,GGA3,METAP1,RGL1,RCOR1,KANK1,CYFIP1,CYFIP1,PSME4,PSME4,FLT1,FLT3,DTX4,DTX4,RHOBTB2,ARHGEF9,FLT3LG,PLCB1,PLCB1,ARC,ARC,PHLPP1,FLT4,FLT4,OTUD3,MCF2L,NUP160,WWC1,IQCE,RPGRIP1L,NEDD4L,NEDD4L,CLASP1,FN1,ARHGEF12,ARHGEF18,ARHGEF18,SRGAP2,NCSTN,NCSTN,FNTA,PIP5K1C,FRAT2,FNTB,ITGB3BP,ITGB3BP,GPR161,RHOQ,ATP1B4,HEY1,HEY1,ICMT,CBX6,CBX6,HEY2,HEY2,POFUT1,SUZ12,SUZ12,SCRIB,ARHGAP45,FOS,PIK3R5,PIK3R5,OR52A1,FOSB,RBFOX2,ATP6V0A2,LPAR3,FPR1,FPR2,FPR3,OPN3,MKRN1,NTSR2,PPP1R15A,FLRT3,FLRT2,FLRT1,ARHGAP8,ABCA4,ABCA4,FRK,ATP6V0D2,ATP6V1C2,MTOR,FSHB,CENPI,FSHR,NR5A2,ABL1,GAST,ACKR1,RICTOR,FYN,FYN,FZD2,FZD2,OR6C74,OR6C3,OR2T6,GAB1,GAB1,OR1L4,GABBR1,EPGN,GABRA1,H2BC1,H2BC1,COL24A1,OR52B2,GABRB1,COL6A5,GABRB2,OR4C3,OR4S1,GABRB3,GABRG2,GABRG3,NPB,ARHGAP30,UTS2B,TAB3,SHC2,SHC2,CBY1,RASGRP3,NGEF,BAMBI,GALR1,AHCTF1,GALNT3,NELFB,TAS2R39,TAS2R40,TAS2R41,TAS2R43,TAS2R31,TAS2R46,TAS2R30,TAS2R19,TAS2R20,TAS2R50,NSL1,WWTR1,SH2B1,ULK3,CLIP3,LRIG1,LRP10,DNM3,ARHGEF26,GPSM1,ABHD12,PYGO1,IFT172,RGS22,NPHP4,OR1C1,OR1A2,GAS1,OR2F1,OR2B6,OR1J4,GAS8,TIAM2,OR2M4,OR2L2,OR2K2,GATA3,FGF20,OR7A17,OR5L2,OR5K1,OR5H1,GCG,PTPN18,OR10J1,KAT2A,KAT2A,OR8G2P,OR8B8,OR8G1,OR10A3,HEYL,HEYL,OPN1MW,AGO1,LATS2,OR12D2,OR11A1,OR10H3,OR10G3,OR10H2,OR10H1,AATF,RGS17,RGS17,GDF2,OR8B2,GDI1,OR7E24,GDI2,OR7C2,OR7A5,OR7C1,GDNF,OR4F4,OR4F3,OR4E1,OR4D1,OR2W1,OR2V1,OR2T1,GFAP,OR2J2,OR2H1,OR1L3,OR1L1,GFRA1,OR1J2,GFRA2,GFRA3,AMH,B4GALT1,AMHR2,GHRH,GHRHR,GHSR,GHSR,GIP,GIPR,NBEA,CYFIP2,CYFIP2,USP21,FGF22,BEX3,NOX1,LAT,PELP1,PDE7B,DKK4,DKK4,DKK2,DKK2,INTU,AGO2,GPC3,HCAR1,OXGR1,C5AR2,ARHGEF16,ERLEC1,BMP10,TNRC6A,ANGPTL3,P2RY10,GLI1,GLI1,GLI2,GLI2,GLI3,GLP1R,GNA11,GNA11,GNA12,GNA15,GNA15,GNAI1,GNAI1,GNAI2,GNAI2,GNAI3,GNAI3,GNAL,GNAO1,GNAO1,GNAQ,GNAQ,GNAS,GNAS,GNAT1,GNAT1,GNAT2,GNAT2,GNAZ,GNAZ,GNB1,GNB1,GNB2,GNB2,GNB3,GNB3,GNG3,GNG3,GNG4,GNG4,GNG5,GNG5,GNG7,GNG7,GNG10,GNG10,GNG11,GNG11,GNGT1,GNGT1,GNGT2,GNGT2,GNRH1,GNRH2,GNRHR,SFN,GPC1,CCR10,OR51B5,OR10AG1,OR5J2,GPR4,XCR1,OR4C13,OR4C12,NPBWR1,OR52Z1,OR51V1,BCL9L,OR8D1,OR8D2,OR8B4,OR9G4,NPBWR2,OR10A4,CXCR3,OR6C6,PRLHR,KSR2,OR4N4,UTS2R,GPR15,NPW,ANGPT1,GPR17,GPR18,LAMA1,GPR20,OR2Z1,OR10H5,OR2L13,OR14A16,LPAR4,RSPO1,MCHR1,GPR25,GPR27,RTP5,DLL1,DLL1,GPER1,GPR31,GPR32,CYP4V2,GPR150,OR2V2,GPR35,GPR37,MLNR,SCAI,GPR39,OR13C9,OR13D1,FFAR1,FFAR3,FFAR2,GRK4,GRK5,GRK5,GRK6,GPS2,GRB2,GRB2,GRB7,GRB10,RAPGEF1,RAPGEF1,LAMTOR2,ABR,GRIN1,GRIN2B,GRIN2B,GRIN2D,NR3C1,ARHGAP35,GRM1,MYLIP,GRM2,RACGAP1,GRM3,GRM4,GRM5,GRM6,GRM7,GRM8,CXCL1,CXCL2,CXCL3,GRP,GRPR,GSK3A,GSK3B,GSK3B,GTF2A1,GTF2A2,GTF2F1,GTF2F2,GUCA1A,GUCA1B,ICOS,GUCY2F,GPSM2,EEF2K,EPN1,GPR132,PKN3,LRP12,RHOD,GUCY2D,SH3KBP1,GZMB,ANXA1,H2AC8,H2AC8,H2AC7,H2AC7,H2AX,H2AX,H2AZ1,H2AZ1,H2BC5,H2BC5,H2BC3,H2BC3,H3-3A,H3-3A,H3-3B,H3-3B,HCRT,HCRTR1,HCRTR2,HDAC1,HDAC1,HDAC2,HDAC2,NCKAP1L,NCKAP1L,ADGRE2,HGF,HGFAC,NRG1,NRG1,PIK3R4,TAX1BP3,HIF1A,HIF1A,NR4A1,FOXA1,HNRNPA1,HNRNPF,HNRNPH1,TAAR6,APC,APC,HPN,HRAS,HRAS,HRH1,HRH2,PRMT1,HES1,HES1,BIRC2,HSD17B1,BIRC3,XIAP,HSPB1,BIRC5,HSP90AA1,HSP90AA1,HSP90AB1,DNAJB1,HSPG2,H3C15,H3C15,APOA1,APOA1,HTR1A,HTR1B,HTR1D,HTR1E,HTR1F,HTR2A,HTR2B,HTR2C,APOA2,APOA2,HTR4,HTR5A,HTR6,HTR7,IAPP,APOB,APOB,GPIHBP1,TAS2R60,HCAR2,FFAR4,FFAR4,OR8D4,OR5F1,OR5AP2,OR52L1,OR2AG2,RXFP4,ID1,SPOPL,ID2,ID3,ID4,RSPO2,ZDHHC21,CYP26C1,OR52B6,APOC1,OR2AT4,OR10A2,OR6C2,OR6C4,OR8S1,OR6S1,OR6F1,OR2W3,OR2T8,OR2T3,OR10R2,OR2T29,RSPO4,APOC2,APOC2,RTP2,APOC3,APOC3,APOC4,OR6V1,OR2A12,OR2A1,GNAT3,GNAT3,APOD,QRFP,OR1J1,OR1B1,OR13H1,TUBB8,TUBB2B,IGF1,APOE,APOE,IGF1R,IGF2,SKA2,NUP43,APP,RBPJ,RBPJ,RGSL1,RGSL1,KLK3,IHH,IHH,FAS,IKBKB,IL1RAP,IL2,IL2RA,FASLG,IL2RB,IL2RG,IL3,IL3RA,IL5,IL5RA,IL6,IL6R,IL6ST,CXCL8,CXCR1,CXCR2,INCENP,INHBA,INHBA,INHBB,INHBB,CXCL10,INS,INPP5B,INSL3,INSR,IRAK1,IRAK1,IRS1,IRS1,AR,ITGA2,ITGA2B,ITGA3,ITGAV,ITGB1,ARAF,ITGB3,ITPR1,ITPR1,ITPR2,ITPR2,ITPR3,ITPR3,JAG2,JAG2,JAK1,JAK2,JAK2,JAK3,JUN,JUN,JUNB,JUND,JUP,ANOS1,AREG,KIF7,AGRN,USP17L2,CENPS,KDR,KDR,KEL,KIF2A,KIF5A,KIF5B,KISS1,KIT,KIT,KLK2,ARF6,KNG1,KLC1,KPNA2,KPNA2,KRAS,KRAS,RHOA,RHOA,NPSR1,OR56B1,RHOB,RHOB,CCL4L1,RGS9BP,RGS9BP,HES5,HES5,RHOC,RHOC,OR6B2,ARHGEF37,KTN1,LAMTOR4,OR52K1,OR52I1,OR51D1,OR52A5,OR51B6,OR51M1,OR51Q1,OR51I1,OR51I2,OR52D1,OR52H1,OR52N4,OR52N5,OR52N2,OR52E6,OR52E8,OR52E4,OR56A3,OR56A5,OR10A6,OR4X1,OR5D13,OR5D16,OR5W2,OR8H2,OR8H3,OR5T3,OR5T1,OR8K1,OR5M9,OR5M10,OR5M1,OR9G1,OR5AK2,OR5B2,OR5B12,OR5AN1,OR4D10,OR4D9,OR10V1,OR6X1,OR6M1,OR10G4,OR10G7,OR8B3,OR8A1,OR6C1,OR6C75,OR6C76,OR6C70,OR4N2,OR4K2,OR4K13,OR4K17,OR4N5,OR11G2,OR11H7,OR11H4,OR5AU1,OR4M2,OR4F6,OR4F15,LAMA2,OR7G2,OR7G3,OR7A10,LAMA3,RHOG,LAMA4,LAMA5,OR10K2,OR10K1,OR6Y1,OR6K3,OR11L1,OR2L8,OR2AK2,OR2L3,OR2M2,OR2T33,OR2M7,LAMB1,OR2G6,LAMB2,LAMB3,LAMC1,LAMC2,ARHGAP1,OR2A25,OR13J1,OR13C2,OR1L6,OR5C1,OR1K1,STMN1,ARHGAP4,OR2A5,LCK,LCK,ARHGAP5,LDLR,ARHGAP6,LEP,LEPR,LFNG,ARHGDIA,ARHGDIB,LHB,LHCGR,ARHGDIG,LIMK1,LIMK1,LIMK2,RHOH,SPRED3,OR2A7,CENPP,OR51H1,OR51T1,OR51A4,OR51A2,OR2T2,OR2T5,OR14I1,ARL2,OR5K2,LPL,OR2A42,OR2T27,OR2T35,OR4A47,OR5H14,OR5H15,OR5K3,OR5K4,OR6C65,OR6C68,LRP1,LRP2,LRP6,LRP6,LRP5,LRP5,LYL1,LYN,LYN,ARRB1,ARRB1,MAD2L1,SMAD1,SMAD2,SMAD2,SMAD3,SMAD3,SMAD4,SMAD4,ARRB2,ARRB2,SMAD5,SMAD6,SMAD7,SMAD7,SMAD9,MAG,MARK3,CCL3L3,MATK,MC1R,MC2R,MC3R,MC4R,MC5R,MCF2,MDK,MDM2,MEF2A,MEF2C,MEF2D,MEF2D,MEN1,MET,MFNG,KITLG,KITLG,CXCL9,ASCL1,ASCL1,MLN,MAP3K11,FOXO4,MMP2,MMP3,MMP3,MMP7,RGS21,RGS21,MMP9,MOV10,CCDC88C,OR4F21,OR5B3,OR9K2,OR4Q3,OR4M1,OR10J3,OR2W5,OR13G1,OR2B3,OR2J1,OR2J3,OR14J1,OR10C1,OR2A2,CITED1,ARHGEF35,MST1,MST1R,MTNR1A,MTNR1B,MYB,MYC,MYD88,MYD88,MYH9,MYH9,MYH10,MYH10,MYH11,MYH11,MYL6,MYL6,MYLK,MYLK,MYO7A,MYO9A,MYO9B,PPP1R12A,PPP1R12A,ATF1,ATF1,PPP1R12B,PPP1R12B,NAB1,NAB2,NAB2,HNRNPM,NCAM1,NCBP1,NCF2,NCF4,NCK1,NCK1,NEDD4,NEDD8,H2AB1,H2AB1,NEFL,NF1,NF2,NFATC1,NFKB1,NFKBIA,NGF,NGF,NGFR,NGFR,NMB,NMBR,NMT1,NOS3,NOTCH1,NOTCH1,NPY,NOTCH2,NOTCH2,NOTCH3,NOTCH3,NOTCH4,NOTCH4,ATP2A1,ATP2A2,NPY1R,NPY2R,NPY5R,ATP2A3,NRAS,NRAS,NRTN,YBX1,NTF3,NTF3,NTF4,NTF4,NTRK1,NTRK1,NTRK2,NTRK2,NTRK3,NTRK3,ROR1,ROR2,ROR2,NTS,NTSR1,NUP98,GPR143,OCRL,OMG,OPHN1,OPRD1,OPRK1,OPRL1,OPRM1,OR1D2,OR1F1,OR2C1,OR3A1,OR3A2,OXT,OXTR,P2RY1,P2RY2,P2RY4,P2RY6,P2RY11,P4HB,PEBP1,OR8U9,FURIN,PCSK6,PAFAH1B1,NOX3,COL5A3,SERPINE1,PAK1,PAK1,ATP6V0A4,PAK2,PAK2,PAK3,PAK3,ARHGEF4,ARHGEF3,RDH8,TAS2R3,TAS2R4,TAS2R16,TAS2R1,TAS2R9,TAS2R8,TAS2R7,TAS2R13,TAS2R10,TAS2R14,DHH,DHH,F11R,F11R,TRAT1,PARD6A,PARD6A,HEBP1,PBX1,CDON,PDE11A,SOST,SOST,TMED5,DERL2,PCK1,PRLH,MEMO1,GAL,IFT52,ABHD17B,APH1A,APH1A,RDH11,ZDHHC9,GOLGA7,IRAK4,DYNC1LI1,LEF1,LEF1,VRK3,PCSK5,RXFP3,GMIP,FAM13B,REEP2,SPTBN5,DACT1,PDE1A,PDE1C,UCHL5,PDE2A,ATP6V1D,WNT16,WNT16,PDE3A,PDE3B,PDE3B,PDE4A,PDE4B,AMOTL2,PRKAG2,PDE4C,PDE4D,PDE6A,PDE6A,EVL,PDE6D,PDE6G,PDE6G,PDE7A,PDE8A,NCKIPSD,NCKIPSD,CXXC5,PDE1B,PDGFA,SIRT6,PDGFB,ACKR4,PDGFRA,HDAC7,PDE6B,PDE6B,PDGFRB,TRIM33,PDHA1,ATP6V1H,PDHA2,PDHB,PDK1,PDK2,PDK3,PDK4,SUFU,SUFU,VPS29,PDPK1,PDPK1,NLK,CAB39,PDYN,GHRL,GHRL,WWOX,GNG13,GNG13,PENK,TUBA8,PF4,PFN1,PFN1,PFN2,PFN2,PGF,ATP6V1A,PGR,PHB,ATP6V1B1,ATP6V1B2,ATP6V0C,ATP6V1C1,PIK3C3,ATP6V1E1,PIK3CA,PIK3CA,PIK3CB,PIK3CB,PIK3CD,PIK3CD,PIK3CG,PIK3CG,PIK3R1,PIK3R1,PIK3R2,PIK3R2,PIN1,PIP4K2A,PLA2G4A,PLAT,ATP6V0B,PLCB2,PLCB2,PLCB3,PLCB3,PLCB4,PLCB4,PLCG1,PLCG1,SHC3,SHC3,PLCG2,ATP6V1G2,PLG,PLIN1,PLK1,ATP6V0A1,PLTP,MBD3,MBD3,BCO1,PRKAG3,S1PR5,PLXNB1,PMCH,PNOC,ATP6AP1,PML,P2RY13,GPR84,FGFRL1,RAB4B,PNLIP,TLR9,POLR2A,POLR2B,POLR2C,POLR2D,GNG2,GNG2,POLR2E,POLR2F,POLR2G,WNT4,WNT4,POLR2H,POLR2I,POLR2J,POLR2K,POLR2L,TAS2R5,POMC,NEURL1B,RHOF,POU2F1,APBB1IP,DLL4,DLL4,UGT1A3,PPARD,PPARG,PPARG,MED1,PPBP,PPEF1,IL17RD,ROPN1,ZRANB1,RNF111,RNF111,PPID,GATAD2A,GATAD2A,NDE1,ERCC6L,ESRP1,ARHGEF38,RETSAT,PLPPR1,RNF43,MKS1,SPDL1,PPM1A,PPP1CA,PPP1CA,PPP1CB,PPP1CB,LAMTOR1,PPP1CC,PPP1CC,ZWILCH,RNF31,IFT57,AVP,ARHGAP17,CDCA8,PPP2CA,PPP2CA,PPP2CB,PPP2CB,ARHGEF10L,CENPQ,PPP2R1A,PPP2R1A,PPP2R1B,PPP2R1B,AVPR1A,MOB1A,PPP2R5A,PPP2R5A,PPP2R5B,PPP2R5B,PPP2R5C,PPP2R5C,PPP2R5D,PPP2R5D,RHOT1,PPP2R5E,PPP2R5E,FBXW7,AVPR1B,PPP3CA,PPP3CA,PPP3CB,PPP3CB,PPP3CC,PPP3R1,PPP3R1,PPP5C,LGR4,PPY,AVPR2,NPY4R,H4C15,H4C15,STRADB,SMPD3,MAML3,MAML3,SOX6,PRR5,PRKAA1,STAP2,ZDHHC7,PRKAA2,PRKAB1,PRKAB2,PRKACA,PRKACA,PRKACB,PRKACB,PRKACG,PRKACG,ARHGEF40,PRKAG1,PRKAR1A,HHAT,VPS35,NUP133,PRKAR1B,PRKAR2A,PRKAR2A,IFT122,H2AJ,H2AJ,PRKAR2B,PRKCA,PRKCA,DEPDC1B,PRKCB,PRKCB,AXL,PRKCD,PRKCD,PRKCE,PRKCE,ADCY10,KDM3A,PRKCG,PRKCG,PAG1,PRKCH,PRKCH,CENPN,PRKCI,ARHGAP15,BRK1,BRK1,PKN1,PSENEN,PSENEN,PKN2,HDAC8,GABRQ,PRKCQ,PRKCQ,PRKCZ,PRKCZ,ERBIN,PRKG1,RCC2,PRKG2,PRKG2,APOM,MAPK1,MAPK1,MAPK3,MAPK3,MAPK4,MAPK6,GNG12,GNG12,MAPK7,MAPK7,MAPK8,MAPK8,MAPK11,MAPK11,MAPK13,MAPK13,PDGFC,MAP2K1,MAP2K2,MAP2K5,PRKX,PSPN,PARD3,PARD3,LTB4R2,CCL28,PSAP,CYP26B1,PSEN1,PSEN1,PSEN2,PSEN2,OR2S2,SUCNR1,PSMA1,PSMA1,PSMA2,PSMA2,PSMA3,PSMA3,PSMA4,PSMA4,PSMA5,PSMA5,PSMA6,PSMA6,PSMA7,PSMA7,PSMB1,PSMB1,PSMB2,PSMB2,PSMB3,PSMB3,PSMB4,PSMB4,NMUR2,SPPL2B,PSMB5,PSMB5,PMEPA1,PSMB6,PSMB6,PSMB7,PSMB7,OTUD7B,PSMB8,PSMB8,PYY,PSMB9,PSMB9,POGLUT1,PSMB10,PSMB10,CTNNBIP1,PSMC1,PSMC1,ACKR3,PSMC2,PSMC2,AKR1B10,PSMC3,PSMC3,PSMC4,PSMC4,PSMC5,PSMC5,PSMC6,PSMC6,PSMD1,PSMD1,PSMD2,PSMD2,KNL1,PSMD3,PSMD3,PSMD4,PSMD4,CYSLTR2,PSMD5,PSMD5,TRPC7,GOPC,LPAR5,NUP107,PSMD7,PSMD7,RGL3,PSMD8,PSMD8,RTN4,PSMD9,PSMD9,SMURF1,SMURF1,PSMD10,PSMD10,SALL4,SALL4,PSMD11,PSMD11,PSMD12,PSMD12,PSMD13,PSMD13,BAD,BAD,PSME1,PSME1,PSME2,PSME2,VANGL2,PTAFR,PTBP1,TAS2R38,PTCH1,PTCH1,PTEN,PTGDR,PTGER1,PTGER2,PTGER3,CBX8,CBX8,PTGER4,PTGFR,RHOJ,PTGIR,SPC25,ABHD6,PTH,PTHLH,PLEKHG5,PTH1R,GATAD2B,GATAD2B,PTH2R,PTK2,PTK2,NLN,KIDINS220,KIDINS220,MTA3,MTA3,ARHGAP31,RPTOR,SRGAP1,PTK6,CGN,CGN,MIB1,WDR35,TAOK1,ARHGAP20,PREX1,ARHGAP21,MRTFA,RANBP10,ARHGAP23,RDH14,GPAM,CHD8,TNRC6C,PTPN1,PTPN2,WDR19,SPTBN4,PTPN3,SCUBE2,TRIB3,PTPN6,PTPN7,PTPN11,PTPN11,PTPN12,PTPN12,PTPRA,PTPRJ,PTPRK,PTPRO,PTPRS,BAX,CXCL16,PXN,RHOU,ABHD17C,ARHGAP22,EPS15L1,RRAGD,RGL2,RAB4A,RAB6A,RAC1,RAC1,RAC2,RAC3,RAD21,RAF1,RAG1,RAG2,RALA,RALB,ACTA2,RALGDS,RALGDS,RANBP2,RANGAP1,RAP1A,RAP1A,RAP1B,RARA,RARB,RARG,RASA1,RASA2,RASGRF1,RASGRF2,RBBP4,RBBP4,RBBP5,RBBP7,RBBP7,RBL1,HRH4,GNB4,GNB4,KLHL12,RXFP1,LGR6,RBP1,RBP2,NPS,RBP3,CCND1,CCND1,RBP4,RCVRN,RCVRN,RDH5,BCL2,RELA,REST,REST,RET,BCL2L1,RFNG,TRIM27,RGR,RGS1,RGS1,RGS2,RGS3,RGS4,RGS4,ACTB,ACTB,RGS7,RGS7,RGS10,RGS10,RGS12,RGS12,RGS13,RGS13,RGS16,RGS16,RHEB,RHO,RHO,GRK1,GRK1,RIT2,RING1,RING1,RIT1,RLBP1,RLBP1,RLN2,TGIF2,RNF2,RNF2,SAV1,PROK2,BCL9,ROCK1,ROCK1,OPN1SW,RPE65,BCR,RPS6,RPS6KA1,RPS6KA2,RPS6KA3,RPS6KB1,RPS6KB2,BDKRB1,RPS27,RPS27A,RPS27A,RRAD,BDKRB2,RTKN,RTKN,CLIP1,RXRA,RXRB,RXRG,RYK,RYK,BDNF,BDNF,S100A8,S100A9,S100B,SAA1,SAG,SAG,MAPK12,MAPK12,SCD,SCT,SCTR,CCL1,CCL2,CCL3,CCL3L1,CCL4,CCL5,CCL7,CCL11,CCL13,CCL16,CCL17,CCL19,CCL20,CCL21,CCL22,CCL23,CCL25,CXCL6,CXCL11,CXCL5,XCL1,CX3CL1,SDC1,SDC2,SDC4,CXCL12,ELMO2,GPSM3,SEC13,SEL1L,SEL1L,GFRA4,CENPK,NPFFR1,RTP4,RRAGC,LIN7B,SFPQ,SFRP1,STRA6,MLST8,SFRP2,ABCG5,ABCG8,CXCR5,SOX17,ARHGAP9,GREM2,HHIP,RGS18,RGS18,ARAP3,WIPF3,WIPF3,SGK1,ITSN1,SH3GL1,SH3GL2,SH3GL2,SH3GL3,SH3GL3,ALDH8A1,SHB,SHB,SHC1,SHC1,SHH,SHH,PLPPR2,SMURF2,SMURF2,TNS3,BMI1,BMI1,P2RY12,KLC2,ST3GAL4,PORCN,PLEKHG2,ST3GAL3,RASAL3,CENPH,SKI,SKI,SKIL,SKIL,BMP2,SKP1,SKP1,REEP1,RTN4R,NCF1,H3C13,H3C13,BMPR1A,BMPR1B,BMPR2,SNAI2,SNAI2,SMARCA4,SMO,SMO,SMPD2,SMPD2,SNAI1,SNAI1,SOS1,SOS1,SOS2,SOX2,SOX3,SOX4,SOX9,SP1,SPARC,SPINT1,SPP1,SPTA1,SPTAN1,SPTB,SPTBN1,SPTBN2,SRC,SRC,SREBF1,SRF,SRF,SRMS,BRAF,BRAF,SRY,SST,SSTR1,SSTR2,SSTR3,SSTR4,SSTR5,STAT1,STAT1,STAT3,STAT3,STAT5A,STAT5A,STAT5B,STAT5B,STAT6,STK3,STK4,STK11,BRS3,STRN,XCL2,BTC,BTC,SYK,TAC1,TACR2,TAC3,ADAM17,ADAM17,TACR1,TACR3,MAP3K7,TBL1X,TBL1X,TBP,TBXA2R,TCF7,TCF7,TCF7L2,TCF7L2,TCF12,TCF12,BTK,BUB1,TEC,BUB1B,TEK,TERT,TFDP1,TFDP1,TFDP2,TFDP2,TFF1,TFF3,TGFA,TGFB1,TGFB1,TGFBR1,TGFBR1,TGFBR2,TGFBR2,TGIF1,THBS1,THBS2,THBS3,THBS4,TIA1,TIAL1,TIAM1,TIAM1,TJP1,TLE1,TLE1,TLE2,TLE2,TLE3,TLE3,TLE4,TLE4,TLN1,ACTG1,ACTG1,NR2E1,TNF,TNFAIP3,TNFRSF1A,TP53,TP53,TPH1,C3,TRAF1,TRAF2,TRAF6,TRAF6,C3AR1,TRH,TRHR,TRIO,TRPC3,TRPC6,H2AC19,H2AC19,TSC1,TSC2,TSHB,TSHR,C5,TTR,TUBA4A,TUBA3C,C5AR1,TUBB2A,OPN1MW2,TULP3,CCR2,TYK2,OR4F29,UBA52,UBA52,UBB,UBB,UBC,UBC,UBE2D1,UBE2D3,UCN,USP4,NR1H2,USF1,USF2,VAV1,VAV1,VAV2,VAV2,VCL,VCP,VEGFA,VEGFA,VEGFB,VEGFC,VGF,VIP,VIPR1,VIPR2,VWF,WAS,WAS,WIPF1,WIPF1,DAGLA,WNT1,WNT1,WNT2,WNT2,WNT3,WNT3,WNT5A,WNT5A,WNT6,WNT6,WNT7A,WNT7A,WNT7B,WNT7B,WNT8A,WNT8A,WNT8B,WNT8B,WNT10B,WNT10B,WNT11,WNT11,WNT2B,WNT2B,WNT9A,WNT9A,WNT9B,WNT9B,XK,XPO1,XPO1,FMNL1,FMNL1,YES1,YES1,YY1,YWHAB,YWHAB,YWHAE,YWHAG,YWHAH,YWHAZ,YWHAZ,ZNF217,LRP8,TUBA1A,CXCR4,FZD5,FZD5,MAPKAPK3,USP7,MIS12,CENPM,NUP37,REEP5,MAPKAP1,CENPO,OR13A1,OR5H6,OR52E1,OR5H2,OR4K5,OR2H2,OR51G1,OR11H2,OR51B4,OR51B2,OR4C5,KREMEN2,KREMEN2,OR51J1,OR52N1,OR5AL1,OR4F5,OR2A4,OR4K1,CDC73,CALCA,ARHGAP10,DYNC2H1,CENPU,CALCB,TBL1XR1,TBL1XR1,FZD3,FZD3,MYH14,MYH14,SEM1,SEM1,TTC21B,CAMKMT,PIP4K2C,ARHGEF5,TUBAL3,HDAC11,CALCR,NUP85,ADM2,PLPPR3,WLS,DSN1,ESRP2,PHC3,PHC3,CALM1,CALM1,CENPT,FUZ,PREX2,STAM,PDGFD,WNT10A,WNT10A,REEP4,TNKS2,SHOC2,ADAM12,CALM2,CALM2,PDHX,FOSL1,CUL5,ARHGAP39,FGF23,B9D2,CALM3,CALM3,EEPD1,DUSP16,TAS1R2,TAS1R1,KMT2D,IFT88,TUBB1,WNT5B,WNT5B,OR5AC2,OR11H1,OR4F17,GPR68,OR4K15,OR8J3,OR51G2,OR51E2,OR4P4,OR4C15,OR4A5,OR4A16,OR4A15,OR10W1,OR2AE1,OR4F16,CAMK4,CAMK4,OR6N2,OR6K2,OR2L5,OR2G3,OR2G2,OR2C3,CAMK2A,CAMK2A,NDEL1,CAMK2B,CAMK2B,CAB39L,DIAPH3,OR5V1,OR2B2,CAMK2D,CAMK2D,OR12D3,CAMK2G,CAMK2G,RNF146,SHARPIN,ABHD17A,SEH1L,KIF18A,NCOA3,NRIP1,USP9X,SMC1A,H3-4,H3-4,H4C9,H4C9,TRRAP,TRRAP,AXIN1,AXIN1,AXIN2,BRAP,FZD1,FZD1,FZD4,FZD4,FZD6,FZD6,FZD7,FZD7,FZD8,FZD8,FZD9,H2AC14,H2AC14,H2AC6,H2AC6,H2AC4,H2AC4,H2AC18,H2AC18,H2AC20,H2AC20,H2BC8,H2BC8,H2BC13,H2BC13,H2BC15,H2BC15,H2BC14,H2BC14,H2BC7,H2BC7,TCF7L1,TCF7L1,H2BC6,H2BC6,H2BC9,H2BC9,H2BC10,H2BC10,APH1B,APH1B,H2BC4,H2BC4,ARHGAP24,H2BC17,H2BC17,H2BC21,H2BC21,CASP2,H3C1,H3C1,H3C4,H3C4,H3C3,H3C3,H3C6,H3C6,H3C11,H3C11,NUF2,H3C8,H3C8,TAAR8,H3C12,H3C12,H3C10,H3C10,H3C2,H3C2,H4C1,H4C1,SOX7,RTP3,CASP3,H4C4,H4C4,H4C6,H4C6,H4C12,H4C12,H4C11,H4C11,H4C3,H4C3,H4C8,H4C8,H4C2,H4C2,H4C5,H4C5,H4C13,H4C13,H4C14,H4C14,ITCH,ITCH,TAS1R3,MAD1L1,OR1A1,OR1D5,OR1E1,BCO2,OR1E2,FGFBP2,OR1G1,OR3A3,HDAC10,PIP5K1A,PIP5K1B,PIP4K2B,KREMEN1,KREMEN1,OBSCN,SPOP,KBTBD7,CASP8,QRFPR,ZNRF3,SYDE2,PPP1R1B,PPP1R1B,CASP9,MAF1,MAF1,CAMKK1,CAMKK1,WDR83,CASP10,AKT1S1,RASAL1,NSMAF,NCK2,PROK1,MAML2,MAML2,SYVN1,EFCAB7,CUL3,MCHR2,CUL1,CUL1,CASR,TUBB6,KISS1R,KIF2B,ADGRE3,IRS4,CBX2,CBX2,GPR65,TUBA1C,OFD1,CILP,GALR3,RSPO3,SPPL2A,LINGO1,RGS5,RGS5,ARHGEF39,TNS4,ARHGAP19,PIK3R3,IKBKG,H2BC12,H2BC12,DGKZ,DGKE,DGKD,COL27A1,CBX4,CBX4,SYDE1,RGS8,RGS8,RHPN2,RHPN2,DOCK7,DISP2,DISP2,LGR5,MAPKAPK5,CDC14B,CDC14A,MADD,MKNK1,CAV1,CAV2,OR6A2,RGS20,RGS20,RUVBL1,RUVBL1,RDH16,RUNX1,NPFF,PDE8B,HSD17B6,RUNX3,PTCH2,AKR1C3,NCOA1,LAMTOR3,CBFB,NUMB,NUMB,SOCS1,DYNLL1,TNKS,IRS2,IRS2,CBL,PEA15,S1PR4,TRADD,SNX3,EED,EED,RIPK1,TNFSF10,RIPK2,FADD,RGS11,RGS11,RGS9,RGS9,DLK1,TNFRSF10D,TNFRSF10B,TNFRSF10A,ACTN2,GALR2,CCNK,FGF18,FGF17,FGF16,IQGAP1,NRP2,NRP1,SYNGAP1,CFLAR,HDAC3,HDAC3,HCAR3,KSR1,CCK,KAT2B,KAT2B,CDK5R1,CDK5R1,ALDH1A2,CCKAR,APLN,CCKBR,IER3,ARHGEF7,SPHK1,SQSTM1,TAX1BP1,CCNC,CCNC,FOXH1,WASF1,WASF1,CDK5R2,BTRC,BTRC,CCND3,H3C7,H3C7,H2BC11,H2BC11,USP13,WASL,WASL,WNT3A,WNT3A,CCNE1,ARHGAP11B,FGD3,ATP6V0E1,RHOT2,KLC4,KALRN,F2RL3,SOCS3,SOCS3,UCN2,OTULIN,CCRL2,TAAR5,CCNT1,UBE2M,ATP6V1E2,SH2D2A,ARTN,CCNT2,PRC1,STARD13,ASH2L,PYGO2,IL33,USP2,ACVR1B,USP8,FMNL3,MTMR4,MTA1,MTA1,LATS1,ATP6V0D1,SMC3,ARHGEF1,HGS,NEURL1,DEPDC7,DGKI,BOC,BOC,EBAG9,CENPL,LPAR2,IL1RL1,ARHGEF2,ZW10,BUB3,CHRDL1,SLC24A1,ACVR2A,ACVR2A,AURKB,MTA2,MTA2,LRAT,STRADA,NOG,DHRS3,RPS6KA5,MOB1B,MAPKAPK2,DNER,GPR37L1,REEP6,TAAR2,GPR55,S1PR2,ATP6V1F,ACVR2B,ACVR2B,CD19,SOCS6,TRIP10,GLP2R,CER1,KL,ARHGAP18,ZFYVE9,ZFYVE9,NMT2,ACVRL1,CD28,GRAP2,CD80,ARHGAP29,ARHGAP12,TJP2,CD86,OPN4,GNG8,GNG8,H2AZ2,H2AZ2,PPP1R14A,ARHGEF6,RASAL2,ROCK2,ROCK2,PSMF1,PSMF1,MAGED1,BAG4,NRG2,NRG2,ATP6V1G1,VPS26A,CCL4L2,BCAR1,BCAR1,GABBR2,SOX13,NCOR1,NCOR1,NCOR2,NCOR2,ABCG1,GUCA1C,RGS6,RGS6,GNA14,GNA14,ARHGEF10,SDC3,KDM4A,GREB1,RAPGEF2,ECE2,HDAC9,KNTC1,USP34,IFT140,ARHGAP32,STARD8,HDAC4,ADGRE5,ZFYVE16,DLGAP5,CKAP5,MAML1,MAML1,ARHGAP11A,ARHGEF11,ARHGEF17,CDK1,ELMO1,GAB2,PSMD6,PSMD6,RHOBTB1,SEPTIN7,PLPPR4,SRGAP3,CDC20,ARHGAP44,KIF14,P2RY14,ARHGAP25,CDC25C,USP15,FGF19,RBX1,RBX1,CDC42,CDC42,FGFBP1,RCE1,CDH1",Signal Transduction,3416

R-HSA-162585,PPIA,Uncoating of the HIV Virion,1

R-HSA-162587,"PDCD6IP,ELOA3D,RNF103-CHMP3,CDK7,CDK9,CDK9,NUP50,RCC1,NUP42,PSIP1,SUPT16H,CCR5,CHMP4B,NUP35,VPS37A,TAF1L,VPS37D,ELOA3,ERCC2,ERCC3,FEN1,NCBP2,NUP205,NUP210,NUP160,NEDD4L,NUP188,NUP62,XRCC6,NELFB,NELFB,CHMP2B,VPS4A,CHMP2A,CHMP4A,GTF2A1,GTF2A2,GTF2B,GTF2E1,GTF2E2,GTF2F1,GTF2F1,GTF2F2,GTF2F2,GTF2H1,GTF2H2,GTF2H3,GTF2H4,HMGA1,NUP43,KPNA1,LIG1,LIG4,GTF2H5,MNAT1,NCBP1,NMT1,NUP88,NUP98,FURIN,VPS28,ELOA2,UBAP1,NELFCD,NELFCD,CHMP5,VTA1,TAF9B,CHMP3,NUP54,POLR2A,POLR2A,POLR2B,POLR2B,POLR2C,POLR2C,POLR2D,POLR2D,POLR2E,POLR2E,POLR2F,POLR2F,POLR2G,POLR2G,POLR2H,POLR2H,POLR2I,POLR2I,POLR2J,POLR2J,POLR2K,POLR2K,POLR2L,POLR2L,TAF7L,PPIA,VPS37C,NDC1,NUP133,NUP107,RAN,RANBP1,RANBP2,RANGAP1,RPS27A,SEC13,SSRP1,SUPT4H1,SUPT4H1,SUPT5H,SUPT5H,TAF1,TAF2,TAF4,TAF4B,TAF5,TAF6,TAF7,TAF9,TAF10,TAF11,TAF12,TAF13,TBP,TCEA1,ELOC,ELOB,ELOA,TPR,TSG101,ELOA3B,UBA52,UBB,UBC,NELFA,NELFA,XPO1,XRCC4,XRCC5,CXCR4,NUP37,NELFE,NELFE,CHMP6,VPS37B,NUP85,NUP214,AAAS,TAF15,ELL,SEH1L,TAF3,RAE1,RNMT,RNGTT,CCNK,BANF1,MVB12B,CCNH,CCNT1,CCNT1,CCNT2,CTDP1,CTDP1,CHMP7,CD4,CHMP4C,MVB12A,NMT2,VPS4B,NUP155,NUP93,NUP58,POM121,NUP153",HIV Life Cycle,175

R-HSA-162588,"PDCD6IP,RNF103-CHMP3,CHMP4B,VPS37A,VPS37D,NEDD4L,CHMP2B,VPS4A,CHMP2A,CHMP4A,VPS28,UBAP1,CHMP5,VTA1,CHMP3,PPIA,VPS37C,RPS27A,TSG101,UBA52,UBB,UBC,CHMP6,VPS37B,MVB12B,CHMP7,CHMP4C,MVB12A,VPS4B",Budding and maturation of HIV virion,29

R-HSA-162589,PPIA,Reverse Transcription of HIV RNA,1

R-HSA-162592,"PSIP1,XRCC6,HMGA1,KPNA1,LIG4,PPIA,XRCC4,XRCC5,BANF1",Integration of provirus,9

R-HSA-162594,"PSIP1,CCR5,FEN1,XRCC6,HMGA1,KPNA1,LIG1,LIG4,PPIA,XRCC4,XRCC5,CXCR4,BANF1,CD4",Early Phase of HIV Life Cycle,14

R-HSA-162599,"PDCD6IP,ELOA3D,RNF103-CHMP3,CDK7,CDK9,CDK9,NUP50,RCC1,NUP42,SUPT16H,CHMP4B,NUP35,VPS37A,TAF1L,VPS37D,ELOA3,ERCC2,ERCC3,NCBP2,NUP205,NUP210,NUP160,NEDD4L,NUP188,NUP62,NELFB,NELFB,CHMP2B,VPS4A,CHMP2A,CHMP4A,GTF2A1,GTF2A2,GTF2B,GTF2E1,GTF2E2,GTF2F1,GTF2F1,GTF2F2,GTF2F2,GTF2H1,GTF2H2,GTF2H3,GTF2H4,NUP43,GTF2H5,MNAT1,NCBP1,NMT1,NUP88,NUP98,FURIN,VPS28,ELOA2,UBAP1,NELFCD,NELFCD,CHMP5,VTA1,TAF9B,CHMP3,NUP54,POLR2A,POLR2A,POLR2B,POLR2B,POLR2C,POLR2C,POLR2D,POLR2D,POLR2E,POLR2E,POLR2F,POLR2F,POLR2G,POLR2G,POLR2H,POLR2H,POLR2I,POLR2I,POLR2J,POLR2J,POLR2K,POLR2K,POLR2L,POLR2L,TAF7L,PPIA,VPS37C,NDC1,NUP133,NUP107,RAN,RANBP1,RANBP2,RANGAP1,RPS27A,SEC13,SSRP1,SUPT4H1,SUPT4H1,SUPT5H,SUPT5H,TAF1,TAF2,TAF4,TAF4B,TAF5,TAF6,TAF7,TAF9,TAF10,TAF11,TAF12,TAF13,TBP,TCEA1,ELOC,ELOB,ELOA,TPR,TSG101,ELOA3B,UBA52,UBB,UBC,NELFA,NELFA,XPO1,NUP37,NELFE,NELFE,CHMP6,VPS37B,NUP85,NUP214,AAAS,TAF15,ELL,SEH1L,TAF3,RAE1,RNMT,RNGTT,CCNK,MVB12B,CCNH,CCNT1,CCNT1,CCNT2,CTDP1,CTDP1,CHMP7,CHMP4C,MVB12A,NMT2,VPS4B,NUP155,NUP93,NUP58,POM121,NUP153",Late Phase of HIV Life Cycle,162

R-HSA-162658,"GORASP2,GOLGA2,GOLGA2,PLK1,PLK1,MAPK1,MAPK3,RAB1A,RAB1A,RAB2A,GORASP1,GORASP1,RAB1B,RAB1B,BLZF1,USO1,CCNB1,CCNB2,CDK1",Golgi Cisternae Pericentriolar Stack Reorganization,19

R-HSA-162699,"DPM3,DPM1,DPM2",Synthesis of dolichyl-phosphate mannose,3

R-HSA-162710,"PIGN,PIGW,PIGP,PIGA,PIGC,PIGF,PIGF,PIGH,PIGG,PIGX,PIGV,PIGZ,PIGO,PIGY,DPM2,PIGQ,PIGM,PIGL,PIGB",Synthesis of glycosylphosphatidylinositol (GPI),19

R-HSA-162791,"PIGK,PIGU,PIGT,PLAUR,PGAP1,GPAA1,PIGS",Attachment of GPI anchor to uPAR,7

R-HSA-162906,"PDCD6IP,ELOA3D,RNF103-CHMP3,AP1M2,PSME3,PSMD14,CDK7,CDK9,CDK9,NUP50,NUP50,RCC1,NUP42,NUP42,PSIP1,SUPT16H,AP2M1,AP1S1,AP2S1,PSMB11,CCR5,CHMP4B,NUP35,NUP35,AP1S3,VPS37A,TAF1L,PSMA8,VPS37D,AP2A1,AP2A2,AP1B1,ELOA3,AP2B1,AP1G1,DOCK2,ERCC2,ERCC3,FEN1,NCBP2,NUP205,NUP205,PSME4,NUP210,NUP210,NUP160,NUP160,NEDD4L,NUP188,NUP188,NUP62,NUP62,FYN,XRCC6,NELFB,NELFB,CHMP2B,VPS4A,CHMP2A,CHMP4A,SLC25A4,SLC25A5,SLC25A6,GTF2A1,GTF2A2,GTF2B,GTF2E1,GTF2E2,GTF2F1,GTF2F1,GTF2F2,GTF2F2,GTF2H1,GTF2H2,GTF2H3,GTF2H4,HCK,HLA-A,HMGA1,NUP43,NUP43,ARF1,KPNA1,KPNB1,KPNB1,LCK,LIG1,LIG4,GTF2H5,MNAT1,NCBP1,NMT1,NPM1,NPM1,NUP88,NUP88,NUP98,NUP98,FURIN,PAK2,VPS28,ELOA2,UBAP1,NELFCD,NELFCD,CHMP5,VTA1,ATP6V1H,TAF9B,CHMP3,NUP54,NUP54,POLR2A,POLR2A,POLR2B,POLR2B,POLR2C,POLR2C,POLR2D,POLR2D,POLR2E,POLR2E,POLR2F,POLR2F,POLR2G,POLR2G,POLR2H,POLR2H,POLR2I,POLR2I,POLR2J,POLR2J,POLR2K,POLR2K,POLR2L,POLR2L,TAF7L,PPIA,VPS37C,PACS1,NDC1,NDC1,NUP133,NUP133,B2M,PSMA1,PSMA2,PSMA3,PSMA4,PSMA5,PSMA6,PSMA7,PSMB1,PSMB2,PSMB3,PSMB4,PSMB5,PSMB6,PSMB7,PSMB8,PSMB9,PSMB10,PSMC1,PSMC2,PSMC3,PSMC4,PSMC5,PSMC6,PSMD1,PSMD2,PSMD3,PSMD4,PSMD5,NUP107,NUP107,PSMD7,PSMD8,PSMD9,PSMD10,PSMD11,PSMD12,PSMD13,PSME1,PSME2,RAC1,RAN,RANBP1,RANBP2,RANBP2,RANGAP1,APOBEC3G,RPS27A,SEC13,SEC13,SKP1,SSRP1,SUPT4H1,SUPT4H1,SUPT5H,SUPT5H,TAF1,TAF2,TAF4,TAF4B,TAF5,TAF6,TAF7,TAF9,TAF10,TAF11,TAF12,TAF13,TBP,TCEA1,ELOC,ELOC,ELOB,ELOB,ELOA,TPR,TPR,TSG101,ELOA3B,UBA52,UBB,UBC,NELFA,NELFA,XPO1,XRCC4,XRCC5,CXCR4,NUP37,NUP37,NELFE,NELFE,CHMP6,VPS37B,SEM1,NUP85,NUP85,NUP214,NUP214,CUL5,AAAS,AAAS,TAF15,ELL,SEH1L,SEH1L,TAF3,RAE1,RAE1,RNMT,RNGTT,CCNK,BANF1,AP1S2,AP1M1,BTRC,MVB12B,CCNH,CCNT1,CCNT1,CCNT2,CTDP1,CTDP1,CHMP7,CD247,CD4,CHMP4C,CD8B,MVB12A,NMT2,CD28,PSMF1,VPS4B,NUP155,NUP155,NUP93,NUP93,NUP58,NUP58,ELMO1,PSMD6,POM121,POM121,NUP153,NUP153,RBX1",HIV Infection,290

R-HSA-162909,"AP1M2,PSME3,PSMD14,CDK9,NUP50,NUP50,RCC1,NUP42,NUP42,PSIP1,AP2M1,AP1S1,AP2S1,PSMB11,NUP35,NUP35,AP1S3,PSMA8,AP2A1,AP2A2,AP1B1,AP2B1,AP1G1,DOCK2,NUP205,NUP205,PSME4,NUP210,NUP210,NUP160,NUP160,NUP188,NUP188,NUP62,NUP62,FYN,SLC25A4,SLC25A5,SLC25A6,HCK,HLA-A,HMGA1,NUP43,NUP43,ARF1,KPNA1,KPNB1,KPNB1,LCK,NPM1,NPM1,NUP88,NUP88,NUP98,NUP98,PAK2,ATP6V1H,NUP54,NUP54,PPIA,PACS1,NDC1,NDC1,NUP133,NUP133,B2M,PSMA1,PSMA2,PSMA3,PSMA4,PSMA5,PSMA6,PSMA7,PSMB1,PSMB2,PSMB3,PSMB4,PSMB5,PSMB6,PSMB7,PSMB8,PSMB9,PSMB10,PSMC1,PSMC2,PSMC3,PSMC4,PSMC5,PSMC6,PSMD1,PSMD2,PSMD3,PSMD4,PSMD5,NUP107,NUP107,PSMD7,PSMD8,PSMD9,PSMD10,PSMD11,PSMD12,PSMD13,PSME1,PSME2,RAC1,RAN,RANBP1,RANBP2,RANBP2,RANGAP1,APOBEC3G,RPS27A,SEC13,SEC13,SKP1,ELOC,ELOB,TPR,TPR,UBA52,UBB,UBC,XPO1,NUP37,NUP37,SEM1,NUP85,NUP85,NUP214,NUP214,CUL5,AAAS,AAAS,SEH1L,SEH1L,RAE1,RAE1,BANF1,AP1S2,AP1M1,BTRC,CCNT1,CD247,CD4,CD8B,CD28,PSMF1,NUP155,NUP155,NUP93,NUP93,NUP58,NUP58,ELMO1,PSMD6,POM121,POM121,NUP153,NUP153,RBX1",Host Interactions of HIV factors,161

R-HSA-1630316,"ABCC5,GPC6,GPC6,UST,B3GNT3,ST3GAL6,CSPG5,B3GNT2,HPSE,SLC26A1,LYVE1,B4GAT1,SLC35D2,KERA,CHP1,B4GALT7,CHST14,B3GALT6,B3GAT2,HGSNAT,VCAN,NCAN,CSPG4,DCN,CHST13,ACAN,SLC26A2,EXT1,EXT2,GPC2,GPC2,HS3ST5,GPC4,GPC4,GPC5,GPC5,CHSY1,FMOD,CHST5,GALNS,GLCE,B3GAT3,HS6ST3,B4GALT1,B3GAT1,GPC3,GPC3,GLB1,GNS,GPC1,GPC1,GUSB,DSE,HAS1,HAS2,HAS3,HEXA,HEXB,HMMR,HSPG2,HSPG2,NDST1,HYAL1,CHSY3,IDS,IDUA,SLC35B2,AGRN,AGRN,LUM,ARSB,CHST6,NAGLU,OMD,OGN,CHST11,SLC35B3,CHST15,CHPF2,CSGALNACT2,PRELP,CHST12,STAB2,CSGALNACT1,CHST7,CEMIP,HPSE2,BGN,SDC1,SDC1,BCAN,SDC2,SDC2,SDC4,SDC4,XYLT1,XYLT2,SGSH,NDST4,HS3ST6,ST3GAL1,ST3GAL2,ST3GAL4,ST3GAL3,SLC9A1,B3GNT4,GLB1L,CHPF,CHST9,HYAL3,NDST2,CHST1,HYAL2,B4GALT4,B4GALT3,B4GALT2,HS6ST2,PAPSS2,PAPSS1,DSEL,B3GNT7,B4GALT6,B4GALT5,NDST3,HS6ST1,CHST2,CHST3,CD44,HS2ST1,SDC3,SDC3,HS3ST4,HS3ST3B1,HS3ST3A1,HS3ST2,HS3ST1",Glycosaminoglycan metabolism,136

R-HSA-163125,"PIGK,MSLN,CD52,CEACAM5,CEACAM7,PRSS21,LYPD1,NRN1L,PIGU,LYPD6B,CD109,CPM,LYPD2,OTOA,RTN4RL1,LYPD4,CNTN4,RAET1L,MDGA2,SPACA4,FCGR3B,NTNG1,FOLR2,PIGN,PRND,ALPI,ALPL,ALPG,NEGR1,MDGA1,LYPD3,GP2,GPLD1,PIGW,LYPD5,GPIHBP1,PLET1,RTN4RL2,RAET1G,IZUMO1R,LSAMP,LY6E,LY6H,ART3,ART4,MELTF,OPCML,SPRN,CNTN3,NTM,PIGP,NRN1,PIGT,PIGA,PIGC,PIGF,PIGF,PIGH,PLAUR,CNTN5,DPM3,LY6K,PIGG,PIGX,VNN3,PIGV,LY6G6D,LYPD8,BST1,TECTB,TECTA,THY1,XPNPEP2,PSCA,PGAP1,PIGZ,ULBP2,LY6G6C,TEX101,RECK,NTNG2,PIGO,PIGY,LY6D,GPAA1,DPM1,DPM2,VNN2,VNN1,PIGQ,PIGM,PIGS,PIGL,PIGB",Post-translational modification: synthesis of GPI-anchored proteins,94

R-HSA-163200,"LRPPRC,TRAP1,ATP5PD,ATP5MG,UQCR11,COX20,NDUFA11,COX4I1,COX5B,COX6A1,COX6B1,COX6C,COX7B,COX7C,COX8A,COX11,NDUFAF6,PM20D1,CYC1,ETFA,ETFB,ETFDH,NDUFAF3,UQCRQ,DMAC2L,COX18,ACAD9,NDUFAF4,UQCR10,NDUFS7,ATP6,ATP8,COX1,COX2,COX3,CYTB,ND1,ND2,ND3,ND4,ND5,ND6,NDUFA1,NDUFA2,NDUFA3,NDUFA4,NDUFA5,NDUFA6,NDUFA7,NDUFA8,NDUFA9,NDUFA10,NDUFAB1,NDUFB1,NDUFB2,NDUFB3,NDUFB4,NDUFB5,NDUFB6,NDUFB7,NDUFB8,NDUFB9,NDUFB10,NDUFC1,NDUFC2,NDUFS1,NDUFS2,NDUFS3,NDUFV1,NDUFS4,NDUFS5,NDUFS6,NDUFS8,NDUFV2,NDUFV3,ATP5F1A,ATP5F1B,ATP5F1C,NDUFA13,NDUFAF1,TACO1,COX16,ECSIT,ATP5F1D,TIMMDC1,ATP5F1E,ATP5PB,ATP5MC1,ATP5MC2,ATP5MC3,ATP5ME,ATP5PF,ATP5PO,CYCS,NDUFB11,NDUFAF7,TMEM126B,NDUFA12,SCO1,SDHA,SDHB,SDHC,SDHD,SURF1,UCP1,UCP2,UCP3,UQCRB,UQCRC1,UQCRC2,UQCRFS1,UQCRH,NDUFAF5,COQ10B,NUBPL,COX14,SLC25A14,COX7A2L,NDUFAF2,COQ10A,COX5A,SLC25A27,ATP5MF,SCO2","Respiratory electron transport, ATP synthesis by chemiosmotic coupling, and heat production by uncoupling proteins.",124

R-HSA-163210,"ATP5PD,ATP5MG,DMAC2L,ATP6,ATP8,ATP5F1A,ATP5F1B,ATP5F1C,ATP5F1D,ATP5F1E,ATP5PB,ATP5MC1,ATP5MC2,ATP5MC3,ATP5ME,ATP5PF,ATP5PO,ATP5MF",Formation of ATP by chemiosmotic coupling,18

R-HSA-163282,"POLRMT,TFB2M,TFAM",Mitochondrial transcription initiation,3

R-HSA-1632852,"HDAC6,HDAC6,TOMM6,RNF103-CHMP3,PLIN3,RRAGB,TUBA1B,TUBA1B,TUBB3,TUBB3,TUBB4A,TUBB4A,TUBB4B,TUBB4B,TOMM40,ATG7,ATG7,LAMTOR5,RRAGA,CETN1,CETN1,CFTR,CFTR,WDR45,TUBA3E,TUBA3E,PARK7,PARK7,GABARAP,GABARAPL2,TUBA3D,TUBA3D,ATG4A,PLIN2,CHMP4B,FUNDC1,DYNLL2,DYNLL2,CSNK2A1,CSNK2A2,CSNK2B,SLC38A9,DYNC1H1,DYNC1H1,DYNC1I1,DYNC1I1,DYNC1I2,DYNC1I2,DYNC1LI2,DYNC1LI2,PGAM5,ARL13B,ARL13B,EPAS1,ATG14,ATG4B,GABARAPL1,MTOR,CHMP2B,WIPI2,CHMP2A,ATG9B,LAMTOR2,CHMP4A,PIK3R4,HSF1,HSPA8,HSP90AA1,TUBB8,TUBB8,TUBB2B,TUBB2B,TMEM189-UBE2V1,TMEM189-UBE2V1,LAMTOR4,TOMM5,NBR1,NBR1,MAP1LC3C,ATM,PRKN,MTERF3,DYNC1LI1,DYNC1LI1,PCNT,PCNT,PRKAG2,PRKAG2,CHMP3,TUBA8,TUBA8,PIK3C3,PRKAG3,PRKAG3,TOMM7,LAMTOR1,ATG16L1,ATG16L1,WIPI1,PRKAA1,AMBRA1,PRKAA2,PRKAA2,PRKAB1,PRKAB1,PRKAB2,PRKAB2,MFN1,PRKAG1,PRKAG1,WDR45B,TOMM22,RPTOR,PEX5,RRAGD,RHEB,ATG101,RPS27A,RPS27A,RRAGC,MLST8,MTMR14,ATG3,PINK1,SRC,TSC1,TSC2,TUBA4A,TUBA4A,TUBA3C,TUBA3C,TUBB2A,TUBB2A,UBA52,UBA52,UBB,UBB,UBC,UBC,UBE2N,UBE2N,UBE2V1,UBE2V1,UVRAG,VCP,VDAC1,VIM,TUBA1A,TUBA1A,ATG9A,CHMP6,TUBAL3,TUBAL3,IFT88,IFT88,TUBB1,TUBB1,MAP1LC3B,ATG10,ATG10,ULK1,MAP1LC3A,TUBB6,TUBB6,USP30,TUBA1C,TUBA1C,ATG4C,ATG4D,LAMTOR3,DYNLL1,DYNLL1,BECN1,SQSTM1,MTMR3,ATG12,ATG12,CHMP7,CHMP4C,ATG5,ATG5,ATG13,TOMM20,RB1CC1,TOMM70,MFN2",Macroautophagy,186

R-HSA-163316,MTERF1,Mitochondrial transcription termination,1

R-HSA-163358,"MLXIPL,MLXIPL,PFKFB1,PRKACA,PRKACB,PRKACG",PKA-mediated phosphorylation of key metabolic factors,6

R-HSA-163359,"ADCY1,ADCY2,ADCY3,ADCY5,ADCY6,ADCY7,ADCY8,ADCY9,ADCY4,GCG,GNAS,GNB1,GNB2,GNB3,GNG3,GNG4,GNG5,GNG7,GNG10,GNG11,GNG13,GNG2,PRKACA,PRKACB,PRKACG,PRKAR1A,PRKAR1B,PRKAR2A,PRKAR2B,GNG12,CYSLTR2,GNB4,GNG8",Glucagon signaling in metabolic regulation,33

R-HSA-163560,"PLIN3,MGLL,PNPLA5,FABP4,FABP4,FABP1,FABP2,FABP3,FABP5,FABP6,FABP7,GPD2,LIPE,ABHD5,PLIN1,PPP1CA,PPP1CB,PPP1CC,PRKACA,PRKACB,PRKACG,FABP9,FABP12,PNPLA4,CAV1",Triglyceride catabolism,25

R-HSA-163615,"ADCY1,ADCY2,ADCY3,ADCY5,ADCY6,ADCY7,ADCY8,ADCY9,ADCY4,NBEA,PRKACA,PRKACB,PRKACG,PRKAR1A,PRKAR1B,PRKAR2A,PRKAR2A,PRKAR2B,CALM1,CALM2,CALM3",PKA activation,21

R-HSA-163680,"MLXIPL,ADIPOR1,ADIPOR1,PRKAG2,PRKAG2,PRKAA2,PRKAA2,PRKAB2,PRKAB2,STK11,ADIPOR2,ADIPOR2,ADIPOQ,ADIPOQ",AMPK inhibits chREBP transcriptional activation activity,14

R-HSA-163685,"RAPGEF3,AGPAT1,GNB5,GNB5,ADCY1,AHCYL1,ADCY2,ADCY3,RAPGEF4,ADCY5,ADCY6,ADCY7,CHRM3,ADCY8,ADCY9,ADRA2A,ADRA2C,ADCY4,ACSL3,ACSL4,FASN,PLCB1,PLCB1,KCNG2,GCG,GCG,GLP1R,GLP1R,GNA11,GNA11,GNA15,GNA15,GNAI1,GNAI1,GNAI2,GNAI2,GNAQ,GNAQ,GNAS,GNAS,GNB1,GNB1,GNB2,GNB2,GNB3,GNB3,GNG3,GNG3,GNG4,GNG4,GNG5,GNG5,GNG7,GNG7,GNG10,GNG10,GNG11,GNG11,GNGT1,GNGT1,GNGT2,GNGT2,FFAR1,ACACA,ACACB,INS,ITPR1,ITPR1,ITPR2,ITPR2,ITPR3,ITPR3,KCNB1,KCNC2,KCNJ11,KCNS3,MARCKS,ACLY,MLXIPL,MLXIPL,ADIPOR1,ADIPOR1,PRKAG2,PRKAG2,GNG13,GNG13,PFKFB1,PKLR,PLCB2,PLCB2,PLCB3,PLCB3,GNG2,GNG2,PPP2CA,PPP2CB,PPP2R1A,PPP2R1B,PPP2R5D,PRKAA2,PRKAA2,PRKAB2,PRKAB2,PRKACA,PRKACA,PRKACB,PRKACB,PRKACG,PRKACG,PRKAR1A,PRKAR1A,PRKAR1B,PRKAR1B,PRKAR2A,PRKAR2A,PRKAR2B,PRKAR2B,PRKCA,GNG12,GNG12,CYSLTR2,RAP1A,GNB4,GNB4,SLC2A1,SLC2A2,SNAP25,STK11,STX1A,STXBP1,ABCC8,VAMP2,SYT5,TALDO1,MLX,MLX,TKT,CACNA1A,CACNA1C,CACNA1C,CACNA1D,CACNA1D,CACNA1E,CACNB2,CACNB2,CACNB3,CACNB3,ADIPOR2,ADIPOR2,IQGAP1,CACNA2D2,CACNA2D2,ADIPOQ,ADIPOQ,GNG8,GNG8,CD36,AKAP5,GNA14,GNA14",Integration of energy metabolism,160

R-HSA-163754,"TALDO1,TKT",Insulin effects increased synthesis of Xylulose-5-Phosphate,2

R-HSA-163765,"AGPAT1,FASN,ACACA,ACACB,ACLY,MLXIPL,MLXIPL,PKLR,MLX,MLX",ChREBP activates metabolic gene expression,10

R-HSA-163767,"MLXIPL,PFKFB1,PPP2CA,PPP2CB,PPP2R1A,PPP2R1B,PPP2R5D",PP2A-mediated dephosphorylation of key metabolic factors,7

R-HSA-1638074,"B3GNT3,ST3GAL6,B3GNT2,B4GAT1,SLC35D2,KERA,ACAN,FMOD,CHST5,GALNS,B4GALT1,GLB1,GNS,HEXA,HEXB,LUM,CHST6,OMD,OGN,PRELP,ST3GAL1,ST3GAL2,ST3GAL4,ST3GAL3,B3GNT4,GLB1L,CHST1,B4GALT4,B4GALT3,B4GALT2,B3GNT7,B4GALT6,B4GALT5,CHST2",Keratan sulfate/keratin metabolism,34

R-HSA-1638091,"GPC6,GPC6,CSPG5,HPSE,SLC35D2,B4GALT7,B3GALT6,B3GAT2,HGSNAT,VCAN,NCAN,CSPG4,DCN,EXT1,EXT2,GPC2,GPC2,HS3ST5,GPC4,GPC4,GPC5,GPC5,GLCE,B3GAT3,HS6ST3,B3GAT1,GPC3,GPC3,GLB1,GPC1,GPC1,GUSB,HSPG2,HSPG2,NDST1,IDS,IDUA,AGRN,AGRN,NAGLU,HPSE2,BGN,SDC1,SDC1,BCAN,SDC2,SDC2,SDC4,SDC4,XYLT1,XYLT2,SGSH,NDST4,HS3ST6,GLB1L,NDST2,HS6ST2,NDST3,HS6ST1,HS2ST1,SDC3,SDC3,HS3ST4,HS3ST3B1,HS3ST3A1,HS3ST2,HS3ST1",Heparan sulfate/heparin (HS-GAG) metabolism,67

R-HSA-163841,"DNAJC24,ARSK,DHPS,DPH1,DPH2,EEF2,EEF2,EIF5A,F2,F7,F8,F9,F10,ARSG,ICMT,SUMF2,GAS6,GAS6,GGCX,GGCX,SUMF1,DPH3,ARSI,ARSH,ARSA,ARSB,STS,ARSD,ARSL,ARSF,FURIN,DPH5,PROC,PROS1,EIF5A2,BGLAP,FN3K,ARSJ,FN3KRP,DOHH,TPST2,TPST1,PROZ,DPH6,DPH7","Gamma carboxylation, hypusine formation and arylsulfatase activation",45

R-HSA-1640170,"AKT3,TEN1,SMC4,SMC4,CENPS-CORT,RNF103-CHMP3,PMF1-BGLAP,RAD50,RAD50,KIF20A,ACTR1A,OPTN,AKAP9,CDK2,CDK2,CDK4,CDK4,PSME3,CDK6,PSMD14,CDK7,CDKN1A,CDKN1B,LOC102724334,LOC102724334,STAG1,STAG1,CDKN1C,TPTEP2-CSNK1E,CDKN2A,CDKN2B,CDKN2C,CDKN2D,TUBA1B,TUBB3,TUBB4A,TUBB4B,SYCP2,ANAPC10,NDC80,TUBGCP3,KAT5,DCTN2,CENPA,SMC2,SMC2,CENPC,CENPE,CENPF,CETN2,POLD3,NUDC,PLK4,STAG3,STAG2,STAG2,NUP50,ARPP19,ARPP19,NEK6,SDCCAG8,TUBGCP2,RUVBL2,DBF4,YWHAQ,KIF2C,RCC1,CNTRL,UBE2C,TOPBP1,DIDO1,NUP42,CHEK1,CHEK1,FGFR1OP,ZWINT,DMC1,CEP250,CHEK2,PMF1,DCTN3,TUBA3E,CDCA5,OIP5,TUBA3D,TUBGCP5,ESCO1,HAUS1,RMI2,CKS1B,ANAPC16,NEDD1,H4-16,H4-16,PSMB11,H3C14,H3C14,H2BU1,H2BU1,CHMP4B,NUP35,NUP35,LIN54,LIN54,NEK7,DYNLL2,PSMA8,RAD9B,CSNK1D,CSNK1E,CSNK2A1,CSNK2A2,CSNK2B,SPC24,SGO2,SGO1,ESCO2,DAXX,DCTN1,RNF168,DHFR,DKC1,DNA2,DYNC1H1,DYNC1I1,DYNC1I2,DYNC1LI2,DYRK1A,E2F1,E2F1,E2F2,E2F3,E2F4,E2F4,E2F5,E2F5,E2F6,CC2D1B,EMD,CENPX,ENSA,ENSA,TUBB,EP300,AKT1,AKT2,SKA1,LEMD2,FEN1,PHLDA1,CEP164,MAPRE1,SIRT2,TPX2,NINL,CEP131,CEP152,PDS5B,FOXM1,WAPL,CLASP2,PHF8,ANKLE2,NUP205,NUP205,LPIN1,FBXL7,PSME4,SYNE2,NUP210,ANKRD28,PDS5A,NUP160,NUP160,FBXW11,NCAPD3,CLASP1,SYNE1,SUN1,HAUS5,MAU2,NCAPH,NCAPH,CTDNEP1,ITGB3BP,NUP188,NUP188,LEMD3,ORC6,ORC6,ORC3,ORC3,SPO11,NUP62,NUP62,POLA2,CDC26,CENPI,ABL1,H2BC1,H2BC1,CNEP1R1,SYCE2,SUN2,NIPBL,ANAPC15,AHCTF1,AHCTF1,POT1,POT1,NSL1,ZNF385A,CHMP2B,GORASP2,FBXO5,TINF2,TINF2,MLH3,SMC1B,TUBG2,VPS4A,TUBGCP4,CHMP2A,UBE2S,EML4,GOLGA2,GOLGA2,SFN,PPP2R3B,LIN9,LIN9,CHMP4A,BABAM1,GSK3B,NCAPH2,ANAPC2,PSMC3IP,NME7,RPA4,ANAPC4,H2AC8,H2AC8,H2AC7,H2AC7,H2AX,H2AX,H2AZ1,H2AZ1,H2BC5,H2BC5,H2BC3,H2BC3,H3-3A,H3-3A,H3-3B,H3-3B,HDAC1,HDAC1,HMMR,HSPA2,BIRC5,HSP90AA1,HSP90AB1,H3C15,H3C15,HUS1,TUBB8,TUBB2B,SKA2,NUP43,NUP43,INCENP,JAK2,CENPS,KIF2A,KPNB1,KPNB1,TNPO1,CENPW,KMT5A,LBR,LIG1,LMNA,LMNA,LMNB1,LMNB1,CENPP,LYN,MAD2L1,MAX,MCM2,MCM2,MCM3,MCM3,MCM4,MCM4,MCM5,MCM5,MCM6,MCM6,MCM7,MCM7,MDM2,MDM4,RAB8A,MLH1,MNAT1,MRE11,MRE11,MZT1,MSH4,MSH5,MYBL2,MYBL2,MYC,PPP1R12A,PPP1R12B,NBN,NBN,ATM,ATM,H2AB1,H2AB1,NEK2,NPM1,NUMA1,NUP88,NUP98,NUP98,ODF2,ORC1,ORC1,ORC2,ORC2,ORC4,ORC4,ORC5,ORC5,PAFAH1B1,SYCP3,GMNN,PCM1,PCNA,DYNC1LI1,PCNT,PHF20,FZR1,PPME1,ANAPC5,ANAPC7,LCMT1,GTSE1,ANAPC11,PIAS4,CHMP3,GINS2,GINS2,UIMC1,RTEL1,RSF1,TUBA8,NUP54,NUP54,PLK1,PLK1,MIS18A,POLE3,POLA1,POLD1,POLD2,POLE,POLE2,POLR2A,POLR2B,POLR2C,POLR2D,POLR2E,POLR2F,POLR2G,POLR2H,POLR2I,TERF2IP,TERF2IP,POLR2J,POLR2K,POLR2L,GAR1,ATR,ATR,ATRX,HAUS6,NDE1,ERCC6L,NCAPG2,SPDL1,CHTF8,HAUS4,PPP1CB,PPP1CC,ZWILCH,CEP192,WRAP53,HAUS2,CDCA8,PPP2CA,PPP2CA,PPP2CB,PPP2CB,SHQ1,CENPQ,PPP2R1A,PPP2R1A,PPP2R1B,PPP2R1B,PPP2R2A,PPP2R5A,PPP2R5B,PPP2R5C,PPP2R5D,PPP2R5E,PPP6R3,MIS18BP1,HJURP,PPP6C,MCM10,H4C15,H4C15,NOP10,HAUS7,PRIM1,PRIM2,NHP2,PRKACA,NDC1,NDC1,CEP72,NUP133,NUP133,CDK5RAP2,H2AJ,H2AJ,PRKAR2B,PRKCA,PRKCB,CENPJ,CENPN,PPP2R2D,HDAC8,RCC2,MAPK1,MAPK3,LIN37,LIN37,TEX12,POLE4,PSMA1,PSMA2,PSMA3,PSMA4,PSMA5,PSMA6,PSMA7,PSMB1,PSMB2,PSMB3,PSMB4,PSMB5,PSMB6,PSMB7,PSMB8,PRDM9,PSMB9,PSMB10,PSMC1,PSMC2,PSMC3,PSMC4,PSMC5,PSMC6,PCBP4,PSMD1,PSMD2,KNL1,PSMD3,PSMD4,PSMD5,NUP107,NUP107,PSMD7,PSMD8,PSMD9,PSMD10,PSMD11,PSMD12,PSMD13,PSME1,PSME2,SPC25,PTK6,TAOK1,POLD4,BARD1,RAD1,RAB1A,RAB1A,RAB2A,RAD9A,RAD17,RAD21,RAD21,RAD51,RAD51C,RAN,RAN,RANBP2,RANGAP1,RB1,RB1,RBBP4,RBBP4,RBBP7,RBBP8,RBBP8,RBL1,RBL1,RBL2,RBL2,CCND1,CCND1,RFC1,RFC2,RFC3,RFC4,RFC5,RPA1,RPA1,RPA2,RPA2,RPA3,RPA3,RPS27,RPS27A,RRM2,CLIP1,CHTF18,FKBPL,SEC13,SEC13,CLSPN,BLM,BLM,CENPK,NCAPG,NCAPG,SET,COP1,SYCE3,ANAPC1,GORASP1,GORASP1,GINS3,GINS3,LPIN3,CENPH,SKP1,SKP2,ACD,ACD,H3C13,H3C13,MZT2A,SPAST,SRC,BRCA1,BRCA1,BRCA2,AURKA,SYCP1,BUB1,BUB1B,TERF1,TERF1,TERF2,TERF2,TERT,TFDP1,TFDP1,TFDP2,TFDP2,TK1,TMPO,TOP2A,TOP3A,TOP3A,TP53,TP53BP1,TPR,H2AC19,H2AC19,TUBA4A,TUBA3C,TUBB2A,TUBG1,CDK11A,TYMS,UBA52,UBB,UBC,UBE2D1,UBE2E1,UBE2I,UBE2I,UBE2N,UBE2V2,SUMO1,VRK1,VRK2,WEE1,WEE1,NSD2,WRN,XPO1,YWHAB,YWHAE,YWHAG,YWHAH,YWHAZ,ALMS1,TUBA1A,MIS12,CENPM,NUP37,NUP37,DSCC1,CENPO,BRCC3,HAUS3,CHMP6,MCPH1,CENPU,SEM1,TUBAL3,BORA,NUP85,NUP85,CEP76,DSN1,STN1,RMI1,MZT2B,PIF1,CENPT,CTC1,CEP290,NUP214,CEP63,CEP70,B9D2,AAAS,TUBB1,NDEL1,CDT1,RAB1B,RAB1B,SEH1L,SEH1L,KIF18A,SMC1A,SMC1A,H3-4,H3-4,H4C9,H4C9,CDC7,CDC45,CDC45,H2AC14,H2AC14,H2AC6,H2AC6,H2AC4,H2AC4,H2AC18,H2AC18,H2AC20,H2AC20,H2BC8,H2BC8,H2BC13,H2BC13,H2BC15,H2BC15,H2BC14,H2BC14,H2BC7,H2BC7,H2BC6,H2BC6,H2BC9,H2BC9,H2BC10,H2BC10,H2BC4,H2BC4,H2BC17,H2BC17,H2BC21,H2BC21,H3C1,H3C1,H3C4,H3C4,H3C3,H3C3,H3C6,H3C6,H3C11,H3C11,NUF2,H3C8,H3C8,H3C12,H3C12,H3C10,H3C10,H3C2,H3C2,H4C1,H4C1,H4C4,H4C4,H4C6,H4C6,H4C12,H4C12,H4C11,H4C11,H4C3,H4C3,H4C8,H4C8,H4C2,H4C2,H4C5,H4C5,H4C13,H4C13,RHNO1,H4C14,H4C14,MAD1L1,BRIP1,MND1,ATRIP,CEP78,ABRAXAS1,GINS4,GINS4,MCM8,MCM8,CUL1,TUBB6,KIF2B,SMARCA5,FKBP6,TUBA1C,RAE1,OFD1,MASTL,MASTL,AJUBA,H2BC12,H2BC12,TUBGCP6,BLZF1,CDC14A,RUVBL1,USO1,SSNA1,DYNLL1,CDC23,BANF1,CDC16,CCNA2,CCNA2,CCNA1,CCNA1,CCNB1,CCNB1,HERC2,CCND2,BTRC,CCND3,H3C7,H3C7,H2BC11,H2BC11,CCNE1,CCNH,RNF8,PKMYT1,PKMYT1,SMC3,SMC3,CCNB2,CCNB2,CCNE2,EXO1,CENPL,LIN52,LIN52,NEK9,CABLES1,CHMP7,ZW10,BUB3,AURKB,PTTG1,CHMP4C,HAUS8,SYCE1,H2AZ2,H2AZ2,PSMF1,KIF23,CEP41,BABAM2,NUP155,NUP155,MDC1,CEP135,LPIN2,NUP93,NUP93,ESPL1,CEP57,KNTC1,CCP110,CKAP5,IST1,SFI1,NUP58,NUP58,CDK1,CDK1,GINS1,GINS1,CDK11B,PSMD6,POM121,POM121,CDC6,CDC20,NCAPD2,NCAPD2,CDC25A,CDC25B,CDC25C,CDC27,NUP153,RBX1,REC8",Cell Cycle,862

R-HSA-1643685,"AKT3,ABI1,ABI1,HDAC6,HDAC5,PDCD6IP,BCL2L11,GNE,MAMLD1,ELOA3D,RNF103-CHMP3,AP1M2,ABCB6,ABCC9,MUC12,GPC6,ARPC5,ARPC5,ARPC4,ARPC4,ARPC3,ARPC3,ARPC1B,ARPC1B,ACTR3,ACTR3,ACTR2,ACTR2,OPN1MW3,ARPC2,ARPC2,AKAP9,ABI2,ABI2,TRIM28,WASF2,WASF2,CDK2,CDK4,ALG3,PSME3,ADAM10,CDK5,CDK5,CALCRL,CDK6,PSMD14,CDK7,CDK8,CDK9,CDK9,STAM2,CNKSR1,CDKN1A,RAMP2,RAMP1,RAMP3,CDKN1B,CDKN1C,SIGMAR1,CDKN2A,TLR6,TUBA1B,TUBB3,TUBB4A,TUBB4B,ANAPC10,SPON2,SPON1,VAV3,VAV3,BAIAP2,BAIAP2,SLC9A6,LINC02210-CRHR1,CEBPD,ATG7,ARPC1A,ARPC1A,SLC35A1,SLC34A2,POMT1,SLCO1B1,ST6GALNAC2,ERLIN1,TXNIP,TXNIP,CSPG5,GNB5,ADCY1,ADCY1,NRG3,NUP50,NUP50,AHCYL1,PLK2,NCKAP1,NCKAP1,ADCY2,ADCY2,CFTR,CYSLTR1,CGA,WASF3,WASF3,NOXA1,FRS2,FAM114A2,GPR83,CPSF4,ADCY3,ADCY3,SUGT1,CBX1,OS9,SLC27A4,RCC1,B4GAT1,TENT4A,CPSF6,CNTRL,UBE2C,KERA,ADAMTS13,ADAMTS8,ADAMTS5,NUP42,NUP42,ADCY5,ADCY5,FGFR1OP,SLC7A9,CDC37,CORO1A,ERLIN2,PSIP1,ADAMTS7,ADAMTS6,SUPT16H,ADCY6,ADCY6,DUSP10,RPL35,GPR176,GPR45,MAN1B1,SLC6A14,SNF8,TUBA3E,STX1B,FDX2,B4GALT7,ADCY7,ADCY7,PTH2,CHST14,TUBA3D,CYP2U1,ADCY8,ADCY8,NLRP3,NLRP3,TIRAP,CHUK,ADCY9,ADCY9,ADCYAP1,SLC22A12,NUS1,RPL39L,ADCYAP1R1,AP2M1,AP1S1,AP2S1,RLN3,ANTXR2,MUCL1,CLCN6,PIK3AP1,ANAPC16,CYP2R1,CLTA,CLTC,H4-16,RXFP2,PSMB11,GPHB5,SLC24A4,CCR5,NOXO1,ABCC2,CYP4F22,B3GALT6,H3C14,SYT2,H2BU1,CHMP4B,NUP35,NUP35,AP1S3,COMT,ADM,TAAR9,TAAR1,ADORA2A,CP,ADORA2B,VPS37A,HGSNAT,TAF1L,CREB1,CREB1,CREBBP,CRH,AMER1,CRHR1,CRHR2,CRK,RPS4Y2,MUC17,DYNLL2,ADAMTS14,RPL10L,PARP1,MIB2,SLC34A3,PARP4,MAPK14,PSMA8,MUC15,CSF2RA,CSF2RB,CSK,B3GLCT,CSNK1A1,RDH12,NRG4,VCAN,NCAN,CSPG4,WIPF2,WIPF2,TICAM1,CTBP1,CTBP2,CTNNB1,CTNND1,CTSG,GPBAR1,CTSL,CUX1,KLB,ADRB1,SLC36A2,CYBA,ADRB2,CYP1B1,ADRB3,VPS37D,SBSPON,CYP11A1,CYP11B1,CYP11B2,CYP17A1,CYP19A1,CYP21A2,CYP24A1,CYP27A1,CYP27B1,AP2A1,DAG1,AP2A2,DAXX,SPRED1,AP1B1,ELOA3,AP2B1,DCN,AP1G1,DDX5,MMAA,GPHA2,ADAMTS15,ADAMTS16,ADAMTS17,ADAMTS18,ADAMTS19,ACAN,DYNC1H1,DYNC1I1,DYNC1I2,DYNC1LI2,DOCK1,DOCK2,DPAGT1,DPEP1,SLC26A3,DRD1,DRD5,JAG1,SLC26A2,HBEGF,DUSP6,DUSP7,DUSP8,DUSP9,DVL1,DVL1,DVL2,DVL2,DVL3,DVL3,E2F1,E2F2,E2F3,ABCA1,S1PR1,AHCY,EEF2,EGF,EGFR,ADCY4,ADCY4,ALG14,ELK1,SPRED2,RPL22L1,MUC20,ENO1,TUBB,EP300,EPS15,ERBB2,ERBB3,ERBB4,ERCC2,EREG,AKT1,ERCC3,AKT2,ESR1,ABCA3,ESR2,ETV6,EXT1,EXT2,EZH2,F2,ABCD1,F8,F9,F10,F11,F12,FCGR1A,FCGR2A,FCGR3A,H2AC1,GPC2,THSD7A,NAPEPLD,FDX1,FDXR,FEN1,GPC4,FGA,FGB,FGF1,FGF1,FGF2,FGF2,FGF3,FGF4,FGF5,FGF6,FGF7,FGF8,FGF9,FGF10,FGFR1,FGFR3,GPC5,FGFR2,FGFR2,FGFR4,FGG,FGR,FKBP1A,MRAS,DOLK,CHSY1,CNKSR2,ALDOB,TRAK1,NCBP2,RPIA,SNW1,DKK1,SV2C,FOXO1,FOXO3,FOXO3,MPRIP,NUP205,NUP205,SLC35D1,KANK1,CYFIP1,CYFIP1,GANAB,PSME4,NUP210,NUP210,NUP160,NUP160,FMO3,FMOD,NEDD4L,FN1,NCSTN,SLC35A3,HEY1,BRD4,HEY2,NUP188,NUP188,SUZ12,RPL13A,NUP62,NUP62,LY96,ABCA4,CDC26,SLC17A8,MTOR,FSHB,FSHR,ABL1,RICTOR,FYN,G6PC,SLC37A4,XRCC6,GAA,GAB1,EPGN,H2BC1,ST6GALNAC3,GALE,GALK1,RPL36,GALNS,GALNT1,ANAPC15,GALNT3,GALT,NELFB,NELFB,SIN3A,MMACHC,CHMP2B,FGFR1OP2,ABCA12,B3GAT3,VPS33B,FGF20,GBE1,GCG,GCK,GCKR,KAT2A,SLC17A5,HEYL,OPN1MW,GFPT1,GGCX,GGT1,B4GALT1,GGT5,OPLAH,GHRH,GHRHR,CBLIF,GIP,GIPR,CYFIP2,CYFIP2,FGF22,NOX1,SND1,ST6GALNAC4,DKK4,DKK2,VPS4A,GPC3,GLB1,CHMP2A,ERLEC1,MMADHC,GCLC,GCLM,UBE2S,GLP1R,GNAI1,GNAI2,GNAI3,GNAS,GNAS,GNAZ,GNB1,GNB2,GNB3,GNG3,GNG4,GNG5,GNG7,GNG10,GNG11,GNGT1,GNGT2,GNS,GOLGA2,GOLGA4,GP1BA,GP1BB,GP5,GP9,GPC1,SLCO1B3,KSR2,MUC19,SLC24A5,GPR15,GPR20,GPR25,GPR27,DLL1,SLC9A9,GPR32,GPR150,GPR39,GPS2,GPS2,GRB2,GRB2,C1GALT1C1,NR3C1,CHMP4A,SLC25A4,PYCARD,TBK1,SLC25A5,GRSF1,SLC25A6,GSK3A,GSK3B,GSS,MSH6,GTF2A1,GTF2A2,GTF2B,GTF2E1,GTF2E2,GTF2F1,GTF2F1,GTF2F2,GTF2F2,GTF2H1,GTF2H2,GTF2H3,GTF2H4,GUCY2C,ICOS,ANAPC2,GUSB,GYG1,ALG6,ANAPC4,POMT2,GYS1,GYS2,SH3KBP1,SLC40A1,H2AC8,H2AC7,H2BC5,H2BC3,HCK,HDAC1,HDAC2,NCKAP1L,NCKAP1L,HEXA,HEXB,HGF,NRG1,PIK3R4,HK1,ACACA,HLA-A,HLCS,HMGA1,NR4A1,H2AC21,HNRNPK,TAAR6,APC,HRAS,MMAB,HRH2,HES1,HSPA1A,HSPA1B,HSP90AA1,HSP90AB1,HSPG2,H3C15,APOA1,HTR4,HTR6,HTR7,HYAL1,IAPP,ADAMTSL5,SLC6A19,CYP26C1,IDH1,IDS,IDUA,GNAT3,TUBB8,TUBB2B,SLC6A18,NUP43,NUP43,APP,RBPJ,IHH,IKBKB,IL1A,IL1B,IL1R1,IL1RAP,FASLG,IL6,IL6R,IL10,IL18,IMPDH1,IMPDH2,INSL3,IRS1,ITGA2B,ITGA4,ABCC6,ITGB1,ARAF,ITGB3,ITPR1,ITPR2,ITPR3,JAG2,JAK1,JAK2,JAK3,JUN,JUN,AREG,ARF1,AGRN,KCNJ11,NHLRC1,KDR,KHK,KIT,KLKB1,KPNA1,KPNB1,KPNB1,KPNA2,KPNA3,KPNA4,KPNA5,IPO5,KRAS,NPSR1,HES5,RHOG,RPSA,LCK,LCT,MUC21,LFNG,LHB,LHCGR,LIG1,LIG4,SPRED3,MYO18A,LMNA,LMNA,LMNB1,KPNA7,LRP6,LRP5,GTF2H5,LTF,LUM,LYN,ARRB1,SMAD2,SMAD3,SMAD4,ARRB2,ARSB,MAOA,MARK3,MAT1A,MC1R,MC2R,MC3R,MC4R,MC5R,CHST6,MDM2,MECP2,MECP2,MEFV,MET,MET,MGAT1,MGAT2,KITLG,MLH1,MAP3K11,FOXO4,MNAT1,MPI,MRC1,ALG11,H2BC18,MSH2,MSH3,MTR,MTRR,MUC1,MUC3A,MUC4,MUC5AC,MUC6,MUC7,MMUT,MUTYH,MUTYH,MYC,MYD88,MYH2,MYH9,MYO1C,MYO5A,MYO9B,MYO10,NAGLU,NCBP1,NCK1,NCK1,RPL10A,NEU1,ATP1A1,NF1,NFKB1,NFKB2,NFKBIA,NMT1,NOS2,NOTCH1,NOTCH2,NOTCH3,NOTCH4,NPM1,NPM1,SLC11A2,NRAS,NT5E,NTHL1,NUP88,NUP88,NUP98,NUP98,OMD,OGG1,OGN,SLC22A18,P2RX4,P2RX7,P2RY11,PEBP1,FURIN,PRDX1,PAH,PAK2,DHH,TRAT1,PC,PCCA,PCCB,DERL2,VPS36,RPS27L,APH1A,RPL26L1,IRAK4,DYNC1LI1,VPS28,DCXR,CRBN,CHMP1A,ELOA2,UBAP1,TLR7,CD320,PDCD1,FZR1,BIN2,ANAPC5,ANAPC7,NELFCD,NELFCD,CHMP5,NCKIPSD,NCKIPSD,ANAPC11,VTA1,PDGFA,PDGFB,PDGFRA,HDAC7,PDGFRB,ATP6V1H,TAF9B,CHMP3,PDPK1,SLC26A4,GNG13,TUBA8,CFP,PGK1,PGM1,ABCB4,PHB,PIK3C3,PIK3CA,PIK3CB,PIK3CD,PIK3R1,PIK3R2,PLCG1,PLCG2,NUP54,NUP54,PML,PMM2,GPR84,PMS2,TLR9,POLR2A,POLR2A,POLR2B,POLR2B,POLR2C,POLR2C,POLR2D,POLR2D,GNG2,POLR2E,POLR2E,DPM3,POLR2F,POLR2F,POLR2G,POLR2G,POLR2H,POLR2H,POLR2I,POLR2I,POLR2J,POLR2J,POLR2K,POLR2K,POLR2L,POLR2L,POMC,SEMA5B,TAF7L,NEURL1B,ADAMTSL4,APBB1IP,DLL4,PARP14,UGT1A4,UGT1A1,SLC6A20,CTSA,PPIA,ESRP1,RNF43,PARP16,PPP1CB,PPP1CC,VPS37C,PPP1R3C,AVP,AGGF1,PPP2CA,PPP2CB,PPP2R1A,PPP2R1B,AVPR1A,NEIL3,PPP2R5A,PPP2R5B,PPP2R5C,PPP2R5D,PPP2R5E,FBXW7,AVPR1B,SLC29A3,SLC35C1,AVPR2,H4C15,PRELP,MAML3,PRR5,POMGNT1,SLC39A4,PRKACA,PRKACA,PRKACB,PRKACB,PRKACG,PRKACG,PACS1,NDC1,NDC1,PRKAR1A,HHAT,NUP133,NUP133,PRKAR1B,AGK,PRKAR2A,PRKAR2B,LMBRD1,BRK1,BRK1,PSENEN,HDAC8,PRKCSH,THSD1,ERBIN,MAPK1,MAPK3,GNG12,MAPK8,MAP2K1,MAP2K2,ALG1,MAP2K3,MAP2K6,MAP2K7,EIF2AK2,DNAJC3,PRKX,CYP26B1,SLC2A9,PSEN1,PSEN2,MUC13,B2M,PSMA1,ZC3HAV1,PSMA2,PSMA3,PSMA4,PSMA5,PSMA6,PSMA7,PSMB1,PSMB2,PSMB3,C1GALT1,PSMB4,MCCC1,PSMB5,PSMB6,PSMB7,PSMB8,PARP6,PSMB9,PSMB10,ADAMTS9,PSMC1,PSMC2,PSMC3,PSMC4,PSMC5,PSMC6,PSMD1,PSMD2,AGTRAP,PSMD3,PSMD4,CYSLTR2,PSMD5,NUP107,NUP107,PSMD7,PSMD8,PSMD9,PSMD10,PSMD11,PSMD12,ADAMTSL3,PSMD13,BAD,PSME1,PSME2,PTEN,PTGDR,PTGER2,PTGER4,PTGIR,PTH,PTHLH,PTH1R,PTH2R,PTK2,MIB1,WDR48,KIAA1549,RNF213,PTPN11,PTPN12,ABCD4,RAB5A,RAC1,RAC1,RAC2,RAF1,RAN,RANBP1,RANBP2,RANBP2,RANGAP1,RAP1A,RAP1B,RB1,ACE2,RBBP4,RBBP7,GNB4,RXFP1,RBP1,NPS,CCND1,RBP4,RDH5,RELA,TRIM27,ACTB,ACTB,RHAG,RLBP1,RLN2,RNF5,SLC5A7,APOBEC3G,ROCK1,OPN1SW,RPL3,RPL3L,RPL4,RPL5,RPL6,RPL7,BCR,RPL7A,RPL8,RPL9,RPL10,RPL11,RPL12,RPL13,RPL15,RPL17,RPL18,RPL18A,RPL19,RPL21,RPL22,RPL23A,RPL24,RPL26,RPL27,RPL30,RPL27A,RPL28,RPL29,RPL31,RPL32,RPL34,RPL35A,RPL36AL,RPL37,RPL37A,RPL38,RPL39,RPL41,RPL36A,RPLP0,RPLP1,RPLP2,RPS2,RPS3,RPS3A,RPS4X,RPS4Y1,RPS5,RPS6,RPS6KB2,RPS7,RPS8,RPS9,RPS10,RPS11,RPS12,RPS13,RPS14,RPS15,RPS15A,RPS16,RPS17,RPS18,RPS19,RPS20,RPS21,RPS23,RPS24,RPS25,RPS26,RPS27,RPS27A,RPS27A,RPS28,RPS29,BGN,SCT,SCTR,SDC1,BCAN,SDC2,SDC4,ELMO2,SEC13,SEC13,SEL1L,MCCC2,MAP2K4,DPEP2,DPEP3,SFPQ,STRA6,MLST8,ABCG5,ABCG8,SFTPB,SFTPC,SFTPD,WIPF3,WIPF3,SGSH,SH3GL1,SH3GL2,SH3GL3,SHC1,ANAPC1,SHH,SI,SI,ST6GAL1,ST3GAL1,ST3GAL2,ST3GAL4,PORCN,ST3GAL3,SKP1,SKP2,SLC1A1,SLC1A3,SLC2A1,SLC2A2,SLC3A1,SLC3A2,SLC4A1,SLC5A1,SLC5A2,SLC5A5,SLC6A2,SLC6A3,SFTPA1,H3C13,SLC12A1,SLC12A3,SLC16A1,SLC34A1,SLC20A2,SLCO2A1,SLC22A5,SNAP25,SOD2,SOS1,SRC,BRAF,SSRP1,STAT1,STAT3,STAT5A,STAT5B,STRN,STX1A,BSG,SUPT4H1,SUPT4H1,SUPT5H,SUPT5H,ABCC8,VAMP1,VAMP2,BTC,SYK,SYT1,BTD,ADAM17,TAF1,TAF2,TAF4,TAF4B,TAF5,TAF6,TAF7,TAF9,TAF10,TAF11,TAF12,TAF13,TALDO1,TBL1X,TBL1X,TBP,TBXAS1,TCEA1,ELOC,ELOC,ELOB,ELOB,ELOA,TCF7L2,TCN2,BTK,PRDX2,TFDP1,TFDP2,TGFA,TGFB1,TGFBR1,TGFBR2,THBS1,THBS2,C1QBP,TLN1,TLR1,TLR2,TLR3,TLR4,ACTG1,ACTG1,SERPING1,TLR5,TMPRSS2,TPMT,TPR,TPR,C3,TRAF3,C3AR1,H2AC19,TSC2,TSG101,TSHB,TSHR,TTR,TUBA4A,TUBA3C,MUC5B,TUBB2A,OPN1MW2,ELOA3B,SFTPA2,TXN,TXN,TXNRD1,TYK2,UBA52,UBA52,UBB,UBB,UBC,UBC,UBE2D1,UBE2E1,UBE2I,SUMO1,SLC35A2,UVRAG,VAV1,VAV1,VAV2,VAV2,VCL,VCP,VEGFA,VHL,VIP,VIPR1,VIPR2,VWF,WAS,WAS,WIPF1,WIPF1,NELFA,NELFA,WNT5A,WNT5A,XPO1,XRCC4,XRCC5,YES1,YWHAB,YWHAE,ZMYM2,MOGS,TUBA1A,CXCR4,FZD5,RAB7A,NUP37,NUP37,ALG8,ALG12,MAPKAP1,DERL1,NELFE,NELFE,KREMEN2,EPM2A,CALCA,CHMP6,SRD5A3,RHBDF2,NEIL1,PARP8,GALNT12,CALCB,TBL1XR1,TBL1XR1,VPS37B,SEM1,ALG9,PDZD3,TUBAL3,ALG13,CBLL1,THSD4,HDAC11,CALCR,NUP85,NUP85,ADM2,DHDDS,ADAMTS20,CALM1,CALM1,NUP214,NUP214,STAM,CUBN,TNKS2,SHOC2,CALM2,CALM2,CUL5,THSD7B,FGF23,CALM3,CALM3,DUSP16,KDM7A,AAAS,AAAS,FXR1,TUBB1,SLC2A10,PABPN1,CALR,CAMK4,TAF15,CAMK2A,CAMK2B,FIP1L1,UNC93B1,MAP1LC3B,AMN,CAMK2D,ELL,ADAMTS12,TLR10,ADAMTS10,CAMK2G,SEH1L,SEH1L,CANX,CAPN1,CAPN1,CAPN2,CAPN2,CAPNS1,CAPNS1,H4C9,CAST,CAST,AXIN1,BRAP,FZD4,FZD6,FZD7,FZD7,FZD8,H2AC13,H2AC15,H2AC14,H2AC16,H2AC6,H2AC4,H2AC17,H2AC18,H2AC20,H2BC8,CASP1,H2BC13,H2BC15,H2BC14,H2BC7,H2BC6,H2BC9,H2BC10,APH1B,H2BC4,H2BC17,H2BC21,H3C1,H3C4,H3C3,H3C6,H3C11,H3C8,TAAR8,H3C12,H3C10,H3C2,H4C1,H4C4,H4C6,H4C12,H4C11,H4C3,H4C8,H4C2,PARP9,H4C5,H4C13,H4C14,TAF3,HDAC10,KREMEN1,ANTXR1,CASP9,CAPNS2,CAPNS2,VPS25,AKT1S1,MAML2,SYVN1,CUL1,TPST2,TPST1,TUBB6,TUBA1C,RAE1,RAE1,PARP10,PIK3R3,IKBKG,H2AC12,H2BC12,ALG2,ZCRB1,AP3B1,ABCB11,DYNLL1,TNKS,IRS2,CBL,CBL,SLC4A4,BECN1,CDC23,EED,RNMT,RNGTT,RIPK1,TRIM24,CCNK,DPM1,BANF1,FGF18,DPM2,FGF17,FGF16,IQGAP1,HDAC3,HDAC3,KSR1,KAT2B,CDK5R1,CDK5R1,CDC16,AP1S2,AP1M1,GYG2,CCNC,WASF1,WASF1,CCND2,BTRC,CCND3,H3C7,H2AC11,H2BC11,WASL,WASL,WNT3A,CCNE1,MVB12B,CCNH,SEMA5A,TAAR5,CCNT1,CCNT1,RPL14,CCNT2,PSTPIP1,SLC7A7,PAPSS2,PAPSS1,DERL3,CCNE2,RNF185,HGS,HGS,NEURL1,CTDP1,CTDP1,SLC6A5,CD3G,CHMP7,RFT1,SLC24A1,CD247,SLC33A1,CD4,LRRFIP1,LARGE1,LRAT,CHMP4C,G6PC3,CD8B,CD9,H2AW,TAAR2,CD14,ADAMTSL1,CD19,CD163,MVB12A,GLP2R,RPL23,KL,ZFYVE9,NMT2,CD28,MUC16,CD80,CD86,CYP7B1,GNG8,QKI,CHST3,ROCK2,CD36,PSMF1,ACY1,ADAMTS4,ADAMTS3,ADAMTS2,ADAMTS1,VPS4B,MPDU1,ENTPD1,BAG4,NRG2,ENTPD5,NCOR1,NCOR1,NCOR2,NCOR2,NUP155,NUP155,ISG15,SDC3,NUP93,NUP93,FAM131B,ADAMTSL2,HDAC9,HDAC4,MAML1,NUP58,NUP58,HEPH,ELMO1,GAB2,PSMD6,POM121,POM121,SV2B,SV2A,CDC25A,CDC25B,CDC25C,CDC27,FGF19,NUP153,NUP153,RBX1,CDC42,CDC42,CDH1,SLC12A6",Disease,1669

R-HSA-1643713,"CDC37,HBEGF,EGF,EGFR,EREG,GAB1,EPGN,GRB2,HRAS,HSP90AA1,AREG,KRAS,NRAS,PIK3CA,PIK3R1,PLCG1,RPS27A,SHC1,SOS1,BTC,TGFA,UBA52,UBB,UBC,CBL",Signaling by EGFR in Cancer,25

R-HSA-164378,"ADCY1,ADCY2,ADCY3,ADCY5,ADCY6,ADCY7,ADCY8,ADCY9,ADCY4,GNAS,PRKACA,PRKACB,PRKACG,PRKAR1A,PRKAR1B,PRKAR2A,PRKAR2B",PKA activation in glucagon signalling,17

R-HSA-164516,PPIA,Minus-strand DNA synthesis,1

R-HSA-164525,PPIA,Plus-strand DNA synthesis,1

R-HSA-164843,"PSIP1,XRCC6,HMGA1,LIG4,XRCC4,XRCC5,BANF1",2-LTR circle formation,7

R-HSA-164938,"AP1M2,AP2M1,AP1S1,AP2S1,AP1S3,AP2A1,AP2A2,AP1B1,AP2B1,AP1G1,HLA-A,ARF1,LCK,ATP6V1H,PACS1,B2M,AP1S2,AP1M1,CD4,CD8B,CD28",Nef-mediates down modulation of cell surface receptors by recruiting them to clathrin adapters,21

R-HSA-164939,"AP2M1,CD28",Nef mediated downregulation of CD28 cell surface expression,2

R-HSA-164940,"AP1M2,AP1S1,AP1S3,AP1B1,AP1G1,HLA-A,PACS1,B2M,AP1S2,AP1M1",Nef mediated downregulation of MHC class I complex cell surface expression,10

R-HSA-164944,"DOCK2,FYN,HCK,LCK,PAK2,RAC1,CD247,ELMO1",Nef and signal transduction,8

R-HSA-164952,"AP1M2,AP2M1,AP1S1,AP2S1,AP1S3,AP2A1,AP2A2,AP1B1,AP2B1,AP1G1,DOCK2,FYN,HCK,HLA-A,ARF1,LCK,PAK2,ATP6V1H,PACS1,B2M,RAC1,AP1S2,AP1M1,CD247,CD4,CD8B,CD28,ELMO1",The role of Nef in HIV-1 replication and disease pathogenesis,28

R-HSA-165054,"NUP50,RCC1,NUP42,NUP35,NUP205,NUP210,NUP160,NUP188,NUP62,NUP43,NUP88,NUP98,NUP54,NDC1,NUP133,NUP107,RAN,RANBP1,RANBP2,RANGAP1,SEC13,TPR,XPO1,NUP37,NUP85,NUP214,AAAS,SEH1L,RAE1,NUP155,NUP93,NUP58,POM121,NUP153",Rev-mediated nuclear export of HIV RNA,34

R-HSA-1650814,"CRTAP,P3H3,COL1A1,COL1A2,COL2A1,COL3A1,COL4A1,COL4A2,COL4A3,COL4A4,COL4A5,COL4A6,COL5A1,COL5A2,COL6A1,COL6A2,COL6A3,COL7A1,COL8A1,COL8A2,COL9A1,COL9A2,COL9A3,COL10A1,COL11A1,COL11A2,COL12A1,COL13A1,COL15A1,COL16A1,COL17A1,COL19A1,COL6A6,COL26A1,ADAMTS14,COL22A1,COLGALT2,COL24A1,COL6A5,PCOLCE2,P4HA3,COL28A1,P4HA1,P4HB,COL5A3,PCOLCE,PLOD1,PLOD2,PPIB,P3H2,COL20A1,P3H1,BMP1,TLL1,TLL2,COL14A1,COLGALT1,COL18A1,COL21A1,COL25A1,COL27A1,SERPINH1,P4HA2,PLOD3,COL23A1,ADAMTS3,ADAMTS2",Collagen biosynthesis and modifying enzymes,67

R-HSA-165158,"THEM4,AKT2,PDPK1,TRIB3",Activation of AKT2,4

R-HSA-165159,"RRAGB,LAMTOR5,RRAGA,SLC38A9,EIF4B,EIF4E,EIF4EBP1,EIF4G1,AKT1,AKT2,MTOR,LAMTOR2,EEF2K,LAMTOR4,PRKAG2,CAB39,PRKAG3,PPM1A,LAMTOR1,STRADB,PRKAA1,PRKAA2,PRKAB1,PRKAB2,PRKAG1,RPTOR,RRAGD,RHEB,RPS6,RPS6KB1,RRAGC,MLST8,STK11,TSC1,TSC2,YWHAB,CAB39L,AKT1S1,LAMTOR3,STRADA",MTOR signalling,40

R-HSA-165160,"AKT2,PDE3B,PDE3B",PDE3B signalling,3

R-HSA-165181,"AKT2,TSC1,TSC2",Inhibition of TSC complex formation by PKB,3

R-HSA-1655829,"SEC24B,SEC23A,CARM1,NCOA2,PMVK,SEC24A,CREBBP,CYP51A1,DHCR7,FASN,FDFT1,FDPS,SCAP,SCAP,NCOA6,ACACA,HMGCR,HMGCS1,ACACB,IDI1,INSIG1,KPNB1,LSS,MTF1,MVD,MVK,NFYA,NFYA,NFYB,NFYB,NFYC,NFYC,SAR1B,INSIG2,MBTPS2,PPARA,MED1,GPAM,RAN,RXRA,SC5D,SCD,SMARCD3,SP1,SQLE,SREBF1,SREBF1,SREBF2,SREBF2,TBL1X,TM7SF2,ELOVL6,TBL1XR1,CHD9,HELZ2,NCOA1,MBTPS1,GGPS1,SEC24C,TGS1,SEC24D",Regulation of cholesterol biosynthesis by SREBP (SREBF),61

R-HSA-166016,"TANK,TMED7-TICAM2,TLR6,NOD1,TAB1,RIPK3,IRAK3,TIRAP,CHUK,MAP3K8,MAP3K8,CREB1,ATF2,MAPK14,TICAM1,DNM1,AGER,DNM2,DUSP3,DUSP4,DUSP6,DUSP7,ELK1,SARM1,TAB2,FBXW11,FOS,LY96,PELI3,TAB3,DNM3,NKIRAS2,NKIRAS1,TBK1,HMGB1,BIRC2,BIRC3,APP,TICAM2,IKBKB,IRAK1,IRAK2,IRF3,IRF7,ITGAM,ITGB2,JUN,TMEM189-UBE2V1,LBP,CD180,MEF2A,MEF2C,MAP3K1,MYD88,ATF1,NFKB1,NFKB2,NFKBIA,NFKBIB,IRAK4,VRK3,ECSIT,PLCG2,PPP2CA,PPP2CB,PPP2R1A,PPP2R1B,PPP2R5D,MAPK1,MAPK1,MAPK3,MAPK3,MAPK7,MAPK7,MAPK8,MAPK11,MAPK9,MAPK10,MAP2K1,MAP2K3,MAP2K6,MAP2K7,PELI2,PELI1,PTPN4,PTPN11,SIGIRR,RELA,RPS6KA1,RPS6KA2,RPS6KA3,RPS27A,S100A12,S100B,SAA1,NOD2,MAP2K4,MAP2K4,SFTPD,SKP1,SFTPA1,BPI,MAP3K7,BTK,TLR1,TLR2,TLR4,TRAF3,TRAF6,SFTPA2,UBA52,UBB,UBC,UBE2D1,UBE2D2,UBE2D3,UBE2N,UBE2V1,MAPKAPK3,TNIP2,CASP8,CUL1,IKBKG,SOCS1,RIPK1,RIPK2,FADD,BTRC,RPS6KA5,MAPKAPK2,CD14,LY86,CD36,IKBKE",Toll Like Receptor 4 (TLR4) Cascade,134

R-HSA-166020,"LBP,CD14",Transfer of LPS from LBP carrier to CD14,2

R-HSA-1660499,"PIK3R6,PLEKHA6,PIP5K1C,PIK3R5,INPP5J,INPP4A,INPP5D,INPPL1,ARF1,MTM1,OCRL,RAB14,INPP5K,PIK3C2A,PIK3C2B,PIK3C2G,PIK3CA,PIK3CB,PIK3CD,PIK3CG,PIK3R1,PIK3R2,PIP4K2A,PLEKHA5,PI4K2B,PI4K2A,MTMR8,PTEN,PLEKHA4,PTPN13,RAB4A,RAB5A,PLEKHA1,PLEKHA2,MTMR14,PLEKHA3,BMX,MTMR9,PIP4K2C,RUFY1,SBF2,PIP5K1A,PIP5K1B,PIP4K2B,PLEKHA8,PIK3R3,MTMR1,INPP4B,SYNJ1,SYNJ2,MTMR3,MTMR2,MTMR6",Synthesis of PIPs at the plasma membrane,53

R-HSA-1660514,"PIKFYVE,PIKFYVE,SACM1L,PIK3R4,ARF1,ARF3,OCRL,PIK3C2A,PIK3C2G,PIK3C3,PI4KA,PI4KB,PI4K2B,PI4K2A,VAC14,VAC14,INPP5E,TPTE,TPTE2,FIG4,FIG4",Synthesis of PIPs at the Golgi membrane,21

R-HSA-1660516,"PIKFYVE,PIKFYVE,INPP5F,PIK3R4,INPP4A,MTM1,PIK3C2A,PIK3C3,MTMR12,MTMR10,PI4K2B,PI4K2A,VAC14,VAC14,INPP4B,MTMR2,MTMR2,MTMR4,FIG4,FIG4",Synthesis of PIPs at the early endosome membrane,20

R-HSA-1660517,"PIKFYVE,PIKFYVE,PIK3R4,MTM1,PIK3C2A,PIK3C3,VAC14,VAC14,MTMR9,MTMR2,MTMR7,MTMR7,MTMR4,FIG4,FIG4",Synthesis of PIPs at the late endosome membrane,15

R-HSA-166058,"TLR6,NOD1,TAB1,IRAK3,TIRAP,CHUK,MAP3K8,MAP3K8,CREB1,ATF2,MAPK14,AGER,DUSP3,DUSP4,DUSP6,DUSP7,ELK1,TAB2,FBXW11,FOS,LY96,PELI3,TAB3,NKIRAS2,NKIRAS1,HMGB1,APP,IKBKB,IRAK1,IRAK2,JUN,TMEM189-UBE2V1,MEF2A,MEF2C,MAP3K1,MYD88,ATF1,NFKB1,NFKB2,NFKBIA,NFKBIB,IRAK4,VRK3,ECSIT,PPP2CA,PPP2CB,PPP2R1A,PPP2R1B,PPP2R5D,MAPK1,MAPK1,MAPK3,MAPK3,MAPK7,MAPK7,MAPK8,MAPK11,MAPK9,MAPK10,MAP2K1,MAP2K3,MAP2K6,MAP2K7,PELI2,PELI1,SIGIRR,RELA,RPS6KA1,RPS6KA2,RPS6KA3,RPS27A,S100A12,S100B,SAA1,NOD2,MAP2K4,MAP2K4,SKP1,MAP3K7,BTK,TLR1,TLR2,TLR4,TRAF6,UBA52,UBB,UBC,UBE2N,UBE2V1,MAPKAPK3,TNIP2,CUL1,IKBKG,SOCS1,RIPK2,BTRC,RPS6KA5,MAPKAPK2,CD14,CD36",MyD88:MAL(TIRAP) cascade initiated on plasma membrane,100

R-HSA-1660661,"CERT1,SPTLC1,CERS1,DEGS2,ACER1,SGPP2,SAMD8,CSNK1G2,PPM1L,SPTSSB,SGMS2,SPTSSA,CERS3,ALDH3B1,ALDH3A2,PRKD3,KDSR,CERS6,PRKD2,SGMS1,ORMDL2,CERS2,ACER2,OSBP,SPTLC3,ACER3,PRKD1,SPHK2,FA2H,CERS4,SGPP1,DEGS1,PLPP1,PLPP2,PLPP3,SPHK1,SGPL1,CERS5,VAPB,VAPA,ORMDL1,ORMDL3,SPTLC2",Sphingolipid de novo biosynthesis,43

R-HSA-1660662,"NEU3,NEU4,ARSK,ARSG,ESYT1,GALC,B4GALNT1,SUMF2,GBA,GLA,GLB1,GM2A,SUMF1,HEXA,HEXB,ENPP7,ARSI,ARSH,ARSA,ARSB,STS,ARSD,ARSL,ARSF,ASAH1,NEU1,NEU2,GLTP,CTSA,SMPD3,SMPD4,PSAP,ASAH2,ESYT2,GBA2,GBA3,CERK,SMPD1,SMPD2,UGCG,UGT8,GLB1L,ARSJ,CPTP,ESYT3,B3GALNT1",Glycosphingolipid metabolism,46

R-HSA-166166,"TANK,TMED7-TICAM2,NOD1,TAB1,RIPK3,CHUK,MAP3K8,MAP3K8,CREB1,ATF2,MAPK14,TICAM1,AGER,DUSP3,DUSP4,DUSP6,DUSP7,ELK1,SARM1,TAB2,FBXW11,FOS,LY96,TAB3,NKIRAS2,NKIRAS1,TBK1,HMGB1,BIRC2,BIRC3,APP,TICAM2,IKBKB,IRAK1,IRAK2,IRF3,IRF7,JUN,TMEM189-UBE2V1,MEF2A,MEF2C,ATF1,NFKB1,NFKB2,NFKBIA,NFKBIB,VRK3,PPP2CA,PPP2CB,PPP2R1A,PPP2R1B,PPP2R5D,MAPK1,MAPK1,MAPK3,MAPK3,MAPK7,MAPK7,MAPK8,MAPK11,MAPK9,MAPK10,MAP2K1,MAP2K3,MAP2K6,MAP2K7,PTPN11,RELA,RPS6KA1,RPS6KA2,RPS6KA3,RPS27A,S100A12,S100B,SAA1,NOD2,MAP2K4,MAP2K4,SKP1,MAP3K7,TLR4,TRAF3,TRAF6,UBA52,UBB,UBC,UBE2D1,UBE2D2,UBE2D3,UBE2N,UBE2V1,MAPKAPK3,TNIP2,CASP8,CUL1,IKBKG,RIPK1,RIPK2,FADD,BTRC,RPS6KA5,MAPKAPK2,CD14,IKBKE",MyD88-independent TLR4 cascade ,104

R-HSA-166187,"PM20D1,UCP1,UCP2,UCP3,SLC25A14,SLC25A27",Mitochondrial Uncoupling,6

R-HSA-166208,"RRAGB,LAMTOR5,RRAGA,SLC38A9,EIF4B,EIF4E,EIF4EBP1,EIF4G1,MTOR,LAMTOR2,EEF2K,LAMTOR4,LAMTOR1,RPTOR,RRAGD,RHEB,RPS6,RPS6KB1,RRAGC,MLST8,YWHAB,AKT1S1,LAMTOR3",mTORC1-mediated signalling,23

R-HSA-1663150,"ARSK,ARSG,SUMF2,SUMF1,ARSI,ARSH,ARSA,ARSB,STS,ARSD,ARSL,ARSF,ARSJ",The activation of arylsulfatases,13

R-HSA-166520,"DNAL4,CDK5,TRIB1,FRS3,FRS2,FRS2,CHD4,ADCYAP1,ADCYAP1R1,AP2M1,AP2M1,AP2S1,AP2S1,CLTA,CLTA,CLTC,CLTC,ADORA2A,CREB1,CREB1,ATF2,ATF2,CRK,CRK,CRKL,CRKL,MAPK14,MAPK14,AP2A1,AP2A1,AP2A2,AP2A2,AP2B1,AP2B1,DNM1,DNM2,DOCK3,DOCK3,DUSP3,DUSP4,DUSP6,DUSP7,EGR1,EGR1,EGR2,EGR2,EGR3,EGR3,EGR4,ELK1,ELK1,EP300,EP300,F3,ARC,ARC,FOS,FOSB,FYN,FYN,GAB1,SHC2,SHC2,NELFB,DNM3,GRB2,GRB2,RAPGEF1,RAPGEF1,GRIN2B,HRAS,HRAS,ID1,ID2,ID3,ID4,IRS1,IRS1,JUNB,JUND,KRAS,KRAS,RHOA,LYL1,MEF2A,MEF2C,MEF2D,MEF2D,ASCL1,ASCL1,ATF1,ATF1,NAB1,NAB2,NAB2,NGF,NGF,NRAS,NRAS,NTF3,NTF3,NTF4,NTF4,NTRK1,NTRK1,NTRK2,NTRK2,NTRK3,NTRK3,FURIN,PCSK6,VRK3,PCSK5,PIK3CA,PIK3CA,PIK3CB,PIK3R1,PIK3R1,PIK3R2,PLCG1,PLCG1,SHC3,SHC3,PPP2CA,PPP2CB,PPP2R1A,PPP2R1B,PPP2R5D,MAPK1,MAPK1,MAPK3,MAPK3,MAPK7,MAPK7,MAPK11,MAPK11,MAPK13,MAP2K1,MAP2K2,MAP2K5,KIDINS220,KIDINS220,PTPN11,PTPRO,PTPRS,BAX,RAC1,RAC1,RALA,RALB,RALGDS,RALGDS,RAP1A,RAP1A,REST,RIT2,RIT1,RPS6KA1,RPS6KA2,RPS6KA3,RRAD,BDNF,BDNF,MAPK12,SGK1,SH3GL2,SH3GL2,SH3GL3,SHC1,SHC1,SOS1,SOS1,SRC,SRC,SRF,SRF,BRAF,BRAF,STAT3,TCF12,TCF12,TIAM1,TPH1,VGF,YWHAB,YWHAB,MAPKAPK3,FOSL1,IRS2,IRS2,CDK5R1,CDK5R1,CDK5R2,RPS6KA5,MAPKAPK2",Signaling by NTRKs,195

R-HSA-166658,"C4B_2,COLEC10,MASP2,CFHR4,CFHR3,CLU,CPB2,CPN1,CPN2,CR1,CR2,CRP,CD55,CFD,ELANE,F2,FCN1,FCN2,C5AR2,GZMM,CFH,CFHR1,CFHR2,CFI,MBL2,CD46,CFP,PROS1,MASP1,CFB,SERPING1,C1QA,C1QB,C1QC,C1R,C1S,C2,C3,C3AR1,C4A,C4B,C4BPA,C4BPB,C5,C5AR1,C6,C7,C8A,C8B,C8G,C9,VTN,COLEC11,CFHR5,FCN3,CD19,CD59,CD81",Complement cascade,58

R-HSA-166662,"COLEC10,MASP2,FCN1,FCN2,MBL2,MASP1,COLEC11,FCN3",Lectin pathway of complement activation,8

R-HSA-166663,"C4B_2,COLEC10,MASP2,CRP,CFD,FCN1,FCN2,GZMM,MBL2,CFP,MASP1,CFB,C1QA,C1QB,C1QC,C1R,C1S,C2,C3,C4A,C4B,COLEC11,FCN3",Initial triggering of complement,23

R-HSA-166665,"CLU,C5,C6,C7,C8A,C8B,C8G,C9",Terminal pathway of complement,8

R-HSA-166786,"COLEC10,MASP2,CRP,FCN1,FCN2,MBL2,MASP1,C1QA,C1QB,C1QC,C1R,C1S,COLEC11,FCN3",Creation of C4 and C2 activators,14

R-HSA-167021,"NGF,NTRK1,PLCG1",PLC-gamma1 signalling,3

R-HSA-167044,"MAPK14,MAPK14,SHC2,SHC2,GRB2,GRB2,HRAS,HRAS,KRAS,KRAS,NGF,NRAS,NRAS,NTRK1,SHC3,SHC3,MAPK11,MAPK11,MAPK13,RALA,RALB,RALGDS,RALGDS,MAPK12,SHC1,SHC1,SOS1,SOS1,SRC,MAPKAPK3,MAPKAPK2",Signalling to RAS,31

R-HSA-167060,"NGF,NGF,FURIN,PCSK6,PCSK5",NGF processing,5

R-HSA-167152,"ELOA3D,CDK7,CDK9,SUPT16H,ELOA3,ERCC2,ERCC3,NCBP2,NELFB,GTF2F1,GTF2F2,GTF2H1,GTF2H2,GTF2H3,GTF2H4,GTF2H5,MNAT1,NCBP1,ELOA2,NELFCD,POLR2A,POLR2B,POLR2C,POLR2D,POLR2E,POLR2F,POLR2G,POLR2H,POLR2I,POLR2J,POLR2K,POLR2L,SSRP1,SUPT4H1,SUPT5H,TCEA1,ELOC,ELOB,ELOA,ELOA3B,NELFA,NELFE,ELL,CCNK,CCNH,CCNT1,CCNT2,CTDP1",Formation of HIV elongation complex in the absence of HIV Tat,48

R-HSA-167158,"CDK7,ERCC2,ERCC3,NCBP2,NELFB,GTF2F1,GTF2F2,GTF2H1,GTF2H2,GTF2H3,GTF2H4,GTF2H5,MNAT1,NCBP1,NELFCD,POLR2A,POLR2B,POLR2C,POLR2D,POLR2E,POLR2F,POLR2G,POLR2H,POLR2I,POLR2J,POLR2K,POLR2L,SUPT4H1,SUPT5H,NELFA,NELFE,CCNH,CTDP1",Formation of the HIV-1 Early Elongation Complex,33

R-HSA-167160,"CDK7,ERCC2,ERCC3,GTF2F1,GTF2F2,GTF2H1,GTF2H2,GTF2H3,GTF2H4,GTF2H5,MNAT1,POLR2A,POLR2B,POLR2C,POLR2D,POLR2E,POLR2F,POLR2G,POLR2H,POLR2I,POLR2J,POLR2K,POLR2L,SUPT5H,RNMT,RNGTT,CCNH",RNA Pol II CTD phosphorylation and interaction with CE during HIV infection,27

R-HSA-167161,"CDK7,TAF1L,ERCC2,ERCC3,GTF2A1,GTF2A2,GTF2B,GTF2E1,GTF2E2,GTF2F1,GTF2F2,GTF2H1,GTF2H2,GTF2H3,GTF2H4,GTF2H5,MNAT1,TAF9B,POLR2A,POLR2B,POLR2C,POLR2D,POLR2E,POLR2F,POLR2G,POLR2H,POLR2I,POLR2J,POLR2K,POLR2L,TAF7L,TAF1,TAF2,TAF4,TAF4B,TAF5,TAF6,TAF7,TAF9,TAF10,TAF11,TAF12,TAF13,TBP,TAF15,TAF3,CCNH",HIV Transcription Initiation,47

R-HSA-167162,"CDK7,TAF1L,ERCC2,ERCC3,GTF2A1,GTF2A2,GTF2B,GTF2E1,GTF2E2,GTF2F1,GTF2F2,GTF2H1,GTF2H2,GTF2H3,GTF2H4,GTF2H5,MNAT1,TAF9B,POLR2A,POLR2B,POLR2C,POLR2D,POLR2E,POLR2F,POLR2G,POLR2H,POLR2I,POLR2J,POLR2K,POLR2L,TAF7L,TAF1,TAF2,TAF4,TAF4B,TAF5,TAF6,TAF7,TAF9,TAF10,TAF11,TAF12,TAF13,TBP,TAF15,TAF3,CCNH",RNA Polymerase II HIV Promoter Escape,47

R-HSA-167169,"ELOA3D,CDK7,CDK9,CDK9,SUPT16H,ELOA3,ERCC2,ERCC3,NCBP2,NELFB,NELFB,GTF2F1,GTF2F1,GTF2F2,GTF2F2,GTF2H1,GTF2H2,GTF2H3,GTF2H4,GTF2H5,MNAT1,NCBP1,ELOA2,NELFCD,NELFCD,POLR2A,POLR2A,POLR2B,POLR2B,POLR2C,POLR2C,POLR2D,POLR2D,POLR2E,POLR2E,POLR2F,POLR2F,POLR2G,POLR2G,POLR2H,POLR2H,POLR2I,POLR2I,POLR2J,POLR2J,POLR2K,POLR2K,POLR2L,POLR2L,SSRP1,SUPT4H1,SUPT4H1,SUPT5H,SUPT5H,TCEA1,ELOC,ELOB,ELOA,ELOA3B,NELFA,NELFA,NELFE,NELFE,ELL,CCNH,CCNT1,CCNT1,CTDP1,CTDP1",HIV Transcription Elongation,69

R-HSA-167172,"ELOA3D,CDK7,CDK9,CDK9,SUPT16H,TAF1L,ELOA3,ERCC2,ERCC3,NCBP2,NELFB,NELFB,GTF2A1,GTF2A2,GTF2B,GTF2E1,GTF2E2,GTF2F1,GTF2F1,GTF2F2,GTF2F2,GTF2H1,GTF2H2,GTF2H3,GTF2H4,GTF2H5,MNAT1,NCBP1,ELOA2,NELFCD,NELFCD,TAF9B,POLR2A,POLR2A,POLR2B,POLR2B,POLR2C,POLR2C,POLR2D,POLR2D,POLR2E,POLR2E,POLR2F,POLR2F,POLR2G,POLR2G,POLR2H,POLR2H,POLR2I,POLR2I,POLR2J,POLR2J,POLR2K,POLR2K,POLR2L,POLR2L,TAF7L,SSRP1,SUPT4H1,SUPT4H1,SUPT5H,SUPT5H,TAF1,TAF2,TAF4,TAF4B,TAF5,TAF6,TAF7,TAF9,TAF10,TAF11,TAF12,TAF13,TBP,TCEA1,ELOC,ELOB,ELOA,ELOA3B,NELFA,NELFA,NELFE,NELFE,TAF15,ELL,TAF3,RNMT,RNGTT,CCNK,CCNH,CCNT1,CCNT1,CCNT2,CTDP1,CTDP1",Transcription of the HIV genome,96

R-HSA-167200,"ELOA3D,CDK7,CDK9,CDK9,SUPT16H,ELOA3,ERCC2,ERCC3,NCBP2,NELFB,NELFB,GTF2F1,GTF2F1,GTF2F2,GTF2F2,GTF2H1,GTF2H2,GTF2H3,GTF2H4,GTF2H5,MNAT1,NCBP1,ELOA2,NELFCD,NELFCD,POLR2A,POLR2A,POLR2B,POLR2B,POLR2C,POLR2C,POLR2D,POLR2D,POLR2E,POLR2E,POLR2F,POLR2F,POLR2G,POLR2G,POLR2H,POLR2H,POLR2I,POLR2I,POLR2J,POLR2J,POLR2K,POLR2K,POLR2L,POLR2L,SSRP1,SUPT4H1,SUPT4H1,SUPT5H,SUPT5H,TCEA1,ELOC,ELOB,ELOA,ELOA3B,NELFA,NELFA,NELFE,NELFE,ELL,CCNH,CCNT1,CCNT1,CTDP1,CTDP1",Formation of HIV-1 elongation complex containing HIV-1 Tat,69

R-HSA-167238,"ELOA3D,CDK9,SUPT16H,ELOA3,NELFB,GTF2F1,GTF2F2,ELOA2,NELFCD,POLR2A,POLR2B,POLR2C,POLR2D,POLR2E,POLR2F,POLR2G,POLR2H,POLR2I,POLR2J,POLR2K,POLR2L,SSRP1,SUPT4H1,SUPT5H,TCEA1,ELOC,ELOB,ELOA,ELOA3B,NELFA,NELFE,ELL,CCNT1,CTDP1",Pausing and recovery of Tat-mediated HIV elongation,34

R-HSA-167242,"NCBP2,NELFB,GTF2F1,GTF2F2,NCBP1,NELFCD,POLR2A,POLR2B,POLR2C,POLR2D,POLR2E,POLR2F,POLR2G,POLR2H,POLR2I,POLR2J,POLR2K,POLR2L,SUPT4H1,SUPT5H,NELFA,NELFE,CTDP1",Abortive elongation of HIV-1 transcript in the absence of Tat,23

R-HSA-167243,"ELOA3D,CDK9,SUPT16H,ELOA3,NELFB,GTF2F1,GTF2F2,ELOA2,NELFCD,POLR2A,POLR2B,POLR2C,POLR2D,POLR2E,POLR2F,POLR2G,POLR2H,POLR2I,POLR2J,POLR2K,POLR2L,SSRP1,SUPT4H1,SUPT5H,TCEA1,ELOC,ELOB,ELOA,ELOA3B,NELFA,NELFE,ELL,CCNT1,CTDP1",Tat-mediated HIV elongation arrest and recovery,34

R-HSA-167246,"ELOA3D,CDK7,CDK9,CDK9,SUPT16H,ELOA3,ERCC2,ERCC3,NCBP2,NELFB,NELFB,GTF2F1,GTF2F1,GTF2F2,GTF2F2,GTF2H1,GTF2H2,GTF2H3,GTF2H4,GTF2H5,MNAT1,NCBP1,ELOA2,NELFCD,NELFCD,POLR2A,POLR2A,POLR2B,POLR2B,POLR2C,POLR2C,POLR2D,POLR2D,POLR2E,POLR2E,POLR2F,POLR2F,POLR2G,POLR2G,POLR2H,POLR2H,POLR2I,POLR2I,POLR2J,POLR2J,POLR2K,POLR2K,POLR2L,POLR2L,SSRP1,SUPT4H1,SUPT4H1,SUPT5H,SUPT5H,TCEA1,ELOC,ELOB,ELOA,ELOA3B,NELFA,NELFA,NELFE,NELFE,ELL,CCNH,CCNT1,CCNT1,CTDP1,CTDP1",Tat-mediated elongation of the HIV-1 transcript,69

R-HSA-167287,"ELOA3D,CDK9,SUPT16H,ELOA3,NELFB,GTF2F1,GTF2F2,ELOA2,NELFCD,POLR2A,POLR2B,POLR2C,POLR2D,POLR2E,POLR2F,POLR2G,POLR2H,POLR2I,POLR2J,POLR2K,POLR2L,SSRP1,SUPT4H1,SUPT5H,TCEA1,ELOC,ELOB,ELOA,ELOA3B,NELFA,NELFE,ELL,CCNK,CCNT1,CCNT2,CTDP1",HIV elongation arrest and recovery,36

R-HSA-167290,"ELOA3D,CDK9,SUPT16H,ELOA3,NELFB,GTF2F1,GTF2F2,ELOA2,NELFCD,POLR2A,POLR2B,POLR2C,POLR2D,POLR2E,POLR2F,POLR2G,POLR2H,POLR2I,POLR2J,POLR2K,POLR2L,SSRP1,SUPT4H1,SUPT5H,TCEA1,ELOC,ELOB,ELOA,ELOA3B,NELFA,NELFE,ELL,CCNK,CCNT1,CCNT2,CTDP1",Pausing and recovery of HIV elongation,36

R-HSA-167590,"AP2M1,AP2S1,AP2A1,AP2A2,AP2B1,ARF1,LCK,ATP6V1H,CD4",Nef Mediated CD4 Down-regulation,9

R-HSA-167826,"UCP1,UCP2,UCP3,SLC25A14,SLC25A27",The fatty acid cycling model,5

R-HSA-167827,"UCP1,UCP2,UCP3,SLC25A14,SLC25A27",The proton buffering model,5

R-HSA-1679131,"CNPY3,CTSB,CTSK,CTSL,CTSV,CTSS,TLR7,TLR8,TLR9,LGMN,TLR3,HSP90B1,UNC93B1",Trafficking and processing of endosomal TLR,13

R-HSA-168138,"TMED7-TICAM2,NOD1,TAB1,CHUK,MAP3K8,MAP3K8,CREB1,ATF2,MAPK14,TICAM1,AGER,DUSP3,DUSP4,DUSP6,DUSP7,ELK1,TAB2,FBXW11,FOS,LY96,PELI3,TAB3,NKIRAS2,NKIRAS1,PIK3R4,HMGB1,APP,TICAM2,IKBKB,IRAK1,IRAK2,IRF7,JUN,TMEM189-UBE2V1,MEF2A,MEF2C,MAP3K1,MYD88,ATF1,NFKB1,NFKB2,NFKBIA,NFKBIB,IRAK4,VRK3,TLR7,ECSIT,TLR8,PIK3C3,TLR9,PPP2CA,PPP2CB,PPP2R1A,PPP2R1B,PPP2R5D,MAPK1,MAPK1,MAPK3,MAPK3,MAPK7,MAPK7,MAPK8,MAPK11,MAPK9,MAPK10,MAP2K1,MAP2K3,MAP2K6,MAP2K7,PELI2,PELI1,RELA,RPS6KA1,RPS6KA2,RPS6KA3,RPS27A,S100A12,S100B,SAA1,NOD2,RBSN,MAP2K4,MAP2K4,SKP1,MAP3K7,TLR4,TRAF6,UBA52,UBB,UBC,UBE2N,UBE2V1,MAPKAPK3,TNIP2,EEA1,CUL1,IKBKG,RIPK2,BTRC,RPS6KA5,MAPKAPK2,CD14",Toll Like Receptor 9 (TLR9) Cascade,102

R-HSA-168142,"NOD1,TAB1,CHUK,MAP3K8,MAP3K8,CREB1,ATF2,MAPK14,AGER,DUSP3,DUSP4,DUSP6,DUSP7,ELK1,TAB2,FBXW11,FOS,PELI3,TAB3,NKIRAS2,NKIRAS1,HMGB1,APP,IKBKB,IRAK1,IRAK2,JUN,TMEM189-UBE2V1,MEF2A,MEF2C,MAP3K1,MYD88,ATF1,NFKB1,NFKB2,NFKBIA,NFKBIB,IRAK4,VRK3,ECSIT,PPP2CA,PPP2CB,PPP2R1A,PPP2R1B,PPP2R5D,MAPK1,MAPK1,MAPK3,MAPK3,MAPK7,MAPK7,MAPK8,MAPK11,MAPK9,MAPK10,MAP2K1,MAP2K3,MAP2K6,MAP2K7,PELI2,PELI1,RELA,RPS6KA1,RPS6KA2,RPS6KA3,RPS27A,S100A12,S100B,SAA1,NOD2,MAP2K4,MAP2K4,SKP1,MAP3K7,TLR5,TRAF6,UBA52,UBB,UBC,UBE2N,UBE2V1,MAPKAPK3,TNIP2,TLR10,CUL1,IKBKG,RIPK2,BTRC,RPS6KA5,MAPKAPK2",Toll Like Receptor 10 (TLR10) Cascade,90

R-HSA-168164,"TANK,NOD1,TAB1,RIPK3,CHUK,MAP3K8,MAP3K8,CREB1,ATF2,MAPK14,TICAM1,AGER,DUSP3,DUSP4,DUSP6,DUSP7,ELK1,SARM1,TAB2,FBXW11,FOS,TAB3,NKIRAS2,NKIRAS1,TBK1,HMGB1,BIRC2,BIRC3,APP,IKBKB,IRAK1,IRAK2,IRF3,IRF7,JUN,TMEM189-UBE2V1,MEF2A,MEF2C,ATF1,NFKB1,NFKB2,NFKBIA,NFKBIB,VRK3,PPP2CA,PPP2CB,PPP2R1A,PPP2R1B,PPP2R5D,MAPK1,MAPK1,MAPK3,MAPK3,MAPK7,MAPK7,MAPK8,MAPK11,MAPK9,MAPK10,MAP2K1,MAP2K3,MAP2K6,MAP2K7,RELA,RPS6KA1,RPS6KA2,RPS6KA3,RPS27A,S100A12,S100B,SAA1,NOD2,MAP2K4,MAP2K4,SKP1,MAP3K7,TLR3,TRAF3,TRAF6,UBA52,UBB,UBC,UBE2D1,UBE2D2,UBE2D3,UBE2N,UBE2V1,MAPKAPK3,TNIP2,CASP8,CUL1,IKBKG,RIPK1,RIPK2,FADD,BTRC,RPS6KA5,MAPKAPK2,IKBKE",Toll Like Receptor 3 (TLR3) Cascade,99

R-HSA-168176,"NOD1,TAB1,CHUK,MAP3K8,MAP3K8,CREB1,ATF2,MAPK14,AGER,DUSP3,DUSP4,DUSP6,DUSP7,ELK1,TAB2,FBXW11,FOS,PELI3,TAB3,NKIRAS2,NKIRAS1,HMGB1,APP,IKBKB,IRAK1,IRAK2,JUN,TMEM189-UBE2V1,MEF2A,MEF2C,MAP3K1,MYD88,ATF1,NFKB1,NFKB2,NFKBIA,NFKBIB,IRAK4,VRK3,ECSIT,PPP2CA,PPP2CB,PPP2R1A,PPP2R1B,PPP2R5D,MAPK1,MAPK1,MAPK3,MAPK3,MAPK7,MAPK7,MAPK8,MAPK11,MAPK9,MAPK10,MAP2K1,MAP2K3,MAP2K6,MAP2K7,PELI2,PELI1,RELA,RPS6KA1,RPS6KA2,RPS6KA3,RPS27A,S100A12,S100B,SAA1,NOD2,MAP2K4,MAP2K4,SKP1,MAP3K7,TLR5,TRAF6,UBA52,UBB,UBC,UBE2N,UBE2V1,MAPKAPK3,TNIP2,TLR10,CUL1,IKBKG,RIPK2,BTRC,RPS6KA5,MAPKAPK2",Toll Like Receptor 5 (TLR5) Cascade,90

R-HSA-168179,"TLR6,NOD1,TAB1,IRAK3,TIRAP,CHUK,MAP3K8,MAP3K8,CREB1,ATF2,MAPK14,AGER,DUSP3,DUSP4,DUSP6,DUSP7,ELK1,TAB2,FBXW11,FOS,LY96,PELI3,TAB3,NKIRAS2,NKIRAS1,HMGB1,APP,IKBKB,IRAK1,IRAK2,JUN,TMEM189-UBE2V1,MEF2A,MEF2C,MAP3K1,MYD88,ATF1,NFKB1,NFKB2,NFKBIA,NFKBIB,IRAK4,VRK3,ECSIT,PPP2CA,PPP2CB,PPP2R1A,PPP2R1B,PPP2R5D,MAPK1,MAPK1,MAPK3,MAPK3,MAPK7,MAPK7,MAPK8,MAPK11,MAPK9,MAPK10,MAP2K1,MAP2K3,MAP2K6,MAP2K7,PELI2,PELI1,SIGIRR,RELA,RPS6KA1,RPS6KA2,RPS6KA3,RPS27A,S100A12,S100B,SAA1,NOD2,MAP2K4,MAP2K4,SFTPD,SKP1,SFTPA1,MAP3K7,BTK,TLR1,TLR2,TLR4,TRAF6,SFTPA2,UBA52,UBB,UBC,UBE2N,UBE2V1,MAPKAPK3,TNIP2,CUL1,IKBKG,SOCS1,RIPK2,BTRC,RPS6KA5,MAPKAPK2,CD14,CD36",Toll Like Receptor TLR1:TLR2 Cascade,103

R-HSA-168181,"TMED7-TICAM2,NOD1,TAB1,CHUK,MAP3K8,MAP3K8,CREB1,ATF2,MAPK14,TICAM1,AGER,DUSP3,DUSP4,DUSP6,DUSP7,ELK1,TAB2,FBXW11,FOS,LY96,PELI3,TAB3,NKIRAS2,NKIRAS1,HMGB1,APP,TICAM2,IKBKB,IRAK1,IRAK2,IRF7,JUN,TMEM189-UBE2V1,MEF2A,MEF2C,MAP3K1,MYD88,ATF1,NFKB1,NFKB2,NFKBIA,NFKBIB,IRAK4,VRK3,TLR7,ECSIT,TLR8,TLR9,PPP2CA,PPP2CB,PPP2R1A,PPP2R1B,PPP2R5D,MAPK1,MAPK1,MAPK3,MAPK3,MAPK7,MAPK7,MAPK8,MAPK11,MAPK9,MAPK10,MAP2K1,MAP2K3,MAP2K6,MAP2K7,PELI2,PELI1,RELA,RPS6KA1,RPS6KA2,RPS6KA3,RPS27A,S100A12,S100B,SAA1,NOD2,MAP2K4,MAP2K4,SKP1,MAP3K7,TLR4,TRAF6,UBA52,UBB,UBC,UBE2N,UBE2V1,MAPKAPK3,TNIP2,CUL1,IKBKG,RIPK2,BTRC,RPS6KA5,MAPKAPK2,CD14",Toll Like Receptor 7/8 (TLR7/8) Cascade,98

R-HSA-168188,"TLR6,NOD1,TAB1,IRAK3,TIRAP,CHUK,MAP3K8,MAP3K8,CREB1,ATF2,MAPK14,AGER,DUSP3,DUSP4,DUSP6,DUSP7,ELK1,TAB2,FBXW11,FOS,LY96,PELI3,TAB3,NKIRAS2,NKIRAS1,HMGB1,APP,IKBKB,IRAK1,IRAK2,JUN,TMEM189-UBE2V1,MEF2A,MEF2C,MAP3K1,MYD88,ATF1,NFKB1,NFKB2,NFKBIA,NFKBIB,IRAK4,VRK3,ECSIT,PPP2CA,PPP2CB,PPP2R1A,PPP2R1B,PPP2R5D,MAPK1,MAPK1,MAPK3,MAPK3,MAPK7,MAPK7,MAPK8,MAPK11,MAPK9,MAPK10,MAP2K1,MAP2K3,MAP2K6,MAP2K7,PELI2,PELI1,SIGIRR,RELA,RPS6KA1,RPS6KA2,RPS6KA3,RPS27A,S100A12,S100B,SAA1,NOD2,MAP2K4,MAP2K4,SKP1,MAP3K7,BTK,TLR1,TLR2,TLR4,TRAF6,UBA52,UBB,UBC,UBE2N,UBE2V1,MAPKAPK3,TNIP2,CUL1,IKBKG,SOCS1,RIPK2,BTRC,RPS6KA5,MAPKAPK2,CD14,CD36",Toll Like Receptor TLR6:TLR2 Cascade,100

R-HSA-168249,"A1BG,SIGLEC14,ABI1,ABI1,TANK,KIR2DS2,DEFB130B,DEFB4B,C4B_2,TMED7-TICAM2,TOM1,EPPIN-WFDC6,KLRC4-KLRK1,KLRC4-KLRK1,MUC12,HUWE1,ARPC5,ARPC5,ARPC4,ARPC4,ARPC3,ARPC3,ARPC1B,ARPC1B,ACTR3,ACTR3,ACTR2,ACTR2,ADAM8,ARPC2,ARPC2,ACTR1B,RASGRP1,ABI2,ABI2,ATP6AP2,WASF2,WASF2,PSME3,ADAM10,PSMD14,RASGRP2,LOC102725035,TCIRG1,CRISP3,SIRPB1,TLR6,TUBB4B,NOD1,NOD1,PRG3,ATP8A1,PPIE,VAV3,VAV3,TAB1,BAIAP2,BAIAP2,CLEC10A,CAP1,VAT1,ATG7,PRDX4,ARPC1A,ARPC1A,AGPAT2,OLFM4,CCT2,NPC2,GNLY,COLEC10,POLR3F,POLR3G,POLR3C,TXNIP,TXNIP,CCT8,CNPY3,CFL1,MASP2,CTSC,AHCYL1,NCKAP1,NCKAP1,IQGAP2,LOC107987462,WASF3,WASF3,CEACAM3,HPSE,PGRMC1,FGL2,CFHR4,CFHR3,CEACAM8,RAB10,MALT1,MALT1,SUGT1,CKAP4,SLC27A2,GLIPR1,LILRA3,RAB31,RIPK3,POLR3A,CHGA,CHI3L1,CHIT1,IRAK3,PADI2,TREX1,CD300A,TMC6,PDAP1,CHRNB4,NLRP3,NLRP3,TIRAP,CHUK,PGLYRP2,PGLYRP3,STK11IP,CGAS,RASGRP4,LEAP2,LRG1,DCD,DEFB118,MUCL1,CLU,SERPINA3,SLC15A4,PLD4,RNASE8,PSMB11,CCR6,CANT1,CD300LB,OSCAR,CNN2,ATP6V1G3,ARL8A,BPIFB6,REG3G,COPB1,MAP3K8,MAP3K8,CPB2,CPN1,CPN2,CR1,CR2,HGSNAT,CREB1,ATF2,CREBBP,CRK,AAMP,CRP,MUC17,DEFB104A,BPIFA2,DEFB127,DEFB129,SIRPA,MAPK14,PSMA8,MUC15,CSNK2B,CST3,WIPF2,WIPF2,CSTB,NLRP4,TICAM1,CTNNB1,BPIFB4,NFAM1,CTSB,CTSD,CTSG,CTSH,CTSK,CTSL,CTSV,CTSS,CTSZ,CYBA,CYBB,CYLD,ABCA13,ATP6V0E2,MOSPD2,CLEC12A,CD55,AP2A2,DDOST,DDOST,DDX3X,DHX9,DEFA1,DEFA3,DEFA4,DEFA5,DEFA6,DEFB1,DEFB4A,CFD,CLEC4C,DHX36,POLR3H,CYB5R3,DIAPH1,AGA,DNM1,AGER,AGER,DNASE1L1,DYNC1H1,AGL,DNM2,DOCK1,DOCK2,DSC1,DSG1,DSP,DUSP3,DUSP4,DUSP6,DUSP7,EEF1A1,EEF2,PLPP4,ANO6,AHSG,NLRC3,ELANE,SERPINB1,MCEMP1,ELK1,MUC20,UNC13D,TUBB,EP300,STOM,ALAD,F2,FABP5,FCAR,FCER1A,FCER1A,MS4A2,MS4A2,FCER1G,FCER1G,FCGR1A,FCGR1A,ALDH3B1,FCGR2A,FCGR2A,FCGR3A,FCGR3A,FCGR3B,FCN1,FCN2,ADGRG3,FGA,FGB,ALDOA,FGG,FGR,NLRP1,ENPP4,KLRK1,KLRK1,CD93,RAB18,TRIM32,ALDOC,ERP44,SARM1,NFASC,TAB2,CYFIP1,CYFIP1,FAF2,PSME4,ATP11B,NBEAL2,DTX4,ATP11A,FBXW11,DNAJC13,UBR4,NCSTN,COTL1,COMMD3,FOLR3,ARHGAP45,FOS,ATP6V0A2,FPR1,FPR2,DDX58,DDX58,HEBP2,CLEC5A,LY96,PLD3,ALOX5,PANX1,FRK,DEFB105A,DEFB106A,DEFB107A,DEFB108B,DEFB110,DEFB113,DEFB114,DEFB115,DEFB116,DEFB119,DEFB121,DEFB123,DEFB124,DEFB125,DEFB128,DEFB130A,ATP6V0D2,ATP6V1C2,PELI3,FTH1,ABL1,FTL,FUCA1,FUCA2,FYN,FYN,XRCC6,GAA,TAB3,QPCT,BRI3,GCA,SVIP,METTL7A,ARMC8,GALNS,AOC1,TKFC,DNM3,CLEC4E,CLEC4E,OSTF1,GDI2,B4GALT1,CYFIP2,CYFIP2,LAT,LAT,GLA,SIGLEC9,AMPD3,GLB1,C5AR2,CRCP,GM2A,GNS,GPI,MUC19,SIGLEC15,NKIRAS2,NKIRAS1,GRB2,GRB2,LAMTOR2,GRN,DBNL,ANPEP,COMMD9,PYCARD,PYCARD,TBK1,TBK1,CXCL1,GSN,GSTP1,GUSB,GYG1,DPP7,ACAA1,GZMM,ANXA2,HBB,HCK,NCKAP1L,NCKAP1L,HEXB,CFH,CFHR1,CFHR2,CD209,PIK3R4,UBE2K,HK3,HLA-A,HLA-B,HLA-C,HLA-E,HLA-E,HMGB1,HMGB1,HMOX2,APAF1,HP,HRAS,RAB37,APEH,BIRC2,BIRC3,HSPA1A,HSPA1B,HSPA6,HSPA8,HSP90AA1,HSP90AB1,HTN1,HTN3,APOB,S100A7A,CLEC4D,ICAM2,ICAM3,STING1,IDH1,CD300E,CFI,IFI16,IFNA1,IFNA2,IFNA4,IFNA5,IFNA6,IFNA7,IFNA8,IFNA10,IFNA13,IFNA14,IFNA16,IFNA17,IFNA21,IFNB1,IGF2R,APP,APP,APRT,SLCO4C1,TICAM2,IKBKB,IL1B,CXCR1,CXCR2,ILF2,IMPDH1,IMPDH2,IRAK1,IRAK2,IRF3,IRF3,IRF7,ITGAL,ITGAM,ITGAV,ITGAX,ITGB2,ITK,ITK,ITPR1,ITPR2,ITPR3,JUN,JUP,TMEM179B,TBC1D10C,KIR2DS1,KIR2DS4,KIR2DS5,KIR3DS1,KLRC2,KLRC2,KLRD1,KLRD1,ARG1,KPNB1,KRAS,KRT1,RHOA,C6orf120,TMEM189-UBE2V1,NHLRC3,HRNR,FLG2,LAIR1,RHOG,LAMP1,LAMP2,LBP,LCK,LCK,LCN2,LCP2,LCP2,MUC21,LGALS3,LGALS3,LIMK1,LIMK1,DEFB132,RAB44,LPO,LRMP,LTA4H,LTF,CD180,LYN,LYN,LYZ,ARSA,ARSB,MAN2B1,MANBA,DEFB103A,MBL2,ART1,CD46,MEF2A,MEF2C,MEFV,MAP3K1,MAP3K1,MGST1,ASAH1,MIF,MME,MMP8,MMP9,MNDA,MPO,MRE11,TARM1,MUC1,MUC3A,MUC4,MUC5AC,MUC6,MUC7,MYD88,MYH2,MYH9,MYO1C,MYO5A,MYO9B,MYO10,ATF1,CEACAM6,NCF2,NCF4,NCK1,NCK1,ACLY,NDUFC2,ATOX1,NEU1,NF2,NFATC1,NFATC2,NFATC3,NFKB1,NFKB2,NFKBIA,NFKBIB,NME2,NOS1,NOS2,NOS3,PNP,NRAS,OLR1,ORM1,ORM2,P2RX1,P2RX7,P2RX7,PA2G4,DEFB107B,DEFB104B,DEFB106B,DEFB105B,PAFAH1B2,PAK1,PAK1,ATP6V0A4,PAK2,PAK2,PAK3,REG3A,CLEC4A,PCBP2,DERA,POLR1D,GOLGA7,IRAK4,DYNC1LI1,HERC5,RAB9B,VRK3,PDZD11,TLR7,ECSIT,BPIFA1,TLR8,PLAC8,ATP6V1D,BIN2,DDX41,NCKIPSD,NCKIPSD,RAB14,ATP6V1H,YPEL5,PDPK1,CAB39,POLR3K,PECAM1,CALML5,ADA2,CFP,PFKL,PGAM1,ATP6V1A,PGM1,ATP6V1B1,ATP6V1B2,SERPINA1,PI3,SERPINB6,ATP6V0C,SERPINB10,ATP6V1C1,PIGR,PIK3C3,ATP6V1E1,PIK3CA,PIK3CB,PIK3R1,PIK3R2,PIN1,PKM,PKP1,PLA2G2A,PLAU,PLAUR,ATP6V0B,PLCG1,PLCG1,PLCG2,PLCG2,PLD1,PLD2,ATP6V1G2,ATP6V0A1,ATP7A,GPR84,RAB4B,RAB24,TLR9,TREM2,TREM2,TREM1,POLR2E,POLR2F,POLR2H,POLR2K,POLR2L,TOLLIP,RNF216,RHOF,PPBP,CTSA,PPIA,CMTM6,RNF125,ACP3,LAMTOR1,PPP2CA,PPP2CB,PPP2R1A,PPP2R1B,PGM2,PPP2R5D,PPP3CA,CPPED1,PPP3CB,PPP3R1,PRCP,PRG2,OTUD5,ITLN1,PRKACA,PRKACB,PRKACG,POLR3B,POLR3E,TMEM30A,PRKCD,PRKCE,CAND1,BRK1,BRK1,ACTR10,PRKCQ,PRKCQ,PRKCSH,DEFB103B,PRKDC,MAPK1,MAPK1,MAPK3,MAPK3,MAPK7,MAPK7,MAPK8,MAPK11,MAPK9,MAPK10,MAPK13,MAP2K1,MAP2K3,MAP2K6,MAP2K7,DNAJC3,PROS1,LGMN,PRSS2,PRSS3,MASP1,PRTN3,AZU1,PSAP,PSEN1,MUC13,B2M,B2M,RETN,PSMA1,PSMA2,PSMA3,PSMA4,PSMA5,PSMA6,PSMA7,KCMF1,PSMB1,PSMB2,PSMB3,PSMB4,PSMB5,PSMB6,PSMB7,NIT2,PSMB8,PSMB9,PSMB10,PSMC1,PSMC2,PSMC3,VPS35L,PSMC4,PSMC5,PSMC6,PSMD1,PSMD2,PSMD3,PSMD4,PSMD5,PGLYRP4,EPPIN,CD177,PSMD7,PSMD8,PSMD9,SLC44A2,PSMD10,PELI2,PELI1,PSMD11,PSMD12,PSMD13,PSME1,PSME2,PTAFR,PTK2,MAVS,MAVS,QSOX1,PTPN4,PTPN6,PTPN11,RAP2C,PTPRB,PTPRC,PTPRJ,PTPRN2,PTX3,PYGB,PYGL,NLRC4,NLRC4,TRAPPC1,RAB3A,RAB5B,RAB6A,RAB27A,RAB5C,RAC1,RAC1,RAC2,RAF1,RAP1A,RAP1B,RAP2B,SIGIRR,BCL2,RELA,RELB,BCL2L1,ACTB,ACTB,RNASE2,RNASE3,RNASE6,ROCK1,DEFB135,DEFB136,DEFB134,RPS6KA1,RPS6KA2,RPS6KA3,RPS27A,S100A1,S100A7,S100A8,S100A9,S100A11,S100A12,S100A12,S100B,S100B,S100P,SAA1,SAA1,CFB,MAPK12,SERPINB3,CEACAM1,SDCBP,ELMO2,SELL,SEMG1,TMBIM1,NOD2,NOD2,IFIH1,IFIH1,RBSN,MAP2K4,MAP2K4,CARD9,CARD9,ARHGAP9,MMP25,SFTPD,WIPF3,WIPF3,DEFB131A,CLEC7A,CLEC7A,SHC1,SHC1,SKP1,SLC2A3,SLC2A5,NCF1,SFTPA1,NME1-NME2,SLC11A1,SLPI,POLR3D,SNAP25,SOS1,SOS1,SPTAN1,BPI,SRC,SRP14,TRIM21,STAT6,STK10,BST1,SURF4,BST2,SYK,SYK,MAP3K7,TCN1,BTK,BTK,DYNLT1,TEC,TEC,TIMP2,TLR1,TLR2,TLR3,TLR4,ACTG1,ACTG1,SERPING1,TLR5,C1QA,TNFAIP3,C1QB,TNFAIP6,TNFRSF1B,C1QC,C1R,C1S,C2,C3,HSP90B1,TRAF2,TRAF2,TRAF3,TRAF6,TRAF6,C3AR1,C4A,C4B,C4BPA,TRPM2,C4BPB,C5,TTR,MUC5B,C5AR1,DEFA1B,C6,CCR2,SFTPA2,TXK,TXK,TXN,TXN,C7,TYROBP,TYROBP,C8A,UBA52,UBB,UBC,UBA7,C8B,UBE2D1,UBE2D1,UBE2D2,UBE2D2,UBE2D3,UBE2D3,C8G,UBE2N,UBE2V1,C9,VAV1,VAV1,VAV2,VAV2,VCL,VCP,VTN,WAS,WAS,WIPF1,WIPF1,LAT2,LAT2,XRCC5,YES1,TRIM25,TRIM25,MAPKAPK3,RAB7A,COLEC11,DHX58,TNIP2,C1orf35,NLRX1,SEM1,GSDMD,LPCAT1,ATP8B4,DOK3,DSN1,CALM1,CALM1,PTGES2,SIKE1,CEP290,ALPK1,PLEKHO2,DNAJC5,BPIFB2,CALM2,CALM2,CALM3,CALM3,ZBP1,ZBP1,CFHR5,TXNDC5,TSPAN14,UNC93B1,DEFB126,TLR10,TRIM56,CAMP,CAPN1,EPX,CAPZA1,CAPZA2,CASP1,CASP1,CASP2,CASP4,CRISPLD2,ITCH,ATAD3B,PLA2G6,MAGT1,CASP8,EEA1,NLRC5,CASP9,POLR3GL,RNF135,RNF135,CASP10,HVCN1,CYSTM1,CARD11,CARD11,FRMPD3,PLPP5,GHDC,CUL1,ADGRE3,RNASE7,CAT,CRACR2A,KCNAB2,IKBKG,FCN3,DEGS1,PDXK,CDK13,RNASET2,LAMTOR3,SOCS1,DYNLL1,VAMP8,DGAT1,RIPK1,RIPK2,RIPK2,FADD,SNAP23,SIGLEC5,CREG1,IQGAP1,GGH,VNN1,TAX1BP1,CPNE3,CPNE1,AP1M1,BCL10,BCL10,WASF1,WASF1,BTRC,MGAM,WASL,WASL,SERPINB12,STBD1,ATP6V0E1,PGLYRP1,MAP3K14,UBA3,UBE2M,ATP6V1E2,PSTPIP1,ATP6V0D1,ATG12,SYNGR1,CD3G,CD3G,CD247,CD247,CD4,LRRFIP1,VAPA,UBE2L6,UBE2L6,RPS6KA5,MAPKAPK2,TIFA,BPIFB1,CD14,ATP6V1F,CD19,NAPRT,MS4A3,SNAP29,CLEC6A,CLEC6A,GRAP2,GRAP2,MUC16,ORMDL3,NCR2,AIM2,AIM2,CD33,LY86,ATG5,CD36,PSMF1,SCAMP1,POLR1C,GMFG,RAB3D,ATP6V1G1,PRDX6,CD44,CD47,CD53,ISG15,IKBKE,CD58,CD59,CD63,CD68,TMEM63A,CD81,ADGRE5,MLEC,CDA,IST1,ELMO1,GAB2,GAB2,PSMD6,MVP,CDC34,CDC42,CDC42",Innate Immune System,1177

R-HSA-168255,"NUP50,CPSF4,NUP42,RPL35,RPL39L,CLTA,CLTC,NUP35,RPS4Y2,RPL10L,PARP1,RPL22L1,NUP205,NUP210,NUP160,NUP188,RPL13A,NUP62,RPL36,GRSF1,SLC25A6,GTF2F1,GTF2F2,HSPA1A,HSPA1B,HSP90AA1,NUP43,KPNA1,KPNB1,KPNA2,KPNA3,KPNA4,KPNA5,IPO5,RPSA,KPNA7,RPL10A,NUP88,NUP98,RPS27L,RPL26L1,NUP54,POLR2A,POLR2B,POLR2C,POLR2D,POLR2E,POLR2F,POLR2G,POLR2H,POLR2I,POLR2J,POLR2K,POLR2L,NDC1,NUP133,EIF2AK2,DNAJC3,NUP107,RAN,RANBP2,RPL3,RPL3L,RPL4,RPL5,RPL6,RPL7,RPL7A,RPL8,RPL9,RPL10,RPL11,RPL12,RPL13,RPL15,RPL17,RPL18,RPL18A,RPL19,RPL21,RPL22,RPL23A,RPL24,RPL26,RPL27,RPL30,RPL27A,RPL28,RPL29,RPL31,RPL32,RPL34,RPL35A,RPL36AL,RPL37,RPL37A,RPL38,RPL39,RPL41,RPL36A,RPLP0,RPLP1,RPLP2,RPS2,RPS3,RPS3A,RPS4X,RPS4Y1,RPS5,RPS6,RPS7,RPS8,RPS9,RPS10,RPS11,RPS12,RPS13,RPS14,RPS15,RPS15A,RPS16,RPS17,RPS18,RPS19,RPS20,RPS21,RPS23,RPS24,RPS25,RPS26,RPS27,RPS27A,RPS28,RPS29,SEC13,TGFB1,TPR,UBA52,XPO1,NUP37,NUP85,NUP214,AAAS,PABPN1,CALR,SEH1L,CANX,RAE1,RPL14,RPL23,NUP155,ISG15,NUP93,NUP58,POM121,NUP153",Influenza Infection,156

R-HSA-168256,"A1BG,AKT3,SIGLEC14,ABI1,ABI1,TANK,CD300LD,KIR2DS2,DEFB130B,DEFB4B,C4B_2,GPR75-ASB3,TMED7-TICAM2,TOM1,RANBP9,MICA,EPPIN-WFDC6,KLRC4-KLRK1,KLRC4-KLRK1,CHURC1-FNTB,AP1M2,IL18BP,MUC12,HUWE1,ARPC5,ARPC5,ARPC4,ARPC4,ARPC3,ARPC3,ARPC1B,ARPC1B,ACTR3,ACTR3,ACTR2,ACTR2,ADAM8,TRIM10,ARPC2,ARPC2,KIF20A,ACTR1B,ACTR1A,RASGRP1,RASGRP1,BCAP31,EBI3,ABI2,ABI2,RASA4,ATP6AP2,WASF2,WASF2,RNF41,PSME3,PSME3,ADAM10,PSMD14,PSMD14,KLRG1,CD96,RASGRP2,RASGRP2,CNKSR1,CDKN1A,LOC102725035,STUB1,TRAIP,ADAR,TCIRG1,CRISP3,KLHL41,SIRPB1,TLR6,TRIM22,TUBA1B,IRF9,TUBB3,TUBB4A,TUBB4B,BTN3A3,BTN2A2,NOD1,NOD1,ANAPC10,PRG3,ATP8A1,IFITM3,RAPGEF3,UBAC1,ARIH2,SEC24B,LYPLA1,IFI30,PPIE,VAV3,VAV3,TAB1,BAIAP2,BAIAP2,CLEC10A,TRIM38,UBE2E3,SEC23A,CAP1,LRRC41,VAT1,FBXW10,CEBPD,ATG7,BATF,DCTN2,PRDX4,ARPC1A,ARPC1A,AGPAT2,OLFM4,CCT2,NPC2,GNLY,IFITM2,COLEC10,TRIM3,RBCK1,CENPE,POLR3F,POLR3G,POLR3C,TXNIP,TXNIP,CD226,DCTN6,TNFSF13B,CCT8,CNPY3,NRG3,CFL1,CFL1,MASP2,CTSC,NUP50,AHCYL1,NCKAP1,NCKAP1,IQGAP2,LOC107987462,SEC24A,WASF3,WASF3,FRS3,FRS2,CEACAM3,HPSE,PGRMC1,LILRB1,HCST,CD300C,FGL2,CFHR4,CFHR3,CEACAM8,RAB10,MALT1,MALT1,SUGT1,EDAR,SEC61B,CKAP4,LILRB5,SLC27A2,KIF2C,LILRB4,IL24,GLIPR1,LILRA1,LILRA3,LILRA2,RAB31,RIPK3,WWP1,UBE2C,RAPGEF4,TRIM31,NUP42,BTN3A2,BTN3A1,BTN2A1,CD160,KIF3A,POLR3A,CHGA,IL1RAPL1,GLMN,CHI3L1,CHIT1,IRAK3,DUSP10,PADI2,DCTN3,TUBA3E,USP18,IL17F,KLHL2,TREX1,CD300A,TMC6,PDAP1,TUBA3D,TRIM9,SIGLEC11,CHRNB4,NLRP3,NLRP3,TIRAP,CHUK,CHUK,PGLYRP2,PGLYRP3,STK11IP,SLAMF6,OSBPL1A,FBXO32,CGAS,FBXO17,GBP4,GBP5,CISH,CISH,TNFRSF13C,RASGRP4,RASGRP4,IL22RA2,LEAP2,LRG1,THEM4,SH2D1B,DCD,DEFB118,AP2M1,AP1S1,AP2S1,TRIM6,UBE2J2,MUCL1,PIK3AP1,PIK3AP1,CLU,SERPINA3,JAML,CLTA,SLC15A4,CLTC,PLD4,RNASE8,PSMB11,PSMB11,LRR1,CCR1,CCR5,CCR6,CANT1,CD300LB,OSCAR,FBXO27,CNN2,H3C14,H3C14,CNTF,CNTFR,ATP6V1G3,ASB17,RNF19B,COL1A1,COL1A2,ARL8A,COL2A1,COL3A1,EDARADD,BPIFB6,NUP35,REG3G,AP1S3,COL17A1,TRIM71,CD200R1,COPB1,IL17RE,MAP3K8,MAP3K8,IL31RA,RAET1E,TRIM50,CPB2,ASB10,CPN1,CPN2,CR1,CR2,HGSNAT,CREB1,ATF2,CREBBP,CRK,CRKL,AAMP,CRP,MUC17,ASB11,ASB5,ASB6,ASB7,ASB8,ASB9,DEFB104A,BPIFA2,TRIM69,DYNLL2,UBE2F,DEFB127,DEFB129,SIRPA,MIB2,ASB15,ASB14,ASB12,MAPK14,HECTD2,PSMA8,PSMA8,CSF1,CSF1R,MUC15,CSF2,CSF2RA,CSF2RB,CSF2RB,CSF3,CSF3R,CSH1,CSH1,CSK,FBXL14,NRG4,CSNK2B,FBXL16,IL34,CD300LF,CD300LG,CST3,WIPF2,WIPF2,CSTB,KLC3,NLRP4,TICAM1,UBE2U,CTF1,IL23R,IL23R,CTLA4,CTNNB1,BPIFB4,NFAM1,FBXO41,CTSB,CTSD,CTSE,CTSG,CTSH,CTSK,CTSL,CTSL,CTSV,CTSV,DTX3L,BTLA,BTLA,CTSO,CTSS,CTSS,CTSZ,CXADR,PAQR3,KLB,DAB2IP,CYBA,BTNL9,CYBB,CYLD,RNF217,ABCA13,KCTD7,ATP6V0E2,CBLL2,MOSPD2,AP2A1,CLEC12A,CD55,AP2A2,SPRED1,AP1B1,AP2B1,GBP6,IFNLR1,DCTN1,AP1G1,DDOST,DDOST,DDX3X,DHX9,DEFA1,DEFA3,DEFA4,DEFA5,DEFA6,DEFB1,DEFB4A,CFD,CLEC4C,DHX36,POLR3H,CYB5R3,DIAPH1,DLG1,DLG2,DLG3,DLG4,AGA,DNM1,AGER,AGER,DNASE1L1,DYNC1H1,AGL,DYNC1I1,DYNC1I2,DYNC1LI2,DNM2,DOCK1,DOCK2,DSC1,DSG1,DSP,HBEGF,DUSP1,DUSP2,DUSP3,DUSP4,DUSP5,DUSP6,DUSP7,DUSP8,DUSP9,EDA,S1PR1,EEF1A1,EEF2,EGF,EGFR,EGR1,PLPP4,PIANP,ANO6,AHSG,UBR1,EIF4A1,NLRC3,EIF4A2,EIF4E,EIF4G1,EIF4G2,ELANE,SERPINB1,MCEMP1,TXLNA,ELK1,SPRED2,KCTD6,MUC20,UNC13D,FBXO15,PDE12,TUBB,EP300,STOM,ERBB2,ERBB3,ERBB4,EREG,AKT1,AKT2,AKT2,ALAD,F2,F13A1,FABP5,PTK2B,FCAR,FCER1A,FCER1A,MS4A2,MS4A2,FCER1G,FCER1G,FCER2,FCGR1A,FCGR1A,ALDH3B1,RASGEF1A,FCGR2A,FCGR2A,FCGR2B,FCGR3A,FCGR3A,FCGR3B,RNF182,FCN1,FCN2,FBXL13,ADGRG3,ZNRF2,FGA,FGB,FGF1,FGF2,FGF3,FGF4,FGF5,FGF6,FGF7,FGF8,FGF9,FGF10,ALDOA,FGFR1,FGFR3,FGFR2,FGFR4,FGG,FGR,FKBP1A,MRAS,RASA3,NLRP1,CNKSR2,SEC31A,ENPP4,UBOX5,KLRK1,KLRK1,CD93,KIFAP3,RAB18,TRIM32,ALDOC,FBXO21,ERP44,FOXO1,TRIM35,FOXO3,SARM1,RAP1GAP2,NFASC,TAB2,FLNA,NUP205,FLNB,RGL1,CYFIP1,CYFIP1,FBXL7,FAF2,PSME4,PSME4,ATP11B,NBEAL2,FLT3,FLT3,DTX4,NUP210,FLT3LG,FLT3LG,ATP11A,NUP160,FBXW11,MGRN1,UBR2,ICOSLG,DNAJC13,TRIM2,NEDD4L,FN1,UBR4,UFL1,NCSTN,FNTA,COTL1,COMMD3,FNTB,ICMT,SEC61G,TNFRSF13B,NUP188,FOLR3,ARHGAP45,CLCF1,FOS,ATP6V0A2,LILRA4,FPR1,FPR2,DDX58,DDX58,HEBP2,CLEC5A,MKRN1,NUP62,LY96,PLD3,TRIM29,IL17RA,ALOX5,KIF4A,IFIT5,PANX1,FRK,DEFB105A,DEFB106A,DEFB107A,DEFB108B,DEFB110,DEFB113,DEFB114,DEFB115,DEFB116,DEFB119,DEFB121,DEFB123,DEFB124,DEFB125,DEFB128,DEFB130A,ATP6V0D2,ATP6V1C2,ALOX15,CDC26,PELI3,IL27,IL27,MTOR,FTH1,ABL1,FTL,FUCA1,FUCA2,RICTOR,FYB1,FYB1,FYN,FYN,IFI6,XRCC6,GAA,GAB1,EPGN,RNF144B,TAB3,SHC2,RASGRP3,RASGRP3,FBXO7,QPCT,BRI3,GCA,SVIP,ARIH1,HECTD1,METTL7A,ANAPC13,ARMC8,GALNS,RNF19A,RCHY1,NCR3,PTPN23,SAMHD1,SH2B1,AOC1,TKFC,LTN1,DNM3,HERC4,PTPN20,WSB1,KIF26A,FBXW2,PTPN22,FBXL3,FBXO2,FBXL5,FBXL4,KLHL3,GATA3,CLEC4E,CLEC4E,FBXW8,FBXO22,FBXO10,FBXO9,FBXO6,FBXO4,FGF20,GBP1,GBP2,GBP3,PTPN18,IL36RN,OSTF1,GDI2,GDNF,GFRA1,GFRA2,GFRA3,B4GALT1,GH1,GH1,GH2,GH2,GHR,GHR,CYFIP2,CYFIP2,FGF22,SIGLEC7,LAT,LAT,DAPP1,GLA,IL36B,IL37,IL36A,SIGLEC9,SIGLEC8,IL17C,AMPD3,GLB1,C5AR2,RNF115,PDCD4,KLHL20,CRCP,UBE2S,GM2A,GNS,GPI,IFNL2,IFNL3,IFNL1,KSR2,MUC19,FBXL22,ANGPT1,SIGLEC15,NKIRAS2,NKIRAS1,FBXW12,KIF4B,TREML4,GRB2,GRB2,RAPGEF1,LAMTOR2,GRN,DBNL,ANPEP,GRIN1,GRIN2B,GRIN2D,COMMD9,PYCARD,PYCARD,TBK1,TBK1,MYLIP,CLEC2D,CD274,RACGAP1,CXCL1,CXCL2,PDIA3,PDIA3,GSN,GSTA2,GSTP1,BLNK,BLNK,ICOS,ANAPC2,GUSB,GYG1,SEC61A1,ANAPC4,IL19,DPP7,PILRB,PILRA,ACAA1,SH3KBP1,SH3KBP1,GZMM,ANXA1,ANXA2,HBB,HCK,HCK,NCKAP1L,NCKAP1L,HEXB,CFH,CFHR1,CFHR2,HGF,HGF,CD209,NRG1,PIK3R4,HIF1A,UBE2K,UBE2K,HK3,HLA-A,HLA-A,HLA-B,HLA-B,HLA-C,HLA-C,HLA-DMB,HLA-DOA,HLA-DOB,HLA-DPA1,HLA-DPB1,HLA-DQA1,HLA-DQA2,HLA-DQB1,HLA-DQB2,HLA-DRA,HLA-DRB1,HLA-DRB3,HLA-DRB4,HLA-DRB5,HLA-E,HLA-E,HLA-F,HLA-F,HLA-G,HLA-G,HMGB1,HMGB1,HMOX1,HMOX2,APAF1,EIF4E3,HNRNPA2B1,HNRNPF,HP,HRAS,RAB37,APEH,BIRC2,BIRC3,HSPA1A,HSPA1B,HSPA5,HSPA5,HSPA6,HSPA8,HSPA9,BIRC5,HSP90AA1,HSP90AB1,H3C15,H3C15,HTN1,HTN3,APOB,ICAM1,S100A7A,CLEC4D,ICAM2,ICAM3,ICAM4,CLEC4G,IRF8,STING1,TREML1,IDH1,CD300E,CFI,IFI16,IFI27,IFI35,IFIT2,IFIT1,IFIT3,IFNA1,IFNA1,IFNA2,IFNA2,IFNA4,IFNA4,IFNA5,IFNA5,IFNA6,IFNA6,IFNA7,IFNA7,IFNA8,IFNA8,IFNA10,IFNA10,IFNA13,IFNA13,IFNA14,IFNA14,IFNA16,IFNA16,IFNA17,IFNA17,IFNA21,IFNA21,IFNAR1,IFNAR1,IFNAR2,IFNAR2,IFNB1,IFNB1,IFNG,IFNG,IFNGR1,IFNGR1,IFNGR2,IFNGR2,TUBB8,TUBB2B,IGF2R,NUP43,APP,APP,APRT,SLCO4C1,TICAM2,LILRA5,IKBKB,IKBKB,IL1A,IL1B,IL1R1,IL1RAP,IL1RN,IL2,IL2RA,FASLG,IL2RB,IL2RB,IL2RG,IL2RG,IL3,IL3RA,IL4,IL4R,IL5,IL5RA,IL6,IL6R,IL6ST,IL6ST,IL7,IL7,IL7R,IL7R,CXCL8,CXCR1,IL9,CXCR2,IL9R,IL10,IL10RA,IL10RB,IL11,IL11RA,IL12A,IL12A,IL12B,IL12B,IL12RB1,IL12RB1,IL12RB2,IL12RB2,IL13,IL13RA1,IL13RA2,IL15,IL15,IL15RA,IL15RA,IL16,TNFRSF9,IL17A,IL18,ILF2,IMPDH1,IMPDH2,CXCL10,INPP5D,INPPL1,IRAK1,IRAK2,IRF1,IRF2,IRF3,IRF3,IRF4,IRF5,IRF6,IRF7,IRS1,IRS1,ISG20,ITGA2B,ITGA4,ITGAL,ITGAM,ITGAV,ITGAX,ITGB1,ITGB2,ARAF,ITGB3,ITGB5,ITGB7,ITK,ITK,ITPR1,ITPR1,ITPR2,ITPR2,ITPR3,ITPR3,JAK1,JAK1,JAK2,JAK2,JAK3,JAK3,JUN,JUNB,JUP,AREG,NCR3LG1,TMEM179B,TBC1D10C,ARF1,USP17L2,KIF2A,KIF3C,KIF5A,KIF5B,KIR2DL1,KIR2DL2,KIR2DL3,KIR2DL4,KIR2DS1,KIR2DS4,KIR2DS5,KIR3DL1,KIR3DL2,KIR3DS1,KIT,KLRB1,KLRC1,KLRC2,KLRC2,KLRD1,KLRD1,ARG1,KLC1,KIF11,KIF22,KPNA1,KPNB1,KPNA2,KPNA3,KPNA4,KPNA5,KRAS,KRT1,IL31,RHOA,C6orf120,TMEM189-UBE2V1,NHLRC3,CCL4L1,GBP7,HRNR,FLG2,LAG3,LAIR1,LAIR2,KBTBD13,RHOG,LAMA5,LAMP1,LAMP2,LBP,LCK,LCK,LCN2,LCP1,LCP2,LCP2,MUC21,LGALS3,LGALS3,LGALS9,LIF,LIFR,LIMK1,LIMK1,SPRED3,LMNB1,LMO7,DEFB132,ASB18,LNPEP,RAB44,ARL2,LPO,KPNA7,LRMP,LTA4H,LTA,LTA,LTB,LTB,LTBR,LTBR,LTF,CD180,LYN,LYN,SH2D1A,LYZ,TNFSF12-TNFSF13,ARRB1,SMAD3,ARRB2,ARSA,ARSB,MAN2B1,MANBA,MAOA,MARK3,CCL3L3,DEFB103A,MBL2,ART1,MCL1,CD46,MEF2A,MEF2C,MEFV,MAP3K1,MAP3K1,MAP3K3,MET,KITLG,MGST1,CIITA,CD99,ASAH1,MICB,MID1,MIF,MAP3K11,MME,MMP1,MMP2,MMP3,MMP8,MMP9,MNDA,CD200,MPO,MRC1,MRC1,MRE11,TARM1,MSN,MT2A,MTAP,MUC1,MUC3A,MUC4,MUC5AC,MUC6,MUC7,TRIM37,MX1,MX2,MYC,MYD88,MYH2,MYH9,MYO1C,MYO5A,MYO9B,MYO10,ATF1,CEACAM6,NCAM1,NCF2,NCF4,NCK1,NCK1,NDN,ACLY,NDUFC2,NEDD4,NEFL,ATOX1,NEU1,NF1,NF2,NFATC1,NFATC1,NFATC2,NFATC2,NFATC3,NFATC3,NFKB1,NFKB2,NFKBIA,NFKBIB,NFKBIE,NME2,NOS1,NOS2,NOS3,PNP,NRAS,NRTN,NUP88,NUP98,OAS1,OAS2,OAS3,OLR1,TNFRSF11B,OPRD1,OPRM1,ORM1,ORM2,OSM,P2RX1,P2RX7,P2RX7,P4HB,PA2G4,DEFB107B,DEFB104B,PEBP1,DEFB106B,DEFB105B,CD207,CD207,PAFAH1B2,SERPINB2,PAK1,PAK1,IL20,IL21R,IL22,ATP6V0A4,PAK2,PAK2,PAK3,REG3A,PRKN,TRAT1,CLEC4A,PCBP2,DERA,POLR1D,KLHL5,ABHD17B,ZDHHC9,GOLGA7,TRIM17,SAR1B,ASB3,RLIM,IRAK4,DYNC1LI1,DCTN4,HERC5,RAB9B,VRK3,PDZD11,TLR7,ECSIT,BPIFA1,TLR8,PLAC8,MEX3C,PDCD1,TNFRSF12A,SPTBN5,FZR1,KLRF1,ATP6V1D,PDE3B,PDE3B,BIN2,DDX41,ANAPC5,ANAPC7,RNF138,IP6K2,UBE2J1,EVL,PDE6D,NCKIPSD,NCKIPSD,ANAPC11,PDGFA,PDGFB,RAB14,PDGFRA,IL23A,IL23A,PDGFRB,ATP6V1H,UBE2D4,YPEL5,ASB1,ASB4,ASB2,PDPK1,CAB39,FBXO40,POLR3K,PECAM1,ERAP1,CALML5,TUBA8,ADA2,CFP,PFKL,PGAM1,ATP6V1A,PGM1,PHB,ATP6V1B1,ATP6V1B2,SERPINA1,PI3,SERPINB6,ATP6V0C,SERPINB10,ATP6V1C1,PIGR,PIK3C3,ATP6V1E1,PIK3CA,PIK3CA,PIK3CB,PIK3CB,PIM1,PIK3CD,PIK3CD,PIK3R1,PIK3R1,PIK3R2,PIK3R2,PIN1,PITPNA,PKM,PKP1,PLA2G2A,PLAU,PLAUR,ATP6V0B,BTBD1,PLCG1,PLCG1,SHC3,PLCG2,PLCG2,PLD1,NUP54,PLD2,ATP6V1G2,ATP6V0A1,PML,ATP7A,GPR84,IL20RA,IL20RB,TRIM34,RAB4B,RAB24,BRWD1,BRWD1,TLR9,TREM2,TREM2,TREM1,POLR2E,POLR2F,POLR2H,POLR2K,POLR2L,POMC,FBXW5,TOLLIP,RNF216,RHOF,POU2F1,APBB1IP,FBXL19,PPBP,XAF1,IL17RD,CTSA,RNF111,PPIA,PPIA,FBXL12,CMTM6,UBE2R2,PPL,RNF125,PPM1B,ACP3,PPP1CB,LAMTOR1,HERC6,PPP1CC,DET1,TRIM68,PPP2CA,PPP2CB,KLHL11,SEC61A2,PPP2R1A,RNF220,PPP2R1B,TRIM62,UBA6,PPP2R5A,PPP2R5B,PPP2R5C,PGM2,PPP2R5D,UBE2W,PPP2R5E,BOLA2,FBXW7,PPP3CA,PPP3CA,CPPED1,PPP3CB,PPP3CB,FBXL8,PPP3R1,PPP3R1,PPP5C,PRCP,TRIM36,PRG2,IL17RB,UBE2Q1,OTUD5,ITLN1,PRR5,RNF126,PRKACA,PRKACB,PRKACG,POLR3B,NDC1,POLR3E,ENAH,NUP133,TMEM30A,PRKCB,PRKCD,IL26,PRKCE,RNF130,PAG1,CAND1,BRK1,BRK1,ACTR10,PRKCQ,PRKCQ,PRKCSH,DEFB103B,RNF114,PRKDC,PRKG1,PRKG2,MAPK1,MAPK1,MAPK3,MAPK3,KLHL9,MAPK7,MAPK7,MAPK8,MAPK11,MAPK9,MAPK10,MAPK13,MAP2K1,MAP2K1,MAP2K2,MAP2K3,MAP2K6,MAP2K7,EIF2AK2,DNAJC3,PRL,PRL,PRLR,PRLR,PSPN,BTNL2,CRTAM,PROS1,IL36G,LGMN,PRSS2,PRSS3,MASP1,PRTN3,AZU1,PSAP,PSEN1,NPDC1,TRIM39,MUC13,B2M,B2M,RETN,PSMA1,PSMA1,PSMA2,PSMA2,PSMA3,PSMA3,PSMA4,PSMA4,PSMA5,PSMA5,PSMA6,PSMA6,PSMA7,PSMA7,KCMF1,PSMB1,PSMB1,PSMB2,PSMB2,PSMB3,PSMB3,PSMB4,PSMB4,PSMB5,PSMB5,PSMB6,PSMB6,PSMB7,PSMB7,NIT2,PSMB8,PSMB8,PSMB9,PSMB9,PSMB10,PSMB10,KIF15,PSMC1,PSMC1,PSMC2,PSMC2,PSMC3,PSMC3,VPS35L,PSMC4,PSMC4,PSMC5,PSMC5,PSMC6,PSMC6,PSMD1,PSMD1,PSMD2,PSMD2,PSMD3,PSMD3,PSMD4,PSMD4,PSMD5,PSMD5,PGLYRP4,EPPIN,NUP107,CD177,PSMD7,PSMD7,RGL3,PSMD8,PSMD8,PSMD9,PSMD9,SLC44A2,SMURF1,PSMD10,PSMD10,PELI2,PELI1,PSMD11,PSMD11,PSMD12,PSMD12,PSMD13,PSMD13,PSME1,PSME1,PSME2,PSME2,PTAFR,PTEN,PTGS2,PTK2,MAVS,MAVS,HECW2,HACE1,KLHL42,SH3RF1,RNF213,QSOX1,PTPN1,PTPN1,PTPN2,SPTBN4,PTPN3,PTPN4,TRIB3,PTPN6,PTPN6,PTPN7,PTPN9,PTPN11,PTPN11,PTPN12,SLAMF7,RAP2C,PTPN13,PTPN14,PTPRA,PTPRB,PTPRC,PTPRJ,PTPRN2,PTPRZ1,PTX3,PVR,NECTIN2,PYGB,PYGL,RHOU,NLRC4,NLRC4,TRAPPC1,ABHD17C,RGL2,RAB3A,RAB5B,RAB6A,RAB27A,RAB5C,RAC1,RAC1,RAC2,RAF1,RAG1,RAG2,RALA,IL22RA1,RALGDS,RANBP2,RAP1A,IL21,RAP1B,RAP1GAP,RAP2B,RASA1,RASA2,RASGRF1,RASGRF2,RBBP6,SIGIRR,CCND1,BCL2,REL,RELA,RELB,RET,BCL2L1,ACTB,ACTB,RNASE2,RNASE3,RNASE6,BCL6,EDA2R,RNASEL,RNF4,RNF6,ABCE1,TNFRSF17,ROCK1,RORA,RORC,DEFB135,DEFB136,DEFB134,RPLP0,RPS6KA1,RPS6KA2,RPS6KA3,RPS27A,RPS27A,S100A1,S100A7,S100A8,S100A9,S100A11,S100A12,S100A12,S100B,S100B,S100P,SAA1,SAA1,CFB,MAPK12,SERPINB3,CEACAM1,CCL2,CCL3,CCL3L1,CCL4,CCL5,CCL11,CCL19,CCL20,CCL22,SDC1,SDCBP,RNF123,UBE2O,ELMO2,SEC13,BLK,SELL,SEMG1,GFRA4,CRLF2,CRLF2,TMBIM1,NOD2,NOD2,IFIH1,IFIH1,RBSN,MAP2K4,MAP2K4,ERAP2,CARD9,CARD9,BLMH,PJA1,MLST8,RNF25,ARHGAP9,MMP25,SFTPD,KLHL25,WIPF3,WIPF3,DEFB131A,SH3GL2,CLEC7A,CLEC7A,SHC1,SHC1,FBXW4,ANAPC1,UNKL,SMURF2,SIAH1,SIAH2,IL25,KLC2,RASAL3,SIPA1,SKP1,SKP2,SLC2A3,SLC2A5,UBE2Z,NCF1,SFTPA1,H3C13,H3C13,NME1-NME2,BOLA2B,SLC11A1,SLPI,SMARCA4,POLR3D,SIGLEC1,SNAP25,FSCN1,SNRPA1,SOD1,SOD2,SOS1,SOS1,SOS2,SOX2,SP100,SPTA1,SPTAN1,BPI,SPTB,SPTBN1,SPTBN2,SRC,SRP14,BRAF,TRIM21,STAT1,STAT1,STAT2,STAT2,STAT3,STAT3,STAT4,STAT4,STAT5A,STAT5A,STAT5B,STAT5B,STAT6,STIM1,STIM1,STK10,STX1A,STX3,STX4,STXBP2,BST1,SURF4,BST2,VAMP2,VAMP7,BTC,SYK,SYK,ADAM17,MAP3K7,MAP3K7,TALDO1,TAP1,TAP1,TAP2,TAP2,TAPBP,ELOC,ELOB,ZEB1,TCN1,BTK,BTK,TCP1,BTN1A1,DYNLT1,TEC,TEC,TEK,TGFA,TGFB1,THOP1,TIMP1,TIMP2,ICAM5,TLN1,TLR1,TLR2,TLR3,TLR4,ACTG1,ACTG1,SERPING1,TLR5,C1QA,TNF,TNFAIP3,C1QB,TNFAIP6,TNFRSF1A,TNFRSF1B,C1QC,C1R,TP53,C1S,C2,TPP2,TPR,C3,HSP90B1,TRAF2,TRAF2,TRAF3,TRAF6,TRAF6,C3AR1,C4A,C4B,C4BPA,TRPC1,TRPM2,C4BPB,C5,TTR,TUBA4A,TUBA3C,MUC5B,C5AR1,TUBB2A,DEFA1B,C6,TWIST1,TNFSF4,CCR2,SFTPA2,TNFRSF4,TXK,TXK,TXN,TXN,TYK2,TYK2,C7,TYROBP,TYROBP,C8A,UBA52,UBA52,UBB,UBB,UBC,UBC,UBA1,UBA7,UBE2A,C8B,UBE2B,UBE2D1,UBE2D1,UBE2D2,UBE2D2,UBE2D3,UBE2D3,UBE2E1,UBE2E2,UBE2G1,UBE2G2,UBE2H,C8G,UBE2L3,UBE2N,UBE2V1,UBE2V2,UBE3A,SUMO1,C9,VASP,VAV1,VAV1,VAV2,VAV2,VCAM1,VCL,VCP,VEGFA,VHL,VIM,VTN,VWF,WAS,WAS,WIPF1,WIPF1,LAT2,LAT2,XDH,XRCC5,YES1,YES1,YWHAB,YWHAZ,ZAP70,CA1,ZBTB16,TRIM25,TRIM25,TRIM26,TUBA1A,IL1R2,MAPKAPK3,RAB7A,COLEC11,NUP37,TRIM48,MAPKAP1,DHX58,TNIP2,C1orf35,FBXL15,ULBP3,HECTD3,NLRX1,ASB13,SEM1,SEM1,FBXO31,GSDMD,TUBAL3,TREML2,UBA5,LPCAT1,ATP8B4,NUP85,BTNL8,NANOG,DOK3,DSN1,CALM1,CALM1,TRIM46,PTGES2,SIKE1,SPSB1,CEP290,RNF34,FBXO11,NUP214,ALPK1,ORAI2,TRIM45,PLEKHO2,ULBP1,DNAJC5,BPIFB2,SHOC2,PDCD1LG2,CALM2,CALM2,CUL5,FGF23,CALM3,CALM3,DUSP16,AAAS,TUBB1,ZBP1,ZBP1,COLEC12,CALR,GAN,CFHR5,CAMK2A,TRIM11,TXNDC5,CAMK2B,TRIM8,TSPAN14,UNC93B1,DEFB126,CAMK2D,MADCAM1,TLR10,CAMK2G,TRIM56,ABHD17A,SEH1L,KIF18A,CAMP,CANX,CANX,CAPN1,EPX,CAPZA1,CAPZA2,BRAP,CAPZB,CASP1,CASP1,CASP2,H3C1,H3C1,H3C4,H3C4,H3C3,H3C3,H3C6,H3C6,H3C11,H3C11,RILP,H3C8,H3C8,H3C12,H3C12,H3C10,H3C10,H3C2,H3C2,CASP3,CASP4,CRISPLD2,ITCH,ATAD3B,PLA2G6,MAGT1,KBTBD7,FBXO30,CASP8,EEA1,NLRC5,CASP9,TRAF7,FBXW9,POLR3GL,RNF135,RNF135,WDR83,CASP10,HVCN1,RASAL1,CYSTM1,CARD11,CARD11,FRMPD3,PLPP5,GHDC,DCTN5,CUL3,CUL2,CUL1,KBTBD8,TUBB6,IL1F10,KIF2B,ADGRE3,RNASE7,TRIM63,CAT,LNX1,SPSB2,CRACR2A,TUBA1C,RAE1,IL17RC,KLHL22,PTPN5,HAVCR2,ORAI1,ZNRF1,FBXL20,PIK3R3,PIK3R3,KCNAB2,IKBKG,IKBKG,IFITM1,TRIM5,FCN3,TSLP,TSLP,PIAS1,DEGS1,PDXK,TNFSF11,CDK13,RNASET2,OASL,LAMTOR3,SOCS1,SOCS1,DYNLL1,IRS2,IRS2,CBL,CBL,EIF4G3,VAMP8,CBLB,PEA15,DGAT1,CDC23,TNFRSF25,CTSF,RIPK1,TNFSF14,TNFSF14,TNFSF13,TNFSF12,TNFSF9,TNFRSF14,TNFRSF14,RIPK2,RIPK2,TNFRSF6B,FADD,SNAP23,SIGLEC5,TNFRSF18,TNFRSF11A,ACTN2,CREG1,IL18RAP,IL1RL2,IL18R1,FGF18,FGF17,FGF16,IQGAP1,SYNGAP1,SOCS2,SOCS2,GGH,KSR1,VNN1,SQSTM1,CDC16,TAX1BP1,CPNE3,CPNE1,AP1S2,AP1M1,TRIM4,BCL10,BCL10,HERC3,HERC2,HERC1,WASF1,WASF1,BTRC,H3C7,H3C7,MGAM,WASL,WASL,SERPINB12,SIGLEC10,SIGLEC12,STBD1,KBTBD6,CCNF,UBE3B,ATP6V0E1,PGLYRP1,TNFSF18,KLC4,UBE3D,BTBD6,MAP3K14,SOCS3,SOCS3,KLHL13,UBA3,UBE2M,ATP6V1E2,ARTN,AIP,PSTPIP1,LRSAM1,IL33,CD1A,TRIM41,CD1B,CD1C,ATP6V0D1,CD1D,ATG12,SYNGR1,CD3D,RSAD2,CD3E,LONRF1,CD3G,CD3G,IL1RL1,OSMR,CD247,CD247,CD4,LRRFIP1,VAPA,IL32,SPSB4,CRLF1,UBE2L6,UBE2L6,CD8A,RPS6KA5,ASB16,CD8B,MAPKAPK2,TIFA,BPIFB1,CD14,UBE2Q2,ATP6V1F,CD19,CD19,NAPRT,MS4A3,TRIP12,CD22,CD22,VAMP3,SNAP29,UBE4A,FBXO44,KL,CAPZA3,KIF3B,CD27,CLEC6A,CLEC6A,CD101,CD28,GRAP2,GRAP2,MUC16,CD80,ORMDL3,CD86,TNFRSF8,NCR2,NCR1,TNFSF8,GSTO1,AIM2,AIM2,CD33,LY86,SIGLEC6,RASAL2,IL27RA,IL27RA,CD34,EIF4E2,ATG5,CD36,PSMF1,PSMF1,KIF23,NPEPPS,SCAMP1,POLR1C,GMFG,NRG2,RAB3D,ATP6V1G1,SEC22B,CD40,PRDX6,CD40LG,CD44,RNF14,CD47,RNF7,CD53,NUP155,SEC24C,ISG15,IKBKE,CD58,SOCS5,CD59,DZIP3,CD63,CD68,NUP93,UBE3C,RAPGEF2,CD70,CD74,TMEM63A,CD79A,CD79A,DCAF1,CD79B,CD79B,CD81,ADGRE5,MLEC,EIF4A3,CDA,IST1,KEAP1,NUP58,CUL7,TRIM14,ELMO1,GAB2,GAB2,PSMD6,PSMD6,PJA2,AREL1,SEC24D,POM121,MRC2,MRC2,KLHL21,CDC20,CDC27,MVP,FGF19,TNFSF15,CDC34,NUP153,CLEC2B,RBX1,CDC42,CDC42,RCE1,HNRNPDL,CDH1",Immune System,2461

R-HSA-168268,"CALR,CANX",Virus Assembly and Release,2

R-HSA-168271,"NUP50,NUP42,NUP35,NUP205,NUP210,NUP160,NUP188,NUP62,NUP43,KPNA1,KPNB1,NUP88,NUP98,NUP54,NDC1,NUP133,NUP107,RANBP2,SEC13,TPR,NUP37,NUP85,NUP214,AAAS,SEH1L,RAE1,NUP155,NUP93,NUP58,POM121,NUP153",Transport of Ribonucleoproteins into the Host Nucleus,31

R-HSA-168273,"NUP50,NUP42,RPL35,RPL39L,NUP35,RPS4Y2,RPL10L,PARP1,RPL22L1,NUP205,NUP210,NUP160,NUP188,RPL13A,NUP62,RPL36,GRSF1,GTF2F1,GTF2F2,HSP90AA1,NUP43,IPO5,RPSA,RPL10A,NUP88,NUP98,RPS27L,RPL26L1,NUP54,POLR2A,POLR2B,POLR2C,POLR2D,POLR2E,POLR2F,POLR2G,POLR2H,POLR2I,POLR2J,POLR2K,POLR2L,NDC1,NUP133,DNAJC3,NUP107,RANBP2,RPL3,RPL3L,RPL4,RPL5,RPL6,RPL7,RPL7A,RPL8,RPL9,RPL10,RPL11,RPL12,RPL13,RPL15,RPL17,RPL18,RPL18A,RPL19,RPL21,RPL22,RPL23A,RPL24,RPL26,RPL27,RPL30,RPL27A,RPL28,RPL29,RPL31,RPL32,RPL34,RPL35A,RPL36AL,RPL37,RPL37A,RPL38,RPL39,RPL41,RPL36A,RPLP0,RPLP1,RPLP2,RPS2,RPS3,RPS3A,RPS4X,RPS4Y1,RPS5,RPS6,RPS7,RPS8,RPS9,RPS10,RPS11,RPS12,RPS13,RPS14,RPS15,RPS15A,RPS16,RPS17,RPS18,RPS19,RPS20,RPS21,RPS23,RPS24,RPS25,RPS26,RPS27,RPS27A,RPS28,RPS29,SEC13,TPR,UBA52,NUP37,NUP85,NUP214,AAAS,SEH1L,RAE1,RPL14,RPL23,NUP155,NUP93,NUP58,POM121,NUP153",Influenza Viral RNA Transcription and Replication,135

R-HSA-168274,"NUP50,NUP42,NUP35,NUP205,NUP210,NUP160,NUP188,NUP62,HSPA1A,HSPA1B,NUP43,NUP88,NUP98,NUP54,NDC1,NUP133,NUP107,RAN,RANBP2,SEC13,TPR,XPO1,NUP37,NUP85,NUP214,AAAS,SEH1L,RAE1,NUP155,NUP93,NUP58,POM121,NUP153",Export of Viral Ribonucleoproteins from Nucleus,33

R-HSA-168275,"CLTA,CLTC",Entry of Influenza Virion into Host Cell via Endocytosis,2

R-HSA-168276,"NUP50,CPSF4,NUP42,NUP35,NUP205,NUP210,NUP160,NUP188,NUP62,NUP43,KPNA1,KPNB1,KPNA2,KPNA3,KPNA4,KPNA5,KPNA7,NUP88,NUP98,NUP54,NDC1,NUP133,EIF2AK2,NUP107,RANBP2,SEC13,TPR,NUP37,NUP85,NUP214,AAAS,PABPN1,SEH1L,RAE1,NUP155,ISG15,NUP93,NUP58,POM121,NUP153",NS1 Mediated Effects on Host Pathways,40

R-HSA-168277,"SLC25A6,TGFB1",Influenza Virus Induced Apoptosis,2

R-HSA-168315,"CPSF4,PABPN1",Inhibition of Host mRNA Processing and RNA Silencing,2

R-HSA-168316,"CALR,CANX",Assembly of Viral Components at the Budding Site,2

R-HSA-168325,"NUP50,NUP42,NUP35,NUP205,NUP210,NUP160,NUP188,NUP62,GTF2F1,GTF2F2,NUP43,NUP88,NUP98,NUP54,POLR2A,POLR2B,POLR2C,POLR2D,POLR2E,POLR2F,POLR2G,POLR2H,POLR2I,POLR2J,POLR2K,POLR2L,NDC1,NUP133,NUP107,RANBP2,SEC13,TPR,NUP37,NUP85,NUP214,AAAS,SEH1L,RAE1,NUP155,NUP93,NUP58,POM121,NUP153",Viral Messenger RNA Synthesis,43

R-HSA-168330,"HSPA1A,HSPA1B",Viral RNP Complexes in the Host Cell Nucleus,2

R-HSA-168333,"NUP50,NUP42,NUP35,NUP205,NUP210,NUP160,NUP188,NUP62,NUP43,NUP88,NUP98,NUP54,NDC1,NUP133,NUP107,RAN,RANBP2,SEC13,TPR,XPO1,NUP37,NUP85,NUP214,AAAS,SEH1L,RAE1,NUP155,NUP93,NUP58,POM121,NUP153",NEP/NS2 Interacts with the Cellular Export Machinery,31

R-HSA-168638,"NOD1,NOD1,TAB1,CHUK,AAMP,MAPK14,CYLD,TAB2,TAB3,BIRC2,BIRC3,IKBKB,IRAK1,IRAK2,TMEM189-UBE2V1,MAPK11,MAPK13,MAP2K6,RPS27A,MAPK12,NOD2,NOD2,CARD9,MAP3K7,TNFAIP3,TRAF6,UBA52,UBB,UBC,UBE2N,UBE2V1,CASP1,CASP2,CASP4,ITCH,CASP8,CASP9,IKBKG,RIPK2,RIPK2",NOD1/2 Signaling Pathway,40

R-HSA-168643,"NOD1,NOD1,TAB1,TXNIP,TXNIP,SUGT1,NLRP3,NLRP3,CHUK,AAMP,MAPK14,CYLD,NLRP1,TAB2,PANX1,TAB3,PYCARD,PYCARD,BIRC2,BIRC3,HSP90AB1,APP,IKBKB,IRAK1,IRAK2,TMEM189-UBE2V1,MEFV,NFKB1,NFKB2,P2RX7,P2RX7,MAPK11,MAPK13,MAP2K6,NLRC4,NLRC4,BCL2,RELA,BCL2L1,RPS27A,MAPK12,NOD2,NOD2,CARD9,MAP3K7,TNFAIP3,TRAF6,TXN,TXN,UBA52,UBB,UBC,UBE2N,UBE2V1,CASP1,CASP1,CASP2,CASP4,ITCH,CASP8,CASP9,IKBKG,RIPK2,RIPK2,PSTPIP1,AIM2,AIM2","Nucleotide-binding domain, leucine rich repeat containing receptor (NLR) signaling pathways",67

R-HSA-168799,"STX1B,SYT2,SV2C,SNAP25,STX1A,VAMP1,VAMP2,SYT1,SV2B,SV2A",Neurotoxicity of clostridium toxins,10

R-HSA-168898,"TANK,TMED7-TICAM2,TLR6,NOD1,TAB1,CNPY3,RIPK3,IRAK3,TIRAP,CHUK,MAP3K8,MAP3K8,CREB1,ATF2,MAPK14,TICAM1,CTSB,CTSK,CTSL,CTSV,CTSS,DNM1,AGER,DNM2,DUSP3,DUSP4,DUSP6,DUSP7,ELK1,FGA,FGB,FGG,SARM1,TAB2,FBXW11,FOS,LY96,PELI3,TAB3,DNM3,NKIRAS2,NKIRAS1,TBK1,PIK3R4,HMGB1,BIRC2,BIRC3,APOB,APP,TICAM2,IKBKB,IRAK1,IRAK2,IRF3,IRF7,ITGAM,ITGB2,JUN,TMEM189-UBE2V1,LBP,CD180,MEF2A,MEF2C,MAP3K1,MYD88,ATF1,NFKB1,NFKB2,NFKBIA,NFKBIB,IRAK4,VRK3,TLR7,ECSIT,TLR8,PIK3C3,PLCG2,TLR9,PPP2CA,PPP2CB,PPP2R1A,PPP2R1B,PPP2R5D,MAPK1,MAPK1,MAPK3,MAPK3,MAPK7,MAPK7,MAPK8,MAPK11,MAPK9,MAPK10,MAP2K1,MAP2K3,MAP2K6,MAP2K7,LGMN,PELI2,PELI1,PTPN4,PTPN11,SIGIRR,RELA,RPS6KA1,RPS6KA2,RPS6KA3,RPS27A,S100A1,S100A8,S100A9,S100A12,S100B,SAA1,NOD2,RBSN,MAP2K4,MAP2K4,SFTPD,SKP1,SFTPA1,BPI,MAP3K7,BTK,TLR1,TLR2,TLR3,TLR4,TLR5,HSP90B1,TRAF3,TRAF6,SFTPA2,UBA52,UBB,UBC,UBE2D1,UBE2D2,UBE2D3,UBE2N,UBE2V1,MAPKAPK3,TNIP2,UNC93B1,TLR10,CASP8,EEA1,CUL1,IKBKG,SOCS1,RIPK1,RIPK2,FADD,BTRC,RPS6KA5,MAPKAPK2,CD14,LY86,CD36,IKBKE",Toll-like Receptor Cascades,160

R-HSA-168927,"RIPK3,CHUK,TICAM1,BIRC2,BIRC3,IKBKB,TMEM189-UBE2V1,RPS27A,TLR3,TRAF6,UBA52,UBB,UBC,UBE2D1,UBE2D2,UBE2D3,UBE2N,UBE2V1,IKBKG,RIPK1","TICAM1, RIP1-mediated IKK complex recruitment",20

R-HSA-168928,"TANK,CHUK,CREBBP,CYLD,AGER,EP300,DDX58,DDX58,TKFC,NKIRAS2,NKIRAS1,TBK1,UBE2K,HMGB1,IFNA1,IFNA2,IFNA4,IFNA5,IFNA6,IFNA7,IFNA8,IFNA10,IFNA13,IFNA14,IFNA16,IFNA17,IFNA21,IFNB1,APP,IKBKB,IRF3,IRF7,MAP3K1,MAP3K1,NFKB1,NFKB2,NFKBIA,NFKBIB,PCBP2,HERC5,PIN1,RNF216,RNF125,OTUD5,MAVS,MAVS,RELA,RPS27A,S100A12,S100B,SAA1,IFIH1,IFIH1,TNFAIP3,TRAF2,TRAF2,TRAF3,TRAF6,TRAF6,UBA52,UBB,UBC,UBA7,UBE2D1,UBE2D2,UBE2D3,TRIM25,TRIM25,DHX58,NLRX1,SIKE1,ITCH,CASP8,NLRC5,RNF135,RNF135,CASP10,IKBKG,RIPK1,FADD,TAX1BP1,ATG12,UBE2L6,UBE2L6,ATG5,ISG15,IKBKE",DDX58/IFIH1-mediated induction of interferon-alpha/beta,87

R-HSA-169131,EIF2AK2,Inhibition of PKR,1

R-HSA-169893,"FRS2,FRS2,CRK,CRK,CRKL,CRKL,RAPGEF1,RAPGEF1,NGF,NGF,NTRK1,NTRK1,MAPK1,MAPK3,MAP2K1,MAP2K2,KIDINS220,KIDINS220,RAP1A,RAP1A,BRAF,BRAF,YWHAB,YWHAB",Prolonged ERK activation events,24

R-HSA-169911,"PSME3,PSMD14,OMA1,PSMB11,PSMA8,PSME4,OPA1,PAK2,PSMA1,PSMA2,PSMA3,PSMA4,PSMA5,PSMA6,PSMA7,PSMB1,PSMB2,PSMB3,PSMB4,PSMB5,PSMB6,PSMB7,PSMB8,PSMB9,PSMB10,PSMC1,PSMC2,PSMC3,PSMC4,PSMC5,PSMC6,PSMD1,PSMD2,PSMD3,PSMD4,PSMD5,PSMD7,PSMD8,PSMD9,PSMD10,PSMD11,PSMD12,PSMD13,PSME1,PSME2,RPS27A,UBA52,UBB,UBC,ARHGAP10,SEM1,PSMF1,PSMD6",Regulation of Apoptosis,53

R-HSA-170145,"CCNA2,CCNA1,CDK1",Phosphorylation of proteins involved in the G2/M transition by Cyclin A:Cdc2 complexes,3

R-HSA-170660,"ADCY1,ADCY2,ADCY3,ADCY5,ADCY6,ADCY7,ADCY8,ADCY9,ADCY4,GNAL",Adenylate cyclase activating pathway,10

R-HSA-170670,"ADCY1,ADCY1,ADCY2,ADCY2,ADCY3,ADCY3,ADCY5,ADCY5,ADCY6,ADCY6,ADCY7,ADCY7,ADCY8,ADCY8,ADCY9,ADCY9,ADCY4,ADCY4,GNAI1,GNAI1,GNAI2,GNAI2,GNAI3,GNAI3,GNAL,GNAT3,GNAT3",Adenylate cyclase inhibitory pathway,27

R-HSA-170822,"NUP50,NUP42,NUP35,NUP205,NUP210,NUP160,NUP188,NUP62,GCK,GCKR,NUP43,NUP88,NUP98,NUP54,NDC1,NUP133,NUP107,RANBP2,SEC13,TPR,NUP37,NUP85,NUP214,AAAS,SEH1L,RAE1,NUP155,NUP93,NUP58,POM121,NUP153",Regulation of Glucokinase by Glucokinase Regulatory Protein,31

R-HSA-170834,"CDK8,CDK9,STUB1,CDKN2B,STRAP,STRAP,PARP1,E2F4,E2F5,FKBP1A,FKBP1A,SNW1,NEDD4L,NEDD4L,ARHGEF18,ARHGEF18,ATP1B4,PPP1R15A,BAMBI,WWTR1,HDAC1,JUNB,RHOA,RHOA,SMAD2,SMAD2,SMAD3,SMAD3,SMAD4,SMAD4,SMAD7,SMAD7,MEN1,MYC,NEDD8,FURIN,SERPINE1,F11R,F11R,PARD6A,PARD6A,UCHL5,TRIM33,RNF111,RNF111,PPM1A,PPP1CA,PPP1CB,PPP1CC,PRKCZ,PRKCZ,PARD3,PARD3,PMEPA1,SMURF1,SMURF1,CGN,CGN,RBL1,TGIF2,RPS27A,RPS27A,SMURF2,SMURF2,SKI,SKI,SKIL,SKIL,SP1,TFDP1,TFDP2,TGFB1,TGFB1,TGFBR1,TGFBR1,TGFBR2,TGFBR2,TGIF1,UBA52,UBA52,UBB,UBB,UBC,UBC,UBE2D1,UBE2D3,XPO1,USP9X,CBL,CCNK,CCNC,CCNT1,UBE2M,CCNT2,MTMR4,ZFYVE9,ZFYVE9,NCOR1,NCOR1,NCOR2,NCOR2,USP15",Signaling by TGF-beta Receptor Complex,102

R-HSA-170968,"FRS2,FRS2,CRKL,CRKL,RAPGEF1,RAPGEF1,NGF,NGF,NTRK1,NTRK1,MAPK1,MAPK3,MAP2K1,MAP2K2,RAP1A,RAP1A,BRAF,BRAF,YWHAB,YWHAB",Frs2-mediated activation,20

R-HSA-170984,"CRK,CRK,NGF,NGF,NTRK1,NTRK1,KIDINS220,KIDINS220,RAP1A,RAP1A,BRAF,YWHAB",ARMS-mediated activation,12

R-HSA-171007,"MAPK14,MAPK14,HRAS,HRAS,KRAS,KRAS,NRAS,NRAS,MAPK11,MAPK11,MAPK13,RALA,RALB,RALGDS,RALGDS,MAPK12,SRC,MAPKAPK3,MAPKAPK2",p38MAPK events,19

R-HSA-171286,FURIN,Synthesis and processing of ENV and VPU,1

R-HSA-171306,"LOC102724334,H4-16,H2BU1,H2BC1,POT1,TINF2,H2AC8,H2AC7,H2AX,H2AZ1,H2BC5,H2BC3,H2AB1,TERF2IP,H4C15,H2AJ,ACD,TERF1,TERF2,H2AC19,H3-4,H4C9,H2AC14,H2AC6,H2AC4,H2AC18,H2AC20,H2BC8,H2BC13,H2BC15,H2BC14,H2BC7,H2BC6,H2BC9,H2BC10,H2BC4,H2BC17,H2BC21,H4C1,H4C4,H4C6,H4C12,H4C11,H4C3,H4C8,H4C2,H4C5,H4C13,H4C14,H2BC12,H2BC11,H2AZ2",Packaging Of Telomere Ends,52

R-HSA-171319,"CDK2,RUVBL2,DKC1,ANKRD28,POT1,TINF2,RTEL1,TERF2IP,GAR1,WRAP53,SHQ1,PPP6R3,PPP6C,NOP10,NHP2,ACD,TERF1,TERF2,TERT,PIF1,RUVBL1,CCNA2,CCNA1",Telomere Extension By Telomerase,23

R-HSA-173107,"CCR5,PPIA,CXCR4,CD4",Binding and entry of HIV virion,4

R-HSA-173599,"SLC35D1,UGDH,UGP2","Formation of the active cofactor, UDP-glucuronate",3

R-HSA-173623,"CRP,C1QA,C1QB,C1QC,C1R,C1S",Classical antibody-mediated complement activation,6

R-HSA-173736,"CFD,GZMM,CFP,CFB,C3",Alternative complement activation,5

R-HSA-174048,"ANAPC10,UBE2C,ANAPC16,CDC26,ANAPC15,UBE2S,ANAPC2,ANAPC4,ANAPC5,ANAPC7,ANAPC11,RPS27A,ANAPC1,UBA52,UBB,UBC,UBE2D1,UBE2E1,CDC23,CDC16,CCNB1,CDK1,CDC20,CDC27",APC/C:Cdc20 mediated degradation of Cyclin B,24

R-HSA-174084,"PSME3,PSMD14,ANAPC10,UBE2C,ANAPC16,CDC26,ANAPC15,UBE2S,ANAPC2,ANAPC4,FZR1,ANAPC5,ANAPC7,ANAPC11,PSMA1,PSMA2,PSMA3,PSMA4,PSMA5,PSMA6,PSMA7,PSMB1,PSMB2,PSMB3,PSMB4,PSMB5,PSMB6,PSMB7,PSMB8,PSMB9,PSMB10,PSMC1,PSMC2,PSMC3,PSMC4,PSMC5,PSMC6,PSMD1,PSMD2,PSMD3,PSMD4,PSMD5,PSMD7,PSMD8,PSMD9,PSMD10,PSMD11,PSMD12,PSMD13,PSME1,PSME2,RPS27A,ANAPC1,UBA52,UBB,UBC,UBE2D1,UBE2E1,SEM1,CDC23,CDC16,PSMF1,PSMD6,CDC27",Autodegradation of Cdh1 by Cdh1:APC/C,64

R-HSA-174113,"PSME3,PSMD14,PSMB11,PSMA8,PSME4,FBXO5,FZR1,PSMA1,PSMA2,PSMA3,PSMA4,PSMA5,PSMA6,PSMA7,PSMB1,PSMB2,PSMB3,PSMB4,PSMB5,PSMB6,PSMB7,PSMB8,PSMB9,PSMB10,PSMC1,PSMC2,PSMC3,PSMC4,PSMC5,PSMC6,PSMD1,PSMD2,PSMD3,PSMD4,PSMD5,PSMD7,PSMD8,PSMD9,PSMD10,PSMD11,PSMD12,PSMD13,PSME1,PSME2,RPS27A,SKP1,UBA52,UBB,UBC,SEM1,CUL1,BTRC,PSMF1,PSMD6,CDC20",SCF-beta-TrCP mediated degradation of Emi1,55

R-HSA-174143,"CDK2,PSME3,PSMD14,ANAPC10,UBE2C,ANAPC16,PSMB11,PSMA8,PSME4,CDC26,ANAPC15,FBXO5,UBE2S,ANAPC2,ANAPC4,MAD2L1,NEK2,FZR1,ANAPC5,ANAPC7,ANAPC11,PLK1,PSMA1,PSMA2,PSMA3,PSMA4,PSMA5,PSMA6,PSMA7,PSMB1,PSMB2,PSMB3,PSMB4,PSMB5,PSMB6,PSMB7,PSMB8,PSMB9,PSMB10,PSMC1,PSMC2,PSMC3,PSMC4,PSMC5,PSMC6,PSMD1,PSMD2,PSMD3,PSMD4,PSMD5,PSMD7,PSMD8,PSMD9,PSMD10,PSMD11,PSMD12,PSMD13,PSME1,PSME2,RB1,RPS27A,ANAPC1,SKP1,SKP2,AURKA,BUB1B,UBA52,UBB,UBC,UBE2D1,UBE2E1,SEM1,CUL1,CDC14A,CDC23,CDC16,CCNA2,CCNA1,CCNB1,BTRC,BUB3,AURKB,PTTG1,PSMF1,CDK1,PSMD6,CDC20,CDC27",APC/C-mediated degradation of cell cycle proteins,88

R-HSA-174154,"PSME3,PSMD14,ANAPC10,UBE2C,ANAPC16,PSMB11,PSMA8,PSME4,CDC26,ANAPC15,UBE2S,ANAPC2,ANAPC4,ANAPC5,ANAPC7,ANAPC11,PSMA1,PSMA2,PSMA3,PSMA4,PSMA5,PSMA6,PSMA7,PSMB1,PSMB2,PSMB3,PSMB4,PSMB5,PSMB6,PSMB7,PSMB8,PSMB9,PSMB10,PSMC1,PSMC2,PSMC3,PSMC4,PSMC5,PSMC6,PSMD1,PSMD2,PSMD3,PSMD4,PSMD5,PSMD7,PSMD8,PSMD9,PSMD10,PSMD11,PSMD12,PSMD13,PSME1,PSME2,RPS27A,ANAPC1,UBA52,UBB,UBC,UBE2D1,UBE2E1,SEM1,CDC23,CDC16,PTTG1,PSMF1,PSMD6,CDC20,CDC27",APC/C:Cdc20 mediated degradation of Securin,68

R-HSA-174178,"PSME3,PSMD14,ANAPC10,UBE2C,ANAPC16,PSMB11,PSMA8,PSME4,CDC26,ANAPC15,UBE2S,ANAPC2,ANAPC4,FZR1,ANAPC5,ANAPC7,ANAPC11,PLK1,PSMA1,PSMA2,PSMA3,PSMA4,PSMA5,PSMA6,PSMA7,PSMB1,PSMB2,PSMB3,PSMB4,PSMB5,PSMB6,PSMB7,PSMB8,PSMB9,PSMB10,PSMC1,PSMC2,PSMC3,PSMC4,PSMC5,PSMC6,PSMD1,PSMD2,PSMD3,PSMD4,PSMD5,PSMD7,PSMD8,PSMD9,PSMD10,PSMD11,PSMD12,PSMD13,PSME1,PSME2,RB1,RPS27A,ANAPC1,SKP2,AURKA,UBA52,UBB,UBC,UBE2D1,UBE2E1,SEM1,CDC23,CDC16,AURKB,PTTG1,PSMF1,PSMD6,CDC20,CDC27",APC/C:Cdh1 mediated degradation of Cdc20 and other APC/C:Cdh1 targeted proteins in late mitosis/early G1,74

R-HSA-174184,"PSME3,PSMD14,ANAPC10,UBE2C,ANAPC16,PSMB11,PSMA8,PSME4,CDC26,ANAPC15,UBE2S,ANAPC2,ANAPC4,MAD2L1,ANAPC5,ANAPC7,ANAPC11,PSMA1,PSMA2,PSMA3,PSMA4,PSMA5,PSMA6,PSMA7,PSMB1,PSMB2,PSMB3,PSMB4,PSMB5,PSMB6,PSMB7,PSMB8,PSMB9,PSMB10,PSMC1,PSMC2,PSMC3,PSMC4,PSMC5,PSMC6,PSMD1,PSMD2,PSMD3,PSMD4,PSMD5,PSMD7,PSMD8,PSMD9,PSMD10,PSMD11,PSMD12,PSMD13,PSME1,PSME2,RPS27A,ANAPC1,BUB1B,UBA52,UBB,UBC,UBE2D1,UBE2E1,SEM1,CDC23,CDC16,CCNA2,CCNA1,BUB3,PSMF1,CDK1,PSMD6,CDC20,CDC27",Cdc20:Phospho-APC/C mediated degradation of Cyclin A,73

R-HSA-174362,"SLC26A1,SLC26A2,SLC35B2,SLC35B3,PAPSS2,PAPSS1",Transport and synthesis of PAPS,6

R-HSA-174403,"GGT6,GGT1,GGT7,GGT5,OPLAH,GCLC,GCLM,GSS,CHAC2,CNDP2,GGCT,CHAC1",Glutathione synthesis and recycling,12

R-HSA-174411,"TEN1,POLD3,POLA2,POT1,TINF2,PCNA,POLA1,POLD1,POLD2,TERF2IP,CHTF8,PRIM1,PRIM2,POLD4,RFC1,RFC2,RFC3,RFC4,RFC5,CHTF18,ACD,TERF1,TERF2,DSCC1,STN1,CTC1",Polymerase switching on the C-strand of the telomere,26

R-HSA-174414,"POLD3,DNA2,FEN1,POT1,TINF2,LIG1,PCNA,POLD1,POLD2,TERF2IP,POLD4,RPA1,RPA2,RPA3,BLM,ACD,TERF1,TERF2,WRN",Processive synthesis on the C-strand of the telomere,19

R-HSA-174417,"TEN1,POLD3,DNA2,FEN1,POLA2,POT1,TINF2,LIG1,PCNA,POLA1,POLD1,POLD2,TERF2IP,CHTF8,PRIM1,PRIM2,POLD4,RFC1,RFC2,RFC3,RFC4,RFC5,RPA1,RPA2,RPA3,CHTF18,BLM,ACD,TERF1,TERF2,WRN,DSCC1,STN1,CTC1",Telomere C-strand (Lagging Strand) Synthesis,34

R-HSA-174430,"TEN1,POLA2,POT1,TINF2,POLA1,TERF2IP,PRIM1,PRIM2,ACD,TERF1,TERF2,STN1,CTC1",Telomere C-strand synthesis initiation,13

R-HSA-174437,"POLD3,DNA2,FEN1,POT1,TINF2,PCNA,POLD1,POLD2,TERF2IP,POLD4,RPA1,RPA2,RPA3,ACD,TERF1,TERF2,WRN",Removal of the Flap Intermediate from the C-strand,17

R-HSA-174490,"VPS37A,VPS37D,VPS28,UBAP1,VPS37C,RPS27A,TSG101,UBA52,UBB,UBC,VPS37B,MVB12B,MVB12A,NMT2",Membrane binding and targetting of GAG proteins,14

R-HSA-174495,"VPS37A,VPS37D,VPS28,UBAP1,VPS37C,RPS27A,TSG101,UBA52,UBB,UBC,VPS37B,MVB12B,MVB12A,NMT2","Synthesis And Processing Of GAG, GAGPOL Polyproteins",14

R-HSA-174577,"C4B_2,CFP,CFB,C2,C3,C4A,C4B,C5",Activation of C3 and C5,8

R-HSA-174824,"NR1H3,NPC2,CETP,APOA5,APOA5,AP2M1,AP2S1,CLTA,CLTC,AP2A1,AP2A2,AP2B1,ABCA1,A2M,ALB,CES3,PCSK9,LDLRAP1,FGF21,ANGPTL3,MYLIP,ZDHHC8,HDLBP,APOF,APOA1,APOA2,APOB,APOB,GPIHBP1,APOC1,APOC1,APOC2,APOC2,APOC3,APOC4,APOC4,APOE,APOE,LCAT,LDLR,LDLR,LIPA,LIPC,LPL,MTTP,NPC1,P4HB,FURIN,PCSK6,SAR1B,ANGPTL4,PCSK5,MBTPS2,LSR,PLTP,PRKACA,PRKACB,PRKACG,ANGPTL8,APOBR,NCEH1,CIDEC,LMF1,BMP1,SOAT1,NR1H2,VLDLR,CUBN,AMN,SOAT2,CREB3L3,MBTPS1,LMF2,LIPG,SCARB1,ABCG1","Plasma lipoprotein assembly, remodeling, and clearance",76

R-HSA-175474,"VPS37A,VPS37D,FURIN,VPS28,UBAP1,PPIA,VPS37C,RPS27A,TSG101,UBA52,UBB,UBC,VPS37B,MVB12B,MVB12A,NMT2",Assembly Of The HIV Virion,16

R-HSA-175567,"PSIP1,HMGA1,BANF1",Integration of viral DNA into host genomic DNA,3

R-HSA-176033,"NUP50,NUP42,PSIP1,NUP35,NUP205,NUP210,NUP160,NUP188,NUP62,SLC25A4,SLC25A5,SLC25A6,HMGA1,NUP43,KPNA1,NUP88,NUP98,NUP54,NDC1,NUP133,NUP107,RANBP2,SEC13,TPR,NUP37,NUP85,NUP214,AAAS,SEH1L,RAE1,BANF1,NUP155,NUP93,NUP58,POM121,NUP153",Interactions of Vpr with host cellular proteins,36

R-HSA-176034,"CDK9,CCNT1",Interactions of Tat with host cellular proteins,2

R-HSA-176187,"CDK2,DBF4,CHEK1,RAD9B,ORC6,ORC3,HUS1,MCM2,MCM3,MCM4,MCM5,MCM6,MCM7,ORC1,ORC2,ORC4,ORC5,ATR,MCM10,RAD1,RAD9A,RAD17,RFC2,RFC3,RFC4,RFC5,RPA1,RPA2,RPA3,CLSPN,CDC7,CDC45,ATRIP,MCM8,CDC6,CDC25A,CDC25C",Activation of ATR in response to replication stress,37

R-HSA-176407,"ANAPC10,UBE2C,ANAPC16,CDC26,ANAPC15,UBE2S,ANAPC2,ANAPC4,FZR1,ANAPC5,ANAPC7,ANAPC11,ANAPC1,UBE2D1,UBE2E1,CDC14A,CDC23,CDC16,CDC20,CDC27",Conversion from APC/C:Cdc20 to APC/C:Cdh1 in late anaphase,20

R-HSA-176408,"CDK2,PSME3,PSMD14,ANAPC10,UBE2C,ANAPC16,PSMB11,PSMA8,PSME4,CDC26,ANAPC15,FBXO5,UBE2S,ANAPC2,ANAPC4,MAD2L1,FZR1,ANAPC5,ANAPC7,ANAPC11,PLK1,PSMA1,PSMA2,PSMA3,PSMA4,PSMA5,PSMA6,PSMA7,PSMB1,PSMB2,PSMB3,PSMB4,PSMB5,PSMB6,PSMB7,PSMB8,PSMB9,PSMB10,PSMC1,PSMC2,PSMC3,PSMC4,PSMC5,PSMC6,PSMD1,PSMD2,PSMD3,PSMD4,PSMD5,PSMD7,PSMD8,PSMD9,PSMD10,PSMD11,PSMD12,PSMD13,PSME1,PSME2,RPS27A,ANAPC1,SKP1,BUB1B,UBA52,UBB,UBC,UBE2D1,UBE2E1,SEM1,CUL1,CDC23,CDC16,CCNA2,CCNA1,CCNB1,BTRC,BUB3,PSMF1,CDK1,PSMD6,CDC20,CDC27",Regulation of APC/C activators between G1/S and early anaphase,81

R-HSA-176409,"PSME3,PSMD14,ANAPC10,UBE2C,ANAPC16,PSMB11,PSMA8,PSME4,CDC26,ANAPC15,UBE2S,ANAPC2,ANAPC4,MAD2L1,NEK2,ANAPC5,ANAPC7,ANAPC11,PSMA1,PSMA2,PSMA3,PSMA4,PSMA5,PSMA6,PSMA7,PSMB1,PSMB2,PSMB3,PSMB4,PSMB5,PSMB6,PSMB7,PSMB8,PSMB9,PSMB10,PSMC1,PSMC2,PSMC3,PSMC4,PSMC5,PSMC6,PSMD1,PSMD2,PSMD3,PSMD4,PSMD5,PSMD7,PSMD8,PSMD9,PSMD10,PSMD11,PSMD12,PSMD13,PSME1,PSME2,RPS27A,ANAPC1,BUB1B,UBA52,UBB,UBC,UBE2D1,UBE2E1,SEM1,CDC23,CDC16,CCNA2,CCNA1,CCNB1,BUB3,PTTG1,PSMF1,CDK1,PSMD6,CDC20,CDC27",APC/C:Cdc20 mediated degradation of mitotic proteins,76

R-HSA-176412,"ANAPC10,UBE2C,ANAPC16,CDC26,ANAPC15,UBE2S,ANAPC2,ANAPC4,ANAPC5,ANAPC7,ANAPC11,PLK1,ANAPC1,UBE2D1,UBE2E1,CDC23,CDC16,CCNB1,CDK1,CDC27",Phosphorylation of the APC/C,20

R-HSA-176417,"FBXO5,FZR1,PLK1,CCNB1,CDK1,CDC20",Phosphorylation of Emi1,6

R-HSA-176814,"PSME3,PSMD14,ANAPC10,UBE2C,ANAPC16,PSMB11,PSMA8,PSME4,CDC26,ANAPC15,UBE2S,ANAPC2,ANAPC4,MAD2L1,NEK2,ANAPC5,ANAPC7,ANAPC11,PLK1,PSMA1,PSMA2,PSMA3,PSMA4,PSMA5,PSMA6,PSMA7,PSMB1,PSMB2,PSMB3,PSMB4,PSMB5,PSMB6,PSMB7,PSMB8,PSMB9,PSMB10,PSMC1,PSMC2,PSMC3,PSMC4,PSMC5,PSMC6,PSMD1,PSMD2,PSMD3,PSMD4,PSMD5,PSMD7,PSMD8,PSMD9,PSMD10,PSMD11,PSMD12,PSMD13,PSME1,PSME2,RPS27A,ANAPC1,BUB1B,UBA52,UBB,UBC,UBE2D1,UBE2E1,SEM1,CDC23,CDC16,CCNA2,CCNA1,CCNB1,BUB3,PTTG1,PSMF1,CDK1,PSMD6,CDC20,CDC27",Activation of APC/C and APC/C:Cdc20 mediated degradation of mitotic proteins,77

R-HSA-176974,"MCM2,MCM3,MCM4,MCM5,MCM6,MCM7,GINS2,GINS2,GINS3,GINS3,CDC45,GINS4,GINS4,MCM8,GINS1,GINS1",Unwinding of DNA,16

R-HSA-177128,"GLYAT,ACSM2A,GLYATL2,ACSM4,ACSM2B,GLYATL3,ACSM5,GLYATL1",Conjugation of salicylate with glycine,8

R-HSA-177135,"GLYAT,ACSM1,GLYATL2,ACSM2B,GLYATL3,GLYATL1",Conjugation of benzoate with glycine,6

R-HSA-177162,"ACSM1,ACSM2B",Conjugation of phenylacetate with glutamine,2

R-HSA-177243,"NUP50,NUP50,RCC1,NUP42,NUP42,NUP35,NUP35,NUP205,NUP205,NUP210,NUP210,NUP160,NUP160,NUP188,NUP188,NUP62,NUP62,NUP43,NUP43,KPNB1,KPNB1,NPM1,NPM1,NUP88,NUP88,NUP98,NUP98,NUP54,NUP54,NDC1,NDC1,NUP133,NUP133,NUP107,NUP107,RAN,RANBP1,RANBP2,RANBP2,RANGAP1,SEC13,SEC13,TPR,TPR,XPO1,NUP37,NUP37,NUP85,NUP85,NUP214,NUP214,AAAS,AAAS,SEH1L,SEH1L,RAE1,RAE1,NUP155,NUP155,NUP93,NUP93,NUP58,NUP58,POM121,POM121,NUP153,NUP153",Interactions of Rev with host cellular proteins,67

R-HSA-177504,"DNAL4,AP2M1,AP2M1,AP2S1,AP2S1,CLTA,CLTA,CLTC,CLTC,AP2A1,AP2A1,AP2A2,AP2A2,AP2B1,AP2B1,DNM1,DNM2,DNM3,NGF,NGF,NTRK1,NTRK1,SH3GL2,SH3GL2",Retrograde neurotrophin signalling,24

R-HSA-177539,"PSIP1,HMGA1,BANF1",Autointegration results in viral DNA circles,3

R-HSA-177929,"ADAM10,SPRY1,SPRY2,STAM2,AAMP,CSK,HBEGF,EGF,EGF,EGFR,EPS15,EREG,GAB1,EPGN,LRIG1,GRB2,EPN1,SH3KBP1,HRAS,AREG,KRAS,NRAS,PIK3CA,PIK3R1,PLCG1,PAG1,PTPN3,PTPN11,PTPN12,PTPRK,PXN,EPS15L1,RPS27A,SH3GL1,SH3GL2,SH3GL3,SHC1,SOS1,SRC,BTC,ADAM17,TGFA,UBA52,UBB,UBC,STAM,ADAM12,CBL,ARHGEF7,HGS,CDC42",Signaling by EGFR,51

R-HSA-1793185,"GPC6,UST,CSPG5,B4GALT7,CHST14,B3GALT6,B3GAT2,VCAN,NCAN,CSPG4,DCN,CHST13,GPC2,GPC4,GPC5,CHSY1,B3GAT3,B3GAT1,GPC3,GPC1,DSE,HEXA,HEXB,HSPG2,HYAL1,CHSY3,IDS,IDUA,AGRN,ARSB,CHST11,CHST15,CHPF2,CSGALNACT2,CHST12,CSGALNACT1,CHST7,BGN,SDC1,BCAN,SDC2,SDC4,XYLT1,XYLT2,CHPF,CHST9,HYAL3,DSEL,CHST3,SDC3",Chondroitin sulfate/dermatan sulfate metabolism,50

R-HSA-179409,"ANAPC10,UBE2C,ANAPC16,CDC26,ANAPC15,UBE2S,ANAPC2,ANAPC4,MAD2L1,NEK2,ANAPC5,ANAPC7,ANAPC11,RPS27A,ANAPC1,BUB1B,UBA52,UBB,UBC,UBE2D1,UBE2E1,CDC23,CDC16,BUB3,CDC20,CDC27",APC-Cdc20 mediated degradation of Nek2A,26

R-HSA-179419,"PSME3,PSMD14,ANAPC10,UBE2C,ANAPC16,PSMB11,PSMA8,PSME4,CDC26,ANAPC15,UBE2S,ANAPC2,ANAPC4,MAD2L1,NEK2,ANAPC5,ANAPC7,ANAPC11,PSMA1,PSMA2,PSMA3,PSMA4,PSMA5,PSMA6,PSMA7,PSMB1,PSMB2,PSMB3,PSMB4,PSMB5,PSMB6,PSMB7,PSMB8,PSMB9,PSMB10,PSMC1,PSMC2,PSMC3,PSMC4,PSMC5,PSMC6,PSMD1,PSMD2,PSMD3,PSMD4,PSMD5,PSMD7,PSMD8,PSMD9,PSMD10,PSMD11,PSMD12,PSMD13,PSME1,PSME2,RPS27A,ANAPC1,BUB1B,UBA52,UBB,UBC,UBE2D1,UBE2E1,SEM1,CDC23,CDC16,CCNA2,CCNA1,BUB3,PSMF1,CDK1,PSMD6,CDC20,CDC27",APC:Cdc20 mediated degradation of cell cycle proteins prior to satisfation of the cell cycle checkpoint,74

R-HSA-179812,"HBEGF,EGF,EGFR,EREG,EPGN,GRB2,HRAS,AREG,KRAS,NRAS,SOS1,BTC,TGFA",GRB2 events in EGFR signaling,13

R-HSA-1799339,"SEC61B,RPL35,RPL35,RPL39L,RPL39L,RPS4Y2,RPS4Y2,RPL10L,RPL10L,DDOST,RPL22L1,RPL22L1,TRAM1,SEC11A,SEC61G,RPL13A,RPL13A,RPL36,RPL36,SPCS1,SEC61A1,RPSA,RPSA,RPL10A,RPL10A,RPS27L,RPS27L,RPL26L1,RPL26L1,SEC61A2,SRPRB,SPCS3,RPL3,RPL3,RPL3L,RPL3L,RPL4,RPL4,RPL5,RPL5,RPL6,RPL6,RPL7,RPL7,RPL7A,RPL7A,RPL8,RPL8,RPL9,RPL9,RPL10,RPL10,RPL11,RPL11,RPL12,RPL12,RPL13,RPL13,RPL15,RPL15,RPL17,RPL17,RPL18,RPL18,RPL18A,RPL18A,RPL19,RPL19,RPL21,RPL21,RPL22,RPL22,RPL23A,RPL23A,RPL24,RPL24,RPL26,RPL26,RPL27,RPL27,RPL30,RPL30,RPL27A,RPL27A,RPL28,RPL28,RPL29,RPL29,RPL31,RPL31,RPL32,RPL32,RPL34,RPL34,RPL35A,RPL35A,RPL36AL,RPL36AL,RPL37,RPL37,RPL37A,RPL37A,RPL38,RPL38,RPL39,RPL39,RPL41,RPL41,RPL36A,RPL36A,RPLP0,RPLP0,RPLP1,RPLP1,RPLP2,RPLP2,RPN1,RPN2,RPS2,RPS2,RPS3,RPS3,RPS3A,RPS3A,RPS4X,RPS4X,RPS4Y1,RPS4Y1,RPS5,RPS5,RPS6,RPS6,RPS7,RPS7,RPS8,RPS8,RPS9,RPS9,RPS10,RPS10,RPS11,RPS11,RPS12,RPS12,RPS13,RPS13,RPS14,RPS14,RPS15,RPS15,RPS15A,RPS15A,RPS16,RPS16,RPS17,RPS17,RPS18,RPS18,RPS19,RPS19,RPS20,RPS20,RPS21,RPS21,RPS23,RPS23,RPS24,RPS24,RPS25,RPS25,RPS26,RPS26,RPS27,RPS27,RPS27A,RPS27A,RPS28,RPS28,RPS29,RPS29,SRP9,SRP9,SRP14,SRP14,SRP19,SRP19,SRP54,SRP54,SRP68,SRP68,SRP72,SRP72,SRPRA,SSR1,SSR2,SSR3,SSR4,UBA52,UBA52,RPL14,RPL14,SEC11C,RPL23,RPL23,SPCS2",SRP-dependent cotranslational protein targeting to membrane,205

R-HSA-180024,"CDK5,PDE4A,PDE4B,PDE4C,PDE4D,PPP1CA,PPP2CA,PPP2CB,PPP2R1A,PPP2R1B,PPP2R5D,PPP3CA,PPP3CB,PPP3CC,PPP3R1,PRKACA,PRKACB,PRKACG,PRKAR1A,PRKAR1B,PRKAR2A,PRKAR2B,CALM1,CALM2,CALM3,PPP1R1B,PPP1R1B",DARPP-32 events,27

R-HSA-180292,"CSK,HBEGF,EGF,EGFR,EREG,GAB1,EPGN,GRB2,AREG,PIK3CA,PIK3R1,PAG1,PTPN11,PXN,SRC,BTC,TGFA",GAB1 signalosome,17

R-HSA-180336,"HBEGF,EGF,EGFR,EREG,EPGN,GRB2,HRAS,AREG,KRAS,NRAS,SHC1,SOS1,BTC,TGFA",SHC1 events in EGFR signaling,14

R-HSA-180534,"PSME3,PSMD14,PSMB11,PSMA8,PSME4,PSMA1,PSMA2,PSMA3,PSMA4,PSMA5,PSMA6,PSMA7,PSMB1,PSMB2,PSMB3,PSMB4,PSMB5,PSMB6,PSMB7,PSMB8,PSMB9,PSMB10,PSMC1,PSMC2,PSMC3,PSMC4,PSMC5,PSMC6,PSMD1,PSMD2,PSMD3,PSMD4,PSMD5,PSMD7,PSMD8,PSMD9,PSMD10,PSMD11,PSMD12,PSMD13,PSME1,PSME2,RPS27A,SKP1,UBA52,UBB,UBC,SEM1,BTRC,CD4,PSMF1,PSMD6",Vpu mediated degradation of CD4,52

R-HSA-180585,"PSME3,PSMD14,PSMB11,PSMA8,PSME4,PSMA1,PSMA2,PSMA3,PSMA4,PSMA5,PSMA6,PSMA7,PSMB1,PSMB2,PSMB3,PSMB4,PSMB5,PSMB6,PSMB7,PSMB8,PSMB9,PSMB10,PSMC1,PSMC2,PSMC3,PSMC4,PSMC5,PSMC6,PSMD1,PSMD2,PSMD3,PSMD4,PSMD5,PSMD7,PSMD8,PSMD9,PSMD10,PSMD11,PSMD12,PSMD13,PSME1,PSME2,APOBEC3G,RPS27A,ELOC,ELOB,UBA52,UBB,UBC,SEM1,CUL5,PSMF1,PSMD6,RBX1",Vif-mediated degradation of APOBEC3G,54

R-HSA-180689,"PSIP1,HMGA1,PPIA,APOBEC3G,BANF1",APOBEC3G mediated resistance to HIV-1 infection,5

R-HSA-180746,"NUP50,RCC1,NUP42,NUP35,NUP205,NUP210,NUP160,NUP188,NUP62,NUP43,KPNB1,KPNB1,NPM1,NPM1,NUP88,NUP98,NUP54,NDC1,NUP133,NUP107,RAN,RANBP2,SEC13,TPR,NUP37,NUP85,NUP214,AAAS,SEH1L,RAE1,NUP155,NUP93,NUP58,POM121,NUP153",Nuclear import of Rev protein,35

R-HSA-180786,"TEN1,CDK2,POLD3,RUVBL2,DKC1,DNA2,FEN1,ANKRD28,POLA2,POT1,TINF2,LIG1,PCNA,RTEL1,POLA1,POLD1,POLD2,TERF2IP,GAR1,CHTF8,WRAP53,SHQ1,PPP6R3,PPP6C,NOP10,PRIM1,PRIM2,NHP2,POLD4,RFC1,RFC2,RFC3,RFC4,RFC5,RPA1,RPA2,RPA3,CHTF18,BLM,ACD,TERF1,TERF2,TERT,WRN,DSCC1,STN1,PIF1,CTC1,RUVBL1,CCNA2,CCNA1",Extension of Telomeres,51

R-HSA-180897,"SLC25A4,SLC25A5,SLC25A6",Vpr-mediated induction of apoptosis by mitochondrial outer membrane permeabilization,3

R-HSA-180910,"NUP50,NUP42,PSIP1,NUP35,NUP205,NUP210,NUP160,NUP188,NUP62,HMGA1,NUP43,KPNA1,NUP88,NUP98,NUP54,NDC1,NUP133,NUP107,RANBP2,SEC13,TPR,NUP37,NUP85,NUP214,AAAS,SEH1L,RAE1,BANF1,NUP155,NUP93,NUP58,POM121,NUP153",Vpr-mediated nuclear import of PICs,33

R-HSA-1810476,"RIPK3,CHUK,TICAM1,DHX9,NKIRAS2,NKIRAS1,IKBKB,MYD88,NFKB1,NFKB2,NFKBIA,NFKBIB,RELA,TLR3,ZBP1,IKBKG,RIPK1",RIP-mediated NFkB activation via ZBP1,17

R-HSA-181429,"UNC13B,CPLX1,RIMS1,RAB3A,SLC18A2,SNAP25,STX1A,STXBP1,VAMP2,SYN1,SYN2,SYT1,SYN3,PPFIA4,PPFIA2,PPFIA1,PPFIA3,TSPOAP1",Serotonin Neurotransmitter Release Cycle,18

R-HSA-181430,"UNC13B,CPLX1,RIMS1,MAOA,RAB3A,SLC18A2,SLC22A1,SLC22A2,SNAP25,STX1A,STXBP1,VAMP2,SYT1,PPFIA4,PPFIA2,PPFIA1,PPFIA3,TSPOAP1",Norepinephrine Neurotransmitter Release Cycle,18

R-HSA-181431,"CHRNA1,CHRNA2,CHRNA3,CHRNA4,CHRNA5,CHRNA7,CHRNB2,CHRNB3,CHRNB4,CHRND,CHRNE,CHRNG,CHRNA9,CHRNA6,CHRFAM7A",Acetylcholine binding and downstream events,15

R-HSA-181438,"TLR6,NOD1,TAB1,IRAK3,TIRAP,CHUK,MAP3K8,MAP3K8,CREB1,ATF2,MAPK14,AGER,DUSP3,DUSP4,DUSP6,DUSP7,ELK1,TAB2,FBXW11,FOS,LY96,PELI3,TAB3,NKIRAS2,NKIRAS1,HMGB1,APP,IKBKB,IRAK1,IRAK2,JUN,TMEM189-UBE2V1,MEF2A,MEF2C,MAP3K1,MYD88,ATF1,NFKB1,NFKB2,NFKBIA,NFKBIB,IRAK4,VRK3,ECSIT,PPP2CA,PPP2CB,PPP2R1A,PPP2R1B,PPP2R5D,MAPK1,MAPK1,MAPK3,MAPK3,MAPK7,MAPK7,MAPK8,MAPK11,MAPK9,MAPK10,MAP2K1,MAP2K3,MAP2K6,MAP2K7,PELI2,PELI1,SIGIRR,RELA,RPS6KA1,RPS6KA2,RPS6KA3,RPS27A,S100A12,S100B,SAA1,NOD2,MAP2K4,MAP2K4,SFTPD,SKP1,SFTPA1,MAP3K7,BTK,TLR1,TLR2,TLR4,TRAF6,SFTPA2,UBA52,UBB,UBC,UBE2N,UBE2V1,MAPKAPK3,TNIP2,CUL1,IKBKG,SOCS1,RIPK2,BTRC,RPS6KA5,MAPKAPK2,CD14,CD36",Toll Like Receptor 2 (TLR2) Cascade,103

R-HSA-182218,"AP2M1,AP2S1,AP2A1,AP2A2,AP2B1,ATP6V1H,CD8B",Nef Mediated CD8 Down-regulation,7

R-HSA-182971,"SPRY1,SPRY2,STAM2,HBEGF,EGF,EGFR,EPS15,EREG,EPGN,GRB2,EPN1,SH3KBP1,AREG,PTPN3,PTPN12,PTPRK,EPS15L1,RPS27A,SH3GL1,SH3GL2,SH3GL3,BTC,TGFA,UBA52,UBB,UBC,STAM,CBL,ARHGEF7,HGS,CDC42",EGFR downregulation,31

R-HSA-1834941,"TREX1,CGAS,NLRP4,NLRC3,DTX4,XRCC6,TBK1,STING1,IFI16,IRF3,MRE11,DDX41,PRKDC,TRIM21,STAT6,XRCC5",STING mediated induction of host immune responses,16

R-HSA-1834949,"POLR3F,POLR3G,POLR3C,RIPK3,POLR3A,TREX1,CHUK,CGAS,CREBBP,NLRP4,TICAM1,CTNNB1,DHX9,DHX36,POLR3H,NLRC3,EP300,TRIM32,DTX4,XRCC6,CRCP,NKIRAS2,NKIRAS1,TBK1,TBK1,STING1,IFI16,IKBKB,IRF3,IRF3,IRF7,MRE11,MYD88,NFKB1,NFKB2,NFKBIA,NFKBIB,POLR1D,DDX41,POLR3K,POLR2E,POLR2F,POLR2H,POLR2K,POLR2L,POLR3B,POLR3E,PRKDC,RELA,RPS27A,POLR3D,TRIM21,STAT6,TLR3,UBA52,UBB,UBC,XRCC5,ZBP1,ZBP1,TRIM56,POLR3GL,IKBKG,RIPK1,LRRFIP1,POLR1C",Cytosolic sensors of pathogen-associated DNA ,66

R-HSA-1839117,"CPSF6,CNTRL,FGFR1OP,CUX1,FGFR1OP2,GRB2,MYO18A,PIK3CA,PIK3R1,BCR,STAT1,STAT3,STAT5A,STAT5B,ZMYM2,TRIM24,LRRFIP1,GAB2",Signaling by cytosolic FGFR1 fusion mutants,18

R-HSA-1839120,FGFR1,Signaling by FGFR1 amplification mutants,1

R-HSA-1839122,"FGF1,FGF2,FGF4,FGF5,FGF6,FGF8,FGF9,FGFR1,FGF20,FGF23,FGF17",Signaling by activated point mutants of FGFR1,11

R-HSA-1839124,"CPSF6,CNTRL,FGFR1OP,ERLIN2,CUX1,FGF1,FGF2,FGF4,FGF5,FGF6,FGF8,FGF9,FGFR1,FGFR1OP2,FGF20,GRB2,MYO18A,PIK3CA,PIK3R1,BCR,STAT1,STAT3,STAT5A,STAT5B,ZMYM2,FGF23,TRIM24,FGF17,LRRFIP1,BAG4,GAB2",FGFR1 mutant receptor activation,31

R-HSA-1839126,"FGF1,FGF1,FGF2,FGF2,FGF3,FGF4,FGF5,FGF6,FGF7,FGF8,FGF9,FGF10,FGFR2,FGFR2,NCBP2,FGF20,FGF22,GTF2F1,GTF2F2,NCBP1,POLR2A,POLR2B,POLR2C,POLR2D,POLR2E,POLR2F,POLR2G,POLR2H,POLR2I,POLR2J,POLR2K,POLR2L,FGF23,FGF18,FGF17,FGF16",FGFR2 mutant receptor activation,36

R-HSA-1839128,FGFR4,FGFR4 mutant receptor activation,1

R-HSA-1839130,"FGF1,FGF2,FGF4,FGF5,FGF8,FGF9,FGFR3,FGF20,FGF23,FGF18,FGF17,FGF16",Signaling by activated point mutants of FGFR3,12

R-HSA-1852241,"HDAC6,ACTR1A,AKAP9,TPTEP2-CSNK1E,TUBA1B,TUBB3,TUBB4A,TUBB4B,ATP5PD,CARM1,NCOA2,DCTN2,CCT4,CCT2,ATP5MG,EXOC5,MTX2,CETN2,CCT8,PLK4,SDCCAG8,PPARGC1A,PPARGC1A,IMMT,IFT27,IFT27,CNTRL,FGFR1OP,KIF3A,CEP250,POLG2,DCTN3,TUBA3E,IFT43,EXOC3,TUBA3D,HAUS1,RAB3IP,NEDD1,TTC8,CNGB1,CNGB1,MICOS13,CNGA2,CNGA2,CNGA4,CNGA4,BBS5,SCLT1,PPARGC1B,PPARGC1B,CREB1,CREB1,ATF2,CREBBP,APOOL,DYNLL2,MAPK14,CSNK1D,CSNK1E,TTBK2,EXOC8,TTC30B,TTC30B,DCTN1,BBS12,DYNC1H1,DYNC1I2,CYS1,TCTEX1D1,CRTC2,ARL13B,TUBB,ESRRA,ESRRA,ALAS1,CEP162,CEP164,MAPRE1,KIFAP3,CCT5,NINL,CEP131,CEP152,NCOA6,CLUAP1,CLUAP1,PPRC1,EXOC7,RPGRIP1L,CLASP1,HAUS5,CRTC1,SIRT5,SIRT4,SIRT3,GABPA,GABPB1,TCTEX1D2,SAMM50,C2CD3,TCTN3,TRAF3IP1,TRAF3IP1,IFT172,IFT172,NPHP4,NPHP3,B9D1,DMAC2L,BBS9,GLUD1,GLUD2,MCHR1,IFT81,IFT81,HCFC1,HSPA9,HSP90AA1,IDH2,KIF24,TUBB8,TUBB2B,ARF4,ARF4,KIF3C,TNPO1,ARL3,MEF2C,MEF2D,RAB8A,MICOS10,ATP6,ATP8,MTX1,SEPTIN2,NEK2,NPHP1,NRF1,NRF1,ODF2,ATP5F1A,PAFAH1B1,ATP5F1B,ASAP1,ASAP1,ATP5F1C,PCM1,IFT52,IFT52,TFB1M,CEP83,PCNT,TMEM216,ATP5F1D,ATP5F1E,PRKAG2,PDE6D,ATP5PB,ATP5MC1,DYNC2LI1,HSPB11,HSPB11,ATP5MC2,ATP5MC3,TUBA8,ATP5ME,ATP5PF,PKD1,PKD1,PKD2,PKD2,PLK1,PRKAG3,ATP5PO,CYCS,POLRMT,EXOC6,LZTFL1,PPARA,MED1,HAUS6,AHI1,NDE1,MKS1,CHCHD3,HAUS4,IFT57,IFT57,WDR60,CEP192,HAUS2,PPP2R1A,BBS7,HAUS7,PRKAA2,PRKAB1,PRKAB2,PRKACA,PRKAG1,CEP72,DNAJC11,CDK5RAP2,EXOC1,IFT122,PRKAR2B,EXOC2,CENPJ,ACSS2,MAPK11,INPP5E,TWNK,IFT46,IFT46,WDR35,CC2D2A,IFT80,IFT80,KIF17,WDR19,MARK4,BBS1,BBS2,BBS4,RHO,RHO,EXOC4,RP2,RXRA,MAPK12,TFB2M,CRTC3,IFT22,IFT22,SMARCD3,SMO,SOD2,SSBP1,SSTR3,TBL1X,TCP1,TCTE3,TFAM,TFAM,CCT3,TUBA4A,TUBA3C,TUBB2A,TUBG1,YWHAE,YWHAG,ALMS1,TUBA1A,APOO,HAUS3,CEP97,TCTN1,DYNC2H1,TBL1XR1,BBS10,MTERF1,TTC21B,TUBAL3,TCTN2,CEP76,ATAT1,TTC26,TTC26,CALM1,IFT74,IFT74,CEP290,CHD9,CEP63,CEP70,CALM2,B9D2,CALM3,IFT88,IFT88,TUBB1,CAMK4,MKKS,DYNLRB2,DYNLRB1,ARL6,CEP78,CHCHD6,TUBB6,UNC119B,TUBA1C,PERM1,OFD1,CEP89,FBF1,HELZ2,SSNA1,NCOA1,DYNLL1,GBF1,RAB11A,TMEM11,HDAC3,WDR34,IFT20,IFT20,TMEM67,TTC30A,TTC30A,BBIP1,TRIP11,HAUS8,KIF3B,ATP5MF,CEP41,NR1D1,NCOR1,IQCB1,CEP135,TGS1,CEP57,RAB11FIP3,CCP110,IFT140,CKAP5,SFI1,CDK1",Organelle biogenesis and maintenance,327

R-HSA-1855167,"NUDT4,NUDT3,IP6K3,NUDT10,PPIP5K2,ITPK1,NUDT11,IPPK,PPIP5K1,IP6K1",Synthesis of pyrophosphates in the cytosol,10

R-HSA-1855183,"INPP5J,IMPA1,IMPA2,INPP1,INPP4A,INPP5A,INPP5B,OCRL,ISYNA1,MIOX,MTMR9,INPP4B,SYNJ1,MTMR7","Synthesis of IP2, IP, and Ins in the cytosol",14

R-HSA-1855191,"IPMK,IP6K2,IPPK,IP6K1",Synthesis of IPs in the nucleus,4

R-HSA-1855204,"PLCD3,PLD4,PLCH1,PLCB1,INPP5J,INPP5B,INPP5D,INPPL1,ITPK1,ITPKA,ITPKB,OCRL,PLCE1,PLCB2,PLCB3,PLCB4,PLCD1,PLCG1,PLCG2,PTEN,CALM1,ITPKC,CALM2,CALM3,PLCD4,SYNJ1,PLCZ1,PLCH2",Synthesis of IP3 and IP4 in the cytosol,28

R-HSA-1855231,MINPP1,Synthesis of IPs in the ER lumen,1

R-HSA-186712,"AKT3,MAMLD1,CREBBP,EP300,AKT1,AKT2,RFX6,FGF10,SNW1,FOXO1,FOXO1,NR5A2,PTF1A,GCK,KAT2A,FOXA2,FOXA3,HNF4A,HNF4G,ONECUT1,HES1,IAPP,RBPJ,INS,INSM1,PDX1,MAFA,ONECUT3,NEUROD1,NEUROD1,NKX2-2,NKX2-2,NKX6-1,NOTCH1,NEUROG3,NEUROG3,PAX6,PKLR,MAML3,SLC2A2,HNF1A,HNF1B,MAML2,KAT2B,MAML1",Regulation of beta-cell development,45

R-HSA-186763,"CRK,CRKL,GRB2,GRB7,RAPGEF1,HRAS,KRAS,NCK1,NRAS,PDGFA,PDGFB,PDGFRA,PDGFRB,PIK3CA,PIK3CB,PIK3R1,PIK3R2,PLCG1,PTPN11,RASA1,SOS1,SRC,STAT1,STAT3,STAT5A,STAT5B,STAT6,NCK2,BCAR1",Downstream signal transduction,29

R-HSA-186797,"COL2A1,COL3A1,COL4A1,COL4A2,COL4A3,COL4A4,COL4A5,COL5A1,COL5A2,COL6A1,COL6A2,COL6A3,COL9A1,COL9A2,COL9A3,COL6A6,CRK,CRKL,COL6A5,GRB2,GRB7,RAPGEF1,HRAS,KRAS,NCK1,NRAS,FURIN,COL5A3,PDGFA,PDGFB,PDGFRA,PDGFRB,PIK3CA,PIK3CB,PIK3R1,PIK3R2,PLAT,PLCG1,PLG,PDGFC,PTPN11,PTPN12,RASA1,SOS1,SPP1,SRC,STAT1,STAT3,STAT5A,STAT5B,STAT6,THBS1,THBS2,THBS3,THBS4,PDGFD,NCK2,BCAR1",Signaling by PDGF,58

R-HSA-187015,"ADCYAP1,ADCYAP1R1,ADORA2A,NGF,NGF,NTRK1,NTRK1,NTRK2",Activation of TRKA receptors,8

R-HSA-187024,"ADCYAP1,ADCYAP1R1,ADORA2A,NTRK1,NTRK2",NGF-independant TRKA activation,5

R-HSA-187037,"DNAL4,CDK5,TRIB1,FRS2,FRS2,CHD4,ADCYAP1,ADCYAP1R1,AP2M1,AP2M1,AP2S1,AP2S1,CLTA,CLTA,CLTC,CLTC,ADORA2A,CREB1,CREB1,ATF2,ATF2,CRK,CRK,CRKL,CRKL,MAPK14,MAPK14,AP2A1,AP2A1,AP2A2,AP2A2,AP2B1,AP2B1,DNM1,DNM2,DUSP3,DUSP4,DUSP6,DUSP7,EGR1,EGR1,EGR2,EGR2,EGR3,EGR3,EGR4,ELK1,ELK1,EP300,EP300,F3,ARC,ARC,FOS,FOSB,SHC2,SHC2,DNM3,GRB2,GRB2,RAPGEF1,RAPGEF1,HRAS,HRAS,ID1,ID2,ID3,ID4,IRS1,IRS1,JUNB,JUND,KRAS,KRAS,RHOA,LYL1,MEF2A,MEF2C,MEF2D,MEF2D,ASCL1,ASCL1,ATF1,ATF1,NAB1,NAB2,NAB2,NGF,NGF,NRAS,NRAS,NTRK1,NTRK1,NTRK2,VRK3,PIK3CA,PIK3CB,PIK3R1,PIK3R2,PLCG1,SHC3,SHC3,PPP2CA,PPP2CB,PPP2R1A,PPP2R1B,PPP2R5D,MAPK1,MAPK1,MAPK3,MAPK3,MAPK7,MAPK7,MAPK11,MAPK11,MAPK13,MAP2K1,MAP2K2,MAP2K5,KIDINS220,KIDINS220,RALA,RALB,RALGDS,RALGDS,RAP1A,RAP1A,REST,RIT2,RIT1,RPS6KA1,RPS6KA2,RPS6KA3,RRAD,MAPK12,SGK1,SH3GL2,SH3GL2,SH3GL3,SHC1,SHC1,SOS1,SOS1,SRC,SRF,SRF,BRAF,BRAF,STAT3,TCF12,TCF12,TPH1,VGF,YWHAB,YWHAB,MAPKAPK3,FOSL1,IRS2,IRS2,CDK5R1,CDK5R1,CDK5R2,RPS6KA5,MAPKAPK2",Signaling by NTRK1 (TRKA),164

R-HSA-187042,"NGF,NGF,NTRK1,NTRK1",TRKA activation by NGF,4

R-HSA-187577,"CDK2,CDK4,PSME3,PSMD14,CDKN1A,CDKN1B,CKS1B,PSMA1,PSMA2,PSMA3,PSMA4,PSMA5,PSMA6,PSMA7,PSMB1,PSMB2,PSMB3,PSMB4,PSMB5,PSMB6,PSMB7,PSMB8,PSMB9,PSMB10,PSMC1,PSMC2,PSMC3,PSMC4,PSMC5,PSMC6,PSMD1,PSMD2,PSMD3,PSMD4,PSMD5,PSMD7,PSMD8,PSMD9,PSMD10,PSMD11,PSMD12,PSMD13,PSME1,PSME2,PTK6,CCND1,RPS27A,SKP1,SKP2,UBA52,UBB,UBC,SEM1,CUL1,CCNA2,CCNA1,CCNE1,CCNE2,PSMF1,PSMD6",SCF(Skp2)-mediated degradation of p27/p21,60

R-HSA-187687,"FRS2,FRS2,CRK,CRK,CRKL,CRKL,MAPK14,MAPK14,SHC2,SHC2,GRB2,GRB2,RAPGEF1,RAPGEF1,HRAS,HRAS,KRAS,KRAS,NGF,NGF,NRAS,NRAS,NTRK1,NTRK1,SHC3,SHC3,MAPK1,MAPK3,MAPK11,MAPK11,MAPK13,MAP2K1,MAP2K2,KIDINS220,KIDINS220,RALA,RALB,RALGDS,RALGDS,RAP1A,RAP1A,RIT2,RIT1,MAPK12,SHC1,SHC1,SOS1,SOS1,SRC,BRAF,BRAF,YWHAB,YWHAB,MAPKAPK3,MAPKAPK2",Signalling to ERKs,55

R-HSA-187706,"NGF,NTRK1,RIT2,RIT1,BRAF",Signalling to p38 via RIT and RIN,5

R-HSA-189085,"CHIT1,TREH,CHIA,AMY1A,AMY1B,AMY1C,AMY2A,AMY2B,LCT,SI,MGAM",Digestion of dietary carbohydrate,11

R-HSA-189200,"SLC2A6,SLC5A10,SLC2A14,SLC2A12,SLC2A7,SLC5A9,FGF21,SLC2A8,SLC50A1,SLC2A9,SLC2A1,SLC2A2,SLC2A3,SLC2A4,SLC5A1,SLC5A2,SLC5A4,SLC2A11,SLC2A10,SLC45A3,MFSD4B",Cellular hexose transport,21

R-HSA-189445,"SLCO1B1,SLCO2B1,ABCC2,COX10,COX15,CPOX,ALAD,ALAS1,ALAS2,ALB,FABP1,FECH,SLCO1B3,FLVCR1,GSTA1,HMBS,HMOX1,HMOX2,ABCC1,UGT1A4,PPOX,BLVRA,BLVRB,UROD,UROS,ABCG2",Metabolism of porphyrins,26

R-HSA-189451,"COX10,COX15,CPOX,ALAD,ALAS1,ALAS2,ALB,FECH,FLVCR1,HMBS,PPOX,UROD,UROS,ABCG2",Heme biosynthesis,14

R-HSA-189483,"SLCO1B1,SLCO2B1,ABCC2,ALB,FABP1,SLCO1B3,GSTA1,HMOX1,HMOX2,ABCC1,UGT1A4,BLVRA,BLVRB,ABCG2",Heme degradation,14

R-HSA-190236,"SPRY2,FRS3,FRS2,FGFBP3,KLB,SPRED1,SPRED2,FGF1,FGF2,FGF3,FGF4,FGF5,FGF6,FGF7,FGF8,FGF9,FGF10,FGFR1,FGFR3,FGFR2,FGFR4,NCBP2,RBFOX2,FLRT3,FLRT2,FLRT1,GAB1,GALNT3,FGF20,FGF22,GRB2,GTF2F1,GTF2F2,HNRNPA1,HNRNPF,HNRNPH1,HRAS,ANOS1,KRAS,HNRNPM,NCBP1,NRAS,PIK3CA,PIK3R1,PLCG1,FGFRL1,POLR2A,POLR2B,POLR2C,POLR2D,POLR2E,POLR2F,POLR2G,POLR2H,POLR2I,POLR2J,POLR2K,POLR2L,ESRP1,PPP2CA,PPP2CB,PPP2R1A,MAPK1,MAPK3,PTBP1,PTPN11,RPS27A,SHC1,SOS1,SRC,BRAF,TIA1,TIAL1,UBA52,UBB,UBC,ESRP2,FGF23,FGFBP2,MKNK1,CBL,FGF18,FGF17,FGF16,KL,FGF19,FGFBP1",Signaling by FGFR,87

R-HSA-190239,"FGF1,FGF2,FGF4,FGF5,FGF8,FGF9,FGFR3,GALNT3,FGF20,FGF23,FGF18,FGF17,FGF16",FGFR3 ligand binding and activation,13

R-HSA-190241,"FGFBP3,FGF1,FGF2,FGF3,FGF4,FGF5,FGF6,FGF7,FGF8,FGF9,FGF10,FGFR2,FGF20,FGF22,FGF23,FGFBP2,FGF18,FGF17,FGF16,FGFBP1",FGFR2 ligand binding and activation,20

R-HSA-190242,"FGF1,FGF2,FGF3,FGF4,FGF5,FGF6,FGF8,FGF9,FGF10,FGFR1,FGF20,FGF22,ANOS1,FGF23,FGF17,KL",FGFR1 ligand binding and activation,16

R-HSA-190322,"KLB,FGF1,FGF2,FGF4,FGF6,FGF8,FGF9,FGFR4,FGF20,FGF23,FGF18,FGF17,FGF16,FGF19",FGFR4 ligand binding and activation,14

R-HSA-190370,"FGF1,FGF2,FGF3,FGF10,FGFR1,FGF22",FGFR1b ligand binding and activation,6

R-HSA-190371,"FGF1,FGF8,FGF9,FGFR3,FGF20,FGF18,FGF17",FGFR3b ligand binding and activation,7

R-HSA-190372,"FGF1,FGF2,FGF4,FGF5,FGF8,FGF9,FGFR3,GALNT3,FGF20,FGF23,FGF18,FGF17,FGF16",FGFR3c ligand binding and activation,13

R-HSA-190373,"FGF1,FGF2,FGF4,FGF5,FGF6,FGF8,FGF9,FGFR1,FGF20,ANOS1,FGF23,FGF17",FGFR1c ligand binding and activation,12

R-HSA-190374,"FGFR1,FGF23,KL",FGFR1c and Klotho ligand binding and activation,3

R-HSA-190375,"FGF1,FGF2,FGF4,FGF5,FGF6,FGF8,FGF9,FGFR2,FGF20,FGF23,FGF18,FGF17,FGF16",FGFR2c ligand binding and activation,13

R-HSA-190377,"FGFBP3,FGF1,FGF2,FGF3,FGF7,FGF10,FGFR2,FGF22,FGFBP2,FGFBP1",FGFR2b ligand binding and activation,10

R-HSA-190704,"GJA1,GJB1,GJB1,GJB2",Oligomerization of connexins into connexons,4

R-HSA-190827,"GJA1,GJB1,GJB2",Transport of connexins along the secretory pathway,3

R-HSA-190828,"GJC1,TUBA1B,TUBA1B,TUBB3,TUBB3,TUBB4A,TUBB4A,TUBB4B,TUBB4B,GJB6,TUBA3E,TUBA3E,TUBA3D,TUBA3D,AP2M1,CLTA,CLTB,CLTC,GJD3,GJB4,DAB2,DNM1,DNM2,GJD4,GJA1,GJA1,GJA3,GJA4,GJA5,GJA8,GJB1,GJB1,GJB2,GJB2,GJB3,GJB5,TUBB8,TUBB8,TUBB2B,TUBB2B,GJB7,MYO6,TUBA8,TUBA8,GJC2,GJD2,ACTB,ACTG1,TUBA4A,TUBA4A,TUBA3C,TUBA3C,TUBB2A,TUBB2A,TUBA1A,TUBA1A,TUBAL3,TUBAL3,GJA9,TUBB1,TUBB1,CLTCL1,TUBB6,TUBB6,GJA10,TUBA1C,TUBA1C",Gap junction trafficking,67

R-HSA-190840,"TUBA1B,TUBB3,TUBB4A,TUBB4B,TUBA3E,TUBA3D,GJA1,TUBB8,TUBB2B,TUBA8,TUBA4A,TUBA3C,TUBB2A,TUBA1A,TUBAL3,TUBB1,TUBB6,TUBA1C",Microtubule-dependent trafficking of connexons from Golgi to the plasma membrane,18

R-HSA-190861,"GJC1,TUBA1B,TUBA1B,TUBB3,TUBB3,TUBB4A,TUBB4A,TUBB4B,TUBB4B,GJB6,TUBA3E,TUBA3E,TUBA3D,TUBA3D,GJD3,GJB4,GJD4,GJA1,GJA1,GJA3,GJA4,GJA5,GJA8,GJB1,GJB1,GJB2,GJB2,GJB3,GJB5,TUBB8,TUBB8,TUBB2B,TUBB2B,GJB7,TUBA8,TUBA8,GJC2,GJD2,TUBA4A,TUBA4A,TUBA3C,TUBA3C,TUBB2A,TUBB2A,TUBA1A,TUBA1A,TUBAL3,TUBAL3,GJA9,TUBB1,TUBB1,TUBB6,TUBB6,GJA10,TUBA1C,TUBA1C",Gap junction assembly,56

R-HSA-190872,"TUBA1B,TUBB3,TUBB4A,TUBB4B,TUBA3E,TUBA3D,GJA1,GJB2,TUBB8,TUBB2B,TUBA8,TUBA4A,TUBA3C,TUBB2A,TUBA1A,TUBAL3,TUBB1,TUBB6,TUBA1C",Transport of connexons to the plasma membrane,19

R-HSA-190873,"AP2M1,CLTA,CLTB,CLTC,DAB2,DNM1,DNM2,GJA1,MYO6,ACTB,ACTG1,CLTCL1",Gap junction degradation,12

R-HSA-1912399,"POFUT1,NOTCH1,NOTCH2,NOTCH3,NOTCH4,POGLUT1",Pre-NOTCH Processing in the Endoplasmic Reticulum,6

R-HSA-1912408,"MAMLD1,MAMLD1,LOC102724334,H4-16,H3C14,H2BU1,CREBBP,CREBBP,E2F1,E2F1,E2F3,E2F3,AGO3,AGO4,ELF3,EP300,EP300,SNW1,SNW1,TNRC6B,H2BC1,KAT2A,KAT2A,AGO1,AGO2,TNRC6A,H2AC8,H2AC7,H2AX,H2AZ1,H2BC5,H2BC3,H3-3A,H3-3B,H3C15,RBPJ,RBPJ,JUN,MOV10,H2AB1,NOTCH1,NOTCH1,NOTCH2,NOTCH3,NOTCH4,SIRT6,H4C15,MAML3,MAML3,H2AJ,PRKCI,TNRC6C,CCND1,H3C13,TFDP1,TFDP1,TFDP2,TFDP2,TP53,H2AC19,H4C9,H2AC14,H2AC6,H2AC4,H2AC18,H2AC20,H2BC8,H2BC13,H2BC15,H2BC14,H2BC7,H2BC6,H2BC9,H2BC10,H2BC4,H2BC17,H2BC21,H3C1,H3C4,H3C3,H3C6,H3C11,H3C8,H3C12,H3C10,H3C2,H4C1,H4C4,H4C6,H4C12,H4C11,H4C3,H4C8,H4C2,H4C5,H4C13,H4C14,MAML2,MAML2,H2BC12,RUNX1,KAT2B,KAT2B,H3C7,H2BC11,H2AZ2,MAML1,MAML1",Pre-NOTCH Transcription and Translation,108

R-HSA-1912420,"ST3GAL6,TMED2,B4GALT1,LFNG,MFNG,NOTCH1,NOTCH1,NOTCH2,NOTCH2,NOTCH3,NOTCH3,NOTCH4,NOTCH4,ATP2A1,ATP2A2,ATP2A3,FURIN,RAB6A,RFNG,SEL1L,ST3GAL4,ST3GAL3",Pre-NOTCH Processing in Golgi,22

R-HSA-1912422,"MAMLD1,MAMLD1,LOC102724334,ST3GAL6,TMED2,H4-16,H3C14,H2BU1,CREBBP,CREBBP,E2F1,E2F1,E2F3,E2F3,AGO3,AGO4,ELF3,EP300,EP300,SNW1,SNW1,TNRC6B,POFUT1,H2BC1,KAT2A,KAT2A,AGO1,B4GALT1,AGO2,TNRC6A,H2AC8,H2AC7,H2AX,H2AZ1,H2BC5,H2BC3,H3-3A,H3-3B,H3C15,RBPJ,RBPJ,JUN,LFNG,MFNG,MOV10,H2AB1,NOTCH1,NOTCH1,NOTCH2,NOTCH2,NOTCH3,NOTCH3,NOTCH4,NOTCH4,ATP2A1,ATP2A2,ATP2A3,FURIN,SIRT6,H4C15,MAML3,MAML3,H2AJ,PRKCI,POGLUT1,TNRC6C,RAB6A,CCND1,RFNG,SEL1L,ST3GAL4,ST3GAL3,H3C13,TFDP1,TFDP1,TFDP2,TFDP2,TP53,H2AC19,H4C9,H2AC14,H2AC6,H2AC4,H2AC18,H2AC20,H2BC8,H2BC13,H2BC15,H2BC14,H2BC7,H2BC6,H2BC9,H2BC10,H2BC4,H2BC17,H2BC21,H3C1,H3C4,H3C3,H3C6,H3C11,H3C8,H3C12,H3C10,H3C2,H4C1,H4C4,H4C6,H4C12,H4C11,H4C3,H4C8,H4C2,H4C5,H4C13,H4C14,MAML2,MAML2,H2BC12,RUNX1,KAT2B,KAT2B,H3C7,H2BC11,H2AZ2,MAML1,MAML1",Pre-NOTCH Expression and Processing,127

R-HSA-191273,"PMVK,EBP,CYP51A1,DHCR7,DHCR24,FDFT1,FDPS,HMGCR,HMGCS1,IDI1,ACAT2,LBR,PLPP6,LSS,MVD,MVK,NSDHL,HSD17B7,MSMO1,SC5D,ARV1,SQLE,TM7SF2,IDI2,GGPS1",Cholesterol biosynthesis,25

R-HSA-191650,"GJA1,GJA1,SRC,TJP1,TJP1",Regulation of gap junction activity,5

R-HSA-191859,"SNUPN,PRMT5,NUP50,NUP42,DDX20,CLNS1A,NUP35,NCBP2,NUP205,NUP210,NUP160,NUP188,NUP62,GEMIN5,NUP43,NCBP1,NUP88,NUP98,GEMIN4,PHAX,NUP54,GEMIN8,NDC1,NUP133,NUP107,RANBP2,SEC13,SMN1,SMN2,SNRPB,SNRPD1,SNRPD2,SNRPD3,SNRPE,SNRPF,SNRPG,TPR,NUP37,WDR77,GEMIN7,GEMIN6,NUP85,NUP214,AAAS,SEH1L,RAE1,GEMIN2,NUP155,TGS1,NUP93,NUP58,POM121,NUP153",snRNP Assembly,53

R-HSA-192105,"ACOT8,NCOA2,CYP46A1,SLC27A5,SLC27A2,AKR1C4,OSBPL1A,OSBPL6,OSBPL7,OSBPL9,CYP7A1,CYP8B1,CYP27A1,AKR1C1,AKR1C2,AMACR,OSBPL3,HSD17B4,OSBP,CYP39A1,BAAT,PTGIS,RXRA,SCP2,AKR1D1,HSD3B7,ACOX2,AKR1C3,ABCB11,NCOA1,CH25H,CYP7B1,OSBPL2,NR1H4",Synthesis of bile acids and bile salts,34

R-HSA-192456,"PNLIPRP3,CLPS,PNLIP,PNLIPRP1,PNLIPRP2,LIPF",Digestion of dietary lipid,6

R-HSA-192814,PARP1,vRNA Synthesis,1

R-HSA-192823,"RPL35,RPL39L,RPS4Y2,RPL10L,RPL22L1,RPL13A,RPL36,GRSF1,RPSA,RPL10A,RPS27L,RPL26L1,DNAJC3,RPL3,RPL3L,RPL4,RPL5,RPL6,RPL7,RPL7A,RPL8,RPL9,RPL10,RPL11,RPL12,RPL13,RPL15,RPL17,RPL18,RPL18A,RPL19,RPL21,RPL22,RPL23A,RPL24,RPL26,RPL27,RPL30,RPL27A,RPL28,RPL29,RPL31,RPL32,RPL34,RPL35A,RPL36AL,RPL37,RPL37A,RPL38,RPL39,RPL41,RPL36A,RPLP0,RPLP1,RPLP2,RPS2,RPS3,RPS3A,RPS4X,RPS4Y1,RPS5,RPS6,RPS7,RPS8,RPS9,RPS10,RPS11,RPS12,RPS13,RPS14,RPS15,RPS15A,RPS16,RPS17,RPS18,RPS19,RPS20,RPS21,RPS23,RPS24,RPS25,RPS26,RPS27,RPS27A,RPS28,RPS29,UBA52,RPL14,RPL23",Viral mRNA Translation,89

R-HSA-192905,"HSP90AA1,IPO5",vRNP Assembly,2

R-HSA-193048,"CGA,CYP17A1,HSD3B1,HSD3B2,HSD17B3,LHB,HSD17B12,POMC,SRD5A1,SRD5A2,SRD5A3",Androgen biosynthesis,11

R-HSA-193144,"CYP19A1,HSD17B1,HSD17B2,AKR1B15,HSD17B11,HSD17B14",Estrogen biosynthesis,6

R-HSA-193368,"ACOT8,NCOA2,SLC27A5,SLC27A2,AKR1C4,CYP7A1,CYP8B1,CYP27A1,AKR1C1,AKR1C2,AMACR,HSD17B4,BAAT,PTGIS,RXRA,SCP2,AKR1D1,HSD3B7,ACOX2,AKR1C3,ABCB11,NCOA1,CYP7B1,NR1H4",Synthesis of bile acids and bile salts via 7alpha-hydroxycholesterol,24

R-HSA-193634,"RHOA,ARHGDIA,MAG,MCF2,NGFR,OMG,RTN4,RTN4R,LINGO1",Axonal growth inhibition (RHOA activation),9

R-HSA-193639,"IKBKB,IRAK1,IRAK1,MYD88,MYD88,NFKB1,NFKBIA,NGF,NGF,NGFR,NGFR,PRKCI,RELA,RPS27A,TRAF6,UBA52,UBB,UBC,RIPK2,SQSTM1",p75NTR signals via NF-kB,20

R-HSA-193648,"BCL2L11,ARHGEF33,NET1,VAV3,GNA13,AKAP13,FGD4,ARHGEF19,ECT2,FGD2,FGD1,ARHGEF15,ARHGEF9,MCF2L,ARHGEF12,ARHGEF18,NGEF,ARHGEF26,TIAM2,AATF,ARHGEF16,ABR,ARHGEF37,MCF2,ARHGEF35,NGF,NGFR,ARHGEF4,ARHGEF3,ARHGEF38,ARHGEF10L,ARHGEF40,MAPK8,MAPK8,BAD,PLEKHG5,PREX1,RAC1,RASGRF2,ITSN1,PLEKHG2,SOS1,SOS2,TIAM1,TRIO,VAV1,VAV2,ARHGEF5,OBSCN,ARHGEF39,ARHGEF7,FGD3,KALRN,ARHGEF1,ARHGEF2,ARHGEF6,MAGED1,ARHGEF10,ARHGEF11,ARHGEF17",NRAGE signals death through JNK,60

R-HSA-193670,"PRDM4,HDAC1,HDAC2,NGF,NGFR,HDAC3",p75NTR negatively regulates cell cycle via SC1,6

R-HSA-193681,"NGF,NGFR,SMPD2",Ceramide signalling,3

R-HSA-193692,"NCSTN,NFKB1,NGFR,APH1A,PSENEN,PSEN1,PSEN2,RELA,ADAM17,TRAF6,APH1B",Regulated proteolysis of p75NTR,11

R-HSA-193697,"RHOA,ARHGDIA,MAG,MCF2,NGF,NGFR,OMG,RTN4,RTN4R,LINGO1",p75NTR regulates axonogenesis,10

R-HSA-193704,"BCL2L11,ARHGEF33,NET1,VAV3,GNA13,PRDM4,AKAP13,FGD4,ARHGEF19,ECT2,FGD2,FGD1,ARHGEF15,ARHGEF9,MCF2L,ARHGEF12,ARHGEF18,NCSTN,ITGB3BP,ITGB3BP,NGEF,ARHGEF26,TIAM2,AATF,BEX3,ARHGEF16,ABR,HDAC1,HDAC2,IKBKB,IRAK1,IRAK1,RHOA,ARHGEF37,ARHGDIA,MAG,MCF2,ARHGEF35,MYD88,MYD88,NFKB1,NFKBIA,NGF,NGF,NGFR,NGFR,OMG,ARHGEF4,ARHGEF3,APH1A,ARHGEF38,ARHGEF10L,ARHGEF40,PRKCI,PSENEN,MAPK8,MAPK8,PSEN1,PSEN2,RTN4,BAD,PLEKHG5,PREX1,RAC1,RASGRF2,RELA,RPS27A,ITSN1,PLEKHG2,RTN4R,SMPD2,SOS1,SOS2,ADAM17,TIAM1,TRAF6,TRAF6,TRIO,UBA52,UBB,UBC,VAV1,VAV2,YWHAE,ARHGEF5,APH1B,CASP2,CASP3,OBSCN,LINGO1,ARHGEF39,RIPK2,HDAC3,ARHGEF7,SQSTM1,FGD3,KALRN,ARHGEF1,ARHGEF2,ARHGEF6,MAGED1,ARHGEF10,ARHGEF11,ARHGEF17",p75 NTR receptor-mediated signalling,104

R-HSA-193775,"CYP46A1,SLC27A5,SLC27A2,AKR1C4,CYP8B1,CYP27A1,AKR1C1,AKR1C2,AMACR,CYP39A1,PTGIS,AKR1D1,HSD3B7,AKR1C3",Synthesis of bile acids and bile salts via 24-hydroxycholesterol,14

R-HSA-193807,"NCOA2,AKR1C4,CYP7A1,CYP8B1,CYP27A1,AKR1C1,AKR1C2,PTGIS,RXRA,AKR1D1,HSD3B7,AKR1C3,NCOA1,CYP7B1,NR1H4",Synthesis of bile acids and bile salts via 27-hydroxycholesterol,15

R-HSA-193993,"CGA,CYP11B2,CYP21A2,HSD3B1,HSD3B2,LHB",Mineralocorticoid biosynthesis,6

R-HSA-194002,"CYP11B1,CYP11B2,CYP17A1,CYP21A2,HSD3B1,HSD3B2,HSD11B1,HSD11B2,POMC,SERPINA6",Glucocorticoid biosynthesis,10

R-HSA-194068,"ACOT8,NCOA2,SLCO1B1,CYP46A1,SLC27A5,SLC27A2,AKR1C4,OSBPL1A,OSBPL6,OSBPL7,OSBPL9,CYP7A1,CYP8B1,CYP27A1,AKR1C1,AKR1C2,ALB,FABP6,AMACR,OSBPL3,SLCO1B3,HSD17B4,OSBP,CYP39A1,BAAT,PTGIS,RXRA,SCP2,SLC10A1,SLC10A2,SLCO1A2,AKR1D1,HSD3B7,STARD5,ACOX2,AKR1C3,ABCB11,NCOA1,ABCC3,CH25H,CYP7B1,OSBPL2,NR1H4",Bile acid and bile salt metabolism,43

R-HSA-194138,"AKT3,ABI1,CDH5,ABI2,WASF2,VAV3,VAV3,BAIAP2,AHCYL1,NCKAP1,WASF3,THEM4,CRK,AAMP,MAPK14,CTNNA1,CTNNB1,CTNND1,CYBA,CYBB,DOCK1,AKT1,AKT2,PTK2B,VEGFD,CYFIP1,FLT1,FLT4,MTOR,RICTOR,FYN,SHC2,CYFIP2,NCKAP1L,HRAS,HSPB1,HSP90AA1,HSP90AA1,ITGAV,ITGB3,ITPR1,ITPR2,ITPR3,JUP,KDR,KDR,KRAS,RHOA,NCF2,NCF4,NCK1,NOS3,NRAS,PAK1,PAK2,PAK3,PDPK1,PGF,PIK3CA,PIK3CA,PIK3CB,PIK3CB,PIK3R1,PIK3R1,PIK3R2,PIK3R2,PLCG1,PLCG1,PRR5,PRKACA,PRKACB,PRKACG,PRKCA,PRKCB,AXL,PRKCD,BRK1,PRKCZ,MAPK11,MAPK13,PTK2,PTK2,TRIB3,PXN,RAC1,RASA1,ACTB,ROCK1,MAPK12,ELMO2,MLST8,SHB,SHB,NCF1,SRC,SRC,ACTG1,VAV1,VAV1,VAV2,VAV2,VEGFA,VEGFA,VEGFB,VEGFC,MAPKAPK3,MAPKAP1,CALM1,CALM1,CALM2,CALM2,CALM3,CALM3,NCK2,CAV1,NRP2,NRP1,SPHK1,WASF1,SH2D2A,MAPKAPK2,ROCK2,BCAR1,BCAR1,ELMO1,CDC42",Signaling by VEGF,126

R-HSA-194306,"FLT1,KDR,NRP2,NRP1",Neurophilin interactions with VEGF and VEGFR,4

R-HSA-194313,"VEGFD,FLT1,FLT4,KDR,PGF,VEGFA,VEGFB,VEGFC",VEGF ligand-receptor interactions,8

R-HSA-194315,"ABI1,ABI1,ARHGEF33,CENPS-CORT,PMF1-BGLAP,ARPC5,ARPC5,ARPC4,ARPC4,ARPC3,ARPC3,ARPC1B,ARPC1B,ACTR3,ACTR3,ACTR2,ACTR2,ARPC2,ARPC2,FAM13A,ABI2,ABI2,WASF2,WASF2,CDKN1B,LOC102724334,NET1,TUBA1B,TUBB3,TUBB4A,TUBB4B,MYL12B,MYL12B,DLC1,MYL9,MYL9,NDC80,VAV3,BAIAP2,BAIAP2,NCOA2,ARPC1A,ARPC1A,CENPA,CENPC,CENPE,MYL12A,MYL12A,CENPF,GNA13,CFL1,NUDC,NCKAP1,NCKAP1,IQGAP2,CFTR,WASF3,WASF3,NOXA1,RALBP1,YWHAQ,KIF2C,CIT,CIT,ZWINT,AKAP13,CHN1,CHN2,PMF1,TUBA3E,TUBA3D,FMNL2,RHPN1,RHPN1,ARHGAP33,ARAP2,ARAP1,TAGAP,H4-16,FGD4,NOXO1,H3C14,IQGAP3,ARHGEF19,H2BU1,DYNLL2,MAPK14,ARHGAP42,WIPF2,WIPF2,KLC3,SPC24,CTNNA1,CTNNB1,SGO2,SGO1,CYBA,CYBB,ARHGAP36,RHOV,DIAPH1,DIAPH2,DLG4,DYNC1H1,DYNC1I1,DYNC1I2,DYNC1LI2,DVL1,DVL2,DVL3,ECT2,A2M,ARHGAP27,CTTN,SKA1,FGD2,FGD1,ARHGEF15,MAPRE1,DAAM1,KDM1A,KDM4C,ARHGAP26,CLASP2,FLNA,CYFIP1,CYFIP1,RHOBTB2,ARHGEF9,MCF2L,NUP160,CLASP1,ARHGEF12,ARHGEF18,SRGAP2,ITGB3BP,RHOQ,ARHGAP45,ARHGAP8,CENPI,ABL1,H2BC1,ARHGAP30,NGEF,AHCTF1,NSL1,ARHGEF26,TIAM2,GDI1,GDI2,CYFIP2,CYFIP2,NOX1,ARHGEF16,SFN,SCAI,GRB2,GRB2,ABR,ARHGAP35,RACGAP1,PKN3,RHOD,H2AC8,H2AC7,H2AX,H2AZ1,H2BC5,H2BC3,H3-3A,H3-3B,NCKAP1L,NCKAP1L,PIK3R4,TAX1BP3,BIRC5,H3C15,TUBB8,TUBB2B,SKA2,NUP43,KLK3,INCENP,INPP5B,AR,ITGB1,CENPS,KIF2A,KIF5A,KIF5B,KLK2,KLC1,RHOA,RHOA,RHOB,RHOB,RHOC,RHOC,ARHGEF37,KTN1,RHOG,ARHGAP1,ARHGAP4,ARHGAP5,ARHGAP6,ARHGDIA,ARHGDIB,ARHGDIG,LIMK1,LIMK1,LIMK2,RHOH,CENPP,MAD2L1,MCF2,MEN1,ARHGEF35,MYH9,MYH9,MYH10,MYH10,MYH11,MYH11,MYL6,MYL6,MYLK,MYLK,MYO9A,MYO9B,PPP1R12A,PPP1R12A,PPP1R12B,PPP1R12B,NCF2,NCF4,NCK1,NCK1,H2AB1,NF2,NUP98,OCRL,OPHN1,PAFAH1B1,NOX3,PAK1,PAK1,PAK2,PAK2,PAK3,PAK3,ARHGEF4,ARHGEF3,DYNC1LI1,GMIP,FAM13B,EVL,NCKIPSD,NCKIPSD,PDPK1,TUBA8,PFN1,PFN1,PFN2,PFN2,PIK3C3,PIK3R2,PIN1,PLK1,RHOF,ROPN1,NDE1,ERCC6L,ARHGEF38,SPDL1,PPP1CB,PPP1CB,PPP1CC,ZWILCH,ARHGAP17,CDCA8,PPP2CA,PPP2CB,ARHGEF10L,CENPQ,PPP2R1A,PPP2R1B,PPP2R5A,PPP2R5B,PPP2R5C,PPP2R5D,RHOT1,PPP2R5E,H4C15,ARHGEF40,NUP133,H2AJ,PRKCA,DEPDC1B,PRKCB,PRKCD,CENPN,ARHGAP15,BRK1,BRK1,PKN1,PKN2,PRKCZ,RCC2,MAPK1,MAPK3,MAPK11,KNL1,GOPC,NUP107,RHOJ,SPC25,PLEKHG5,PTK2,ARHGAP31,SRGAP1,TAOK1,ARHGAP20,PREX1,ARHGAP21,MRTFA,ARHGAP23,RHOU,ARHGAP22,RAC1,RAC1,RAC2,RAC3,RANBP2,RANGAP1,RASGRF2,ACTB,ACTB,ROCK1,ROCK1,BCR,RPS27,RTKN,RTKN,CLIP1,S100A8,S100A9,SEC13,CENPK,LIN7B,ARHGAP9,ARAP3,WIPF3,WIPF3,ITSN1,KLC2,PLEKHG2,CENPH,NCF1,H3C13,SOS1,SOS2,SRC,SRF,BTK,BUB1,BUB1B,TIAM1,ACTG1,ACTG1,TRIO,H2AC19,TUBA4A,TUBA3C,TUBB2A,VAV1,VAV2,WAS,WAS,WIPF1,WIPF1,XPO1,FMNL1,FMNL1,YWHAB,YWHAE,YWHAG,YWHAH,YWHAZ,TUBA1A,MIS12,CENPM,NUP37,CENPO,ARHGAP10,CENPU,MYH14,MYH14,ARHGEF5,TUBAL3,NUP85,DSN1,CALM1,CALM1,CENPT,CALM2,CALM2,ARHGAP39,B9D2,CALM3,CALM3,TUBB1,NDEL1,DIAPH3,SEH1L,KIF18A,H4C9,H2AC14,H2AC6,H2AC4,H2AC18,H2AC20,H2BC8,H2BC13,H2BC15,H2BC14,H2BC7,H2BC6,H2BC9,H2BC10,H2BC4,ARHGAP24,H2BC17,H2BC21,H3C1,H3C4,H3C3,H3C6,H3C11,NUF2,H3C8,H3C12,H3C10,H3C2,H4C1,H4C4,H4C6,H4C12,H4C11,H4C3,H4C8,H4C2,H4C5,H4C13,H4C14,MAD1L1,OBSCN,SYDE2,TUBB6,KIF2B,TUBA1C,ARHGEF39,ARHGAP19,H2BC12,SYDE1,RHPN2,RHPN2,DYNLL1,IQGAP1,ARHGEF7,WASF1,WASF1,H3C7,H2BC11,WASL,WASL,ARHGAP11B,FGD3,RHOT2,KLC4,KALRN,PRC1,STARD13,FMNL3,ARHGEF1,DEPDC7,CENPL,ARHGEF2,ZW10,BUB3,AURKB,TRIP10,ARHGAP18,ARHGAP29,ARHGAP12,H2AZ2,PPP1R14A,ARHGEF6,ROCK2,ROCK2,ARHGEF10,KNTC1,ARHGAP32,STARD8,CKAP5,ARHGAP11A,ARHGEF11,ARHGEF17,RHOBTB1,SRGAP3,CDC20,ARHGAP44,KIF14,ARHGAP25,CDC25C,CDC42,CDC42,CDH1",Signaling by Rho GTPases,517

R-HSA-194441,"SNUPN,PRMT5,NUP50,NUP42,DDX20,CLNS1A,NUP35,NCBP2,NUP205,NUP210,NUP160,NUP188,NUP62,GEMIN5,NUP43,NCBP1,NUP88,NUP98,GEMIN4,PHAX,NUP54,GEMIN8,NDC1,NUP133,NUP107,RANBP2,SEC13,SMN1,SMN2,SNRPB,SNRPD1,SNRPD2,SNRPD3,SNRPE,SNRPF,SNRPG,TPR,NUP37,WDR77,GEMIN7,GEMIN6,NUP85,NUP214,AAAS,SEH1L,RAE1,GEMIN2,NUP155,TGS1,NUP93,NUP58,POM121,NUP153",Metabolism of non-coding RNA,53

R-HSA-194840,"ARHGEF33,FAM13A,NET1,DLC1,VAV3,GNA13,RALBP1,AKAP13,CHN1,CHN2,ARHGAP33,ARAP2,ARAP1,TAGAP,FGD4,ARHGEF19,ARHGAP42,ARHGAP36,RHOV,ECT2,A2M,ARHGAP27,FGD2,FGD1,ARHGEF15,ARHGAP26,RHOBTB2,ARHGEF9,MCF2L,ARHGEF12,ARHGEF18,SRGAP2,RHOQ,ARHGAP45,ARHGAP8,ARHGAP30,NGEF,ARHGEF26,TIAM2,GDI1,GDI2,ARHGEF16,ABR,ARHGAP35,RACGAP1,RHOD,INPP5B,RHOA,RHOB,RHOC,ARHGEF37,RHOG,ARHGAP1,ARHGAP4,ARHGAP5,ARHGAP6,ARHGDIA,ARHGDIB,ARHGDIG,RHOH,MCF2,ARHGEF35,MYO9A,MYO9B,OCRL,OPHN1,ARHGEF4,ARHGEF3,GMIP,FAM13B,PIK3R2,RHOF,ARHGEF38,ARHGAP17,ARHGEF10L,RHOT1,ARHGEF40,DEPDC1B,ARHGAP15,RHOJ,PLEKHG5,ARHGAP31,SRGAP1,ARHGAP20,PREX1,ARHGAP21,ARHGAP23,RHOU,ARHGAP22,RAC1,RAC2,RAC3,RASGRF2,BCR,ARHGAP9,ARAP3,ITSN1,PLEKHG2,SOS1,SOS2,TIAM1,TRIO,VAV1,VAV2,ARHGAP10,ARHGEF5,ARHGAP39,ARHGAP24,OBSCN,SYDE2,ARHGEF39,ARHGAP19,SYDE1,ARHGEF7,ARHGAP11B,FGD3,RHOT2,KALRN,STARD13,ARHGEF1,DEPDC7,ARHGEF2,TRIP10,ARHGAP18,ARHGAP29,ARHGAP12,ARHGEF6,ARHGEF10,ARHGAP32,STARD8,ARHGAP11A,ARHGEF11,ARHGEF17,RHOBTB1,SRGAP3,ARHGAP44,ARHGAP25,CDC42",Rho GTPase cycle,138

R-HSA-195253,"FRAT1,PSME3,PSMD14,PSMB11,AMER1,PSMA8,CSNK1A1,CTBP1,CTBP2,CTNNB1,TLE5,PSME4,FRAT2,GSK3B,HDAC1,HDAC1,APC,LEF1,LEF1,ZRANB1,PPP2CA,PPP2CB,PPP2R1A,PPP2R1B,PPP2R5A,PPP2R5B,PPP2R5C,PPP2R5D,PPP2R5E,PSMA1,PSMA2,PSMA3,PSMA4,PSMA5,PSMA6,PSMA7,PSMB1,PSMB2,PSMB3,PSMB4,PSMB5,PSMB6,PSMB7,PSMB8,PSMB9,PSMB10,PSMC1,PSMC2,PSMC3,PSMC4,PSMC5,PSMC6,PSMD1,PSMD2,PSMD3,PSMD4,PSMD5,PSMD7,PSMD8,PSMD9,PSMD10,PSMD11,PSMD12,PSMD13,PSME1,PSME2,RPS27A,SKP1,TCF7,TCF7,TCF7L2,TCF7L2,TLE1,TLE1,TLE2,TLE2,TLE3,TLE3,TLE4,TLE4,UBA52,UBB,UBC,SEM1,AXIN1,TCF7L1,TCF7L1,CUL1,BTRC,PSMF1,PSMD6,RBX1",Degradation of beta-catenin by the destruction complex,92

R-HSA-195258,"ABI1,ABI1,CENPS-CORT,PMF1-BGLAP,ARPC5,ARPC5,ARPC4,ARPC4,ARPC3,ARPC3,ARPC1B,ARPC1B,ACTR3,ACTR3,ACTR2,ACTR2,ARPC2,ARPC2,ABI2,ABI2,WASF2,WASF2,CDKN1B,LOC102724334,TUBA1B,TUBB3,TUBB4A,TUBB4B,MYL12B,MYL12B,MYL9,MYL9,NDC80,BAIAP2,BAIAP2,NCOA2,ARPC1A,ARPC1A,CENPA,CENPC,CENPE,MYL12A,MYL12A,CENPF,CFL1,NUDC,NCKAP1,NCKAP1,IQGAP2,CFTR,WASF3,WASF3,NOXA1,YWHAQ,KIF2C,CIT,CIT,ZWINT,PMF1,TUBA3E,TUBA3D,FMNL2,RHPN1,RHPN1,H4-16,NOXO1,H3C14,IQGAP3,H2BU1,DYNLL2,MAPK14,WIPF2,WIPF2,KLC3,SPC24,CTNNA1,CTNNB1,SGO2,SGO1,CYBA,CYBB,DIAPH1,DIAPH2,DLG4,DYNC1H1,DYNC1I1,DYNC1I2,DYNC1LI2,DVL1,DVL2,DVL3,CTTN,SKA1,MAPRE1,DAAM1,KDM1A,KDM4C,CLASP2,FLNA,CYFIP1,CYFIP1,NUP160,CLASP1,SRGAP2,ITGB3BP,RHOQ,CENPI,ABL1,H2BC1,AHCTF1,NSL1,CYFIP2,CYFIP2,NOX1,SFN,SCAI,GRB2,GRB2,PKN3,RHOD,H2AC8,H2AC7,H2AX,H2AZ1,H2BC5,H2BC3,H3-3A,H3-3B,NCKAP1L,NCKAP1L,PIK3R4,TAX1BP3,BIRC5,H3C15,TUBB8,TUBB2B,SKA2,NUP43,KLK3,INCENP,AR,ITGB1,CENPS,KIF2A,KIF5A,KIF5B,KLK2,KLC1,RHOA,RHOA,RHOB,RHOB,RHOC,RHOC,KTN1,RHOG,LIMK1,LIMK1,LIMK2,CENPP,MAD2L1,MEN1,MYH9,MYH9,MYH10,MYH10,MYH11,MYH11,MYL6,MYL6,MYLK,MYLK,PPP1R12A,PPP1R12A,PPP1R12B,PPP1R12B,NCF2,NCF4,NCK1,NCK1,H2AB1,NF2,NUP98,PAFAH1B1,NOX3,PAK1,PAK1,PAK2,PAK2,PAK3,PAK3,DYNC1LI1,EVL,NCKIPSD,NCKIPSD,PDPK1,TUBA8,PFN1,PFN1,PFN2,PFN2,PIK3C3,PIN1,PLK1,ROPN1,NDE1,ERCC6L,SPDL1,PPP1CB,PPP1CB,PPP1CC,ZWILCH,CDCA8,PPP2CA,PPP2CB,CENPQ,PPP2R1A,PPP2R1B,PPP2R5A,PPP2R5B,PPP2R5C,PPP2R5D,PPP2R5E,H4C15,NUP133,H2AJ,PRKCA,PRKCB,PRKCD,CENPN,BRK1,BRK1,PKN1,PKN2,PRKCZ,RCC2,MAPK1,MAPK3,MAPK11,KNL1,GOPC,NUP107,SPC25,PTK2,TAOK1,MRTFA,RAC1,RAC1,RAC2,RANBP2,RANGAP1,ACTB,ACTB,ROCK1,ROCK1,RPS27,RTKN,RTKN,CLIP1,S100A8,S100A9,SEC13,CENPK,LIN7B,WIPF3,WIPF3,KLC2,CENPH,NCF1,H3C13,SRC,SRF,BTK,BUB1,BUB1B,ACTG1,ACTG1,H2AC19,TUBA4A,TUBA3C,TUBB2A,WAS,WAS,WIPF1,WIPF1,XPO1,FMNL1,FMNL1,YWHAB,YWHAE,YWHAG,YWHAH,YWHAZ,TUBA1A,MIS12,CENPM,NUP37,CENPO,CENPU,MYH14,MYH14,TUBAL3,NUP85,DSN1,CALM1,CALM1,CENPT,CALM2,CALM2,B9D2,CALM3,CALM3,TUBB1,NDEL1,DIAPH3,SEH1L,KIF18A,H4C9,H2AC14,H2AC6,H2AC4,H2AC18,H2AC20,H2BC8,H2BC13,H2BC15,H2BC14,H2BC7,H2BC6,H2BC9,H2BC10,H2BC4,H2BC17,H2BC21,H3C1,H3C4,H3C3,H3C6,H3C11,NUF2,H3C8,H3C12,H3C10,H3C2,H4C1,H4C4,H4C6,H4C12,H4C11,H4C3,H4C8,H4C2,H4C5,H4C13,H4C14,MAD1L1,TUBB6,KIF2B,TUBA1C,H2BC12,RHPN2,RHPN2,DYNLL1,IQGAP1,WASF1,WASF1,H3C7,H2BC11,WASL,WASL,KLC4,PRC1,FMNL3,CENPL,ZW10,BUB3,AURKB,H2AZ2,PPP1R14A,ROCK2,ROCK2,KNTC1,CKAP5,CDC20,KIF14,CDC25C,CDC42,CDC42,CDH1",RHO GTPase Effectors,389

R-HSA-195399,"VEGFD,FLT1,FLT4,KDR,PGF,VEGFA,VEGFB,VEGFC",VEGF binds to VEGFR leading to receptor dimerization,8

R-HSA-195721,"FRAT1,PSME3,PSMD14,LOC102724334,LOC102724334,TPTEP2-CSNK1E,KAT5,KAT5,GNB5,GNB5,WIF1,AP2M1,AP2S1,CLTA,CLTB,CLTC,H4-16,H4-16,PSMB11,LEO1,H3C14,H2BU1,H2BU1,CREBBP,AMER1,AMER1,PSMA8,PRICKLE1,CSNK1A1,CSNK1A1,CSNK1E,CSNK1G2,CSNK2A1,CSNK2A2,CSNK2B,CTBP1,CTBP2,CTNNB1,CTNNB1,AP2A1,AP2A2,AP2B1,TLE5,DVL1,DVL1,DVL2,DVL2,DVL3,DVL3,AGO3,AGO4,EP300,AKT1,AKT2,DKK1,DKK1,DAAM1,HECW1,TNRC6B,PSME4,PLCB1,FRAT2,SCRIB,FZD2,FZD2,H2BC1,H2BC1,CBY1,PYGO1,AGO1,DKK4,DKK4,DKK2,DKK2,AGO2,TNRC6A,GNAO1,GNAO1,GNAT2,GNAT2,GNB1,GNB1,GNB2,GNB2,GNB3,GNB3,GNG3,GNG3,GNG4,GNG4,GNG5,GNG5,GNG7,GNG7,GNG10,GNG10,GNG11,GNG11,GNGT1,GNGT1,GNGT2,GNGT2,BCL9L,RSPO1,GSK3B,GSK3B,H2AC8,H2AC8,H2AC7,H2AC7,H2AX,H2AX,H2AZ1,H2AZ1,H2BC5,H2BC5,H2BC3,H2BC3,H3-3A,H3-3B,HDAC1,HDAC1,APC,APC,XIAP,H3C15,RSPO2,RSPO4,ITPR1,ITPR2,ITPR3,KRAS,RHOA,LRP6,LRP6,LRP5,LRP5,ARRB2,MEN1,MOV10,CCDC88C,MYC,H2AB1,H2AB1,NFATC1,ROR1,ROR2,ROR2,PARD6A,SOST,SOST,TMED5,LEF1,LEF1,DACT1,WNT16,WNT16,PDE6A,PDE6A,PDE6G,PDE6G,PDE6B,PDE6B,VPS29,NLK,GNG13,GNG13,PFN1,PLCB2,PLCB3,GNG2,GNG2,WNT4,WNT4,ZRANB1,RNF43,PPP2CA,PPP2CA,PPP2CB,PPP2CB,PPP2R1A,PPP2R1A,PPP2R1B,PPP2R1B,PPP2R5A,PPP2R5A,PPP2R5B,PPP2R5B,PPP2R5C,PPP2R5C,PPP2R5D,PPP2R5D,PPP2R5E,PPP2R5E,PPP3CA,PPP3CB,PPP3R1,LGR4,H4C15,H4C15,SOX6,VPS35,H2AJ,H2AJ,PRKCA,PRKCA,PRKCB,PRKCG,PRKG1,PRKG2,GNG12,GNG12,PSMA1,PSMA2,PSMA3,PSMA4,PSMA5,PSMA6,PSMA7,PSMB1,PSMB2,PSMB3,PSMB4,PSMB5,PSMB6,PSMB7,PSMB8,PSMB9,PSMB10,CTNNBIP1,PSMC1,PSMC2,PSMC3,PSMC4,PSMC5,PSMC6,PSMD1,PSMD2,PSMD3,PSMD4,PSMD5,PSMD7,PSMD8,PSMD9,SMURF1,PSMD10,PSMD11,PSMD12,PSMD13,PSME1,PSME2,VANGL2,CHD8,TNRC6C,RAC1,RAC2,RAC3,RBBP5,GNB4,GNB4,KLHL12,LGR6,BCL9,RPS27A,RYK,RYK,SFRP1,SFRP2,SOX17,SMURF2,PORCN,SKP1,H3C13,SMARCA4,SOX2,SOX3,SOX4,SOX9,SRY,MAP3K7,TCF7,TCF7,TCF7L2,TCF7L2,TERT,TLE1,TLE1,TLE2,TLE2,TLE3,TLE3,TLE4,TLE4,H2AC19,H2AC19,UBA52,UBB,UBC,WNT1,WNT1,WNT2,WNT2,WNT3,WNT3,WNT5A,WNT5A,WNT6,WNT6,WNT7A,WNT7A,WNT7B,WNT7B,WNT8A,WNT8A,WNT8B,WNT8B,WNT10B,WNT10B,WNT11,WNT11,WNT2B,WNT2B,WNT9A,WNT9A,WNT9B,WNT9B,XPO1,YWHAZ,FZD5,FZD5,KREMEN2,KREMEN2,CDC73,FZD3,FZD3,SEM1,WLS,CALM1,WNT10A,WNT10A,TNKS2,CALM2,CALM3,KMT2D,WNT5B,WNT5B,CAMK2A,RNF146,H3-4,H3-4,H4C9,H4C9,TRRAP,TRRAP,AXIN1,AXIN1,AXIN2,FZD1,FZD1,FZD4,FZD4,FZD6,FZD6,FZD7,FZD7,FZD8,H2AC14,H2AC14,H2AC6,H2AC6,H2AC4,H2AC4,H2AC18,H2AC18,H2AC20,H2AC20,H2BC8,H2BC8,H2BC13,H2BC13,H2BC15,H2BC15,H2BC14,H2BC14,H2BC7,H2BC7,TCF7L1,TCF7L1,H2BC6,H2BC6,H2BC9,H2BC9,H2BC10,H2BC10,H2BC4,H2BC4,H2BC17,H2BC17,H2BC21,H2BC21,H3C1,H3C4,H3C3,H3C6,H3C11,H3C8,H3C12,H3C10,H3C2,H4C1,H4C1,SOX7,H4C4,H4C4,H4C6,H4C6,H4C12,H4C12,H4C11,H4C11,H4C3,H4C3,H4C8,H4C8,H4C2,H4C2,H4C5,H4C5,H4C13,H4C13,H4C14,H4C14,PIP5K1B,KREMEN1,KREMEN1,ZNRF3,CUL3,CUL1,RSPO3,H2BC12,H2BC12,LGR5,CAV1,RUVBL1,RUVBL1,RUNX3,TNKS,SNX3,BTRC,H3C7,H2BC11,H2BC11,WNT3A,WNT3A,ASH2L,PYGO2,USP8,GNG8,GNG8,H2AZ2,H2AZ2,PSMF1,VPS26A,SOX13,USP34,PSMD6,RBX1",Signaling by WNT,467

R-HSA-196025,"AP2M1,CLTA,CLTB,CLTC,DAB2,DNM1,DNM2,GJA1,ACTB,ACTG1,CLTCL1",Formation of annular gap junctions,11

R-HSA-196071,"CGA,STARD3,FDX2,STARD4,STARD6,CYP11A1,CYP11B1,CYP11B2,CYP17A1,CYP19A1,CYP21A2,FDX1,FDXR,AKR1B1,HSD3B1,HSD3B2,HSD11B1,HSD11B2,HSD17B1,HSD17B3,HSD17B2,LHB,AKR1B15,HSD17B12,HSD17B11,HSD17B14,POMC,SRD5A1,SRD5A2,STAR,TSPO,SRD5A3,STARD3NL,SERPINA6,TSPOAP1",Metabolism of steroid hormones,35

R-HSA-196108,"STARD3,FDX2,STARD4,STARD6,CYP11A1,FDX1,FDXR,AKR1B1,STAR,TSPO,STARD3NL,TSPOAP1",Pregnenolone biosynthesis,12

R-HSA-196299,"FRAT1,AMER1,CSNK1A1,CTNNB1,FRAT2,GSK3B,APC,PPP2CA,PPP2CB,PPP2R1A,PPP2R1B,PPP2R5A,PPP2R5B,PPP2R5C,PPP2R5D,PPP2R5E,AXIN1",Beta-catenin phosphorylation cascade,17

R-HSA-1963640,"NRG3,NRG4,HBEGF,EGF,EGFR,ERBB2,ERBB2,ERBB4,EREG,GRB2,GRB2,NRG1,HRAS,HRAS,KRAS,KRAS,NRAS,NRAS,SOS1,SOS1,BTC,NRG2",GRB2 events in ERBB2 signaling,22

R-HSA-1963642,"NRG3,NRG4,HBEGF,EGF,EGFR,ERBB2,ERBB3,ERBB4,EREG,GAB1,GRB2,NRG1,PIK3CA,PIK3R1,BTC,NRG2",PI3K events in ERBB2 signaling,16

R-HSA-196741,"CTRC,CTRB1,MMAA,MMACHC,CBLIF,MMADHC,MMAB,ABCC1,CTRB2,MTR,MTRR,MMUT,CD320,LMBRD1,PRSS1,PRSS3,ABCD4,TCN1,TCN2,CUBN,AMN","Cobalamin (Cbl, vitamin B12) transport and metabolism",21

R-HSA-196757,"MTHFS,MTHFD2,ALDH1L1,SLC46A1,ALDH1L2,DHFR,DHFR2,FOLR2,FPGS,MTHFD1L,MTHFD2L,MTHFD1,MTHFR,SHMT1,SHMT2,SLC19A1,SLC25A32",Metabolism of folate and pterines,17

R-HSA-196780,"ACACA,HLCS,ACACB,PC,PCCA,PCCB,PDZD11,MCCC1,MCCC2,BTD,SLC5A6",Biotin transport and metabolism,11

R-HSA-196783,"PANK1,PANK4,PPCDC,PANK3,PPCS,PANK2,SLC25A16,COASY",Coenzyme A biosynthesis,8

R-HSA-196791,"CYP2R1,CYP24A1,CYP27B1,GC,LRP2,PIAS4,LGMN,SUMO2,UBE2I,VDR,CUBN",Vitamin D (calciferol) metabolism,11

R-HSA-196807,"NAMPT,NAXE,NADK2,PARP4,CYP8B1,SLC5A8,NMNAT2,QPRT,NMRK2,NMNAT3,NNMT,NT5E,PARP14,PARP16,NMRK1,NADSYN1,RNLS,NAXD,PARP6,PTGIS,PTGS2,NMNAT1,NADK,BST1,PARP8,NUDT12,PARP9,PARP10,NAPRT,SLC22A13,CD38",Nicotinate metabolism,31

R-HSA-196819,"SLC19A2,TPK1,SLC25A19,THTPA,SLC19A3",Vitamin B1 (thiamin) metabolism,5

R-HSA-196836,"GSTO2,CYB5A,CYB5R3,SLC2A1,SLC2A3,GSTO1,SLC23A2,SLC23A1",Vitamin C (ascorbate) metabolism,8

R-HSA-196843,"SLC52A3,ENPP1,ACP5,SLC52A1,RFK,SLC52A2,FLAD1",Vitamin B2 (riboflavin) metabolism,7

R-HSA-196849,"NAMPT,GPHN,SLC19A2,MTHFS,MTHFD2,ALDH1L1,SLC46A1,SLC52A3,CTRC,GSTO2,NAXE,NADK2,PARP4,CTRB1,CYB5A,CYP8B1,ALDH1L2,SLC5A8,MMAA,DHFR,CYB5R3,DHFR2,FASN,NMNAT2,QPRT,FOLR2,FPGS,MTHFD1L,MMACHC,CBLIF,TPK1,NMRK2,MMADHC,MOCS3,ACACA,HLCS,AOX1,ACACB,MMAB,NMNAT3,MOCS1,MOCS2,ABCC1,CTRB2,MTHFD2L,MTHFD1,MTHFR,MTR,MTRR,MMUT,NNMT,NT5E,PC,PCCA,PCCB,PDZD11,CD320,ENPP1,ENPP2,ENPP3,PANK1,ACP5,PARP14,PARP16,NMRK1,MOCOS,SLC52A1,PNPO,NADSYN1,PANK4,RFK,RNLS,NAXD,LMBRD1,PRSS1,PRSS3,MCCC1,PARP6,PTGIS,PTGS2,ABCD4,SLC25A19,PPCDC,AASDHPPT,MCCC2,SHMT1,SHMT2,NMNAT1,SLC2A1,SLC2A3,NADK,SLC19A1,BST1,BTD,TCN1,TCN2,THTPA,SLC52A2,PANK3,PARP8,PPCS,PANK2,CUBN,FLAD1,SLC25A16,COASY,SLC19A3,SLC25A32,AMN,NUDT12,PARP9,PARP10,PDXK,VNN2,VNN1,SLC5A6,NFS1,NAPRT,SLC22A13,GSTO1,CD38,SLC23A2,SLC23A1",Metabolism of water-soluble vitamins and cofactors,123

R-HSA-196854,"GPC6,NAMPT,COQ7,GPHN,SLC19A2,MTHFS,MTHFD2,ALDH1L1,AKR1C4,SLC46A1,SLC52A3,CTRC,GSTO2,CLPS,NAXE,NADK2,PARP4,CTRB1,PLB1,CYB5A,VKORC1L1,CYP8B1,ALDH1L2,SLC5A8,AKR1C1,MMAA,DHFR,DHFR,CYB5R3,DHFR2,AKT1,FASN,GPC2,GPC4,GPC5,NMNAT2,QPRT,FOLR2,FPGS,PDSS1,MTHFD1L,MMACHC,LRP10,GCH1,GCHFR,CBLIF,TPK1,GPC3,NMRK2,MMADHC,MOCS3,GPC1,UBIAD1,LRP12,ACACA,HLCS,AOX1,ACACB,MMAB,HSP90AA1,HSPG2,APOA1,APOA1,APOA2,APOA2,APOB,APOB,GPIHBP1,IDH1,APOC2,APOC2,APOC3,APOC3,APOE,APOE,NMNAT3,AGRN,LDLR,LPL,LRP1,LRP2,MOCS1,MOCS2,ABCC1,CTRB2,MTHFD2L,MTHFD1,MTHFR,MTR,MTRR,MMUT,NNMT,NOS3,NT5E,PC,PCCA,PCCB,COQ6,RDH11,PDZD11,CD320,ENPP1,ENPP2,ENPP3,COQ3,PANK1,BCO1,ACP5,PNLIP,PARP14,RETSAT,PARP16,NMRK1,MOCOS,SLC52A1,PNPO,NADSYN1,PANK4,RFK,RNLS,NAXD,LMBRD1,PRKG2,APOM,PRSS1,PRSS3,MCCC1,PARP6,AKR1B10,COQ9,PDSS2,PTGIS,PTGS2,PTS,ABCD4,RBP1,RBP2,RBP4,SLC25A19,PPCDC,AASDHPPT,SDC1,SDC2,SDC4,MCCC2,SHMT1,SHMT2,NMNAT1,SLC2A1,SLC2A3,NADK,SLC19A1,SPR,BST1,BTD,TCN1,TCN2,TTPA,TTR,LRP8,VKORC1,THTPA,SLC52A2,PANK3,PARP8,PPCS,PANK2,CALM1,CUBN,FLAD1,SLC25A16,COASY,CALM2,SLC19A3,CALM3,SLC25A32,AMN,NUDT12,PARP9,BCO2,COQ5,PARP10,PDXK,AKR1C3,VNN2,VNN1,SLC5A6,NFS1,LRAT,NAPRT,SLC22A13,GSTO1,CD38,SDC3,SLC23A2,SLC23A1",Metabolism of vitamins and cofactors,196

R-HSA-1971475,"GPC6,CSPG5,B4GALT7,B3GALT6,B3GAT2,VCAN,NCAN,CSPG4,DCN,GPC2,GPC4,GPC5,B3GAT3,B3GAT1,GPC3,GPC1,HSPG2,AGRN,BGN,SDC1,BCAN,SDC2,SDC4,XYLT1,XYLT2,SDC3",A tetrasaccharide linker sequence is required for GAG synthesis,26

R-HSA-197264,"NAMPT,NAXE,PARP4,CYP8B1,SLC5A8,NNMT,PARP14,PARP16,RNLS,NAXD,PARP6,PTGIS,PTGS2,PARP8,NUDT12,PARP9,PARP10,NAPRT,SLC22A13",Nicotinamide salvaging,19

R-HSA-1980143,"HDAC6,HDAC5,MAMLD1,MAMLD1,ADAM10,CDK8,DTX2,DTX2,CNTN1,CREBBP,CREBBP,MIB2,JAG1,DTX1,DTX1,EP300,EP300,SNW1,SNW1,DTX4,DTX4,NCSTN,HEY1,HEY2,KAT2A,KAT2A,HEYL,NBEA,DLL1,HDAC1,HDAC2,HIF1A,HES1,RBPJ,RBPJ,JAG2,JAG2,HES5,ARRB1,ARRB2,MYC,NOTCH1,NOTCH1,APH1A,HDAC7,NEURL1B,DLL4,FBXW7,MAML3,MAML3,PSENEN,HDAC8,PSEN1,PSEN2,MIB1,RPS27A,RPS27A,SKP1,ADAM17,TBL1X,TLE1,TLE2,TLE3,TLE4,UBA52,UBA52,UBB,UBB,UBC,UBC,TBL1XR1,HDAC11,APH1B,ITCH,ITCH,HDAC10,MAML2,MAML2,CUL1,NUMB,DLK1,HDAC3,KAT2B,KAT2B,CCNC,NEURL1,DNER,NCOR1,NCOR2,HDAC9,HDAC4,MAML1,MAML1,RBX1",Signaling by NOTCH1,94

R-HSA-1980145,"MAMLD1,MAMLD1,ADAM10,CNTN1,CREB1,MIB2,JAG1,EP300,FCER2,NCSTN,DLL1,GZMB,HES1,RBPJ,RBPJ,JAG2,HES5,MDK,NOTCH2,NOTCH2,APH1A,NEURL1B,DLL4,DLL4,MAML3,MAML3,PSENEN,PSEN1,PSEN2,MIB1,RPS27A,UBA52,UBB,UBC,APH1B,MAML2,MAML2,NEURL1,MAML1,MAML1",Signaling by NOTCH2,40

R-HSA-198203,"IRS1,IRS1,RHOA,NGF,NGF,NTRK1,NTRK1,PIK3CA,PIK3CB,PIK3R1,PIK3R2,IRS2,IRS2",PI3K/AKT activation,13

R-HSA-198323,"AKT3,AKT3,CDKN1A,CDKN1B,CHUK,AKT1,AKT1,AKT2,AKT2,MKRN1,GSK3A,GSK3B,MDM2,BAD,TSC2,CASP9,AKT1S1",AKT phosphorylates targets in the cytosol,17

R-HSA-198693,"AKT3,CREB1,AKT1,AKT2,FOXO1,FOXO3,NR4A1,FOXO4,RPS6KB2",AKT phosphorylates targets in the nucleus,9

R-HSA-198725,"CDK5,TRIB1,CHD4,CREB1,CREB1,ATF2,ATF2,MAPK14,DNM2,DUSP3,DUSP4,DUSP6,DUSP7,EGR1,EGR1,EGR2,EGR2,EGR3,EGR3,EGR4,ELK1,ELK1,EP300,EP300,F3,ARC,ARC,FOS,FOSB,ID1,ID2,ID3,ID4,JUNB,JUND,LYL1,MEF2A,MEF2C,MEF2D,MEF2D,ASCL1,ASCL1,ATF1,ATF1,NAB1,NAB2,NAB2,VRK3,PPP2CA,PPP2CB,PPP2R1A,PPP2R1B,PPP2R5D,MAPK1,MAPK1,MAPK3,MAPK3,MAPK7,MAPK7,MAPK11,REST,RPS6KA1,RPS6KA2,RPS6KA3,RRAD,SGK1,SH3GL3,SRF,SRF,TCF12,TCF12,TPH1,VGF,FOSL1,CDK5R1,CDK5R1,CDK5R2,RPS6KA5,MAPKAPK2",Nuclear Events (kinase and transcription factor activation),79

R-HSA-198745,"NGF,NTRK1,STAT3",Signalling to STAT3,3

R-HSA-198753,"MAPK14,DUSP3,DUSP4,DUSP6,DUSP7,ELK1,MEF2A,MEF2C,VRK3,PPP2CA,PPP2CB,PPP2R1A,PPP2R1B,PPP2R5D,MAPK1,MAPK1,MAPK3,MAPK3,MAPK7,MAPK7,MAPK11,RPS6KA1,RPS6KA2,RPS6KA3,RPS6KA5",ERK/MAPK targets,25

R-HSA-198765,"MAPK7,MAPK7,MAP2K5",Signalling to ERK5,3

R-HSA-198933,"CD300LD,KIR2DS2,MICA,KLRC4-KLRK1,KLRG1,CD96,LOC102725035,CD226,LOC107987462,LILRB1,HCST,CD300C,LILRB5,LILRB4,LILRA1,LILRA3,LILRA2,CD160,CD300A,SIGLEC11,SLAMF6,SH2D1B,JAML,CD300LB,OSCAR,COL1A1,COL1A2,COL2A1,COL3A1,COL17A1,CD200R1,RAET1E,CD300LF,CD300LG,CXADR,PIANP,FCGR1A,FCGR2B,FCGR3A,KLRK1,LILRA4,NCR3,SIGLEC7,SIGLEC9,SIGLEC8,TREML4,CLEC2D,PILRB,PILRA,HLA-A,HLA-B,HLA-C,HLA-E,HLA-F,HLA-G,ICAM1,ICAM2,ICAM3,ICAM4,CLEC4G,TREML1,CD300E,LILRA5,ITGA4,ITGAL,ITGB1,ITGB2,ITGB7,NCR3LG1,KIR2DL1,KIR2DL2,KIR2DL3,KIR2DL4,KIR2DS1,KIR3DL1,KIR3DL2,KLRB1,KLRC1,KLRD1,LAIR1,LAIR2,SH2D1A,CD99,MICB,CD200,KLRF1,TREM2,TREM1,CRTAM,NPDC1,B2M,SLAMF7,PVR,NECTIN2,SELL,SFTPD,SIGLEC1,ICAM5,C3,TYROBP,VCAM1,ULBP3,TREML2,ULBP1,COLEC12,MADCAM1,IFITM1,SIGLEC5,SIGLEC10,SIGLEC12,CD1A,CD1B,CD1C,CD1D,CD3D,CD3E,CD3G,CD247,CD8A,CD8B,CD19,CD22,NCR2,NCR1,CD33,SIGLEC6,CD34,CD40,CD40LG,CD81,CLEC2B,CDH1",Immunoregulatory interactions between a Lymphoid and a non-Lymphoid cell,132

R-HSA-1989781,"MED6,MED16,NR1H3,CDK8,CARM1,CARM1,NCOA2,NCOA2,PPARGC1A,GLIPR1,MED8,APOA5,PLIN2,PPARGC1B,CPT1A,CPT2,CREBBP,CREBBP,CYP1A1,CYP4A11,CYP7A1,AGT,ABCA1,AHR,EP300,ESRRA,ALAS1,FABP1,ACSL1,MED19,FDFT1,FHL2,NCOA6,NCOA6,CDK19,RGL1,MED13L,TIAM2,ANKRD1,TNFRSF21,GPS2,MED4,GRHL1,HMGCR,HMGCS1,HMGCS2,APOA1,APOA2,ACADM,SLC27A1,FADS1,MED11,ARNT,ARNTL,ME1,MTF1,NFYA,NFYB,NFYC,NPAS2,NPAS2,NRF1,G0S2,ACOX1,MED31,ANGPTL4,MED15,ABCB4,UGT1A9,PPARA,PPARA,PPARG,MED1,MED1,MED18,MED9,MED29,AHRR,TRIB3,RORA,RXRA,RXRA,RXRB,SMARCD3,SMARCD3,SP1,SREBF1,SREBF2,SULT2A1,MED22,TBL1X,TBL1X,TXNRD1,NR1H2,TBL1XR1,TBL1XR1,CHD9,CHD9,MED28,MED25,NCOA3,MED10,FAM120B,HELZ2,HELZ2,NCOA1,NCOA1,PEX11A,HDAC3,CCNC,MED30,MED14,MED21,MED23,MED17,MED26,MED27,MED7,MED20,CD36,NR1D1,CLOCK,NCOR1,NCOR2,TGS1,TGS1,MED24,ARNT2,THRAP3,MED12,MED13,NR1H4",PPARA activates gene expression,132

R-HSA-199220,"FASN,PDZD11,ENPP1,ENPP2,ENPP3,PANK1,PANK4,PPCDC,AASDHPPT,PANK3,PPCS,PANK2,SLC25A16,COASY,VNN2,VNN1,SLC5A6",Vitamin B5 (pantothenate) metabolism,17

R-HSA-199418,"AKT3,NRG3,FRS2,THEM4,PIK3AP1,NRG4,KLB,HBEGF,EGF,EGFR,ERBB2,ERBB3,ERBB4,EREG,AKT1,AKT2,ESR1,ESR2,FGF1,FGF2,FGF3,FGF4,FGF5,FGF6,FGF7,FGF8,FGF9,FGF10,FGFR1,FGFR3,FGFR2,FGFR4,PHLPP2,PHLPP1,PIP5K1C,FYN,GAB1,EPGN,FGF20,FGF22,GRB2,ICOS,HGF,NRG1,IL1RAP,INS,INSR,IRAK1,IRS1,AREG,KIT,RHOG,LCK,MET,KITLG,MYD88,TRAT1,IRAK4,PDGFA,PDGFB,PDGFRA,PDGFRB,PIK3CA,PIK3CB,PIK3CD,PIK3R1,PIK3R2,PIP4K2A,PPP2CA,PPP2CB,PPP2R1A,PPP2R1B,PPP2R5A,PPP2R5B,PPP2R5C,PPP2R5D,PPP2R5E,MAPK1,MAPK3,PTEN,TRIB3,PTPN11,RAC1,RAC2,SRC,STRN,BTC,TGFA,TRAF6,VAV1,PIP4K2C,FGF23,PIP5K1A,PIP5K1B,PIP4K2B,PIK3R3,IRS2,FGF18,FGF17,FGF16,IER3,IL33,IL1RL1,CD19,KL,CD28,CD80,CD86,NRG2,FGF19",Negative regulation of the PI3K/AKT network,110

R-HSA-199920,"CREB1,ATF1,RPS6KA1,RPS6KA2,RPS6KA3,RPS6KA5,MAPKAPK2",CREB phosphorylation,7

R-HSA-199977,"TMED7-TICAM2,PREB,ACTR1A,CNIH1,BET1,TFG,TUBA1B,TUBB3,TUBB4A,TUBB4B,SEC24B,COG5,SEC23A,DCTN2,YKT6,DCTN6,CTSC,SEC24A,KDELR1,TMED2,LMAN2,TMED10,KDELR2,KDELR3,TMEM115,SEC23IP,DCTN3,TUBA3E,COPE,TUBA3D,TRAPPC6B,TRAPPC5,TBC1D20,COL7A1,COPA,COPB1,DYNLL2,CSNK1D,CNIH3,CTSZ,CD55,DCTN1,DYNC1H1,DYNC1I1,DYNC1I2,DYNC1LI2,F5,F8,COG2,COPZ1,COPG1,PPP6R1,SEC31A,ANKRD28,SCFD1,TMED3,FOLR1,CNIH2,COG4,ARFGAP3,COPG2,SEC22A,TRAPPC3,GOLGA2,GOLGB1,ANK1,ANK2,ANK3,GRIA1,TUBB8,TUBB2B,INS,ARCN1,AREG,ARF1,MIA3,ARF3,ARF4,ARF5,LMAN1,MIA2,NSF,TMED7,SAR1B,DYNC1LI1,DCTN4,COPZ2,BET1L,SPTBN5,TRAPPC4,TRAPPC2L,TUBA8,SERPINA1,TMED9,STX17,PPP6R3,PPP6C,ARFGAP1,ACTR10,COG6,SPTBN4,TRAPPC1,RAB1A,NAPB,SEC13,TRAPPC2,GORASP1,SPTA1,SPTAN1,SPTB,SPTBN1,SPTBN2,STX5,TGFA,TRAPPC10,TUBA4A,TUBA3C,TUBB2A,TUBA1A,TRAPPC6A,LMAN1L,TUBAL3,TUBB1,LMAN2L,RAB1B,CAPZA1,CAPZA2,CAPZB,COG3,TRAPPC9,COG8,ARFGAP2,DCTN5,TUBB6,TUBA1C,USO1,DYNLL1,GBF1,NAPG,NAPA,SEC16B,MCFD2,SEC22C,COG7,COPB2,CAPZA3,COG1,GOSR1,SEC22B,GOSR2,SEC24C,CD59,SEC24D,SEC16A",ER to Golgi Anterograde Transport,154

R-HSA-199991,"AKT3,TMED7-TICAM2,GJC1,RNF103-CHMP3,AP1M2,ARPC5,ARPC4,ARPC3,ACTR3,ACTR2,ARPC2,KIF20A,PREB,ACTR1A,OPTN,ARFRP1,ARFRP1,CNIH1,PLIN3,STX6,RABEPK,STAM2,DENND4A,BET1,TFG,TUBA1B,TUBA1B,TUBB3,TUBB3,TUBB4A,TUBB4A,TUBB4B,TUBB4B,SEC24B,COG5,SEC23A,DCTN2,ARPC1A,TGOLN2,CENPE,EXOC5,YKT6,DCTN6,TBC1D7-LOC100130357,AP4B1,KIF1C,CTSC,CFTR,SEC24A,GJB6,RAB10,RAB10,MAN1A2,COPS8,KDELR1,TMED2,LMAN2,YWHAQ,TMED10,COPS6,RAB32,COPS5,KIF2C,KDELR2,KDELR3,RAB35,RAB31,STON1,TMEM115,KIF3A,KIF3A,AP4S1,SEC23IP,CHM,CHML,PACSIN2,SNX18,DCTN3,SNF8,TUBA3E,TUBA3E,CHRM2,VPS45,COPE,KIF12,EXOC3,GABARAP,GABARAPL2,TUBA3D,TUBA3D,FCHO2,RAB39B,RAB3IP,AP2M1,AP1S1,AP2S1,AP3S1,CLTA,CLTB,CLTC,TRAPPC6B,NAA30,KIF19,TBC1D16,GJD3,TRAPPC5,RINL,GJB4,SYT2,TBC1D20,CHMP4B,COL7A1,AP1S3,COPA,COPB1,CLVS2,CPD,VPS37A,DYNLL2,VTI1A,SYT9,CSNK1D,KIF18B,KLC3,CNIH3,EXOC8,CTSZ,CUX1,SH3D19,ADRB2,VPS37D,GRK2,GRK3,CLVS1,AP2A1,DAB2,CD55,DENND5B,AP2A2,AP1B1,AP2B1,DENND2C,DENND1B,DCTN1,AP1G1,DNM1,DNASE2,DYNC1H1,DYNC1I1,DYNC1I2,DYNC1LI2,DNM2,HBEGF,AGTR1,DVL2,TOR1A,EGF,EGFR,RAB12,DENND6A,CTTN,EPS15,EREG,AKT1,AKT1,AKT2,AKT2,F5,F8,GJD4,KIF6,CCZ1B,COG2,COPZ1,COPG1,RHOBTB3,AAK1,PPP6R1,SEC31A,TRAPPC8,MON1B,DENND3,EPN2,KIFAP3,KIFAP3,RAB3GAP1,RAB18,RAB21,KIF21B,FNBP1,GGA2,KIF1B,FCHO1,GGA3,SYT11,TBC1D1,ANKRD28,SCFD1,DENND5A,EXOC7,BICD2,KIF13B,PIP5K1C,TMED3,AP4E1,RHOQ,FOLR1,SNAPIN,RABGAP1,ARFIP2,RAB38,KIF4A,FTH1,ALPP,FTL,CNIH2,EPGN,RAB3GAP2,GAK,COG4,GALNT1,GALNT2,ALS2CL,NECAP1,CHMP2B,TBC1D10B,DNM3,GGA1,LDLRAP1,GAPVD1,KIF26A,BLOC1S6,ARFGAP3,BLOC1S1,GDI1,GDI2,COPG2,GJA1,GJA1,SEC22A,GJA3,GJA4,GJA5,GJA8,GJB1,GJB1,GJB2,GJB2,GJB3,GJB5,TRAPPC3,CYTH4,SNX5,DENND2A,VPS4A,CHMP2A,AMPH,RAB30,GOLIM4,RABGEF1,TOR1B,BIN1,GNS,GOLGA1,GOLGA2,GOLGA4,GOLGB1,SFN,KIF4B,ANK1,YIPF6,STON1-GTF2A1L,ANK2,GPS1,ANK3,GRB2,GRIA1,CHMP4A,RACGAP1,PACSIN3,EPN1,UBQLN2,UBQLN1,SLC2A8,PACSIN1,SH3KBP1,HIP1,HPS1,AGFG1,HSPA8,APOB,RAB7B,RAB43,RAB41,TUBB8,TUBB8,TUBB2B,TUBB2B,IGF2R,APP,IL7R,INS,ARCN1,AREG,TBC1D10C,ARF1,MIA3,GJB7,ARF3,ARF4,KIF2A,KIF3C,KIF5A,KIF5B,ARF5,ARF6,KLC1,KIF11,KIFC1,KIF25,KIF22,BLOC1S3,LDLR,LMAN1,ARL1,LNPEP,LRP2,M6PR,ARRB1,ARRB2,MAN1A1,MAN2A2,MAN2A1,TBC1D3C,DENND6B,RAB8A,RAB8A,MIA2,MYH9,MYO1C,MYO5A,MYO6,NEDD8,NSF,TBC1D25,OCRL,PAFAH1B1,PAFAH1B2,PAFAH1B3,ITSN2,COPS7A,TMED7,VPS36,TRAPPC12,SAR1B,COPS4,DYNC1LI1,VPS28,DCTN4,RAB9B,COPZ2,TBC1D7,UBAP1,BET1L,SPTBN5,TRAPPC4,PRKAG2,SNX9,CHMP5,VTA1,VPS54,RAB14,RAB14,RAB6B,NBAS,CCZ1,CHMP3,TRAPPC2L,RAB8B,TUBA8,TUBA8,SERPINA1,PIK3C2A,PLA2G4A,STX18,PRKAG3,RIN2,EXOC6,TBC1D13,KIF1A,TMED9,RAB39A,FNBP1L,TBC1D8B,STX17,VPS37C,KIF26B,AVP,VPS53,PPP6R3,BLOC1S4,TBC1D2,PPP6C,AVPR2,KIF27,KIF21A,KIF16B,PRKAA2,PRKAB1,PRKAB2,DENND4C,NECAP2,PRKAG1,ARFGAP1,EXOC1,EXOC2,USE1,ACTR10,AGPAT3,KIF15,MAN1C1,RALGAPB,GJC2,RALGAPA2,GJD2,TBC1D24,COG6,TBC1D14,RIC1,ALS2,DENND1A,SPTBN4,TRAPPC1,EPS15L1,RAB1A,RAB3A,RAB3IL1,RAB4A,RAB4A,RAB5A,RAB5B,RAB6A,RAB13,RAB13,RAB27A,RAB27B,RAB5C,RAC1,RALA,ACTB,ACTB,EXOC4,NAA35,RINT1,SCOC,TRAPPC11,RPS27A,SORT1,VPS52,SBF1,BICD1,NAPB,SEC13,TRAPPC2,KIF9,ITSN1,SH3GL1,SH3GL2,SH3GL3,GORASP1,COPS7B,ACBD3,TBC1D15,KLC2,SLC2A4,SLC18A3,BNIP1,SNX2,SPTA1,SPTAN1,SPTB,SPTBN1,SPTBN2,SRC,DENND2B,STX4,STX4,STX5,STXBP3,SURF4,VAMP2,VAMP2,VAMP7,BTC,SYT1,TACR1,TF,TFRC,TGFA,TJP1,TJP1,ACTG1,ACTG1,TRAPPC10,TMF1,TPD52,TPD52L1,TSC1,TSC2,TSG101,TUBA4A,TUBA4A,TUBA3C,TUBA3C,TUBB2A,TUBB2A,TBC1D3,UBA52,UBB,UBC,VPS51,WNT5A,YWHAB,YWHAE,YWHAE,YWHAG,YWHAH,YWHAZ,TUBA1A,TUBA1A,RAB7A,ASPSCR1,TRAPPC6A,GCC1,CHMP6,VPS37B,TBC1D17,LMAN1L,TUBAL3,TUBAL3,RIN3,DENND1C,DENND2D,TRAPPC13,CALM1,STAM,CALM2,CALM3,GJA9,TUBB1,TUBB1,LMAN2L,TXNDC5,MAP1LC3B,SBF2,RAB1B,KIF18A,CLTCL1,CAPZA1,CAPZA2,PICALM,CAPZB,FZD4,RAB33B,COG3,TRAPPC9,TBC1D10A,PLA2G6,DTNBP1,ANKRD27,ULK1,SGIP1,VPS25,MON1A,NAA38,COG8,ARFGAP2,DCTN5,TUBB6,TUBB6,KIF2B,GJA10,TUBA1C,TUBA1C,SYTL1,REPS1,COPS3,STON2,AP3B1,MADD,USO1,DYNLL1,CBL,VAMP8,VAMP4,STX16,STX10,GBF1,RAB11A,RAB11A,SNAP23,SNAP23,NAPG,NAPA,SYNJ1,SYNJ2,AP1S2,AP1G2,AP1M1,WASL,HPS4,MVB12B,SEC16B,KLC4,SYT8,SYS1,HIP1R,MCFD2,KIFC2,SEC22C,RABEP1,HGS,CD3D,CD3G,CHMP7,AP4M1,ZW10,REPS2,COG7,CD4,RAB11B,CHMP4C,CYTH3,CYTH2,CYTH1,COPB2,COPS2,TRIP11,TRIP10,MVB12A,VAMP3,SNAP29,RAB33A,CAPZA3,RAB9A,KIF3B,KIF3B,COG1,KIF23,SCARB2,VPS4B,GOSR1,SEC22B,GOSR2,KIF20B,RAB36,RIN1,SEC24C,GCC2,CD59,CLINT1,PUM1,USP6NL,RGP1,DNAJC6,C2CD5,KIAA0319,SEC24D,TBC1D4,SNAP91,DENND4B,SEC16A,GOLGA5",Membrane Trafficking,673

R-HSA-199992,"AP1M2,TGOLN2,AP4B1,AP4S1,AP1S1,AP3S1,CLTA,CLTB,CLTC,AP1S3,CLVS2,CPD,CTSZ,SH3D19,CLVS1,AP1B1,AP1G1,DNASE2,DNM2,AP4E1,SNAPIN,FTH1,FTL,GAK,NECAP1,BLOC1S6,BLOC1S1,SNX5,CHMP2A,GNS,GOLGB1,YIPF6,HSPA8,IGF2R,APP,ARF1,BLOC1S3,M6PR,ARRB1,OCRL,SNX9,PIK3C2A,TBC1D8B,BLOC1S4,RAB5C,SORT1,SH3GL2,ACBD3,SNX2,STX4,VAMP2,VAMP7,TFRC,TPD52,TPD52L1,TXNDC5,PICALM,DTNBP1,AP3B1,VAMP8,GBF1,SNAP23,NAPA,AP1S2,AP1G2,AP1M1,HIP1R,HGS,AP4M1,CLINT1,PUM1,DNAJC6",trans-Golgi Network Vesicle Budding,72

R-HSA-200425,"CPT1A,CPT1B,CPT2,ACACA,ACACB,PRKAG2,PPARD,PRKAA2,PRKAB2,MID1IP1,RXRA,SLC22A5,THRSP,SLC25A20",Carnitine metabolism,14

R-HSA-201451,"FSTL1,GDF2,AMH,AMHR2,BMP10,SMAD1,SMAD4,SMAD5,SMAD6,SMAD7,SMAD9,SMURF1,GREM2,SMURF2,SKI,BMP2,BMPR1A,BMPR1B,BMPR2,UBE2D1,UBE2D3,CHRDL1,ACVR2A,NOG,ACVR2B,CER1,ACVRL1,ZFYVE16",Signaling by BMP,28

R-HSA-201681,"FRAT1,PSME3,PSMD14,LOC102724334,LOC102724334,TPTEP2-CSNK1E,KAT5,KAT5,WIF1,H4-16,H4-16,PSMB11,LEO1,H3C14,H2BU1,H2BU1,CREBBP,AMER1,AMER1,PSMA8,CSNK1A1,CSNK1A1,CSNK1E,CSNK1G2,CSNK2A1,CSNK2A2,CSNK2B,CTBP1,CTNNB1,CTNNB1,DVL1,DVL1,DVL2,DVL2,DVL3,DVL3,EP300,AKT1,AKT2,DKK1,DKK1,HECW1,PSME4,FRAT2,FZD2,FZD2,H2BC1,H2BC1,CBY1,PYGO1,DKK4,DKK4,DKK2,DKK2,BCL9L,RSPO1,GSK3B,GSK3B,H2AC8,H2AC8,H2AC7,H2AC7,H2AX,H2AX,H2AZ1,H2AZ1,H2BC5,H2BC5,H2BC3,H2BC3,H3-3A,H3-3B,HDAC1,APC,APC,XIAP,H3C15,RSPO2,RSPO4,LRP6,LRP6,LRP5,LRP5,MEN1,CCDC88C,MYC,H2AB1,H2AB1,SOST,SOST,LEF1,LEF1,DACT1,WNT4,RNF43,PPP2CA,PPP2CA,PPP2CB,PPP2CB,PPP2R1A,PPP2R1A,PPP2R1B,PPP2R1B,PPP2R5A,PPP2R5A,PPP2R5B,PPP2R5B,PPP2R5C,PPP2R5C,PPP2R5D,PPP2R5D,PPP2R5E,PPP2R5E,LGR4,H4C15,H4C15,SOX6,H2AJ,H2AJ,PSMA1,PSMA2,PSMA3,PSMA4,PSMA5,PSMA6,PSMA7,PSMB1,PSMB2,PSMB3,PSMB4,PSMB5,PSMB6,PSMB7,PSMB8,PSMB9,PSMB10,CTNNBIP1,PSMC1,PSMC2,PSMC3,PSMC4,PSMC5,PSMC6,PSMD1,PSMD2,PSMD3,PSMD4,PSMD5,PSMD7,PSMD8,PSMD9,PSMD10,PSMD11,PSMD12,PSMD13,PSME1,PSME2,CHD8,RBBP5,KLHL12,LGR6,BCL9,RPS27A,RYK,SFRP1,SFRP2,SOX17,SMURF2,H3C13,SMARCA4,SOX2,SOX3,SOX4,SOX9,SRY,TCF7,TCF7,TCF7L2,TCF7L2,TERT,TLE1,TLE2,TLE3,TLE4,H2AC19,H2AC19,UBA52,UBB,UBC,WNT1,WNT1,WNT3,WNT5A,WNT5A,WNT8A,WNT8A,WNT8B,WNT8B,WNT9A,XPO1,YWHAZ,FZD5,KREMEN2,KREMEN2,CDC73,SEM1,TNKS2,KMT2D,RNF146,H3-4,H3-4,H4C9,H4C9,TRRAP,TRRAP,AXIN1,AXIN1,AXIN2,FZD1,FZD1,FZD4,FZD6,FZD8,H2AC14,H2AC14,H2AC6,H2AC6,H2AC4,H2AC4,H2AC18,H2AC18,H2AC20,H2AC20,H2BC8,H2BC8,H2BC13,H2BC13,H2BC15,H2BC15,H2BC14,H2BC14,H2BC7,H2BC7,TCF7L1,TCF7L1,H2BC6,H2BC6,H2BC9,H2BC9,H2BC10,H2BC10,H2BC4,H2BC4,H2BC17,H2BC17,H2BC21,H2BC21,H3C1,H3C4,H3C3,H3C6,H3C11,H3C8,H3C12,H3C10,H3C2,H4C1,H4C1,SOX7,H4C4,H4C4,H4C6,H4C6,H4C12,H4C12,H4C11,H4C11,H4C3,H4C3,H4C8,H4C8,H4C2,H4C2,H4C5,H4C5,H4C13,H4C13,H4C14,H4C14,PIP5K1B,KREMEN1,KREMEN1,ZNRF3,CUL3,RSPO3,H2BC12,H2BC12,LGR5,CAV1,RUVBL1,RUVBL1,RUNX3,TNKS,BTRC,H3C7,H2BC11,H2BC11,WNT3A,WNT3A,ASH2L,PYGO2,USP8,H2AZ2,H2AZ2,PSMF1,SOX13,USP34,PSMD6,RBX1",TCF dependent signaling in response to WNT,319

R-HSA-201688,"TPTEP2-CSNK1E,CSNK1E,CSNK2A1,CSNK2A2,CSNK2B,DVL1,DVL2,DVL3,PIP5K1B",WNT mediated activation of DVL,9

R-HSA-201722,"LOC102724334,LOC102724334,KAT5,KAT5,H4-16,H4-16,LEO1,H3C14,H2BU1,H2BU1,CREBBP,CTNNB1,CTNNB1,EP300,H2BC1,H2BC1,PYGO1,BCL9L,H2AC8,H2AC8,H2AC7,H2AC7,H2AX,H2AX,H2AZ1,H2AZ1,H2BC5,H2BC5,H2BC3,H2BC3,H3-3A,H3-3B,HDAC1,H3C15,MEN1,MYC,H2AB1,H2AB1,LEF1,LEF1,H4C15,H4C15,H2AJ,H2AJ,RBBP5,BCL9,H3C13,SMARCA4,TCF7,TCF7,TCF7L2,TCF7L2,TERT,TLE1,TLE2,TLE3,TLE4,H2AC19,H2AC19,CDC73,KMT2D,H3-4,H3-4,H4C9,H4C9,TRRAP,TRRAP,AXIN2,H2AC14,H2AC14,H2AC6,H2AC6,H2AC4,H2AC4,H2AC18,H2AC18,H2AC20,H2AC20,H2BC8,H2BC8,H2BC13,H2BC13,H2BC15,H2BC15,H2BC14,H2BC14,H2BC7,H2BC7,TCF7L1,TCF7L1,H2BC6,H2BC6,H2BC9,H2BC9,H2BC10,H2BC10,H2BC4,H2BC4,H2BC17,H2BC17,H2BC21,H2BC21,H3C1,H3C4,H3C3,H3C6,H3C11,H3C8,H3C12,H3C10,H3C2,H4C1,H4C1,H4C4,H4C4,H4C6,H4C6,H4C12,H4C12,H4C11,H4C11,H4C3,H4C3,H4C8,H4C8,H4C2,H4C2,H4C5,H4C5,H4C13,H4C13,H4C14,H4C14,H2BC12,H2BC12,RUVBL1,RUVBL1,RUNX3,H3C7,H2BC11,H2BC11,ASH2L,PYGO2,H2AZ2,H2AZ2",Formation of the beta-catenin:TCF transactivating complex,145

R-HSA-202040,"GNB5,GNAI1,GNAI2,GNAI3,GNAO1,GNAT1,GNAT2,GNAZ,GNB1,GNB2,GNB3,GNG3,GNG4,GNG5,GNG7,GNG10,GNG11,GNGT1,GNGT2,GNAT3,OPRM1,PDYN,GNG13,GNG2,POMC,GNG12,GNB4,GNG8",G-protein activation,28

R-HSA-202131,"LYPLA1,CYGB,NOSTRIN,DNM2,AKT1,DDAH2,DDAH1,HSP90AA1,ZDHHC21,NOS3,NOSIP,SPR,CALM1,CALM2,CALM3,CAV1,WASL",Metabolism of nitric oxide: NOS3 activation and regulation,17

R-HSA-2022090,"COL1A1,COL1A2,COL2A1,COL3A1,COL4A1,COL4A2,COL4A3,COL4A4,COL4A5,COL4A6,COL5A1,COL5A2,COL6A1,COL6A2,COL6A3,COL7A1,COL8A1,COL8A2,COL9A1,COL9A2,COL9A3,COL10A1,COL11A1,COL11A2,COL12A1,COL15A1,COL17A1,COL6A6,CTSB,CTSL,CTSV,CTSS,COL24A1,COL6A5,ITGA6,ITGB4,LAMA3,LAMB3,LAMC2,LOX,LOXL1,LOXL2,MMP3,MMP7,MMP9,MMP13,COL5A3,PCOLCE,PLEC,BMP1,DST,TLL1,TLL2,COL14A1,PXDN,COL18A1,LOXL4,LOXL3,COL27A1,MMP20,CD151",Assembly of collagen fibrils and other multimeric structures,61

R-HSA-2022377,"ATP6AP2,CES1,CMA1,CPA3,CPB1,CPB2,CTSD,CTSG,CTSZ,ACE,AGT,AGT,ENPEP,ANPEP,GZMH,MME,ACE2,REN,AOPEP",Metabolism of Angiotensinogen to Angiotensins,19

R-HSA-2022854,"B3GNT3,ST3GAL6,B3GNT2,B4GAT1,SLC35D2,KERA,ACAN,FMOD,CHST5,B4GALT1,LUM,CHST6,OMD,OGN,PRELP,ST3GAL1,ST3GAL2,ST3GAL4,ST3GAL3,B3GNT4,CHST1,B4GALT4,B4GALT3,B4GALT2,B3GNT7,B4GALT6,B4GALT5,CHST2",Keratan sulfate biosynthesis,28

R-HSA-2022857,"KERA,ACAN,FMOD,GALNS,GLB1,GNS,HEXA,HEXB,LUM,OMD,OGN,PRELP,GLB1L",Keratan sulfate degradation,13

R-HSA-2022870,"CSPG5,VCAN,NCAN,CSPG4,DCN,CHST13,CHSY1,CHSY3,CHST11,CHST15,CHPF2,CSGALNACT2,CHST12,CSGALNACT1,CHST7,BGN,BCAN,CHPF,CHST9,CHST3",Chondroitin sulfate biosynthesis,20

R-HSA-2022923,"UST,CSPG5,CHST14,VCAN,NCAN,CSPG4,DCN,DSE,BGN,BCAN,DSEL",Dermatan sulfate biosynthesis,11

R-HSA-2022928,"GPC6,GPC6,SLC35D2,EXT1,EXT2,GPC2,GPC2,HS3ST5,GPC4,GPC4,GPC5,GPC5,GLCE,HS6ST3,GPC3,GPC3,GPC1,GPC1,HSPG2,HSPG2,NDST1,AGRN,AGRN,SDC1,SDC1,SDC2,SDC2,SDC4,SDC4,NDST4,HS3ST6,NDST2,HS6ST2,NDST3,HS6ST1,HS2ST1,SDC3,SDC3,HS3ST4,HS3ST3B1,HS3ST3A1,HS3ST2,HS3ST1",HS-GAG biosynthesis,43

R-HSA-2023837,FGFR2,Signaling by FGFR2 amplification mutants,1

R-HSA-202403,"PSME3,PSMD14,MALT1,CHUK,PSMB11,PSMA8,CSK,TAB2,PSME4,FBXW11,FYB1,FYB1,PTPN22,LAT,HLA-DPA1,HLA-DPB1,HLA-DQA1,HLA-DQA2,HLA-DQB1,HLA-DQB2,HLA-DRA,HLA-DRB1,HLA-DRB3,HLA-DRB4,HLA-DRB5,IKBKB,INPP5D,ITK,TMEM189-UBE2V1,LCK,LCP2,LCP2,NCK1,NFKB1,NFKBIA,PAK1,PAK2,PAK3,TRAT1,EVL,PDPK1,PIK3CA,PIK3CB,PIK3R1,PIK3R2,PLCG1,PLCG2,ENAH,PAG1,PRKCQ,PSMA1,PSMA2,PSMA3,PSMA4,PSMA5,PSMA6,PSMA7,PSMB1,PSMB2,PSMB3,PSMB4,PSMB5,PSMB6,PSMB7,PSMB8,PSMB9,PSMB10,PSMC1,PSMC2,PSMC3,PSMC4,PSMC5,PSMC6,PSMD1,PSMD2,PSMD3,PSMD4,PSMD5,PSMD7,PSMD8,PSMD9,PSMD10,PSMD11,PSMD12,PSMD13,PSME1,PSME2,PTEN,PTPRC,PTPRJ,RELA,RPS27A,SKP1,MAP3K7,TRAF6,UBA52,UBB,UBC,UBE2D1,UBE2D2,UBE2N,UBE2V1,VASP,WAS,ZAP70,SEM1,CARD11,CUL1,IKBKG,RIPK2,BCL10,BTRC,CD3D,CD3E,CD3G,CD247,CD4,CD101,GRAP2,PSMF1,PSMD6,CDC34",TCR signaling,122

R-HSA-2024096,"GPC6,HPSE,HGSNAT,GPC2,GPC4,GPC5,GPC3,GLB1,GPC1,GUSB,HSPG2,IDS,IDUA,AGRN,NAGLU,HPSE2,SDC1,SDC2,SDC4,SGSH,GLB1L,SDC3",HS-GAG degradation,22

R-HSA-2024101,"CSPG5,VCAN,NCAN,CSPG4,DCN,HEXA,HEXB,HYAL1,IDS,IDUA,ARSB,BGN,BCAN,HYAL3",CS/DS degradation,14

R-HSA-202424,"PSME3,PSMD14,MALT1,CHUK,PSMB11,PSMA8,TAB2,PSME4,FBXW11,HLA-DPA1,HLA-DPB1,HLA-DQA1,HLA-DQA2,HLA-DQB1,HLA-DQB2,HLA-DRA,HLA-DRB1,HLA-DRB3,HLA-DRB4,HLA-DRB5,IKBKB,INPP5D,TMEM189-UBE2V1,LCK,NFKB1,NFKBIA,TRAT1,PDPK1,PIK3CA,PIK3CB,PIK3R1,PIK3R2,PRKCQ,PSMA1,PSMA2,PSMA3,PSMA4,PSMA5,PSMA6,PSMA7,PSMB1,PSMB2,PSMB3,PSMB4,PSMB5,PSMB6,PSMB7,PSMB8,PSMB9,PSMB10,PSMC1,PSMC2,PSMC3,PSMC4,PSMC5,PSMC6,PSMD1,PSMD2,PSMD3,PSMD4,PSMD5,PSMD7,PSMD8,PSMD9,PSMD10,PSMD11,PSMD12,PSMD13,PSME1,PSME2,PTEN,RELA,RPS27A,SKP1,MAP3K7,TRAF6,UBA52,UBB,UBC,UBE2D1,UBE2D2,UBE2N,UBE2V1,SEM1,CARD11,CUL1,IKBKG,RIPK2,BCL10,BTRC,CD3D,CD3E,CD3G,CD247,CD4,PSMF1,PSMD6,CDC34",Downstream TCR signaling,98

R-HSA-202427,"CSK,PTPN22,HLA-DPA1,HLA-DPB1,HLA-DQA1,HLA-DQA2,HLA-DQB1,HLA-DQB2,HLA-DRA,HLA-DRB1,HLA-DRB3,HLA-DRB4,HLA-DRB5,LCK,PAG1,PTPRC,PTPRJ,CD3D,CD3E,CD3G,CD247,CD4",Phosphorylation of CD3 and TCR zeta chains,22

R-HSA-202430,"PTPN22,HLA-DPA1,HLA-DPB1,HLA-DQA1,HLA-DQA2,HLA-DQB1,HLA-DQB2,HLA-DRA,HLA-DRB1,HLA-DRB3,HLA-DRB4,HLA-DRB5,LCK,ZAP70,CD3D,CD3E,CD3G,CD247,CD4",Translocation of ZAP-70 to Immunological synapse,19

R-HSA-202433,"FYB1,FYB1,LAT,HLA-DPA1,HLA-DPB1,HLA-DQA1,HLA-DQA2,HLA-DQB1,HLA-DQB2,HLA-DRA,HLA-DRB1,HLA-DRB3,HLA-DRB4,HLA-DRB5,ITK,LCK,LCP2,LCP2,NCK1,PAK1,PAK2,PAK3,EVL,PLCG1,PLCG2,ENAH,VASP,WAS,ZAP70,CD3D,CD3E,CD3G,CD247,CD4,CD101,GRAP2",Generation of second messenger molecules,36

R-HSA-2025928,"FKBP1A,NFATC1,NFATC1,NFATC2,NFATC2,NFATC3,NFATC3,PPIA,PPP3CA,PPP3CA,PPP3CB,PPP3CB,PPP3R1,PPP3R1,CALM1,CALM1,CALM2,CALM2,CALM3,CALM3",Calcineurin activates NFAT,20

R-HSA-202670,"DUSP3,DUSP4,DUSP6,DUSP7,VRK3,PPP2CA,PPP2CB,PPP2R1A,PPP2R1B,PPP2R5D,MAPK1,MAPK3,MAPK7",ERKs are inactivated,13

R-HSA-202733,"MERTK,CEACAM5,PROCR,CEACAM3,CEACAM8,SLC7A9,JAML,COL1A1,COL1A2,SIRPA,CXADR,F2,FCER1G,FN1,SLC7A8,SLC16A8,SLC7A11,PPIL2,FYN,GAS6,GLG1,GPC1,ANGPT1,ANGPT1,ANGPT2,GRB2,GRB7,GRB14,VPREB3,GYPA,GYPB,GYPC,HRAS,APOB,JCHAIN,IGLL1,INPP5D,ITGA6,ITGA3,ITGA4,ITGA5,ITGAL,ITGAM,ITGAV,ITGAX,ITGB1,ITGB2,ITGB3,KRAS,L1CAM,LCK,LYN,EPCAM,MAG,CD99,MIF,MMP1,PSG8,CEACAM6,ATP1B1,ATP1B2,ATP1B3,NRAS,OLR1,F11R,GP6,ANGPT4,CD244,PECAM1,PF4,PF4V1,PIK3CA,PIK3CB,PIK3R1,PIK3R2,PLCG1,TREM1,PPIA,SIRPG,PROC,PROS1,SLC7A10,PSG1,PSG2,PSG3,PSG4,PSG5,PSG6,PSG7,PSG9,PSG11,CD177,PTPN6,PTPN11,JAM2,CEACAM1,SDC1,SDC2,SDC4,SELE,SELL,SELP,SELPLG,SHC1,SLC3A2,SLC16A1,SOS1,SPN,SRC,BSG,TEK,TEK,TGFB1,THBD,TSPAN7,VPREB1,YES1,SLC7A5,CD99L2,JAM3,FCAMR,CAV1,TNFRSF10D,TNFRSF10B,TNFRSF10A,CD84,DOK2,SLC7A7,SLC7A6,ESAM,SLC16A3,CD2,PICK1,CD44,CD47,CD48,CD58,SDC3,CD74",Cell surface interactions at the vascular wall,139

R-HSA-2028269,"YAP1,AMOT,AMOTL1,DVL2,WWC1,WWTR1,NPHP4,LATS2,AMOTL2,MOB1A,SAV1,STK3,STK4,TJP1,YWHAB,YWHAE,CASP3,LATS1,MOB1B,TJP2",Signaling by Hippo,20

R-HSA-2029480,"ABI1,ABI1,ARPC5,ARPC5,ARPC4,ARPC4,ARPC3,ARPC3,ARPC1B,ARPC1B,ACTR3,ACTR3,ACTR2,ACTR2,ARPC2,ARPC2,ABI2,ABI2,WASF2,WASF2,VAV3,BAIAP2,BAIAP2,ARPC1A,ARPC1A,CFL1,AHCYL1,NCKAP1,NCKAP1,WASF3,WASF3,PLD4,CRK,WIPF2,WIPF2,DOCK1,PLPP4,FCGR1A,FCGR1A,FCGR2A,FCGR2A,FCGR3A,FCGR3A,FGR,CYFIP1,CYFIP1,PLD3,ABL1,FYN,CYFIP2,CYFIP2,GRB2,GRB2,HCK,NCKAP1L,NCKAP1L,HSP90AA1,HSP90AB1,ITPR1,ITPR2,ITPR3,LIMK1,LIMK1,LYN,MYH2,MYH9,MYO1C,MYO5A,MYO9B,MYO10,NCK1,NCK1,NF2,PAK1,NCKIPSD,NCKIPSD,PIK3CA,PIK3CB,PIK3R1,PIK3R2,PLCG1,PLCG1,PLCG2,PLD1,PLD2,PRKCD,PRKCE,BRK1,BRK1,MAPK1,MAPK3,PTK2,RAC1,RAC1,ACTB,ACTB,ELMO2,WIPF3,WIPF3,SRC,SYK,SYK,BTK,ACTG1,ACTG1,VAV1,VAV2,WAS,WAS,WIPF1,WIPF1,YES1,PLA2G6,PLPP5,WASF1,WASF1,WASL,WASL,CD3G,CD3G,CD247,CD247,ELMO1,CDC42,CDC42",Fcgamma receptor (FCGR) dependent phagocytosis,125

R-HSA-2029481,"FCGR1A,FCGR1A,FCGR2A,FCGR2A,FCGR3A,FCGR3A,FGR,FYN,HCK,LYN,SRC,SYK,SYK,YES1,CD3G,CD3G,CD247,CD247",FCGR activation,18

R-HSA-2029482,"ABI1,ABI1,ARPC5,ARPC5,ARPC4,ARPC4,ARPC3,ARPC3,ARPC1B,ARPC1B,ACTR3,ACTR3,ACTR2,ACTR2,ARPC2,ARPC2,ABI2,ABI2,WASF2,WASF2,VAV3,BAIAP2,BAIAP2,ARPC1A,ARPC1A,CFL1,NCKAP1,NCKAP1,WASF3,WASF3,CRK,WIPF2,WIPF2,DOCK1,FCGR1A,FCGR2A,FCGR3A,CYFIP1,CYFIP1,ABL1,CYFIP2,CYFIP2,GRB2,GRB2,NCKAP1L,NCKAP1L,HSP90AA1,HSP90AB1,LIMK1,LIMK1,MYH2,MYH9,MYO1C,MYO5A,MYO9B,MYO10,NCK1,NCK1,NF2,PAK1,NCKIPSD,NCKIPSD,BRK1,BRK1,MAPK1,MAPK3,PTK2,RAC1,RAC1,ACTB,ACTB,ELMO2,WIPF3,WIPF3,SYK,BTK,ACTG1,ACTG1,VAV1,VAV2,WAS,WAS,WIPF1,WIPF1,WASF1,WASF1,WASL,WASL,CD3G,CD247,ELMO1,CDC42,CDC42",Regulation of actin dynamics for phagocytic cup formation,93

R-HSA-2029485,"AHCYL1,PLD4,PLPP4,FCGR1A,FCGR2A,FCGR3A,PLD3,ITPR1,ITPR2,ITPR3,PIK3CA,PIK3CB,PIK3R1,PIK3R2,PLCG1,PLCG1,PLCG2,PLD1,PLD2,PRKCD,PRKCE,SYK,PLA2G6,PLPP5,CD3G,CD247",Role of phospholipids in phagocytosis,26

R-HSA-2032785,"YAP1,NKX2-5,CCN2,HIPK1,WWTR1,WWTR1,GATA4,HIPK2,NPPA,TBX5,TEAD1,TEAD4,TEAD3,TEAD2,RUNX2,KAT2B",YAP1- and WWTR1 (TAZ)-stimulated gene expression,16

R-HSA-2033514,"FGF1,FGF2,FGF4,FGF5,FGF8,FGF9,FGFR3,FGF20,FGF23,FGF18,FGF17,FGF16",FGFR3 mutant receptor activation,12

R-HSA-2033515,FGFR3,t(4;14) translocations of FGFR3,1

R-HSA-2033519,"FGF1,FGF2,FGF3,FGF4,FGF5,FGF6,FGF7,FGF8,FGF9,FGF10,FGFR2,FGF20,FGF22,FGF23,FGF18,FGF17,FGF16",Activated point mutants of FGFR2,17

R-HSA-203615,"LYPLA1,CYGB,AKT1,DDAH2,DDAH1,HSP90AA1,ZDHHC21,NOS3,SPR,CALM1,CALM2,CALM3,CAV1",eNOS activation,13

R-HSA-203641,"NOSTRIN,DNM2,NOS3,CAV1,WASL",NOSTRIN mediated eNOS trafficking,5

R-HSA-203754,"NOS3,NOSIP",NOSIP mediated eNOS trafficking,2

R-HSA-203927,"BCDIN3D,AGO3,AGO4,DICER1,AGO1,AGO2,DROSHA,POLR2A,POLR2B,POLR2C,POLR2D,POLR2E,POLR2F,POLR2G,POLR2H,POLR2I,POLR2J,POLR2K,POLR2L,DGCR8,XPO5,RAN,TARBP2,PRKRA",MicroRNA (miRNA) biogenesis,24

R-HSA-204005,"PREB,CNIH1,BET1,TFG,SEC24B,SEC23A,YKT6,CTSC,SEC24A,TMED2,LMAN2,TMED10,SEC23IP,TRAPPC6B,TRAPPC5,TBC1D20,COL7A1,CSNK1D,CNIH3,CTSZ,F5,F8,PPP6R1,SEC31A,ANKRD28,SCFD1,FOLR1,CNIH2,SEC22A,TRAPPC3,GOLGA2,GRIA1,AREG,LMAN1,NSF,SAR1B,TRAPPC4,TRAPPC2L,SERPINA1,STX17,PPP6R3,PPP6C,TRAPPC1,RAB1A,NAPB,SEC13,TRAPPC2,GORASP1,STX5,TGFA,TRAPPC10,TRAPPC6A,LMAN1L,LMAN2L,RAB1B,TRAPPC9,USO1,NAPG,NAPA,SEC16B,MCFD2,SEC22C,SEC22B,GOSR2,SEC24C,CD59,SEC24D,SEC16A",COPII-mediated vesicle transport,68

R-HSA-204174,"DLAT,DLD,GSTZ1,PDHA1,PDHA2,PDHB,PDK1,PDK2,PDK3,PDK4,PPARD,PDP1,PDPR,PDP2,RXRA,PDHX",Regulation of pyruvate dehydrogenase (PDH) complex,16

R-HSA-2046104,"ACOT8,ABCD1,ACSL1,ACAA1,HSD17B4,FADS1,ACOX1,ELOVL2,ELOVL5,SCP2,ELOVL1,ELOVL3,FADS2,FADS2",alpha-linolenic (omega3) and linoleic (omega6) acid metabolism,14

R-HSA-2046105,"ABCD1,ACSL1,FADS1,ELOVL2,ELOVL5,ELOVL1,ELOVL3,FADS2",Linoleic acid (LA) metabolism,8

R-HSA-2046106,"ACOT8,ABCD1,ACSL1,ACAA1,HSD17B4,FADS1,ACOX1,ELOVL2,ELOVL5,SCP2,ELOVL1,ELOVL3,FADS2,FADS2",alpha-linolenic acid (ALA) metabolism,14

R-HSA-204626,"DHPS,EIF5A,EIF5A2,DOHH",Hypusine synthesis from eIF5A-lysine,4

R-HSA-204998,"BCL2L11,ARHGEF33,NET1,VAV3,GNA13,AKAP13,FGD4,ARHGEF19,ECT2,FGD2,FGD1,ARHGEF15,ARHGEF9,MCF2L,ARHGEF12,ARHGEF18,NCSTN,ITGB3BP,ITGB3BP,NGEF,ARHGEF26,TIAM2,AATF,BEX3,ARHGEF16,ABR,ARHGEF37,MCF2,ARHGEF35,NGF,NGF,NGFR,NGFR,ARHGEF4,ARHGEF3,APH1A,ARHGEF38,ARHGEF10L,ARHGEF40,PSENEN,MAPK8,MAPK8,PSEN1,PSEN2,BAD,PLEKHG5,PREX1,RAC1,RASGRF2,RPS27A,ITSN1,PLEKHG2,SOS1,SOS2,TIAM1,TRAF6,TRAF6,TRIO,UBA52,UBB,UBC,VAV1,VAV2,YWHAE,ARHGEF5,APH1B,CASP2,CASP3,OBSCN,ARHGEF39,ARHGEF7,SQSTM1,FGD3,KALRN,ARHGEF1,ARHGEF2,ARHGEF6,MAGED1,ARHGEF10,ARHGEF11,ARHGEF17","Cell death signalling via NRAGE, NRIF and NADE",81

R-HSA-205017,"NGF,NGFR",NFG and proNGF binds to p75NTR,2

R-HSA-205025,"BEX3,NGF,NGFR,YWHAE,CASP2,CASP3",NADE modulates death signalling,6

R-HSA-205043,"NCSTN,ITGB3BP,ITGB3BP,NGF,NGF,NGFR,NGFR,APH1A,PSENEN,MAPK8,PSEN1,PSEN2,RPS27A,TRAF6,TRAF6,UBA52,UBB,UBC,APH1B,SQSTM1",NRIF signals cell death from the nucleus,20

R-HSA-209543,"IKBKB,IRAK1,IRAK1,MYD88,MYD88,NGF,NGF,NGFR,NGFR,PRKCI,RPS27A,TRAF6,UBA52,UBB,UBC,RIPK2,SQSTM1",p75NTR recruits signalling complexes,17

R-HSA-209560,"IKBKB,IRAK1,NFKB1,NFKBIA,NGF,NGFR,RELA,RPS27A,TRAF6,UBA52,UBB,UBC,SQSTM1",NF-kB is activated and signals survival,13

R-HSA-209563,"RHOA,ARHGDIA,NGF,NGFR",Axonal growth stimulation,4

R-HSA-209776,"CGA,TPH2,AANAT,DBH,DDC,DIO1,DIO2,DIO3,IYD,ASMT,DUOX2,DUOX1,PNMT,SLC5A5,TH,TPH1,TPO,TSHB",Metabolism of amine-derived hormones,18

R-HSA-209822,"CGA,CGB3,FSHB,INHA,INHBA,INHBB,INHBC,LHB,TSHB,INHBE,CGB5,CGB8",Glycoprotein hormones,12

R-HSA-209905,"DBH,DDC,PNMT,TH",Catecholamine biosynthesis,4

R-HSA-209931,"TPH2,AANAT,DDC,ASMT,TPH1",Serotonin and melatonin biosynthesis,5

R-HSA-209952,"CGA,CGB3,FSHB,INHA,INHBA,INHBB,INHBC,LHB,PCSK1,POMC,TSHB,INHBE,CGB5,CGB8",Peptide hormone biosynthesis,14

R-HSA-209968,"CGA,DIO1,DIO2,DIO3,IYD,DUOX2,DUOX1,SLC5A5,TPO,TSHB",Thyroxine biosynthesis,10

R-HSA-210455,"GLUL,SLC1A2,SLC1A3,SLC38A1",Astrocytic Glutamate-Glutamine Uptake And Metabolism,4

R-HSA-210500,"UNC13B,ARL6IP5,CPLX1,RIMS1,GLS2,GLS,SLC38A2,SLC17A7,RAB3A,SLC1A1,SLC1A2,SLC1A3,SLC1A6,SLC1A7,SNAP25,STX1A,STXBP1,VAMP2,SYT1,PPFIA4,PPFIA2,PPFIA1,PPFIA3,TSPOAP1",Glutamate Neurotransmitter Release Cycle,24

R-HSA-210744,"MAMLD1,CREBBP,EP300,SNW1,KAT2A,ONECUT1,HES1,RBPJ,ONECUT3,NOTCH1,NEUROG3,MAML3,HNF1B,MAML2,KAT2B,MAML1",Regulation of gene expression in late stage (branching morphogenesis) pancreatic bud precursor cells,16

R-HSA-210745,"AKT3,AKT1,AKT2,RFX6,FOXO1,FOXO1,GCK,FOXA2,FOXA3,HNF4A,HNF4G,IAPP,INS,PDX1,MAFA,NEUROD1,NKX2-2,NKX6-1,PAX6,PKLR,SLC2A2,HNF1A",Regulation of gene expression in beta cells,22

R-HSA-210746,"INSM1,NEUROD1,NKX2-2,NEUROG3",Regulation of gene expression in endocrine-committed (NEUROG3+) progenitor cells,4

R-HSA-210747,"FGF10,NR5A2,PTF1A,ONECUT1,PDX1,ONECUT3,NKX6-1,HNF1B",Regulation of gene expression in early pancreatic precursor cells,8

R-HSA-210990,"FYN,INPP5D,ITGAV,ITGB3,LCK,LYN,PECAM1,PLCG1,PTPN6,PTPN11,SRC,YES1",PECAM1 interactions,12

R-HSA-210991,"SLC7A9,SLC7A8,SLC16A8,SLC7A11,PPIL2,ITGA6,ITGA3,ITGB1,L1CAM,MAG,MMP1,ATP1B1,ATP1B2,ATP1B3,PPIA,SLC7A10,SLC3A2,SLC16A1,SPN,BSG,SLC7A5,CAV1,SLC7A7,SLC7A6,SLC16A3",Basigin interactions,25

R-HSA-210993,"ANGPT1,ANGPT1,ANGPT2,GRB2,GRB7,GRB14,HRAS,KRAS,NRAS,ANGPT4,PIK3CA,PIK3CB,PIK3R1,PIK3R2,PTPN11,SHC1,SOS1,TEK,TEK,DOK2",Tie2 Signaling,20

R-HSA-211000,"LOC102724334,IPO8,NUP50,TDRKH,NUP42,HENMT1,H4-16,TDRD9,H3C14,H2BU1,NUP35,ASZ1,PIWIL4,BCDIN3D,AGO3,AGO4,PLD6,TDRD6,TNRC6B,NUP205,NUP210,NUP160,DICER1,NUP188,NUP62,H2BC1,AGO1,AGO2,TNRC6A,DROSHA,H2AC8,H2AC7,H2AX,H2AZ1,H2BC5,H2BC3,H3-3A,H3-3B,HSP90AA1,H3C15,NUP43,MYBL1,H2AB1,NUP88,NUP98,NUP54,POLR2A,POLR2A,POLR2B,POLR2B,POLR2C,POLR2C,POLR2D,POLR2D,POLR2E,POLR2E,POLR2F,POLR2F,POLR2G,POLR2G,POLR2H,POLR2H,POLR2I,POLR2I,POLR2J,POLR2J,POLR2K,POLR2K,POLR2L,POLR2L,MOV10L1,DGCR8,DDX4,PIWIL2,H4C15,NDC1,NUP133,H2AJ,TDRD1,NUP107,XPO5,TNRC6C,RAN,RANBP2,SEC13,H3C13,TARBP2,TPR,H2AC19,TSN,TSNAX,NUP37,NUP85,NUP214,AAAS,SEH1L,H4C9,H2AC14,H2AC6,H2AC4,H2AC18,H2AC20,H2BC8,H2BC13,H2BC15,H2BC14,H2BC7,H2BC6,H2BC9,H2BC10,H2BC4,H2BC17,H2BC21,H3C1,H3C4,H3C3,H3C6,H3C11,H3C8,H3C12,H3C10,H3C2,H4C1,H4C4,H4C6,H4C12,H4C11,H4C3,H4C8,H4C2,H4C5,H4C13,H4C14,FKBP6,RAE1,MAEL,H2BC12,PRKRA,H3C7,H2BC11,TDRD12,PIWIL1,H2AZ2,NUP155,NUP93,NUP58,POM121,NUP153",Gene Silencing by RNA,148

R-HSA-211163,"AKT3,AKT1,AKT2,FOXO1",AKT-mediated inactivation of FOXO1A,4

R-HSA-211728,"PAK2,ARHGAP10",Regulation of PAK-2p34 activity by PS-GAP/RHG10,2

R-HSA-211733,"PSME3,PSMD14,PSMB11,PSMA8,PSME4,PAK2,PSMA1,PSMA2,PSMA3,PSMA4,PSMA5,PSMA6,PSMA7,PSMB1,PSMB2,PSMB3,PSMB4,PSMB5,PSMB6,PSMB7,PSMB8,PSMB9,PSMB10,PSMC1,PSMC2,PSMC3,PSMC4,PSMC5,PSMC6,PSMD1,PSMD2,PSMD3,PSMD4,PSMD5,PSMD7,PSMD8,PSMD9,PSMD10,PSMD11,PSMD12,PSMD13,PSME1,PSME2,RPS27A,UBA52,UBB,UBC,SEM1,PSMF1,PSMD6",Regulation of activated PAK-2p34 by proteasome mediated degradation,50

R-HSA-211736,"PAK2,CASP3",Stimulation of the cell death response by PAK-2p34,2

R-HSA-211859,"NAT2,CYP3A7-CYP3A51P,GLYAT,AKR1A1,BPNT1,NCOA2,CES1,UGT2B11,PTGES3,CYP46A1,SLC26A1,UGT2A1,FDX2,CYP4F8,CYP2U1,ACSM1,GSTO2,CYP2R1,ACSM2A,ADH1A,GGT6,ADH1B,ADH1C,CYP4F22,ADH4,ADH5,AADAC,ADH6,ADH7,COMT,UGT3A1,CMBL,CYP1A1,CYP1A2,CYP1B1,CYP2A6,CYP2A7,CYP3A7,CYP2A13,CYP2B6,CYP2C19,CYP2C8,CYP2C9,CYP2C18,CYP2D6,CYP2E1,CYP2F1,CYP2J2,CYP3A4,CYP3A5,CYP4A11,CYP4B1,CYP7A1,CYP8B1,CYP11A1,CYP11B1,CYP11B2,CYP19A1,CYP21A2,CYP24A1,CYP26A1,CYP27A1,CYP27B1,CYP51A1,UGT3A2,CYB5R3,DPEP1,SLC26A2,AHCY,AHR,PAOX,EPHX1,ESD,ALDH1A1,ALDH2,ALDH3A1,ALDH1B1,GLYATL2,GSTA5,FDX1,FDXR,AKR7A3,SLC35D1,FMO1,FMO2,FMO3,CES3,AKR7L,SULT4A1,AOC1,GGT1,GGT7,GGT5,OPLAH,SULT1C4,SULT1B1,GCLC,GCLM,HPGDS,MAT2B,CYP4A22,CYP4V2,N6AMT1,GSS,GSTA1,GSTA2,GSTA3,GSTA4,GSTM1,GSTM2,GSTM3,GSTM4,GSTM5,GSTP1,GSTT1,GSTT2,GSTZ1,CYP2S1,AOC2,HSP90AB1,CYP26C1,ACSM4,SLC35B2,ACSM2B,GSTK1,GLYATL3,SULT6B1,ARNT,CYP4F3,MAOA,MAOB,MAT1A,MAT2A,MGST1,MGST2,MGST3,SULT1A4,MTR,MTRR,NQO2,NNMT,CHAC2,PODXL2,SLC35B3,CYP39A1,TRMT112,POMC,POR,UGT2B28,SMOX,UGT1A10,UGT1A8,UGT1A7,UGT1A6,UGT1A5,UGT1A9,UGT1A4,UGT1A1,UGT1A3,CYP2W1,IMPAD1,ACSM5,MTARC2,ABHD10,CNDP2,ACSS2,CYP26B1,PTGIS,AS3MT,PTGS1,UGT2A2,AHRR,CYP4F11,RXRA,DPEP2,DPEP3,MTARC1,CYP3A43,GSTT2B,CYP4F12,BPHL,SULT1E1,SULT1A2,SULT1A1,SULT1A3,SULT1C2,SULT2B1,SULT2A1,TBXAS1,TPMT,UGDH,UGP2,UGT2B4,UGT2B7,UGT2B10,UGT2B15,UGT2B17,GGCT,CHAC1,UGT2A3,CYB5B,ACSS1,TPST2,TPST1,ABHD14B,CYP4F2,AKR7A2,AOC3,NCOA1,CBR3,CES2,NAT1,AIP,PAPSS2,PAPSS1,ACY3,GLYATL1,CYP7B1,GSTO1,ACY1,ARNT2,NR1H4",Biological oxidations,222

R-HSA-211897,"CYP3A7-CYP3A51P,NCOA2,CYP46A1,FDX2,CYP4F8,CYP2U1,CYP2R1,CYP4F22,CYP1A1,CYP1A2,CYP1B1,CYP2A6,CYP2A7,CYP3A7,CYP2A13,CYP2B6,CYP2C19,CYP2C8,CYP2C9,CYP2C18,CYP2D6,CYP2E1,CYP2F1,CYP2J2,CYP3A4,CYP3A5,CYP4A11,CYP4B1,CYP7A1,CYP8B1,CYP11A1,CYP11B1,CYP11B2,CYP19A1,CYP21A2,CYP24A1,CYP26A1,CYP27A1,CYP27B1,CYP51A1,AHR,FDX1,FDXR,CYP4A22,CYP4V2,CYP2S1,CYP26C1,ARNT,CYP4F3,CYP39A1,POMC,POR,CYP2W1,CYP26B1,PTGIS,AHRR,CYP4F11,RXRA,CYP3A43,CYP4F12,TBXAS1,CYP4F2,NCOA1,CYP7B1,ARNT2,NR1H4",Cytochrome P450 - arranged by substrate type,66

R-HSA-211916,"CYP2R1,CYP24A1,CYP26A1,CYP27B1,CYP26C1,CYP26B1",Vitamins,6

R-HSA-211935,"CYP4F8,CYP4F22,CYP2A7,CYP2A13,CYP2B6,CYP2D6,CYP2F1,CYP2J2,CYP4A11,CYP4B1,CYP4A22,CYP4F3,CYP4F11,CYP4F12,CYP4F2",Fatty acids,15

R-HSA-211945,"CYP3A7-CYP3A51P,NCOA2,CES1,PTGES3,CYP46A1,FDX2,CYP4F8,CYP2U1,CYP2R1,ADH1A,ADH1B,ADH1C,CYP4F22,ADH4,ADH5,AADAC,ADH6,ADH7,CMBL,CYP1A1,CYP1A2,CYP1B1,CYP2A6,CYP2A7,CYP3A7,CYP2A13,CYP2B6,CYP2C19,CYP2C8,CYP2C9,CYP2C18,CYP2D6,CYP2E1,CYP2F1,CYP2J2,CYP3A4,CYP3A5,CYP4A11,CYP4B1,CYP7A1,CYP8B1,CYP11A1,CYP11B1,CYP11B2,CYP19A1,CYP21A2,CYP24A1,CYP26A1,CYP27A1,CYP27B1,CYP51A1,CYB5R3,AHR,PAOX,EPHX1,ALDH1A1,ALDH2,ALDH3A1,ALDH1B1,FDX1,FDXR,FMO1,FMO2,FMO3,CES3,AOC1,CYP4A22,CYP4V2,CYP2S1,AOC2,HSP90AB1,CYP26C1,ARNT,CYP4F3,MAOA,MAOB,NQO2,CYP39A1,POMC,POR,SMOX,CYP2W1,MTARC2,ACSS2,CYP26B1,PTGIS,PTGS1,AHRR,CYP4F11,RXRA,MTARC1,CYP3A43,CYP4F12,BPHL,TBXAS1,CYB5B,ACSS1,CYP4F2,AOC3,NCOA1,CBR3,CES2,AIP,CYP7B1,ARNT2,NR1H4",Phase I - Functionalization of compounds,106

R-HSA-211957,CYP1A2,Aromatic amines can be N-hydroxylated or N-dealkylated by CYP1A2,1

R-HSA-211958,"CYP2U1,CYP4F22,CYP2D6,CYP4A11,CYP4B1,CYP4A22,CYP2S1,CYP4F3,CYP2W1,CYP4F11,CYP3A43,CYP4F2",Miscellaneous substrates,12

R-HSA-211976,"NCOA2,CYP46A1,FDX2,CYP1B1,CYP7A1,CYP8B1,CYP11A1,CYP11B1,CYP11B2,CYP19A1,CYP21A2,CYP27A1,CYP51A1,AHR,FDX1,FDXR,CYP4V2,ARNT,CYP39A1,POMC,PTGIS,AHRR,RXRA,NCOA1,CYP7B1,ARNT2,NR1H4",Endogenous sterols,27

R-HSA-211979,"CYP4F8,CYP4F22,CYP4A11,CYP4B1,CYP8B1,CYP4A22,CYP4F3,PTGIS,CYP4F11,CYP4F12,TBXAS1,CYP4F2",Eicosanoids,12

R-HSA-211981,"CYP3A7-CYP3A51P,CYP1A1,CYP1A2,CYP2A6,CYP2A7,CYP3A7,CYP2A13,CYP2B6,CYP2C19,CYP2C8,CYP2C9,CYP2C18,CYP2D6,CYP2E1,CYP2F1,CYP2J2,CYP3A4,CYP3A5,AHR,CYP2S1,ARNT,CYP2W1,AHRR,CYP3A43,ARNT2",Xenobiotics,25

R-HSA-211994,"CYP8B1,PTGIS",Sterols are 12-hydroxylated by CYP8B1,2

R-HSA-211999,"CYP2A6,CYP2A7,CYP2A13,CYP2B6,CYP2C19,CYP2C8,CYP2C9,CYP2D6,CYP2E1,CYP2F1,CYP2S1",CYP2E1 reactions,11

R-HSA-212165,"RNA45SN5,CDK7,LOC102724334,LOC102724334,SAP18,MYBBP1A,CD3EAP,CD3EAP,EHMT2,CHD3,CHD4,BAZ2A,CBX3,H4-16,H4-16,AEBP2,H3C14,H3C14,H2BU1,H2BU1,DNMT1,DNMT1,DNMT3A,DNMT3A,DNMT3B,DNMT3B,TET3,EP300,ERCC2,ERCC3,ERCC6,ERCC6,EZH2,TWISTNB,TWISTNB,MTF2,SIN3B,RRP8,SIRT1,SIRT1,SF3B1,SUZ12,H2BC1,H2BC1,POLR1A,POLR1A,SIN3A,PHF19,KAT2A,SAP30BP,UHRF1,GSK3B,GTF2H1,GTF2H2,GTF2H3,GTF2H4,DNMT3L,H2AC8,H2AC8,H2AC7,H2AC7,H2AX,H2AX,H2AZ1,H2AZ1,H2BC5,H2BC5,H2BC3,H2BC3,H3-3A,H3-3A,H3-3B,H3-3B,HDAC1,HDAC2,ZNRD1,ZNRD1,H3C15,H3C15,JARID2,GTF2H5,MNAT1,MYO1C,H2AB1,H2AB1,POLR1D,POLR1D,ARID4B,PHF1,MBD3,POLR2E,POLR2E,POLR2F,POLR2F,POLR2H,POLR2H,POLR2K,POLR2K,POLR2L,POLR2L,TET2,GATAD2A,H4C15,H4C15,H2AJ,H2AJ,GATAD2B,MTA3,RBBP4,RBBP4,RBBP7,RBBP7,ACTB,POLR1E,POLR1E,SUDS3,H3C13,H3C13,SUV39H1,TBP,TBP,TDG,H2AC19,H2AC19,TTF1,UBTF,TAF1D,TAF1D,DEK,SAP130,SAP30L,TET1,H4C9,H4C9,H2AC14,H2AC14,H2AC6,H2AC6,H2AC4,H2AC4,H2AC18,H2AC18,H2AC20,H2AC20,H2BC8,H2BC8,H2BC13,H2BC13,H2BC15,H2BC15,H2BC14,H2BC14,H2BC7,H2BC7,H2BC6,H2BC6,H2BC9,H2BC9,H2BC10,H2BC10,H2BC4,H2BC4,H2BC17,H2BC17,H2BC21,H2BC21,H3C1,H3C1,H3C4,H3C4,H3C3,H3C3,H3C6,H3C6,H3C11,H3C11,H3C8,H3C8,H3C12,H3C12,H3C10,H3C10,H3C2,H3C2,H4C1,H4C1,H4C4,H4C4,H4C6,H4C6,H4C12,H4C12,H4C11,H4C11,H4C3,H4C3,H4C8,H4C8,H4C2,H4C2,H4C5,H4C5,H4C13,H4C13,H4C14,H4C14,POLR1B,POLR1B,SMARCA5,SMARCA5,H2BC12,H2BC12,EED,SAP30,KAT2B,MBD2,H3C7,H3C7,H2BC11,H2BC11,TAF1C,TAF1C,TAF1B,TAF1B,TAF1A,TAF1A,CCNH,BAZ1B,MTA1,DDX21,MTA2,H2AZ2,H2AZ2,POLR1C,POLR1C",Epigenetic regulation of gene expression,235

R-HSA-2122947,"HDAC6,HDAC5,MAMLD1,MAMLD1,CDK8,CREBBP,CREBBP,EP300,EP300,SNW1,SNW1,HEY1,HEY2,KAT2A,KAT2A,HEYL,NBEA,HDAC1,HDAC2,HIF1A,HES1,RBPJ,RBPJ,HES5,MYC,NOTCH1,NOTCH1,HDAC7,FBXW7,MAML3,MAML3,HDAC8,RPS27A,SKP1,TBL1X,TLE1,TLE2,TLE3,TLE4,UBA52,UBB,UBC,TBL1XR1,HDAC11,HDAC10,MAML2,MAML2,CUL1,HDAC3,KAT2B,KAT2B,CCNC,NCOR1,NCOR2,HDAC9,HDAC4,MAML1,MAML1,RBX1",NOTCH1 Intracellular Domain Regulates Transcription,59

R-HSA-2122948,"ADAM10,DTX2,DTX2,CNTN1,MIB2,JAG1,DTX1,DTX1,DTX4,DTX4,NCSTN,DLL1,JAG2,JAG2,ARRB1,ARRB2,NOTCH1,NOTCH1,APH1A,NEURL1B,DLL4,PSENEN,PSEN1,PSEN2,MIB1,RPS27A,RPS27A,ADAM17,UBA52,UBA52,UBB,UBB,UBC,UBC,APH1B,ITCH,ITCH,NUMB,DLK1,NEURL1,DNER",Activated NOTCH1 Transmits Signal to the Nucleus,41

R-HSA-212300,"LOC102724334,H4-16,AEBP2,H3C14,H2BU1,DNMT1,DNMT3A,DNMT3B,EZH2,MTF2,SUZ12,H2BC1,PHF19,H2AC8,H2AC7,H2AX,H2AZ1,H2BC5,H2BC3,H3-3A,H3-3B,H3C15,JARID2,H2AB1,PHF1,H4C15,H2AJ,RBBP4,RBBP7,H3C13,H2AC19,H4C9,H2AC14,H2AC6,H2AC4,H2AC18,H2AC20,H2BC8,H2BC13,H2BC15,H2BC14,H2BC7,H2BC6,H2BC9,H2BC10,H2BC4,H2BC17,H2BC21,H3C1,H3C4,H3C3,H3C6,H3C11,H3C8,H3C12,H3C10,H3C2,H4C1,H4C4,H4C6,H4C12,H4C11,H4C3,H4C8,H4C2,H4C5,H4C13,H4C14,H2BC12,EED,H3C7,H2BC11,H2AZ2",PRC2 methylates histones and DNA,73

R-HSA-212436,"AKT3,AKT3,MED6,NR2E3,ZNF730,ZNF737,HDAC6,HDAC6,ZNF705E,ZNF705G,HDAC5,BCL2L11,BCL2L11,MED16,ZNF729,ZNF605,MAMLD1,MAMLD1,OCLN,ELOA3D,RBM14-RBM4,COMMD3-BMI1,NR1H3,RAD50,ZNF263,LRPPRC,YAF2,TRIM28,ZNF197,CDK2,ZNF256,CDK4,PSME3,PSME3,CDK5,CDK6,PSMD14,PSMD14,CDK7,ZNF443,CDK8,CDK9,CDKN1A,CDKN1B,CDKN1B,LOC102724334,LOC102724334,STUB1,CDKN2A,CDKN2B,ZNF267,RRAGB,CITED2,ANAPC10,NDRG1,MYL9,YAP1,YAP1,PRMT5,RBM14,CARM1,CEBPB,ZNF211,KAT5,LAMTOR5,TXNIP,PRELID3A,RRAGA,KDM5B,PLK2,ZNF234,ZNF266,ZNF274,ZNF273,ZNF460,ZNF268,CGA,CGB3,PPP1R13L,PPARGC1A,PPARGC1A,EHMT2,YWHAQ,WWP1,UBE2C,CHD3,TOPBP1,CHD4,WDR5,PRDM7,CHEK1,SUPT16H,CHEK2,CHM,TP53RK,MED8,CBX3,ZNF257,ZIM3,GPRIN1,FBXO32,ZNF554,ZNF689,RMI2,COX20,TWIST2,RFFL,ZNF354B,ANAPC16,ZNF641,SP7,H4-16,H4-16,PSMB11,PSMB11,SOCS4,ZNF720,TMEM219,ZNF543,ZNF441,ZNF440,ZNF573,PLK3,ZNF792,NR2C2AP,H3C14,H3C14,ZNF684,COL1A1,H2BU1,H2BU1,COX4I1,COX5B,SETD9,PPARGC1B,PPARGC1B,COX6A1,JMY,COX6B1,COX6C,COX7B,COX7C,COX8A,COX11,ZNF786,CR1,TAF1L,CREB1,ATF2,CREBBP,CREBBP,CRH,ZFP28,PARP1,MAPK14,PSMA8,PSMA8,CSF1R,SESN3,CSF2,ZNF664,E2F7,RAD9B,CSNK2A1,CSNK2A2,CSNK2B,ZFP90,ZNF597,ZNF785,ZNF688,ZNF480,ZNF418,ZNF417,ZNF548,ZNF560,ZNF563,ZNF420,ZNF565,ZNF582,ZNF583,ZNF599,ZNF558,NKX2-5,ZNF714,ZNF681,ZNF555,ZNF569,ZNF570,CCN2,CTLA4,CTNNB1,CTSK,CTSL,CTSV,ZNF595,SLC38A9,ZNF425,ZNF746,ZNF483,ZNF782,DAXX,ZFPM1,ZFP1,ZNF519,ELOA3,ZNF610,ZNF600,ZNF320,ZNF550,ZNF791,ZNF564,ZNF709,ZNF433,ZNF114,ZNF567,ZNF383,ZNF676,ZNF100,ZNF540,CITED4,DDB2,GADD45A,GADD45A,DDIT3,DDIT3,ZNF92,ZNF679,ZNF596,ZNF169,ZNF431,ZNF721,ZNF675,DLX5,DLX6,DNA2,AGRP,JAG1,ARID3A,E2F1,E2F4,E2F5,E2F6,NR0B1,PHC1,PHC2,AGO3,AGO3,AGO4,AGO4,EGFR,ARID2,ZNF778,ZNF627,ELF1,ZNF585A,ZNF626,ELF2,KCTD6,ZNF584,EP300,EP300,HIPK1,NR2F6,ERBB2,ERCC2,AKT1,AKT1,ERCC3,AKT2,AKT2,ESR1,ESR1,ESR2,ESRRA,ESRRA,ESRRB,ESRRG,EZH2,FANCC,FANCD2,ZNF804B,ZNF25,ZNF485,UCMA,ZSCAN25,ZFP30,ZNF510,FKBP5,FOXG1,FOXG1,SNW1,SNW1,SCMH1,TPX2,CNOT1,SETD1B,FOXO1,FOXO1,FOXO3,FOXO3,TNRC6B,TNRC6B,PSME4,PSME4,MGA,SIN3B,SATB2,NEDD4L,PPP1R13B,SIRT3,SIRT3,SIRT1,SIRT1,RYBP,ATP1B4,ABCA6,HEY1,CBX6,CBX5,HEY2,SUZ12,FOS,ZIM2,ZKSCAN5,BRD1,CNOT6L,CDC26,MTOR,NR5A2,ABL1,ABL1,NR5A1,RICTOR,ZNF620,G6PC,G6PD,ZNF718,H2BC1,H2BC1,ZNF549,GAD1,GAD2,ZNF324,PRDX5,ZNF473,CNOT10,ANAPC15,NELFB,ZNF521,GAMT,WWTR1,WWTR1,SIN3A,SIN3A,ZNF385A,L3MBTL1,ZNF500,AUTS2,ZNF658,ZNF337,NOC2L,GATA1,GATA2,GATA3,GATA4,GCK,KAT2A,KAT2A,NR6A1,AGO1,AGO1,MSTN,ZNF285,BBC3,ZNF777,BRPF3,AGO2,AGO2,GLS2,PRELID1,SESN1,VENTX,ZNF544,TNRC6A,TNRC6A,UBE2S,GLI2,GLI3,GLS,SFN,GP1BA,GPI,ZNF311,ZNF740,KCTD1,ZNF547,ZIK1,ZNF776,ZNF615,ZNF493,ZNF875,DLL1,ZNF619,ZNF621,ZNF660,COX18,ZNF454,ZNF775,ZNF707,GPS2,GPX2,GRIA2,LAMTOR2,RGCC,HIPK2,ATAD2,GRIN2A,GRIN2B,MED4,NR3C1,NR3C1,BRD7,GSK3B,GSR,GTF2F1,GTF2F2,GTF2H1,GTF2H2,GTF2H3,GTF2H4,ANAPC2,CNOT7,ANAPC4,NRBP1,NRBF2,H2AC8,H2AC8,H2AC7,H2AC7,H2AX,H2AX,H2AZ1,H2AZ1,H2BC5,H2BC5,H2BC3,H2BC3,H3-3A,H3-3A,H3-3B,H3-3B,HTT,HDAC1,HDAC1,HDAC2,HDAC2,ZNF354C,NR4A1,APAF1,HNF4A,HNF4G,PRMT1,HES1,HES1,BIRC5,HSPD1,H3C15,H3C15,HUS1,ZNF546,TFAP2E,TFAP2E,ZFP69,ZNF680,ZNF517,ZNF774,ZNF677,ZNF860,IFNG,APOE,ZNF530,IGFBP1,IGFBP3,RBPJ,RBPJ,ZNF429,ZNF445,ZNF233,IHH,FAS,IL2,IL2RA,FASLG,IL3,IL6,ING2,INS,INS,IRAK1,AR,AR,ITGA2B,ITGA4,ITGA5,ITGAL,JUN,JUNB,ZNF710,ZNF699,ZNF568,ZNF773,KIT,KRAS,ZKSCAN4,KMT5A,ZNF790,ZNF761,ZNF470,ZNF749,ZNF324B,ZNF662,LAMTOR4,ZNF793,LBR,LGALS3,LIFR,LMO1,LMO2,ZNF772,GTF2H5,SMAD1,SMAD2,SMAD2,SMAD3,SMAD3,SMAD4,SMAD4,SMAD6,SMAD7,SMAD7,MAF,MAX,MDM2,MDM4,MECP2,MECP2,MEF2C,MEN1,MET,ATXN3,MLH1,KMT2A,KMT2A,FOXO4,FOXO4,NR3C2,MMP13,MNAT1,MOBP,MOV10,MOV10,MRE11,ZNF705A,ZNF506,ZNF716,ZNF727,CITED1,MSH2,MSX2,COX1,COX2,COX3,MYB,MYBL2,MYC,ZFHX3,NBN,NDUFA4,ATM,H2AB1,H2AB1,NFATC2,NFE2,NFKB1,NFYA,NFYB,NFYC,NOP2,CNOT2,CNOT3,CNOT4,NOTCH1,NOTCH1,NPY,NOTCH2,NOTCH3,NOTCH4,NPM1,NPPA,NR4A2,OPRK1,OPRM1,RRM2B,PRDX1,SERPINE1,PAX5,FOXP3,MED31,PCK1,ZNF691,PCNA,ZNF706,LEF1,TACO1,ELOA2,PHF20,COX16,ZNF571,ZNF771,FZR1,ZNF589,ACTL6B,PRKAG2,ANAPC5,ANAPC7,NELFCD,TRIAP1,ANAPC11,HDAC7,MED15,TRIM33,TAF9B,PDPK1,WWOX,CDK12,PF4,PGR,SERPINB13,PIN1,PIP4K2A,PITX2,PLAGL1,MBD3,PRKAG3,PMAIP1,PML,PMS2,CYCS,POLR2A,POLR2B,POLR2C,POLR2D,POLR2E,POLR2F,POLR2G,POLR2H,POLR2I,POLR2J,POLR2K,POLR2L,POMC,TAF7L,DGCR8,ATR,DDIT4,POU4F1,POU4F2,PPARA,PPARD,PPARG,PPARG,MED1,RBFOX1,RNF111,RNF111,ZNF586,ZNF562,GATAD2A,ZSCAN32,PPM1A,BANP,ZNF770,LAMTOR1,PPP2CA,PPP2CB,PRMT6,PPP2R1A,PPP2R1B,PBRM1,FANCI,STEAP3,PPP2R5C,FBXW7,PIDD1,ZNF331,H4C15,H4C15,MAML3,MAML3,CNOT11,PRR5,PRKAA1,PRKAA2,KRBOX4,PRKAB1,PRKAB2,ZNF692,ZNF416,PRKACA,ZNF446,PRKAG1,ZNF334,ZNF701,H2AJ,H2AJ,ZNF839,ZNF415,PRKCB,CENPJ,HDAC8,PRKCQ,ZKSCAN7,ZNF302,KMT2E,MAPK1,MAPK3,MAPK11,MAP2K6,ZNF253,RETN,PSMA1,PSMA1,PSMA2,PSMA2,PSMA3,PSMA3,PSMA4,PSMA4,PSMA5,PSMA5,PSMA6,PSMA6,PSMA7,PSMA7,PSMB1,PSMB1,PSMB2,PSMB2,PSMB3,PSMB3,PSMB4,PSMB4,PSMB5,PSMB5,PSMB6,PSMB6,PSMB7,PSMB7,SMYD2,PSMB8,PSMB8,PSMB9,PSMB9,PSMB10,PSMB10,PSMC1,PSMC1,PSMC2,PSMC2,PSMC3,PSMC3,PSMC4,PSMC4,PSMC5,PSMC5,PSMC6,PSMC6,PCBP4,PSMD1,PSMD1,PSMD2,PSMD2,PSMD3,PSMD3,PSMD4,PSMD4,TIGAR,PSMD5,PSMD5,PSMD7,PSMD7,PSMD8,PSMD8,PSMD9,PSMD9,SMURF1,SMURF1,PSMD10,PSMD10,PSMD11,PSMD11,PSMD12,PSMD12,PSMD13,PSMD13,PSME1,PSME1,ZNF248,PSME2,PSME2,PTEN,CBX8,ZNF286A,ZNF287,ZNF304,GATAD2B,ZNF490,ARID1B,RPTOR,ZNF398,ZNF624,ZNF471,ZNF492,ZFP14,GPAM,TNRC6C,TNRC6C,ZNF317,PTPN1,ZNF529,PTPN4,PTPN11,NKX3-2,BARD1,BAX,RAD1,PVALB,NLRC4,ZNF71,ZNF77,ZNF250,KMT2C,RRAGD,RABGGTA,RABGGTB,RAD9A,RAD17,RAD51,RAD51D,RARA,RARB,RARG,RB1,HIVEP3,RBBP4,RBBP5,RBBP7,RBBP8,RBL1,RBL2,RBL2,ZNF350,CCND1,RELA,RFC2,RFC3,RFC4,RFC5,RHEB,RING1,BCL6,TGIF2,RNF2,BRD2,RORA,RORC,RORC,RPA1,RPA2,RPA3,ZNF704,RPS27A,RPS27A,RRM2,RXRA,RXRB,RXRG,BDNF,BGLAP,SCO1,BID,PRDM1,ZNF667,TP53AIP1,BLK,PERP,BLM,RRAGC,MLST8,SGK1,SGK1,ANAPC1,SMURF2,SMURF2,MEAF6,BMI1,SKI,SKI,SKIL,SKIL,BMP2,BMP2,SKP1,SKP1,PINK1,SKP2,SLC2A3,ZFP69B,ZNF649,H3C13,H3C13,ZNF732,SMARCA2,SMARCA4,SMARCB1,ZNF747,SMARCC1,SMARCC2,SMARCD1,SMARCD2,SMARCD3,SMARCE1,SOD2,BNIP3L,SOX2,SOX9,SP1,SPI1,SPP1,SRC,SRC,BRCA1,SREBF1,SSRP1,SST,STAT1,AURKA,STK11,SUPT4H1,SUPT5H,SURF1,TAF1,TAF2,TAF4,TAF4B,TAF5,TAF6,TAF7,TAF9,TAF10,TAF11,TAF12,TAF13,TAL1,TBL1X,TBL1X,TBP,TBX5,TCEA1,ELOC,ELOB,ELOA,TCF3,TCF7,TCF7L2,TCF12,BTG1,ZNF354A,PRDX2,TEAD1,TEAD4,TEAD3,TFAP2A,TFAP2A,TFAP2B,TFAP2B,TFAP2C,TFAP2C,NR2F1,TFDP1,TFDP2,TGFA,TGFB1,TGIF1,THBS1,THRA,THRB,TJP1,NR2E1,CLDN5,TOP3A,TP53,TP53BP2,TP73,NR2C1,NR2C2,TRPC3,H2AC19,H2AC19,TSC1,TSC2,ZNF736,ELOA3B,ZNF705D,TWIST1,TWIST1,TXN,TXN,TXNRD1,ZNF726,ZNF735,UBA52,UBA52,UBB,UBB,UBC,UBC,UBE2D1,UBE2D3,UBE2E1,UBE2I,UBE2I,SUMO1,SUMO1,NR1H2,VDR,VEGFA,NELFA,WRN,YES1,YY1,YWHAB,YWHAE,YWHAG,YWHAH,YWHAZ,ZFP37,ZNF2,ZNF3,ZNF711,ZNF10,ZNF12,ZNF14,ZNF708,ZNF17,ZNF18,ZNF19,ZNF20,ZNF23,ZNF26,ZNF28,ZNF33A,ZNF33B,ZKSCAN1,ZNF37A,ZNF41,ZNF43,ZNF45,ZNF70,ZNF74,ZNF75D,ZNF75A,ZNF79,ZNF221,ZNF99,ZNF222,ZNF124,ZNF133,ZNF135,ZNF136,ZNF138,ZNF140,ZNF141,PCGF2,ZNF154,ZNF155,ZNF157,ZNF175,ZNF180,ZNF184,ZNF189,ZKSCAN8,ZNF195,ZNF200,ZNF202,ZNF205,ZNF208,ZNF213,ZNF214,ZNF215,ZNF223,ZNF224,ZNF225,ZNF226,ZNF227,ZNF112,ZNF230,BTG2,BRPF1,USP7,ZNF655,KCTD15,ZNF426,MAPKAP1,DEK,ZNF343,ZNF557,NELFE,BCL2L14,TBL1XR1,TBL1XR1,E2F8,ZNF419,ZNF750,ZNF668,ZNF665,SEM1,SEM1,EHMT1,ZNF552,PIP4K2C,ZNF669,ZNF212,HDAC11,ZNF671,ZNF613,KAT6A,ZNF696,ZNF442,RMI1,PHC3,ZNF556,ZNF606,CALM1,ZFP2,ZNF614,NR4A3,ZNF703,RNF34,ZNF430,EPC1,ZKSCAN3,CALM2,ZNF34,CALM3,ZNF436,KMT2D,YEATS4,CAMK4,TAF15,CAMK2A,CAMK2B,CAMK2D,ELL,CAMK2G,ZNF611,MED25,USP9X,ARID1A,H4C9,H4C9,AXIN1,CDC7,H2AC14,H2AC14,H2AC6,H2AC6,H2AC4,H2AC4,H2AC18,H2AC18,H2AC20,H2AC20,H2BC8,H2BC8,CASP1,H2BC13,H2BC13,H2BC15,H2BC15,H2BC14,H2BC14,H2BC7,H2BC7,TCF7L1,H2BC6,H2BC6,H2BC9,H2BC9,H2BC10,H2BC10,H2BC4,H2BC4,H2BC17,H2BC17,H2BC21,H2BC21,CASP2,H3C1,H3C1,H3C4,H3C4,H3C3,H3C3,H3C6,H3C6,H3C11,H3C11,H3C8,H3C8,H3C12,H3C12,H3C10,H3C10,H3C2,H3C2,H4C1,H4C1,H4C4,H4C4,H4C6,H4C6,H4C12,H4C12,H4C11,H4C11,H4C3,H4C3,H4C8,H4C8,H4C2,H4C2,SESN2,H4C5,H4C5,H4C13,H4C13,RHNO1,H4C14,H4C14,ITCH,TFAP2D,TFAP2D,ZNF484,L3MBTL2,TAF3,CASP6,HDAC10,PIP4K2B,BRIP1,UXT,PCGF6,ZNF394,ATRIP,MED10,ZNF282,ING5,CASP10,NR0B2,PCGF5,ZNF528,MAML2,MAML2,ZNF333,DYRK2,ZNF559,CUL1,CUL1,KRBA1,TEAD2,DPY30,ZNF347,TRIM63,CAT,CBX2,ZNF577,ZNF607,ZNF496,RSPO3,ZNF514,AIFM2,ZNF382,ZNF587,ZNF566,PPM1D,COX14,H2BC12,H2BC12,CBX4,TNKS1BP1,MAPKAPK5,CAV1,ACTL6A,RUNX2,RUNX2,RUNX1,RUNX1,CDK13,TP63,RUNX3,RUNX3,LAMTOR3,CBFB,CBFB,CDC23,EED,CRADD,TNFRSF18,TNFRSF10D,TNFRSF10C,TNFRSF10B,TNFRSF10A,CCNK,HDAC3,HDAC3,KAT2B,KAT2B,CDK5R1,NR1I2,LDB1,CDC16,CCNA2,CCNA1,CCNB1,CCNC,CCND2,CCND3,H3C7,H3C7,H2BC11,H2BC11,CCNE1,CCNG1,ZNF30,CCNG2,CCNH,SOCS3,ZNF551,ZNF616,ZNF468,ZNF160,MED30,CCNT1,CCNT2,ZNF799,ZNF625,ZNF700,ZNF439,ZNF486,ASH2L,PIP4P1,ZNF479,ZNF697,USP2,ZNF682,CNOT9,CCNE2,CTDP1,EXO1,PLXNA4,COX7A2L,TTC5,ZNF300,AURKB,AURKB,MTA2,ZNF461,ZNF585B,ZNF764,MED14,ZNF235,ZNF561,KLF4,CNOT8,ZNF670,ITGBL1,CGB5,COX5A,ZNF101,CGB8,ZNF264,H2AZ2,H2AZ2,TP53INP1,MED23,MED17,MED26,MED27,MED7,HAND2,MED20,PSMF1,PSMF1,ZNF254,TP53I3,NR1D1,NCOR1,NCOR1,NCOR2,NCOR2,MDC1,ZNF432,HDAC9,SETD1A,KMT2B,HDAC4,HDAC4,MAML1,MAML1,CDK1,PSMD6,PSMD6,MED24,NUAK1,CDC25C,CDC27,MED12,MED13,NR1I3,NR1H4,NR1D2,RBX1,RBX1,SCO2",Generic Transcription Pathway,1434

R-HSA-212676,"UNC13B,CPLX1,RIMS1,APBA1,LIN7C,RAB3A,LIN7B,SLC18A2,SNAP25,STX1A,STXBP1,VAMP2,SYN1,SYN2,SYT1,SYN3,PPFIA4,PPFIA2,PPFIA1,PPFIA3,CASK,LIN7A,TSPOAP1",Dopamine Neurotransmitter Release Cycle,23

R-HSA-212718,"HBEGF,EGF,EGFR,EREG,EPGN,AREG,PLCG1,BTC,TGFA",EGFR interacts with phospholipase C-gamma,9

R-HSA-2129379,"FBLN5,FBLN5,EMILIN1,EMILIN1,ELN,ELN,FBLN1,FBLN1,FBLN2,FBLN2,FBN1,FBN1,FBN2,FBN2,EFEMP1,EFEMP1,FN1,BMP10,EFEMP2,EFEMP2,ITGAV,ITGB1,ITGB3,ITGB5,ITGB6,ITGB8,LTBP1,LTBP2,LTBP3,MFAP1,MFAP2,MFAP2,MFAP3,MFAP4,BMP2,BMP4,BMP7,TGFB1,TGFB2,TGFB3,VTN,MFAP5,MFAP5,GDF5,EMILIN2,EMILIN2,LTBP4,FBN3,FBN3,ITGA8,EMILIN3,EMILIN3",Molecules associated with elastic fibres,52

R-HSA-2132295,"AP1M2,KIF20A,ACTR1B,ACTR1A,TUBA1B,TUBB3,TUBB4A,TUBB4B,SEC24B,IFI30,SEC23A,DCTN2,CENPE,DCTN6,CTSC,SEC24A,KIF2C,KIF3A,DCTN3,TUBA3E,TUBA3D,OSBPL1A,AP2M1,AP1S1,AP2S1,CLTA,CLTC,AP1S3,DYNLL2,KLC3,CTSB,CTSD,CTSE,CTSH,CTSK,CTSL,CTSV,CTSO,CTSS,AP2A1,AP2A2,AP1B1,AP2B1,DCTN1,AP1G1,DNM1,DYNC1H1,DYNC1I1,DYNC1I2,DYNC1LI2,DNM2,SEC31A,KIFAP3,KIF4A,DNM3,KIF26A,KIF4B,RACGAP1,HLA-DMB,HLA-DOA,HLA-DOB,HLA-DPA1,HLA-DPB1,HLA-DQA1,HLA-DQA2,HLA-DQB1,HLA-DQB2,HLA-DRA,HLA-DRB1,HLA-DRB3,HLA-DRB4,HLA-DRB5,TUBB8,TUBB2B,ARF1,KIF2A,KIF3C,KIF5A,KIF5B,KLC1,KIF11,KIF22,LAG3,SAR1B,DYNC1LI1,DCTN4,TUBA8,CTSA,ACTR10,LGMN,KIF15,SEC13,SH3GL2,KLC2,SPTBN2,TUBA4A,TUBA3C,TUBB2A,TUBA1A,RAB7A,TUBAL3,TUBB1,KIF18A,CANX,CAPZA1,CAPZA2,CAPZB,RILP,DCTN5,TUBB6,KIF2B,TUBA1C,DYNLL1,CTSF,AP1S2,AP1M1,KLC4,CAPZA3,KIF3B,KIF23,SEC24C,CD74,SEC24D",MHC class II antigen presentation,123

R-HSA-2142670,"CYP1A1,CYP1A2,CYP1B1,CYP2C19,CYP2C8,CYP2C9,CYP2J2,EPHX2",Synthesis of epoxy (EET) and dihydroxyeicosatrienoic acids (DHET),8

R-HSA-2142688,"ALOX5,ALOX5AP,GPX1,GPX2,GPX4,LTC4S,PON1,PON2,PON3",Synthesis of 5-eicosatetraenoic acids,9

R-HSA-2142691,"CYP4F8,CYP4F22,CYP4A11,CYP4B1,DPEP1,PTGR1,ALOX5,ALOX5AP,ALOX15,GGT1,GGT5,CYP4A22,LTA4H,CYP4F3,LTC4S,ABCC1,CYP4F11,DPEP2,DPEP3,CYP4F2,MAPKAPK2",Synthesis of Leukotrienes (LT) and Eoxins (EX),21

R-HSA-2142696,ALOX12,Synthesis of Hepoxilins (HX) and Trioxilins (TrX),1

R-HSA-2142700,"PTGR1,ALOX12,ALOX5,ALOX5AP,HPGD,LTC4S",Synthesis of Lipoxins (LX),6

R-HSA-2142712,"ALOX12,ALOX12B,ALOX15,GPX1,GPX2,GPX4,ALOXE3",Synthesis of 12-eicosatetraenoic acid derivatives,7

R-HSA-2142753,"PTGES3,CYP4F8,CYP2U1,CYP4F22,PRXL2B,PTGR2,CYP1A1,CYP1A2,CYP1B1,CYP2C19,CYP2C8,CYP2C9,CYP2J2,CYP4A11,CYP4B1,CYP8B1,FAAH2,AWAT1,DPEP1,EPHX2,FAAH,PTGR1,ALOX12,ALOX5,ALOX5AP,ALOX12B,ALOX15,ALOX15B,GGT1,GGT5,HPGDS,CYP4A22,GPX1,GPX1,GPX2,GPX2,GPX4,GPX4,HPGD,LTA4H,CYP4F3,LTC4S,ABCC1,PLA2G4A,PON1,PON2,PON3,PTGDS,PTGIS,PTGS1,PTGS2,CYP4F11,ALOXE3,DPEP2,DPEP3,TBXAS1,PTGES2,CYP4F2,AKR1C3,CBR1,MAPKAPK2,PTGES",Arachidonic acid metabolism,62

R-HSA-2142770,"ALOX15,ALOX15B,GPX1,GPX2,GPX4,PTGS2",Synthesis of 15-eicosatetraenoic acid derivatives,6

R-HSA-2142789,"COQ7,PDSS1,COQ6,COQ3,COQ9,PDSS2,COQ5",Ubiquinol biosynthesis,7

R-HSA-2142816,"CYP2U1,CYP1A1,CYP1A2,CYP1B1,CYP2C19,CYP2C8,CYP2C9,CYP4A11,CYP4F2",Synthesis of (16-20)-hydroxyeicosatetraenoic acids (HETE),9

R-HSA-2142845,"ABCC5,LYVE1,CHP1,GUSB,HAS1,HAS2,HAS3,HEXA,HEXB,HMMR,HYAL1,STAB2,CEMIP,SLC9A1,HYAL3,HYAL2,CD44",Hyaluronan metabolism,17

R-HSA-2142850,"ABCC5,HAS1,HAS2,HAS3,CEMIP",Hyaluronan biosynthesis and export,5

R-HSA-2151201,"CARM1,NCOA2,PPARGC1A,POLG2,PPARGC1B,PPARGC1B,CREB1,CREB1,ATF2,CREBBP,CRTC2,ESRRA,ESRRA,ALAS1,NCOA6,PPRC1,CRTC1,SIRT5,SIRT4,SIRT3,GABPA,GABPB1,GLUD1,GLUD2,HCFC1,IDH2,MEF2C,MEF2D,NRF1,NRF1,ATP5F1B,TFB1M,CYCS,POLRMT,PPARA,MED1,ACSS2,TWNK,RXRA,TFB2M,CRTC3,SMARCD3,SOD2,SSBP1,TBL1X,TFAM,TFAM,TBL1XR1,MTERF1,CALM1,CHD9,CALM2,CALM3,CAMK4,PERM1,HELZ2,NCOA1,HDAC3,NR1D1,NCOR1,TGS1",Transcriptional activation of mitochondrial biogenesis,61

R-HSA-2151209,"PPARGC1A,MAPK14,PRKAG2,PRKAG3,PRKAA2,PRKAB1,PRKAB2,PRKAG1,MAPK11,MAPK12",Activation of PPARGC1A (PGC-1alpha) by phosphorylation,10

R-HSA-2160456,PAH,Phenylketonuria,1

R-HSA-216083,"COL1A1,COL1A1,COL1A2,COL1A2,COL2A1,COL2A1,COL3A1,COL4A1,COL4A1,COL4A2,COL4A2,COL4A3,COL4A3,COL4A4,COL4A4,COL4A5,COL4A5,COL4A6,COL4A6,COL5A1,COL5A2,COL6A1,COL6A2,COL6A3,COL7A1,COL8A1,COL8A2,COL9A1,COL9A2,COL9A3,COL10A1,COL13A1,COL16A1,COMP,COL6A6,FBN1,FGA,FGB,FGG,ITGA11,ITGA11,FN1,COL6A5,HSPG2,TNC,IBSP,ICAM1,ICAM2,ICAM3,ICAM4,ITGA6,ITGA6,ITGA1,ITGA1,ITGA2,ITGA2,ITGA2B,ITGA3,ITGA3,ITGA4,ITGA4,ITGA5,ITGA5,ITGA7,ITGA9,ITGA9,ITGAD,ITGAE,ITGAL,ITGAM,ITGAV,ITGAV,ITGAX,ITGB1,ITGB1,ITGB2,ITGB3,ITGB3,ITGB5,ITGB5,ITGB6,ITGB7,ITGB8,AGRN,KDR,LUM,COL5A3,F11R,PECAM1,JAM2,SPP1,SPP1,BSG,THBS1,ICAM5,VCAM1,VTN,VWF,COL18A1,MADCAM1,JAM3,ITGA10,ITGA10,ITGA8,ITGA8,COL23A1,CD44,CD47,CDH1",Integrin cell surface interactions,109

R-HSA-2160916,"LYVE1,CHP1,GUSB,HEXA,HEXB,HMMR,HYAL1,STAB2,SLC9A1,HYAL3,HYAL2,CD44",Hyaluronan uptake and degradation,12

R-HSA-2161517,"ABCB1,SLC22A1,SLC22A3,SLC22A2,ABCG2",Abacavir transmembrane transport,5

R-HSA-2161522,"ADH1A,ADAL,NT5C2,GUK1,PCK1,ABCB1,SLC22A1,SLC22A3,SLC22A2,ABCG2",Abacavir transport and metabolism,10

R-HSA-2161541,"ADH1A,ADAL,NT5C2,GUK1,PCK1",Abacavir metabolism,5

R-HSA-2162123,"PTGES3,PRXL2B,PTGR2,CYP8B1,HPGDS,HPGD,PTGDS,PTGIS,PTGS1,PTGS2,TBXAS1,PTGES2,AKR1C3,CBR1,PTGES",Synthesis of Prostaglandins (PG) and Thromboxanes (TX),15

R-HSA-2168880,"ALB,AMBP,HBA1,HBA2,HBB,HP,HPR,HPX,APOA1,JCHAIN,LRP1,APOL1,CD163",Scavenging of heme from plasma,13

R-HSA-2172127,"SIGLEC14,KIR2DS2,KLRC4-KLRK1,KLRC4-KLRK1,SIRPB1,VAV3,CD300LB,KLRK1,KLRK1,CLEC5A,FYN,FYN,LAT,SIGLEC15,GRB2,HLA-B,HLA-C,HLA-E,HLA-E,HRAS,CD300E,KIR2DS1,KIR2DS4,KIR2DS5,KIR3DS1,KLRC2,KLRC2,KLRD1,KLRD1,KRAS,LCK,LCK,LCP2,NRAS,PIK3CA,PIK3CB,PIK3R1,PIK3R2,PLCG1,PLCG2,TREM2,TREM2,TREM1,B2M,B2M,RAC1,SHC1,SOS1,SYK,BTK,TYROBP,TYROBP,VAV2,GRAP2,NCR2",DAP12 interactions,55

R-HSA-217271,"FMO1,FMO2,FMO3",FMO oxidises nucleophiles,3

R-HSA-2173782,"HYOU1,HYOU1,HSPH1,HSPH1,SCGB3A2,SCGB3A2,COL1A1,COL1A2,COL3A1,COL4A1,COL4A2,ALB,STAB1,FTH1,FTL,AMBP,SSC5D,SCARA5,HBA1,HBA2,HBB,HP,HPR,HPX,HSP90AA1,HSP90AA1,APOA1,APOA1,APOB,APOB,APOE,APOE,JCHAIN,LRP1,MSR1,MSR1,STAB2,MASP1,SAA1,SPARC,HSP90B1,HSP90B1,COLEC11,COLEC12,CALR,CALR,APOL1,SCARF1,SCARF1,MARCO,MARCO,CD163,CD36,CD36,SCARB1",Binding and Uptake of Ligands by Scavenger Receptors,55

R-HSA-2173788,"STUB1,STRAP,STRAP,NEDD4L,PPP1R15A,BAMBI,SMAD2,SMAD3,SMAD7,SMAD7,UCHL5,PPP1CA,PPP1CB,PPP1CC,PMEPA1,SMURF1,RPS27A,RPS27A,SMURF2,SMURF2,TGFB1,TGFB1,TGFBR1,TGFBR1,TGFBR2,TGFBR2,UBA52,UBA52,UBB,UBB,UBC,UBC,XPO1,MTMR4,ZFYVE9,USP15",Downregulation of TGF-beta receptor signaling,36

R-HSA-2173789,"STUB1,STRAP,STRAP,FKBP1A,NEDD4L,PPP1R15A,BAMBI,SMAD2,SMAD3,SMAD4,SMAD7,SMAD7,NEDD8,FURIN,UCHL5,PPP1CA,PPP1CB,PPP1CC,PMEPA1,SMURF1,RPS27A,RPS27A,SMURF2,SMURF2,TGFB1,TGFB1,TGFBR1,TGFBR1,TGFBR2,TGFBR2,UBA52,UBA52,UBB,UBB,UBC,UBC,XPO1,CBL,UBE2M,MTMR4,ZFYVE9,ZFYVE9,USP15",TGF-beta receptor signaling activates SMADs,43

R-HSA-2173791,"FKBP1A,ARHGEF18,ARHGEF18,RHOA,RHOA,F11R,F11R,PARD6A,PARD6A,PRKCZ,PRKCZ,PARD3,PARD3,SMURF1,CGN,CGN,RPS27A,TGFB1,TGFB1,TGFBR1,TGFBR1,TGFBR2,TGFBR2,UBA52,UBB,UBC",TGF-beta receptor signaling in EMT (epithelial to mesenchymal transition),26

R-HSA-2173793,"CDK8,CDK9,CDKN2B,PARP1,E2F4,E2F5,SNW1,NEDD4L,ATP1B4,WWTR1,HDAC1,JUNB,SMAD2,SMAD2,SMAD3,SMAD3,SMAD4,SMAD4,SMAD7,SMAD7,MEN1,MYC,SERPINE1,TRIM33,RNF111,RNF111,PPM1A,RBL1,TGIF2,RPS27A,RPS27A,SMURF2,SMURF2,SKI,SKI,SKIL,SKIL,SP1,TFDP1,TFDP2,TGIF1,UBA52,UBA52,UBB,UBB,UBC,UBC,UBE2D1,UBE2D3,USP9X,CCNK,CCNC,CCNT1,CCNT2,NCOR1,NCOR1,NCOR2,NCOR2",Transcriptional activity of SMAD2/SMAD3:SMAD4 heterotrimer,58

R-HSA-2173795,"PARP1,NEDD4L,HDAC1,SMAD2,SMAD2,SMAD3,SMAD3,SMAD4,SMAD4,TRIM33,RNF111,PPM1A,TGIF2,RPS27A,RPS27A,SMURF2,SMURF2,SKI,SKI,SKIL,SKIL,TGIF1,UBA52,UBA52,UBB,UBB,UBC,UBC,UBE2D1,UBE2D3,USP9X,NCOR1,NCOR1,NCOR2,NCOR2",Downregulation of SMAD2/3:SMAD4 transcriptional activity,35

R-HSA-2173796,"CDK8,CDK9,CDKN2B,E2F4,E2F5,SNW1,ATP1B4,WWTR1,HDAC1,JUNB,SMAD2,SMAD3,SMAD4,SMAD7,SMAD7,MEN1,MYC,SERPINE1,RNF111,RNF111,RBL1,TGIF2,RPS27A,RPS27A,SP1,TFDP1,TFDP2,TGIF1,UBA52,UBA52,UBB,UBB,UBC,UBC,CCNK,CCNC,CCNT1,CCNT2",SMAD2/SMAD3:SMAD4 heterotrimer regulates transcription,38

R-HSA-2179392,"HBEGF,HBEGF,EGFR,GRB2,HRAS,KRAS,MMP3,MMP3,NRAS,PRKCA,SOS1",EGFR Transactivation by Gastrin,11

R-HSA-2187335,"OPN1MW3,AWAT2,OPN1MW,RBP3,RLBP1,RLBP1,OPN1SW,OPN1MW2,DHRS3",The retinoid cycle in cones (daylight vision),9

R-HSA-2187338,"CHURC1-FNTB,GPC6,OPN1MW3,DHRS9,GNB5,GNB5,METAP2,AKR1C4,CLPS,SDR9C7,CNGB1,CNGA1,GRK7,RDH12,PLB1,RDH10,AWAT2,AKR1C1,GPC2,NAPEPLD,GPC4,GPC5,METAP1,FNTA,FNTB,ABCA4,ABCA4,LRP10,OPN1MW,GPC3,GNAT1,GNAT1,GNB1,GNB1,GNGT1,GNGT1,GPC1,CYP4V2,GRK4,GUCA1A,GUCA1B,GUCY2F,LRP12,GUCY2D,HSD17B1,HSPG2,APOA1,APOA1,APOA2,APOA2,APOB,APOB,GPIHBP1,APOC2,APOC2,APOC3,APOC3,APOE,APOE,AGRN,RGS9BP,RGS9BP,LDLR,LPL,LRP1,LRP2,MYO7A,NMT1,RDH8,RDH11,PDE6A,PDE6A,PDE6G,PDE6G,PDE6B,PDE6B,BCO1,PNLIP,PPEF1,RETSAT,PRKCA,PRKCQ,APOM,AKR1B10,RBP1,RBP2,RBP3,RBP4,RCVRN,RCVRN,RDH5,RHO,RHO,GRK1,GRK1,RLBP1,RLBP1,OPN1SW,RPE65,SAG,SAG,SDC1,SDC2,SDC4,STRA6,TTR,OPN1MW2,LRP8,CAMKMT,CALM1,CALM1,CALM2,CALM2,CALM3,CALM3,BCO2,RDH16,HSD17B6,AKR1C3,RGS9,RGS9,SLC24A1,LRAT,DHRS3,NMT2,GUCA1C,SDC3",Visual phototransduction,127

R-HSA-2197563,"MAMLD1,MAMLD1,CREB1,EP300,FCER2,GZMB,HES1,RBPJ,RBPJ,HES5,NOTCH2,NOTCH2,MAML3,MAML3,MAML2,MAML2,MAML1,MAML1",NOTCH2 intracellular domain regulates transcription,18

R-HSA-2206280,HYAL1,MPS IX - Natowicz syndrome,1

R-HSA-2206281,"HGSNAT,GALNS,GLB1,GNS,GUSB,HYAL1,IDS,IDUA,ARSB,NAGLU,SGSH",Mucopolysaccharidoses,11

R-HSA-2206282,NAGLU,MPS IIIB - Sanfilippo syndrome B,1

R-HSA-2206285,ARSB,MPS VI - Maroteaux-Lamy syndrome,1

R-HSA-2206290,GALNS,MPS IV - Morquio syndrome A,1

R-HSA-2206291,HGSNAT,MPS IIIC - Sanfilippo syndrome C,1

R-HSA-2206292,GUSB,MPS VII - Sly syndrome,1

R-HSA-2206296,IDS,MPS II - Hunter syndrome,1

R-HSA-2206302,IDUA,MPS I - Hurler syndrome,1

R-HSA-2206305,GNS,MPS IIID - Sanfilippo syndrome D,1

R-HSA-2206307,SGSH,MPS IIIA - Sanfilippo syndrome A,1

R-HSA-2206308,GLB1,MPS IV - Morquio syndrome B,1

R-HSA-2214320,"COL1A1,COL1A2,COL4A1,COL4A2,COL4A3,COL4A4,COL4A5,COL4A6,COL7A1,LAMA3,LAMB3,LAMC2,BMP1,TLL1,TLL2",Anchoring fibril formation,15

R-HSA-2219528,"AKT3,CDKN1A,CDKN1B,NRG3,FRS2,CHUK,PIK3AP1,CREB1,NRG4,KLB,HBEGF,EGF,EGFR,ERBB2,ERBB3,ERBB4,EREG,AKT1,AKT2,ESR1,ESR2,FGF1,FGF2,FGF3,FGF4,FGF5,FGF6,FGF7,FGF8,FGF9,FGF10,FGFR1,FGFR3,FGFR2,FGFR4,FOXO1,FOXO3,MTOR,RICTOR,FYN,GAB1,EPGN,FGF20,FGF22,GRB2,GSK3A,GSK3B,ICOS,HGF,NRG1,NR4A1,IRS1,AREG,KIT,RHOG,LCK,MDM2,MET,KITLG,FOXO4,TRAT1,PDGFA,PDGFB,PDGFRA,PDGFRB,PDPK1,PIK3CA,PIK3CB,PIK3CD,PIK3R1,PIK3R2,PRR5,BAD,PTEN,PTPN11,RAC1,RAC2,RPS6KB2,MLST8,SRC,STRN,BTC,TGFA,TSC2,VAV1,MAPKAP1,FGF23,CASP9,AKT1S1,PIK3R3,IRS2,FGF18,FGF17,FGF16,CD19,KL,CD28,CD80,CD86,NRG2,FGF19",PI3K/AKT Signaling in Cancer,101

R-HSA-2219530,"NRG3,FRS2,PIK3AP1,NRG4,KLB,HBEGF,EGF,EGFR,ERBB2,ERBB3,ERBB4,EREG,ESR1,ESR2,FGF1,FGF2,FGF3,FGF4,FGF5,FGF6,FGF7,FGF8,FGF9,FGF10,FGFR1,FGFR3,FGFR2,FGFR4,FYN,GAB1,EPGN,FGF20,FGF22,GRB2,ICOS,HGF,NRG1,IRS1,AREG,KIT,RHOG,LCK,MET,KITLG,TRAT1,PDGFA,PDGFB,PDGFRA,PDGFRB,PIK3CA,PIK3CB,PIK3CD,PIK3R1,PIK3R2,PTPN11,RAC1,RAC2,SRC,STRN,BTC,TGFA,VAV1,FGF23,PIK3R3,IRS2,FGF18,FGF17,FGF16,CD19,KL,CD28,CD80,CD86,NRG2,FGF19",Constitutive Signaling by Aberrant PI3K in Cancer,75

R-HSA-2243919,"COL1A1,COL1A2,COL4A1,COL4A2,COL4A3,COL4A4,COL4A5,COL4A6,LOX,LOXL1,LOXL2,PCOLCE,BMP1,TLL1,TLL2,PXDN,LOXL4,LOXL3",Crosslinking of collagen fibrils,18

R-HSA-2262752,"HDAC6,DNAJB6,COMMD3-BMI1,RAD50,ACTR1A,CDK2,CDK4,PSME3,CDK6,PSMD14,CDKN1A,CDKN1B,LOC102724334,CDKN2A,DNAJA2,CDKN2B,CDKN2C,TCIRG1,CDKN2D,RRAGB,CITED2,TUBA1B,TUBB3,TUBB4A,TUBB4B,ANAPC10,CEBPB,CEBPB,KAT5,CEBPG,DCTN2,LAMTOR5,TXNRD2,NPRL2,RRAGA,DCTN6,PTGES3,NUP50,HSPH1,EHMT2,PRDX3,STIP1,GCN1,UBE2C,NUP42,KPTN,RPL35,EGLN2,EGLN2,EGLN3,EGLN3,DCTN3,TUBA3E,TUBA3D,RPL39L,HSPA12B,ANAPC16,H4-16,PSMB11,WTIP,WTIP,H3C14,ATP6V1G3,H2BU1,NUP35,ATF2,ATF2,CREBBP,RPS4Y2,DYNLL2,RPL10L,CRYAB,MAPK14,PSMA8,C12orf66,SLC38A9,CYBA,CYBB,BMT2,ATP6V0E2,DCTN1,DDIT3,DYNC1H1,DYNC1I1,DYNC1I2,DYNC1LI2,E2F1,E2F2,E2F3,PHC1,PHC2,EEF1A1,AGO3,AGO4,EIF2S1,EIF2S3,RPL22L1,FLCN,EP300,EPAS1,EPAS1,EPO,ERF,ERF,ETS1,ETS2,EZH2,ATF5,HSPA4L,FKBP4,FKBP5,SCMH1,TNIK,TNRC6B,KDM6B,NUP205,PSME4,NUP210,NUP160,SZT2,SIRT1,CBX6,NUP188,SUZ12,RPL13A,CABIN1,FOS,NUP62,PPP1R15A,SH3BP4,ATP6V0D2,ATP6V1C2,CDC26,MTOR,H2BC1,GPX6,PRDX5,TXN2,ASF1A,RPL36,ANAPC15,POT1,HSPA12A,HIGD1A,TINF2,HSPB8,AGO1,DNAJC2,EIF2AK1,SESN1,VENTX,TNRC6A,UBE2S,GPX1,GPX2,GPX3,GPX5,GPX7,GRB10,LAMTOR2,NR3C1,GSK3B,GSR,GSTP1,UBN1,ANAPC2,ANAPC4,ERO1A,H1-0,H1-2,H1-3,H1-4,H1-5,H2AC8,H2AC7,H2AX,H2AZ1,H2BC5,H2BC3,H3-3A,H3-3B,H1-1,HIF1A,HMGA1,NUDT2,HSBP1,HSF1,DNAJA1,HSPA1A,HSPA1B,HSPA1L,HSPA2,HSPA4,HSPA5,HSPA6,HSPA8,HSPA9,HSP90AA1,HSP90AB1,DNAJB1,H3C15,ID1,AQP8,IFNB1,TUBB8,TUBB2B,NUP43,IGFBP7,IL1A,IL6,CXCL8,AR,JUN,LAMTOR4,RPSA,LMNB1,ARNT,MDM2,MDM4,MAP3K5,MAP3K5,NR3C2,MOV10,MRE11,ASNS,EIF2AK4,ATF3,ATF3,ATF4,ATF4,NBN,NCF2,NCF4,ATM,RPL10A,H2AB1,ATOX1,NFKB1,NUP88,NUP98,GPX8,P4HB,MINK1,NOX4,PRDX1,RPS27L,RPL26L1,DYNC1LI1,DCTN4,HSPA14,FZR1,ATP6V1D,ANAPC5,ANAPC7,HIKESHI,ANAPC11,ATP6V1H,TUBA8,ATP6V1A,PGR,ATP6V1B1,ATP6V1B2,ATP6V0C,ATP6V1C1,ATP6V1E1,ATP6V0B,NUP54,ATP6V1G2,ATP7A,CYCS,TERF2IP,MIOS,ATR,EGLN1,EGLN1,LAMTOR1,IMPACT,H4C15,DNAJA4,HIF1AN,NDC1,NUP133,H2AJ,ITFG2,ACTR10,MAPK1,MAPK1,MAPK3,MAPK3,MAPK7,MAPK8,MAPK11,MAPK9,MAPK10,MAP2K3,MAP2K6,MAP2K7,PSMA1,PSMA2,PSMA3,PSMA4,PSMA5,PSMA6,PSMA7,PSMB1,PSMB2,PSMB3,PSMB4,PSMB5,PSMB6,PSMB7,PSMB8,PSMB9,PSMB10,PSMC1,PSMC2,PSMC3,PSMC4,PSMC5,PSMC6,PSMD1,PSMD2,PSMD3,PSMD4,PSMD5,NUP107,PSMD7,PSMD8,PSMD9,PSMD10,PSMD11,PSMD12,PSMD13,PSME1,PSME2,BAG1,CBX8,RPTOR,FNIP2,EP400,TNRC6C,TRIB3,CCAR2,RRAGD,RANBP2,RB1,RBBP4,RBBP7,RELA,RHEB,RING1,RNF2,RPA1,RPA2,RPA3,RPL3,RPL3L,RPL4,RPL5,RPL6,RPL7,RPL7A,RPL8,RPL9,RPL10,RPL11,RPL12,RPL13,RPL15,RPL17,RPL18,RPL18A,RPL19,RPL21,RPL22,RPL23A,RPL24,RPL26,RPL27,RPL30,RPL27A,RPL28,RPL29,RPL31,RPL32,RPL34,RPL35A,RPL36AL,RPL37,RPL37A,RPL38,RPL39,RPL41,RPL36A,RPLP0,RPLP1,RPLP2,RPS2,RPS3,RPS3A,RPS4X,RPS4Y1,RPS5,RPS6,RPS6KA1,RPS6KA1,RPS6KA2,RPS6KA2,RPS6KA3,RPS6KA3,RPS7,RPS8,RPS9,RPS10,RPS11,RPS12,RPS13,RPS14,RPS15,RPS15A,RPS16,RPS17,RPS18,RPS19,RPS20,RPS21,RPS23,RPS24,RPS25,RPS26,RPS27,RPS27A,RPS27A,RPS28,RPS29,SEC13,RRAGC,MAP2K4,MAP2K4,MLST8,HIF3A,ANAPC1,BMI1,ACD,CASTOR1,NCF1,H3C13,SOD1,SOD1,SOD2,SOD3,SOD3,SP1,ST13,STAT3,HSPA13,ELOC,ELOC,ELOB,ELOB,PRDX2,TERF1,TERF2,TFDP1,TFDP2,TP53,TPR,H2AC19,DNAJC7,TUBA4A,TUBA3C,TUBB2A,HIRA,CASTOR2,TXN,TXNRD1,UBA52,UBA52,UBB,UBC,UBE2D1,UBE2D2,UBE2D3,UBE2E1,VCP,VEGFA,VHL,VHL,YWHAE,CA9,TUBA1A,MAPKAPK3,NUP37,CHAC1,NOX5,WDR59,SEM1,EHMT1,TUBAL3,NUP85,PHC3,NUP214,AAAS,HMGA2,TUBB1,NPRL3,CAMK2A,CAMK2B,CAMK2D,CAMK2G,SEH1L,CAPZA1,H3-4,H4C9,CAPZA2,CAPZB,H2AC14,H2AC6,H2AC4,H2AC18,H2AC20,H2BC8,H2BC13,H2BC15,H2BC14,H2BC7,H2BC6,H2BC9,H2BC10,H2BC4,H2BC17,H2BC21,H3C1,H3C4,H3C3,H3C6,H3C11,H3C8,H3C12,H3C10,H3C2,H4C1,H4C4,H4C6,H4C12,H4C11,H4C3,H4C8,H4C2,SESN2,H4C5,H4C13,H4C14,WDR24,AKT1S1,DCTN5,CUL2,CUL2,TUBB6,CAT,CBX2,TUBA1C,RAE1,AJUBA,AJUBA,H2BC12,CBX4,MAPKAPK5,LAMTOR3,DYNLL1,CDC23,EED,CDC16,EIF2S2,CCNA2,CCNA1,H3C7,H2BC11,CCNE1,ATP6V0E1,LIMD1,LIMD1,ATP6V1E2,RPL14,ATP6V0D1,CCNE2,RPS19BP1,MAPKAPK2,ATP6V1F,RPL23,CAPZA3,H2AZ2,MAP4K4,PSMF1,BAG5,BAG4,BAG3,BAG2,ATP6V1G1,PRDX6,NUP155,FNIP1,DEPDC5,NUP93,NUP58,PSMD6,POM121,CDC27,NUP153,CCS,CCS,RBX1,RBX1",Cellular responses to stress,613

R-HSA-2299718,"SMC4,LOC102724334,SMC2,H4-16,H3C14,H2BU1,PHF8,NCAPD3,H2BC1,NCAPH2,H2AC8,H2AC7,H2AX,H2AZ1,H2BC5,H2BC3,H3-3A,H3-3B,H3C15,KMT5A,H2AB1,PLK1,NCAPG2,H4C15,H2AJ,RB1,SET,H3C13,H2AC19,MCPH1,H3-4,H4C9,H2AC14,H2AC6,H2AC4,H2AC18,H2AC20,H2BC8,H2BC13,H2BC15,H2BC14,H2BC7,H2BC6,H2BC9,H2BC10,H2BC4,H2BC17,H2BC21,H3C1,H3C4,H3C3,H3C6,H3C11,H3C8,H3C12,H3C10,H3C2,H4C1,H4C4,H4C6,H4C12,H4C11,H4C3,H4C8,H4C2,H4C5,H4C13,H4C14,H2BC12,CCNB1,H3C7,H2BC11,H2AZ2,CDK1",Condensation of Prophase Chromosomes,74

R-HSA-2393930,"NUDT5,NUDT16,NUDT1,NUDT9,NUDT15,ADPRM,NUDT18",Phosphate bond hydrolysis by NUDT proteins,7

R-HSA-2395516,"FDX2,FDX1,FDXR",Electron transport from NADPH to Ferredoxin,3

R-HSA-2404192,"FRS2,THEM4,KLB,AKT2,AKT2,FGF1,FGF2,FGF3,FGF4,FGF5,FGF6,FGF7,FGF8,FGF9,FGF10,FGFR1,FGFR3,FGFR2,FGFR4,FLT3,FLT3LG,GAB1,FGF20,FGF22,GRB2,GRB2,PIK3R4,HRAS,IGF1,IGF1R,IGF2,IRS1,IRS1,KRAS,NRAS,PDE3B,PDE3B,PDPK1,PIK3C3,PIK3CA,PIK3CB,PIK3R1,PIK3R2,TLR9,TRIB3,PTPN11,SHC1,SHC1,SOS1,SOS1,FGF23,IRS4,CILP,IRS2,IRS2,FGF18,FGF17,FGF16,KL,GAB2,FGF19",Signaling by Type 1 Insulin-like Growth Factor 1 Receptor (IGF1R),61

R-HSA-2408508,"CBSL,CTH,AHCY,GNMT,HNMT,MAT1A,NNMT,SCLY,CBS","Metabolism of ingested SeMet, Sec, MeSec into H2Se",9

R-HSA-2408522,"CBSL,INMT,RPL35,RPL39L,PSTK,RPS4Y2,RPL10L,CTH,DARS1,AHCY,RPL22L1,EPRS1,SEPHS2,RPL13A,RPL36,GNMT,GSR,HNMT,IARS1,KARS1,RPSA,MARS1,MAT1A,RPL10A,NNMT,RPS27L,SEPSECS,RPL26L1,LARS1,SCLY,QARS1,RARS1,EEFSEC,RPL3,RPL3L,RPL4,RPL5,RPL6,RPL7,RPL7A,RPL8,RPL9,RPL10,RPL11,RPL12,RPL13,RPL15,RPL17,RPL18,RPL18A,RPL19,RPL21,RPL22,RPL23A,RPL24,RPL26,RPL27,RPL30,RPL27A,RPL28,RPL29,RPL31,RPL32,RPL34,RPL35A,RPL36AL,RPL37,RPL37A,RPL38,RPL39,RPL41,RPL36A,RPLP0,RPLP1,RPLP2,RPS2,RPS3,RPS3A,RPS4X,RPS4Y1,RPS5,RPS6,RPS7,RPS8,RPS9,RPS10,RPS11,RPS12,RPS13,RPS14,RPS15,RPS15A,RPS16,RPS17,RPS18,RPS19,RPS20,RPS21,RPS23,RPS24,RPS25,RPS26,RPS27,RPS27A,RPS28,RPS29,SARS1,TXNRD1,TXNRD1,UBA52,SECISBP2,AIMP2,CBS,RPL14,PAPSS2,PAPSS1,AIMP1,RPL23,EEF1E1",Selenoamino acid metabolism,119

R-HSA-2408550,"GSR,TXNRD1,PAPSS2,PAPSS1",Metabolism of ingested H2SeO4 and H2SeO3 into H2Se,4

R-HSA-2408552,INMT,Methylation of MeSeH for excretion,1

R-HSA-2408557,"RPL35,RPL39L,PSTK,RPS4Y2,RPL10L,RPL22L1,SEPHS2,RPL13A,RPL36,RPSA,RPL10A,RPS27L,SEPSECS,RPL26L1,EEFSEC,RPL3,RPL3L,RPL4,RPL5,RPL6,RPL7,RPL7A,RPL8,RPL9,RPL10,RPL11,RPL12,RPL13,RPL15,RPL17,RPL18,RPL18A,RPL19,RPL21,RPL22,RPL23A,RPL24,RPL26,RPL27,RPL30,RPL27A,RPL28,RPL29,RPL31,RPL32,RPL34,RPL35A,RPL36AL,RPL37,RPL37A,RPL38,RPL39,RPL41,RPL36A,RPLP0,RPLP1,RPLP2,RPS2,RPS3,RPS3A,RPS4X,RPS4Y1,RPS5,RPS6,RPS7,RPS8,RPS9,RPS10,RPS11,RPS12,RPS13,RPS14,RPS15,RPS15A,RPS16,RPS17,RPS18,RPS19,RPS20,RPS21,RPS23,RPS24,RPS25,RPS26,RPS27,RPS27A,RPS28,RPS29,SARS1,UBA52,SECISBP2,RPL14,RPL23",Selenocysteine synthesis,93

R-HSA-2424491,"KLRC4-KLRK1,KLRC4-KLRK1,VAV3,KLRK1,KLRK1,FYN,FYN,LAT,GRB2,HLA-E,HLA-E,HRAS,KLRC2,KLRC2,KLRD1,KLRD1,KRAS,LCK,LCK,LCP2,NRAS,PIK3CA,PIK3CB,PIK3R1,PIK3R2,PLCG1,PLCG2,TREM2,TREM2,B2M,B2M,RAC1,SHC1,SOS1,SYK,BTK,TYROBP,TYROBP,VAV2,GRAP2",DAP12 signaling,40

R-HSA-2426168,"CARM1,NCOA2,PMVK,CREBBP,CYP51A1,DHCR7,FASN,FDFT1,FDPS,NCOA6,ACACA,HMGCR,HMGCS1,ACACB,IDI1,LSS,MTF1,MVD,MVK,NFYA,NFYA,NFYB,NFYB,NFYC,NFYC,PPARA,MED1,GPAM,RXRA,SC5D,SCD,SMARCD3,SP1,SQLE,SREBF1,SREBF1,SREBF2,SREBF2,TBL1X,TM7SF2,ELOVL6,TBL1XR1,CHD9,HELZ2,NCOA1,GGPS1,TGS1",Activation of gene expression by SREBF (SREBP),47

R-HSA-2428924,"FRS2,THEM4,KLB,AKT2,AKT2,FGF1,FGF2,FGF3,FGF4,FGF5,FGF6,FGF7,FGF8,FGF9,FGF10,FGFR1,FGFR3,FGFR2,FGFR4,FLT3,FLT3LG,GAB1,FGF20,FGF22,GRB2,GRB2,PIK3R4,HRAS,IGF1,IGF1R,IGF2,IRS1,IRS1,KRAS,NRAS,PDE3B,PDE3B,PDPK1,PIK3C3,PIK3CA,PIK3CB,PIK3R1,PIK3R2,TLR9,TRIB3,PTPN11,SHC1,SHC1,SOS1,SOS1,FGF23,IRS4,IRS2,IRS2,FGF18,FGF17,FGF16,KL,GAB2,FGF19",IGF1R signaling cascade,60

R-HSA-2428928,"FRS2,THEM4,KLB,AKT2,AKT2,FGF1,FGF2,FGF3,FGF4,FGF5,FGF6,FGF7,FGF8,FGF9,FGF10,FGFR1,FGFR3,FGFR2,FGFR4,FLT3,FLT3LG,GAB1,FGF20,FGF22,GRB2,GRB2,PIK3R4,HRAS,IGF1,IGF1R,IGF2,IRS1,IRS1,KRAS,NRAS,PDE3B,PDE3B,PDPK1,PIK3C3,PIK3CA,PIK3CB,PIK3R1,PIK3R2,TLR9,TRIB3,PTPN11,SOS1,SOS1,FGF23,IRS4,IRS2,IRS2,FGF18,FGF17,FGF16,KL,GAB2,FGF19",IRS-related events triggered by IGF1R,58

R-HSA-2428933,"GRB2,GRB2,HRAS,IGF1,IGF1R,IGF2,KRAS,NRAS,SHC1,SHC1,SOS1,SOS1",SHC-related events triggered by IGF1R,12

R-HSA-2453864,"OPN1MW3,RDH12,NAPEPLD,ABCA4,OPN1MW,RBP1,RBP4,RDH5,RLBP1,OPN1SW,STRA6,TTR,OPN1MW2,LRAT",Retinoid cycle disease events,14

R-HSA-2453902,"DHRS9,SDR9C7,RDH12,RDH10,NAPEPLD,ABCA4,ABCA4,CYP4V2,HSD17B1,MYO7A,RDH8,RDH11,RBP1,RBP3,RBP4,RDH5,RHO,RLBP1,RPE65,STRA6,TTR,RDH16,HSD17B6,LRAT",The canonical retinoid cycle in rods (twilight vision),24

R-HSA-2454202,"RASGRP1,PSME3,PSMD14,RASGRP2,VAV3,VAV3,TAB1,AHCYL1,MALT1,MALT1,CHUK,RASGRP4,PSMB11,PSMA8,FCER1A,FCER1A,MS4A2,MS4A2,FCER1G,FCER1G,TAB2,PSME4,FBXW11,FOS,FYN,TAB3,LAT,LAT,GRB2,GRB2,HRAS,IKBKB,ITK,ITK,ITPR1,ITPR2,ITPR3,JUN,KRAS,TMEM189-UBE2V1,LCP2,LCP2,LYN,LYN,MAP3K1,MAP3K1,NFATC1,NFATC2,NFATC3,NFKB1,NFKBIA,NRAS,PAK1,PAK1,PAK2,PAK2,PDPK1,PIK3CA,PIK3CB,PIK3R1,PIK3R2,PLCG1,PLCG1,PLCG2,PLCG2,PPP3CA,PPP3CB,PPP3R1,PRKCQ,PRKCQ,MAPK1,MAPK3,MAPK8,MAPK9,MAPK10,MAP2K7,PSMA1,PSMA2,PSMA3,PSMA4,PSMA5,PSMA6,PSMA7,PSMB1,PSMB2,PSMB3,PSMB4,PSMB5,PSMB6,PSMB7,PSMB8,PSMB9,PSMB10,PSMC1,PSMC2,PSMC3,PSMC4,PSMC5,PSMC6,PSMD1,PSMD2,PSMD3,PSMD4,PSMD5,PSMD7,PSMD8,PSMD9,PSMD10,PSMD11,PSMD12,PSMD13,PSME1,PSME2,RAC1,RELA,RPS27A,MAP2K4,SHC1,SHC1,SKP1,SOS1,SOS1,SYK,SYK,MAP3K7,BTK,BTK,TEC,TEC,TRAF6,TXK,TXK,UBA52,UBB,UBC,UBE2D1,UBE2D2,UBE2N,UBE2V1,VAV1,VAV1,VAV2,VAV2,LAT2,LAT2,SEM1,CALM1,CALM1,CALM2,CALM2,CALM3,CALM3,CARD11,CARD11,CUL1,IKBKG,BCL10,BCL10,BTRC,GRAP2,GRAP2,PSMF1,GAB2,GAB2,PSMD6,CDC34",Fc epsilon receptor (FCERI) signaling,166

R-HSA-2465910,"ARPP19,ARPP19,ENSA,ENSA,PPP2CA,PPP2CB,PPP2R1A,PPP2R1B,PPP2R2D,MASTL,MASTL,CCNB1,CDK1",MASTL Facilitates Mitotic Progression,13

R-HSA-2466712,NAPEPLD,"Biosynthesis of A2E, implicated in retinal degradation",1

R-HSA-2467813,"CENPS-CORT,PMF1-BGLAP,PSME3,PSMD14,STAG1,TUBA1B,TUBB3,TUBB4A,TUBB4B,ANAPC10,NDC80,CENPA,CENPC,CENPE,CENPF,NUDC,STAG2,KIF2C,UBE2C,ZWINT,PMF1,TUBA3E,CDCA5,TUBA3D,ANAPC16,PSMB11,DYNLL2,PSMA8,SPC24,SGO2,SGO1,DYNC1H1,DYNC1I1,DYNC1I2,DYNC1LI2,SKA1,MAPRE1,PDS5B,WAPL,CLASP2,PSME4,PDS5A,NUP160,CLASP1,ITGB3BP,CDC26,CENPI,ANAPC15,AHCTF1,NSL1,UBE2S,ANAPC2,ANAPC4,BIRC5,TUBB8,TUBB2B,SKA2,NUP43,INCENP,CENPS,KIF2A,CENPP,MAD2L1,NUP98,PAFAH1B1,DYNC1LI1,ANAPC5,ANAPC7,ANAPC11,TUBA8,PLK1,NDE1,ERCC6L,SPDL1,PPP1CC,ZWILCH,CDCA8,PPP2CA,PPP2CB,CENPQ,PPP2R1A,PPP2R1B,PPP2R5A,PPP2R5B,PPP2R5C,PPP2R5D,PPP2R5E,NUP133,CENPN,HDAC8,RCC2,PSMA1,PSMA2,PSMA3,PSMA4,PSMA5,PSMA6,PSMA7,PSMB1,PSMB2,PSMB3,PSMB4,PSMB5,PSMB6,PSMB7,PSMB8,PSMB9,PSMB10,PSMC1,PSMC2,PSMC3,PSMC4,PSMC5,PSMC6,PSMD1,PSMD2,KNL1,PSMD3,PSMD4,PSMD5,NUP107,PSMD7,PSMD8,PSMD9,PSMD10,PSMD11,PSMD12,PSMD13,PSME1,PSME2,SPC25,TAOK1,RAD21,RANBP2,RANGAP1,RPS27,RPS27A,CLIP1,SEC13,CENPK,ANAPC1,CENPH,BUB1,BUB1B,TUBA4A,TUBA3C,TUBB2A,UBA52,UBB,UBC,UBE2D1,UBE2E1,XPO1,TUBA1A,MIS12,CENPM,NUP37,CENPO,CENPU,SEM1,TUBAL3,NUP85,DSN1,CENPT,B9D2,TUBB1,NDEL1,SEH1L,KIF18A,SMC1A,NUF2,MAD1L1,TUBB6,KIF2B,TUBA1C,DYNLL1,CDC23,CDC16,SMC3,CENPL,ZW10,BUB3,AURKB,PTTG1,PSMF1,ESPL1,KNTC1,CKAP5,PSMD6,CDC20,CDC27",Separation of Sister Chromatids,191

R-HSA-2468052,"STAG1,STAG2,CDCA5,ESCO1,ESCO2,PDS5B,WAPL,PDS5A,RAD21,SMC1A,SMC3",Establishment of Sister Chromatid Cohesion,11

R-HSA-2470946,"STAG1,STAG2,PDS5B,WAPL,PDS5A,MAU2,NIPBL,RAD21,SMC1A,SMC3",Cohesin Loading onto Chromatin,10

R-HSA-2473224,"FSTL3,FST,INHBA,INHBB",Antagonism of Activin by Follistatin,4

R-HSA-2474795,"OPN1MW3,RDH12,NAPEPLD,ABCA4,OPN1MW,RBP1,RBP4,RDH5,RLBP1,OPN1SW,STRA6,TTR,OPN1MW2,LRAT",Diseases associated with visual transduction,14

R-HSA-2485179,"CNGB1,CNGA1,GNAT1,GNAT1,GNB1,GNB1,GNGT1,GNGT1,PDE6A,PDE6A,PDE6G,PDE6G,PDE6B,PDE6B,RHO,RHO,SAG,SLC24A1",Activation of the phototransduction cascade,18

R-HSA-2500257,"CENPS-CORT,PMF1-BGLAP,STAG1,TUBA1B,TUBB3,TUBB4A,TUBB4B,NDC80,CENPA,CENPC,CENPE,CENPF,NUDC,STAG2,KIF2C,ZWINT,PMF1,TUBA3E,CDCA5,TUBA3D,DYNLL2,SPC24,SGO2,SGO1,DYNC1H1,DYNC1I1,DYNC1I2,DYNC1LI2,SKA1,MAPRE1,PDS5B,WAPL,CLASP2,PDS5A,NUP160,CLASP1,ITGB3BP,CENPI,AHCTF1,NSL1,BIRC5,TUBB8,TUBB2B,SKA2,NUP43,INCENP,CENPS,KIF2A,CENPP,MAD2L1,NUP98,PAFAH1B1,DYNC1LI1,TUBA8,PLK1,NDE1,ERCC6L,SPDL1,PPP1CC,ZWILCH,CDCA8,PPP2CA,PPP2CB,CENPQ,PPP2R1A,PPP2R1B,PPP2R5A,PPP2R5B,PPP2R5C,PPP2R5D,PPP2R5E,NUP133,CENPN,HDAC8,RCC2,KNL1,NUP107,SPC25,TAOK1,RAD21,RANBP2,RANGAP1,RPS27,CLIP1,SEC13,CENPK,CENPH,BUB1,BUB1B,TUBA4A,TUBA3C,TUBB2A,XPO1,TUBA1A,MIS12,CENPM,NUP37,CENPO,CENPU,TUBAL3,NUP85,DSN1,CENPT,B9D2,TUBB1,NDEL1,SEH1L,KIF18A,SMC1A,NUF2,MAD1L1,TUBB6,KIF2B,TUBA1C,DYNLL1,CCNB1,SMC3,CCNB2,CENPL,ZW10,BUB3,AURKB,KNTC1,CKAP5,CDK1,CDC20",Resolution of Sister Chromatid Cohesion,126

R-HSA-2514853,"SMC4,SMC4,SMC2,SMC2,CSNK2A1,CSNK2A2,CSNK2B,NCAPH,NCAPH,NCAPG,NCAPG,CCNB1,CCNB2,CDK1,NCAPD2,NCAPD2",Condensation of Prometaphase Chromosomes,16

R-HSA-2514856,"CHURC1-FNTB,GNB5,GNB5,METAP2,CNGB1,CNGA1,GRK7,METAP1,FNTA,FNTB,GNAT1,GNAT1,GNB1,GNB1,GNGT1,GNGT1,GRK4,GUCA1A,GUCA1B,GUCY2F,GUCY2D,RGS9BP,RGS9BP,NMT1,PDE6A,PDE6A,PDE6G,PDE6G,PDE6B,PDE6B,PPEF1,PRKCA,PRKCQ,RCVRN,RCVRN,RHO,RHO,GRK1,GRK1,SAG,SAG,CAMKMT,CALM1,CALM1,CALM2,CALM2,CALM3,CALM3,RGS9,RGS9,SLC24A1,NMT2,GUCA1C",The phototransduction cascade,53

R-HSA-2514859,"CHURC1-FNTB,GNB5,GNB5,METAP2,CNGB1,CNGA1,GRK7,METAP1,FNTA,FNTB,GNAT1,GNAT1,GNB1,GNGT1,GNGT1,GRK4,GUCA1A,GUCA1B,GUCY2F,GUCY2D,RGS9BP,RGS9BP,NMT1,PDE6A,PDE6G,PDE6B,PPEF1,PRKCA,PRKCQ,RCVRN,RCVRN,RHO,RHO,GRK1,GRK1,SAG,SAG,CAMKMT,CALM1,CALM1,CALM2,CALM2,CALM3,CALM3,RGS9,RGS9,NMT2,GUCA1C","Inactivation, recovery and regulation of the phototransduction cascade",48

R-HSA-2534343,"ADAM30,ZP1,ADAM2,B4GALT1,OVGP1,ZP4,SPAM1,ZP2,ZP3,ADAM21,ADAM20",Interaction With Cumulus Cells And The Zona Pellucida,11

R-HSA-2555396,"CENPS-CORT,RNF103-CHMP3,PMF1-BGLAP,PSME3,PSMD14,STAG1,TUBA1B,TUBB3,TUBB4A,TUBB4B,ANAPC10,NDC80,CENPA,CENPC,CENPE,CENPF,NUDC,STAG2,KIF2C,RCC1,UBE2C,ZWINT,PMF1,TUBA3E,CDCA5,TUBA3D,ANAPC16,PSMB11,CHMP4B,NUP35,NUP35,DYNLL2,PSMA8,SPC24,SGO2,SGO1,DYNC1H1,DYNC1I1,DYNC1I2,DYNC1LI2,CC2D1B,EMD,SKA1,LEMD2,MAPRE1,SIRT2,PDS5B,WAPL,CLASP2,ANKLE2,NUP205,NUP205,PSME4,PDS5A,NUP160,NUP160,CLASP1,ITGB3BP,NUP188,NUP188,LEMD3,NUP62,CDC26,CENPI,ANAPC15,AHCTF1,AHCTF1,NSL1,CHMP2B,FBXO5,VPS4A,CHMP2A,UBE2S,CHMP4A,ANAPC2,ANAPC4,BIRC5,TUBB8,TUBB2B,SKA2,NUP43,NUP43,INCENP,CENPS,KIF2A,KPNB1,KPNB1,TNPO1,LBR,LMNA,LMNB1,CENPP,MAD2L1,NUP98,NUP98,PAFAH1B1,DYNC1LI1,ANAPC5,ANAPC7,ANAPC11,CHMP3,TUBA8,NUP54,PLK1,NDE1,ERCC6L,SPDL1,PPP1CC,ZWILCH,CDCA8,PPP2CA,PPP2CB,CENPQ,PPP2R1A,PPP2R1B,PPP2R2A,PPP2R5A,PPP2R5B,PPP2R5C,PPP2R5D,PPP2R5E,NDC1,NDC1,NUP133,NUP133,CENPN,HDAC8,RCC2,PSMA1,PSMA2,PSMA3,PSMA4,PSMA5,PSMA6,PSMA7,PSMB1,PSMB2,PSMB3,PSMB4,PSMB5,PSMB6,PSMB7,PSMB8,PSMB9,PSMB10,PSMC1,PSMC2,PSMC3,PSMC4,PSMC5,PSMC6,PSMD1,PSMD2,KNL1,PSMD3,PSMD4,PSMD5,NUP107,NUP107,PSMD7,PSMD8,PSMD9,PSMD10,PSMD11,PSMD12,PSMD13,PSME1,PSME2,SPC25,TAOK1,RAD21,RAN,RAN,RANBP2,RANGAP1,RPS27,RPS27A,CLIP1,SEC13,SEC13,CENPK,ANAPC1,CENPH,SPAST,BUB1,BUB1B,TMPO,TUBA4A,TUBA3C,TUBB2A,UBA52,UBB,UBC,UBE2D1,UBE2E1,UBE2I,SUMO1,VRK1,VRK2,XPO1,TUBA1A,MIS12,CENPM,NUP37,NUP37,CENPO,CHMP6,CENPU,SEM1,TUBAL3,NUP85,NUP85,DSN1,CENPT,B9D2,TUBB1,NDEL1,SEH1L,SEH1L,KIF18A,SMC1A,NUF2,MAD1L1,TUBB6,KIF2B,TUBA1C,DYNLL1,CDC23,BANF1,CDC16,CCNB1,SMC3,CCNB2,CENPL,CHMP7,ZW10,BUB3,AURKB,PTTG1,CHMP4C,PSMF1,NUP155,NUP155,NUP93,NUP93,ESPL1,KNTC1,CKAP5,IST1,NUP58,CDK1,PSMD6,POM121,POM121,CDC20,CDC27",Mitotic Metaphase and Anaphase,256

R-HSA-2559580,"COMMD3-BMI1,CDK4,CDK6,LOC102724334,CDKN2A,CDKN2B,CDKN2C,CDKN2D,H4-16,H3C14,H2BU1,MAPK14,E2F1,E2F2,E2F3,PHC1,PHC2,AGO3,AGO4,EZH2,SCMH1,TNIK,TNRC6B,KDM6B,CBX6,SUZ12,FOS,H2BC1,AGO1,TNRC6A,H2AC8,H2AC7,H2AX,H2AZ1,H2BC5,H2BC3,H3-3A,H3-3B,H3C15,IFNB1,JUN,MDM2,MDM4,MAP3K5,MAP3K5,MOV10,H2AB1,MINK1,H4C15,H2AJ,MAPK1,MAPK3,MAPK8,MAPK11,MAPK9,MAPK10,MAP2K3,MAP2K6,MAP2K7,CBX8,TNRC6C,RBBP4,RBBP7,RING1,RNF2,RPS27A,MAP2K4,MAP2K4,BMI1,H3C13,TFDP1,TFDP2,TP53,H2AC19,TXN,UBA52,UBB,UBC,MAPKAPK3,PHC3,H4C9,H2AC14,H2AC6,H2AC4,H2AC18,H2AC20,H2BC8,H2BC13,H2BC15,H2BC14,H2BC7,H2BC6,H2BC9,H2BC10,H2BC4,H2BC17,H2BC21,H3C1,H3C4,H3C3,H3C6,H3C11,H3C8,H3C12,H3C10,H3C2,H4C1,H4C4,H4C6,H4C12,H4C11,H4C3,H4C8,H4C2,H4C5,H4C13,H4C14,CBX2,H2BC12,CBX4,MAPKAPK5,EED,H3C7,H2BC11,MAPKAPK2,H2AZ2,MAP4K4",Oxidative Stress Induced Senescence,127

R-HSA-2559582,"CDK2,CDK4,CDK6,CDKN1A,CDKN1B,LOC102724334,CDKN2A,CDKN2B,CDKN2C,CDKN2D,ANAPC10,CEBPB,CEBPB,EHMT2,UBE2C,ANAPC16,H4-16,H3C14,H2BU1,FOS,CDC26,H2BC1,ANAPC15,VENTX,UBE2S,ANAPC2,ANAPC4,H2AC8,H2AC7,H2AX,H2AZ1,H2BC5,H2BC3,H3-3A,H3-3B,H3C15,IGFBP7,IL1A,IL6,CXCL8,JUN,H2AB1,NFKB1,FZR1,ANAPC5,ANAPC7,ANAPC11,H4C15,H2AJ,MAPK1,MAPK3,MAPK7,RELA,RPS6KA1,RPS6KA1,RPS6KA2,RPS6KA2,RPS6KA3,RPS6KA3,RPS27A,ANAPC1,H3C13,STAT3,H2AC19,UBA52,UBB,UBC,UBE2D1,UBE2E1,EHMT1,H4C9,H2AC14,H2AC6,H2AC4,H2AC18,H2AC20,H2BC8,H2BC13,H2BC15,H2BC14,H2BC7,H2BC6,H2BC9,H2BC10,H2BC4,H2BC17,H2BC21,H3C1,H3C4,H3C3,H3C6,H3C11,H3C8,H3C12,H3C10,H3C2,H4C1,H4C4,H4C6,H4C12,H4C11,H4C3,H4C8,H4C2,H4C5,H4C13,H4C14,H2BC12,CDC23,CDC16,CCNA2,CCNA1,H3C7,H2BC11,H2AZ2,CDC27",Senescence-Associated Secretory Phenotype (SASP),116

R-HSA-2559583,"COMMD3-BMI1,RAD50,CDK2,CDK4,CDK6,CDKN1A,CDKN1B,LOC102724334,CDKN2A,CDKN2B,CDKN2C,CDKN2D,ANAPC10,CEBPB,CEBPB,KAT5,EHMT2,UBE2C,ANAPC16,H4-16,H3C14,H2BU1,MAPK14,E2F1,E2F2,E2F3,PHC1,PHC2,AGO3,AGO4,ERF,ERF,ETS1,ETS2,EZH2,SCMH1,TNIK,TNRC6B,KDM6B,CBX6,SUZ12,CABIN1,FOS,CDC26,H2BC1,ASF1A,ANAPC15,POT1,TINF2,AGO1,VENTX,TNRC6A,UBE2S,UBN1,ANAPC2,ANAPC4,H1-0,H1-2,H1-3,H1-4,H1-5,H2AC8,H2AC7,H2AX,H2AZ1,H2BC5,H2BC3,H3-3A,H3-3B,H1-1,HMGA1,H3C15,ID1,IFNB1,IGFBP7,IL1A,IL6,CXCL8,JUN,LMNB1,MDM2,MDM4,MAP3K5,MAP3K5,MOV10,MRE11,NBN,ATM,H2AB1,NFKB1,MINK1,FZR1,ANAPC5,ANAPC7,ANAPC11,TERF2IP,H4C15,H2AJ,MAPK1,MAPK1,MAPK3,MAPK3,MAPK7,MAPK8,MAPK11,MAPK9,MAPK10,MAP2K3,MAP2K6,MAP2K7,CBX8,EP400,TNRC6C,RB1,RBBP4,RBBP7,RELA,RING1,RNF2,RPS6KA1,RPS6KA1,RPS6KA2,RPS6KA2,RPS6KA3,RPS6KA3,RPS27A,MAP2K4,MAP2K4,ANAPC1,BMI1,ACD,H3C13,SP1,STAT3,TERF1,TERF2,TFDP1,TFDP2,TP53,H2AC19,HIRA,TXN,UBA52,UBB,UBC,UBE2D1,UBE2E1,MAPKAPK3,EHMT1,PHC3,HMGA2,H3-4,H4C9,H2AC14,H2AC6,H2AC4,H2AC18,H2AC20,H2BC8,H2BC13,H2BC15,H2BC14,H2BC7,H2BC6,H2BC9,H2BC10,H2BC4,H2BC17,H2BC21,H3C1,H3C4,H3C3,H3C6,H3C11,H3C8,H3C12,H3C10,H3C2,H4C1,H4C4,H4C6,H4C12,H4C11,H4C3,H4C8,H4C2,H4C5,H4C13,H4C14,CBX2,H2BC12,CBX4,MAPKAPK5,CDC23,EED,CDC16,CCNA2,CCNA1,H3C7,H2BC11,CCNE1,CCNE2,MAPKAPK2,H2AZ2,MAP4K4,CDC27",Cellular Senescence,206

R-HSA-2559584,"CABIN1,ASF1A,UBN1,H1-0,H1-2,H1-3,H1-4,H1-5,H1-1,HMGA1,LMNB1,EP400,RB1,TP53,HIRA,HMGA2",Formation of Senescence-Associated Heterochromatin Foci (SAHF),16

R-HSA-2559585,"CDK4,CDK6,CDKN2A,CDKN2B,CDKN2C,CDKN2D,E2F1,E2F2,E2F3,AGO3,AGO4,ERF,ERF,ETS1,ETS2,TNRC6B,AGO1,TNRC6A,ID1,MDM2,MDM4,MOV10,MAPK1,MAPK1,MAPK3,MAPK3,TNRC6C,RB1,RPS27A,SP1,TFDP1,TFDP2,TP53,UBA52,UBB,UBC",Oncogene Induced Senescence,36

R-HSA-2559586,"RAD50,CDK2,CDKN1A,CDKN1B,LOC102724334,KAT5,H4-16,H2BU1,CABIN1,H2BC1,ASF1A,POT1,TINF2,UBN1,H1-0,H1-2,H1-3,H1-4,H1-5,H2AC8,H2AC7,H2AX,H2AZ1,H2BC5,H2BC3,H1-1,HMGA1,LMNB1,MRE11,NBN,ATM,H2AB1,TERF2IP,H4C15,H2AJ,EP400,RB1,ACD,TERF1,TERF2,TP53,H2AC19,HIRA,HMGA2,H3-4,H4C9,H2AC14,H2AC6,H2AC4,H2AC18,H2AC20,H2BC8,H2BC13,H2BC15,H2BC14,H2BC7,H2BC6,H2BC9,H2BC10,H2BC4,H2BC17,H2BC21,H4C1,H4C4,H4C6,H4C12,H4C11,H4C3,H4C8,H4C2,H4C5,H4C13,H4C14,H2BC12,CCNA2,CCNA1,H2BC11,CCNE1,CCNE2,H2AZ2",DNA Damage/Telomere Stress Induced Senescence,80

R-HSA-2562578,"TMED7-TICAM2,RIPK3,TICAM1,LY96,TICAM2,TLR4,CASP8,RIPK1,FADD,CD14",TRIF-mediated programmed cell death,10

R-HSA-2564830,"NUBP2,ERCC2,ABCB7,NDOR1,NUBP1,CIAO2B,RTEL1,POLD1,CIAPIN1,MMS19,CIAO3,BRIP1,CIAO1",Cytosolic iron-sulfur cluster assembly,13

R-HSA-2565942,"ACTR1A,OPTN,AKAP9,TPTEP2-CSNK1E,TUBB4A,TUBB4B,DCTN2,CETN2,PLK4,SDCCAG8,CNTRL,FGFR1OP,CEP250,DCTN3,HAUS1,NEDD1,CSNK1D,CSNK1E,DCTN1,DYNC1H1,DYNC1I2,TUBB,CEP164,MAPRE1,NINL,CEP131,CEP152,FBXW11,CLASP1,HAUS5,HSP90AA1,RAB8A,PPP1R12A,PPP1R12B,NEK2,ODF2,PAFAH1B1,PCM1,PCNT,PLK1,HAUS6,NDE1,HAUS4,PPP1CB,CEP192,HAUS2,PPP2R1A,HAUS7,PRKACA,CEP72,CDK5RAP2,PRKAR2B,CENPJ,RPS27A,SKP1,AURKA,TUBA4A,TUBG1,UBA52,UBB,UBC,YWHAE,YWHAG,ALMS1,TUBA1A,HAUS3,BORA,CEP76,CEP290,CEP63,CEP70,CEP78,CUL1,OFD1,AJUBA,SSNA1,DYNLL1,CCNB1,BTRC,CCNB2,HAUS8,CEP41,CEP135,CEP57,CCP110,CKAP5,SFI1,CDK1",Regulation of PLK1 Activity at G2/M Transition,88

R-HSA-2586552,"SH2B1,IRS1,JAK2,LEP,LEPR,PTPN11,STAT3,STAT5A,STAT5A,STAT5B,STAT5B,IRS2,SOCS3",Signaling by Leptin,13

R-HSA-2644602,"HDAC6,HDAC5,MAMLD1,ADAM10,CDK8,CREBBP,MIB2,JAG1,EP300,SNW1,NCSTN,HEY1,HEY2,KAT2A,HEYL,DLL1,HDAC1,HDAC2,HES1,RBPJ,JAG2,HES5,MYC,NOTCH1,APH1A,HDAC7,NEURL1B,DLL4,FBXW7,MAML3,PSENEN,HDAC8,PSEN1,PSEN2,MIB1,RPS27A,SKP1,ADAM17,TBL1X,UBA52,UBB,UBC,TBL1XR1,HDAC11,APH1B,HDAC10,MAML2,CUL1,HDAC3,KAT2B,CCNC,NEURL1,NCOR1,NCOR2,HDAC9,HDAC4,MAML1,RBX1",Signaling by NOTCH1 PEST Domain Mutants in Cancer,58

R-HSA-2644603,"HDAC6,HDAC5,MAMLD1,ADAM10,CDK8,CREBBP,MIB2,JAG1,EP300,SNW1,NCSTN,HEY1,HEY2,KAT2A,HEYL,DLL1,HDAC1,HDAC2,HES1,RBPJ,JAG2,HES5,MYC,NOTCH1,APH1A,HDAC7,NEURL1B,DLL4,FBXW7,MAML3,PSENEN,HDAC8,PSEN1,PSEN2,MIB1,RPS27A,SKP1,ADAM17,TBL1X,UBA52,UBB,UBC,TBL1XR1,HDAC11,APH1B,HDAC10,MAML2,CUL1,HDAC3,KAT2B,CCNC,NEURL1,NCOR1,NCOR2,HDAC9,HDAC4,MAML1,RBX1",Signaling by NOTCH1 in Cancer,58

R-HSA-2644605,"NOTCH1,FBXW7,SKP1,CUL1,RBX1",FBXW7 Mutants and NOTCH1 in Cancer,5

R-HSA-2644606,"HDAC6,HDAC5,MAMLD1,ADAM10,CDK8,CREBBP,MIB2,JAG1,EP300,SNW1,NCSTN,HEY1,HEY2,KAT2A,HEYL,DLL1,HDAC1,HDAC2,HES1,RBPJ,JAG2,HES5,MYC,NOTCH1,APH1A,HDAC7,NEURL1B,DLL4,FBXW7,MAML3,PSENEN,HDAC8,PSEN1,PSEN2,MIB1,RPS27A,SKP1,ADAM17,TBL1X,UBA52,UBB,UBC,TBL1XR1,HDAC11,APH1B,HDAC10,MAML2,CUL1,HDAC3,KAT2B,CCNC,NEURL1,NCOR1,NCOR2,HDAC9,HDAC4,MAML1,RBX1",Constitutive Signaling by NOTCH1 PEST Domain Mutants,58

R-HSA-2644607,"NOTCH1,FBXW7,SKP1,CUL1,RBX1",Loss of Function of FBXW7 in Cancer and NOTCH1 Signaling,5

R-HSA-264642,"UNC13B,CPLX1,CHAT,RIMS1,RAB3A,SLC5A7,SLC18A3,SNAP25,STX1A,STXBP1,VAMP2,SYT1,PPFIA4,PPFIA2,PPFIA1,PPFIA3,TSPOAP1",Acetylcholine Neurotransmitter Release Cycle,17

R-HSA-264870,"ADD1,GAS2,DBNL,GSN,MAPT,PLEC,SPTAN1,VIM,CASP3,CASP6,CASP7,CASP8",Caspase-mediated cleavage of cytoskeletal proteins,12

R-HSA-264876,"EXOC5,EXOC3,CPE,SLC30A7,EXOC8,SLC30A8,EXOC7,MYRIP,ERO1A,INS,INS,KIF5A,KIF5B,KIF5C,MYO5A,PCSK1,PCSK2,EXOC6,SLC30A6,EXOC1,EXOC2,ERO1B,CLTRN,RAB27A,EXOC4,SLC30A5,STX1A,VAMP2",Insulin processing,28

R-HSA-2660825,"ADAM10,JAG1,DLL1,JAG2,NOTCH1,DLL4,ADAM17",Signaling by NOTCH1 t(7;9)(NOTCH1:M1580_K2555) Translocation Mutant,7

R-HSA-2660826,"ADAM10,JAG1,DLL1,JAG2,NOTCH1,DLL4,ADAM17",Constitutive Signaling by NOTCH1 t(7;9)(NOTCH1:M1580_K2555) Translocation Mutant,7

R-HSA-2672351,"C8orf44-SGK3,SGK2,TRDN,SLC17A3,RIPK3,WWP1,CLCA1,CLCN1,CLCN2,CLCN3,CLCN4,CLCN5,CLCN6,CLCN7,CLCNKA,CLCNKB,CLIC2,ANO4,SLC9B2,TRPM6,BEST3,SLC9B1,STOML3,TRPV3,TSC22D3,ANO6,MLKL,ANO5,STOM,TPCN2,CLCA4,FKBP1B,NEDD4L,SGK3,MCOLN2,NALCN,TRPC4AP,BEST4,SLC9C2,UNC80,SLC9C1,OSTM1,TRPM5,ANO9,ASIC2,ASIC1,TRPM1,ASPH,ANO7,TRPV2,ASIC5,TPCN1,TRPM4,TRPM7,BEST2,ANO1,ANO10,MCOLN3,TRPV6,ASIC4,TRPV5,ANO2,TRPC7,MCOLN1,TTYH1,UNC79,ANO8,RAF1,TRPV4,RPS27A,RYR1,RYR2,RYR3,SCNN1A,SCNN1B,SCNN1D,SCNN1G,ANO3,SGK1,WNK1,WNK4,WNK3,WNK2,SRI,TRPC1,TRPC3,TRPC4,TRPC5,TRPC6,TRPM2,UBA52,UBB,UBC,BEST1,TRPV1,BSND,TRPM8,TRPM3,CALM1,CALM2,TTYH3,CALM3,CASQ1,CASQ2,RIPK1,TRPA1,ASIC3,TTYH2,CLCA2",Stimuli-sensing channels,109

R-HSA-2682334,"ARPC5,ARPC4,ARPC3,ARPC1B,ACTR3,ACTR2,ARPC2,ADAM10,MYL12B,MYL9,VAV3,ARPC1A,MYL12A,MYL12A,CFL1,AP2M1,AP2S1,CLTA,CLTB,CLTC,AP2A1,AP2A2,AP2B1,DNM1,EFNA1,EFNA1,EFNA2,EFNA2,EFNA3,EFNA3,EFNA4,EFNA4,EFNA5,EFNA5,EFNB1,EFNB1,EFNB2,EFNB2,EFNB3,EFNB3,EPHA2,EPHA2,EPHA1,EPHA1,EPHA3,EPHA3,EPHA4,EPHA4,EPHA5,EPHA5,EPHA7,EPHA7,EPHA8,EPHA8,EPHB1,EPHB1,EPHB2,EPHB2,EPHB3,EPHB3,EPHB4,EPHB4,EPHB6,EPHB6,NCSTN,FYN,FYN,NGEF,NGEF,EPHA10,EPHA10,EPHA6,EPHA6,GIT1,GIT1,GRIN1,GRIN1,GRIN2B,GRIN2B,HRAS,RHOA,RHOA,LIMK1,LIMK2,LYN,LYN,MMP2,MMP9,MYH9,MYH10,MYH11,MYL6,PAK1,PAK2,PAK3,APH1A,PSENEN,PSEN1,PSEN2,PTK2,PTK2,RAC1,RASA1,RASA1,ACTB,ROCK1,ROCK1,SDC2,SDCBP,ARHGEF28,ITSN1,ITSN1,SRC,SRC,TIAM1,ACTG1,VAV2,YES1,YES1,MYH14,CLTCL1,APH1B,NCK2,ARHGEF7,WASL,WASL,KALRN,ROCK2,ROCK2,CDC42,CDC42",EPH-Ephrin signaling,131

R-HSA-2691230,"ADAM10,MIB2,JAG1,DLL1,JAG2,NOTCH1,NEURL1B,DLL4,MIB1,RPS27A,ADAM17,UBA52,UBB,UBC,NEURL1",Signaling by NOTCH1 HD Domain Mutants in Cancer,15

R-HSA-2691232,"ADAM10,MIB2,JAG1,DLL1,JAG2,NOTCH1,NEURL1B,DLL4,MIB1,RPS27A,ADAM17,UBA52,UBB,UBC,NEURL1",Constitutive Signaling by NOTCH1 HD Domain Mutants,15

R-HSA-2730905,"FCER1A,FCER1A,MS4A2,MS4A2,FCER1G,FCER1G,FYN,GRB2,GRB2,LYN,LYN,PDPK1,PIK3CA,PIK3CB,PIK3R1,PIK3R2,SHC1,SHC1,SOS1,SOS1,SYK,SYK,LAT2,LAT2,GAB2,GAB2",Role of LAT2/NTAL/LAB on calcium mobilization,26

R-HSA-2855086,"MASP2,FCN1,FCN2,MASP1,FCN3",Ficolins bind to repetitive carbohydrate structures on the target cell surface,5

R-HSA-2871796,"VAV3,FCER1A,MS4A2,FCER1G,FOS,LAT,GRB2,HRAS,JUN,KRAS,LCP2,LYN,MAP3K1,MAP3K1,NRAS,PAK1,PAK1,PAK2,PAK2,PLCG1,PLCG2,MAPK1,MAPK3,MAPK8,MAPK9,MAPK10,MAP2K7,RAC1,MAP2K4,SHC1,SOS1,SYK,VAV1,VAV2,GRAP2",FCERI mediated MAPK activation,35

R-HSA-2871809,"VAV3,VAV3,AHCYL1,FCER1A,FCER1A,MS4A2,MS4A2,FCER1G,FCER1G,LAT,LAT,GRB2,GRB2,ITK,ITK,ITPR1,ITPR2,ITPR3,LCP2,LCP2,LYN,LYN,NFATC1,NFATC2,NFATC3,PLCG1,PLCG1,PLCG2,PLCG2,PPP3CA,PPP3CB,PPP3R1,SHC1,SHC1,SOS1,SOS1,SYK,SYK,BTK,BTK,TEC,TEC,TXK,TXK,VAV1,VAV1,VAV2,VAV2,CALM1,CALM1,CALM2,CALM2,CALM3,CALM3,GRAP2,GRAP2",FCERI mediated Ca+2 mobilization,56

R-HSA-2871837,"RASGRP1,PSME3,PSMD14,RASGRP2,TAB1,MALT1,MALT1,CHUK,RASGRP4,PSMB11,PSMA8,FCER1A,MS4A2,FCER1G,TAB2,PSME4,FBXW11,TAB3,IKBKB,TMEM189-UBE2V1,LYN,NFKB1,NFKBIA,PDPK1,PRKCQ,PRKCQ,PSMA1,PSMA2,PSMA3,PSMA4,PSMA5,PSMA6,PSMA7,PSMB1,PSMB2,PSMB3,PSMB4,PSMB5,PSMB6,PSMB7,PSMB8,PSMB9,PSMB10,PSMC1,PSMC2,PSMC3,PSMC4,PSMC5,PSMC6,PSMD1,PSMD2,PSMD3,PSMD4,PSMD5,PSMD7,PSMD8,PSMD9,PSMD10,PSMD11,PSMD12,PSMD13,PSME1,PSME2,RELA,RPS27A,SKP1,MAP3K7,TRAF6,UBA52,UBB,UBC,UBE2D1,UBE2D2,UBE2N,UBE2V1,SEM1,CARD11,CARD11,CUL1,IKBKG,BCL10,BCL10,BTRC,PSMF1,PSMD6,CDC34",FCERI mediated NF-kB activation,86

R-HSA-2892245,"POU5F1,SOX2,NANOG","POU5F1 (OCT4), SOX2, NANOG repress genes related to differentiation",3

R-HSA-2892247,"EPHA1,FGF2,NR6A1,FOXD3,POU5F1,DPPA4,SALL4,SALL1,SOX2,STAT3,TDGF1,ZIC3,NANOG","POU5F1 (OCT4), SOX2, NANOG activate genes related to proliferation",13

R-HSA-2894858,"HDAC6,HDAC5,MAMLD1,ADAM10,CDK8,CREBBP,MIB2,JAG1,EP300,SNW1,NCSTN,HEY1,HEY2,KAT2A,HEYL,DLL1,HDAC1,HDAC2,HES1,RBPJ,JAG2,HES5,MYC,NOTCH1,APH1A,HDAC7,NEURL1B,DLL4,FBXW7,MAML3,PSENEN,HDAC8,PSEN1,PSEN2,MIB1,RPS27A,SKP1,ADAM17,TBL1X,UBA52,UBB,UBC,TBL1XR1,HDAC11,APH1B,HDAC10,MAML2,CUL1,HDAC3,KAT2B,CCNC,NEURL1,NCOR1,NCOR2,HDAC9,HDAC4,MAML1,RBX1",Signaling by NOTCH1 HD+PEST Domain Mutants in Cancer,58

R-HSA-2894862,"HDAC6,HDAC5,MAMLD1,ADAM10,CDK8,CREBBP,MIB2,JAG1,EP300,SNW1,NCSTN,HEY1,HEY2,KAT2A,HEYL,DLL1,HDAC1,HDAC2,HES1,RBPJ,JAG2,HES5,MYC,NOTCH1,APH1A,HDAC7,NEURL1B,DLL4,FBXW7,MAML3,PSENEN,HDAC8,PSEN1,PSEN2,MIB1,RPS27A,SKP1,ADAM17,TBL1X,UBA52,UBB,UBC,TBL1XR1,HDAC11,APH1B,HDAC10,MAML2,CUL1,HDAC3,KAT2B,CCNC,NEURL1,NCOR1,NCOR2,HDAC9,HDAC4,MAML1,RBX1",Constitutive Signaling by NOTCH1 HD+PEST Domain Mutants,58

R-HSA-2978092,IDH1,Abnormal conversion of 2-oxoglutarate to 2-hydroxyglutarate,1

R-HSA-2979096,"ADAM10,CNTN1,MIB2,JAG1,NCSTN,DLL1,JAG2,MDK,NOTCH2,NOTCH2,APH1A,NEURL1B,DLL4,DLL4,PSENEN,PSEN1,PSEN2,MIB1,RPS27A,UBA52,UBB,UBC,APH1B,NEURL1",NOTCH2 Activation and Transmission of Signal to the Nucleus,24

R-HSA-2980736,"ATP6AP2,CDX2,EXOC5,CES1,CGA,CGB3,EXOC3,CMA1,CPA3,CPB1,CPB2,CPE,CRHR2,GPR119,SLC30A7,EXOC8,CTNNB1,CTSD,CTSG,CTSZ,ACE,SLC30A8,DPP4,AGT,AGT,ENPEP,EXOC7,SEC11A,SEC11A,FSHB,MYRIP,GATA4,GCG,GCG,GH1,GIP,GIP,GNB3,FFAR1,SPCS1,SPCS1,ANPEP,GRP,GZMH,ERO1A,FFAR4,GNAT3,IGF1,INHA,INHBA,INHBB,INHBC,INS,INS,ISL1,KIF5A,KIF5B,KIF5C,LEP,LHB,ACHE,MME,MYO5A,PAX6,PCSK1,PCSK1,PCSK2,GHRL,GNG13,POMC,EXOC6,SLC30A6,EXOC1,EXOC2,ERO1B,CLTRN,RAB27A,BCHE,ACE2,REN,EXOC4,SPCS3,SPCS3,MBOAT4,SLC30A5,STX1A,VAMP2,TCF7L2,TSHB,UCN,PLA2G7,INHBE,AOPEP,SEC11C,SEC11C,KLF4,CGB5,CGB8,SPCS2,SPCS2",Peptide hormone metabolism,100

R-HSA-2980766,"NUP50,NEK6,NUP42,NUP35,NEK7,EMD,LEMD2,NUP205,LPIN1,NUP210,NUP160,CTDNEP1,NUP188,LEMD3,NUP62,CNEP1R1,NUP43,LMNA,LMNB1,NUP88,NUP98,NUP54,PLK1,NDC1,NUP133,PRKCA,PRKCB,NUP107,RANBP2,SEC13,LPIN3,TMPO,TPR,VRK1,VRK2,NUP37,NUP85,NUP214,AAAS,SEH1L,RAE1,BANF1,CCNB1,CCNB2,NEK9,NUP155,LPIN2,NUP93,NUP58,CDK1,POM121,NUP153",Nuclear Envelope Breakdown,52

R-HSA-2980767,"NEK6,NEK7,PLK1,CCNB1,CCNB2,NEK9,CDK1","Activation of NIMA Kinases NEK9, NEK6, NEK7",7

R-HSA-2990846,"COMMD3-BMI1,COMMD3-BMI1,UBA2,UBA2,SAE1,SAE1,NR1H3,TRIM28,TOPORS,STAG1,CDKN2A,PIAS3,NCOA2,DDX17,CETN2,STAG2,NUP50,NUP50,PPARGC1A,CHD3,NUP42,NUP42,PARK7,UHRF2,H4-16,NUP35,NUP35,CREBBP,PARP1,CTBP1,DAXX,DDX5,RNF168,DNMT1,DNMT3A,DNMT3B,PHC1,PHC1,PHC2,PHC2,NSMCE1,EP300,SENP5,ESR1,SCMH1,SCMH1,SMC5,NUP205,NUP205,NUP210,NUP210,NUP160,NUP160,SATB2,CBX5,NUP188,NUP188,SUZ12,NUP62,NUP62,NR5A2,NR5A1,SIN3A,RWDD3,NSMCE2,HIPK2,NR3C1,SENP1,HDAC1,HDAC2,HIC1,HNRNPC,HNRNPK,BIRC5,NUP43,NUP43,INCENP,ING2,AR,MBD1,MDM2,MITF,NR3C2,NFKB2,NFKBIA,NPM1,NUP88,NUP88,NUP98,NUP98,NR4A2,EID3,PCNA,HDAC7,PIAS4,PIAS4,NOP58,PGR,NUP54,NUP54,PML,PPARA,PPARG,NSMCE4A,CDCA8,H4C15,NDC1,NDC1,NUP133,NUP133,NSMCE3,NUP107,NUP107,CBX8,CBX8,MRTFA,RAD21,RAD52,RANBP2,RANBP2,RANGAP1,RARA,SENP2,ZNF350,RELA,TRIM27,RING1,RING1,RNF2,RNF2,RORA,RPA1,RXRA,SAFB,SATB1,SEC13,SEC13,BLM,BMI1,BMI1,SUMO3,SUMO3,SUMO2,SUMO2,SP3,SP100,FOXL2,BRCA1,AURKA,TDG,TFAP2A,TFAP2B,TFAP2C,THRA,THRB,TOP1,TOP2A,TOP2A,TOP2B,TP53,TP53BP1,TPR,TPR,NR2C1,UBE2I,UBE2I,SUMO1,SUMO1,NR1H2,VDR,VHL,WRN,XPC,XRCC4,ZNF131,PCGF2,PCGF2,NUP37,NUP37,SMC6,NUP85,NUP85,PHC3,PHC3,NUP214,NUP214,AAAS,AAAS,SEH1L,SEH1L,NRIP1,SMC1A,H4C9,H4C1,H4C4,H4C6,H4C12,H4C11,H4C3,H4C8,H4C2,H4C5,H4C13,H4C14,L3MBTL2,CBX2,CBX2,RAE1,RAE1,IKBKG,CBX4,CBX4,PIAS1,PIAS1,NCOA1,NR1I2,HERC2,PIAS2,PIAS2,MTA1,SMC3,ZBED1,AURKB,NCOR2,NUP155,NUP155,IKBKE,MDC1,NUP93,NUP93,HDAC4,NUP58,NUP58,POM121,POM121,NR1H4,NUP153,NUP153,CASP8AP2",SUMOylation,239

R-HSA-2995383,"EMD,LEMD2,SIRT2,ANKLE2,LEMD3,KPNB1,LBR,LMNA,LMNB1,PPP2CA,PPP2R1A,PPP2R2A,TMPO,VRK1,VRK2,BANF1,CCNB1,CCNB2,CDK1",Initiation of Nuclear Envelope (NE) Reformation,19

R-HSA-2995410,"RNF103-CHMP3,TUBA1B,TUBB3,TUBB4A,TUBB4B,RCC1,TUBA3E,TUBA3D,CHMP4B,NUP35,NUP35,CC2D1B,EMD,LEMD2,SIRT2,ANKLE2,NUP205,NUP205,NUP160,NUP160,NUP188,NUP188,LEMD3,NUP62,AHCTF1,AHCTF1,CHMP2B,VPS4A,CHMP2A,CHMP4A,TUBB8,TUBB2B,NUP43,NUP43,KPNB1,KPNB1,TNPO1,LBR,LMNA,LMNB1,NUP98,NUP98,CHMP3,TUBA8,NUP54,PPP2CA,PPP2R1A,PPP2R2A,NDC1,NDC1,NUP133,NUP133,NUP107,NUP107,RAN,RAN,RANGAP1,SEC13,SEC13,SPAST,TMPO,TUBA4A,TUBA3C,TUBB2A,UBE2I,SUMO1,VRK1,VRK2,TUBA1A,NUP37,NUP37,CHMP6,TUBAL3,NUP85,NUP85,TUBB1,SEH1L,SEH1L,TUBB6,TUBA1C,BANF1,CCNB1,CCNB2,CHMP7,CHMP4C,NUP155,NUP155,NUP93,NUP93,IST1,NUP58,CDK1,POM121,POM121",Nuclear Envelope (NE) Reassembly,94

R-HSA-3000157,"LAMC3,LAMC3,COL4A1,COL4A1,COL4A2,COL4A2,COL4A3,COL4A3,COL4A4,COL4A4,COL4A5,COL4A5,COL4A6,COL4A6,COL7A1,COL7A1,NID2,NID2,LAMA1,LAMA1,HSPG2,HSPG2,ITGA6,ITGA6,ITGA1,ITGA2,ITGA2,ITGA3,ITGA7,ITGA7,ITGAV,ITGB1,ITGB1,ITGB4,LAMA2,LAMA2,LAMA3,LAMA3,LAMA4,LAMA4,LAMA5,LAMA5,LAMB1,LAMB1,LAMB2,LAMB2,LAMB3,LAMB3,LAMC1,LAMC1,LAMC2,LAMC2,NID1,NID1,COL18A1",Laminin interactions,55

R-HSA-3000170,"COL1A1,COL1A2,COL3A1,COL5A1,COL5A2,FGF2,FN1,TNC,ITGA6,ITGA2,ITGAV,ITGAV,ITGB1,ITGB3,ITGB3,ITGB4,ITGB5,ITGB5,COL5A3,TRAPPC4,PRKCA,SDC1,SDC1,SDC2,SDC2,SDC4,SDC4,TGFB1,THBS1,VTN,CASK,ACTN1,SDC3,SDC3",Syndecan interactions,34

R-HSA-3000171,"LAMC3,COL1A1,COL1A1,COL1A2,COL1A2,COL2A1,COL3A1,COL3A1,COL4A1,COL4A2,COL4A3,COL4A4,COL4A5,COL4A6,COL5A1,COL5A1,COL5A2,COL5A2,COL10A1,COL11A1,COL11A2,DAG1,DMD,FGF2,FN1,LAMA1,HSPG2,HSPG2,TNC,ITGA6,ITGA2,ITGAV,ITGAV,ITGB1,ITGB3,ITGB3,ITGB4,ITGB5,ITGB5,AGRN,LAMA2,LAMA3,LAMA4,LAMA5,LAMB1,LAMB2,LAMB3,LAMC1,LAMC2,DDR2,COL5A3,COL5A3,TRAPPC4,PDGFA,PDGFB,PRKCA,NTN4,SDC1,SDC1,SDC2,SDC2,SDC4,SDC4,TGFB1,THBS1,TTR,VTN,DDR1,CASK,ACTN1,NRXN1,SDC3,SDC3",Non-integrin membrane-ECM interactions,73

R-HSA-3000178,"COL1A1,COL1A1,COL1A2,COL1A2,COL2A1,COL2A1,COL3A1,COL3A1,COL4A1,COL4A2,COL4A3,COL4A4,COL4A5,COL4A6,COL5A1,COL5A2,COL6A1,COL6A1,COL6A2,COL6A2,COL6A3,COL6A3,COL9A1,COL9A2,COL9A3,COMP,COL6A6,COL6A6,HAPLN1,VCAN,NCAN,DAG1,DAG1,DCN,DCN,DMP1,ACAN,DSPP,FMOD,FN1,COL6A5,COL6A5,LAMA1,HSPG2,TNC,IBSP,APP,ITGA2,ITGA2B,ITGA7,ITGA9,ITGAV,ITGAX,ITGB1,ITGB3,ITGB5,ITGB6,AGRN,AGRN,LAMA2,LAMA3,LAMA4,LAMA5,LAMB1,LAMB2,LAMC1,LRP4,LUM,MATN1,MATN3,MUSK,NCAM1,COL5A3,SERPINE1,ASPN,PTPRS,BGN,BGN,BCAN,TNN,SPARC,TGFB1,TGFB2,TGFB3,TNR,TNXB,VTN,VTN,ITGA8,MATN4",ECM proteoglycans,90

R-HSA-3000471,"SSC5D,APOA1,APOA1,APOB,APOB,SAA1,CD36,CD36,SCARB1",Scavenging by Class B Receptors,9

R-HSA-3000480,"SCGB3A2,SCGB3A2,COL1A1,COL1A2,COL3A1,COL4A1,COL4A2,FTH1,FTL,SCARA5,APOA1,APOA1,APOB,APOB,APOE,APOE,MSR1,MSR1,MASP1,HSP90B1,HSP90B1,COLEC11,COLEC12,CALR,CALR,MARCO,MARCO",Scavenging by Class A Receptors,27

R-HSA-3000484,"HYOU1,HYOU1,HSPH1,HSPH1,HSP90AA1,HSP90AA1,APOB,APOB,CALR,CALR,SCARF1,SCARF1",Scavenging by Class F Receptors,12

R-HSA-3000497,"STAB1,APOB,STAB2,SPARC",Scavenging by Class H Receptors,4

R-HSA-3065676,"UBA2,UBA2,SAE1,SAE1,SUMO3,SUMO2,SUMO1",SUMO is conjugated to E1 (UBA2:SAE1),7

R-HSA-3065678,"UBA2,UBA2,SAE1,SAE1,RWDD3,SUMO3,SUMO2,UBE2I,UBE2I,SUMO1","SUMO is transferred from E1 to E2 (UBE2I, UBC9)",10

R-HSA-3065679,"SENP5,SENP1,SENP2,SUMO3,SUMO2,SUMO1",SUMO is proteolytically processed,6

R-HSA-3108214,"COMMD3-BMI1,STAG1,CDKN2A,CETN2,STAG2,NUP50,NUP42,NUP35,PARP1,RNF168,PHC1,PHC2,NSMCE1,SCMH1,SMC5,NUP205,NUP210,NUP160,NUP188,NUP62,NSMCE2,NUP43,NUP88,NUP98,EID3,HDAC7,PIAS4,NUP54,PML,NSMCE4A,NDC1,NUP133,NSMCE3,NUP107,CBX8,RAD21,RAD52,RANBP2,RING1,RNF2,RPA1,SEC13,BLM,BMI1,SUMO3,SUMO2,SP100,BRCA1,TDG,TPR,UBE2I,SUMO1,WRN,XPC,XRCC4,PCGF2,NUP37,SMC6,NUP85,PHC3,NUP214,AAAS,SEH1L,SMC1A,CBX2,RAE1,CBX4,PIAS1,HERC2,PIAS2,SMC3,NUP155,MDC1,NUP93,NUP58,POM121,NUP153",SUMOylation of DNA damage response and repair proteins,77

R-HSA-3108232,"COMMD3-BMI1,COMMD3-BMI1,NR1H3,TRIM28,TOPORS,STAG1,CDKN2A,PIAS3,NCOA2,DDX17,CETN2,STAG2,NUP50,NUP50,PPARGC1A,CHD3,NUP42,NUP42,PARK7,UHRF2,H4-16,NUP35,NUP35,CREBBP,PARP1,CTBP1,DAXX,DDX5,RNF168,DNMT1,DNMT3A,DNMT3B,PHC1,PHC1,PHC2,PHC2,NSMCE1,EP300,ESR1,SCMH1,SCMH1,SMC5,NUP205,NUP205,NUP210,NUP210,NUP160,NUP160,SATB2,CBX5,NUP188,NUP188,SUZ12,NUP62,NUP62,NR5A2,NR5A1,SIN3A,NSMCE2,HIPK2,NR3C1,HDAC1,HDAC2,HIC1,HNRNPC,HNRNPK,BIRC5,NUP43,NUP43,INCENP,ING2,AR,MBD1,MDM2,MITF,NR3C2,NFKB2,NFKBIA,NPM1,NUP88,NUP88,NUP98,NUP98,NR4A2,EID3,PCNA,HDAC7,PIAS4,PIAS4,NOP58,PGR,NUP54,NUP54,PML,PPARA,PPARG,NSMCE4A,CDCA8,H4C15,NDC1,NDC1,NUP133,NUP133,NSMCE3,NUP107,NUP107,CBX8,CBX8,MRTFA,RAD21,RAD52,RANBP2,RANBP2,RANGAP1,RARA,ZNF350,RELA,TRIM27,RING1,RING1,RNF2,RNF2,RORA,RPA1,RXRA,SAFB,SATB1,SEC13,SEC13,BLM,BMI1,BMI1,SUMO3,SUMO3,SUMO2,SUMO2,SP3,SP100,FOXL2,BRCA1,AURKA,TDG,TFAP2A,TFAP2B,TFAP2C,THRA,THRB,TOP1,TOP2A,TOP2A,TOP2B,TP53,TP53BP1,TPR,TPR,NR2C1,UBE2I,UBE2I,SUMO1,SUMO1,NR1H2,VDR,VHL,WRN,XPC,XRCC4,ZNF131,PCGF2,PCGF2,NUP37,NUP37,SMC6,NUP85,NUP85,PHC3,PHC3,NUP214,NUP214,AAAS,AAAS,SEH1L,SEH1L,NRIP1,SMC1A,H4C9,H4C1,H4C4,H4C6,H4C12,H4C11,H4C3,H4C8,H4C2,H4C5,H4C13,H4C14,L3MBTL2,CBX2,CBX2,RAE1,RAE1,IKBKG,CBX4,CBX4,PIAS1,PIAS1,NCOA1,NR1I2,HERC2,PIAS2,PIAS2,MTA1,SMC3,ZBED1,AURKB,NCOR2,NUP155,NUP155,IKBKE,MDC1,NUP93,NUP93,HDAC4,NUP58,NUP58,POM121,POM121,NR1H4,NUP153,NUP153,CASP8AP2",SUMO E3 ligases SUMOylate target proteins,231

R-HSA-3134963,"DHX9,DHX36,IRF7,MYD88,NFKB1,NFKB2,RELA",DEx/H-box helicases activate type I IFN and inflammatory cytokines production ,7

R-HSA-3134973,"CREBBP,CTNNB1,EP300,IRF3,LRRFIP1",LRR FLII-interacting protein 1 (LRRFIP1) activates type I IFN production,5

R-HSA-3134975,"TREX1,NLRP4,TRIM32,DTX4,TBK1,STING1,IRF3,DDX41,RPS27A,TRIM21,UBA52,UBB,UBC,ZBP1,TRIM56",Regulation of innate immune responses to cytosolic DNA,15

R-HSA-3214815,"SAP18,HMG20B,CHD3,CHD4,H4-16,H3C14,H2BU1,H2AC1,KDM1A,RCOR1,H2BC1,BRMS1,GPS2,H2AC8,H2AC7,H2BC5,H2BC3,HDAC1,HDAC2,H2AC21,H3C15,H2BC18,PHF21A,ARID4B,MBD3,GATAD2A,H4C15,HDAC8,GATAD2B,MTA3,ARID4A,RBBP4,RBBP7,REST,SUDS3,H3C13,TBL1X,H2AC19,SAP30L,TBL1XR1,H4C9,H2AC13,H2AC15,H2AC14,H2AC16,H2AC6,H2AC4,H2AC17,H2AC18,H2AC20,H2BC8,H2BC13,H2BC15,H2BC14,H2BC7,H2BC6,H2BC9,H2BC10,H2BC4,H2BC17,H2BC21,H3C1,H3C4,H3C3,H3C6,H3C11,H3C8,H3C12,H3C10,H3C2,H4C1,H4C4,H4C6,H4C12,H4C11,H4C3,H4C8,H4C2,H4C5,H4C13,H4C14,HDAC10,H2AC12,H2BC12,SAP30,HDAC3,H3C7,H2AC11,H2BC11,MTA1,MTA2,H2AW,NCOR1,NCOR2",HDACs deacetylate histones,94

R-HSA-3214841,"EHMT2,WDR5,H4-16,AEBP2,H3C14,MECOM,EZH2,SETD1B,SUZ12,SETD2,H3C15,KMT5A,KMT2A,NFKB1,NFKB2,KMT5B,NSD3,H4C15,ATF7IP,ASH1L,KMT2E,SMYD2,PRDM9,KMT2C,RBBP4,RBBP5,RBBP7,RELA,PRDM16,NSD1,SMYD3,H3C13,SUV39H1,NSD2,SUV39H2,EHMT1,SETD6,KMT2D,SETD7,H4C9,H3C1,H3C4,H3C3,H3C6,H3C11,H3C8,H3C12,H3C10,H3C2,H4C1,H4C4,H4C6,H4C12,H4C11,H4C3,H4C8,H4C2,H4C5,H4C13,H4C14,SETDB2,SETD3,DOT1L,DPY30,KMT5C,EED,H3C7,ASH2L,SETD1A,KMT2B,SETDB1",PKMTs methylate histone lysines,71

R-HSA-3214842,"KDM5B,H4-16,H3C14,KDM1B,KDM2A,KDM1A,KDM4B,KDM4C,PHF8,KDM6B,JMJD6,H3C15,KDM3B,PHF2,H4C15,KDM4D,KDM3A,KDM5A,H3C13,KDM6A,UTY,KDM7A,KDM5C,KDM5D,H4C9,H3C1,H3C4,H3C3,H3C6,H3C11,H3C8,H3C12,H3C10,H3C2,H4C1,H4C4,H4C6,H4C12,H4C11,H4C3,H4C8,H4C2,H4C5,H4C13,H4C14,ARID5B,KDM2B,RIOX2,H3C7,KDM4A",HDMs demethylate histones,50

R-HSA-3214847,"MCRS1,TADA3,NCOA2,KAT5,TAF6L,RUVBL2,BRD8,MORF4L1,MSL3,WDR5,WDR5,KAT7,SGF29,TADA1,H4-16,H4-16,H3C14,H3C14,H2BU1,H2BU1,ATF2,CREBBP,DR1,EP300,H2AC1,H2AC1,USP22,JADE2,KAT6B,ELP5,BRD1,H2BC1,H2BC1,ZZZ3,KAT2A,ELP4,TAF5L,BRPF3,KANSL1,H2AC8,H2AC8,H2AC7,H2AC7,H2BC5,H2BC5,H2BC3,H2BC3,HCFC1,H2AC21,H2AC21,H3C15,H3C15,MSL1,H2BC18,H2BC18,PAX3,ING4,PHF20,MBIP,ING3,ELP6,KANSL2,ELP3,MSL2,ELP2,MRGBP,H4C15,H4C15,SUPT20H,KANSL3,YEATS2,DMAP1,ENY2,ATXN7L3,KAT14,EP400,RBBP7,ACTB,ATXN7,MEAF6,MEAF6,H3C13,H3C13,TADA2A,TAF9,TAF10,TAF12,VPS72,H2AC19,H2AC19,BRPF1,SAP130,KAT6A,JADE1,EPC1,YEATS4,H4C9,H4C9,TRRAP,H2AC13,H2AC13,H2AC15,H2AC15,H2AC14,H2AC14,H2AC16,H2AC16,H2AC6,H2AC6,H2AC4,H2AC4,H2AC17,H2AC17,H2AC18,H2AC18,H2AC20,H2AC20,H2BC8,H2BC8,H2BC13,H2BC13,H2BC15,H2BC15,H2BC14,H2BC14,H2BC7,H2BC7,H2BC6,H2BC6,H2BC9,H2BC9,H2BC10,H2BC10,H2BC4,H2BC4,H2BC17,H2BC17,H2BC21,H2BC21,H3C1,H3C1,H3C4,H3C4,H3C3,H3C3,H3C6,H3C6,H3C11,H3C11,H3C8,H3C8,H3C12,H3C12,H3C10,H3C10,H3C2,H3C2,H4C1,H4C1,H4C4,H4C4,H4C6,H4C6,H4C12,H4C12,H4C11,H4C11,H4C3,H4C3,H4C8,H4C8,H4C2,H4C2,H4C5,H4C5,H4C13,H4C13,H4C14,H4C14,KAT8,ING5,SUPT3H,OGT,ELP1,HAT1,H2AC12,H2AC12,H2BC12,H2BC12,ACTL6A,RUVBL1,NCOA1,KAT2B,H3C7,H3C7,H2AC11,H2AC11,H2BC11,H2BC11,H2AW,H2AW,TADA2B,CLOCK,MORF4L2,JADE3,SUPT7L",HATs acetylate histones,206

R-HSA-3214858,"CDK4,PRMT3,PRMT5,CARM1,WDR5,H4-16,H3C14,DNMT3A,ARID2,H2AC1,H2AC8,H2AC7,H2AX,H2AZ1,H2AC21,PRMT1,H3C15,JAK2,H2AB1,ACTL6B,PRMT7,PRMT6,PBRM1,COPRS,H4C15,H2AJ,ARID1B,RBBP7,CCND1,RPS2,H3C13,SMARCA2,SMARCA4,SMARCB1,SMARCC1,SMARCC2,SMARCD1,SMARCD2,SMARCD3,SMARCE1,H2AC19,WDR77,ARID1A,H4C9,H2AC13,H2AC15,H2AC14,H2AC16,H2AC6,H2AC4,H2AC17,H2AC18,H2AC20,H3C1,H3C4,H3C3,H3C6,H3C11,H3C8,H3C12,H3C10,H3C2,H4C1,H4C4,H4C6,H4C12,H4C11,H4C3,H4C8,H4C2,H4C5,H4C13,H4C14,H2AC12,ACTL6A,H3C7,H2AC11,H2AW,H2AZ2",RMTs methylate histone arginines,79

R-HSA-3215018,"UBA2,UBA2,SAE1,SAE1,SENP5,RWDD3,SENP1,SENP2,SUMO3,SUMO3,SUMO2,SUMO2,UBE2I,UBE2I,SUMO1",Processing and activation of SUMO,15

R-HSA-3229121,"G6PC,SLC37A4,GAA,GBE1,GYG1,GYS1,GYS2,NHLRC1,PPP1R3C,RPS27A,UBA52,UBB,UBC,EPM2A,GYG2,G6PC3",Glycogen storage diseases,16

R-HSA-3229133,SLC37A4,Glycogen storage disease type Ib (SLC37A4),1

R-HSA-3232118,"CDKN2A,PIAS3,HIC1,MDM2,MITF,PIAS4,SUMO3,SUMO2,SP3,FOXL2,TFAP2A,TFAP2B,TFAP2C,TP53,TP53BP1,UBE2I,UBE2I,SUMO1,SUMO1,PIAS1,PIAS2,MTA1",SUMOylation of transcription factors,22

R-HSA-3232142,"NUP50,NUP42,NUP35,NUP205,NUP210,NUP160,NUP188,NUP62,NUP43,MDM2,NUP88,NUP98,PIAS4,NUP54,PML,NDC1,NUP133,NUP107,RANBP2,TRIM27,SEC13,TPR,UBE2I,SUMO1,VHL,NUP37,NUP85,NUP214,AAAS,SEH1L,RAE1,PIAS1,PIAS2,NUP155,NUP93,NUP58,POM121,NUP153",SUMOylation of ubiquitinylation proteins,38

R-HSA-3238698,"TMED5,WNT16,WNT16,VPS29,WNT4,WNT4,VPS35,PORCN,WNT1,WNT1,WNT2,WNT2,WNT3,WNT3,WNT5A,WNT5A,WNT6,WNT6,WNT7A,WNT7A,WNT7B,WNT7B,WNT8A,WNT8A,WNT8B,WNT8B,WNT10B,WNT10B,WNT11,WNT11,WNT2B,WNT2B,WNT9A,WNT9A,WNT9B,WNT9B,WLS,WNT10A,WNT10A,WNT5B,WNT5B,SNX3,WNT3A,WNT3A,VPS26A",WNT ligand biogenesis and trafficking,45

R-HSA-3247509,"CDK4,PRMT3,SAP18,HMG20B,PRMT5,MCRS1,TADA3,CARM1,NCOA2,KAT5,TAF6L,KDM5B,RUVBL2,BRD8,EHMT2,MORF4L1,MSL3,CHD3,CHD4,WDR5,WDR5,KAT7,PADI2,SGF29,TADA1,H4-16,H4-16,AEBP2,H3C14,H3C14,H2BU1,H2BU1,ATF2,CREBBP,DNMT3A,DR1,ARID2,EP300,MECOM,EZH2,H2AC1,H2AC1,KDM1B,KDM2A,KDM1A,KDM4B,SETD1B,KDM4C,PHF8,KDM6B,RCOR1,JMJD6,USP22,JADE2,SUZ12,KAT6B,PADI4,ELP5,BRD1,H2BC1,H2BC1,BRMS1,ZZZ3,KAT2A,ELP4,TAF5L,BRPF3,KANSL1,GPS2,SETD2,PADI1,H2AC8,H2AC8,H2AC7,H2AC7,H2AX,H2AZ1,H2BC5,H2BC5,H2BC3,H2BC3,HCFC1,HDAC1,HDAC2,H2AC21,H2AC21,PRMT1,H3C15,H3C15,MSL1,PADI6,JAK2,KMT5A,KMT2A,H2BC18,H2BC18,H2AB1,NFKB1,NFKB2,PAX3,KMT5B,ING4,PHF20,PHF21A,ACTL6B,MBIP,PADI3,ARID4B,KDM3B,PHF2,MBD3,BRWD1,PRMT7,ING3,GATAD2A,ELP6,NSD3,KANSL2,ELP3,MSL2,PRMT6,PBRM1,ELP2,MRGBP,COPRS,H4C15,H4C15,SUPT20H,KANSL3,YEATS2,KDM4D,ATF7IP,H2AJ,KDM3A,HDAC8,ASH1L,KMT2E,DMAP1,ENY2,SMYD2,ATXN7L3,PRDM9,KAT14,GATAD2B,ARID1B,MTA3,EP400,KMT2C,ARID4A,KDM5A,RBBP4,RBBP5,RBBP7,CCND1,RELA,REST,ACTB,RPS2,ATXN7,PRDM16,NSD1,SUDS3,SMYD3,MEAF6,MEAF6,H3C13,H3C13,SMARCA2,SMARCA4,SMARCB1,SMARCC1,SMARCC2,SMARCD1,SMARCD2,SMARCD3,SMARCE1,SUV39H1,TADA2A,TAF9,TAF10,TAF12,TBL1X,VPS72,H2AC19,H2AC19,KDM6A,UTY,NSD2,BRPF1,WDR77,SAP130,SAP30L,TBL1XR1,SUV39H2,EHMT1,SETD6,KAT6A,JADE1,EPC1,KMT2D,KDM7A,SETD7,YEATS4,KDM5C,KDM5D,ARID1A,H4C9,H4C9,TRRAP,H2AC13,H2AC13,H2AC15,H2AC15,H2AC14,H2AC14,H2AC16,H2AC16,H2AC6,H2AC6,H2AC4,H2AC4,H2AC17,H2AC17,H2AC18,H2AC18,H2AC20,H2AC20,H2BC8,H2BC8,H2BC13,H2BC13,H2BC15,H2BC15,H2BC14,H2BC14,H2BC7,H2BC7,H2BC6,H2BC6,H2BC9,H2BC9,H2BC10,H2BC10,H2BC4,H2BC4,H2BC17,H2BC17,H2BC21,H2BC21,H3C1,H3C1,H3C4,H3C4,H3C3,H3C3,H3C6,H3C6,H3C11,H3C11,H3C8,H3C8,H3C12,H3C12,H3C10,H3C10,H3C2,H3C2,H4C1,H4C1,H4C4,H4C4,H4C6,H4C6,H4C12,H4C12,H4C11,H4C11,H4C3,H4C3,H4C8,H4C8,H4C2,H4C2,H4C5,H4C5,H4C13,H4C13,H4C14,H4C14,SETDB2,HDAC10,KAT8,ARID5B,SETD3,ING5,DOT1L,SUPT3H,DPY30,KDM2B,OGT,KMT5C,RIOX2,ELP1,HAT1,H2AC12,H2AC12,H2BC12,H2BC12,ACTL6A,ACTL6A,RUVBL1,NCOA1,EED,SAP30,HDAC3,KAT2B,H3C7,H3C7,H2AC11,H2AC11,H2BC11,H2BC11,ASH2L,MTA1,MTA2,H2AW,H2AW,TADA2B,H2AZ2,CLOCK,NCOR1,NCOR2,MORF4L2,KDM4A,SETD1A,KMT2B,JADE3,SETDB1,SUPT7L",Chromatin modifying enzymes,339

R-HSA-3248023,TREX1,Regulation by TREX1,1

R-HSA-3249367,"TBK1,STING1,STAT6",STAT6-mediated induction of chemokines,3

R-HSA-3270619,"TREX1,NLRP4,NLRC3,DTX4,XRCC6,TBK1,STING1,IFI16,IRF3,MRE11,DDX41,PRKDC,XRCC5",IRF3-mediated induction of type I IFN,13

R-HSA-3274531,G6PC,Glycogen storage disease type Ia (G6PC),1

R-HSA-3282872,G6PC3,Severe congenital neutropenia type 4 (G6PC3),1

R-HSA-3295583,"RIPK3,TRPM6,TRPV3,MLKL,MCOLN2,TRPC4AP,TRPM5,TRPM1,TRPV2,TRPM4,TRPM7,MCOLN3,TRPV6,TRPV5,TRPC7,MCOLN1,TRPV4,TRPC1,TRPC3,TRPC4,TRPC5,TRPC6,TRPM2,TRPV1,TRPM8,TRPM3,RIPK1,TRPA1",TRP channels,28

R-HSA-3296197,"HCAR1,HCAR2,HCAR3",Hydroxycarboxylic acid-binding receptors,3

R-HSA-3296469,"MMAA,MMACHC,CBLIF,MMADHC,MMAB,MTR,MTRR,MMUT,CD320,LMBRD1,ABCD4,TCN2,CUBN,AMN",Defects in cobalamin (B12) metabolism,14

R-HSA-3296482,"MMAA,MMACHC,CBLIF,MMADHC,ACACA,HLCS,MMAB,MTR,MTRR,MMUT,PC,PCCA,PCCB,CD320,LMBRD1,MCCC1,ABCD4,MCCC2,BTD,TCN2,CUBN,AMN",Defects in vitamin and cofactor metabolism,22

R-HSA-3299685,"TXNRD2,PRDX3,CYBA,CYBB,GPX6,PRDX5,TXN2,GPX1,GPX2,GPX3,GPX5,GPX7,GSR,GSTP1,ERO1A,NUDT2,AQP8,NCF2,NCF4,ATOX1,GPX8,P4HB,NOX4,PRDX1,ATP7A,CYCS,NCF1,SOD1,SOD1,SOD2,SOD3,SOD3,PRDX2,TXN,TXNRD1,NOX5,CAT,PRDX6,CCS,CCS",Detoxification of Reactive Oxygen Species,40

R-HSA-3301854,"NUP50,NEK6,NUP42,NUP35,NEK7,NUP205,NUP210,NUP160,NUP188,NUP62,NUP43,NUP88,NUP98,NUP54,NDC1,NUP133,NUP107,RANBP2,SEC13,TPR,NUP37,NUP85,NUP214,AAAS,SEH1L,RAE1,CCNB1,CCNB2,NEK9,NUP155,NUP93,NUP58,CDK1,POM121,NUP153",Nuclear Pore Complex (NPC) Disassembly,35

R-HSA-3304347,"SMAD2,SMAD3,SMAD4",Loss of Function of SMAD4 in Cancer,3

R-HSA-3304349,"SMAD2,SMAD3,SMAD4,TGFB1,TGFBR1,TGFBR2,ZFYVE9",Loss of Function of SMAD2/3 in Cancer,7

R-HSA-3304351,"FKBP1A,SMAD2,SMAD3,SMAD4,TGFB1,TGFBR1,TGFBR2,ZFYVE9",Signaling by TGF-beta Receptor Complex in Cancer,8

R-HSA-3304356,"SMAD2,SMAD3,TGFB1,TGFBR1,TGFBR2,ZFYVE9",SMAD2/3 Phosphorylation Motif Mutants in Cancer,6

R-HSA-3311021,"SMAD2,SMAD3,SMAD4",SMAD4 MH2 Domain Mutants in Cancer,3

R-HSA-3315487,"SMAD2,SMAD3,SMAD4",SMAD2/3 MH2 Domain Mutants in Cancer,3

R-HSA-3322077,"GBE1,PGM2L1,GYG1,GYS1,GYS2,NHLRC1,PGM1,PPP1R3C,PGM2,RPS27A,UBA52,UBB,UBC,UGP2,EPM2A,GYG2",Glycogen synthesis,16

R-HSA-3323169,"ACACA,HLCS,PC,PCCA,PCCB,MCCC1,MCCC2,BTD",Defects in biotin (Btn) metabolism,8

R-HSA-3359454,TCN2,Defective TCN2 causes hereditary megaloblastic anemia,1

R-HSA-3359457,CBLIF,Defective GIF causes intrinsic factor deficiency,1

R-HSA-3359458,LMBRD1,Defective LMBRD1 causes methylmalonic aciduria and homocystinuria type cblF,1

R-HSA-3359462,"CBLIF,CUBN,AMN",Defective AMN causes hereditary megaloblastic anemia 1,3

R-HSA-3359463,"CBLIF,CUBN,AMN",Defective CUBN causes hereditary megaloblastic anemia 1,3

R-HSA-3359467,"MTR,MTRR",Defective MTRR causes methylmalonic aciduria and homocystinuria type cblE,2

R-HSA-3359469,"MTR,MTRR",Defective MTR causes methylmalonic aciduria and homocystinuria type cblG,2

R-HSA-3359471,MMAB,Defective MMAB causes methylmalonic aciduria type cblB,1

R-HSA-3359473,"MMACHC,MMADHC",Defective MMADHC causes methylmalonic aciduria and homocystinuria type cblD,2

R-HSA-3359474,MMACHC,Defective MMACHC causes methylmalonic aciduria and homocystinuria type cblC,1

R-HSA-3359475,"MMAA,MMUT",Defective MMAA causes methylmalonic aciduria type cblA,2

R-HSA-3359478,"MMAA,MMUT",Defective MUT causes methylmalonic aciduria mut type,2

R-HSA-3359485,"CD320,TCN2",Defective CD320 causes methylmalonic aciduria,2

R-HSA-3371378,"FAS,FASLG,TRAF2,CASP8,TRADD,RIPK1,TNFSF10,FADD,TNFRSF10B,TNFRSF10A,CFLAR",Regulation by c-FLIP,11

R-HSA-3371453,"DNAJB6,NUP50,HSPH1,NUP42,HSPA12B,NUP35,HSPA4L,NUP205,NUP210,NUP160,SIRT1,NUP188,NUP62,HSPA12A,DNAJC2,GSK3B,HSF1,HSPA1A,HSPA1B,HSPA1L,HSPA2,HSPA4,HSPA5,HSPA6,HSPA8,HSPA9,DNAJB1,NUP43,ATM,NUP88,NUP98,HSPA14,HIKESHI,NUP54,ATR,NDC1,NUP133,MAPK1,MAPK3,NUP107,BAG1,CCAR2,RANBP2,RPA1,RPA2,RPA3,SEC13,ST13,HSPA13,TPR,DNAJC7,YWHAE,NUP37,NUP85,NUP214,AAAS,SEH1L,RAE1,RPS19BP1,MAPKAPK2,BAG5,BAG4,BAG3,BAG2,NUP155,NUP93,NUP58,POM121,NUP153",Regulation of HSF1-mediated heat shock response,69

R-HSA-3371497,"ACTR1A,DNAJA2,TUBA1B,TUBB3,TUBB4A,TUBB4B,DCTN2,DCTN6,PTGES3,STIP1,DCTN3,TUBA3E,TUBA3D,DYNLL2,DCTN1,DYNC1H1,DYNC1I1,DYNC1I2,DYNC1LI2,FKBP4,FKBP5,NR3C1,DNAJA1,HSPA1A,HSPA1B,HSPA1L,HSPA2,HSPA8,HSP90AA1,HSP90AB1,DNAJB1,TUBB8,TUBB2B,AR,NR3C2,DYNC1LI1,DCTN4,TUBA8,PGR,DNAJA4,ACTR10,TUBA4A,TUBA3C,TUBB2A,TUBA1A,TUBAL3,TUBB1,CAPZA1,CAPZA2,CAPZB,DCTN5,TUBB6,TUBA1C,DYNLL1,CAPZA3",HSP90 chaperone cycle for steroid hormone receptors (SHR),55

R-HSA-3371511,"HDAC6,PTGES3,EEF1A1,HSBP1,HSF1,HSP90AA1,HSP90AB1,RPA1,RPA2,RPA3,VCP,YWHAE",HSF1 activation,12

R-HSA-3371556,"HDAC6,DNAJB6,PTGES3,NUP50,HSPH1,NUP42,HSPA12B,NUP35,CREBBP,CRYAB,EEF1A1,EP300,HSPA4L,FKBP4,NUP205,NUP210,NUP160,SIRT1,NUP188,NUP62,MTOR,HSPA12A,HSPB8,DNAJC2,GSK3B,HSBP1,HSF1,HSPA1A,HSPA1B,HSPA1L,HSPA2,HSPA4,HSPA5,HSPA6,HSPA8,HSPA9,HSP90AA1,HSP90AB1,DNAJB1,NUP43,ATM,NUP88,NUP98,HSPA14,HIKESHI,NUP54,ATR,NDC1,NUP133,MAPK1,MAPK3,NUP107,BAG1,RPTOR,CCAR2,RANBP2,RPA1,RPA2,RPA3,SEC13,MLST8,ST13,HSPA13,TPR,DNAJC7,VCP,YWHAE,NUP37,NUP85,NUP214,AAAS,CAMK2A,CAMK2B,CAMK2D,CAMK2G,SEH1L,AKT1S1,RAE1,RPS19BP1,MAPKAPK2,BAG5,BAG4,BAG3,BAG2,NUP155,NUP93,NUP58,POM121,NUP153",Cellular response to heat stress,89

R-HSA-3371568,"PTGES3,CREBBP,EP300,FKBP4,HSBP1,HSF1,HSPA1A,HSPA1B,HSPA1L,HSPA2,HSPA8,HSP90AA1,HSP90AB1,DNAJB1",Attenuation phase,14

R-HSA-3371571,"PTGES3,CREBBP,CRYAB,EP300,FKBP4,MTOR,HSPB8,HSBP1,HSF1,HSPA1A,HSPA1B,HSPA1L,HSPA2,HSPA8,HSP90AA1,HSP90AB1,DNAJB1,RPTOR,MLST8,CAMK2A,CAMK2B,CAMK2D,CAMK2G,AKT1S1",HSF1-dependent transactivation,24

R-HSA-3371598,BTD,Defective BTD causes biotidinase deficiency,1

R-HSA-3371599,"ACACA,HLCS,PC,PCCA,PCCB,MCCC1,MCCC2",Defective HLCS causes multiple carboxylase deficiency,7

R-HSA-349425,"PSME3,PSMD14,PSMB11,PSMA8,PSME4,ATM,PSMA1,PSMA2,PSMA3,PSMA4,PSMA5,PSMA6,PSMA7,PSMB1,PSMB2,PSMB3,PSMB4,PSMB5,PSMB6,PSMB7,PSMB8,PSMB9,PSMB10,PSMC1,PSMC2,PSMC3,PSMC4,PSMC5,PSMC6,PSMD1,PSMD2,PSMD3,PSMD4,PSMD5,PSMD7,PSMD8,PSMD9,PSMD10,PSMD11,PSMD12,PSMD13,PSME1,PSME2,RPS27A,COP1,TP53,UBA52,UBB,UBC,SEM1,PSMF1,PSMD6",Autodegradation of the E3 ubiquitin ligase COP1,52

R-HSA-350054,"HDAC6,HDAC5,MAMLD1,CREBBP,SNW1,KAT2A,HDAC1,HDAC2,RBPJ,NOTCH1,NOTCH2,NOTCH3,NOTCH4,HDAC7,MAML3,HDAC8,TBL1X,TBL1XR1,HDAC11,HDAC10,MAML2,HDAC3,KAT2B,NCOR1,NCOR2,HDAC9,HDAC4,MAML1",Notch-HLH transcription pathway,28

R-HSA-350562,"PSME3,PSMD14,PSMB11,PSMA8,NQO1,PSME4,OAZ1,OAZ2,ODC1,AZIN1,OAZ3,PSMA1,PSMA2,PSMA3,PSMA4,PSMA5,PSMA6,PSMA7,PSMB1,PSMB2,PSMB3,PSMB4,PSMB5,PSMB6,PSMB7,PSMB8,PSMB9,PSMB10,PSMC1,PSMC2,PSMC3,PSMC4,PSMC5,PSMC6,PSMD1,PSMD2,PSMD3,PSMD4,PSMD5,PSMD7,PSMD8,PSMD9,PSMD10,PSMD11,PSMD12,PSMD13,PSME1,PSME2,SEM1,PSMF1,PSMD6",Regulation of ornithine decarboxylase (ODC),51

R-HSA-350864,"DIO1,DIO2,DIO3",Regulation of thyroid hormone activity,3

R-HSA-351143,"AZIN2,AGMAT",Agmatine biosynthesis,2

R-HSA-351200,"PAOX,SMOX,SAT1",Interconversion of polyamines,3

R-HSA-351202,"PSME3,PSMD14,AZIN2,PSMB11,PSMA8,NQO1,PAOX,PSME4,AMD1,OAZ1,OAZ2,ODC1,AZIN1,OAZ3,SMOX,PSMA1,PSMA2,PSMA3,PSMA4,PSMA5,PSMA6,PSMA7,PSMB1,PSMB2,PSMB3,PSMB4,PSMB5,PSMB6,PSMB7,PSMB8,PSMB9,PSMB10,PSMC1,PSMC2,PSMC3,PSMC4,PSMC5,PSMC6,PSMD1,PSMD2,PSMD3,PSMD4,PSMD5,PSMD7,PSMD8,PSMD9,PSMD10,PSMD11,PSMD12,PSMD13,PSME1,PSME2,SAT1,SMS,SRM,SEM1,AGMAT,PSMF1,PSMD6",Metabolism of polyamines,59

R-HSA-351906,"OCLN,CTNNB1,DSG1,DSG2,DSG3,DSP,PKP1,TJP1,CASP3,TJP2,CDH1",Apoptotic cleavage of cell adhesion proteins,11

R-HSA-352230,"SLC38A3,SLC7A9,SLC6A14,SLC16A10,SLC36A4,SLC25A29,SLC43A2,SLC36A2,SLC36A1,SLC7A8,SLC7A11,SLC6A19,SLC6A18,SLC38A2,SLC6A20,SLC38A4,SLC6A15,SLC7A10,SLC1A4,SLC1A5,SLC3A1,SLC3A2,SLC6A6,SLC6A12,SLC7A1,SLC7A2,SLC7A5,SLC38A1,SLC7A3,SLC43A1,SLC7A7,SLC7A6,SLC38A5",Amino acid transport across the plasma membrane,33

R-HSA-352238,"LMNA,LMNB1,CASP6",Breakdown of the nuclear lamina,3

R-HSA-354192,"RASGRP1,RASGRP2,RAPGEF3,RAPGEF4,CRK,CSK,AKT1,FGA,FGB,FGG,FN1,GRB2,ITGA2B,ITGB3,PDPK1,APBB1IP,PTK2,PTPN1,RAP1A,RAP1B,SHC1,SOS1,SRC,SYK,TLN1,VWF,BCAR1",Integrin signaling,27

R-HSA-354194,"FGA,FGB,FGG,FN1,GRB2,ITGA2B,ITGB3,APBB1IP,PTK2,RAP1A,RAP1B,SOS1,SRC,TLN1,VWF",GRB2:SOS provides linkage to MAPK signaling for Integrins ,15

R-HSA-3560782,"GPC6,CSPG5,KERA,B4GALT7,CHST14,B3GALT6,VCAN,NCAN,CSPG4,DCN,ACAN,SLC26A2,EXT1,EXT2,GPC2,GPC4,GPC5,CHSY1,FMOD,B3GAT3,B4GALT1,GPC3,GPC1,HEXA,HEXB,HSPG2,AGRN,LUM,CHST6,OMD,OGN,PRELP,BGN,SDC1,BCAN,SDC2,SDC4,ST3GAL3,PAPSS2,CHST3,SDC3",Diseases associated with glycosaminoglycan metabolism,41

R-HSA-3560783,"GPC6,CSPG5,B4GALT7,VCAN,NCAN,CSPG4,DCN,GPC2,GPC4,GPC5,GPC3,GPC1,HSPG2,AGRN,BGN,SDC1,BCAN,SDC2,SDC4,SDC3","Defective B4GALT7 causes EDS, progeroid type",20

R-HSA-3560792,SLC26A2,Defective SLC26A2 causes chondrodysplasias,1

R-HSA-3560796,PAPSS2,Defective PAPSS2 causes SEMD-PA,1

R-HSA-3560801,"GPC6,CSPG5,VCAN,NCAN,CSPG4,DCN,GPC2,GPC4,GPC5,B3GAT3,GPC3,GPC1,HSPG2,AGRN,BGN,SDC1,BCAN,SDC2,SDC4,SDC3",Defective B3GAT3 causes JDSSDHD,20

R-HSA-3595172,"CSPG5,VCAN,NCAN,CSPG4,DCN,BGN,BCAN,CHST3",Defective CHST3 causes SEDCJD,8

R-HSA-3595174,"CSPG5,CHST14,VCAN,NCAN,CSPG4,DCN,BGN,BCAN","Defective CHST14 causes EDS, musculocontractural type",8

R-HSA-3595177,"CSPG5,VCAN,NCAN,CSPG4,DCN,CHSY1,BGN,BCAN",Defective CHSY1 causes TPBS,8

R-HSA-3642278,"TGFB1,TGFBR1,TGFBR2",Loss of Function of TGFBR2 in Cancer,3

R-HSA-3642279,"TGFB1,TGFBR2",TGFBR2 MSI Frameshift Mutants in Cancer,2

R-HSA-3645790,"TGFB1,TGFBR1,TGFBR2",TGFBR2 Kinase Domain Mutants in Cancer,3

R-HSA-3656225,"KERA,ACAN,FMOD,LUM,CHST6,OMD,OGN,PRELP",Defective CHST6 causes MCDC1,8

R-HSA-3656234,HEXA,Defective HEXA causes GM2G1,1

R-HSA-3656237,"GPC6,EXT1,EXT2,GPC2,GPC4,GPC5,GPC3,GPC1,HSPG2,AGRN,SDC1,SDC2,SDC4,SDC3",Defective EXT2 causes exostoses 2,14

R-HSA-3656243,"KERA,ACAN,FMOD,LUM,OMD,OGN,PRELP,ST3GAL3",Defective ST3GAL3 causes MCT12 and EIEE15,8

R-HSA-3656244,"KERA,ACAN,FMOD,B4GALT1,LUM,OMD,OGN,PRELP",Defective B4GALT1 causes B4GALT1-CDG (CDG-2d),8

R-HSA-3656248,HEXB,Defective HEXB causes GM2G2,1

R-HSA-3656253,"GPC6,EXT1,EXT2,GPC2,GPC4,GPC5,GPC3,GPC1,HSPG2,AGRN,SDC1,SDC2,SDC4,SDC3","Defective EXT1 causes exostoses 1, TRPS2 and CHDS",14

R-HSA-3656532,"SMAD2,SMAD3,TGFB1,TGFBR1,TGFBR2,ZFYVE9",TGFBR1 KD Mutants in Cancer,6

R-HSA-3656534,"FKBP1A,SMAD2,SMAD3,TGFB1,TGFBR1,TGFBR2,ZFYVE9",Loss of Function of TGFBR1 in Cancer,7

R-HSA-3656535,"FKBP1A,TGFB1,TGFBR1,TGFBR2",TGFBR1 LBD Mutants in Cancer,4

R-HSA-3700989,"AKT3,ELOA3D,RAD50,LRPPRC,CDK2,CDK5,CDK7,CDK9,CDKN1A,CDKN1B,CDKN2A,RRAGB,NDRG1,PRMT5,CARM1,KAT5,LAMTOR5,PRELID3A,RRAGA,PLK2,PPP1R13L,EHMT2,YWHAQ,CHD3,TOPBP1,CHD4,CHEK1,SUPT16H,CHEK2,CHM,TP53RK,RMI2,COX20,RFFL,TMEM219,PLK3,COX4I1,COX5B,SETD9,COX6A1,JMY,COX6B1,COX6C,COX7B,COX7C,COX8A,COX11,TAF1L,ATF2,CREBBP,MAPK14,SESN3,E2F7,RAD9B,CSNK2A1,CSNK2A2,CSNK2B,ZNF420,SLC38A9,DAXX,ELOA3,DDB2,GADD45A,DNA2,ARID3A,E2F1,E2F4,AGO3,AGO4,EP300,HIPK1,ERCC2,AKT1,ERCC3,AKT2,FANCC,FANCD2,TPX2,CNOT1,TNRC6B,PPP1R13B,FOS,BRD1,CNOT6L,MTOR,RICTOR,G6PD,PRDX5,CNOT10,NELFB,ZNF385A,L3MBTL1,NOC2L,AGO1,BBC3,BRPF3,AGO2,GLS2,PRELID1,SESN1,TNRC6A,GLS,SFN,GPI,COX18,GPX2,LAMTOR2,RGCC,HIPK2,BRD7,GSR,GTF2F1,GTF2F2,GTF2H1,GTF2H2,GTF2H3,GTF2H4,CNOT7,HDAC1,HDAC2,APAF1,PRMT1,BIRC5,HUS1,IGFBP3,FAS,ING2,JUN,KMT5A,LAMTOR4,GTF2H5,MDM2,MDM4,MLH1,MNAT1,MOV10,MRE11,MSH2,COX1,COX2,COX3,NBN,NDUFA4,ATM,CNOT2,CNOT3,CNOT4,NPM1,RRM2B,PRDX1,PCNA,TACO1,ELOA2,PHF20,COX16,PRKAG2,NELFCD,TRIAP1,TAF9B,PDPK1,CDK12,PIN1,PIP4K2A,PLAGL1,MBD3,PRKAG3,PMAIP1,PML,PMS2,CYCS,POLR2A,POLR2B,POLR2C,POLR2D,POLR2E,POLR2F,POLR2G,POLR2H,POLR2I,POLR2J,POLR2K,POLR2L,TAF7L,ATR,DDIT4,POU4F1,POU4F2,GATAD2A,BANP,LAMTOR1,PPP2CA,PPP2CB,PPP2R1A,PPP2R1B,FANCI,STEAP3,PPP2R5C,PIDD1,CNOT11,PRR5,PRKAA1,PRKAA2,PRKAB1,PRKAB2,PRKAG1,CENPJ,MAPK11,MAP2K6,SMYD2,PCBP4,TIGAR,PTEN,GATAD2B,RPTOR,TNRC6C,BARD1,BAX,RAD1,NLRC4,RRAGD,RABGGTA,RABGGTB,RAD9A,RAD17,RAD51D,RBBP4,RBBP7,RBBP8,RBL1,RBL2,RFC2,RFC3,RFC4,RFC5,RHEB,BCL6,RPA1,RPA2,RPA3,RPS27A,SCO1,BID,PRDM1,TP53AIP1,PERP,BLM,RRAGC,MLST8,SGK1,MEAF6,BNIP3L,BRCA1,SSRP1,AURKA,STK11,SUPT4H1,SUPT5H,SURF1,TAF1,TAF2,TAF4,TAF4B,TAF5,TAF6,TAF7,TAF9,TAF10,TAF11,TAF12,TAF13,TBP,TCEA1,ELOC,ELOB,ELOA,PRDX2,TFDP1,TFDP2,TOP3A,TP53,TP53BP2,TP73,TSC1,TSC2,ELOA3B,TXN,TXNRD1,UBA52,UBB,UBC,NELFA,WRN,YWHAB,YWHAE,YWHAG,YWHAH,YWHAZ,BTG2,BRPF1,USP7,MAPKAP1,NELFE,BCL2L14,E2F8,EHMT1,PIP4K2C,KAT6A,RMI1,RNF34,TAF15,ELL,CASP1,CASP2,SESN2,RHNO1,TAF3,CASP6,PIP4K2B,BRIP1,ATRIP,ING5,CASP10,DYRK2,AIFM2,COX14,TNKS1BP1,MAPKAPK5,CDK13,TP63,LAMTOR3,CRADD,TNFRSF10D,TNFRSF10C,TNFRSF10B,TNFRSF10A,CCNK,CDK5R1,CCNA2,CCNA1,CCNB1,CCNE1,CCNG1,CCNH,CCNT1,CCNT2,PIP4P1,USP2,CNOT9,CCNE2,CTDP1,EXO1,COX7A2L,TTC5,AURKB,MTA2,CNOT8,COX5A,TP53INP1,TP53I3,MDC1,CDK1,NUAK1,CDC25C,SCO2",Transcriptional Regulation by TP53,364

R-HSA-372708,"CRK,FGA,FGB,FGG,FN1,ITGA2B,ITGB3,APBB1IP,PTK2,RAP1A,RAP1B,SRC,TLN1,VWF,BCAR1",p130Cas linkage to MAPK signaling for integrins,15

R-HSA-372790,"AKT3,INSL5,ARHGEF33,CHURC1-FNTB,GPC6,NPY4R2,OPN1MW3,RASGRP1,LPAR6,DHRS9,CDK5,CALCRL,RASGRP2,RAMP2,RAMP1,RAMP3,LOC102723532,NET1,RGS19,RGS19,NMUR1,VAV3,LINC02210-CRHR1,CXCL13,RGS14,RGS14,CAMKK2,CAMKK2,CXCR6,GNA13,GNB5,GNB5,RRH,ADCY1,ADCY1,AHCYL1,OR5I1,ADCY2,ADCY2,CYSLTR1,CCR9,CGA,PDE10A,CCL27,NMU,NPFFR2,PROKR1,GPR83,ADCY3,ADCY3,UTS2,METAP2,EEF1AKMT4-ECE2,AKR1C4,ADCY5,ADCY5,ADCY6,ADCY6,FZD10,AKAP13,GPR176,GPR45,PTGDR2,HRH3,CHRM1,CHRM2,ADCY7,ADCY7,PTH2,CHRM3,CHRM4,CHRM5,MGLL,ADCY8,ADCY8,UCN3,ADCY9,ADCY9,ARHGEF25,ADCYAP1,ADCYAP1R1,RLN3,OR52E2,OR52J3,OR51L1,OR51A7,OR51S1,OR51F2,OR52R1,OR4C46,OR4X2,OR4B1,OR52M1,OR52K2,OR5P2,OR5P3,OR8I2,OR2D3,OR2D2,OR52W1,OR56A4,OR56A1,CLPS,OR2AP1,OR10P1,SDR9C7,OR10AD1,OR10A7,FGD4,RXFP2,OR4K14,OR4L1,OR11H6,GPHB5,CCR1,CCR3,CCR4,CCR5,CCR6,CCR7,CCR8,ACKR2,CMKLR1,LTB4R,OR4D2,CNGB1,CNGA1,OR7D4,OR7G1,OR1M1,PCP2,OR1I1,OR10H4,CNR1,CNR2,OR2M5,OR2M3,OR2T12,OR14C36,OR2T34,OR2T10,OR2T4,OR2T11,OR10J5,OR2B11,ARHGEF19,OR10T2,OR6P1,OR10X1,OR10Z1,OR6K6,OR6N1,PROKR2,NMS,OR9A4,GRK7,RTP1,CORT,ADM,ADORA1,OR2Y1,TAAR9,TAAR1,ADORA2A,OR9A2,OR2A14,OR6B1,OR2F2,ADORA2B,CREB1,CREB1,OR13C5,OR13C8,OR13C3,OR13C4,OR13F1,OR1L8,OR1N2,OR1N1,DGKK,CRH,CRHBP,CRHR1,CRHR2,ADORA3,OR52I2,OR51E1,OR10A5,OR2AG1,RDH12,ADRA1D,PIK3R6,ADRA1B,ADRA1A,ADRA2A,OR6B3,ADRA2B,PLB1,GPBAR1,ADRA2C,CX3CR1,ADRB1,ADRB2,ADRB3,GRK2,GRK3,RDH10,OR1Q1,AWAT2,CD55,DGKA,DGKB,DGKG,DGKH,DGKQ,OR7D2,PLPPR5,AKR1C1,OXER1,GPHA2,DRD1,DRD2,DRD3,DRD4,DRD5,AGT,HBEGF,HBEGF,AGTR1,AGTR2,APLNR,GPR183,ECE1,ECT2,S1PR1,LPAR1,S1PR3,EDN1,EDN2,EDN3,EDNRA,EDNRB,EGFR,OR56B4,ADCY4,ADCY4,ADGRE1,AKT1,AKT2,F2,F2R,F2RL1,F2RL2,OR8U1,OR4C16,OR4C11,OR4S2,OR4C6,OR5D14,OR5L1,OR5D18,OR5AS1,OR8K5,OR5T2,OR8H1,OR8K3,OR8J1,OR5R1,OR5M3,OR5M8,OR5M11,OR5AR1,OR8B12,OR8G5,OR10G8,OR10G9,OR10S1,OR6T1,OR4D5,OR6Q1,OR9I1,OR9Q1,OR9Q2,OR1S2,OR1S1,OR10Q1,OR5B17,OR5B21,OR5A2,OR5A1,OR4D6,OR4D11,REEP3,OPN5,FGD2,GPC2,DAGLB,NAPEPLD,GPRC6A,GPC4,FGD1,GPC5,ARHGEF15,METAP1,ARHGEF9,PLCB1,PLCB1,MCF2L,ARHGEF12,ARHGEF18,FNTA,FNTB,PIK3R5,OR52A1,LPAR3,FPR1,FPR2,FPR3,OPN3,NTSR2,ABCA4,ABCA4,FSHB,FSHR,GAST,ACKR1,FZD2,OR6C74,OR6C3,OR2T6,OR1L4,GABBR1,OR52B2,OR4C3,OR4S1,NPB,UTS2B,NGEF,GALR1,TAS2R39,TAS2R40,TAS2R41,TAS2R43,TAS2R31,TAS2R46,TAS2R30,TAS2R19,TAS2R20,TAS2R50,LRP10,ARHGEF26,GPSM1,ABHD12,RGS22,OR1C1,OR1A2,OR2F1,OR2B6,OR1J4,TIAM2,OR2M4,OR2L2,OR2K2,OR7A17,OR5L2,OR5K1,OR5H1,GCG,OR10J1,OR8G2P,OR8B8,OR8G1,OR10A3,OPN1MW,OR12D2,OR11A1,OR10H3,OR10G3,OR10H2,OR10H1,RGS17,RGS17,OR8B2,OR7E24,OR7C2,OR7A5,OR7C1,OR4F4,OR4F3,OR4E1,OR4D1,OR2W1,OR2V1,OR2T1,OR2J2,OR2H1,OR1L3,OR1L1,OR1J2,GHRH,GHRHR,GHSR,GHSR,GIP,GIPR,NBEA,PDE7B,GPC3,HCAR1,OXGR1,C5AR2,ARHGEF16,P2RY10,GLP1R,GNA11,GNA11,GNA12,GNA15,GNA15,GNAI1,GNAI1,GNAI2,GNAI2,GNAI3,GNAI3,GNAL,GNAO1,GNAO1,GNAQ,GNAQ,GNAS,GNAS,GNAT1,GNAT1,GNAT2,GNAT2,GNAZ,GNAZ,GNB1,GNB1,GNB2,GNB3,GNG3,GNG4,GNG5,GNG7,GNG10,GNG11,GNGT1,GNGT1,GNGT2,GNRH1,GNRH2,GNRHR,GPC1,CCR10,OR51B5,OR10AG1,OR5J2,GPR4,XCR1,OR4C13,OR4C12,NPBWR1,OR52Z1,OR51V1,OR8D1,OR8D2,OR8B4,OR9G4,NPBWR2,OR10A4,CXCR3,OR6C6,PRLHR,OR4N4,UTS2R,GPR15,NPW,GPR17,GPR18,GPR20,OR2Z1,OR10H5,OR2L13,OR14A16,LPAR4,MCHR1,GPR25,GPR27,RTP5,GPER1,GPR31,GPR32,CYP4V2,GPR150,OR2V2,GPR35,GPR37,MLNR,GPR39,OR13C9,OR13D1,FFAR1,FFAR3,FFAR2,GRK4,GRK5,GRK5,GRK6,GRB2,ABR,GRM1,GRM2,GRM3,GRM4,GRM5,GRM6,GRM7,GRM8,CXCL1,CXCL2,CXCL3,GRP,GRPR,GUCA1A,GUCA1B,GUCY2F,GPSM2,GPR132,LRP12,GUCY2D,ANXA1,HCRT,HCRTR1,HCRTR2,ADGRE2,TAAR6,HRAS,HRH1,HRH2,HSD17B1,HSPG2,APOA1,APOA1,HTR1A,HTR1B,HTR1D,HTR1E,HTR1F,HTR2A,HTR2B,HTR2C,APOA2,APOA2,HTR4,HTR5A,HTR6,HTR7,IAPP,APOB,APOB,GPIHBP1,TAS2R60,HCAR2,FFAR4,FFAR4,OR8D4,OR5F1,OR5AP2,OR52L1,OR2AG2,RXFP4,OR52B6,OR2AT4,OR10A2,OR6C2,OR6C4,OR8S1,OR6S1,OR6F1,OR2W3,OR2T8,OR2T3,OR10R2,OR2T29,APOC2,APOC2,RTP2,APOC3,APOC3,OR6V1,OR2A12,OR2A1,GNAT3,GNAT3,QRFP,OR1J1,OR1B1,OR13H1,APOE,APOE,APP,RGSL1,RGSL1,IHH,CXCL8,CXCR1,CXCR2,CXCL10,INSL3,ITPR1,ITPR1,ITPR2,ITPR2,ITPR3,ITPR3,AGRN,KEL,KISS1,KNG1,KPNA2,KRAS,RHOA,NPSR1,OR56B1,RHOB,CCL4L1,RGS9BP,RGS9BP,RHOC,OR6B2,ARHGEF37,OR52K1,OR52I1,OR51D1,OR52A5,OR51B6,OR51M1,OR51Q1,OR51I1,OR51I2,OR52D1,OR52H1,OR52N4,OR52N5,OR52N2,OR52E6,OR52E8,OR52E4,OR56A3,OR56A5,OR10A6,OR4X1,OR5D13,OR5D16,OR5W2,OR8H2,OR8H3,OR5T3,OR5T1,OR8K1,OR5M9,OR5M10,OR5M1,OR9G1,OR5AK2,OR5B2,OR5B12,OR5AN1,OR4D10,OR4D9,OR10V1,OR6X1,OR6M1,OR10G4,OR10G7,OR8B3,OR8A1,OR6C1,OR6C75,OR6C76,OR6C70,OR4N2,OR4K2,OR4K13,OR4K17,OR4N5,OR11G2,OR11H7,OR11H4,OR5AU1,OR4M2,OR4F6,OR4F15,OR7G2,OR7G3,OR7A10,OR10K2,OR10K1,OR6Y1,OR6K3,OR11L1,OR2L8,OR2AK2,OR2L3,OR2M2,OR2T33,OR2M7,OR2G6,OR2A25,OR13J1,OR13C2,OR1L6,OR5C1,OR1K1,OR2A5,LDLR,LHB,LHCGR,OR2A7,OR51H1,OR51T1,OR51A4,OR51A2,OR2T2,OR2T5,OR14I1,OR5K2,LPL,OR2A42,OR2T27,OR2T35,OR4A47,OR5H14,OR5H15,OR5K3,OR5K4,OR6C65,OR6C68,LRP1,LRP2,ARRB1,ARRB2,CCL3L3,MC1R,MC2R,MC3R,MC4R,MC5R,MCF2,CXCL9,MLN,MMP3,MMP3,RGS21,RGS21,OR4F21,OR5B3,OR9K2,OR4Q3,OR4M1,OR10J3,OR2W5,OR13G1,OR2B3,OR2J1,OR2J3,OR14J1,OR10C1,OR2A2,ARHGEF35,MTNR1A,MTNR1B,MYO7A,NMB,NMBR,NMT1,NPY,NPY1R,NPY2R,NPY5R,NRAS,NTS,NTSR1,GPR143,OPRD1,OPRK1,OPRL1,OPRM1,OR1D2,OR1F1,OR2C1,OR3A1,OR3A2,OXT,OXTR,P2RY1,P2RY2,P2RY4,P2RY6,P2RY11,OR8U9,PAK1,ARHGEF4,ARHGEF3,RDH8,TAS2R3,TAS2R4,TAS2R16,TAS2R1,TAS2R9,TAS2R8,TAS2R7,TAS2R13,TAS2R10,TAS2R14,DHH,HEBP1,PDE11A,PRLH,GAL,RDH11,RXFP3,REEP2,PDE1A,PDE1C,PDE2A,WNT16,PDE3A,PDE3B,PDE4A,PDE4B,PDE4C,PDE4D,PDE6A,PDE6A,PDE6G,PDE6G,PDE7A,PDE8A,PDE1B,ACKR4,PDE6B,PDE6B,PDPK1,PDYN,GHRL,GHRL,GNG13,PENK,PF4,PIK3CA,PIK3CG,PIK3R1,PIK3R2,PLA2G4A,PLCB2,PLCB2,PLCB3,PLCB3,PLCB4,PLCB4,BCO1,S1PR5,PLXNB1,PMCH,PNOC,P2RY13,GPR84,PNLIP,GNG2,WNT4,TAS2R5,POMC,PPBP,PPEF1,ARHGEF38,RETSAT,PLPPR1,PPP1CA,AVP,PPP2CA,PPP2CB,ARHGEF10L,PPP2R1A,PPP2R1B,AVPR1A,PPP2R5D,AVPR1B,PPP3CA,PPP3CB,PPP3CC,PPP3R1,PPY,AVPR2,NPY4R,PRKACA,PRKACA,PRKACB,PRKACB,PRKACG,PRKACG,ARHGEF40,PRKAR1A,PRKAR1B,PRKAR2A,PRKAR2A,PRKAR2B,PRKCA,PRKCA,PRKCB,PRKCD,PRKCD,PRKCE,PRKCE,PRKCG,PRKCG,PRKCH,PRKCH,PRKCQ,PRKCQ,APOM,MAPK1,MAPK3,GNG12,MAPK7,PRKX,LTB4R2,CCL28,PSAP,OR2S2,SUCNR1,NMUR2,PYY,ACKR3,AKR1B10,CYSLTR2,TRPC7,LPAR5,PTAFR,TAS2R38,PTCH1,PTGDR,PTGER1,PTGER2,PTGER3,PTGER4,PTGFR,PTGIR,ABHD6,PTH,PTHLH,PLEKHG5,PTH1R,PTH2R,NLN,PREX1,CXCL16,RASGRF2,HRH4,GNB4,RXFP1,RBP1,RBP2,NPS,RBP3,RBP4,RCVRN,RCVRN,RDH5,RGR,RGS1,RGS1,RGS2,RGS3,RGS4,RGS4,RGS7,RGS7,RGS10,RGS10,RGS12,RGS12,RGS13,RGS13,RGS16,RGS16,RHO,RHO,GRK1,GRK1,RLBP1,RLBP1,RLN2,PROK2,ROCK1,OPN1SW,RPE65,RPS6KA1,RPS6KA2,RPS6KA3,BDKRB1,BDKRB2,SAA1,SAG,SAG,SCT,SCTR,CCL1,CCL2,CCL3,CCL3L1,CCL4,CCL5,CCL7,CCL11,CCL13,CCL16,CCL17,CCL19,CCL20,CCL21,CCL22,CCL23,CCL25,CXCL6,CXCL11,CXCL5,XCL1,CX3CL1,SDC1,SDC2,SDC4,CXCL12,GPSM3,NPFFR1,RTP4,STRA6,CXCR5,RGS18,RGS18,ITSN1,SHH,PLPPR2,P2RY12,PLEKHG2,REEP1,SMO,SOS1,SOS2,SRC,SRC,SST,SSTR1,SSTR2,SSTR3,SSTR4,SSTR5,BRS3,XCL2,TAC1,TACR2,TAC3,TACR1,TACR3,TBXA2R,BTK,TIAM1,C3,C3AR1,TRH,TRHR,TRIO,TRPC3,TRPC6,TSHB,TSHR,C5,TTR,C5AR1,OPN1MW2,CCR2,OR4F29,UCN,VAV1,VAV2,VIP,VIPR1,VIPR2,DAGLA,WNT1,WNT2,WNT3,WNT5A,WNT6,WNT7A,WNT7B,WNT8A,WNT8B,WNT10B,WNT11,WNT2B,WNT9A,WNT9B,XK,LRP8,CXCR4,FZD5,REEP5,OR13A1,OR5H6,OR52E1,OR5H2,OR4K5,OR2H2,OR51G1,OR11H2,OR51B4,OR51B2,OR4C5,OR51J1,OR52N1,OR5AL1,OR4F5,OR2A4,OR4K1,CALCA,CALCB,FZD3,CAMKMT,ARHGEF5,CALCR,ADM2,PLPPR3,CALM1,CALM1,WNT10A,REEP4,CALM2,CALM2,CALM3,CALM3,TAS1R2,TAS1R1,OR5AC2,OR11H1,OR4F17,GPR68,OR4K15,OR8J3,OR51G2,OR51E2,OR4P4,OR4C15,OR4A5,OR4A16,OR4A15,OR10W1,OR2AE1,OR4F16,CAMK4,CAMK4,OR6N2,OR6K2,OR2L5,OR2G3,OR2G2,OR2C3,CAMK2A,CAMK2B,OR5V1,OR2B2,CAMK2D,OR12D3,CAMK2G,FZD1,FZD4,FZD6,FZD7,FZD8,FZD9,TAAR8,RTP3,TAS1R3,OR1A1,OR1D5,OR1E1,BCO2,OR1E2,OR1G1,OR3A3,OBSCN,QRFPR,PPP1R1B,PPP1R1B,CAMKK1,CAMKK1,PROK1,MCHR2,CASR,KISS1R,ADGRE3,GPR65,GALR3,RGS5,RGS5,ARHGEF39,PIK3R3,DGKZ,DGKE,DGKD,RGS8,RGS8,OR6A2,RGS20,RGS20,RDH16,NPFF,PDE8B,HSD17B6,PTCH2,AKR1C3,S1PR4,RGS11,RGS11,RGS9,RGS9,GALR2,HCAR3,CCK,CCKAR,APLN,CCKBR,ARHGEF7,WNT3A,FGD3,KALRN,F2RL3,UCN2,CCRL2,TAAR5,ARHGEF1,DGKI,LPAR2,ARHGEF2,SLC24A1,LRAT,DHRS3,GPR37L1,REEP6,TAAR2,GPR55,S1PR2,GLP2R,NMT2,OPN4,GNG8,ARHGEF6,ROCK2,CCL4L2,GABBR2,GUCA1C,RGS6,RGS6,GNA14,GNA14,ARHGEF10,SDC3,ECE2,ADGRE5,ARHGEF11,ARHGEF17,PLPPR4,P2RY14,CDC42",Signaling by GPCR,1276

R-HSA-373076,"INSL5,NPY4R2,OPN1MW3,LPAR6,NMUR1,CXCL13,CXCR6,RRH,CYSLTR1,CCR9,CGA,CCL27,NMU,NPFFR2,PROKR1,UTS2,EEF1AKMT4-ECE2,PTGDR2,HRH3,CHRM1,CHRM2,CHRM3,CHRM4,CHRM5,RLN3,RXFP2,GPHB5,CCR1,CCR3,CCR4,CCR5,CCR6,CCR7,CCR8,ACKR2,CMKLR1,LTB4R,CNR1,CNR2,PROKR2,NMS,CORT,ADORA1,TAAR9,TAAR1,ADORA2A,ADORA2B,ADORA3,ADRA1D,ADRA1B,ADRA1A,ADRA2A,ADRA2B,GPBAR1,ADRA2C,CX3CR1,ADRB1,ADRB2,ADRB3,PLPPR5,OXER1,GPHA2,DRD1,DRD2,DRD3,DRD4,DRD5,AGT,AGTR1,AGTR2,APLNR,GPR183,ECE1,S1PR1,LPAR1,S1PR3,EDN1,EDN2,EDN3,EDNRA,EDNRB,F2,F2R,F2RL1,F2RL2,OPN5,LPAR3,FPR1,FPR2,FPR3,OPN3,NTSR2,FSHB,FSHR,ACKR1,NPB,UTS2B,GALR1,OPN1MW,GHSR,HCAR1,OXGR1,C5AR2,P2RY10,GNRH1,GNRH2,GNRHR,CCR10,GPR4,XCR1,NPBWR1,NPBWR2,CXCR3,PRLHR,UTS2R,NPW,GPR17,GPR18,LPAR4,MCHR1,GPER1,GPR31,GPR35,GPR37,MLNR,GPR39,FFAR1,FFAR3,FFAR2,CXCL1,CXCL2,CXCL3,GRP,GRPR,GPR132,ANXA1,HCRT,HCRTR1,HCRTR2,TAAR6,HRH1,HRH2,HTR1A,HTR1B,HTR1D,HTR1E,HTR1F,HTR2A,HTR2B,HTR2C,HTR4,HTR5A,HTR6,HTR7,HCAR2,FFAR4,RXFP4,QRFP,APP,CXCL8,CXCR1,CXCR2,CXCL10,INSL3,KEL,KISS1,KNG1,NPSR1,CCL4L1,LHB,LHCGR,CCL3L3,MC1R,MC2R,MC3R,MC4R,MC5R,CXCL9,MLN,MTNR1A,MTNR1B,NMB,NMBR,NPY,NPY1R,NPY2R,NPY5R,NTS,NTSR1,GPR143,OPRD1,OPRK1,OPRL1,OPRM1,OXT,OXTR,P2RY1,P2RY2,P2RY4,P2RY6,P2RY11,HEBP1,PRLH,GAL,RXFP3,ACKR4,PDYN,GHRL,PENK,PF4,S1PR5,PMCH,PNOC,P2RY13,POMC,PPBP,PLPPR1,AVP,AVPR1A,AVPR1B,PPY,AVPR2,NPY4R,LTB4R2,CCL28,PSAP,SUCNR1,NMUR2,PYY,ACKR3,CYSLTR2,LPAR5,PTAFR,PTGDR,PTGER1,PTGER2,PTGER3,PTGER4,PTGFR,PTGIR,NLN,CXCL16,HRH4,RXFP1,NPS,RGR,RHO,RLN2,PROK2,OPN1SW,BDKRB1,BDKRB2,SAA1,CCL1,CCL2,CCL3,CCL3L1,CCL4,CCL5,CCL7,CCL11,CCL13,CCL16,CCL17,CCL19,CCL20,CCL21,CCL22,CCL23,CCL25,CXCL6,CXCL11,CXCL5,XCL1,CX3CL1,CXCL12,NPFFR1,CXCR5,PLPPR2,P2RY12,SST,SSTR1,SSTR2,SSTR3,SSTR4,SSTR5,BRS3,XCL2,TAC1,TACR2,TAC3,TACR1,TACR3,TBXA2R,C3,C3AR1,TRH,TRHR,TSHB,TSHR,C5,C5AR1,OPN1MW2,CCR2,XK,CXCR4,PLPPR3,GPR68,TAAR8,QRFPR,PROK1,MCHR2,KISS1R,GPR65,GALR3,NPFF,S1PR4,GALR2,HCAR3,CCK,CCKAR,APLN,CCKBR,F2RL3,CCRL2,TAAR5,LPAR2,GPR37L1,TAAR2,GPR55,S1PR2,OPN4,ECE2,PLPPR4,P2RY14",Class A/1 (Rhodopsin-like receptors),335

R-HSA-373080,"CALCRL,RAMP2,RAMP1,RAMP3,LINC02210-CRHR1,GNB5,FZD10,PTH2,UCN3,ADCYAP1,ADCYAP1R1,ADM,CRH,CRHBP,CRHR1,CRHR2,CD55,ADGRE1,FZD2,GCG,GHRH,GHRHR,GIP,GIPR,GLP1R,GNAS,GNB1,GNB2,GNB3,GNG3,GNG4,GNG5,GNG7,GNG10,GNG11,GNGT1,GNGT2,ADGRE2,IAPP,IHH,DHH,WNT16,GNG13,GNG2,WNT4,GNG12,CYSLTR2,PTCH1,PTH,PTHLH,PTH1R,PTH2R,GNB4,SCT,SCTR,SHH,SMO,UCN,VIP,VIPR1,VIPR2,WNT1,WNT2,WNT3,WNT5A,WNT6,WNT7A,WNT7B,WNT8A,WNT8B,WNT10B,WNT11,WNT2B,WNT9A,WNT9B,FZD5,CALCA,CALCB,FZD3,CALCR,ADM2,WNT10A,FZD1,FZD4,FZD6,FZD7,FZD8,FZD9,ADGRE3,PTCH2,WNT3A,UCN2,GLP2R,GNG8,ADGRE5",Class B/2 (Secretin family receptors),95

R-HSA-373752,"AGAP2,UNC5D,MAPK14,HJV,DCC,DCC,DOCK1,DOCK1,DSCAM,DSCAM,UNC5B,ABLIM3,FYN,FYN,RGMB,ABLIM1,MYO10,NCK1,NCK1,NEO1,PAK1,PITPNA,PLCG1,PRKCQ,MAPK8,MAPK11,MAPK13,RGMA,TRPC7,DSCAML1,PTK2,PTK2,PTPN11,RAC1,RAC1,NTN4,ROBO1,MAPK12,SIAH1,SIAH2,SLIT1,SLIT3,SRC,SRC,TRIO,TRIO,TRPC1,TRPC3,TRPC4,TRPC5,TRPC6,EZR,ABLIM2,UNC5C,WASL,UNC5A,UNC5A,SLIT2,NTN1,NTN1,CDC42",Netrin-1 signaling,61

R-HSA-373753,"CD2AP,FYN,FYN,NCK1,NPHS1,NPHS1,PIK3CA,PIK3CB,PIK3R1,PIK3R2,KIRREL1,KIRREL1,SPTAN1,SPTBN1,NPHS2,ACTN4,KIRREL2,NCK2,KIRREL3,CASK,ACTN1,ACTN2,IQGAP1,ACTN3,WASL,MAGI2",Nephrin family interactions,26

R-HSA-373755,"PLXNC1,CDK5,CDK5,SEMA3A,SEMA3A,MYL12B,MYL9,SEMA4D,DPYSL4,MYL12A,CFL1,CRMP1,DPYSL2,DPYSL3,ERBB2,FES,FES,PLXND1,ARHGEF12,PIP5K1C,FYN,FYN,RND1,RND1,ARHGAP35,GSK3B,HSP90AA1,HSP90AB1,ITGA1,ITGB1,RHOA,RHOA,RHOB,RHOB,RHOC,RHOC,LIMK1,LIMK1,LIMK2,MET,MYH9,MYH10,MYH11,MYL6,PAK1,PAK2,PAK3,PLXNA1,PLXNA1,PLXNA2,PLXNA2,PLXNB1,PLXNB3,TREM2,PLXNA3,PLXNA3,DPYSL5,SEMA6A,PTPRC,RAC1,RAC1,ROCK1,ROCK1,RRAS,RRAS,SEMA4A,TLN1,TYROBP,MYH14,SEMA6D,SEMA7A,SEMA7A,NRP1,NRP1,CDK5R1,CDK5R1,SEMA5A,PLXNA4,PLXNA4,ROCK2,ROCK2,CD72,SEMA3E,ARHGEF11,FARP2,FARP2",Semaphorin interactions,86

R-HSA-373756,"SDK1,SDK2",SDK interactions,2

R-HSA-373760,"CD24,RANBP9,TUBA1B,TUBA1B,TUBB3,TUBB3,TUBB4A,TUBB4A,TUBB4B,TUBB4B,CHL1,TUBA3E,TUBA3E,SCN11A,LYPLA2,TUBA3D,TUBA3D,AP2M1,AP2M1,AP2S1,AP2S1,CLTA,CLTA,CLTC,CLTC,CNTN1,CSNK2A1,CSNK2A2,CSNK2B,NCAN,AP2A1,AP2A1,AP2A2,AP2A2,AP2B1,AP2B1,DCX,DLG1,DLG3,DLG4,DNM1,DNM2,DPYSL2,EGFR,EPHB2,ALCAM,FGFR1,NFASC,NFASC,KIF4A,KIF4A,GAP43,DNM3,CNTN6,RPS6KA6,LAMA1,KIF4B,KIF4B,ANK1,ANK1,ANK2,ANK2,ANK3,ANK3,HSPA8,TUBB8,TUBB8,TUBB2B,TUBB2B,ITGA1,ITGA2,ITGA2B,ITGA5,ITGA9,ITGAV,ITGB1,ITGB1,ITGB3,KCNQ2,KCNQ3,L1CAM,L1CAM,LAMB1,LAMC1,MSN,MSN,NCAM1,NRCAM,NRCAM,PAK1,SPTBN5,SPTBN5,TUBA8,TUBA8,SCN3B,MAPK1,MAPK3,MAP2K1,MAP2K2,SHTN1,SPTBN4,SPTBN4,RAC1,RDX,RDX,ACTB,ACTB,RPS6KA1,RPS6KA2,RPS6KA3,SCN1A,SCN1B,SCN2A,SCN2B,SCN3A,SCN4A,SCN4B,SCN5A,SCN7A,SCN8A,SCN9A,SCN10A,SDCBP,SH3GL2,SH3GL2,SPTA1,SPTA1,SPTAN1,SPTAN1,SPTB,SPTB,SPTBN1,SPTBN1,SPTBN2,SPTBN2,SRC,CNTN2,ACTG1,ACTG1,TUBA4A,TUBA4A,TUBA3C,TUBA3C,TUBB2A,TUBB2A,VAV2,EZR,EZR,TUBA1A,TUBA1A,TUBAL3,TUBAL3,TUBB1,TUBB1,TUBB6,TUBB6,TUBA1C,TUBA1C,CNTNAP1,ITGA10,NUMB,NRP2,NRP1,NRP1,RPS6KA4,RPS6KA5",L1CAM interactions,166

R-HSA-375165,"COL2A1,COL3A1,COL4A1,COL4A2,COL4A3,COL4A4,COL4A5,COL5A1,COL5A2,COL6A1,COL6A2,COL6A3,COL9A1,COL9A2,COL9A3,COL6A6,CREB1,NCAN,FGFR1,FYN,FYN,COL6A5,GDNF,GFRA1,GFRA2,GRB2,HRAS,AGRN,KRAS,NCAM1,NCAM1,NRAS,NRTN,COL5A3,SPTBN5,SPTBN5,MAPK1,MAPK3,PRNP,PSPN,PTK2,PTK2,SPTBN4,SPTBN4,PTPRA,PTPRA,GFRA4,SOS1,SPTA1,SPTA1,SPTAN1,SPTAN1,SPTB,SPTB,SPTBN1,SPTBN1,SPTBN2,SPTBN2,SRC,CNTN2,CACNA1C,CACNA1D,CACNA1S,CACNB1,CACNB2,CACNB3,CACNB4,ST8SIA4,ST8SIA2,CACNA1I,CACNA1H,CACNA1G,ARTN,RPS6KA5",NCAM signaling for neurite out-growth,74

R-HSA-375276,"INSL5,NPY4R2,NMUR1,CXCL13,CXCR6,CCR9,CCL27,NMU,NPFFR2,PROKR1,UTS2,EEF1AKMT4-ECE2,RLN3,RXFP2,CCR1,CCR3,CCR4,CCR5,CCR6,CCR7,CCR8,ACKR2,PROKR2,NMS,CORT,CX3CR1,AGT,AGTR1,AGTR2,APLNR,ECE1,EDN1,EDN2,EDN3,EDNRA,EDNRB,F2,F2R,F2RL1,F2RL2,FPR1,FPR2,FPR3,NTSR2,ACKR1,NPB,UTS2B,GALR1,GHSR,C5AR2,CCR10,XCR1,NPBWR1,NPBWR2,CXCR3,PRLHR,UTS2R,NPW,MCHR1,GPER1,GPR37,MLNR,CXCL1,CXCL2,CXCL3,GRP,GRPR,ANXA1,HCRT,HCRTR1,HCRTR2,RXFP4,QRFP,APP,CXCL8,CXCR1,CXCR2,CXCL10,INSL3,KEL,KISS1,KNG1,NPSR1,CCL4L1,CCL3L3,MC1R,MC2R,MC3R,MC4R,MC5R,CXCL9,MLN,NMB,NMBR,NPY,NPY1R,NPY2R,NPY5R,NTS,NTSR1,OPRD1,OPRK1,OPRL1,OPRM1,OXT,OXTR,HEBP1,PRLH,GAL,RXFP3,ACKR4,PDYN,GHRL,PENK,PF4,PMCH,PNOC,POMC,PPBP,AVP,AVPR1A,AVPR1B,PPY,AVPR2,NPY4R,CCL28,PSAP,NMUR2,PYY,ACKR3,NLN,CXCL16,RXFP1,NPS,RLN2,PROK2,BDKRB1,BDKRB2,SAA1,CCL1,CCL2,CCL3,CCL3L1,CCL4,CCL5,CCL7,CCL11,CCL13,CCL16,CCL17,CCL19,CCL20,CCL21,CCL22,CCL23,CCL25,CXCL6,CXCL11,CXCL5,XCL1,CX3CL1,CXCL12,NPFFR1,CXCR5,SST,SSTR1,SSTR2,SSTR3,SSTR4,SSTR5,BRS3,XCL2,TAC1,TACR2,TAC3,TACR1,TACR3,C3,C3AR1,TRH,TRHR,C5,C5AR1,CCR2,XK,CXCR4,QRFPR,PROK1,MCHR2,KISS1R,GALR3,NPFF,GALR2,CCK,CCKAR,APLN,CCKBR,F2RL3,CCRL2,GPR37L1,ECE2",Peptide ligand-binding receptors,201

R-HSA-375280,"HRH3,CHRM1,CHRM2,CHRM3,CHRM4,CHRM5,TAAR9,TAAR1,ADRA1D,ADRA1B,ADRA1A,ADRA2A,ADRA2B,ADRA2C,ADRB1,ADRB2,ADRB3,DRD1,DRD2,DRD3,DRD4,DRD5,TAAR6,HRH1,HRH2,HTR1A,HTR1B,HTR1D,HTR1E,HTR1F,HTR2A,HTR2B,HTR2C,HTR4,HTR5A,HTR6,HTR7,GPR143,HRH4,TAAR8,TAAR5,TAAR2",Amine ligand-binding receptors,42

R-HSA-375281,"CGA,GPHB5,GPHA2,FSHB,FSHR,GNRH1,GNRH2,GNRHR,LHB,LHCGR,TSHB,TSHR",Hormone ligand-binding receptors,12

R-HSA-376172,"MAPK14,DCC,DSCAM,DSCAM,PAK1,MAPK8,MAPK11,MAPK13,DSCAML1,RAC1,MAPK12,NTN1",DSCAM interactions,12

R-HSA-376176,"PSME3,PSMD14,PAK4,CAP2,CAP1,BUB1B-PAK6,RNPS1,RPL35,RPL39L,PSMB11,COL4A5,RPS4Y2,RPL10L,PSMA8,DAG1,DCC,EIF4G1,RPL22L1,ETF1,CASC3,NCBP2,USP33,ZSWIM8,CLASP2,PSME4,CLASP1,SRGAP2,RPL13A,GSPT2,FLRT3,ABL1,ABL1,RPL36,UPF2,PABPC1,ABL2,ABL2,GPC1,GSPT1,HOXA2,ISL1,RHOA,RPSA,MAGOH,MSI1,MYO9B,NCBP1,NCK1,RPL10A,NELL2,PAK1,PAK2,PAK3,RPS27L,RPL26L1,EVL,EVL,PFN1,PFN2,MAGOHB,PPP3CB,PRKACA,PRKACB,PRKACG,ENAH,ENAH,PRKAR2A,PRKCA,PRKCA,PSMA1,PSMA2,PSMA3,PSMA4,PSMA5,PSMA6,PSMA7,PSMB1,PSMB2,PSMB3,PSMB4,PAK6,PSMB5,PSMB6,PSMB7,LHX9,PSMB8,PSMB9,PSMB10,PSMC1,PSMC2,PSMC3,PSMC4,PSMC5,PSMC6,PSMD1,PSMD2,PSMD3,PSMD4,PSMD5,PSMD7,PSMD8,PAK5,PSMD9,PSMD10,PSMD11,PSMD12,PSMD13,PSME1,PSME2,SRGAP1,RAC1,ROBO1,ROBO1,ROBO2,ROBO2,RPL3,RPL3L,RPL4,RPL5,RPL6,RPL7,RPL7A,RPL8,RPL9,RPL10,RPL11,RPL12,RPL13,RPL15,RPL17,RPL18,RPL18A,RPL19,RPL21,RPL22,RPL23A,RPL24,RPL26,RPL27,RPL30,RPL27A,RPL28,RPL29,RPL31,RPL32,RPL34,RPL35A,RPL36AL,RPL37,RPL37A,RPL38,RPL39,RPL41,RPL36A,RPLP0,RPLP1,RPLP2,RPS2,RPS3,RPS3A,RPS4X,RPS4Y1,RPS5,RPS6,RPS7,RPS8,RPS9,RPS10,RPS11,RPS12,RPS13,RPS14,RPS15,RPS15A,RPS16,RPS17,RPS18,RPS19,RPS20,RPS21,RPS23,RPS24,RPS25,RPS26,RPS27,RPS27A,RPS27A,RPS28,RPS29,CXCL12,ROBO3,ROBO3,UPF3B,UPF3A,SLIT1,SLIT3,SLIT3,SOS1,SOS2,SRC,ELOC,ELOB,UBA52,UBA52,UBB,UBB,UBC,UBC,VASP,VASP,CXCR4,SEM1,LHX3,ARHGAP39,NCK2,CUL2,NRP1,LDB1,LHX4,RPL14,RPL23,SLIT2,SLIT2,LHX2,NTN1,PSMF1,AKAP5,AKAP5,EIF4A3,PSMD6,SRGAP3,RBM8A,RBX1,CDC42",Signaling by ROBO receptors,234

R-HSA-3769402,"CTBP1,CTNNB1,AKT1,AKT2,CBY1,PYGO1,BCL9L,HDAC1,APC,XIAP,MEN1,LEF1,SOX6,CTNNBIP1,CHD8,RBBP5,BCL9,RPS27A,SOX17,SOX2,SOX3,SOX4,SOX9,SRY,TCF7,TCF7L2,TLE1,TLE2,TLE3,TLE4,UBA52,UBB,UBC,XPO1,YWHAZ,KMT2D,TCF7L1,SOX7,BTRC,ASH2L,PYGO2,SOX13",Deactivation of the beta-catenin transactivating complex,42

R-HSA-3772470,"WIF1,DKK1,DKK4,DKK2,LRP6,LRP5,SOST,WNT4,SFRP1,SFRP2,WNT5A,WNT9A,KREMEN2,KREMEN1,WNT3A",Negative regulation of TCF-dependent signaling by WNT ligand antagonists,15

R-HSA-3781860,"ALG3,MAN1B1,DPAGT1,ALG14,B4GALT1,ALG6,MGAT2,ALG11,ALG1,MOGS,ALG8,ALG12,ALG9,ALG13,ALG2,RFT1,MPDU1",Diseases associated with N-glycosylation of proteins,17

R-HSA-3781865,"GNE,MUC12,GPC6,ALG3,SPON2,SPON1,POMT1,CSPG5,B4GAT1,KERA,ADAMTS13,ADAMTS8,ADAMTS5,ADAMTS7,ADAMTS6,MAN1B1,B4GALT7,CHST14,NUS1,MUCL1,B3GALT6,MUC17,ADAMTS14,MUC15,B3GLCT,VCAN,NCAN,CSPG4,SBSPON,DAG1,DCN,ADAMTS15,ADAMTS16,ADAMTS17,ADAMTS18,ADAMTS19,ACAN,DPAGT1,SLC26A2,ALG14,MUC20,EXT1,EXT2,GPC2,THSD7A,GPC4,GPC5,DOLK,CHSY1,FMOD,GALE,GALK1,GALNT3,GALT,B3GAT3,GFPT1,B4GALT1,GPC3,GLB1,GPC1,MUC19,C1GALT1C1,ALG6,POMT2,HEXA,HEXB,HSPG2,ADAMTSL5,AGRN,MUC21,LFNG,LUM,CHST6,MGAT2,MPI,ALG11,MUC1,MUC3A,MUC4,MUC5AC,MUC6,MUC7,NEU1,NOTCH1,NOTCH2,NOTCH3,NOTCH4,OMD,OGN,CFP,PGM1,PMM2,DPM3,SEMA5B,ADAMTSL4,CTSA,PRELP,POMGNT1,THSD1,ALG1,MUC13,C1GALT1,ADAMTS9,ADAMTSL3,BGN,SDC1,BCAN,SDC2,SDC4,ST3GAL3,THBS1,THBS2,MUC5B,MOGS,ALG8,ALG12,SRD5A3,GALNT12,ALG9,ALG13,THSD4,DHDDS,ADAMTS20,THSD7B,ADAMTS12,ADAMTS10,ALG2,DPM1,DPM2,SEMA5A,PAPSS2,RFT1,LARGE1,ADAMTSL1,MUC16,CHST3,ADAMTS4,ADAMTS3,ADAMTS2,ADAMTS1,MPDU1,SDC3,ADAMTSL2",Diseases of glycosylation,143

R-HSA-3785653,"GYG1,GYS1,NHLRC1,PPP1R3C,RPS27A,UBA52,UBB,UBC,EPM2A",Myoclonic epilepsy of Lafora,9

R-HSA-379397,"COMT,LRTOMT,MAOA",Enzymatic degradation of dopamine by COMT,3

R-HSA-379398,"COMT,MAOA",Enzymatic degradation of Dopamine by monoamine oxidase,2

R-HSA-379401,"COMT,LRTOMT,MAOA,SLC6A3",Dopamine clearance from the synaptic cleft,4

R-HSA-379716,"FARSB,AARS1,DARS1,EPRS1,FARSA,GARS1,HARS1,IARS1,KARS1,MARS1,NARS1,LARS1,PPA1,QARS1,RARS1,SARS1,TARS1,VARS1,WARS1,AIMP2,CARS1,YARS1,AIMP1,EEF1E1",Cytosolic tRNA aminoacylation,24

R-HSA-379724,"FARSB,WARS2,FARS2,EARS2,AARS1,DARS1,EPRS1,FARSA,LARS2,HARS2,PARS2,GARS1,PPA2,HARS1,IARS1,KARS1,MARS1,NARS1,YARS2,LARS1,PPA1,SARS2,DARS2,IARS2,RARS2,VARS2,AARS2,QARS1,RARS1,SARS1,TARS1,VARS1,WARS1,CARS2,AIMP2,NARS2,TARS2,CARS1,YARS1,AIMP1,MARS2,EEF1E1",tRNA Aminoacylation,42

R-HSA-379726,"WARS2,FARS2,EARS2,LARS2,HARS2,PARS2,GARS1,PPA2,KARS1,YARS2,SARS2,DARS2,IARS2,RARS2,VARS2,AARS2,QARS1,CARS2,NARS2,TARS2,MARS2",Mitochondrial tRNA aminoacylation,21

R-HSA-380095,"TAC1,TACR2,TAC3,TACR1,TACR3",Tachykinin receptors bind tachykinins,5

R-HSA-380108,"CXCL13,CXCR6,CCR9,CCL27,CCR1,CCR3,CCR4,CCR5,CCR6,CCR7,CCR8,ACKR2,CX3CR1,CCR10,XCR1,CXCR3,CXCL1,CXCL2,CXCL3,CXCL8,CXCR1,CXCR2,CXCL10,CCL4L1,CCL3L3,CXCL9,ACKR4,PF4,PPBP,CCL28,ACKR3,CXCL16,CCL1,CCL2,CCL3,CCL3L1,CCL4,CCL5,CCL7,CCL11,CCL13,CCL16,CCL17,CCL19,CCL20,CCL21,CCL22,CCL25,CXCL6,CXCL11,CXCL5,XCL1,CX3CL1,CXCL12,CXCR5,XCL2,CCR2,CXCR4,CCRL2",Chemokine receptors bind chemokines,59

R-HSA-380259,"ACTR1A,AKAP9,TPTEP2-CSNK1E,TUBB4A,TUBB4B,DCTN2,CETN2,PLK4,SDCCAG8,CNTRL,FGFR1OP,CEP250,DCTN3,HAUS1,NEDD1,CSNK1D,CSNK1E,DCTN1,DYNC1H1,DYNC1I2,TUBB,CEP164,MAPRE1,NINL,CEP131,CEP152,CLASP1,HAUS5,HSP90AA1,NEK2,ODF2,PAFAH1B1,PCM1,PCNT,PLK1,HAUS6,NDE1,HAUS4,CEP192,HAUS2,PPP2R1A,HAUS7,PRKACA,CEP72,CDK5RAP2,PRKAR2B,CENPJ,TUBA4A,TUBG1,YWHAE,YWHAG,ALMS1,TUBA1A,HAUS3,CEP76,CEP290,CEP63,CEP70,CEP78,OFD1,SSNA1,DYNLL1,HAUS8,CEP41,CEP135,CEP57,CCP110,CKAP5,SFI1,CDK1",Loss of Nlp from mitotic centrosomes,70

R-HSA-380270,"ACTR1A,AKAP9,TPTEP2-CSNK1E,TUBB4A,TUBB4B,TUBGCP3,DCTN2,CETN2,PLK4,SDCCAG8,TUBGCP2,CNTRL,FGFR1OP,CEP250,DCTN3,TUBGCP5,HAUS1,NEDD1,CSNK1D,CSNK1E,DCTN1,DYNC1H1,DYNC1I2,TUBB,CEP164,MAPRE1,NINL,CEP131,CEP152,CLASP1,HAUS5,TUBG2,TUBGCP4,NME7,HSP90AA1,MZT1,NEK2,ODF2,PAFAH1B1,PCM1,PCNT,PLK1,HAUS6,NDE1,HAUS4,CEP192,HAUS2,PPP2R1A,HAUS7,PRKACA,CEP72,CDK5RAP2,PRKAR2B,CENPJ,MZT2A,TUBA4A,TUBG1,CDK11A,YWHAE,YWHAG,ALMS1,TUBA1A,HAUS3,CEP76,MZT2B,CEP290,CEP63,CEP70,CEP78,OFD1,TUBGCP6,SSNA1,DYNLL1,HAUS8,CEP41,CEP135,CEP57,CCP110,CKAP5,SFI1,CDK1,CDK11B",Recruitment of mitotic centrosome proteins and complexes,82

R-HSA-380284,"ACTR1A,AKAP9,TPTEP2-CSNK1E,TUBB4A,TUBB4B,DCTN2,CETN2,PLK4,SDCCAG8,CNTRL,FGFR1OP,CEP250,DCTN3,HAUS1,NEDD1,CSNK1D,CSNK1E,DCTN1,DYNC1H1,DYNC1I2,TUBB,CEP164,MAPRE1,NINL,CEP131,CEP152,CLASP1,HAUS5,HSP90AA1,NEK2,ODF2,PAFAH1B1,PCM1,PCNT,PLK1,HAUS6,NDE1,HAUS4,CEP192,HAUS2,PPP2R1A,HAUS7,PRKACA,CEP72,CDK5RAP2,PRKAR2B,CENPJ,TUBA4A,TUBG1,YWHAE,YWHAG,ALMS1,TUBA1A,HAUS3,CEP76,CEP290,CEP63,CEP70,CEP78,OFD1,SSNA1,DYNLL1,HAUS8,CEP41,CEP135,CEP57,CCP110,CKAP5,SFI1,CDK1",Loss of proteins required for interphase microtubule organization from the centrosome,70

R-HSA-380287,"ACTR1A,AKAP9,TPTEP2-CSNK1E,TUBB4A,TUBB4B,TUBGCP3,DCTN2,CETN2,PLK4,SDCCAG8,TUBGCP2,CNTRL,FGFR1OP,CEP250,DCTN3,TUBGCP5,HAUS1,NEDD1,CSNK1D,CSNK1E,DCTN1,DYNC1H1,DYNC1I2,TUBB,CEP164,MAPRE1,NINL,CEP131,CEP152,CLASP1,HAUS5,TUBG2,TUBGCP4,NME7,HSP90AA1,MZT1,NEK2,ODF2,PAFAH1B1,PCM1,PCNT,PLK1,HAUS6,NDE1,HAUS4,CEP192,HAUS2,PPP2R1A,HAUS7,PRKACA,CEP72,CDK5RAP2,PRKAR2B,CENPJ,MZT2A,TUBA4A,TUBG1,CDK11A,YWHAE,YWHAG,ALMS1,TUBA1A,HAUS3,CEP76,MZT2B,CEP290,CEP63,CEP70,CEP78,OFD1,TUBGCP6,SSNA1,DYNLL1,HAUS8,CEP41,CEP135,CEP57,CCP110,CKAP5,SFI1,CDK1,CDK11B",Centrosome maturation,82

R-HSA-380320,"ACTR1A,AKAP9,TPTEP2-CSNK1E,TUBA1B,TUBB3,TUBB4A,TUBB4B,TUBGCP3,DCTN2,CETN2,PLK4,SDCCAG8,TUBGCP2,CNTRL,FGFR1OP,CEP250,DCTN3,TUBA3E,TUBA3D,TUBGCP5,HAUS1,NEDD1,CSNK1D,CSNK1E,DCTN1,DYNC1H1,DYNC1I2,TUBB,CEP164,MAPRE1,NINL,CEP131,CEP152,CLASP1,HAUS5,TUBG2,TUBGCP4,NME7,HSP90AA1,TUBB8,TUBB2B,MZT1,NEK2,NUMA1,ODF2,PAFAH1B1,PCM1,PCNT,TUBA8,PLK1,HAUS6,NDE1,HAUS4,CEP192,HAUS2,PPP2R1A,HAUS7,PRKACA,CEP72,CDK5RAP2,PRKAR2B,CENPJ,MZT2A,TUBA4A,TUBA3C,TUBB2A,TUBG1,YWHAE,YWHAG,ALMS1,TUBA1A,HAUS3,TUBAL3,CEP76,MZT2B,CEP290,CEP63,CEP70,TUBB1,CEP78,TUBB6,TUBA1C,OFD1,TUBGCP6,SSNA1,DYNLL1,HAUS8,CEP41,CEP135,CEP57,CCP110,CKAP5,SFI1,CDK1",Recruitment of NuMA to mitotic centrosomes,94

R-HSA-380612,"ALDH2,MAOA",Metabolism of serotonin,2

R-HSA-380615,"ALDH2,MAOA,SLC6A4",Serotonin clearance from the synaptic cleft,3

R-HSA-380972,"RRAGB,LAMTOR5,RRAGA,SLC38A9,MTOR,LAMTOR2,LAMTOR4,PRKAG2,CAB39,PRKAG3,PPM1A,LAMTOR1,STRADB,PRKAA1,PRKAA2,PRKAB1,PRKAB2,PRKAG1,RPTOR,RRAGD,RHEB,RRAGC,MLST8,STK11,TSC1,TSC2,CAB39L,LAMTOR3,STRADA",Energy dependent regulation of mTOR by LKB1-AMPK,29

R-HSA-380994,"CEBPB,CEBPG,EXOSC8,EXOSC6,DDIT3,DCP2,DIS3,ATF6,EXOSC7,EXOSC2,IGFBP1,CXCL8,ASNS,ATF3,ATF4,ATF4,NFYA,NFYB,NFYC,PARN,EXOSC3,EXOSC1,EXOSC9,EXOSC4,EXOSC5,CCL2,KHSRP,HERPUD1",ATF4 activates genes in response to endoplasmic reticulum stress,28

R-HSA-381033,"DDIT3,ATF6,ATF6,HSPA5,ATF4,ATF4,NFYA,NFYA,NFYB,NFYB,NFYC,NFYC,MBTPS2,HSP90B1,XBP1,CALR,MBTPS1",ATF6 (ATF6-alpha) activates chaperones,17

R-HSA-381038,"PLA2G4B,CTDSP2,PREB,PDIA6,HYOU1,YIF1A,PDIA5,KDELR3,KLHDC3,ADD1,TPP1,DCTN1,DDX11,EXTL1,EXTL2,EXTL3,SEC31A,GFPT1,SERP1,GSK3A,HDGF,CXXC1,ACADVL,LMNA,DNAJB9,SULT1A4,DNAJB11,FKBP14,WIPI1,PPP2R5B,ARFGAP1,MYDGF,DNAJC3,SRPRB,TSPYL2,SHC1,SRPRA,SSR1,SULT1A3,TLN1,WFS1,XBP1,ZBTB17,SYVN1,JMJD7-PLA2G4B,ATP6V0D1,GOSR2,EDEM1,TATDN2,CUL7",XBP1(S) activates chaperone genes,50

R-HSA-381042,"CEBPB,CEBPG,EXOSC8,EXOSC6,DDIT3,DCP2,EIF2S1,EIF2S1,EIF2S3,EIF2S3,DIS3,ATF6,EXOSC7,EXOSC2,HSPA5,IGFBP1,CXCL8,ASNS,ATF3,ATF4,ATF4,NFYA,NFYB,NFYC,PARN,EXOSC3,EXOSC1,EXOSC9,EXOSC4,EXOSC5,CCL2,KHSRP,EIF2S2,EIF2S2,EIF2AK3,HERPUD1",PERK regulates gene expression,36

R-HSA-381070,"PLA2G4B,CTDSP2,PREB,PDIA6,HYOU1,YIF1A,PDIA5,KDELR3,KLHDC3,ADD1,TPP1,DCTN1,DDX11,ERN1,ERN1,EXTL1,EXTL2,EXTL3,SEC31A,GFPT1,SERP1,GSK3A,HDGF,CXXC1,HSPA5,ACADVL,LMNA,DNAJB9,SULT1A4,DNAJB11,FKBP14,WIPI1,PPP2R5B,ARFGAP1,MYDGF,DNAJC3,SRPRB,TSPYL2,SHC1,SRPRA,SSR1,SULT1A3,TLN1,WFS1,XBP1,ZBTB17,SYVN1,JMJD7-PLA2G4B,ATP6V0D1,GOSR2,EDEM1,TATDN2,CUL7",IRE1alpha activates chaperones,53
[truncated: 532,450 more chars]
