# Supplementary material for: The mining and construction of a knowledge base for gene-disease association in mitochondrial diseases
Source: Sci Rep. 2021 Dec 13;11:23909. doi: 10.1038/s41598-021-03249-0 (PMC8668972; doi:10.1038/s41598-021-03249-0)
Supplement: Supplementary file 8 — Supplementary Information 8. [file 41598_2021_3249_MOESM8_ESM.docx]

ID,geneSymbol,name,geneN

hsa00010,"HK3,HK1,HK2,HKDC1,GCK,GPI,PFKM,PFKP,PFKL,FBP1,FBP2,ALDOC,ALDOA,ALDOB,TPI1,GAPDH,GAPDHS,PGK2,PGK1,PGAM1,PGAM2,PGAM4,ENO3,ENO2,ENO1,ENO4,PKM,PKLR,PDHA2,PDHA1,PDHB,DLAT,DLD,PDHX,LDHAL6A,LDHAL6B,LDHA,LDHB,LDHC,ADH1A,ADH1B,ADH1C,ADH7,ADH4,ADH5,ADH6,AKR1A1,ALDH2,ALDH3A2,ALDH1B1,ALDH7A1,ALDH9A1,ALDH3B1,ALDH3B2,ALDH1A3,ALDH3A1,ACSS1,ACSS2,GALM,PGM1,PGM2,G6PC,G6PC2,G6PC3,ADPGK,BPGM,MINPP1,PCK1,PCK2",Glycolysis / Gluconeogenesis,69

hsa00020,"CS,ACLY,ACO2,ACO1,IDH1,IDH2,IDH3B,IDH3G,IDH3A,OGDHL,OGDH,DLST,DLD,SUCLG1,SUCLG2,SUCLA2,SDHA,SDHB,SDHC,SDHD,FH,MDH1,MDH2,PC,PCK1,PCK2,PDHA2,PDHA1,PDHB,DLAT,PDHX",Citrate cycle (TCA cycle),31

hsa00030,"GPI,G6PD,PGLS,H6PD,PGD,RPE,RPEL1,TKT,TKTL2,TKTL1,TALDO1,RPIA,DERA,RBKS,PGM1,PGM2,PRPS1L1,PRPS2,PRPS1,RGN,IDNK,GLYCTK,ALDOC,ALDOA,ALDOB,FBP1,FBP2,PFKM,PFKP,PFKL",Pentose phosphate pathway,30

hsa00040,"GUSB,KL,UGT2A1,UGT2A3,UGT2B17,UGT2B11,UGT2B28,UGT1A6,UGT1A4,UGT1A1,UGT1A3,UGT2B10,UGT1A9,UGT2B7,UGT1A10,UGT1A8,UGT1A5,UGT2B15,UGT1A7,UGT2B4,UGT2A2,UGDH,UGP2,AKR1A1,CRYL1,RPE,RPEL1,XYLB,AKR1B1,AKR1B10,DCXR,SORD,DHDH,CRPPA",Pentose and glucuronate interconversions,34

hsa00051,"MPI,PMM2,PMM1,GMPPB,GMPPA,GMDS,TSTA3,FPGT,FCSK,ENOSF1,HK3,HK1,HK2,HKDC1,PFKM,PFKP,PFKL,FBP1,FBP2,PFKFB1,PFKFB2,PFKFB3,PFKFB4,TIGAR,KHK,SORD,AKR1B1,AKR1B10,ALDOC,ALDOA,ALDOB,TPI1,TKFC",Fructose and mannose metabolism,33

hsa00052,"GALM,GALK1,GALT,GALE,UGP2,PGM1,PGM2,HK3,HK1,HK2,HKDC1,GCK,G6PC,G6PC2,G6PC3,GLB1,LCT,LALBA,B4GALT1,B4GALT2,GLA,AKR1B1,AKR1B10,PFKM,PFKP,PFKL,MGAM,MGAM2,GAA,GANC,SI",Galactose metabolism,31

hsa00053,"UGDH,UGT2A1,UGT2A3,UGT2B17,UGT2B11,UGT2B28,UGT1A6,UGT1A4,UGT1A1,UGT1A3,UGT2B10,UGT1A9,UGT2B7,UGT1A10,UGT1A8,UGT1A5,UGT2B15,UGT1A7,UGT2B4,UGT2A2,MIOX,RGN,ALDH2,ALDH3A2,ALDH1B1,ALDH7A1,ALDH9A1",Ascorbate and aldarate metabolism,27

hsa00061,"ACACA,ACACB,ACSF3,NDUFAB1,MCAT,FASN,OXSM,CBR4,HSD17B8,HTD2,MECR,OLAH,ACSL6,ACSL4,ACSL1,ACSL5,ACSL3,ACSBG1,ACSBG2",Fatty acid biosynthesis,19

hsa00062,"ACAA2,HADHB,HADH,HADHA,ECHS1,MECR,PPT1,PPT2,ELOVL1,ELOVL2,ELOVL3,ELOVL4,ELOVL5,ELOVL6,ELOVL7,HSD17B12,HACD2,HACD1,HACD4,HACD3,TECR,ACOT4,ACOT2,ACOT1,ACOT7,THEM4,THEM5",Fatty acid elongation,27

hsa00071,"ACAT2,ACAT1,ACAA1,ACAA2,HADHB,HADH,HADHA,EHHADH,ECHS1,ACOX3,ACOX1,ACADS,ACADM,ACADL,ACADSB,ACADVL,GCDH,ACSL6,ACSL4,ACSL1,ACSL5,ACSL3,ACSBG1,ACSBG2,CPT1A,CPT1B,CPT1C,CPT2,ECI1,ECI2,CYP4A11,CYP4A22,ADH1A,ADH1B,ADH1C,ADH7,ADH4,ADH5,ADH6,ALDH2,ALDH3A2,ALDH1B1,ALDH7A1,ALDH9A1",Fatty acid degradation,44

hsa00072,"HMGCS1,HMGCS2,HMGCL,HMGCLL1,OXCT1,OXCT2,ACAT2,ACAT1,BDH1,BDH2",Synthesis and degradation of ketone bodies,10

hsa00100,"FDFT1,SQLE,LSS,CYP51A1,TM7SF2,MSMO1,NSDHL,HSD17B7,EBP,DHCR24,SC5D,DHCR7,LIPA,CEL,SOAT2,SOAT1,CYP2R1,CYP27B1,CYP24A1",Steroid biosynthesis,19

hsa00120,"CYP46A1,CYP39A1,HSD3B7,CH25H,CYP7B1,CYP7A1,CYP27A1,CYP8B1,AKR1D1,AKR1C4,SLC27A5,AMACR,ACOX2,HSD17B4,SCP2,ACOT8,BAAT",Primary bile acid biosynthesis,17

hsa00130,"TAT,COQ2,COQ3,COQ6,COQ5,COQ7,NQO1,GGCX,VKORC1,VKORC1L1,HPD",Ubiquinone and other terpenoid-quinone biosynthesis,11

hsa00140,"CYP11A1,CYP17A1,STS,SULT2B1,CYP21A2,HSD3B1,HSD3B2,SRD5A1,SRD5A2,SRD5A3,AKR1C2,AKR1C3,CYP11B1,CYP11B2,AKR1D1,AKR1C4,HSD11B1,HSD11B2,AKR1C1,CYP7B1,SULT1E1,HSD17B1,HSD17B2,HSD17B6,HSD17B7,HSD17B8,HSD17B12,DHRS11,CYP1A1,CYP1A2,CYP3A5,CYP3A7,CYP3A7-CYP3A51P,CYP2E1,CYP3A4,CYP1B1,CYP19A1,CYP7A1,UGT2A1,UGT2A3,UGT2B17,UGT2B11,UGT2B28,UGT1A6,UGT1A4,UGT1A1,UGT1A3,UGT2B10,UGT1A9,UGT2B7,UGT1A10,UGT1A8,UGT1A5,UGT2B15,UGT1A7,UGT2B4,UGT2A2,COMT,LRTOMT,HSD17B3",Steroid hormone biosynthesis,60

hsa00190,"ND1,ND2,ND3,ND4,ND4L,ND5,ND6,NDUFS1,NDUFS2,NDUFS3,NDUFS4,NDUFS5,NDUFS6,NDUFS7,NDUFS8,NDUFV1,NDUFV2,NDUFV3,NDUFA1,NDUFA2,NDUFA3,NDUFA4,NDUFA4L2,NDUFA5,NDUFA6,NDUFA7,NDUFA8,NDUFA9,NDUFA10,NDUFAB1,NDUFA11,NDUFA12,NDUFA13,NDUFB1,NDUFB2,NDUFB3,NDUFB4,NDUFB5,NDUFB6,NDUFB7,NDUFB8,NDUFB9,NDUFB10,NDUFB11,NDUFC1,NDUFC2,NDUFC2-KCTD14,SDHA,SDHB,SDHC,SDHD,UQCRFS1,CYTB,CYC1,UQCRC1,UQCRC2,UQCRH,UQCRHL,UQCRB,UQCRQ,UQCR10,UQCR11,COX10,COX3,COX1,COX2,COX4I2,COX4I1,COX5A,COX5B,COX6A1,COX6A2,COX6B1,COX6B2,COX6C,COX7A1,COX7A2,COX7A2L,COX7B,COX7B2,COX7C,COX8C,COX8A,COX11,COX15,COX17,ATP5F1A,ATP5F1B,ATP5F1C,ATP5F1D,ATP5F1E,ATP5PO,ATP6,ATP5PB,ATP5MC1,ATP5MC2,ATP5MC3,ATP5PD,ATP5ME,ATP5MF,ATP5MG,ATP5PF,ATP8,ATP6V1A,ATP6V1B1,ATP6V1B2,ATP6V1C2,ATP6V1C1,ATP6V1D,ATP6V1E2,ATP6V1E1,ATP6V1F,ATP6V1G1,ATP6V1G3,ATP6V1G2,ATP6V1H,TCIRG1,ATP6V0A2,ATP6V0A4,ATP6V0A1,ATP6V0C,ATP6V0B,ATP6V0D1,ATP6V0D2,ATP6V0E1,ATP6V0E2,ATP6AP1,ATP4A,ATP4B,ATP12A,PPA2,PPA1,LHPP",Oxidative phosphorylation,133

hsa00220,"OTC,ASS1,ASL,ARG2,ARG1,NOS1,NOS2,NOS3,GLS2,GLS,GLUL,GLUD2,GLUD1,CPS1,GOT1,GOT2,GPT2,GPT,NAGS,ACY1,ABHD14A-ACY1",Arginine biosynthesis,21

hsa00230,"NUDT9,ADPRM,NUDT5,PGM1,PGM2,PRPS1L1,PRPS2,PRPS1,PPAT,GART,PFAS,PAICS,ADSL,ATIC,APRT,NT5C2,NT5C1A,NT5C1B,NT5C,NT5M,NT5C3A,NT5C3B,NT5C1B-RDH14,NT5E,PNP,HPRT1,IMPDH1,IMPDH2,NME6,NME7,NME2,NME4,NME1,NME3,NME1-NME2,AK9,ENTPD3,ENTPD8,ENTPD1,CANT1,ENTPD4,ENTPD5,ENTPD6,NUDT16,ITPA,XDH,NUDT2,GMPS,GMPR,GMPR2,GDA,GUK1,PKM,PKLR,RRM1,RRM2B,RRM2,DGUOK,HDDC3,PRUNE1,ADCY1,ADCY2,ADCY3,ADCY4,ADCY5,ADCY6,ADCY7,ADCY8,ADCY9,ADCY10,GUCY1A2,GUCY1A1,GUCY1B1,GUCY2C,GUCY2D,GUCY2F,NPR1,NPR2,PDE1A,PDE1B,PDE1C,PDE2A,PDE3A,PDE3B,PDE5A,PDE6A,PDE6B,PDE6C,PDE6D,PDE6G,PDE6H,PDE9A,PDE10A,PDE11A,ADSS1,ADSS2,AMPD2,AMPD3,AMPD1,ADK,DCK,ADA,ADA2,AK7,AK4,AK5,AK2,AK1,LOC390877,AK8,AK6,AK3,ENTPD2,NTPCR,PDE4A,PDE4B,PDE4C,PDE4D,PDE7A,PDE7B,PDE8B,PDE8A,FHIT,ENPP4,PAPSS2,PAPSS1,ENPP1,ENPP3,URAD,ALLC",Purine metabolism,130

hsa00232,"CYP1A2,NAT2,NAT1,CYP2A6,XDH",Caffeine metabolism,5

hsa00240,"CAD,DHODH,UMPS,CMPK1,CMPK2,NME6,NME7,NME2,NME4,NME1,NME3,NME1-NME2,AK9,ENTPD3,ENTPD8,ENTPD1,CANT1,ENTPD4,ENTPD5,ENTPD6,CTPS1,CTPS2,UCK1,UCK2,UCKL1,NT5C2,NT5C1A,NT5C1B,NT5C,NT5M,NT5C3A,NT5C3B,NT5C1B-RDH14,NT5E,UPP2,UPP1,UPRT,DPYD,DPYS,UPB1,ENPP1,ENPP3,RRM1,RRM2B,RRM2,DCTPP1,DUT,TYMS,CDA,TYMP,PNP,DCK,DCTD,TK2,TK1,DTYMK,NUDT2",Pyrimidine metabolism,57

hsa00250,"GOT1,GOT2,IL4I1,DDO,ASRGL1,ASNS,NIT2,GPT2,GPT,AGXT,AGXT2,ASS1,ASL,ADSS1,ADSS2,ADSL,NAT8L,RIMKLB,RIMKLA,FOLH1,ASPA,GAD1,GAD2,ABAT,ALDH5A1,GLUD2,GLUD1,ALDH4A1,GLUL,CAD,GLS2,GLS,CPS1,GFPT2,GFPT1,PPAT","Alanine, aspartate and glutamate metabolism",36

hsa00260,"SHMT2,SHMT1,AGXT,GRHPR,GLYCTK,PGAM1,PGAM2,PGAM4,BPGM,PHGDH,PSAT1,PSPH,GCAT,ALAS1,ALAS2,MAOB,MAOA,AOC3,AOC2,GLDC,AMT,DLD,GCSH,DAO,AGXT2,GATM,GAMT,CHDH,ALDH7A1,BHMT,DMGDH,PIPOX,SARDH,GNMT,CBS,CBSL,CTH,SDS,SDSL,SRR","Glycine, serine and threonine metabolism",40

hsa00270,"CTH,KYAT3,KYAT1,CBS,CBSL,BHMT,BHMT2,MTR,MAT2B,MAT1A,MAT2A,AMD1,SRM,SMS,MTAP,MRI1,APIP,ENOPH1,ADI1,TAT,IL4I1,DNMT1,DNMT3A,DNMT3B,AHCYL2,AHCYL1,AHCY,BCAT2,BCAT1,AGXT2,GCLC,GCLM,GSS,CDO1,GOT1,GOT2,MPST,TST,LDHAL6A,LDHAL6B,LDHA,LDHB,LDHC,MDH1,MDH2,SDS,SDSL,PHGDH,PSAT1",Cysteine and methionine metabolism,49

hsa00280,"BCAT2,BCAT1,IL4I1,BCKDHA,BCKDHB,DBT,DLD,ACADS,ACADM,IVD,ACADSB,ACAD8,HADHA,EHHADH,ECHS1,HADH,HSD17B10,ACAA1,ACAA2,HADHB,PCCA,PCCB,MCEE,MMUT,HIBCH,HIBADH,ALDH6A1,ALDH2,ALDH3A2,ALDH1B1,ALDH7A1,ALDH9A1,AOX1,ACSF3,ABAT,AGXT2,MCCC1,MCCC2,AUH,HMGCL,HMGCLL1,OXCT1,OXCT2,AACS,ACAT2,ACAT1,HMGCS1,HMGCS2","Valine, leucine and isoleucine degradation",48

hsa00290,"SDS,SDSL,BCAT2,BCAT1","Valine, leucine and isoleucine biosynthesis",4

hsa00310,"AASS,ALDH7A1,AADAT,DHTKD1,DLST,DLD,GCDH,HADHA,EHHADH,ECHS1,HADH,ACAT2,ACAT1,HYKK,PHYKPL,PIPOX,ASH1L,DOT1L,EHMT2,EHMT1,KMT2A,KMT2D,KMT2C,KMT2B,KMT2E,NSD1,NSD2,NSD3,SETD1B,SETD1A,SETD2,SETD3,SETD7,KMT5A,SETDB1,SETDB2,SETMAR,SUV39H1,SUV39H2,KMT5C,KMT5B,EZH1,EZH2,PRDM2,PRDM6,PRDM9,PRDM7,CAMKMT,TMLHE,ALDH2,ALDH3A2,ALDH1B1,ALDH9A1,BBOX1,PLOD1,PLOD2,PLOD3,COLGALT1,COLGALT2",Lysine degradation,59

hsa00330,"GATM,GAMT,CKM,CKMT1A,CKMT2,CKB,CKMT1B,AZIN2,AGMAT,ODC1,SRM,SMS,AMD1,AOC1,SMOX,ALDH2,ALDH3A2,ALDH1B1,ALDH7A1,ALDH9A1,CNDP1,CNDP2,CARNS1,SAT2,SAT1,MAOB,MAOA,NOS1,NOS2,NOS3,ARG2,ARG1,OAT,PYCR3,PYCR2,PYCR1,PRODH,LOC102724788,ALDH4A1,ALDH18A1,LAP3,P4HA2,P4HA3,P4HA1,PRODH2,GOT1,GOT2,HOGA1,DAO,L3HYPDH",Arginine and proline metabolism,50

hsa00340,"HAL,UROC1,AMDHD1,FTCD,HDC,AOC1,ALDH2,ALDH3A2,ALDH1B1,ALDH7A1,ALDH9A1,ASPA,HNMT,MAOB,MAOA,ALDH3B1,ALDH3B2,ALDH1A3,ALDH3A1,CARNS1,CARNMT1,CNDP2,CNDP1",Histidine metabolism,23

hsa00350,"GOT1,GOT2,TAT,IL4I1,HPD,HGD,GSTZ1,FAH,TYR,TH,DCT,TYRP1,DDC,DBH,PNMT,COMT,LRTOMT,MAOB,MAOA,AOC3,AOC2,ALDH3B1,ALDH3B2,ALDH1A3,ALDH3A1,ADH1A,ADH1B,ADH1C,ADH7,ADH4,ADH5,ADH6,TPO,AOX1,FAHD1,MIF",Tyrosine metabolism,36

hsa00360,"PAH,DDC,AOC3,AOC2,MAOB,MAOA,ALDH3B1,ALDH3B2,ALDH1A3,ALDH3A1,GOT1,GOT2,TAT,IL4I1,HPD,MIF,GLYAT",Phenylalanine metabolism,17

hsa00380,"TDO2,IDO1,IDO2,AFMID,KMO,KYNU,HAAO,ACMSD,ALDH8A1,DHTKD1,DLST,DLD,GCDH,HADHA,EHHADH,ECHS1,HADH,ACAT2,ACAT1,KYAT3,KYAT1,AADAT,TPH2,TPH1,DDC,MAOB,MAOA,ALDH2,ALDH3A2,ALDH1B1,ALDH7A1,ALDH9A1,AOX1,ASMT,AANAT,CYP1A1,CYP1A2,CYP1B1,INMT,IL4I1,AOC1,CAT",Tryptophan metabolism,42

hsa00400,"GOT1,GOT2,TAT,IL4I1,PAH","Phenylalanine, tyrosine and tryptophan biosynthesis",5

hsa00410,"GADL1,GAD1,GAD2,CNDP1,CARNS1,CNDP2,ABAT,SRM,SMS,SMOX,AOC3,AOC2,ALDH2,ALDH3A2,ALDH1B1,ALDH7A1,ALDH9A1,ALDH3B1,ALDH3B2,ALDH1A3,ALDH3A1,DPYD,DPYS,UPB1,HIBCH,HADHA,EHHADH,ECHS1,ACADS,ACOX3,ACOX1,MLYCD,ALDH6A1",beta-Alanine metabolism,33

hsa00430,"CDO1,GAD1,GAD2,CSAD,GADL1,ADO,GGT7,GGT6,GGT1,GGT5,BAAT",Taurine and hypotaurine metabolism,11

hsa00440,"PCYT2,SELENOI,CEPT1,PCYT1B,PCYT1A,CHPT1",Phosphonate and phosphinate metabolism,6

hsa00450,"KYAT3,KYAT1,MTR,CTH,SCLY,TXNRD1,TXNRD3,TXNRD2,INMT,PAPSS2,PAPSS1,SEPHS2,SEPHS1,PSTK,SEPSECS,MARS1,MARS2",Selenocompound metabolism,17

hsa00471,"GLS2,GLS,GLUD2,GLUD1,DGLUCY",D-Glutamine and D-glutamate metabolism,5

hsa00472,DAO,D-Arginine and D-ornithine metabolism,1

hsa00480,"GGT7,GGT6,GGT1,GGT5,GGCT,CHAC1,CHAC2,OPLAH,GCLC,GCLM,GSS,LAP3,ANPEP,GSTA5,GSTA2,GSTA4,GSTO2,GSTM4,GSTT2,GSTT1,GSTM3,MGST1,MGST3,GSTP1,GSTM1,GSTM5,MGST2,GSTA1,GSTM2,GSTA3,GSTO1,GSTT2B,GSTK1,HPGDS,NAT8,NAT8B,GSR,IDH1,IDH2,PGD,G6PD,TXNDC12,GPX6,GPX7,GPX2,GPX3,GPX1,GPX5,GPX8,GPX4,ODC1,SRM,SMS,RRM1,RRM2B,RRM2",Glutathione metabolism,56

hsa00500,"MGAM,MGAM2,GAA,GANC,SI,UGP2,ENPP1,ENPP3,GBA3,GYS2,GYS1,GYG1,GYG2,GBE1,PYGL,PYGM,PYGB,AGL,AMY1C,AMY2A,AMY1A,AMY2B,AMY1B,TREH,PGM1,PGM2,HK3,HK1,HK2,HKDC1,GCK,G6PC,G6PC2,G6PC3,PGM2L1,GPI",Starch and sucrose metabolism,36

hsa00510,"SRD5A3,DOLK,DPAGT1,ALG5,ALG13,ALG14,DPM1,DPM2,DPM3,ALG1,ALG2,ALG11,ALG3,ALG9,ALG12,ALG6,ALG8,ALG10,ALG10B,STT3A,STT3B,RPN1,RPN2,DAD1,TUSC3,DDOST,DOLPP1,MOGS,GANAB,MAN1B1,MAN1A2,MAN1C1,MAN1A1,MGAT1,MAN2A1,MAN2A2,MGAT2,FUT8,B4GALT1,B4GALT2,B4GALT3,ST6GAL1,ST6GAL2,MGAT3,MGAT4A,MGAT4B,MGAT4D,MGAT5,MGAT5B,MGAT4C",N-Glycan biosynthesis,50

hsa00511,"NEU1,NEU3,NEU4,NEU2,GLB1,HEXA,HEXB,HEXD,MAN2C1,MAN2B1,MAN2B2,MANBA,ENGASE,FUCA1,FUCA2,AGA,GBA,GBA2",Other glycan degradation,18

hsa00512,"GALNTL6,GALNT5,GALNT17,GALNT11,GALNT12,GALNT13,GALNT14,GALNT16,GALNT15,GALNT18,GALNTL5,GALNT10,GALNT2,GALNT3,GALNT1,GALNT6,GALNT4,GALNT9,GALNT7,GALNT8,POC1B-GALNT4,C1GALT1,C1GALT1C1,GCNT1,GCNT3,GCNT4,ST3GAL1,ST3GAL2,ST6GALNAC1,B3GNT6,B4GALT5",Mucin type O-glycan biosynthesis,31

hsa00513,"ALG13,ALG14,ALG1,ALG2,ALG11,ALG3,ALG9,ALG12,STT3A,STT3B,RPN1,RPN2,DAD1,TUSC3,DDOST,MAN1B1,MAN1A2,MAN1C1,MAN1A1,MGAT1,MAN2A1,MAN2A2,MGAT2,HEXA,HEXB,FUT8,HEXD,MGAT4C,MGAT4A,MGAT4B,MGAT4D,B4GALT1,B4GALT2,B4GALT3,ST3GAL3,B4GALNT3,B4GALNT4,CHST8,CHST9",Various types of N-glycan biosynthesis,39

hsa00514,"OGT,EOGT,POFUT1,POFUT2,MFNG,LFNG,RFNG,B4GALT1,B4GALT2,B4GALT3,ST6GAL1,ST6GAL2,ST3GAL3,B3GLCT,POGLUT1,GXYLT1,GXYLT2,COLGALT1,COLGALT2,PLOD3,POMT1,POMT2",Other types of O-glycan biosynthesis,22

hsa00515,"POMT1,POMT2,POMGNT1,B4GALT1,B4GALT2,B4GALT3,ST3GAL3,B3GAT1,B3GAT2,CHST10,FUT9,FUT4,MGAT5B,POMGNT2,B3GALNT2,POMK,CRPPA,FKTN,FKRP,RXYLT1,B4GAT1,LARGE1,LARGE2",Mannose type O-glycan biosynthesis,23

hsa00520,"CHIA,CHIT1,HEXA,HEXB,NAGK,PGM3,UAP1,UAP1L1,GNE,RENBP,NANS,NANP,NPL,CMAS,CYB5R1,CYB5R3,CYB5R2,CYB5RL,CYB5R4,HK3,HK1,HK2,HKDC1,AMDHD2,GNPNAT1,GNPDA1,GNPDA2,GFPT2,GFPT1,UXS1,GCK,GPI,PGM1,PGM2,UGP2,UGDH,GALK1,GALT,GALE,PMM2,PMM1,GMPPB,GMPPA,GMDS,MPI,FCSK,FPGT,TSTA3",Amino sugar and nucleotide sugar metabolism,48

hsa00524,"HK3,HK1,HK2,HKDC1,GCK","Neomycin, kanamycin and gentamicin biosynthesis",5

hsa00531,"HYAL2,HYAL1,SPAM1,HYAL4,HYAL3,GUSB,IDS,IDUA,ARSB,HPSE,HPSE2,SGSH,HGSNAT,NAGLU,GALNS,GLB1,GNS,HEXA,HEXB",Glycosaminoglycan degradation,19

hsa00532,"XYLT1,XYLT2,B4GALT7,B3GALT6,B3GAT3,CSGALNACT1,CSGALNACT2,CHSY3,CHSY1,CHPF,CHPF2,DSE,CHST11,CHST12,CHST13,CHST3,CHST7,CHST15,UST,CHST14",Glycosaminoglycan biosynthesis - chondroitin sulfate / dermatan sulfate,20

hsa00533,"FUT8,B4GALT1,B4GALT2,B4GALT3,B3GNT2,CHST6,B4GALT4,B3GNT7,CHST1,ST3GAL3,CHST2,CHST4,ST3GAL1,ST3GAL2",Glycosaminoglycan biosynthesis - keratan sulfate,14

hsa00534,"XYLT1,XYLT2,B4GALT7,B3GALT6,B3GAT3,EXTL2,EXTL3,EXTL1,EXT1,EXT2,NDST1,NDST2,NDST3,NDST4,GLCE,HS2ST1,HS6ST1,HS6ST2,HS6ST3,HS3ST1,HS3ST2,HS3ST3B1,HS3ST3A1,HS3ST5",Glycosaminoglycan biosynthesis - heparan sulfate / heparin,24

hsa00561,"GLYCTK,ALDH2,ALDH3A2,ALDH1B1,ALDH7A1,ALDH9A1,AKR1B1,AKR1B10,AKR1A1,TKFC,GK2,GK,GPAM,GPAT2,GPAT4,GPAT3,AGPAT1,AGPAT2,AGPAT3,AGPAT4,AGPAT5,LCLAT1,MBOAT1,MBOAT2,PLPP1,PLPP3,PLPP2,LPIN1,LPIN3,LPIN2,PLPP5,PLPP4,DGKZ,DGKD,DGKI,DGKA,DGKE,DGKB,DGKH,DGKG,DGKQ,DGKK,DGAT1,DGAT2,MOGAT3,CEL,PNPLA2,PNPLA3,PNLIP,PNLIPRP1,PNLIPRP2,PNLIPRP3,LIPC,LIPF,LIPG,LPL,AGK,MGLL,MOGAT1,MOGAT2,GLA",Glycerolipid metabolism,61

hsa00562,"PIK3C3,MTM1,MTMR1,MTMR2,MTMR3,MTMR4,MTMR8,MTMR6,MTMR7,MTMR14,PI4KA,PI4KB,PI4K2A,PI4K2B,SACM1L,INPP5F,PIP5K1C,PIP5K1A,PIP5K1B,PIP5KL1,OCRL,INPP5B,INPP5E,SYNJ1,SYNJ2,PIK3CA,PIK3CD,PIK3CB,PIK3CG,PTEN,INPP5D,INPPL1,PIK3C2G,PIK3C2A,PIK3C2B,INPP4A,INPP4B,PIKFYVE,FIG4,PIP4K2C,PIP4K2A,PIP4K2B,PLCB1,PLCB2,PLCB3,PLCB4,PLCD1,PLCD3,PLCD4,PLCE1,PLCG1,PLCG2,PLCZ1,PLCH1,PLCH2,IMPA2,IMPA1,IMPAD1,CDIPT,ISYNA1,INPP1,INPP5K,INPP5J,INPP5A,MINPP1,ITPKB,ITPKA,ITPKC,ITPK1,IPMK,IPPK,MIOX,ALDH6A1,TPI1",Inositol phosphate metabolism,74

hsa00563,"PIGA,PIGC,PIGH,PIGP,PIGQ,PIGY,DPM2,PIGL,PIGW,GPLD1,PIGM,PIGX,PIGV,PIGN,PIGB,PIGO,PIGF,GPAA1,PIGK,PIGS,PIGT,PIGU,PGAP1,PIGZ,PIGG",Glycosylphosphatidylinositol (GPI)-anchor biosynthesis,25

hsa00564,"GPD1L,GPD1,GPD2,GPAM,GPAT2,GPAT4,GPAT3,AGPAT1,AGPAT2,AGPAT3,AGPAT4,AGPAT5,LCLAT1,MBOAT1,MBOAT2,GNPAT,ADPRM,PLPP1,PLPP3,PLPP2,LPIN1,LPIN3,LPIN2,PLPP5,PLPP4,DGKZ,DGKD,DGKI,DGKA,DGKE,DGKB,DGKH,DGKG,DGKQ,DGKK,CHPT1,CEPT1,PLD1,PLD2,PLD3,PLD4,LCAT,PLA2G10,PLA2G2D,PLA2G2E,PLA2G3,PLA2G2F,PLA2G12A,PLA2G12B,PLA2G1B,PLA2G5,PLA2G2A,PLA2G2C,PLA2G4E,PLA2G4A,JMJD7-PLA2G4B,PLA2G4B,PLA2G4C,PLA2G4D,PLA2G4F,PLA2G6,PLB1,PLAAT3,LPCAT2,LPCAT1,LPCAT4,LPCAT3,LYPLA1,PLA2G15,LYPLA2,PNPLA6,PNPLA7,GPCPD1,CHAT,ACHE,CHKA,CHKB,PHOSPHO1,PCYT1B,PCYT1A,SELENOI,ETNK1,ETNK2,PCYT2,ETNPPL,PEMT,CDS1,CDS2,PTDSS1,PTDSS2,PISD,PGS1,CRLS1,TAZ,LPGAT1,CDIPT,MBOAT7",Glycerophospholipid metabolism,97

hsa00565,"AGPS,PLPP1,PLPP3,PLPP2,SELENOI,CHPT1,CEPT1,PLA2G10,PLA2G2D,PLA2G2E,PLA2G3,PLA2G2F,PLA2G12A,PLA2G12B,PLA2G1B,PLA2G5,PLA2G2A,PLA2G2C,PLA2G4E,PLA2G4A,JMJD7-PLA2G4B,PLA2G4B,PLA2G4C,PLA2G4D,PLA2G4F,PLA2G6,PLB1,PLAAT3,LPCAT4,ENPP6,ENPP2,GDPD3,GDPD1,TMEM86B,PLD1,PLD2,PLD3,PLD4,UGT8,GAL3ST1,LPCAT2,LPCAT1,PAFAH1B1,PAFAH1B2,PAFAH1B3,PLA2G7,PAFAH2",Ether lipid metabolism,47

hsa00590,"PLA2G10,PLA2G2D,PLA2G2E,PLA2G3,PLA2G2F,PLA2G12A,PLA2G12B,PLA2G1B,PLA2G5,PLA2G2A,PLA2G2C,PLA2G4E,PLA2G4A,JMJD7-PLA2G4B,PLA2G4B,PLA2G4C,PLA2G4D,PLA2G4F,PLA2G6,PLB1,PLAAT3,PTGS1,PTGS2,PTGES,PTGES2,PTGES3,CBR1,CBR3,PRXL2B,TBXAS1,PTGDS,HPGDS,AKR1C3,PTGIS,ALOX5,LTA4H,CYP4F2,CYP4F3,LTC4S,GGT1,GGT5,GPX6,GPX7,GPX2,GPX3,GPX1,GPX5,GPX8,CYP2E1,CYP2J2,CYP2U1,CYP4A11,CYP4A22,CYP2C19,CYP4F8,ALOX12,ALOX12B,ALOX15B,CYP2B6,CYP2C8,CYP2C9,EPHX2,ALOX15",Arachidonic acid metabolism,63

hsa00591,"PLA2G10,PLA2G2D,PLA2G2E,PLA2G3,PLA2G2F,PLA2G12A,PLA2G12B,PLA2G1B,PLA2G5,PLA2G2A,PLA2G2C,PLA2G4E,PLA2G4A,JMJD7-PLA2G4B,PLA2G4B,PLA2G4C,PLA2G4D,PLA2G4F,PLA2G6,PLB1,PLAAT3,ALOX15,CYP1A2,CYP2C8,CYP2C9,CYP2C19,CYP2J2,CYP2E1,CYP3A4",Linoleic acid metabolism,29

hsa00592,"PLA2G10,PLA2G2D,PLA2G2E,PLA2G3,PLA2G2F,PLA2G12A,PLA2G12B,PLA2G1B,PLA2G5,PLA2G2A,PLA2G2C,PLA2G4E,PLA2G4A,JMJD7-PLA2G4B,PLA2G4B,PLA2G4C,PLA2G4D,PLA2G4F,PLA2G6,PLB1,PLAAT3,FADS2,ACOX3,ACOX1,ACAA1",alpha-Linolenic acid metabolism,25

hsa00600,"SPTLC1,SPTLC2,SPTLC3,KDSR,CERS2,CERS3,CERS6,CERS1,CERS4,CERS5,ASAH1,ASAH2,ACER2,ACER1,ACER3,DEGS1,DEGS2,SGMS1,SGMS2,SMPD1,SMPD2,SMPD3,SMPD4,ENPP7,CERK,PLPP1,PLPP3,PLPP2,SGPP1,SGPP2,SPHK1,SPHK2,SGPL1,UGCG,GBA,GBA2,B4GALT6,GLB1,UGT8,GALC,GAL3ST1,ARSA,NEU1,NEU3,NEU4,NEU2,GLA",Sphingolipid metabolism,47

hsa00601,"B3GNT5,B3GALT1,B3GALT2,B3GALT5,FUT1,FUT2,FUT3,ST3GAL3,ST3GAL4,ABO,B4GALT1,B4GALT2,B4GALT3,B4GALT4,FUT9,FUT4,FUT5,FUT6,FUT7,ST3GAL6,ST8SIA1,A4GALT,B3GALNT1,B3GNT2,B3GNT3,B3GNT4,GCNT2",Glycosphingolipid biosynthesis - lacto and neolacto series,27

hsa00603,"A3GALT2,B3GALNT1,GBGT1,A4GALT,GLA,B3GALT5,HEXA,HEXB,NAGA,FUT1,FUT2,ST3GAL1,ST3GAL2,ST8SIA1,FUT9",Glycosphingolipid biosynthesis - globo and isoglobo series,15

hsa00604,"B4GALNT1,B3GALT4,ST3GAL2,ST3GAL1,ST8SIA5,ST3GAL5,ST8SIA1,SLC33A1,ST6GALNAC3,ST6GALNAC4,ST6GALNAC5,ST6GALNAC6,GLB1,HEXA,HEXB",Glycosphingolipid biosynthesis - ganglio series,15

hsa00620,"ACSS1,ACSS2,PDHA2,PDHA1,PDHB,DLAT,DLD,PDHX,PKM,PKLR,ACACA,ACACB,ACYP2,ACYP1,ALDH2,ALDH3A2,ALDH1B1,ALDH7A1,ALDH9A1,ACOT12,LDHAL6A,LDHAL6B,LDHA,LDHB,LDHC,LDHD,GLO1,HAGH,GRHPR,ME2,ME3,ME1,PC,MDH1,MDH2,FH,PCK1,PCK2,ACAT2,ACAT1",Pyruvate metabolism,40

hsa00630,"ACSS1,ACSS2,MDH1,MDH2,CS,ACO2,ACO1,ACAT2,ACAT1,MCEE,PCCA,PCCB,MMUT,HAO2,HAO1,CAT,GRHPR,PGP,AGXT,GLUL,SHMT2,SHMT1,GLDC,AMT,DLD,GCSH,HYI,GLYCTK,HOGA1,AFMID",Glyoxylate and dicarboxylate metabolism,30

hsa00640,"ACSS1,ACSS2,ACSS3,BCKDHA,BCKDHB,DBT,DLD,ACADS,ACOX3,ACOX1,HADHA,EHHADH,ECHS1,HIBCH,ACACA,ACACB,MLYCD,ABAT,PCCA,PCCB,ECHDC1,MCEE,MMUT,SUCLG1,SUCLG2,SUCLA2,ALDH6A1,LDHAL6A,LDHAL6B,LDHA,LDHB,LDHC,ACAT2,ACAT1",Propanoate metabolism,34

hsa00650,"ACAT2,ACAT1,HADH,HADHA,EHHADH,ECHS1,ACADS,ACSM1,ACSM2A,ACSM4,ACSM5,ACSM3,ACSM2B,ACSM6,L2HGDH,GAD1,GAD2,ABAT,ALDH5A1,HMGCS1,HMGCS2,HMGCL,HMGCLL1,OXCT1,OXCT2,AACS,BDH1,BDH2",Butanoate metabolism,28

hsa00670,"DHFR,DHFR2,MTHFD1L,MTHFD1,MTHFD2,MTHFD2L,SHMT2,SHMT1,GART,ATIC,FTCD,MTFMT,AMT,MTR,TYMS,ALDH1L1,ALDH1L2,MTHFR,MTHFS,ST20-MTHFS",One carbon pool by folate,20

hsa00730,"NFS1,NTPCR,ALPL,ALPI,ALPP,ALPG,ACP1,TPK1,AK7,AK4,AK5,AK2,AK1,LOC390877,AK8,THTPA",Thiamine metabolism,16

hsa00740,"RFK,ACP5,ACP1,ACP2,FLAD1,ENPP1,ENPP3,BLVRB",Riboflavin metabolism,8

hsa00750,"PNPO,PDXK,PDXP,PHOSPHO2,AOX1,PSAT1",Vitamin B6 metabolism,6

hsa00760,"ASPDH,QPRT,NAPRT,PNP,NMRK1,NMRK2,NT5C2,NT5C1A,NT5C1B,NT5C,NT5M,NT5C3A,NT5C3B,NT5C1B-RDH14,NT5E,NMNAT3,NMNAT1,NMNAT2,ENPP1,ENPP3,NUDT12,NADSYN1,NAMPT,CD38,BST1,SIRT1,SIRT2,SIRT3,SIRT4,SIRT5,SIRT6,SIRT7,NADK,NADK2,NNT,NNMT,AOX1",Nicotinate and nicotinamide metabolism,37

hsa00770,"PANK1,PANK4,PANK3,PANK2,PPCS,PPCDC,ENPP1,ENPP3,COASY,AASDHPPT,VNN1,VNN2,VNN3,BCAT2,BCAT1,DPYD,DPYS,UPB1,GADL1",Pantothenate and CoA biosynthesis,19

hsa00780,"OXSM,HLCS,BTD",Biotin metabolism,3

hsa00785,"LIAS,LIPT2,LIPT1",Lipoic acid metabolism,3

hsa00790,"GCH1,ALPL,ALPI,ALPP,ALPG,DHFR,DHFR2,FPGS,GGH,PTS,SPR,AKR1B1,AKR1B10,CBR1,AKR1C3,QDPR,PCBD2,PCBD1,PAH,TH,TPH2,TPH1,MOCS1,MOCS2,GPHN,MOCOS",Folate biosynthesis,26

hsa00830,"BCO1,ADH1A,ADH1B,ADH1C,ADH7,ADH4,ADH5,ADH6,DHRS3,DHRS4,DHRS4L1,DHRS4L2,DHRS9,RDH8,RDH10,RDH11,RDH12,RDH16,SDR16C5,HSD17B6,LRAT,DGAT1,AWAT2,PNPLA4,RPE65,RDH5,AOX1,ALDH1A2,ALDH1A1,CYP26A1,CYP26B1,CYP26C1,CYP1A1,CYP1A2,CYP2A6,CYP2B6,CYP2C8,CYP2C9,CYP2C18,CYP2S1,CYP3A4,CYP3A5,CYP3A7,CYP3A7-CYP3A51P,CYP4A11,CYP4A22,UGT2A1,UGT2A3,UGT2B17,UGT2B11,UGT2B28,UGT1A6,UGT1A4,UGT1A1,UGT1A3,UGT2B10,UGT1A9,UGT2B7,UGT1A10,UGT1A8,UGT1A5,UGT2B15,UGT1A7,UGT2B4,UGT2A2,RETSAT,CYP27C1",Retinol metabolism,67

hsa00860,"ALAS1,ALAS2,EARS2,EPRS1,ALAD,HMBS,UROS,UROD,CPOX,PPOX,FECH,COX10,COX15,MMAB,HMOX1,HMOX2,BLVRA,BLVRB,UGT2A1,UGT2A3,UGT2B17,UGT2B11,UGT2B28,UGT1A6,UGT1A4,UGT1A1,UGT1A3,UGT2B10,UGT1A9,UGT2B7,UGT1A10,UGT1A8,UGT1A5,UGT2B15,UGT1A7,UGT2B4,UGT2A2,GUSB,HCCS,CP,HEPH,FXN",Porphyrin and chlorophyll metabolism,42

hsa00900,"ACAT2,ACAT1,HMGCS1,HMGCS2,HMGCR,MVK,PMVK,MVD,IDI1,IDI2,FDPS,GGPS1,PDSS1,PDSS2,DHDDS,NUS1,FNTA,FNTB,RCE1,ZMPSTE24,ICMT,PCYOX1",Terpenoid backbone biosynthesis,22

hsa00910,"GLUD2,GLUD1,GLUL,CPS1,CA13,CA1,CA6,CA7,CA12,CA5B,CA14,CA9,CA3,CA5A,CA8,CA2,CA4",Nitrogen metabolism,17

hsa00920,"PAPSS2,PAPSS1,BPNT1,IMPAD1,SUOX,SQOR,ETHE1,MPST,TST,SELENBP1",Sulfur metabolism,10

hsa00970,"TRNA,TRNR,TRNN,TRND,TRNC,TRNQ,TRNE,TRNG,TRNH,TRNI,TRNL2,TRNL1,TRNK,TRNM,TRNF,TRNP,TRNS2,TRNS1,TRNT,TRNW,TRNY,TRNV,EARS2,EPRS1,QRSL1,GATB,GATC,QARS1,AARS2,AARS1,DARS1,DARS2,NARS2,NARS1,GARS1,TARS2,TARS1,TARS3,SARS2,SARS1,PSTK,SEPSECS,CARS1,CARS2,MARS1,MARS2,MTFMT,VARS2,VARS1,LARS1,LARS2,IARS1,IARS2,KARS1,RARS2,RARS1,PARS2,HARS1,HARS2,FARSA,FARS2,FARSB,YARS1,YARS2,WARS2,WARS1",Aminoacyl-tRNA biosynthesis,66

hsa00980,"CYP1A1,CYP2C9,CYP3A4,CYP1B1,GSTA5,GSTA2,GSTA4,GSTO2,GSTM4,GSTT2,GSTT1,GSTM3,MGST1,MGST3,GSTP1,GSTM1,GSTM5,MGST2,GSTA1,GSTM2,GSTA3,GSTO1,GSTT2B,GSTK1,HPGDS,EPHX1,CYP2B6,SULT2A1,CYP1A2,CYP2A6,CYP2E1,CYP2F1,CYP2S1,AKR1C1,DHDH,CYP2A13,CYP2D6,LOC107987478,CYP2D7,LOC107987479,HSD11B1,CBR1,CBR3,UGT2A1,UGT2A3,UGT2B17,UGT2B11,UGT2B28,UGT1A6,UGT1A4,UGT1A1,UGT1A3,UGT2B10,UGT1A9,UGT2B7,UGT1A10,UGT1A8,UGT1A5,UGT2B15,UGT1A7,UGT2B4,UGT2A2,CYP3A5,AKR7A2,AKR7A3,ALDH3B1,ALDH3B2,ALDH1A3,ALDH3A1,ADH1A,ADH1B,ADH1C,ADH7,ADH4,ADH5,ADH6",Metabolism of xenobiotics by cytochrome P450,76

hsa00982,"CYP2D6,LOC107987478,CYP2D7,LOC107987479,CYP2C9,CYP3A4,FMO1,FMO2,FMO5,FMO3,FMO4,CYP2C19,CYP2B6,CYP3A5,GSTA5,GSTA2,GSTA4,GSTO2,GSTM4,GSTT2,GSTT1,GSTM3,MGST1,MGST3,GSTP1,GSTM1,GSTM5,MGST2,GSTA1,GSTM2,GSTA3,GSTO1,GSTT2B,GSTK1,HPGDS,ADH1A,ADH1B,ADH1C,ADH7,ADH4,ADH5,ADH6,ALDH3B1,ALDH3B2,ALDH1A3,ALDH3A1,MAOB,MAOA,AOX1,UGT2A1,UGT2A3,UGT2B17,UGT2B11,UGT2B28,UGT1A6,UGT1A4,UGT1A1,UGT1A3,UGT2B10,UGT1A9,UGT2B7,UGT1A10,UGT1A8,UGT1A5,UGT2B15,UGT1A7,UGT2B4,UGT2A2,CYP1A2,CYP2E1,CYP2C8,CYP2A6",Drug metabolism - cytochrome P450,72

hsa00983,"HPRT1,IMPDH1,IMPDH2,GMPS,TPMT,XDH,ITPA,CES1,CES2,UGT2A1,UGT2A3,UGT2B17,UGT2B11,UGT2B28,UGT1A6,UGT1A4,UGT1A1,UGT1A3,UGT2B10,UGT1A9,UGT2B7,UGT1A10,UGT1A8,UGT1A5,UGT2B15,UGT1A7,UGT2B4,UGT2A2,GUSB,CYP3A4,CDA,TYMP,DPYD,DPYS,UPB1,CYP2A6,UPP2,UPP1,UCK1,UCK2,UCKL1,TK2,TK1,UMPS,CMPK1,RRM1,RRM2B,RRM2,NME6,NME7,NME2,NME4,NME1,NME3,NME1-NME2,DUT,MPO,NAT2,NAT1,CYP2E1,GSTA5,GSTA2,GSTA4,GSTO2,GSTM4,GSTT2,GSTT1,GSTM3,MGST1,MGST3,GSTP1,GSTM1,GSTM5,MGST2,GSTA1,GSTM2,GSTA3,GSTO1,GSTT2B",Drug metabolism - other enzymes,79

hsa01040,"ELOVL1,ELOVL2,ELOVL3,ELOVL4,ELOVL5,ELOVL6,ELOVL7,HSD17B12,HACD2,HACD1,HACD4,HACD3,TECR,SCD,SCD5,FADS2,FADS1,ACOX3,ACOX1,HSD17B4,ACAA1,SCP2,ACOT4,ACOT2,ACOT1,ACOT7,BAAT",Biosynthesis of unsaturated fatty acids,27

hsa01521,"EGF,TGFA,EGFR,ERBB2,NRG1,NRG2,ERBB3,HGF,MET,IGF1,IGF1R,VEGFA,KDR,PDGFA,PDGFB,PDGFC,PDGFD,PDGFRA,PDGFRB,FGF2,FGFR2,FGFR3,GAS6,AXL,IL6,IL6R,JAK1,JAK2,STAT3,SRC,GAB1,PIK3CA,PIK3CD,PIK3CB,PIK3R1,PIK3R2,PIK3R3,PTEN,AKT1,AKT2,AKT3,MTOR,RPS6KB1,RPS6KB2,RPS6,EIF4EBP1,EIF4E,EIF4E2,EIF4E1B,BAD,GSK3B,FOXO3,BCL2L11,PLCG1,PLCG2,PRKCA,PRKCB,PRKCG,SHC1,SHC2,SHC3,SHC4,GRB2,SOS1,SOS2,HRAS,KRAS,NRAS,ARAF,BRAF,RAF1,MAP2K1,MAP2K2,MAPK1,MAPK3,NF1,BAX,BCL2L1,BCL2",EGFR tyrosine kinase inhibitor resistance,79

hsa01522,"CYP2D6,LOC107987478,CYP2D7,LOC107987479,ABCB11,ESR1,ESR2,NCOA3,MED1,CARM1,FOS,JUN,SP1,NCOR1,GPER1,GNAS,SRC,MMP2,MMP9,HBEGF,ADCY1,ADCY2,ADCY3,ADCY4,ADCY5,ADCY6,ADCY7,ADCY8,ADCY9,PRKACA,PRKACB,PRKACG,EGFR,ERBB2,IGF1,IGF1R,SHC1,SHC2,SHC3,SHC4,GRB2,SOS1,SOS2,HRAS,KRAS,NRAS,ARAF,BRAF,RAF1,MAP2K1,MAP2K2,MAPK1,MAPK3,PIK3CA,PIK3CD,PIK3CB,PIK3R1,PIK3R2,PIK3R3,AKT1,AKT2,AKT3,MTOR,RPS6KB1,RPS6KB2,PTK2,MAPK11,MAPK12,MAPK13,MAPK14,MAPK8,MAPK10,MAPK9,BCL2,BAX,BAD,BIK,JAG1,JAG2,DLL3,DLL1,DLL4,NOTCH1,NOTCH2,NOTCH3,NOTCH4,CCND1,CDK4,RB1,E2F1,E2F2,E2F3,MDM2,TP53,CDKN2A,CDKN2C,CDKN1A,CDKN1B",Endocrine resistance,98

hsa01523,"FOLR1,FOLR2,FOLR3,IZUMO1R,SLC19A1,SLC46A1,FPGS,GGH,TYMS,DHFR,DHFR2,SHMT2,SHMT1,MTHFR,ALOX12,GART,ATIC,CHUK,IKBKB,IKBKG,NFKB1,RELA,TNF,IL1B,IL6,ABCC1,ABCC2,ABCC3,ABCC4,ABCC5,ABCG2",Antifolate resistance,31

hsa01524,"SLC31A1,ATP7B,ATP7A,GSTA5,GSTA2,GSTA4,GSTO2,GSTM4,GSTT2,GSTT1,GSTM3,MGST1,MGST3,GSTP1,GSTM1,GSTM5,MGST2,GSTA1,GSTM2,GSTA3,GSTO1,GSTT2B,ABCC2,POLH,REV3L,MSH2,MSH3,MSH6,MLH1,XPA,ERCC1,TOP2B,TOP2A,BRCA1,ATM,TP53,CDKN2A,MDM2,FAS,BAX,BAK1,PMAIP1,BBC3,FASLG,FADD,CASP8,CASP3,BID,BIRC2,BIRC3,XIAP,BIRC5,ERBB2,PIK3CA,PIK3CD,PIK3CB,PIK3R1,PIK3R2,PIK3R3,PDPK1,AKT1,AKT2,AKT3,CDKN1A,MAP3K5,MAPK1,MAPK3,BAD,BCL2L1,BCL2,CYCS,APAF1,CASP9",Platinum drug resistance,73

hsa02010,"DEFB1,ABCA1,ABCA2,ABCA3,ABCA4,ABCA7,ABCA12,ABCA13,ABCA5,ABCA6,ABCA8,ABCA9,ABCA10,TAP1,TAP2,ABCB8,ABCB9,ABCB10,ABCB1,ABCB4,ABCB5,ABCB6,ABCB7,ABCB11,ABCC1,ABCC2,ABCC3,ABCC4,ABCC5,ABCC6,ABCC10,ABCC11,ABCC12,CFTR,ABCC8,ABCC9,ABCD1,ABCD2,ABCD3,ABCD4,ABCG1,ABCG4,ABCG2,ABCG5,ABCG8",ABC transporters,45

hsa03008,"CSNK2B,CSNK2A1,CSNK2A2,CSNK2A3,NOL6,RRP7A,WDR43,UTP4,UTP15,HEATR1,WDR75,UTP18,WDR36,TBL3,WDR3,UTP6,PWP2,MPHOSPH10,LOC643802,IMP3,IMP4,TCOF1,FBL,NOP56,NOP58,SNU13,DKC1,NHP2,GAR1,NOP10,FCF1,UTP14C,UTP14A,DROSHA,EMG1,BMS1,NAT10,RCL1,POP1,RPP38,POP4,POP5,RPP25L,RPP25,POP7,RPP30,RPP40,LOC100288562,REXO1,LOC101929601,REXO5,LOC101929627,REXO2,XRN1,XRN2,GTPBP4,GNL2,GNL3,GNL3L,NVL,MDN1,RBM28,NOB1,RAN,XPO1,NMD3,NXF1,NXF2,NXF2B,NXF5,NXF3,NXT1,NXT2,EIF6,SBDS,EFL1,LSG1,SPATA5,AK6,RIOK1,RIOK2,RNR1,RNR2,RNA5S1,RNA5S16,RNA5S6,RNA5S11,RNA5S5,RNA5S13,RNA5S4,RNA5S3,RNA5S2,RNA5S10,RNA5S12,RNA5S8,RNA5S7,RNA5S14,RNA5S15,RNA5S17,RNA5S9,SNORD3A,SNORD3C,SNORD3B-2,SNORD3B-1,RMRP",Ribosome biogenesis in eukaryotes,105

hsa03010,"MRPS2,MRPS5,MRPS6,MRPS7,MRPS9,MRPS10,MRPS11,MRPS12,MRPS14,MRPS15,MRPS16,MRPS17,MRPS18C,MRPS18A,MRPS21,RPS2,RPS3,RPS3A,RPS4Y1,RPS4X,RPS4Y2,RPS5,RPS6,RPS7,RPS8,RPS9,RPS10,RPS10-NUDT3,RPS11,RPS12,RPS13,RPS14,RPS15,RPS15A,RPS16,RPS17,RPS18,RPS19,RPS20,RPS21,RPS23,RPS24,RPS25,RPS26,RPS27,RPS27L,RPS27A,RPS28,RPS29,FAU,RPSA,MRPL1,MRPL2,MRPL3,MRPL4,MRPL12,MRPL9,MRPL10,MRPL11,MRPL13,MRPL14,MRPL15,MRPL16,MRPL17,MRPL18,MRPL19,MRPL20,MRPL21,MRPL22,MRPL23,MRPL24,MRPL27,MRPL28,MRPL30,MRPL32,MRPL33,MRPL34,MRPL35,MRPL36,RPL3L,RPL3,RPL4,RPL5,RPL6,RPL7,RPL7A,RPL8,RPL9,RPL10L,RPL10,RPL10A,RPL11,RPL12,RPL13,RPL13A,RPL14,RPL15,RPL17,RPL17-C18orf32,RPL18,RPL18A,RPL19,RPL21,RPL22L1,RPL22,RPL23,RPL23A,RSL24D1,RPL24,RPL26,RPL26L1,RPL27,RPL27A,RPL28,RPL29,RPL30,RPL31,RPL32,RPL34,RPL35,RPL35A,RPL36,RPL37,RPL37A,RPL38,RPL39,UBA52,RPL41,RPL36AL,RPL36A,RPL36A-HNRNPH2,RPLP0,RPLP1,RPLP2,RNR1,RNR2,RNA5S1,RNA5S16,RNA5S6,RNA5S11,RNA5S5,RNA5S13,RNA5S4,RNA5S3,RNA5S2,RNA5S10,RNA5S12,RNA5S8,RNA5S7,RNA5S14,RNA5S15,RNA5S17,RNA5S9",Ribosome,153

hsa03013,"POP1,RPP38,POP4,POP5,RPP25L,RPP25,POP7,RPP14,RPP30,RPP21,RPP40,ELAC2,ELAC1,TRNT1,RAN,XPOT,XPO5,EEF1A1,EEF1A2,XPO1,PHAX,NCBP1,NCBP2,CLNS1A,PRMT5,TGS1,KPNB1,SNUPN,NMD3,PYM1,TPR,NUP50,NUP153,SENP2,NUP98,RAE1,SEC13,SEH1L,NUP133,NUP107,NUP37,NUP160,NUP85,NUP43,NUP62,NUP58,NUP54,NUP93,NUP205,NUP188,NUP155,NUP35,NUP210,NUP210L,NDC1,POM121C,POM121,POM121L2,NUP214,NUP88,RANBP2,RANGAP1,UBE2I,SUMO3,SUMO2,SUMO1,SUMO4,AAAS,NUP42,SMN1,SMN2,GEMIN2,DDX20,GEMIN4,GEMIN5,GEMIN6,GEMIN7,GEMIN8,STRAP,EIF3J,EIF3I,EIF3H,EIF3G,EIF3F,EIF3E,EIF3D,EIF3C,EIF3CL,EIF3B,EIF3A,EIF1AY,EIF1AX,EIF1B,EIF1,EIF5,EIF2S1,EIF2S2,EIF2S3,EIF2B1,EIF2B2,EIF2B4,EIF2B3,EIF2B5,EIF5B,EIF4G3,EIF4G1,EIF4G2,EIF4E,EIF4E2,EIF4E1B,EIF4A1,EIF4A2,EIF4B,PABPC1,PABPC5,PABPC3,PABPC1L2B,PABPC1L,PABPC1L2A,PABPC4,PABPC4L,PAIP1,EIF4EBP1,EIF4EBP2,EIF4EBP3,TACC3,CYFIP1,CYFIP2,FMR1,FXR1,FXR2,RBM8A,MAGOH,MAGOHB,CASC3,EIF4A3,SAP18,PNN,ACIN1,RNPS1,ALYREF,DDX39B,THOC1,THOC2,THOC5,THOC6,THOC7,THOC3,SRRM1,NXF1,NXF2,NXF2B,NXF5,NXF3,NXT1,NXT2,UPF1,UPF2,UPF3B,UPF3A,RNU6-1,SNORD3A,SNORD3C,SNORD3B-2,SNORD3B-1",RNA transport,165

hsa03015,"NCBP1,NCBP2,UPF3B,UPF3A,RBM8A,MAGOH,MAGOHB,CASC3,EIF4A3,SAP18,PNN,ACIN1,RNPS1,ALYREF,NXF1,NXF2,NXF2B,NXF5,NXF3,NXT1,NXT2,DDX39B,PYM1,SRRM1,RNGTT,RNMT,PABPN1,PABPN1L,BCL2L2-PABPN1,NUDT21,CPSF6,CPSF7,PAPOLG,PAPOLB,PAPOLA,CLP1,PCF11,CPSF1,CPSF2,CPSF3,CPSF4,FIP1L1,WDR33,WDR82,PPP1CA,PPP1CB,PPP1CC,SSU72,CSTF1,CSTF2,CSTF2T,CSTF3,SYMPK,DAZAP1,MSI1,MSI2,PABPC1,PABPC5,PABPC3,PABPC1L2B,PABPC1L,PABPC1L2A,PABPC4,PABPC4L,ETF1,GSPT2,GSPT1,UPF1,UPF2,SMG1,SMG7,SMG5,SMG6,PPP2CA,PPP2CB,PPP2R1B,PPP2R1A,PPP2R2A,PPP2R2B,PPP2R2C,PPP2R2D,PPP2R3B,PPP2R3C,PPP2R3A,PPP2R5B,PPP2R5C,PPP2R5D,PPP2R5E,PPP2R5A,HBS1L,PELO",mRNA surveillance pathway,91

hsa03018,"DCPS,EXOSC1,EXOSC2,EXOSC3,EXOSC8,EXOSC6,EXOSC7,EXOSC4,EXOSC5,EXOSC9,DIS3,EXOSC10,C1D,MPHOSPH6,DIS3L,TENT4A,TENT4B,ZCCHC7,MTREX,SKIV2L,TTC37,WDR61,CNOT6,CNOT6L,CNOT1,CNOT2,CNOT3,CNOT4,CNOT7,CNOT8,CNOT9,CNOT10,DHX36,PARN,PNLDC1,TOB1,TOB2,BTG3,BTG4,BTG1,BTG2,PABPC1,PABPC5,PABPC3,PABPC1L2B,PABPC1L,PABPC1L2A,PABPC4,PABPC4L,PAN2,PAN3,DCP1A,DCP1B,DCP2,DDX6,EDC3,EDC4,PATL1,XRN1,XRN2,NUDT16,LSM1,LSM2,LSM3,LSM4,LSM5,LSM6,LSM7,LSM8,ENO3,ENO2,ENO1,ENO4,PNPT1,PFKM,PFKP,PFKL,HSPA9,HSPD1",RNA degradation,79

hsa03020,"POLR2B,POLR2A,POLR2C,POLR2J,POLR2J3,POLR2J2,POLR2D,POLR2G,POLR2I,POLR2E,POLR2F,POLR2H,POLR2K,POLR2L,POLR3B,POLR3A,POLR1D,POLR1C,POLR3C,POLR3D,POLR3E,POLR3K,POLR3H,POLR3GL,POLR3G,POLR3F,POLR1B,POLR1A,ZNRD1,POLR1E,TWISTNB",RNA polymerase,31

hsa03022,"GTF2A1L,GTF2A1,GTF2A2,GTF2B,TBPL2,TBPL1,TBP,TAF1,TAF1L,TAF2,TAF7,TAF7L,TAF8,TAF3,TAF10,TAF5L,TAF5,TAF4B,TAF4,TAF12,TAF6,TAF6L,TAF9,TAF9B,TAF11,TAF13,TAF15,GTF2E1,GTF2E2,GTF2F1,GTF2F2,GTF2H1,GTF2H2,GTF2H2C_2,GTF2H2C,GTF2H3,GTF2H4,ERCC3,ERCC2,GTF2H5,CDK7,MNAT1,CCNH,GTF2IRD1,GTF2I",Basal transcription factors,45

hsa03030,"SSBP1,RNASEH1,RPA1,PCNA,DNA2,FEN1,LIG1,POLA1,POLA2,PRIM1,PRIM2,POLD1,POLD2,POLD3,POLD4,POLE,POLE2,POLE3,POLE4,MCM2,MCM3,MCM4,MCM5,MCM6,MCM7,RPA2,RPA4,RPA3,RFC1,RFC4,RFC2,RFC5,RFC3,RNASEH2A,RNASEH2B,RNASEH2C",DNA replication,36

hsa03040,"RNU6-1,DDX46,DDX39B,DHX16,DHX38,CDC40,PRPF18,DHX8,SLU7,DHX15,SNRPB,SNRPD1,SNRPD2,SNRPD3,SNRPE,SNRPF,SNRPG,SNRNP70,SNRPA,SNRPC,PRPF40B,PRPF40A,RBM25,DDX5,TCERG1,SNRPA1,SNRPB2,SF3A1,SF3A2,SF3A3,SF3B1,SF3B2,SF3B3,SF3B4,SF3B5,SF3B6,PHF5A,DDX42,U2AF1,U2AF1L5,U2AF1L4,U2AF2,PUF60,SMNDC1,RBM17,CHERP,U2SURP,RP9,LSM2,LSM3,LSM4,LSM5,LSM6,LSM7,LSM8,PRPF3,PRPF4,PPIH,PRPF31,SNU13,SNRNP27,USP39,SART1,ZMAT2,PRPF38A,PRPF38B,EFTUD2,SNRNP200,PRPF6,PRPF8,SNRNP40,DDX23,TXNL4A,PRPF19,CDC5L,BCAS2,PLRG1,CWC15,CTNNBL1,HSPA8,HSPA1A,HSPA2,HSPA1L,HSPA1B,HSPA6,PQBP1,WBP11,SNW1,XAB2,SYF2,CRNKL1,ISY1,ISY1-RAB43,PPIL1,PPIE,CCDC12,RBM22,BUD31,AQR,ACIN1,EIF4A3,RBM8A,MAGOH,MAGOHB,THOC1,THOC2,THOC3,ALYREF,NCBP1,NCBP2,HNRNPA3,HNRNPA1,HNRNPA1L2,HNRNPC,HNRNPCL1,RBMX,RBMXL1,RBMXL2,RBMXL3,HNRNPK,HNRNPM,HNRNPU,PCBP1,SRSF1,SRSF2,SRSF8,SRSF3,SRSF4,SRSF5,SRSF6,SRSF7,SRSF9,TRA2A,TRA2B,SRSF10",Spliceosome,135

hsa03050,"PSMD3,PSMD12,PSMD11,PSMD6,PSMD7,PSMD13,PSMD14,PSMD8,SEM1,PSMD4,PSMD2,PSMD1,ADRM1,PSMC2,PSMC1,PSMC5,PSMC6,PSMC3,PSMC4,PSME1,PSME2,PSME3,PSME4,PSMA6,PSMA2,PSMA4,PSMA7,PSMA8,PSMA5,PSMA1,PSMA3,PSMB6,PSMB7,PSMB3,PSMB2,PSMB5,PSMB1,PSMB4,PSMB9,PSMB10,PSMB8,PSMB11,IFNG,PSMF1,POMP",Proteasome,45

hsa03060,"OXA1L,SRP54,SEC61A1,SEC61A2,SEC61B,SEC61G,SEC62,SEC63,HSPA5,SRP9,SRP14,SRP72,SRP68,SRP19,SRPRA,SRPRB,SPCS1,SPCS2,SPCS3,SEC11C,SEC11A,IMMP1L,IMMP2L",Protein export,23

hsa03320,"CD36,SLC27A1,SLC27A4,SLC27A2,SLC27A5,SLC27A6,FABP1,FABP2,FABP3,FABP4,FABP5,FABP6,FABP7,PPARA,RXRA,RXRB,RXRG,PPARD,PPARG,HMGCS1,HMGCS2,APOA1,APOA2,APOC3,APOA5,PLTP,ME3,ME1,FADS2,SCD,SCD5,CYP7A1,CYP8B1,NR1H3,CYP27A1,DBI,LPL,ACSL6,ACSL4,ACSL1,ACSL5,ACSL3,ACSBG1,ACSBG2,OLR1,EHHADH,CYP4A11,CYP4A22,ACAA1,SCP2,ACOX3,ACOX1,ACOX2,CPT1A,CPT1B,CPT1C,CPT2,ACADL,ACADM,ANGPTL4,SORBS1,PLIN1,PLIN2,PLIN4,PLIN5,ADIPOQ,MMP1,UCP1,ILK,PDPK1,UBC,PCK1,PCK2,GK2,GK,AQP7",PPAR signaling pathway,76

hsa03410,"OGG1,NTHL1,NEIL1,NEIL2,NEIL3,UNG,SMUG1,MUTYH,MPG,MBD4,TDG,APEX1,APEX2,POLB,POLL,HMGB1,XRCC1,PCNA,POLD1,POLD2,POLD3,POLD4,POLE,POLE2,POLE3,POLE4,LIG1,LIG3,PARP2,PARP1,PARP3,PARP4,FEN1",Base excision repair,33

hsa03420,"RBX1,CUL4B,CUL4A,DDB1,DDB2,XPC,RAD23B,RAD23A,CETN2,ERCC8,ERCC6,CDK7,MNAT1,CCNH,ERCC3,ERCC2,GTF2H5,GTF2H1,GTF2H2,GTF2H2C_2,GTF2H2C,GTF2H3,GTF2H4,ERCC5,BIVM-ERCC5,XPA,RPA1,RPA2,RPA3,RPA4,ERCC4,ERCC1,POLD1,POLD2,POLD3,POLD4,POLE,POLE2,POLE3,POLE4,PCNA,RFC1,RFC4,RFC2,RFC5,RFC3,LIG1",Nucleotide excision repair,47

hsa03430,"SSBP1,PMS2,MLH1,MSH6,MSH2,MSH3,MLH3,RFC1,RFC4,RFC2,RFC5,RFC3,PCNA,EXO1,RPA1,RPA2,RPA3,RPA4,POLD1,POLD2,POLD3,POLD4,LIG1",Mismatch repair,23

hsa03440,"SSBP1,RAD50,MRE11,NBN,ATM,BRCA1,BARD1,RBBP8,BRIP1,TOPBP1,ABRAXAS1,UIMC1,BABAM1,BABAM2,BRCC3,PALB2,BRCA2,SEM1,SYCP3,RPA1,RPA2,RPA3,RPA4,RAD51,RAD52,RAD51B,RAD51C,RAD51D,XRCC2,XRCC3,RAD54L,RAD54B,POLD1,POLD2,POLD3,POLD4,BLM,TOP3A,TOP3B,MUS81,EME1",Homologous recombination,41

hsa03450,"XRCC6,XRCC5,DCLRE1C,PRKDC,POLL,POLM,DNTT,LIG4,XRCC4,NHEJ1,RAD50,MRE11,FEN1",Non-homologous end-joining,13

hsa03460,"ATRIP,ATR,FANCM,FAAP24,CENPS,CENPS-CORT,CENPX,TELO2,HES1,FAAP100,FANCA,FANCB,FANCC,FANCE,FANCF,FANCG,FANCL,WDR48,USP1,UBE2T,FANCI,FANCD2,BRCA2,PALB2,RAD51C,RAD51,BRCA1,BRIP1,FAN1,MLH1,PMS2,REV1,REV3L,POLH,POLI,POLK,POLN,RMI1,RMI2,TOP3A,TOP3B,BLM,RPA1,RPA2,RPA3,RPA4,MUS81,EME1,EME2,ERCC4,ERCC1,SLX1A,SLX1B,SLX4",Fanconi anemia pathway,54

hsa04010,"CACNA1A,CACNA1B,CACNA1C,CACNA1D,CACNA1E,CACNA1F,CACNA1G,CACNA1H,CACNA1I,CACNA1S,CACNA2D1,CACNA2D2,CACNA2D3,CACNA2D4,CACNB1,CACNB2,CACNB3,CACNB4,CACNG1,CACNG2,CACNG3,CACNG4,CACNG5,CACNG6,CACNG7,CACNG8,PRKACA,PRKACB,PRKACG,PRKCA,PRKCB,PRKCG,GNA12,GNG12,PPP3CA,PPP3CB,PPP3CC,PPP3R1,PPP3R2,RASGRF1,RASGRF2,RASGRP1,RASGRP2,RASGRP3,RASGRP4,RAPGEF2,NF1,RASA1,RASA2,RAP1A,RAP1B,EGF,TGFA,EREG,AREG,FGF1,FGF2,FGF3,FGF4,FGF17,FGF6,FGF7,FGF8,FGF9,FGF10,FGF16,FGF5,FGF18,FGF20,FGF22,FGF19,FGF21,FGF23,NGF,BDNF,NTF3,NTF4,INS,IGF1,IGF2,PDGFA,PDGFB,PDGFC,PDGFD,CSF1,KITLG,FLT3LG,VEGFA,VEGFB,PGF,VEGFC,VEGFD,HGF,ANGPT1,ANGPT2,ANGPT4,EFNA1,EFNA2,EFNA3,EFNA4,EFNA5,EGFR,ERBB2,ERBB3,ERBB4,FGFR1,FGFR2,FGFR3,FGFR4,NGFR,NTRK1,NTRK2,INSR,IGF1R,PDGFRA,PDGFRB,CSF1R,KIT,FLT3,FLT1,FLT4,KDR,MET,TEK,EPHA2,GRB2,SOS1,SOS2,HRAS,KRAS,NRAS,RRAS,RRAS2,MRAS,ARAF,BRAF,RAF1,MAP2K1,MAP2K2,LAMTOR3,MAPK1,MAPK3,MKNK1,MKNK2,RPS6KA3,RPS6KA1,RPS6KA2,RPS6KA6,ATF4,ELK1,ELK4,MYC,SRF,FOS,MAPT,STMN1,PLA2G4E,PLA2G4A,JMJD7-PLA2G4B,PLA2G4B,PLA2G4C,PLA2G4D,PLA2G4F,TNF,IL1A,IL1B,TGFB1,TGFB2,TGFB3,TNFRSF1A,IL1R1,IL1RAP,TGFBR1,TGFBR2,FASLG,FAS,CD14,RAC1,RAC2,RAC3,CDC42,TRADD,CASP3,TRAF2,DAXX,MYD88,IRAK1,IRAK4,TRAF6,GADD45A,GADD45B,GADD45G,TAB1,TAB2,ECSIT,MAP4K3,MAP4K4,MAP4K1,PAK1,PAK2,STK4,STK3,MAP4K2,MAP3K8,MAP3K1,MAP3K11,MAP3K2,MAP3K3,MAP3K13,MAP3K12,MAP3K20,MAP3K6,MAP3K5,MAP3K7,MAP3K4,TAOK2,TAOK3,TAOK1,MAP2K4,MAP2K7,MAP2K3,MAP2K6,MAPK8IP1,MAPK8IP2,MAPK8IP3,FLNA,FLNC,FLNB,CRK,CRKL,ARRB1,ARRB2,MAPK8,MAPK10,MAPK9,MAPK11,MAPK12,MAPK13,MAPK14,MAPKAPK5,MAPKAPK2,MAPKAPK3,RPS6KA5,RPS6KA4,CDC25B,NFATC1,NFATC3,JUN,JUND,ATF2,TP53,DDIT3,MAX,MEF2C,HSPB1,AKT1,AKT2,AKT3,PPM1A,PTPRR,PTPN5,PTPN7,DUSP1,DUSP4,DUSP2,DUSP7,DUSP8,DUSP5,DUSP16,DUSP6,DUSP9,DUSP10,DUSP3,PPP5C,PPP5D1,PPM1B,HSPA8,HSPA1A,HSPA2,HSPA1L,HSPA1B,HSPA6,MECOM,MAP2K5,MAPK7,NR4A1,MAP3K14,CHUK,IKBKB,IKBKG,NLK,NFKB1,NFKB2,RELA,RELB",MAPK signaling pathway,295

hsa04012,"EGF,TGFA,AREG,EGFR,ERBB2,PLCG1,PLCG2,CAMK2A,CAMK2D,CAMK2B,CAMK2G,PRKCA,PRKCB,PRKCG,CBL,CBLB,STAT5A,STAT5B,SRC,PTK2,CRK,CRKL,ABL1,ABL2,NCK1,NCK2,PAK1,PAK2,PAK3,PAK4,PAK5,PAK6,BUB1B-PAK6,MAP2K4,MAP2K7,MAPK8,MAPK10,MAPK9,JUN,ELK1,BTC,HBEGF,EREG,ERBB3,NRG1,NRG2,ERBB4,SHC1,SHC2,SHC3,SHC4,GRB2,SOS1,SOS2,HRAS,KRAS,NRAS,ARAF,BRAF,RAF1,MAP2K1,MAP2K2,MAPK1,MAPK3,MYC,GAB1,NRG3,NRG4,PIK3CA,PIK3CD,PIK3CB,PIK3R1,PIK3R2,PIK3R3,AKT1,AKT2,AKT3,MTOR,RPS6KB1,RPS6KB2,EIF4EBP1,BAD,GSK3B,CDKN1B,CDKN1A",ErbB signaling pathway,85

hsa04014,"EGF,TGFA,FGF1,FGF2,FGF3,FGF4,FGF17,FGF6,FGF7,FGF8,FGF9,FGF10,FGF16,FGF5,FGF18,FGF20,FGF22,FGF19,FGF21,FGF23,NGF,BDNF,NTF3,NTF4,INS,IGF1,IGF2,PDGFA,PDGFB,PDGFC,PDGFD,CSF1,KITLG,FLT3LG,VEGFA,VEGFB,PGF,VEGFC,VEGFD,HGF,ANGPT1,ANGPT2,ANGPT4,EFNA1,EFNA2,EFNA3,EFNA4,EFNA5,EGFR,FGFR1,FGFR2,FGFR3,FGFR4,NGFR,NTRK1,NTRK2,INSR,IGF1R,PDGFRA,PDGFRB,CSF1R,KIT,FLT3,FLT1,FLT4,KDR,MET,TEK,EPHA2,GRB2,GAB1,GAB2,SHC1,SHC2,SHC3,SHC4,PTPN11,SOS1,SOS2,PLCG1,PLCG2,RASGRP1,RASGRP2,RASGRP3,RASGRP4,ZAP70,LAT,HTR7,GNB1,GNB2,GNB3,GNB4,GNB5,GNG2,GNG3,GNG4,GNG5,GNG7,GNG8,GNG10,GNG11,GNG12,GNG13,GNGT1,GNGT2,PRKACA,PRKACB,PRKACG,RASGRF1,RASGRF2,GRIN1,GRIN2A,GRIN2B,CALML3,CALM2,CALM3,CALM1,CALML6,CALML5,CALML4,HRAS,KRAS,NRAS,MRAS,RRAS,RRAS2,NF1,RASA1,RASA2,RASA3,RASA4,RASA4B,SYNGAP1,RASAL1,RASAL2,RASAL3,RASSF1,RASSF5,STK4,TIAM1,RAC1,RAC2,RAC3,PAK1,PAK2,PAK3,PAK4,PAK5,PAK6,BUB1B-PAK6,RHOA,PIK3CA,PIK3CD,PIK3CB,PIK3R1,PIK3R2,PIK3R3,AKT1,AKT2,AKT3,IKBKG,CHUK,IKBKB,NFKB1,RELA,BAD,BCL2L1,FOXO4,FASLG,AFDN,SHOC2,RAF1,MAP2K1,MAP2K2,MAPK1,MAPK3,PLA1A,PLA2G10,PLA2G2D,PLA2G2E,PLA2G3,PLA2G2F,PLA2G12A,PLA2G12B,PLA2G1B,PLA2G5,PLA2G2A,PLA2G2C,PLA2G4E,PLA2G4A,JMJD7-PLA2G4B,PLA2G4B,PLA2G4C,PLA2G4D,PLA2G4F,PLA2G6,PLAAT3,ELK1,ETS1,ETS2,BRAP,KSR1,KSR2,RAPGEF5,RAP1A,RAP1B,RALGDS,RGL1,RGL2,RALA,RALB,MAPK8,MAPK10,MAPK9,EXOC2,TBK1,REL,PLD1,PLD2,RALBP1,CDC42,PLCE1,PRKCA,PRKCB,PRKCG,RIN1,ABL1,ABL2,RAB5A,RAB5B,RAB5C,ARF6",Ras signaling pathway,232

hsa04015,"RAP1A,RAP1B,MRAS,RAPGEF5,DOCK4,GRIN1,GRIN2A,GRIN2B,CALML3,CALM2,CALM3,CALM1,CALML6,CALML5,CALML4,F2R,F2RL3,P2RY1,FPR1,LPAR1,LPAR2,LPAR3,LPAR4,LPAR5,ADORA2A,ADORA2B,GNAS,ADCY1,ADCY2,ADCY3,ADCY4,ADCY5,ADCY6,ADCY7,ADCY8,ADCY9,RAPGEF3,RAPGEF4,GNAQ,PLCB1,PLCB2,PLCB3,PLCB4,RASGRP3,RASGRP2,EGF,FGF1,FGF2,FGF3,FGF4,FGF17,FGF6,FGF7,FGF8,FGF9,FGF10,FGF16,FGF5,FGF18,FGF20,FGF22,FGF19,FGF21,FGF23,NGF,INS,IGF1,PDGFA,PDGFB,PDGFC,PDGFD,CSF1,KITLG,VEGFA,VEGFB,PGF,VEGFC,VEGFD,HGF,ANGPT1,ANGPT2,ANGPT4,EFNA1,EFNA2,EFNA3,EFNA4,EFNA5,EGFR,FGFR1,FGFR2,FGFR3,FGFR4,NGFR,INSR,IGF1R,PDGFRA,PDGFRB,CSF1R,KIT,FLT1,FLT4,KDR,MET,TEK,EPHA2,CRK,CRKL,RAPGEF1,BCAR1,CDH1,CTNNB1,MAGI1,MAGI2,MAGI3,RAPGEF2,RAPGEF6,LAT,FYB1,LCP2,SKAP1,PLCG1,PRKCA,PRKCB,PRKCG,PRKD1,PRKD3,PRKD2,DRD2,CNR1,GNAI1,GNAI3,GNAI2,GNAO1,RAP1GAP,SIPA1L1,SIPA1,SIPA1L2,SIPA1L3,ID1,THBS1,RALGDS,RALA,RALB,RAC1,RAC2,RAC3,APBB1IP,TLN1,TLN2,ITGA2B,ITGB3,PFN3,PFN1,PFN2,PFN4,VASP,ENAH,EVL,ACTB,ACTG1,ARAP3,RHOA,SRC,FARP2,CDC42,VAV3,VAV1,VAV2,TIAM1,PARD3,PARD6A,PARD6G,PARD6B,PRKCZ,PRKCI,AFDN,CTNND1,KRIT1,RGS14,RASSF5,ITGAL,ITGAM,ITGB2,ITGB1,BRAF,RAF1,MAP2K1,MAP2K2,MAPK1,MAPK3,MAP2K3,MAP2K6,MAPK11,MAPK12,MAPK13,MAPK14,PIK3CA,PIK3CD,PIK3CB,PIK3R1,PIK3R2,PIK3R3,AKT1,AKT2,AKT3,PLCE1,HRAS,KRAS,NRAS,RRAS",Rap1 signaling pathway,210

hsa04020,"SLC8A1,SLC8A2,SLC8A3,ATP2B1,ATP2B3,ATP2B4,ATP2B2,CHRM1,CHRM3,CHRM5,ADORA2A,ADORA2B,ADRB1,ADRB2,ADRB3,DRD1,DRD5,HRH2,HTR4,HTR5A,HTR6,HTR7,GNAS,GNAL,ADCY1,ADCY2,ADCY3,ADCY4,ADCY7,ADCY8,ADCY9,PRKACA,PRKACB,PRKACG,PLN,ATP2A1,ATP2A3,ATP2A2,HRC,STIM1,STIM2,ORAI1,ORAI2,ORAI3,CACNA1C,CACNA1D,CACNA1F,CACNA1S,CACNA1A,CACNA1B,CACNA1E,CACNA1G,CACNA1H,CACNA1I,CHRNA7,P2RX1,P2RX2,P2RX3,P2RX4,P2RX5,P2RX7,P2RX6,GRIN1,GRIN2A,GRIN2C,GRIN2D,RYR1,RYR2,RYR3,TRDN,CASQ1,CASQ2,ASPH,CYSLTR1,CYSLTR2,CHRM2,ADRA1A,ADRA1B,ADRA1D,AGTR1,EDNRA,EDNRB,F2R,GRM1,GRM5,GRPR,HRH1,HTR2A,HTR2B,HTR2C,LHCGR,NTSR1,OXTR,AVPR1A,AVPR1B,LTB4R2,PTAFR,PTGER1,PTGER3,PTGFR,BDKRB1,BDKRB2,TACR1,TACR2,TACR3,TBXA2R,TRHR,CCKAR,CCKBR,CXCR4,EGFR,ERBB2,ERBB3,ERBB4,PDGFRA,PDGFRB,GNAQ,GNA11,GNA14,GNA15,LOC102723407,PLCD1,PLCD3,PLCD4,PLCB1,PLCB2,PLCB3,PLCB4,PLCG1,PLCG2,PLCE1,PLCZ1,ITPR1,ITPR2,ITPR3,CD38,TPCN1,TPCN2,SPHK1,SPHK2,MCU,VDAC1,VDAC2,VDAC3,SLC25A4,SLC25A5,SLC25A6,SLC25A31,PPIF,TNNC1,TNNC2,CALML3,CALM2,CALM3,CALM1,CALML6,CALML5,CALML4,PHKG1,PHKG2,PHKB,PHKA2,PHKA1,MYLK,MYLK2,MYLK3,MYLK4,CAMK1D,CAMK1G,CAMK1,CAMK2A,CAMK2D,CAMK2B,CAMK2G,CAMK4,PPP3CA,PPP3CB,PPP3CC,PPP3R1,PPP3R2,NOS1,NOS2,NOS3,PDE1A,PDE1B,PDE1C,PTK2B,ITPKB,ITPKA,ITPKC,PRKCA,PRKCB,PRKCG",Calcium signaling pathway,193

hsa04022,"AGTR1,EDNRA,EDNRB,ADRA1A,ADRA1B,ADRA1D,ADRA2A,ADRA2B,ADRA2C,GNAQ,GNA11,GNA12,GNA13,TRPC6,PPP3CA,PPP3CB,PPP3CC,PPP3R1,PPP3R2,MEF2A,BORCS8-MEF2B,MEF2B,MEF2C,MEF2D,NFATC1,NFATC2,NFATC3,NFATC4,SRF,GATA4,MYH7,MYH6,NPPB,NPPA,NPR1,NPPC,NPR2,PRKG1,PRKG2,CACNA1C,CACNA1D,CACNA1F,CACNA1S,KCNMA1,KCNU1,KCNMB1,KCNMB2,KCNMB3,KCNMB4,SLC8A1,SLC8A2,SLC8A3,ATP1A1,ATP1A2,ATP1A3,ATP1A4,ATP1B4,ATP1B1,ATP1B2,ATP1B3,FXYD2,ATP2B1,ATP2B3,ATP2B4,ATP2B2,RGS2,GTF2IRD1,GTF2I,PLCB1,PLCB2,PLCB3,PLCB4,ITPR1,ITPR2,ITPR3,MRVI1,PLN,ATP2A1,ATP2A3,ATP2A2,RHOA,ROCK1,ROCK2,PPP1R12A,PPP1CA,PPP1CB,PPP1CC,CALML3,CALM2,CALM3,CALM1,CALML6,CALML5,CALML4,MYLK,MYLK2,MYLK3,MYLK4,MYL9,CNGA1,CNGB1,ADCY1,ADCY2,ADCY3,ADCY4,ADCY5,ADCY6,ADCY7,ADCY8,ADCY9,PDE2A,PDE3A,PDE3B,PDE5A,GUCY1A2,GUCY1A1,GUCY1B1,KNG1,BDKRB2,OPRD1,ADORA1,ADORA3,GNAI1,GNAI3,GNAI2,INS,INSR,IRS1,IRS2,IRS4,PIK3CG,PIK3R5,PIK3R6,AKT1,AKT2,AKT3,NOS3,ADRB1,ADRB2,ADRB3,PRKCE,KCNJ8,VDAC1,VDAC2,VDAC3,SLC25A4,SLC25A5,SLC25A6,SLC25A31,PPIF,CREB1,ATF2,ATF4,CREB3,CREB3L1,CREB3L2,CREB3L3,CREB3L4,CREB5,ATF6B,BAD,VASP,RAF1,MAP2K1,MAP2K2,MAPK1,MAPK3",cGMP-PKG signaling pathway,167

hsa04024,"ADCYAP1,VIP,CGA,TSHB,POMC,GCG,GIP,FSHB,NPPA,ADRB1,DRD1,DRD5,ADORA2A,HTR4,HTR6,PTGER2,ADCYAP1R1,VIPR2,TSHR,MC2R,GLP1R,GIPR,GPR119,FSHR,NPR1,GNAS,EDN1,EDN2,EDN3,SST,NPY,GHRL,OXT,ADRB2,HTR1A,HTR1B,HTR1D,HTR1E,HTR1F,CHRM1,CHRM2,DRD2,GABBR1,GABBR2,ADORA1,EDNRA,NPY1R,SSTR1,SSTR2,SSTR5,HCAR1,HCAR2,HCAR3,FFAR2,SUCNR1,PTGER3,OXTR,GHSR,GNAI1,GNAI3,GNAI2,ADCY1,ADCY2,ADCY3,ADCY4,ADCY5,ADCY6,ADCY7,ADCY8,ADCY9,ADCY10,HCN2,HCN4,CNGA1,CNGA2,CNGA3,CNGA4,CNGB1,CNGB3,CALML3,CALM2,CALM3,CALM1,CALML6,CALML5,CALML4,CAMK2A,CAMK2D,CAMK2B,CAMK2G,CAMK4,ABCC4,RAPGEF3,RAPGEF4,RRAS,RRAS2,PLD1,PLD2,PLCE1,MAPK8,MAPK10,MAPK9,RAP1A,RAP1B,TIAM1,VAV3,VAV1,VAV2,RAC1,RAC2,RAC3,PAK1,ARAP3,RHOA,AFDN,PIK3CA,PIK3CD,PIK3CB,PIK3R1,PIK3R2,PIK3R3,AKT1,AKT2,AKT3,BRAF,RAF1,MAP2K1,MAP2K2,MAPK1,MAPK3,PRKACA,PRKACB,PRKACG,PPP1R1B,PPP1CA,PPP1CB,PPP1CC,CREB1,CREB3,CREB3L1,CREB3L2,CREB3L3,CREB3L4,CREB5,CREBBP,EP300,BDNF,FOS,JUN,GLI3,GLI1,PTCH1,HHIP,NFKBIA,NFKB1,RELA,SOX9,AMH,PPARA,ACOX3,ACOX1,NFATC1,F2R,BAD,LIPE,ROCK1,ROCK2,PPP1R12A,MYL9,TNNI3,PLN,ATP2A1,ATP2A3,ATP2A2,RYR2,GRIN1,GRIN2A,GRIN2B,GRIN2C,GRIN2D,GRIN3A,GRIN3B,GRIA1,GRIA2,GRIA3,GRIA4,CFTR,ATP1A1,ATP1A2,ATP1A3,ATP1A4,ATP1B4,ATP1B1,ATP1B2,ATP1B3,FXYD2,FXYD1,SLC9A1,ORAI1,ATP2B1,ATP2B3,ATP2B4,ATP2B2,CACNA1C,CACNA1D,CACNA1F,CACNA1S,PDE3A,PDE3B,PDE4A,PDE4B,PDE4C,PDE4D,PDE10A",cAMP signaling pathway,214

hsa04060,"CCL1,CCL25,CCL19,CCL21,CCL3,CCL3L1,CCL3L3,CCL4,CCL4L2,CCL4L1,CCL17,CCL22,CCL5,CCL8,CCL14,CCL16,CCL15,CCL23,CCL13,CCL7,CCL2,CCL11,CCL24,CCL26,CCL27,CCL28,CCL18,CCL20,CXCL1,CXCL2,CXCL3,CXCL5,CXCL6,CXCL8,PPBP,PF4,PF4V1,CXCL9,CXCL10,CXCL11,CXCL13,CXCL12,CXCL16,CXCL14,CXCL17,XCL1,XCL2,CX3CL1,IL2,IL4,IL7,IL9,IL15,IL21,TSLP,IL3,IL5,CSF2,IL13,EPO,GH1,GH2,CSH1,CSH2,PRL,THPO,CSF3,LEP,IL6,IL11,IL12A,IL12B,IL23A,IL27,IL31,CLCF1,CNTF,CTF1,LIF,OSM,IL10,IL19,IL20,IL24,IL22,IL26,IFNL2,IFNL3,IFNL1,IFNA1,IFNA2,IFNA4,IFNA5,IFNA6,IFNA7,IFNA8,IFNA10,IFNA13,IFNA14,IFNA16,IFNA17,IFNA21,IFNB1,IFNW1,IFNK,IFNE,IFNG,IL1A,IL1B,IL1RN,IL36RN,IL36A,IL36B,IL36G,IL1F10,IL37,IL18,IL33,IL17A,IL17F,IL17B,IL17C,IL17D,IL25,IL16,IL32,IL34,CSF1,TNF,LTA,LTB,TNFSF14,FASLG,TNFSF15,TNFSF10,EDA,NGF,TNFSF11,TNFSF12,CD70,TNFSF8,CD40LG,TNFSF9,TNFSF4,TNFSF18,TNFSF13,TNFSF13B,TGFB1,TGFB2,TGFB3,GDF15,GDF2,BMP10,INHA,BMP3,GDF10,GDF11,MSTN,INHBA,INHBB,GDF1,GDF3,NODAL,GDF9,INHBC,INHBE,AMH,BMP2,BMP4,GDF5,GDF6,GDF7,BMP15,BMP5,BMP6,BMP7,BMP8B,BMP8A,CCR8,CCR9,ACKR4,CCR7,CCR4,CCR5,CCR3,CCR2,CCR1,CCR10,CCR6,CXCR1,CXCR2,CXCR3,CXCR5,CXCR4,ACKR3,CXCR6,XCR1,CX3CR1,IL2RA,IL2RB,IL2RG,IL4R,IL7R,IL9R,IL15RA,IL21R,CRLF2,IL3RA,CSF2RB,IL5RA,CSF2RA,IL13RA1,IL13RA2,EPOR,GHR,PRLR,MPL,CSF3R,LEPR,IL6R,IL6ST,IL11RA,IL12RB1,IL12RB2,IL23R,IL27RA,IL31RA,CNTFR,LIFR,OSMR,IL10RA,IL10RB,IL20RA,IL20RB,IL22RA1,IFNLR1,IFNAR1,IFNAR2,IFNGR1,IFNGR2,IL1R1,IL1RAP,IL1R2,IL1RL2,IL18R1,IL18RAP,IL1RL1,IL17RA,IL17RC,IL17RB,IL17RE,CD4,CSF1R,TNFRSF1A,TNFRSF1B,LTBR,TNFRSF14,TNFRSF6B,FAS,TNFRSF25,TNFRSF10A,TNFRSF10B,TNFRSF10C,TNFRSF10D,TNFRSF21,EDAR,EDA2R,NGFR,TNFRSF11B,TNFRSF11A,TNFRSF12A,CD27,TNFRSF8,CD40,TNFRSF9,TNFRSF4,TNFRSF18,TNFRSF17,TNFRSF13B,TNFRSF13C,TNFRSF19,RELT,TGFBR1,TGFBR2,ACVRL1,ACVR2A,BMPR2,ACVR2B,ACVR1B,ACVR1C,ACVR1,AMHR2,BMPR1A,BMPR1B",Cytokine-cytokine receptor interaction,294

hsa04061,"CCL5,CCL3,CCL3L1,CCL3L3,CCL8,CCL14,CCL16,CCL15,CCL23,CCL13,CCL7,CCL2,CCL27,CCL11,CCL24,CCL26,CCL28,CCL17,CCL22,CCL4,CCL4L2,CCL4L1,CCL20,CCL1,CCL25,CCL18,CCL19,CCL21,CXCL1,CXCL2,CXCL3,CXCL5,CXCL6,CXCL8,PPBP,PF4,PF4V1,CXCL9,CXCL10,CXCL11,CXCL13,CXCL12,CXCL14,XCL1,XCL2,CX3CL1,IL10,IL6,IL2,IL18,IL37,IL19,IL20,IL24,TNF,LTA,TNFSF14,TNFSF10,IL34,CSF1,CCR1,CCR2,CCR3,CCR4,CCR5,CCR6,CCR8,CCR9,ACKR4,CCR10,CCR7,CXCR1,CXCR2,CXCR3,CXCR5,CXCR4,ACKR3,XCR1,CX3CR1,IL10RA,IL10RB,IL6R,IL6ST,IL2RA,IL2RB,IL2RG,IL18R1,IL18RAP,IL20RA,IL20RB,IL22RA1,TNFRSF1A,TNFRSF1B,LTBR,TNFRSF14,TNFRSF10A,TNFRSF10B,TNFRSF10C,TNFRSF10D,CSF1R",Viral protein interaction with cytokine and cytokine receptor,100

hsa04062,"CXCL1,CXCL2,CXCL3,CXCL5,CXCL6,PPBP,CXCL8,CXCL9,CXCL10,CXCL11,CXCL12,CXCL13,CXCL16,PF4,PF4V1,CXCL14,XCL1,XCL2,CX3CL1,CCL1,CCL2,CCL3,CCL3L1,CCL3L3,CCL4,CCL4L2,CCL4L1,CCL5,CCL7,CCL8,CCL11,CCL13,CCL14,CCL15,CCL23,CCL16,CCL17,CCL18,CCL19,CCL20,CCL21,CCL22,CCL24,CCL25,CCL26,CCL27,CCL28,CXCR2,CXCR1,CXCR3,CXCR4,CXCR5,CXCR6,XCR1,CX3CR1,CCR8,CCR6,CCR9,CCR4,CCR7,CCR2,CCR5,CCR1,CCR3,CCR10,JAK2,JAK3,STAT1,STAT2,STAT3,STAT5B,GNAI1,GNAI3,GNAI2,ADCY1,ADCY2,ADCY3,ADCY4,ADCY5,ADCY6,ADCY7,ADCY8,ADCY9,PRKACA,PRKACB,PRKACG,LYN,HCK,FGR,SRC,SHC1,SHC2,SHC3,SHC4,GRB2,SOS1,SOS2,HRAS,KRAS,NRAS,RAF1,BRAF,MAP2K1,MAPK1,MAPK3,PIK3CA,PIK3CD,PIK3CB,PIK3R1,PIK3R2,PIK3R3,PIK3CG,PIK3R5,PIK3R6,PRKCZ,AKT1,AKT2,AKT3,FOXO3,CHUK,IKBKB,IKBKG,NFKBIA,NFKBIB,NFKB1,RELA,BAD,GSK3A,GSK3B,ITK,VAV3,VAV1,VAV2,RAC1,RAC2,RAC3,PAK1,CDC42,WAS,WASL,RHOA,ROCK1,ROCK2,GNB1,GNB2,GNB3,GNB4,GNB5,GNG2,GNG3,GNG4,GNG5,GNG7,GNG8,GNG10,GNG11,GNG12,GNG13,GNGT1,GNGT2,PREX1,ELMO1,DOCK2,PTK2,PXN,BCAR1,CRK,CRKL,PTK2B,PLCB1,PLCB2,PLCB3,PLCB4,RASGRP2,RAP1A,RAP1B,PARD3,TIAM1,PRKCB,PRKCD,NCF1,GRK7,GRK1,GRK2,GRK3,GRK4,GRK5,GRK6,ARRB1,ARRB2",Chemokine signaling pathway,190

hsa04064,"LCK,ZAP70,LAT,PLCG1,PRKCQ,LOC102723407,SYK,LYN,BLNK,BTK,PLCG2,PRKCB,CARD10,CARD11,CARD14,BCL10,MALT1,IL1B,IL1R1,MYD88,IRAK1,IRAK4,TRAF6,TNF,TNFRSF1A,RIPK1,TRADD,TRAF2,TRAF5,BIRC2,BIRC3,EDA,EDAR,EDARADD,CYLD,EDA2R,DDX58,TRIM25,LBP,CD14,TLR4,LY96,TIRAP,TICAM2,TICAM1,CD40LG,CD40,TRAF3,TNFSF11,TNFRSF11A,LTA,LTB,TNFSF14,LTBR,MAP3K14,MAP3K7,TAB1,TAB2,TAB3,TNFSF13B,TNFRSF13C,IKBKG,CHUK,IKBKB,PIAS4,UBE2I,ATM,PIDD1,ERC1,NFKBIA,NFKB1,RELA,CFLAR,XIAP,BCL2L1,BCL2,TRAF1,BCL2A1,NFKB2,CXCL8,TNFAIP3,PTGS2,CCL4,CCL4L2,CCL4L1,VCAM1,PLAU,CSNK2A1,CSNK2A2,CSNK2A3,CSNK2B,RELB,CCL13,CCL19,CCL21,CXCL12,ICAM1,PARP1,CXCL2,GADD45B",NF-kappa B signaling pathway,100

hsa04066,"IL6,IL6R,STAT3,TLR4,IFNG,IFNGR1,IFNGR2,RELA,NFKB1,INS,EGF,IGF1,INSR,EGFR,IGF1R,ERBB2,MAP2K1,MAP2K2,MAPK1,MAPK3,MKNK1,MKNK2,PIK3CA,PIK3CD,PIK3CB,PIK3R1,PIK3R2,PIK3R3,AKT1,AKT2,AKT3,MTOR,EIF4EBP1,EIF4E,EIF4E2,EIF4E1B,RPS6KB1,RPS6KB2,RPS6,HIF1A,VHL,RBX1,ELOC,ELOB,CUL2,EGLN1,EGLN3,EGLN2,ARNT,CREBBP,EP300,CYBB,PLCG1,PLCG2,PRKCA,PRKCB,PRKCG,CAMK2A,CAMK2D,CAMK2B,CAMK2G,TIMP1,LTBR,EPO,TF,TFRC,VEGFA,FLT1,SERPINE1,ANGPT1,ANGPT2,ANGPT4,TEK,EDN1,NOS2,NOS3,HMOX1,NPPA,SLC2A1,PDK1,HK3,HK1,HK2,HKDC1,PFKM,PFKP,PFKL,GAPDH,ALDOC,ALDOA,ALDOB,ENO3,ENO2,ENO1,ENO4,PGK2,PGK1,PFKFB3,LDHAL6A,LDHAL6B,LDHA,LDHB,LDHC,BCL2,CDKN1A,CDKN1B,PDHA2,PDHA1,PDHB",HIF-1 signaling pathway,109

hsa04068,"TGFB1,TGFB2,TGFB3,TGFBR1,TGFBR2,SMAD4,SMAD2,SMAD3,STK11,PRKAA1,PRKAA2,PRKAB1,PRKAB2,PRKAG1,PRKAG3,PRKAG2,NLK,SKP2,MDM2,SETD7,CREBBP,EP300,SIRT1,USP7,SLC2A4,MAPK8,MAPK10,MAPK9,STK4,IGF1,IGF1R,INS,INSR,IRS1,IRS2,IRS4,PIK3CA,PIK3CD,PIK3CB,PIK3R1,PIK3R2,PIK3R3,PDPK1,CHUK,IKBKB,PTEN,AKT1,AKT2,AKT3,SGK1,SGK2,SGK3,C8orf44-SGK3,FOXO1,FOXO3,FOXO4,FOXO6,GRM1,HOMER1,HOMER2,HOMER3,AGAP2,CSNK1E,TPTEP2-CSNK1E,IL6,IL10,STAT3,EGF,EGFR,GRB2,SOS1,SOS2,HRAS,KRAS,NRAS,BRAF,RAF1,ARAF,MAP2K1,MAP2K2,MAPK1,MAPK3,MAPK11,MAPK12,MAPK13,MAPK14,CDK2,PRMT1,FOXG1,CCNB1,CCNB2,CCNB3,CCND1,CCND2,CCNG2,CDKN2B,CDKN2D,CDKN1A,CDKN1B,RBL2,PLK1,PLK2,PLK3,PLK4,GADD45A,GADD45B,GADD45G,FASLG,BCL2L11,TNFSF10,BCL6,BNIP3,ATG12,GABARAP,GABARAPL1,GABARAPL2,CAT,SOD2,ATM,PCK1,PCK2,G6PC,G6PC2,G6PC3,IL7R,KLF2,S1PR1,S1PR4,RAG1,RAG2,FBXO25,FBXO32",FoxO signaling pathway,132

hsa04070,"PI4KA,PI4KB,PI4K2A,PI4K2B,SACM1L,INPP5F,PIP5K1C,PIP5K1A,PIP5K1B,OCRL,INPP5B,INPP5E,SYNJ1,SYNJ2,PIK3CA,PIK3CD,PIK3CB,PIK3R1,PIK3R2,PIK3R3,PTEN,INPP5D,INPPL1,INPP4A,INPP4B,PIKFYVE,MTM1,MTMR1,MTMR2,MTMR3,MTMR4,MTMR8,MTMR6,MTMR7,MTMR14,PIP4K2C,PIP4K2A,PIP4K2B,PIP4P1,PIP4P2,PIK3C3,PIK3C2G,PIK3C2A,PIK3C2B,PLCB1,PLCB2,PLCB3,PLCB4,PLCD1,PLCD3,PLCD4,PLCE1,PLCG1,PLCG2,PLCZ1,IPMK,ITPKB,ITPKA,ITPKC,INPP5K,INPP5J,INPP5A,ITPK1,IPPK,IP6K1,IP6K2,IP6K3,PPIP5K1,PPIP5K2,INPP1,IMPA2,IMPA1,IMPAD1,DGKZ,DGKD,DGKI,DGKA,DGKE,DGKB,DGKH,DGKG,DGKQ,DGKK,CDS1,CDS2,CDIPT,ITPR1,ITPR2,ITPR3,PRKCA,PRKCB,PRKCG,CALML3,CALM2,CALM3,CALM1,CALML6,CALML5,CALML4",Phosphatidylinositol signaling system,99

hsa04071,"SPTLC1,SPTLC2,SPTLC3,CERS2,CERS3,CERS6,CERS1,CERS4,CERS5,DEGS1,DEGS2,SMPD1,SGMS1,SGMS2,TNF,TNFRSF1A,NSMAF,SMPD2,ASAH1,ASAH2,ACER2,ACER1,TRADD,TRAF2,CTSD,BID,BAX,MAP3K5,MAPK8,MAPK10,MAPK9,MAPK11,MAPK12,MAPK13,MAPK14,PRKCZ,AKT1,AKT2,AKT3,BCL2,PPP2CA,PPP2CB,PPP2R1B,PPP2R1A,PPP2R2A,PPP2R2B,PPP2R2C,PPP2R2D,PPP2R3B,PPP2R3C,PPP2R3A,PPP2R5B,PPP2R5C,PPP2R5D,PPP2R5E,PPP2R5A,TP53,FCER1A,MS4A2,FCER1G,FYN,GAB2,PIK3CA,PIK3CD,PIK3CB,PIK3R1,PIK3R2,PIK3R3,PLD1,PLD2,SPHK1,SPHK2,KNG1,ADORA1,ADORA3,BDKRB2,OPRD1,GNAI1,GNAI3,GNAI2,PLCB1,PLCB2,PLCB3,PLCB4,PRKCE,MAPK1,MAPK3,SGPP1,SGPP2,ABCC1,SGPL1,S1PR1,S1PR2,HRAS,KRAS,NRAS,RAF1,MAP2K1,MAP2K2,PDPK1,NOS3,RAC1,RAC2,RAC3,S1PR3,GNAQ,PRKCA,PRKCB,PRKCG,S1PR4,S1PR5,GNA12,GNA13,RHOA,ROCK1,ROCK2,PTEN,NFKB1,RELA",Sphingolipid signaling pathway,119

hsa04072,"EGF,PDGFA,PDGFB,PDGFC,PDGFD,KITLG,INS,EGFR,PDGFRA,PDGFRB,KIT,INSR,GRB2,GAB1,GAB2,SHC1,SHC2,SHC3,SHC4,PTPN11,SOS1,SOS2,HRAS,KRAS,NRAS,MRAS,RRAS,RRAS2,RALGDS,RALA,RALB,PIK3CA,PIK3CD,PIK3CB,PIK3R1,PIK3R2,PIK3R3,AKT1,AKT2,AKT3,TSC1,TSC2,RHEB,MTOR,RHOA,ARF1,ARF6,PLCG1,PLCG2,LOC102723407,FCER1A,MS4A2,FCER1G,FYN,SYK,PRKCA,PLD1,PLD2,PLA2G4E,PLA2G4A,JMJD7-PLA2G4B,PLA2G4B,PLA2G4C,PLA2G4D,PLA2G4F,AGPAT1,AGPAT2,AGPAT3,AGPAT4,AGPAT5,SPHK1,SPHK2,RAF1,MAP2K1,MAP2K2,MAPK1,MAPK3,PIP5K1C,PIP5K1A,PIP5K1B,DGKZ,DGKD,DGKI,DGKA,DGKE,DGKB,DGKH,DGKG,DGKQ,DGKK,PLPP1,PLPP3,PLPP2,F2,AGT,AVP,CXCL8,AGTR1,F2R,GRM1,GRM2,GRM3,GRM4,GRM5,GRM6,GRM7,GRM8,AVPR1A,AVPR1B,AVPR2,LPAR1,LPAR2,LPAR3,LPAR4,LPAR5,LPAR6,PTGFR,CXCR1,CXCR2,PLCB1,PLCB2,PLCB3,PLCB4,GNA12,PTK2B,GNA13,PIK3CG,PIK3R5,PIK3R6,CYTH3,CYTH4,CYTH2,CYTH1,GNAS,ADCY1,ADCY2,ADCY3,ADCY4,ADCY5,ADCY6,ADCY7,ADCY8,ADCY9,RAPGEF3,RAPGEF4,DNM1,DNM3,DNM2",Phospholipase D signaling pathway,148

hsa04080,"CHRM1,CHRM2,CHRM3,CHRM4,CHRM5,ADRA1A,ADRA1B,ADRA1D,ADRA2A,ADRA2B,ADRA2C,ADRB1,ADRB2,ADRB3,DRD1,DRD2,DRD3,DRD4,DRD5,HRH1,HRH2,HRH3,HRH4,HTR1A,HTR1B,HTR1D,HTR1E,HTR1F,HTR2A,HTR2B,HTR2C,HTR4,HTR5A,HTR6,HTR7,TAAR9,TAAR6,TAAR1,TAAR8,TAAR5,TAAR2,AGT,AGTR1,AGTR2,APLN,APLNR,NMB,GRP,NMBR,GRPR,BRS3,KNG1,BDKRB1,BDKRB2,C3,C3AR1,C5,C5AR1,FPR1,FPR3,FPR2,CCK,CCKAR,CCKBR,EDN1,EDN2,EDN3,EDNRA,EDNRB,GAL,GALR1,GALR2,GALR3,GHRL,GHSR,KISS1,KISS1R,POMC,MC1R,MC2R,MC3R,MC4R,MC5R,MLN,MLNR,NMU,NMUR1,NMUR2,NPFF,NPFFR1,NPFFR2,NPY,PYY,PPY,NPY1R,NPY2R,NPY4R,NPY4R2,NPY5R,GPR83,NPW,NPB,NPBWR1,NPBWR2,NTS,NTSR1,NTSR2,PENK,PDYN,OPRD1,OPRK1,OPRM1,OPRL1,HCRT,HCRTR1,HCRTR2,OXT,OXTR,SST,CORT,SSTR1,SSTR2,SSTR3,SSTR4,SSTR5,TAC1,TAC4,TACR1,TACR2,TAC3,TACR3,UTS2,UTS2R,AVP,AVPR1A,AVPR1B,AVPR2,F2,CTSG,GZMA,PLG,PRSS3,PRSS2,PRSS1,F2R,F2RL1,F2RL2,F2RL3,PARD3,PRLH,PRLHR,PMCH,MCHR1,MCHR2,FSHB,CGA,FSHR,LHB,LHCGR,TSHB,TSHR,PTGDR,PTGER1,PTGER2,PTGER3,PTGER4,PTGFR,PTGIR,TBXA2R,ADORA1,ADORA2A,ADORA2B,ADORA3,P2RY2,P2RY1,P2RY4,P2RY6,LPAR6,P2RY10,P2RY14,P2RY8,P2RY11,P2RY13,GPR35,CNR1,CNR2,PTAFR,GNRH1,GNRH2,GNRHR,TRH,TRHR,MTNR1A,MTNR1B,GPR50,LPAR1,LPAR2,LPAR3,LPAR4,S1PR1,S1PR2,S1PR3,S1PR4,S1PR5,LTB4R,LTB4R2,MAS1,RLN1,RLN2,RLN3,INSL3,INSL5,RXFP1,RXFP2,RXFP3,RXFP4,CYSLTR1,CYSLTR2,CALCA,CALCB,ADM,IAPP,CALCR,CALCRL,CRH,UCN,UCN3,UCN2,CRHR1,CRHR2,GIP,GIPR,GCG,GCGR,GLP1R,GLP2R,GHRH,GHRHR,PTH,PTH2,PTH1R,PTH2R,ADCYAP1,ADCYAP1R1,SCT,SCTR,VIP,VIPR1,VIPR2,GRM1,GRM2,GRM3,GRM4,GRM5,GRM6,GRM7,GRM8,GABBR1,GABBR2,GPR156,GRIN1,GRIN2A,GRIN2B,GRIN2C,GRIN2D,GRIN3A,GRIN3B,GABRA1,GABRA2,GABRA3,GABRA4,GABRA5,GABRA6,GABRB1,GABRB3,GABRB2,GABRD,GABRE,GABRG1,GABRG2,GABRG3,GABRP,GABRR1,GABRR2,GABRR3,GABRQ,CHRNA1,CHRNA2,CHRNA3,CHRNA4,CHRNA5,CHRNA6,CHRNA7,CHRNA9,CHRNA10,CHRNB1,CHRNB2,CHRNB3,CHRNB4,CHRND,CHRNE,CHRNG,P2RX1,P2RX2,P2RX3,P2RX4,P2RX5,P2RX7,P2RX6,GRIA1,GRIA2,GRIA3,GRIA4,GRIK1,GRIK2,GRIK3,GRIK4,GRIK5,GRID1,GRID2,GLRA1,GLRA2,GLRA3,GLRB,TRPV1,TSPO,NR3C1,GH1,GH2,CSH1,CSH2,GHR,THRA,THRB,LEP,LEPR,PRL,PRLR",Neuroactive ligand-receptor interaction,338

hsa04110,"CCND1,CCND2,CCND3,CDK4,CDK6,RB1,RBL1,RBL2,ABL1,HDAC1,HDAC2,E2F1,E2F2,E2F3,E2F4,E2F5,TFDP1,TFDP2,GSK3B,TGFB1,TGFB2,TGFB3,SMAD2,SMAD3,SMAD4,MYC,ZBTB17,CDKN2A,CDKN2B,CDKN2C,CDKN2D,CDKN1B,CDKN1C,CDKN1A,CCNE1,CCNE2,CDK2,SKP1,CUL1,RBX1,SKP2,CCNA2,CCNA1,CDC6,CDC45,CDC7,DBF4,CDK1,CCNB1,CCNB2,CCNB3,CDC25B,CDC25C,YWHAZ,YWHAB,YWHAQ,YWHAE,YWHAH,YWHAG,PLK1,WEE1,WEE2,PKMYT1,CCNH,CDK7,ANAPC1,ANAPC2,CDC27,ANAPC4,ANAPC5,CDC16,ANAPC7,CDC23,ANAPC10,ANAPC11,CDC26,ANAPC13,CDC20,PTTG1,PTTG2,ESPL1,SMC1A,SMC1B,SMC3,STAG2,STAG1,RAD21,TTK,BUB1,BUB3,BUB1B,MAD1L1,MAD2L1,MAD2L2,FZR1,CDC14B,CDC14A,ATR,ATM,TP53,CHEK1,CHEK2,CREBBP,EP300,PRKDC,MDM2,GADD45A,GADD45B,GADD45G,PCNA,SFN,CDC25A,ORC1,ORC2,ORC3,ORC4,ORC5,ORC6,MCM2,MCM3,MCM4,MCM5,MCM6,MCM7",Cell cycle,124

hsa04114,"INS,IGF1,IGF1R,PGR,AR,ADCY1,ADCY2,ADCY3,ADCY4,ADCY5,ADCY6,ADCY7,ADCY8,ADCY9,PRKACA,PRKACB,PRKACG,AURKA,CPEB1,CPEB2,CPEB3,CPEB4,MOS,MAP2K1,MAPK1,MAPK3,RPS6KA3,RPS6KA1,RPS6KA2,RPS6KA6,PKMYT1,YWHAZ,YWHAB,YWHAQ,YWHAE,YWHAH,YWHAG,SPDYA,SPDYC,SPDYE2B,SPDYE18,SPDYE11,SPDYE16,SPDYE17,SPDYE1,SPDYE4,SPDYE3,SPDYE2,SPDYE5,SPDYE6,CDK1,CDK2,MAPK11,MAPK12,MAPK13,MAPK14,PPP1CA,PPP1CB,PPP1CC,SLK,PLK1,CDC25C,CCNB2,RBX1,CUL1,SKP1,BTRC,FBXW11,FBXO5,ANAPC1,ANAPC2,CDC27,ANAPC4,ANAPC5,CDC16,ANAPC7,CDC23,ANAPC10,ANAPC11,CDC26,ANAPC13,CDC20,FBXO43,PTTG1,PTTG2,ESPL1,REC8,STAG3,SMC1A,SMC1B,SMC3,CCNB1,BUB1,MAD2L1,MAD2L2,MAD1L1,CCNE1,CCNE2,SGO1,PPP2R5B,PPP2R5C,PPP2R5D,PPP2R5E,PPP2R5A,PPP2R1B,PPP2R1A,PPP2CA,PPP2CB,PLCZ1,ITPR1,ITPR2,ITPR3,CALML3,CALM2,CALM3,CALM1,CALML6,CALML5,CALML4,PPP3CA,PPP3CB,PPP3CC,PPP3R1,PPP3R2,CAMK2A,CAMK2D,CAMK2B,CAMK2G",Oocyte meiosis,128

hsa04115,"ATM,CHEK2,ATR,CHEK1,GORAB,CDKN2A,MDM2,MDM4,TP53,CDKN1A,CCND1,CCND2,CCND3,CDK4,CDK6,CCNE1,CCNE2,CDK2,SFN,RPRM,CCNB1,CCNB2,CDK1,GADD45A,GADD45B,GADD45G,GTSE1,FAS,PIDD1,CASP8,BID,BAX,PMAIP1,BBC3,TP53AIP1,SIVA1,BCL2L1,BCL2,TP53I3,EI24,SHISA5,PERP,ZMAT3,SIAH1,CYCS,APAF1,CASP9,CASP3,AIFM2,IGFBP3,IGF1,SERPINE1,ADGRB1,CD82,THBS1,SERPINB5,DDB2,RRM2B,RRM2,SESN1,SESN3,SESN2,PTEN,TSC2,STEAP3,COP1,RCHY1,CCNG1,CCNG2,PPM1D,TP73,TNFRSF10B",p53 signaling pathway,72

hsa04120,"UBA1,SAE1,UBA2,UBA3,UBA7,UBA6,UBE2A,UBE2B,UBE2C,UBE2D4,UBE2D1,UBE2D2,UBE2D3,UBE2E3,UBE2E1,UBE2E2,UBE2F,UBE2G1,UBE2G2,UBE2H,UBE2I,UBE2J1,UBE2J2,UBE2L3,UBE2L6,UBE2M,UBE2NL,UBE2N,UBE2O,UBE2Q1,UBE2Q2,UBE2QL1,UBE2R2,CDC34,UBE2S,UBE2U,UBE2W,UBE2Z,UBE2K,BIRC6,UBE3A,UBE3B,UBE3C,SMURF1,SMURF2,ITCH,WWP1,WWP2,TRIP12,NEDD4,NEDD4L,HUWE1,UBR5,HERC1,HERC2,HERC3,HERC4,UBE4A,UBE4B,STUB1,PPIL2,PRPF19,UBOX5,MDM2,CBL,CBLB,CBLC,PRKN,SIAH1,PML,TRAF6,MAP3K1,COP1,RCHY1,BIRC2,BIRC3,XIAP,BIRC7,BIRC8,PIAS1,PIAS2,PIAS3,PIAS4,SYVN1,NHLRC1,AIRE,MGRN1,BRCA1,FANCL,MID1,TRIM32,TRIM37,RBX1,CUL1,SKP1,BTRC,FBXW11,SKP2,FBXW7,FBXO2,FBXO4,CUL2,ELOC,ELOB,VHL,CUL3,KEAP1,KLHL9,KLHL13,RHOBTB2,RHOBTB1,CUL4B,CUL4A,DDB1,DDB2,ERCC8,DET1,RNF7,CUL5,SOCS1,SOCS3,CUL7,FBXW8,ANAPC11,ANAPC2,CDC20,FZR1,ANAPC1,CDC27,ANAPC4,ANAPC5,CDC16,ANAPC7,CDC23,ANAPC10,CDC26,ANAPC13",Ubiquitin mediated proteolysis,137

hsa04122,"NFS1,MPST,TST,URM1,MOCS3,CTU1,CTU2,MOCS2",Sulfur relay system,8

hsa04130,"STX1A,STX2,STX3,STX1B,STX4,STX19,STX11,STX7,STX16,STX5,STX17,STX18,VTI1B,VTI1A,GOSR1,GOSR2,BNIP1,STX6,STX10,STX8,BET1L,BET1,USE1,SNAP23,SNAP29,VAMP1,VAMP2,VAMP3,VAMP8,VAMP4,VAMP5,VAMP7,YKT6,SEC22B",SNARE interactions in vesicular transport,34

hsa04136,"RPTOR,MTOR,MLST8,ULK2,ATG13,ATG101,IGBP1,PPP2CA,PPP2CB,ATG9B,ATG9A,ATG2A,ATG2B,WIPI2,WIPI1,BECN1,BECN2,PIK3C3,PIK3R4,ATG12,ATG5,ATG16L1,ATG7,ATG10,ATG3,GABARAP,GABARAPL1,GABARAPL2,ATG4A,ATG4B,ATG4C,ATG4D",Autophagy - other,32

hsa04137,"BCL2L13,EIF2AK3,ATF4,MAPK8,MAPK10,MAPK9,JUN,PINK1,TOMM7,PRKN,UBB,MFN1,MFN2,RHOT1,RHOT2,USP8,USP15,USP30,TAX1BP1,SQSTM1,CALCOCO2,OPTN,NBR1,TBK1,GABARAP,GABARAPL1,GABARAPL2,AMBRA1,ATG5,ATG9B,ATG9A,MITF,TFEB,TFE3,BECN1,BECN2,BCL2L1,PGAM5,CSNK2A1,CSNK2A2,CSNK2A3,CSNK2B,SRC,ULK1,FUNDC1,HIF1A,E2F1,RELA,BNIP3,HRAS,KRAS,NRAS,MRAS,RRAS,RRAS2,TP53,BNIP3L,FOXO3,CITED2,SP1,TBC1D15,TBC1D17,RAB7A,RAB7B,FIS1",Mitophagy - animal,65

hsa04140,"INS,IGF1R,IRS1,IRS2,IRS4,PIK3CA,PIK3CD,PIK3CB,PIK3R1,PIK3R2,PIK3R3,PDPK1,AKT1,AKT2,AKT3,PTEN,HRAS,KRAS,NRAS,MRAS,RRAS,RRAS2,RAF1,MAP2K1,MAP2K2,MAPK1,MAPK3,HIF1A,DDIT4,TSC2,TSC1,BNIP3,RHEB,RPTOR,DEPTOR,MTOR,AKT1S1,MLST8,RPS6KB1,RPS6KB2,IGBP1,PPP2CA,PPP2CB,ULK1,ULK2,ATG13,ATG101,RB1CC1,ATG2A,ATG2B,WIPI2,WIPI1,ATG9B,ATG9A,SUPT20H,RRAGA,RRAGB,RRAGC,RRAGD,STK11,PRKAA1,PRKAA2,PRKACA,PRKACB,PRKACG,PRKCD,MAPK8,MAPK10,MAPK9,BCL2,BCL2L1,BAD,BECN1,BECN2,NRBF2,PIK3R4,PIK3C3,ATG14,AMBRA1,TRAF6,TP53INP2,VMP1,STX17,UVRAG,SH3GLB1,RUBCN,CAMKK2,MAP3K7,ERN1,ITPR1,DAPK1,DAPK3,DAPK2,HMGB1,PRAP1,EIF2AK3,EIF2AK4,EIF2S1,MTMR3,MTMR4,MTMR14,ZFYVE1,ATG12,ATG5,ATG16L1,ATG16L2,ATG7,ATG10,ATG3,CFLAR,RAB33B,PRKCQ,GABARAP,GABARAPL1,GABARAPL2,ATG4A,ATG4B,ATG4C,ATG4D,LAMP1,LAMP2,RAB7A,RAB7B,VAMP8,SNAP29,CTSD,CTSL,CTSB",Autophagy - animal,128

hsa04141,"SEC61A1,SEC61A2,SEC61B,SEC61G,SEC62,SEC63,RPN1,RPN2,DAD1,TUSC3,DDOST,STT3A,STT3B,MOGS,CKAP4,RRBP1,SIL1,HYOU1,HSPA5,DNAJB11,DNAJC1,DNAJC3,DNAJC10,HSP90B1,GANAB,PRKCSH,CANX,PDIA3,CALR,MAN1B1,MAN1A2,MAN1C1,MAN1A1,LMAN2,LMAN1,LMAN1L,PREB,SAR1A,SAR1B,SEC13,SEC31A,SEC31B,SEC23B,SEC23A,SEC24B,SEC24A,SEC24C,SEC24D,UGGT2,UGGT1,EDEM1,EDEM2,EDEM3,P4HB,PDIA4,PDIA6,ERP29,TXNDC5,ERO1A,ERO1B,OS9,ERLEC1,SSR1,SSR2,SSR3,SSR4,BCAP31,TRAM1,DERL1,DERL2,DERL3,UBXN6,NSFL1C,SVIP,VCP,NPLOC4,UFD1,HSPA8,HSPA1A,HSPA2,HSPA1L,HSPA1B,HSPA6,DNAJA1,DNAJA2,DNAJB1,DNAJB2,DNAJB12,DNAJC5,DNAJC5B,DNAJC5G,HSP90AA1,HSP90AB1,HSPH1,HSPA4L,BAG1,BAG2,HSPBP1,CRYAA,CRYAA2,CRYAB,YOD1,PLAA,RAD23B,RAD23A,UBQLN1,UBQLN2,UBQLN3,UBQLN4,NGLY1,ATXN3,ATXN3L,UBE4B,EIF2AK1,EIF2AK2,EIF2AK3,EIF2AK4,EIF2S1,NFE2L2,ATF4,PPP1R15A,DDIT3,BCL2,ATF6,ATF6B,WFS1,MBTPS1,MBTPS2,XBP1,ERN1,TRAF2,MAP3K5,MAP2K7,MAPK8,MAPK10,MAPK9,BAX,BAK1,CAPN1,CAPN2,CASP12,MARCHF6,UBE2J1,UBE2J2,UBE2G1,UBE2G2,SYVN1,RNF5,RNF185,SELENOS,SEL1L,SEL1L2,HERPUD1,AMFR,STUB1,UBE2D4,UBE2D1,UBE2D2,UBE2D3,PRKN,RBX1,CUL1,SKP1,FBXO2,FBXO6",Protein processing in endoplasmic reticulum,165

hsa04142,"TCIRG1,ATP6V0A2,ATP6V0A4,ATP6V0A1,ATP6V0D1,ATP6V0D2,ATP6V1H,ATP6AP1,ATP6V0C,ATP6V0B,CTSA,CTSB,CTSC,CTSD,CTSE,CTSF,CTSG,CTSH,CTSK,CTSL,CTSO,CTSS,CTSV,CTSW,CTSZ,NAPSA,LGMN,TPP1,GLA,GLB1,GAA,GBA,IDUA,NAGA,NAGLU,GALC,GUSB,HEXA,HEXB,MANBA,MAN2B1,NEU1,ARSA,ARSB,ARSG,GALNS,GNS,IDS,SGSH,LIPA,PLA2G15,DNASE2,DNASE2B,ACP2,ACP5,SMPD1,ASAH1,AGA,PSAP,PSAPL1,GM2A,PPT1,PPT2,LAMP1,LAMP2,LAMP3,CD68,CD63,SCARB2,NPC1,NPC2,CTNS,SLC17A5,SLC11A1,SLC11A2,LAPTM4B,LAPTM5,LAPTM4A,ABCA2,ABCB9,CD164,ENTPD4,SORT1,CLN3,CLN5,MFSD8,HGSNAT,SUMF1,GNPTAB,GNPTG,NAGPA,IGF2R,M6PR,CLTA,CLTB,CLTC,CLTCL1,AP1G1,AP1G2,AP1B1,AP1M1,AP1M2,AP1S1,AP1S2,AP1S3,AP3D1,AP3B2,AP3B1,AP3M1,AP3M2,AP3S2,AP3S1,AP4E1,AP4B1,AP4M1,AP4S1,GGA2,GGA3,GGA1,MCOLN1,LITAF,FUCA1,HYAL1",Lysosome,123

hsa04144,"ARF6,PIP5K1C,PIP5K1A,PIP5K1B,PIP5KL1,PLD1,PLD2,DNM1,DNM3,DNM2,CLTA,CLTB,CLTC,CLTCL1,AP2A2,AP2A1,AP2B1,AP2M1,AP2S1,EHD2,TGFBR1,TGFBR2,ZFYVE9,ZFYVE16,PML,SMAD2,SMAD3,TFRC,EGFR,PDGFRA,FGFR2,FGFR3,FGFR4,IGF1R,CBL,CBLB,CBLC,SH3KBP1,SH3GL3,SH3GL2,SH3GL1,SH3GLB1,SH3GLB2,AMPH,BIN1,EPN1,EPN3,EPN2,EPS15,EPS15L1,SPART,UBB,NEDD4,NEDD4L,MDM2,TRAF6,RNF41,ITCH,SMURF1,SMURF2,WWP1,LDLR,LDLRAP1,DAB2,CCR5,CXCR1,CXCR2,CXCR4,GRK7,GRK1,GRK2,GRK3,GRK4,GRK5,GRK6,ARRB1,ARRB2,WAS,WASL,ARPC1B,ARPC1A,ARPC2,ARPC3,ARPC4,ARPC5,ARPC5L,WIPF2,WIPF1,WIPF3,HSPA8,HSPA1A,HSPA2,HSPA1L,HSPA1B,HSPA6,DNAJC6,WASHC4,WASHC5,WASHC2C,WASHC2A,WASHC1,WASHC3,CAPZA1,CAPZA2,CAPZA3,CAPZB,VPS29,VPS26B,VPS26A,VPS35,SNX12,SNX3,USP8,STAMBP,RAB7A,HGS,STAM2,STAM,TSG101,MVB12A,MVB12B,VPS28,VPS37D,VPS37A,VPS37B,VPS37C,SNF8,VPS36,VPS25,CHMP6,CHMP4C,CHMP4B,CHMP4A,CHMP3,RNF103-CHMP3,CHMP2A,CHMP2B,CHMP7,VPS4B,VPS4A,VTA1,CHMP1B,CHMP1A,CHMP5,IST1,PDCD6IP,SPG21,IGF2R,SNX1,SNX2,SNX32,SNX5,SNX6,SRC,HRAS,IL2RA,IL2RB,IL2RG,RHOA,HLA-A,HLA-B,HLA-C,HLA-F,HLA-G,HLA-E,FOLR1,FOLR2,FOLR3,IZUMO1R,CAV1,CAV2,CAV3,EHD3,EHD4,EEA1,RAB5A,RAB5B,RAB5C,RABEP1,RBSN,RAB4A,RUFY1,EHD1,VPS45,SNX4,RAB22A,RAB31,RAB10,RAB8A,RAB35,RAB11A,RAB11B,RAB11FIP2,RAB11FIP1,RAB11FIP5,ZFYVE27,KIF5A,KIF5B,KIF5C,RAB11FIP4,RAB11FIP3,PARD3,PARD6A,PARD6G,PARD6B,PRKCZ,PRKCI,CDC42,SMAP2,SMAP1,GIT1,GIT2,ASAP1,ASAP3,ASAP2,ACAP3,ACAP2,ACAP1,ARAP1,ARAP2,ARAP3,AGAP1,AGAP3,AGAP2,ARFGAP1,ARFGAP3,ARFGAP2,PSD3,PSD4,PSD,PSD2,IQSEC2,IQSEC1,IQSEC3,CYTH3,CYTH4,CYTH2,CYTH1,ARFGEF2,ARFGEF1,GBF1,ARF1,ARF3,ARF5",Endocytosis,244

hsa04145,"VAMP3,STX12,STX7,ACTB,ACTG1,CORO1A,STX18,SEC22B,HLA-A,HLA-B,HLA-C,HLA-F,HLA-G,HLA-E,HLA-DMA,HLA-DMB,HLA-DOA,HLA-DOB,HLA-DPA1,HLA-DPB1,HLA-DQA1,HLA-DQA2,HLA-DQB1,HLA-DRA,HLA-DRB1,HLA-DRB3,HLA-DRB4,HLA-DRB5,RAB5A,RAB5B,RAB5C,EEA1,PIK3C3,TFRC,HGS,ATP6V1A,ATP6V1B1,ATP6V1B2,ATP6V1C2,ATP6V1C1,ATP6V1D,ATP6V1E2,ATP6V1E1,ATP6V1F,ATP6V1G1,ATP6V1G3,ATP6V1G2,ATP6V0E1,ATP6V0E2,TCIRG1,ATP6V0A2,ATP6V0A4,ATP6V0A1,ATP6V0D1,ATP6V0D2,ATP6V1H,ATP6V0C,ATP6V0B,ATP6AP1,RAB7A,RAB7B,RILP,DYNC1H1,DYNC2H1,DYNC1I1,DYNC1I2,DYNC1LI2,DYNC1LI1,TUBA1B,TUBA4A,TUBA3C,TUBA1A,TUBA1C,TUBA8,TUBA3E,TUBA3D,TUBAL3,TUBB6,TUBB,TUBB1,TUBB2A,TUBB3,TUBB4A,TUBB8,TUBB2B,TUBB4B,LAMP1,LAMP2,PIKFYVE,M6PR,NOS1,MPO,CTSL,CTSS,SEC61A1,SEC61A2,SEC61B,SEC61G,TAP1,TAP2,CALR,CANX,FCAR,FCGR1A,FCGR2A,FCGR2B,FCGR2C,FCGR3A,FCGR3B,LOC102723407,C1R,ITGAM,ITGB2,C3,COLEC11,COLEC12,MBL2,SFTPA1,SFTPA2,SFTPD,ITGAV,ITGA2,ITGA5,ITGB1,ITGB3,ITGB5,THBS1,COMP,THBS2,THBS3,THBS4,TLR2,TLR6,TLR4,CD14,PLA2R1,MRC1,MRC2,CLEC4M,CD209,CLEC7A,MSR1,MARCO,OLR1,SCARB1,CD36,CYBA,CYBB,RAC1,NCF1,NCF2,NCF4",Phagosome,152

hsa04146,"PEX16,PEX3,PEX19,ABCD3,PEX1,PEX6,PEX26,PEX7,PEX5,PEX5L,PEX14,PEX13,PEX12,PEX10,PEX2,PXMP2,MPV17,MPV17L2,MPV17L,PXMP4,PEX11A,PEX11B,PEX11G,SLC25A17,HACL1,AMACR,PHYH,ACOX3,ACOX1,ACOX2,HSD17B4,SCP2,BAAT,EHHADH,ACAA1,DECR2,ECH1,ABCD1,ABCD2,ABCD4,SLC27A2,ACSL6,ACSL4,ACSL1,ACSL5,ACSL3,PECR,ECI2,NUDT7,NUDT12,NUDT19,ACOT8,CRAT,CROT,MLYCD,GNPAT,AGPS,FAR2,FAR1,MVK,PMVK,AGXT,DAO,DDO,IDH1,IDH2,PAOX,PIPOX,HMGCL,HMGCLL1,HAO2,HAO1,CAT,PRDX5,SOD1,SOD2,NOS2,PRDX1,EPHX2,GSTK1,XDH,DHRS4,DHRS4L1",Peroxisome,83

hsa04150,"SLC7A5,SLC3A2,SLC38A9,ATP6V1A,ATP6V1B1,ATP6V1B2,ATP6V1C2,ATP6V1C1,ATP6V1D,ATP6V1E2,ATP6V1E1,ATP6V1F,ATP6V1G1,ATP6V1G3,ATP6V1G2,ATP6V1H,LAMTOR1,LAMTOR2,LAMTOR3,LAMTOR4,LAMTOR5,FLCN,FNIP1,FNIP2,RRAGA,RRAGB,RRAGC,RRAGD,SESN2,CASTOR1,CASTOR2,MIOS,SEH1L,WDR24,WDR59,SEC13,DEPDC5,NPRL2,NPRL3,SKP2,RNF152,RPTOR,AKT1S1,MTOR,DEPTOR,MLST8,TELO2,TTI1,CLIP1,GRB10,ULK1,ULK2,EIF4EBP1,EIF4E,EIF4E2,EIF4E1B,RPS6KB1,RPS6KB2,EIF4B,RPS6,STRADA,STRADB,STK11,CAB39,CAB39L,PRKAA1,PRKAA2,TSC1,TSC2,TBC1D7,TBC1D7-LOC100130357,RHEB,DDIT4,WNT1,WNT2,WNT2B,WNT3,WNT3A,WNT4,WNT5A,WNT5B,WNT6,WNT7A,WNT7B,WNT8A,WNT8B,WNT9A,WNT9B,WNT10B,WNT10A,WNT11,WNT16,FZD1,FZD7,FZD2,FZD3,FZD4,FZD5,FZD8,FZD6,FZD10,FZD9,LRP5,LRP6,DVL3,DVL2,DVL1,GSK3B,TNF,TNFRSF1A,IKBKB,INS,IGF1,INSR,IGF1R,GRB2,SOS1,SOS2,HRAS,KRAS,NRAS,BRAF,RAF1,MAP2K1,MAP2K2,MAPK1,MAPK3,RPS6KA3,RPS6KA1,RPS6KA2,RPS6KA6,IRS1,PIK3R1,PIK3R2,PIK3R3,PIK3CA,PIK3CD,PIK3CB,PTEN,PDPK1,AKT1,AKT2,AKT3,CHUK,MAPKAP1,RICTOR,PRR5,RHOA,PRKCA,PRKCB,PRKCG,SGK1,LPIN1",mTOR signaling pathway,153

hsa04151,"EGF,TGFA,EREG,AREG,FGF1,FGF2,FGF3,FGF4,FGF17,FGF6,FGF7,FGF8,FGF9,FGF10,FGF16,FGF5,FGF18,FGF20,FGF22,FGF19,FGF21,FGF23,NGF,BDNF,NTF3,NTF4,INS,IGF1,IGF2,PDGFA,PDGFB,PDGFC,PDGFD,CSF1,KITLG,FLT3LG,VEGFA,VEGFB,PGF,VEGFC,VEGFD,HGF,ANGPT1,ANGPT2,ANGPT4,EFNA1,EFNA2,EFNA3,EFNA4,EFNA5,EGFR,ERBB2,ERBB3,ERBB4,FGFR1,FGFR2,FGFR3,FGFR4,NGFR,NTRK1,NTRK2,INSR,IGF1R,PDGFRA,PDGFRB,CSF1R,KIT,FLT3,FLT1,FLT4,KDR,MET,TEK,EPHA2,GRB2,SOS1,SOS2,HRAS,KRAS,NRAS,RAF1,MAP2K1,MAP2K2,MAPK1,MAPK3,IRS1,TLR2,TLR4,RAC1,LOC102723407,SYK,CD19,PIK3AP1,GH1,GH2,CSH1,CSH2,PRL,OSM,IL2,IL3,IL6,IL4,IL7,IFNA1,IFNA2,IFNA4,IFNA5,IFNA6,IFNA7,IFNA8,IFNA10,IFNA13,IFNA14,IFNA16,IFNA17,IFNA21,IFNB1,EPO,CSF3,GHR,PRLR,OSMR,IL2RA,IL2RB,IL2RG,IL3RA,IL6R,IL4R,IL7R,IFNAR1,IFNAR2,EPOR,CSF3R,JAK1,JAK2,JAK3,COL1A1,COL1A2,COL2A1,COL4A2,COL4A4,COL4A6,COL4A1,COL4A5,COL4A3,COL6A1,COL6A2,COL6A3,COL6A6,COL6A5,COL9A1,COL9A2,COL9A3,LAMA1,LAMA2,LAMA3,LAMA5,LAMA4,LAMB1,LAMB2,LAMB3,LAMB4,LAMC1,LAMC2,LAMC3,CHAD,RELN,THBS1,COMP,THBS2,THBS3,THBS4,FN1,SPP1,VTN,TNC,TNN,TNR,TNXB,VWF,IBSP,ITGA1,ITGA2,ITGA2B,ITGA3,ITGA4,ITGA5,ITGA6,ITGA7,ITGA8,ITGA9,ITGA10,ITGA11,ITGAV,ITGB1,ITGB3,ITGB4,ITGB5,ITGB6,ITGB7,ITGB8,PTK2,PIK3CA,PIK3CD,PIK3CB,PIK3R1,PIK3R2,PIK3R3,F2R,CHRM1,CHRM2,LPAR1,LPAR2,LPAR3,LPAR4,LPAR5,LPAR6,GNB1,GNB2,GNB3,GNB4,GNB5,GNG2,GNG3,GNG4,GNG5,GNG7,GNG8,GNG10,GNG11,GNG12,GNG13,GNGT1,GNGT2,PIK3CG,PIK3R5,PIK3R6,PDPK1,STK11,PRKAA1,PRKAA2,DDIT4,TSC1,TSC2,RHEB,MLST8,MTOR,RPTOR,EIF4EBP1,EIF4E,EIF4E2,EIF4E1B,RPS6KB1,RPS6KB2,EIF4B,RPS6,PRKCA,PKN1,PKN3,PKN2,SGK1,SGK2,SGK3,C8orf44-SGK3,AKT1,AKT2,AKT3,MAGI1,MAGI2,PTEN,THEM4,PPP2CA,PPP2CB,PPP2R1B,PPP2R1A,PPP2R2A,PPP2R2B,PPP2R2C,PPP2R2D,PPP2R3B,PPP2R3C,PPP2R3A,PPP2R5B,PPP2R5C,PPP2R5D,PPP2R5E,PPP2R5A,HSP90AA1,HSP90AB1,HSP90B1,CDC37,CRTC2,PHLPP1,PHLPP2,TCL1A,TCL1B,MTCP1,NOS3,BRCA1,GSK3B,GYS2,GYS1,PCK1,PCK2,G6PC,G6PC2,G6PC3,MYC,CCND1,CDKN1A,CDKN1B,CDK2,CDK4,CDK6,CCND2,CCND3,CCNE1,CCNE2,FOXO3,RBL2,FASLG,BCL2L11,YWHAZ,YWHAB,YWHAQ,YWHAE,YWHAH,YWHAG,BAD,BCL2L1,BCL2,CASP9,CREB1,ATF2,ATF4,CREB3,CREB3L1,CREB3L2,CREB3L3,CREB3L4,CREB5,ATF6B,MCL1,RXRA,NR4A1,IKBKG,CHUK,IKBKB,RELA,NFKB1,MYB,MDM2,TP53",PI3K-Akt signaling pathway,354

hsa04152,"MAP3K7,STK11,CAB39,CAB39L,STRADA,STRADB,LEP,LEPR,CAMKK2,ADRA1A,ADIPOQ,ADIPOR1,ADIPOR2,PRKAA1,PRKAA2,PRKAB1,PRKAB2,PRKAG1,PRKAG3,PRKAG2,PFKFB1,PFKFB2,PFKFB3,PFKFB4,FBP1,FBP2,PFKM,PFKP,PFKL,HNF4A,G6PC,G6PC2,G6PC3,PCK1,PCK2,CRTC2,CREB1,CREB3,CREB3L1,CREB3L2,CREB3L3,CREB3L4,CREB5,PPARGC1A,ELAVL1,CCND1,CCNA2,CCNA1,EEF2K,EEF2,FOXO1,FOXO3,SIRT1,SLC2A4,GYS2,GYS1,LIPE,HMGCR,SREBF1,FASN,ACACA,SCD,SCD5,ACACB,MLYCD,CPT1A,CPT1B,CPT1C,PPP2CA,PPP2CB,PPP2R1B,PPP2R1A,PPP2R2A,PPP2R2B,PPP2R2C,PPP2R2D,PPP2R3B,PPP2R3C,PPP2R3A,PPP2R5B,PPP2R5C,PPP2R5D,PPP2R5E,PPP2R5A,TBC1D1,RAB2A,RAB8A,RAB10,RAB11B,RAB14,CFTR,CD36,IGF1,IGF1R,INS,INSR,IRS1,IRS2,IRS4,PIK3CA,PIK3CD,PIK3CB,PIK3R1,PIK3R2,PIK3R3,PDPK1,AKT1,AKT2,AKT3,TSC2,TSC1,RHEB,MTOR,RPTOR,AKT1S1,RPS6KB1,RPS6KB2,EIF4EBP1,PPARG,ULK1",AMPK signaling pathway,120

hsa04210,"TNFSF10,TNFRSF10A,TNFRSF10B,FASLG,FAS,FADD,TNF,TNFRSF1A,TRADD,CFLAR,CASP8,CASP10,CASP6,CASP3,CASP7,BID,BAX,BAK1,DIABLO,SEPTIN4,HTRA2,CYCS,APAF1,CASP9,PRF1,GZMB,TUBA1B,TUBA4A,TUBA3C,TUBA1A,TUBA1C,TUBA8,TUBA3E,TUBA3D,TUBAL3,MCL1,ACTB,ACTG1,SPTA1,SPTAN1,LMNA,LMNB1,LMNB2,PARP2,PARP1,PARP3,PARP4,DFFA,DFFB,ENDOG,AIFM1,ERN1,TRAF2,ITPR1,ITPR2,ITPR3,CAPN1,CAPN2,CASP12,EIF2AK3,EIF2S1,ATF4,DDIT3,CTSB,CTSC,CTSD,CTSF,CTSH,CTSK,CTSL,CTSO,CTSS,CTSV,CTSW,CTSZ,BIRC2,BIRC3,XIAP,BIRC5,BCL2L11,BCL2L1,BCL2,DAXX,RIPK1,DAB2IP,MAP3K5,MAPK8,MAPK10,MAPK9,BAD,JUN,FOS,TP53,HRK,MAP3K14,CHUK,IKBKB,IKBKG,NFKBIA,NFKB1,RELA,PTPN13,GADD45A,GADD45B,GADD45G,TRAF1,BCL2A1,ATM,PIDD1,TP53AIP1,BBC3,PMAIP1,CASP2,NGF,NTRK1,IL3,IL3RA,CSF2RB,PIK3CA,PIK3CD,PIK3CB,PIK3R1,PIK3R2,PIK3R3,PDPK1,AKT1,AKT2,AKT3,HRAS,KRAS,NRAS,RAF1,MAP2K1,MAP2K2,MAPK1,MAPK3",Apoptosis,136

hsa04211,"INS,INSR,IGF1,IGF1R,IRS1,IRS2,IRS4,HRAS,KRAS,NRAS,PIK3CA,PIK3CD,PIK3CB,PIK3R1,PIK3R2,PIK3R3,AKT1,AKT2,AKT3,ADCY1,ADCY2,ADCY3,ADCY4,ADCY5,ADCY6,ADCY7,ADCY8,ADCY9,PRKACA,PRKACB,PRKACG,CREB1,ATF2,CREB3,CREB3L1,CREB3L2,CREB3L3,CREB3L4,ATF4,CREB5,ATF6B,EHMT2,EHMT1,FOXO1,FOXO3,SOD2,CAT,ATG5,KL,TSC1,TSC2,RHEB,MTOR,RPTOR,AKT1S1,ULK1,ATG13,RB1CC1,ATG101,RPS6KB1,RPS6KB2,EIF4EBP1,EIF4E,EIF4E2,EIF4E1B,ADIPOQ,ADIPOR1,ADIPOR2,APPL1,CAMKK2,PRKAA1,PRKAA2,PRKAB1,PRKAB2,PRKAG1,PRKAG3,PRKAG2,SIRT1,BAX,TP53,NFKB1,RELA,PPARG,PPARGC1A,STK11,SESN1,SESN3,SESN2,CAMK4",Longevity regulating pathway,89

hsa04213,"INS,INSR,IGF1,IGF1R,IRS1,IRS2,IRS4,HRAS,KRAS,NRAS,PIK3CA,PIK3CD,PIK3CB,PIK3R1,PIK3R2,PIK3R3,AKT1,AKT2,AKT3,ADCY1,ADCY2,ADCY3,ADCY4,ADCY5,ADCY6,ADCY7,ADCY8,ADCY9,PRKACA,PRKACB,PRKACG,FOXO1,FOXO3,SOD2,CAT,ATG5,PRKAA1,PRKAA2,PRKAB1,PRKAB2,PRKAG1,PRKAG3,PRKAG2,MTOR,RPTOR,AKT1S1,RPS6KB1,RPS6KB2,SIRT1,SOD1,EIF4EBP2,HSPA8,HSPA1A,HSPA2,HSPA1L,HSPA1B,HSPA6,CRYAB,HDAC1,HDAC2,FOXA2,CLPB",Longevity regulating pathway - multiple species,62

hsa04215,"TNFRSF1A,FADD,CASP8,CASP7,CASP3,BID,BCL2L11,PMAIP1,BBC3,BCL2,BCL2L1,BAX,BAK1,CYCS,APAF1,CASP9,DIABLO,HTRA2,SEPTIN4,BIRC2,BIRC3,XIAP,BIRC5,MAPK8,MAPK10,MAPK9,BOK,BIRC7,BIRC8,NGFR,BECN1,BECN2,BIRC6",Apoptosis - multiple species,33

hsa04216,"SLC3A2,SLC7A11,GCLC,GCLM,GSS,GPX4,ALOX15,LPCAT3,ACSL6,ACSL4,ACSL1,ACSL5,ACSL3,TP53,SAT2,SAT1,TF,TFRC,STEAP3,SLC11A2,SLC39A8,SLC39A14,PCBP2,SLC40A1,CP,PCBP1,FTH1,FTL,MAP1LC3C,MAP1LC3B,MAP1LC3A,ATG5,ATG7,NCOA4,PRNP,HMOX1,VDAC2,VDAC3,CYBB,FTMT",Ferroptosis,40

hsa04217,"TNF,TNFRSF1A,TRADD,TRAF2,TRAF5,RIPK1,BIRC2,BIRC3,XIAP,RBCK1,RNF31,SHARPIN,SPATA2L,SPATA2,CYLD,FADD,CASP8,CFLAR,RIPK3,CYBB,CAMK2A,CAMK2D,CAMK2B,CAMK2G,SLC25A4,SLC25A5,SLC25A6,SLC25A31,PPID,VDAC1,VDAC2,VDAC3,GLUD2,GLUD1,GLUL,PYGL,PYGM,PYGB,MAPK8,MAPK10,MAPK9,FTH1,FTL,PLA2G4E,PLA2G4A,JMJD7-PLA2G4B,PLA2G4B,PLA2G4C,PLA2G4D,PLA2G4F,ALOX15,CAPN1,CAPN2,SMPD1,MLKL,PGAM5,DNM1L,NLRP3,PYCARD,CASP1,IL1B,CHMP2A,CHMP2B,CHMP3,RNF103-CHMP3,CHMP4C,CHMP4B,CHMP4A,CHMP6,VPS4B,VPS4A,CHMP1B,CHMP1A,CHMP5,CHMP7,TRPM7,IL1A,IL33,HMGB1,TNFSF10,TNFRSF10A,TNFRSF10B,FASLG,FAS,FAF1,IFNA1,IFNA2,IFNA4,IFNA5,IFNA6,IFNA7,IFNA8,IFNA10,IFNA13,IFNA14,IFNA16,IFNA17,IFNA21,IFNB1,IFNG,IFNAR1,IFNAR2,IFNGR1,IFNGR2,JAK1,JAK2,JAK3,TYK2,STAT1,STAT2,STAT3,STAT4,STAT5A,STAT5B,STAT6,IRF9,EIF2AK2,TLR4,TICAM2,TICAM1,TLR3,ZBP1,USP21,SQSTM1,HSP90AA1,HSP90AB1,TNFAIP3,PARP2,PARP1,PARP3,PARP4,BID,BAX,AIFM1,H2AX,H2AC20,H2AC12,H2AC1,H2AW,H2AB3,H2AC8,H2AC4,MACROH2A2,MACROH2A1,H2AC19,H2AJ,H2AB1,H2AC17,H2AC18,H2AC11,H2AC21,H2AZ2,H2AC7,H2AZ1,H2AC15,H2AC6,H2AC13,H2AC14,H2AC16,H2AB2,PPIA,BCL2",Necroptosis,162

hsa04218,"TGFB1,TGFB2,TGFB3,TGFBR1,TGFBR2,SMAD2,SMAD3,CDKN2B,CDK4,CDK6,CCND1,CCND2,CCND3,RB1,RBL1,RBL2,E2F1,E2F2,E2F3,E2F4,E2F5,PIK3CA,PIK3CD,PIK3CB,PIK3R1,PIK3R2,PIK3R3,FOXO1,FOXO3,CDKN1A,CDK2,CCNE1,CCNE2,HLA-A,HLA-B,HLA-C,HLA-F,HLA-G,HLA-E,KIR2DL2,KIR2DL1,KIR2DL3,KIR2DL4,KIR2DL5A,KRAS,NRAS,RRAS,RRAS2,MRAS,HRAS,AKT1,AKT2,AKT3,TSC1,TSC2,RHEB,MTOR,PTEN,SIRT1,CCNA2,CCNA1,MYBL2,LIN9,LIN37,LIN52,LIN54,RBBP4,FOXM1,MYC,CDKN2A,MDM2,TP53,RASSF5,BTRC,FBXW11,HIPK3,HIPK1,HIPK2,HIPK4,PPP1CA,PPP1CB,PPP1CC,RAF1,MAP2K1,MAP2K2,MAPK1,MAPK3,ETS1,MAP2K3,MAP2K6,MAPK11,MAPK12,MAPK13,MAPK14,GADD45A,GADD45B,GADD45G,CDK1,CCNB1,CCNB2,CCNB3,MRE11,RAD50,NBN,ATM,CHEK2,RAD9A,RAD9B,RAD1,HUS1,ATR,CHEK1,CDC25A,SQSTM1,GATA4,TRAF3IP2,NFKB1,RELA,IL1A,IL6,CXCL8,IGFBP3,SERPINE1,EIF4EBP1,MAPKAPK2,ZFP36L1,ZFP36L2,CACNA1D,TRPV4,TRPM7,CAPN1,CAPN2,CALML3,CALM2,CALM3,CALM1,CALML6,CALML5,CALML4,PPP3CA,PPP3CB,PPP3CC,PPP3R1,PPP3R2,NFATC1,NFATC2,NFATC3,NFATC4,ITPR1,ITPR2,ITPR3,SLC25A4,SLC25A5,SLC25A6,SLC25A31,PPID,VDAC1,VDAC2,VDAC3,MCU",Cellular senescence,160

hsa04260,"CACNA1C,CACNA1D,CACNA1F,CACNA1S,CACNB1,CACNB2,CACNB3,CACNB4,CACNA2D1,CACNA2D2,CACNA2D3,CACNA2D4,CACNG1,CACNG2,CACNG3,CACNG4,CACNG5,CACNG6,CACNG7,CACNG8,RYR2,ASPH,CASQ2,TRDN,HRC,ATP2A1,ATP2A3,ATP2A2,TNNC1,TNNI3,TNNT2,TPM1,TPM2,TPM3,TPM4,ACTC1,MYH7,MYH6,MYL2,MYL3,MYL4,UQCRFS1,CYTB,CYC1,UQCRC1,UQCRC2,UQCRH,UQCRHL,UQCRB,UQCRQ,UQCR10,UQCR11,COX3,COX1,COX2,COX4I2,COX4I1,COX5A,COX5B,COX6A1,COX6A2,COX6B1,COX6B2,COX6C,COX7A1,COX7A2,COX7A2L,COX7B,COX7B2,COX7C,COX8C,COX8A,SLC8A1,SLC8A2,SLC8A3,ATP1A1,ATP1A2,ATP1A3,ATP1A4,ATP1B4,ATP1B1,ATP1B2,ATP1B3,FXYD2,SLC9A1,SLC9A6",Cardiac muscle contraction,86

hsa04261,"ADRB1,ADRB2,GNAS,ADCY1,ADCY2,ADCY3,ADCY4,ADCY5,ADCY6,ADCY7,ADCY8,ADCY9,PRKACA,PRKACB,PRKACG,ATP1A1,ATP1A2,ATP1A3,ATP1A4,ATP1B4,ATP1B1,ATP1B2,ATP1B3,FXYD2,SLC9A1,KCNQ1,KCNE1,ATP2B1,ATP2B3,ATP2B4,ATP2B2,SLC8A1,SLC8A2,SLC8A3,SCN5A,SCN7A,SCN1B,SCN4B,CACNA1C,CACNA1D,CACNA1F,CACNA1S,CACNB1,CACNB2,CACNB3,CACNB4,CACNA2D1,CACNA2D2,CACNA2D3,CACNA2D4,CACNG1,CACNG2,CACNG3,CACNG4,CACNG5,CACNG6,CACNG7,CACNG8,CALML3,CALM2,CALM3,CALM1,CALML6,CALML5,CALML4,CAMK2A,CAMK2D,CAMK2B,CAMK2G,RYR2,PPP2CA,PPP2CB,PPP2R1B,PPP2R1A,PPP2R2A,PPP2R2B,PPP2R2C,PPP2R2D,PPP2R3B,PPP2R3C,PPP2R3A,PPP2R5B,PPP2R5C,PPP2R5D,PPP2R5E,PPP2R5A,PPP1CA,PPP1CB,PPP1CC,TNNC1,TNNI3,TNNT2,TPM1,TPM2,TPM3,TPM4,ACTC1,MYH7,MYH6,MYL2,MYL3,MYL4,PLN,ATP2A1,ATP2A3,ATP2A2,AGT,AGTR1,AGTR2,ADRA1A,ADRA1B,ADRA1D,GNAQ,PLCB1,PLCB2,PLCB3,PLCB4,PRKCA,PPP1R1A,MAPK1,MAPK3,RPS6KA5,CREB1,ATF2,ATF4,CREB3,CREB3L1,CREB3L2,CREB3L3,CREB3L4,CREB5,ATF6B,CREM,BCL2,RAPGEF3,RAPGEF4,MAPK11,MAPK12,MAPK13,MAPK14,GNAI1,GNAI3,GNAI2,PIK3CG,PIK3R5,PIK3R6,AKT1,AKT2,AKT3",Adrenergic signaling in cardiomyocytes,149

hsa04270,"ADRA1A,ADRA1B,ADRA1D,AGT,AGTR1,EDN1,EDN2,EDN3,EDNRA,AVP,AVPR1A,AVPR1B,GNAQ,GNA11,PLA2G10,PLA2G2D,PLA2G2E,PLA2G3,PLA2G2F,PLA2G12A,PLA2G12B,PLA2G1B,PLA2G5,PLA2G2A,PLA2G2C,PLA2G4E,PLA2G4A,JMJD7-PLA2G4B,PLA2G4B,PLA2G4C,PLA2G4D,PLA2G4F,PLA2G6,CYP4A11,CYP4A22,KCNMA1,KCNU1,KCNMB1,KCNMB2,KCNMB3,KCNMB4,CACNA1C,CACNA1D,CACNA1F,CACNA1S,CALML3,CALM2,CALM3,CALM1,CALML6,CALML5,CALML4,MYLK,MYLK2,MYLK3,MYLK4,MYL6B,MYL6,MYL9,PLCB1,PLCB2,PLCB3,PLCB4,ITPR1,ITPR2,ITPR3,PRKCA,PRKCB,PRKCG,PRKCD,PRKCE,PRKCH,PRKCQ,ARAF,BRAF,RAF1,MAP2K1,MAP2K2,MAPK1,MAPK3,CALD1,ACTA2,ACTG2,PPP1R14A,PPP1CA,PPP1CB,PPP1CC,PPP1R12A,PPP1R12B,PPP1R12C,GNA12,GNA13,ARHGEF12,ARHGEF1,ARHGEF11,RHOA,ROCK1,ROCK2,ADORA2A,ADORA2B,PTGIR,CALCA,CALCB,ADM,CALCRL,RAMP1,RAMP2,RAMP3,GNAS,ADCY1,ADCY2,ADCY3,ADCY4,ADCY5,ADCY6,ADCY7,ADCY8,ADCY9,PRKACA,PRKACB,PRKACG,NPPA,NPPB,NPR1,NPPC,NPR2,PRKG1,MRVI1,GUCY1A2,GUCY1A1,GUCY1B1,MYH11",Vascular smooth muscle contraction,132

hsa04310,"PORCN,WNT1,WNT2,WNT2B,WNT3,WNT3A,WNT4,WNT5A,WNT5B,WNT6,WNT7A,WNT7B,WNT8A,WNT8B,WNT9A,WNT9B,WNT10B,WNT10A,WNT11,WNT16,CER1,NOTUM,WIF1,SERPINF1,SOST,DKK1,DKK2,DKK4,SFRP1,SFRP2,SFRP4,SFRP5,RSPO1,RSPO2,RSPO3,RSPO4,LGR4,LGR5,LGR6,RNF43,ZNRF3,FZD1,FZD7,FZD2,FZD3,FZD4,FZD5,FZD8,FZD6,FZD10,FZD9,LRP5,LRP6,BAMBI,CSNK1E,TPTEP2-CSNK1E,DVL3,DVL2,DVL1,FRAT1,FRAT2,CSNK2A1,CSNK2A2,CSNK2A3,CSNK2B,NKD1,NKD2,CXXC4,SENP2,GSK3B,CTNNB1,AXIN1,AXIN2,APC,APC2,CSNK1A1L,CSNK1A1,TCF7,TCF7L1,TCF7L2,LEF1,CTNNBIP1,CBY1,CHD8,SOX17,CTBP1,CTBP2,CTNND2,CREBBP,EP300,RUVBL1,SMAD4,MAP3K7,NLK,MYC,JUN,FOSL1,CCND1,CCND2,CCND3,CCN4,PPARD,MMP7,PSEN1,PRKACA,PRKACB,PRKACG,TP53,SIAH1,CACYBP,SKP1,TBL1X,TBL1Y,TBL1XR1,BTRC,FBXW11,CUL1,RBX1,GPC4,ROR1,ROR2,RYK,VANGL2,VANGL1,PRICKLE1,PRICKLE2,PRICKLE4,PRICKLE3,INVS,DAAM1,DAAM2,RHOA,ROCK2,RAC1,RAC2,RAC3,MAPK8,MAPK10,MAPK9,PLCB1,PLCB2,PLCB3,PLCB4,CAMK2A,CAMK2D,CAMK2B,CAMK2G,PPP3CA,PPP3CB,PPP3CC,PPP3R1,PPP3R2,PRKCA,PRKCB,PRKCG,NFATC1,NFATC2,NFATC3,NFATC4,SMAD3",Wnt signaling pathway,160

hsa04330,"DLL3,DLL1,DLL4,JAG1,JAG2,MFNG,LFNG,RFNG,NOTCH1,NOTCH2,NOTCH3,NOTCH4,RBPJL,RBPJ,HES1,HES5,PTCRA,DVL3,DVL2,DVL1,NUMB,NUMBL,DTX2,DTX3L,DTX1,DTX3,DTX4,ADAM17,PSEN1,PSEN2,PSENEN,NCSTN,APH1A,APH1B,MAML3,MAML2,MAML1,CREBBP,EP300,KAT2B,KAT2A,SNW1,CTBP1,CTBP2,NCOR2,CIR1,HDAC1,HDAC2",Notch signaling pathway,48

hsa04340,"PTCH1,PTCH2,SMO,GPR161,PRKACA,PRKACB,PRKACG,CSNK1A1L,CSNK1A1,CSNK1G2,CSNK1G3,CSNK1G1,CSNK1D,CSNK1E,TPTEP2-CSNK1E,GSK3B,GLI1,GLI2,GLI3,SUFU,KIF7,HHIP,CCND1,CCND2,BCL2,CUL1,BTRC,FBXW11,SHH,IHH,DHH,BOC,CDON,GAS1,LRP2,SMURF1,SMURF2,GRK2,GRK3,EVC,EVC2,ARRB1,ARRB2,KIF3A,CUL3,SPOP,SPOPL",Hedgehog signaling pathway,47

hsa04350,"CHRD,NOG,NBL1,MICOS10-NBL1,GREM1,GREM2,THBS1,DCN,FMOD,LEFTY1,LEFTY2,FST,BMP2,BMP4,BMP6,INHBB,BMP5,BMP7,BMP8B,BMP8A,GDF5,GDF6,GDF7,AMH,THSD4,FBN1,LTBP1,TGFB1,TGFB2,TGFB3,INHBA,INHBC,INHBE,NODAL,NEO1,HJV,BMPR1A,BMPR1B,ACVR1,BMPR2,ACVR2A,RGMA,RGMB,AMHR2,TGFBR1,TGFBR2,ACVR1B,ACVR2B,ACVR1C,BAMBI,SMAD1,SMAD5,SMAD9,SMAD2,SMAD3,SMAD4,SMAD6,SMAD7,SMURF1,SMURF2,ZFYVE9,ZFYVE16,HAMP,ID1,ID2,ID3,ID4,RBL1,E2F4,E2F5,TFDP1,CREBBP,EP300,SP1,TGIF1,TGIF2,MYC,CDKN2B,PITX2,RBX1,CUL1,SKP1,MAPK1,MAPK3,IFNG,TNF,RHOA,ROCK1,PPP2R1B,PPP2R1A,PPP2CA,PPP2CB,RPS6KB1,RPS6KB2",TGF-beta signaling pathway,94

hsa04360,"NTNG1,NTNG2,LRRC4C,LRRC4,NTN1,NTN3,NTN4,DCC,PLCG1,PLCG2,TRPC1,TRPC3,TRPC4,TRPC5,TRPC6,PPP3CA,PPP3CB,PPP3CC,PPP3R1,PPP3R2,NFATC2,NFATC3,NFATC4,PTK2,FYN,SRC,RAC1,RAC2,RAC3,CDC42,ABLIM1,ABLIM3,ABLIM2,NCK1,PAK1,PAK2,PAK3,PAK4,PAK5,PAK6,BUB1B-PAK6,RHOA,ROCK1,ROCK2,UNC5A,UNC5B,UNC5C,UNC5D,PTPN11,RGMA,NEO1,MYL5,MYL9,MYL12B,MYL12A,ARHGEF12,EFNA1,EFNA2,EFNA3,EFNA4,EFNA5,EFNB1,EFNB2,EFNB3,EPHA1,EPHA2,EPHA3,EPHA4,EPHA5,EPHA6,EPHA7,EPHA8,EPHB1,EPHB2,EPHB3,EPHB4,EPHB6,ABL1,ENAH,RASA1,NGEF,HRAS,KRAS,NRAS,MAPK1,MAPK3,NCK2,RGS3,CXCL12,CXCR4,GNAI1,GNAI3,GNAI2,SLIT1,SLIT2,SLIT3,ROBO1,ROBO2,ROBO3,SRGAP2,SRGAP1,SRGAP3,SEMA3A,SEMA3B,SEMA3C,SEMA3D,SEMA3E,SEMA3F,SEMA3G,PLXNA2,PLXNA3,PLXNA1,PLXNA4,NRP1,L1CAM,LIMK1,LIMK2,CFL1,CFL2,RHOD,RND1,FES,DPYSL5,DPYSL2,CDK5,GSK3B,SEMA4F,SEMA4D,SEMA4B,SEMA4C,SEMA4G,SEMA4A,SEMA5A,SEMA5B,SEMA6A,SEMA6B,SEMA6C,SEMA6D,PLXNB1,PLXNB2,PLXNB3,MET,RAF1,RRAS,SEMA7A,PLXNC1,ITGB1,SHH,PTCH1,SMO,PRKCA,ILK,BOC,WNT5A,WNT5B,FZD3,RYK,CAMK2A,CAMK2D,CAMK2B,CAMK2G,WNT4,PIK3CA,PIK3CD,PIK3CB,PIK3R1,PIK3R2,PIK3R3,PDK1,PARD3,PRKCZ,PARD6A,PARD6G,PARD6B,SSH1,SSH3,SSH2,BMP7,GDF7,BMPR2,BMPR1B",Axon guidance,181

hsa04370,"VEGFA,KDR,SH2D2A,PLCG1,PLCG2,PRKCA,PRKCB,PRKCG,SPHK1,SPHK2,HRAS,KRAS,NRAS,RAF1,MAP2K1,MAP2K2,MAPK1,MAPK3,PLA2G4E,PLA2G4A,JMJD7-PLA2G4B,PLA2G4B,PLA2G4C,PLA2G4D,PLA2G4F,PPP3CA,PPP3CB,PPP3CC,PPP3R1,PPP3R2,NFATC2,PTGS2,PTK2,SHC2,PXN,CDC42,MAPK11,MAPK12,MAPK13,MAPK14,MAPKAPK2,MAPKAPK3,HSPB1,SRC,PIK3CA,PIK3CD,PIK3CB,PIK3R1,PIK3R2,PIK3R3,RAC1,RAC2,RAC3,AKT1,AKT2,AKT3,NOS3,CASP9,BAD",VEGF signaling pathway,59

hsa04371,"ADCY1,ADCY2,ADCY3,ADCY4,ADCY5,ADCY6,ADCY7,ADCY8,ADCY9,ADCY10,PRKACA,PRKACB,PRKACG,LIPE,APLN,APLNR,GNAI1,GNAI3,GNAI2,GNB1,GNB2,GNB3,GNB4,GNB5,GNG2,GNG3,GNG4,GNG5,GNG7,GNG8,GNG10,GNG11,GNG12,GNG13,GNGT1,GNGT2,GNAQ,PLCB1,PLCB2,PLCB3,PLCB4,ITPR1,ITPR2,ITPR3,RYR1,RYR2,RYR3,CALML3,CALM2,CALM3,CALM1,CALML6,CALML5,CALML4,NOS1,NOS2,NOS3,PRKCE,SLC9A1,SLC8A1,SLC8A2,SLC8A3,MYLK,MYLK2,MYLK3,MYLK4,MYL2,MYL3,MYL4,HRAS,KRAS,NRAS,RRAS,RRAS2,MRAS,RAF1,MAP2K1,MAP2K2,MAPK1,MAPK3,RPS6KB1,RPS6KB2,RPS6,PIK3CG,PIK3R5,PIK3R6,AKT1,AKT2,AKT3,MTOR,EGR1,SPP1,JAG1,NOTCH3,CCND1,PDE3B,PIK3C3,PIK3R4,BECN1,BECN2,GABARAP,GABARAPL1,GABARAPL2,PRKAA1,PRKAA2,PRKAB1,PRKAB2,PRKAG1,PRKAG3,PRKAG2,PLIN1,PPARGC1A,UCP1,NRF1,TFAM,KLF2,SPHK1,SPHK2,GNA13,CAMK4,HDAC4,HDAC5,MEF2A,BORCS8-MEF2B,MEF2B,MEF2C,MEF2D,TGFBR1,SMAD2,SMAD3,SMAD4,ACTA2,CDH1,CCN2,AGTR1,SERPINE1,PLAT",Apelin signaling pathway,137

hsa04380,"CSF1,CSF1R,GRB2,MAPK1,MAPK3,PIK3CA,PIK3CD,PIK3CB,PIK3R1,PIK3R2,PIK3R3,AKT1,AKT2,AKT3,IFNG,IFNGR1,IFNGR2,STAT1,IL1A,IL1B,IL1R1,TNF,TNFRSF1A,TGFB1,TGFB2,TGFBR1,TGFBR2,TNFSF11,TNFRSF11A,TNFRSF11B,TRAF2,TRAF6,LCK,FYN,MAP3K14,CHUK,RELB,NFKB2,MAP3K7,TAB1,TAB2,IKBKG,IKBKB,NFKBIA,RELA,NFKB1,NFATC2,NFATC1,IFNB1,MAP2K1,MAP2K6,MAPK11,MAPK12,MAPK13,MAPK14,MAP2K7,MAPK8,MAPK10,MAPK9,FOS,FOSB,FOSL2,FOSL1,JUN,JUND,JUNB,RAC1,NOX1,CYBA,NCF2,NCF1,NCF4,BTK,TEC,OSCAR,LILRB2,LILRB1,LILRB5,LILRB4,LILRA1,LILRB3,LILRA3,LILRA2,LILRA4,LILRA6,LILRA5,LOC102725035,FCGR1A,FCGR2A,FCGR2B,FCGR2C,FCGR3A,FCGR3B,TREM2,SIRPA,SIRPG,SIRPB1,TYROBP,SYK,BLNK,LCP2,PLCG2,PPP3CA,PPP3CB,PPP3CC,PPP3R1,PPP3R2,CAMK4,CREB1,SPI1,MITF,CTSK,ACP5,CALCR,ITGB3,PPARG,IFNAR1,IFNAR2,JAK1,TYK2,STAT2,IRF9,SOCS1,SOCS3,GAB2,FHL2,CYLD,SQSTM1",Osteoclast differentiation,128

hsa04390,"CRB2,CRB1,PARD3,PARD6A,PARD6G,PARD6B,PRKCZ,PRKCI,PATJ,MPP5,AMOT,YAP1,WWTR1,CDH1,LIMD1,AJUBA,WTIP,NF2,WWC1,FRMD1,FRMD6,SAV1,STK3,RASSF6,RASSF1,PPP2CA,PPP2CB,PPP2R1B,PPP2R1A,PPP2R2A,PPP2R2B,PPP2R2C,PPP2R2D,LATS2,LATS1,MOB1A,MOB1B,PPP1CA,PPP1CB,PPP1CC,TP53BP2,LLGL2,LLGL1,SCRIB,DLG1,DLG4,DLG2,DLG3,CSNK1D,CSNK1E,TPTEP2-CSNK1E,BTRC,FBXW11,TP73,BBC3,TEAD1,TEAD4,TEAD3,TEAD2,CCN2,GLI2,AREG,BIRC5,AFP,ITGB2,FGF1,TGFB1,TGFB2,TGFB3,TGFBR1,TGFBR2,SMAD7,SMAD2,SMAD3,SMAD4,SERPINE1,BMP2,BMP4,BMP5,BMP6,BMP7,BMP8B,BMP8A,GDF5,GDF6,GDF7,AMH,BMPR1A,BMPR1B,BMPR2,SMAD1,ID1,ID2,WNT1,WNT2,WNT2B,WNT3,WNT3A,WNT4,WNT5A,WNT5B,WNT6,WNT7A,WNT7B,WNT8A,WNT8B,WNT9A,WNT9B,WNT10B,WNT10A,WNT11,WNT16,FZD1,FZD7,FZD2,FZD3,FZD4,FZD5,FZD8,FZD6,FZD10,FZD9,DVL3,DVL2,DVL1,YWHAZ,YWHAB,YWHAQ,YWHAE,YWHAH,YWHAG,GSK3B,CTNNB1,APC,APC2,AXIN1,AXIN2,TCF7,TCF7L1,TCF7L2,LEF1,MYC,CCND1,CCND2,CCND3,SOX2,SNAI2,ACTB,ACTG1,CTNNA3,CTNNA1,CTNNA2,BIRC2,NKD1",Hippo signaling pathway,154

hsa04392,"DCHS1,DCHS2,FAT4,CSNK1E,TPTEP2-CSNK1E,NF2,WWC1,FRMD1,FRMD6,RASSF1,RASSF6,STK3,SAV1,LATS2,LATS1,MOB1A,MOB1B,LIMD1,AJUBA,WTIP,YAP1,WWTR1,TEAD1,TEAD4,TEAD3,TEAD2,RASSF2,RASSF4,PAK1",Hippo signaling pathway - multiple species,29

hsa04510,"COL1A1,COL1A2,COL2A1,COL4A2,COL4A4,COL4A6,COL4A1,COL4A5,COL4A3,COL6A1,COL6A2,COL6A3,COL6A6,COL6A5,COL9A1,COL9A2,COL9A3,LAMA1,LAMA2,LAMA3,LAMA5,LAMA4,LAMB1,LAMB2,LAMB3,LAMB4,LAMC1,LAMC2,LAMC3,CHAD,RELN,THBS1,COMP,THBS2,THBS3,THBS4,FN1,SPP1,VTN,TNC,TNN,TNR,TNXB,VWF,IBSP,ITGA1,ITGA2,ITGA2B,ITGA3,ITGA4,ITGA5,ITGA6,ITGA7,ITGA8,ITGA9,ITGA10,ITGA11,ITGAV,ITGB1,ITGB3,ITGB4,ITGB5,ITGB6,ITGB7,ITGB8,PDGFA,PDGFB,PDGFC,PDGFD,EGF,IGF1,VEGFA,VEGFB,PGF,VEGFC,VEGFD,HGF,PDGFRA,PDGFRB,IGF1R,KDR,EGFR,FLT1,FLT4,MET,ERBB2,SRC,ARHGAP35,ARHGAP5,RHOA,DIAPH1,ROCK1,ROCK2,MYL2,MYL5,MYL7,MYL9,MYL10,MYL12B,MYL12A,MYLPF,PPP1CA,PPP1CB,PPP1CC,PPP1R12A,PPP1R12B,PPP1R12C,MYLK,MYLK2,MYLK3,MYLK4,ACTB,ACTG1,RASGRF1,CAPN2,ACTN1,ACTN4,TLN1,TLN2,FLNA,FLNC,FLNB,PXN,ILK,ZYX,VASP,VCL,PARVB,PARVA,PARVG,PDPK1,AKT1,AKT2,AKT3,GSK3B,CTNNB1,PRKCA,PRKCB,PRKCG,PTK2,PIK3CA,PIK3CD,PIK3CB,PIK3R1,PIK3R2,PIK3R3,PTEN,VAV3,VAV1,VAV2,RAC1,RAC2,RAC3,PAK1,PAK2,PAK3,PAK4,PAK5,PAK6,BUB1B-PAK6,CDC42,BCAR1,CRK,CRKL,DOCK1,RAPGEF1,RAP1A,RAP1B,MAPK8,MAPK10,MAPK9,JUN,BRAF,CAV1,CAV2,CAV3,FYN,SHC1,SHC2,SHC3,SHC4,GRB2,SOS1,SOS2,HRAS,RAF1,MAP2K1,MAPK1,MAPK3,ELK1,CCND1,CCND2,CCND3,BIRC2,BIRC3,XIAP,BAD,BCL2,PIP5K1C",Focal adhesion,199

hsa04512,"COL1A1,COL1A2,COL2A1,COL4A2,COL4A4,COL4A6,COL4A1,COL4A5,COL4A3,COL6A1,COL6A2,COL6A3,COL6A6,COL6A5,COL9A1,COL9A2,COL9A3,LAMA1,LAMA2,LAMA3,LAMA5,LAMA4,LAMB1,LAMB2,LAMB3,LAMB4,LAMC1,LAMC2,LAMC3,CHAD,RELN,THBS1,COMP,THBS2,THBS3,THBS4,FN1,SPP1,VTN,TNC,TNN,TNR,TNXB,NPNT,FRAS1,FREM2,FREM1,DSPP,VWF,IBSP,DMP1,AGRN,HSPG2,ITGA1,ITGA2,ITGA2B,ITGA3,ITGA4,ITGA5,ITGA6,ITGA7,ITGA8,ITGA9,ITGA10,ITGA11,ITGAV,ITGB1,ITGB3,ITGB4,ITGB5,ITGB6,ITGB7,ITGB8,CD44,SDC1,SDC4,SV2C,SV2B,SV2A,CD36,GP5,GP1BA,GP1BB,GP9,GP6,DAG1,CD47,HMMR",ECM-receptor interaction,88

hsa04514,"CD58,CD2,VSIR,IGSF11,CD80,CD274,CD28,CD86,CTLA4,ICOSLG,LOC102723996,ICOS,HLA-DMA,HLA-DMB,HLA-DOA,HLA-DOB,HLA-DPA1,HLA-DPB1,HLA-DQA1,HLA-DQA2,HLA-DQB1,HLA-DRA,HLA-DRB1,HLA-DRB3,HLA-DRB4,HLA-DRB5,CD4,HLA-A,HLA-B,HLA-C,HLA-F,HLA-G,HLA-E,CD8A,CD8B,PDCD1LG2,CD276,VTCN1,PDCD1,CD40,CD40LG,ALCAM,CD6,PVR,CD226,NECTIN2,TIGIT,ITGAL,ITGB2,ICAM1,ICAM2,ICAM3,CD22,PTPRC,SIGLEC1,SPN,NECTIN3,CLDN4,CLDN3,CLDN7,CLDN19,CLDN16,CLDN14,CLDN15,CLDN17,CLDN20,CLDN11,CLDN18,CLDN22,CLDN5,CLDN10,CLDN8,CLDN6,CLDN2,CLDN1,CLDN9,CLDN23,CLDN25,CLDN24,OCLN,F11R,JAM2,JAM3,ESAM,CDH5,PECAM1,CD99,ITGAM,SELPLG,SELP,ITGA4,ITGB1,ITGA9,VCAM1,ITGB7,MADCAM1,SELL,CD34,GLG1,SELE,NECTIN1,CDH2,NCAM1,NCAM2,L1CAM,CADM1,NEGR1,NTNG1,LRRC4C,NTNG2,LRRC4,PTPRF,LRRC4B,SDC1,SDC2,SDC3,SDC4,ITGAV,ITGB8,ITGA8,NRXN1,NRXN2,NRXN3,NLGN1,NLGN2,NLGN3,NLGN4X,NLGN4Y,CADM3,NRCAM,CNTN1,PTPRM,CNTN2,NFASC,CNTNAP1,CNTNAP2,MPZ,MPZL1,MAG,CDH1,VCAN,ITGA6,CDH3,CDH4,CDH15,NEO1",Cell adhesion molecules (CAMs),146

hsa04520,"NECTIN1,NECTIN2,NECTIN3,NECTIN4,PARD3,SRC,FARP2,CDC42,RAC1,RAC2,RAC3,WAS,WASL,IQGAP1,BAIAP2,WASF1,WASF2,WASF3,AFDN,LMO7,SSX2IP,SORBS1,ACTN1,ACTN4,VCL,TJP1,CDH1,CTNND1,CTNNB1,CTNNA3,CTNNA1,CTNNA2,ACTB,ACTG1,RHOA,PTPRM,PTPRB,PTPRF,PTPN1,PTPN6,PTPRJ,CSNK2A1,CSNK2A2,CSNK2A3,CSNK2B,TCF7,TCF7L1,TCF7L2,LEF1,IGF1R,INSR,MET,EGFR,ERBB2,FGFR1,FYN,YES1,MAPK1,MAPK3,SNAI2,SNAI1,TGFBR1,TGFBR2,SMAD2,SMAD3,SMAD4,CREBBP,EP300,MAP3K7,NLK,FER,ACP1",Adherens junction,72

hsa04530,"CRB3,CLDN4,CLDN3,CLDN7,CLDN19,CLDN16,CLDN14,CLDN15,CLDN17,CLDN20,CLDN11,CLDN18,CLDN22,CLDN5,CLDN10,CLDN8,CLDN6,CLDN2,CLDN1,CLDN9,CLDN23,CLDN25,CLDN24,OCLN,F11R,JAM2,JAM3,BVES,CDC42,PARD6A,PARD6G,PARD6B,MPP5,MPP4,TJP3,PATJ,MPDZ,PRKCZ,PRKCI,AMOT,AMOTL1,AMOTL2,ARHGAP17,RAC1,NF2,LLGL2,LLGL1,DLG1,SCRIB,PPP2CA,PPP2CB,PPP2R1B,PPP2R1A,PPP2R2A,PPP2R2B,PPP2R2C,PPP2R2D,PARD3,TIAM1,TJP1,TJAP1,DLG2,DLG3,NEDD4,NEDD4L,CGN,CGNL1,ARHGEF2,RHOA,GATA4,MARVELD3,MAP3K1,MAPK8,MAPK10,MAPK9,JUN,CD1A,CD1B,CD1C,CD1D,CD1E,CFTR,CDK4,YBX3,SYMPK,PCNA,CCND1,ERBB2,RUNX1,HSPA4,SLC9A3R1,EZR,RDX,MSN,PRKCE,ACTB,ACTG1,CACNA1D,MAP3K5,MAP2K7,SRC,CTTN,HCLS1,ACTR2,ACTR3B,ACTR3C,ACTR3,WHAMM,WAS,WASL,VASP,PRKACA,PRKACB,PRKACG,RAB13,ARHGEF18,ROCK1,ROCK2,MYL2,EPB41L4B,STK11,PRKAA1,PRKAA2,PRKAB1,PRKAB2,PRKAG1,PRKAG3,PRKAG2,MYH15,MYH1,MYH2,MYH3,MYH4,MYH8,MYH9,MYH10,MYH11,MYH7B,MYH14,MYH13,MYL6B,MYL6,MYL9,MYL12B,MYL12A,IGSF5,MAGI1,SYNPO,ACTN1,ACTN4,MICALL2,RAB8A,RAB8B,RAPGEF6,RAP1A,ITGB1,AFDN,TJP2,RAPGEF2,RAP2C,MARVELD2,TUBA1B,TUBA4A,TUBA3C,TUBA1A,TUBA1C,TUBA8,TUBA3E,TUBA3D,TUBAL3",Tight junction,170

hsa04540,"GJA1,GJD2,LPAR1,GNAI1,GNAI3,GNAI2,PDGFA,PDGFB,PDGFC,PDGFD,EGF,PDGFRA,PDGFRB,EGFR,GRB2,SOS1,SOS2,HRAS,KRAS,NRAS,RAF1,MAP2K1,MAP2K2,MAPK1,MAPK3,SRC,MAP3K2,MAP2K5,MAPK7,TUBA1B,TUBA4A,TUBA3C,TUBA1A,TUBA1C,TUBA8,TUBA3E,TUBA3D,TUBAL3,TUBB6,TUBB,TUBB1,TUBB2A,TUBB3,TUBB4A,TUBB8,TUBB2B,TUBB4B,CSNK1D,CDK1,TJP1,ADRB1,DRD1,GNAS,DRD2,ADCY1,ADCY2,ADCY3,ADCY4,ADCY5,ADCY6,ADCY7,ADCY8,ADCY9,PRKACA,PRKACB,PRKACG,HTR2A,HTR2B,HTR2C,GRM1,GRM5,GNA11,GNAQ,PLCB1,PLCB2,PLCB3,PLCB4,ITPR1,ITPR2,ITPR3,PRKCA,PRKCB,PRKCG,GUCY1A2,GUCY1A1,GUCY1B1,PRKG1,PRKG2",Gap junction,88

hsa04550,"LIF,LIFR,IL6ST,JAK1,JAK2,JAK3,STAT3,KLF4,SOX2,MYC,GRB2,MAP2K1,MAP2K2,MAPK1,MAPK3,PIK3CA,PIK3CD,PIK3CB,PIK3R1,PIK3R2,PIK3R3,AKT1,AKT2,AKT3,TBX3,NANOG,INHBA,INHBB,INHBC,INHBE,NODAL,ACVR1B,ACVR1C,ACVR2A,ACVR2B,SMAD2,SMAD3,BMP4,BMPR1A,BMPR1B,ACVR1,BMPR2,SMAD1,SMAD5,SMAD9,SMAD4,ID1,ID2,ID3,ID4,DUSP9,MAPK11,MAPK12,MAPK13,MAPK14,WNT1,WNT2,WNT2B,WNT3,WNT3A,WNT4,WNT5A,WNT5B,WNT6,WNT7A,WNT7B,WNT8A,WNT8B,WNT9A,WNT9B,WNT10B,WNT10A,WNT11,WNT16,FZD1,FZD7,FZD2,FZD3,FZD4,FZD5,FZD8,FZD6,FZD10,FZD9,DVL3,DVL2,DVL1,GSK3B,AXIN1,AXIN2,APC,APC2,CTNNB1,TCF3,ESRRB,TCF7,POU5F1,POU5F1B,FGF2,FGFR1,FGFR2,FGFR3,FGFR4,HRAS,KRAS,NRAS,RAF1,IGF1,IGF1R,HESX1,ZIC3,LEFTY1,LEFTY2,SKIL,SMARCAD1,KAT6A,SETDB1,JARID2,REST,RIF1,PCGF1,PCGF2,PCGF3,BMI1,COMMD3-BMI1,PCGF5,PCGF6,PAX6,MEIS1,LHX5,OTX1,NEUROG1,HAND1,DLX5,MYF5,ONECUT1,ISL1,ZFHX3,ESX1,HOXB1",Signaling pathways regulating pluripotency of stem cells,140

hsa04610,"F3,F7,F10,F5,F2,F12,F11,F9,VWF,F8,THBD,PROCR,PROC,F2R,F2RL2,F2RL3,F13A1,F13B,CPB2,FGA,FGB,FGG,KLKB1,KNG1,BDKRB1,BDKRB2,PLG,TFPI,SERPINC1,SERPIND1,SERPINA5,PROS1,SERPINE1,SERPINB2,PLAT,PLAU,PLAUR,SERPINA1,SERPINF2,A2M,CFB,CFD,C3,C5,C6,C7,C8A,C8B,C8G,C9,C1QA,C1QB,C1QC,C1R,C1S,MBL2,MASP1,MASP2,C2,C4A,C4B,C3AR1,VSIG4,CR1,CR2,ITGAM,ITGB2,ITGAX,C5AR1,CFH,CFI,SERPING1,CD55,CD46,C4BPA,C4BPB,CD59,CLU,VTN",Complement and coagulation cascades,79

hsa04611,"TBXA2R,F2,F2R,F2RL3,GNA13,ARHGEF1,ARHGEF12,RHOA,ROCK1,ROCK2,PPP1CA,PPP1CB,PPP1CC,PPP1R12A,MYL12B,MYL12A,P2RX1,ORAI1,ITPR1,ITPR2,ITPR3,STIM1,MYLK,MYLK2,MYLK3,MYLK4,P2RY1,GNAQ,PLCB1,PLCB2,PLCB3,PLCB4,PRKCZ,PRKCI,RASGRP1,RASGRP2,RAP1A,RAP1B,APBB1IP,TLN1,TLN2,ITGA2B,ITGB3,FERMT3,FGA,FGB,FGG,P2RY12,GNAI1,GNAI3,GNAI2,ADCY1,ADCY2,ADCY3,ADCY4,ADCY5,ADCY6,ADCY7,ADCY8,ADCY9,PTGIR,GNAS,PIK3CG,PIK3R5,PIK3R6,PRKACA,PRKACB,PRKACG,VASP,ACTB,ACTG1,SRC,ARHGAP35,FCGR2A,SYK,COL1A1,COL1A2,COL3A1,GP6,FCER1G,LYN,FYN,ITGA2,ITGB1,PIK3CA,PIK3CD,PIK3CB,PIK3R1,PIK3R2,PIK3R3,LCP2,PLCG2,BTK,VWF,GP5,GP1BA,GP1BB,GP9,AKT1,AKT2,AKT3,NOS3,GUCY1A2,GUCY1A1,GUCY1B1,PRKG1,PRKG2,MAPK11,MAPK12,MAPK13,MAPK14,MAPK1,MAPK3,PLA2G4E,PLA2G4A,JMJD7-PLA2G4B,PLA2G4B,PLA2G4C,PLA2G4D,PLA2G4F,PTGS1,TBXAS1,SNAP23,VAMP8",Platelet activation,124

hsa04612,"IFNG,TNF,PSME1,PSME2,PSME3,HSPA8,HSPA1A,HSPA2,HSPA1L,HSPA1B,HSPA6,HSPA4,HSP90AA1,HSP90AB1,HLA-A,HLA-B,HLA-C,HLA-F,HLA-G,HLA-E,CANX,B2M,PDIA3,CALR,TAPBP,TAP1,TAP2,CD8A,CD8B,KIR3DL2,KIR3DL1,KIR3DL3,KIR2DL2,KIR2DL1,KIR2DL3,KIR2DL4,KIR2DL5A,KLRC1,KLRC2,KLRC3,KLRC4,KLRD1,KIR2DS1,KIR2DS3,KIR2DS4,KIR2DS5,KIR2DS2,IFI30,LGMN,CTSB,HLA-DMA,HLA-DMB,HLA-DOA,HLA-DOB,HLA-DPA1,HLA-DPB1,HLA-DQA1,HLA-DQA2,HLA-DQB1,HLA-DRA,HLA-DRB1,HLA-DRB3,HLA-DRB4,HLA-DRB5,CD74,CTSL,CTSS,CD4,CIITA,RFX5,RFXANK,RFXAP,CREB1,NFYA,NFYB,NFYC,HSPA5",Antigen processing and presentation,77

hsa04614,"AGT,REN,ACE,CMA1,CTSG,KLK2,KLK1,ENPEP,ANPEP,PREP,ACE2,CTSA,CPA3,MME,THOP1,NLN,PRCP,MAS1,MRGPRD,AGTR1,AGTR2,LNPEP,ATP6AP2",Renin-angiotensin system,23

hsa04620,"TLR1,TLR2,TLR6,LBP,CD14,LY96,TLR3,TLR4,TLR5,TLR7,TLR8,CTSK,TLR9,RAC1,PIK3CA,PIK3CD,PIK3CB,PIK3R1,PIK3R2,PIK3R3,AKT1,AKT2,AKT3,TOLLIP,MYD88,TIRAP,FADD,CASP8,IRAK4,IRAK1,TRAF6,TAB1,TAB2,MAP3K7,IKBKG,CHUK,IKBKB,NFKBIA,NFKB1,RELA,MAP3K8,MAP2K1,MAP2K2,MAPK1,MAPK3,MAP2K3,MAP2K6,MAP2K4,MAP2K7,MAPK11,MAPK12,MAPK13,MAPK14,MAPK8,MAPK10,MAPK9,JUN,FOS,TNF,IL1B,IL6,IL12A,IL12B,CXCL8,CCL5,CCL3,CCL3L1,CCL3L3,CCL4,CCL4L2,CCL4L1,TICAM2,TICAM1,RIPK1,IRF5,IRF7,SPP1,IKBKE,TBK1,TRAF3,IRF3,CD40,CD80,CD86,IFNA1,IFNA2,IFNA4,IFNA5,IFNA6,IFNA7,IFNA8,IFNA10,IFNA13,IFNA14,IFNA16,IFNA17,IFNA21,IFNB1,IFNAR1,IFNAR2,STAT1,CXCL10,CXCL9,CXCL11",Toll-like receptor signaling pathway,104

hsa04621,"NOD1,RIPK2,IKBKG,CHUK,IKBKB,NFKBIB,NFKBIA,NFKB1,RELA,IL1B,IL18,IL6,TNF,CXCL8,CXCL1,CXCL2,CXCL3,CCL2,CCL5,CAMP,DEFA1,DEFA3,DEFA4,DEFA5,DEFA6,DEFA1B,DEFB4A,DEFB4B,DEFB103A,DEFB103B,NOD2,MAP3K7,TAB1,TAB2,TAB3,MAPK1,MAPK3,MAPK8,MAPK10,MAPK9,MAPK11,MAPK12,MAPK13,MAPK14,JUN,ATG16L1,ATG5,ATG12,GABARAP,GABARAPL1,GABARAPL2,SUGT1,SHARPIN,RBCK1,RNF31,XIAP,CARD6,TRIP6,ERBIN,BIRC2,BIRC3,TRAF2,TRAF5,TRAF6,CARD9,TNFAIP3,MAVS,TRAF3,TBK1,IKBKE,TANK,IRF3,IRF7,IFNA1,IFNA2,IFNA4,IFNA5,IFNA6,IFNA7,IFNA8,IFNA10,IFNA13,IFNA14,IFNA16,IFNA17,IFNA21,IFNB1,NLRX1,ANTXR2,ANTXR1,NLRP1,PYCARD,CASP1,BCL2,BCL2L1,CTSB,NLRP3,PANX1,P2RX7,TRPM2,TRPM7,TRPV2,CASR,GPRC6A,PLCB1,PLCB2,PLCB3,PLCB4,ITPR1,ITPR2,ITPR3,VDAC1,VDAC2,VDAC3,MCU,OAS1,OAS2,OAS3,RNASEL,DHX33,MFN1,MFN2,RIPK1,RIPK3,DNM1L,NAMPT,CYBB,CYBA,TXNIP,TXN,TXN2,NEK7,BRCC3,HSP90AA1,HSP90AB1,CARD8,PYDC1,PYDC2,MEFV,PSTPIP1,CASP8,FADD,CASP12,CARD18,CARD17,CARD16,NLRP6,NLRP7,NLRP12,NAIP,NLRC4,PRKCD,GBP2,GBP5,AIM2,IFI16,TP53BP1,PYDC5,RHOA,GBP1,GBP3,GBP4,GBP7,CASP5,CASP4,GSDMD,IFNAR1,IFNAR2,JAK1,TYK2,STAT1,STAT2,IRF9,TLR4,TICAM1,MYD88,IRAK4,STING1",NOD-like receptor signaling pathway,178

hsa04622,"DDX58,IFIH1,MAVS,DHX58,TRAF3,TANK,AZI2,TBKBP1,IKBKG,TBK1,IKBKE,IRF3,IRF7,IFNA1,IFNA2,IFNA4,IFNA5,IFNA6,IFNA7,IFNA8,IFNA10,IFNA13,IFNA14,IFNA16,IFNA17,IFNA21,IFNB1,IFNW1,IFNE,IFNK,TRADD,FADD,RIPK1,CASP8,CASP10,CHUK,IKBKB,NFKBIB,NFKBIA,NFKB1,RELA,TRAF2,MAP3K7,TRAF6,MAP3K1,MAPK8,MAPK10,MAPK9,MAPK11,MAPK12,MAPK13,MAPK14,CXCL8,TNF,IL12A,IL12B,CXCL10,TRIM25,CYLD,RNF125,ISG15,ATG5,ATG12,NLRX1,STING1,OTUD5,SIKE1,DDX3X,PIN1,TKFC",RIG-I-like receptor signaling pathway,70

hsa04623,"POLR3A,POLR3B,POLR3C,POLR3D,POLR3E,POLR1C,POLR3K,POLR1D,POLR3H,POLR3GL,POLR3G,POLR3F,POLR2E,POLR2F,POLR2H,POLR2K,POLR2L,DDX58,MAVS,NFKB1,RELA,IL6,CGAS,STING1,TBK1,IKBKE,IRF3,IRF7,IFNA1,IFNA2,IFNA4,IFNA5,IFNA6,IFNA7,IFNA8,IFNA10,IFNA13,IFNA14,IFNA16,IFNA17,IFNA21,IFNB1,ZBP1,RIPK1,RIPK3,IKBKG,CHUK,IKBKB,NFKBIB,NFKBIA,CCL4,CCL4L2,CCL4L1,CCL5,CXCL10,AIM2,PYCARD,CASP1,IL1B,IL18,IL33,TREX1,ADAR",Cytosolic DNA-sensing pathway,63

hsa04625,"CLEC7A,LSP1,HRAS,KRAS,NRAS,MRAS,RRAS,RRAS2,RAF1,RELA,RELB,IL12A,IL12B,IL1B,SYK,PTPN11,MAP3K14,CHUK,NFKB2,PLCG2,ITPR1,ITPR2,ITPR3,CALML3,CALM2,CALM3,CALM1,CALML6,CALML5,CALML4,PPP3CA,PPP3CB,PPP3CC,PPP3R1,PPP3R2,NFATC1,NFATC2,NFATC3,NFATC4,IL2,IL10,EGR2,EGR3,PTGS2,PRKCD,MAPK1,MAPK3,NLRP3,PYCARD,CASP1,CARD9,BCL10,MALT1,CASP8,IKBKG,IKBKB,NFKBIA,NFKB1,TNF,IL6,IL23A,MAPK11,MAPK12,MAPK13,MAPK14,MAPK8,MAPK10,MAPK9,JUN,CBLB,CLEC6A,FCER1G,CLEC4M,CD209,ARHGEF12,RHOA,PAK1,KSR1,PLK3,SRC,IKBKE,STAT1,IRF9,STAT2,IL17D,CYLD,BCL3,CCL17,CCL22,MAPKAPK2,CLEC4E,PIK3CA,PIK3CD,PIK3CB,PIK3R1,PIK3R2,PIK3R3,AKT1,AKT2,AKT3,MDM2,IRF1,CLEC4D,CLEC1B",C-type lectin receptor signaling pathway,104

hsa04630,"IL2,IL3,IL4,IL5,IL6,IL7,IL9,IL10,IL11,IL12A,IL12B,IL13,IL15,IL17D,IL19,IL20,IL21,IL22,IL23A,IL24,IFNA1,IFNA2,IFNA4,IFNA5,IFNA6,IFNA7,IFNA8,IFNA10,IFNA13,IFNA14,IFNA16,IFNA17,IFNA21,IFNB1,IFNG,IFNE,IFNK,IFNL1,IFNL2,IFNL3,IFNW1,OSM,LIF,TSLP,CTF1,CSF2,CNTF,CSF3,EPO,GH1,GH2,CSH1,CSH2,LEP,THPO,PRL,EGF,PDGFA,PDGFB,IL2RA,IL2RB,IL2RG,IL3RA,IL4R,IL5RA,IL6R,IL7R,IL9R,IL10RA,IL10RB,IL11RA,IL12RB1,IL12RB2,IL13RA1,IL13RA2,IL15RA,IL20RA,IL20RB,IL21R,IL22RA1,IL22RA2,IL23R,IL27RA,IL6ST,IFNAR1,IFNAR2,IFNGR1,IFNGR2,IFNLR1,OSMR,LIFR,CRLF2,CNTFR,CSF2RA,CSF2RB,CSF3R,EPOR,GHR,LEPR,MPL,PRLR,EGFR,PDGFRA,PDGFRB,JAK1,JAK2,JAK3,TYK2,STAT1,STAT2,STAT3,STAT4,STAT5A,STAT5B,STAT6,CISH,SOCS1,SOCS2,SOCS3,SOCS4,SOCS5,SOCS7,SOCS6,BCL2,MCL1,BCL2L1,PIM1,MYC,CCND1,CCND2,CCND3,CDKN1A,AOX1,GFAP,STAM2,STAM,PTPN2,PTPN6,IRF9,CREBBP,EP300,PIAS1,PIAS2,PIAS3,PIAS4,FHL1,PTPN11,GRB2,SOS1,SOS2,HRAS,RAF1,PIK3CA,PIK3CD,PIK3CB,PIK3R1,PIK3R2,PIK3R3,AKT1,AKT2,AKT3,MTOR",Jak-STAT signaling pathway,162

hsa04640,"KITLG,IL7,IL4,CSF2,FLT3LG,IL5,CSF3,IL3,IL6,IL11,IL1A,IL1B,TNF,CSF1,EPO,THPO,CD34,FLT3,DNTT,HLA-DMA,HLA-DMB,HLA-DOA,HLA-DOB,HLA-DPA1,HLA-DPB1,HLA-DQA1,HLA-DQA2,HLA-DQB1,HLA-DRA,HLA-DRB1,HLA-DRB3,HLA-DRB4,HLA-DRB5,CD44,KIT,IL2RA,IL7R,TFRC,CD38,CD7,CD2,CD5,CD1A,CD1B,CD1C,CD1D,CD1E,CD4,CD8A,CD8B,CD3D,CD3E,CD3G,MME,CD9,CD19,CD22,CD24,MS4A1,CR2,CD37,LOC102723407,FCER2,CR1,CSF2RA,IL3RA,CD33,IL4R,IL6R,FCGR1A,CSF1R,ANPEP,ITGAM,CD14,IL9R,IL1R1,IL1R2,CSF3R,IL5RA,EPOR,CD36,GYPA,CD55,CD59,IL11RA,ITGB3,ITGA2B,GP9,GP1BA,GP1BB,GP5,ITGA1,ITGA2,ITGA3,ITGA4,ITGA5,ITGA6",Hematopoietic cell lineage,97

hsa04650,"HLA-A,HLA-B,HLA-C,HLA-F,HLA-G,HLA-E,KIR3DL2,KIR3DL1,KIR3DL3,KIR2DL2,KIR2DL1,KIR2DL3,KIR2DL4,KIR2DL5A,KLRC1,KLRC2,KLRC3,KLRC4,KLRD1,PTPN6,PTPN11,ICAM1,ICAM2,ITGAL,ITGB2,PTK2B,VAV3,VAV1,VAV2,RAC1,RAC2,RAC3,PAK1,MAP2K1,MAP2K2,MAPK1,MAPK3,TNF,CSF2,IFNG,KIR2DS1,KIR2DS3,KIR2DS4,KIR2DS5,KIR2DS2,NCR2,TYROBP,LCK,LOC102723407,FCGR3A,FCGR3B,NCR1,NCR3,FCER1G,CD247,ZAP70,SYK,LCP2,LAT,PLCG1,PLCG2,SH3BP2,PIK3CA,PIK3CD,PIK3CB,PIK3R1,PIK3R2,PIK3R3,FYN,SHC1,SHC2,SHC3,SHC4,GRB2,SOS1,SOS2,HRAS,KRAS,NRAS,ARAF,BRAF,RAF1,MICB,MICA,ULBP1,ULBP2,ULBP3,RAET1G,RAET1L,RAET1E,KLRK1,KLRC4-KLRK1,HCST,CD48,CD244,PPP3CA,PPP3CB,PPP3CC,PPP3R1,PPP3R2,NFATC1,NFATC2,PRKCA,PRKCB,PRKCG,SH2D1B,SH2D1A,IFNGR1,IFNGR2,IFNA1,IFNA2,IFNA4,IFNA5,IFNA6,IFNA7,IFNA8,IFNA10,IFNA13,IFNA14,IFNA16,IFNA17,IFNA21,IFNB1,IFNAR1,IFNAR2,TNFSF10,TNFRSF10A,TNFRSF10B,FASLG,FAS,GZMB,PRF1,CASP3,BID",Natural killer cell mediated cytotoxicity,134

hsa04657,"IL25,IL17RA,IL17RB,TRADD,FADD,CASP3,CASP8,TRAF3IP2,TRAF6,NFKB1,RELA,FOS,FOSB,JUN,JUND,FOSL1,IL4,IL5,IL13,CCL17,CCL11,IL17A,IL17F,IL17RC,TRAF3,ANAPC5,TNFAIP3,HSP90AA1,HSP90AB1,HSP90B1,TBK1,TAB2,TAB3,MAP3K7,IKBKG,CHUK,IKBKB,NFKBIA,MAPK11,MAPK12,MAPK13,MAPK14,MAPK8,MAPK10,MAPK9,MAPK1,MAPK3,MAPK4,MAPK6,MAPK7,MAPK15,CEBPB,TRAF4,IKBKE,USP25,TRAF5,TRAF2,SRSF1,ELAVL1,GSK3B,CXCL1,CXCL2,CXCL3,CXCL5,CXCL6,CXCL8,CXCL10,CCL2,CCL7,CCL20,IL6,TNF,PTGS2,CSF3,CSF2,DEFB4A,DEFB4B,MUC5AC,MUC5B,S100A7,S100A8,S100A9,LCN2,MMP1,MMP3,MMP9,MMP13,IL17C,IL17RE,IL1B,IFNG,IL17B,IL17D",IL-17 signaling pathway,93

hsa04658,"DLL3,DLL1,DLL4,NOTCH3,MAML3,MAML2,MAML1,RBPJL,RBPJ,NFKB1,RELA,IFNG,IFNGR1,IFNGR2,JAK1,JAK2,STAT1,IL12A,IL12B,IL12RB1,IL12RB2,TYK2,STAT4,TBX21,RUNX3,HLA-DMA,HLA-DMB,HLA-DOA,HLA-DOB,HLA-DPA1,HLA-DPB1,HLA-DQA1,HLA-DQA2,HLA-DQB1,HLA-DRA,HLA-DRB1,HLA-DRB3,HLA-DRB4,HLA-DRB5,CD4,LCK,CD3E,CD3G,CD247,CD3D,ZAP70,LAT,PLCG1,PPP3CA,PPP3CB,PPP3CC,PPP3R1,PPP3R2,NFATC1,NFATC2,NFATC3,PRKCQ,CHUK,IKBKB,IKBKG,NFKBIA,NFKBIB,NFKBIE,MAPK1,MAPK3,FOS,MAPK11,MAPK12,MAPK13,MAPK14,MAPK8,MAPK10,MAPK9,JUN,IL2,IL2RA,IL2RB,IL2RG,JAK3,STAT5A,STAT5B,IL4,IL4R,STAT6,GATA3,IL5,IL13,MAF,JAG1,JAG2,NOTCH1,NOTCH2",Th1 and Th2 cell differentiation,92

hsa04659,"IL1B,IL1R1,IL1RAP,MAPK11,MAPK12,MAPK13,MAPK14,MTOR,IRF4,TGFB1,TGFBR1,TGFBR2,SMAD2,SMAD3,SMAD4,IL21,IL21R,IL2RG,JAK1,JAK3,IL6,IL6R,IL6ST,JAK2,IL23A,IL23R,IL12RB1,TYK2,STAT3,RORC,RORA,HIF1A,HSP90AA1,HSP90AB1,AHR,IL17A,IL17F,IL22,HLA-DMA,HLA-DMB,HLA-DOA,HLA-DOB,HLA-DPA1,HLA-DPB1,HLA-DQA1,HLA-DQA2,HLA-DQB1,HLA-DRA,HLA-DRB1,HLA-DRB3,HLA-DRB4,HLA-DRB5,CD4,LCK,CD3E,CD3G,CD247,CD3D,ZAP70,LAT,PLCG1,PPP3CA,PPP3CB,PPP3CC,PPP3R1,PPP3R2,NFATC1,NFATC2,NFATC3,PRKCQ,CHUK,IKBKB,IKBKG,NFKBIA,NFKBIB,NFKBIE,NFKB1,RELA,MAPK1,MAPK3,FOS,MAPK8,MAPK10,MAPK9,JUN,IL4,IL4R,STAT6,GATA3,RUNX1,IL17D,IL27RA,IFNG,IFNGR1,IFNGR2,STAT1,TBX21,IL2,IL2RA,IL2RB,STAT5A,STAT5B,FOXP3,RARA,RXRA,RXRB,RXRG",Th17 cell differentiation,107

hsa04660,"CD3D,CD3E,CD3G,CD247,CD4,CD8A,CD8B,PTPRC,LCK,FYN,ZAP70,LCP2,LAT,ITK,TEC,NCK1,NCK2,VAV3,VAV1,VAV2,GRAP2,GRB2,PAK1,PAK2,PAK3,PAK4,PAK5,PAK6,BUB1B-PAK6,RHOA,CDC42,DLG1,MAPK11,MAPK12,MAPK13,MAPK14,PLCG1,PPP3CA,PPP3CB,PPP3CC,PPP3R1,PPP3R2,NFATC1,NFATC2,NFATC3,SOS1,SOS2,RASGRP1,HRAS,KRAS,NRAS,RAF1,MAP2K1,MAP2K2,MAPK1,MAPK3,FOS,JUN,PRKCQ,CARD11,BCL10,MALT1,MAP3K7,MAP2K7,MAPK8,MAPK10,MAPK9,CHUK,IKBKB,IKBKG,NFKB1,RELA,NFKBIA,NFKBIB,NFKBIE,CD28,ICOS,CD40LG,PIK3R1,PIK3R2,PIK3R3,PIK3CA,PIK3CD,PIK3CB,PDPK1,AKT1,AKT2,AKT3,MAP3K8,MAP3K14,GSK3B,PDCD1,CTLA4,PTPN6,CBLB,IL2,IL4,IL5,IL10,IFNG,CSF2,TNF,CDK4",T cell receptor signaling pathway,103

hsa04662,"LOC102723407,CD79A,CD79B,LYN,SYK,BTK,DAPP1,BLNK,VAV3,VAV1,VAV2,RAC1,RAC2,RAC3,PLCG2,PPP3CA,PPP3CB,PPP3CC,PPP3R1,PPP3R2,NFATC1,NFATC2,NFATC3,GRB2,SOS1,SOS2,RASGRP3,HRAS,KRAS,NRAS,RAF1,MAP2K1,MAP2K2,MAPK1,MAPK3,FOS,JUN,PRKCB,CARD11,BCL10,MALT1,CHUK,IKBKB,IKBKG,NFKB1,RELA,NFKBIA,NFKBIB,NFKBIE,IFITM1,CD81,CD19,CR2,PIK3R1,PIK3R2,PIK3R3,PIK3CA,PIK3CD,PIK3CB,AKT1,AKT2,AKT3,GSK3B,FCGR2B,INPP5D,INPPL1,LILRB2,LILRB1,LILRB5,LILRB4,LILRA1,LILRB3,LILRA3,LILRA2,LILRA4,LILRA6,LILRA5,LOC102725035,CD22,CD72,PTPN6,PIK3AP1",B cell receptor signaling pathway,82

hsa04664,"LOC102723407,FCER1A,MS4A2,FCER1G,SYK,LYN,BTK,INPP5D,PLCG1,PLCG2,PRKCA,PIK3CA,PIK3CD,PIK3CB,PIK3R1,PIK3R2,PIK3R3,PDPK1,AKT1,AKT2,AKT3,RAC1,RAC2,RAC3,MAP2K4,MAP2K7,MAP2K3,MAP2K6,MAPK8,MAPK10,MAPK9,MAPK11,MAPK12,MAPK13,MAPK14,IL4,IL13,IL3,IL5,CSF2,TNF,LCP2,VAV3,VAV1,VAV2,FYN,GAB2,LAT,GRB2,SOS1,SOS2,HRAS,KRAS,NRAS,RAF1,MAP2K1,MAP2K2,MAPK1,MAPK3,PLA2G4E,PLA2G4A,JMJD7-PLA2G4B,PLA2G4B,PLA2G4C,PLA2G4D,PLA2G4F,ALOX5,ALOX5AP",Fc epsilon RI signaling pathway,68

hsa04666,"LOC102723407,FCGR1A,FCGR2A,FCGR3A,FCGR3B,PTPRC,HCK,LYN,SYK,PIK3CA,PIK3CD,PIK3CB,PIK3R1,PIK3R2,PIK3R3,AKT1,AKT2,AKT3,RPS6KB1,RPS6KB2,PLCG1,PLCG2,PRKCD,PRKCE,RAF1,MAP2K1,MAPK1,MAPK3,PLA2G4E,PLA2G4A,JMJD7-PLA2G4B,PLA2G4B,PLA2G4C,PLA2G4D,PLA2G4F,PLA2G6,MARCKS,MARCKSL1,PLD1,PLD2,PLPP1,PLPP3,PLPP2,SPHK1,SPHK2,PRKCA,PRKCB,PRKCG,NCF1,GSN,SCIN,VAV3,VAV1,VAV2,CDC42,WAS,WASL,VASP,ARPC5,ARPC5L,ARPC4,ARPC3,ARPC1B,ARPC1A,ARPC2,RAC1,RAC2,WASF1,WASF2,WASF3,PAK1,LIMK1,LIMK2,CFL1,CFL2,PIP5K1C,PIP5K1A,PIP5K1B,ARF6,CRK,CRKL,DOCK2,ASAP1,ASAP3,ASAP2,FCGR2B,INPP5D,INPPL1,GAB2,LAT,DNM2,AMPH,BIN1,MYO10",Fc gamma R-mediated phagocytosis,94

hsa04668,"TNF,TNFRSF1A,BAG4,TRADD,TRAF2,TRAF5,RIPK1,BIRC2,BIRC3,MAP3K7,TAB1,TAB2,TAB3,MAP2K4,MAP2K7,MAPK8,MAPK10,MAPK9,JUN,FOS,ITCH,CFLAR,MAP2K3,MAP2K6,MAPK11,MAPK12,MAPK13,MAPK14,CEBPB,MAP3K5,MAP3K14,IKBKG,IKBKB,CHUK,NFKBIA,RELA,NFKB1,MAP3K8,MAP2K1,MAPK1,MAPK3,RPS6KA5,RPS6KA4,CREB1,CREB3,CREB3L1,CREB3L2,CREB3L3,CREB3L4,ATF2,ATF4,CREB5,ATF6B,RIPK3,MLKL,PGAM5,DNM1L,FADD,CASP8,CASP10,CASP7,CASP3,CCL2,CCL5,CCL20,CXCL1,CXCL2,CXCL3,CXCL5,CXCL6,CXCL10,CX3CL1,CSF1,CSF2,FAS,IL18R1,JAG1,IL1B,IL6,IL15,LIF,LTA,BCL3,SOCS3,TNFAIP3,TRAF1,JUNB,MMP3,MMP9,MMP14,EDN1,VEGFC,VEGFD,NOD2,ICAM1,SELE,VCAM1,PTGS2,TNFRSF1B,TRAF3,PIK3CA,PIK3CD,PIK3CB,PIK3R1,PIK3R2,PIK3R3,AKT1,AKT2,AKT3,DAB2IP,IRF1,IFNB1",TNF signaling pathway,112

hsa04670,"JAM3,ITGAM,ITGB2,JAM2,ITGA4,ITGB1,PECAM1,CD99,ITGAL,F11R,CDH5,CLDN4,CLDN3,CLDN7,CLDN19,CLDN16,CLDN14,CLDN15,CLDN17,CLDN20,CLDN11,CLDN18,CLDN22,CLDN5,CLDN10,CLDN8,CLDN6,CLDN2,CLDN1,CLDN9,CLDN23,CLDN25,CLDN24,OCLN,ESAM,VCAM1,EZR,MSN,ACTB,ACTG1,PIK3CA,PIK3CD,PIK3CB,PIK3R1,PIK3R2,PIK3R3,RAC1,CYBB,CYBA,NCF2,NCF1,NCF4,CTNNB1,CTNND1,CTNNA3,CTNNA1,CTNNA2,PTPN11,MMP2,MMP9,MAPK11,MAPK12,MAPK13,MAPK14,ICAM1,PLCG1,PLCG2,PRKCA,PRKCB,PRKCG,PTK2,PXN,BCAR1,THY1,ARHGAP35,ARHGAP5,RHOA,ROCK1,ROCK2,MYL2,MYL5,MYL7,MYL9,MYL10,MYL12B,MYL12A,MYLPF,AFDN,RAP1A,RAP1B,SIPA1,VASP,ACTN1,ACTN4,VCL,CXCL12,CXCR4,GNAI1,GNAI3,GNAI2,RAPGEF3,RAPGEF4,RASSF5,PTK2B,ITK,TXK,VAV3,VAV1,VAV2,RAC2,CDC42,RHOH",Leukocyte transendothelial migration,112

hsa04672,"CD80,CD86,HLA-DMA,HLA-DMB,HLA-DOA,HLA-DOB,HLA-DPA1,HLA-DPB1,HLA-DQA1,HLA-DQA2,HLA-DQB1,HLA-DRA,HLA-DRB1,HLA-DRB3,HLA-DRB4,HLA-DRB5,CD28,IL2,IL4,IL5,IL6,IL10,TGFB1,TNFSF13,TNFSF13B,TNFRSF13B,TNFRSF17,TNFRSF13C,LOC102723407,AICDA,CD40,CD40LG,ICOS,ICOSLG,LOC102723996,CCR9,ITGA4,ITGB7,CXCL12,CXCR4,CCL28,CCR10,CCL25,MADCAM1,LTBR,MAP3K14,IL15,IL15RA,PIGR",Intestinal immune network for IgA production,49

hsa04710,"CSNK1D,CSNK1E,TPTEP2-CSNK1E,PER1,PER2,PER3,CRY1,CRY2,ARNTL,CLOCK,NPAS2,NR1D1,RORA,RORB,RORC,BHLHE40,BHLHE41,RBX1,CUL1,SKP1,BTRC,FBXW11,FBXL3,PRKAA1,PRKAA2,PRKAB1,PRKAB2,PRKAG1,PRKAG3,PRKAG2,CREB1",Circadian rhythm,31

hsa04713,"GRIN1,GRIN2A,GRIN2B,GRIN2C,GRIN2D,CALML3,CALM2,CALM3,CALM1,CALML6,CALML5,CALML4,CAMK2A,CAMK2D,CAMK2B,CAMK2G,NOS1,MAPK1,MAPK3,RPS6KA5,CREB1,ITPR1,ITPR3,RYR1,RYR2,RYR3,NOS1AP,RASD1,GNAI1,GNAI3,GNAI2,GNAO1,GNB1,GNB2,GNB3,GNB4,GNB5,GNG2,GNG3,GNG4,GNG5,GNG7,GNG8,GNG10,GNG11,GNG12,GNG13,GNGT1,GNGT2,GRIA1,GRIA2,GRIA3,GRIA4,CACNA1G,CACNA1H,CACNA1I,ADCYAP1,ADCYAP1R1,GNAS,ADCY1,ADCY2,ADCY3,ADCY4,ADCY5,ADCY6,ADCY7,ADCY8,ADCY9,ADCY10,PRKACA,PRKACB,PRKACG,GUCY1A2,GUCY1A1,GUCY1B1,PRKG1,PRKG2,CACNA1C,CACNA1D,MTNR1B,MTNR1A,GNAQ,PLCB1,PLCB2,PLCB3,PLCB4,PRKCA,PRKCB,PRKCG,KCNJ3,KCNJ5,KCNJ6,KCNJ9,PER1,PER2,PER3,FOS",Circadian entrainment,97

hsa04714,"ADRB3,GNAS,ADCY1,ADCY2,ADCY3,ADCY4,ADCY5,ADCY6,ADCY7,ADCY8,ADCY9,ADCY10,PRKACA,PRKACB,PRKACG,CREB1,CREB3,CREB3L1,CREB3L2,CREB3L3,CREB3L4,CREB5,PRDM16,ZNF516,KDM1A,UCP1,KDM3A,KDM3B,SMARCA2,SMARCA4,SMARCB1,SMARCC1,SMARCC2,SMARCD1,SMARCD2,SMARCD3,SMARCE1,ACTB,ACTG1,ACTL6A,ACTL6B,ARID1B,ARID1A,DPF1,DPF3,PPARG,MAP3K5,MAP2K3,MAPK11,MAPK12,MAPK13,MAPK14,PPARGC1A,ATF2,SIRT6,FGF21,PLIN1,LIPE,PNPLA2,MGLL,PRKAA1,PRKAA2,PRKAB1,PRKAB2,PRKAG1,PRKAG3,PRKAG2,GCG,BMP8B,BMP8A,CNR1,NPPA,NPPB,NPR1,PRKG1,PRKG2,KLB,FGFR1,FRS2,GRB2,SOS1,SOS2,HRAS,KRAS,NRAS,RPS6KA3,RPS6KA1,RPS6KA2,RPS6KA6,TSC1,TSC2,RHEB,RPTOR,MTOR,MLST8,AKT1S1,RPS6KB1,RPS6KB2,RPS6,ACSL6,ACSL4,ACSL1,ACSL5,ACSL3,ND1,ND2,ND3,ND4,ND4L,ND5,ND6,NDUFS1,NDUFS2,NDUFS3,NDUFS4,NDUFS5,NDUFS6,NDUFS7,NDUFS8,NDUFV1,NDUFV2,NDUFV3,NDUFA1,NDUFA2,NDUFA3,NDUFA4,NDUFA4L2,NDUFA5,NDUFA6,NDUFA7,NDUFA8,NDUFA9,NDUFA10,NDUFAB1,NDUFA11,NDUFA12,NDUFA13,NDUFB1,NDUFB2,NDUFB3,NDUFB4,NDUFB5,NDUFB6,NDUFB7,NDUFB8,NDUFB9,NDUFB10,NDUFB11,NDUFC1,NDUFC2,NDUFC2-KCTD14,NDUFAF1,NDUFAF2,NDUFAF3,NDUFAF4,NDUFAF5,NDUFAF6,NDUFAF7,SDHA,SDHB,SDHC,SDHD,UQCRFS1,CYTB,CYC1,UQCRC1,UQCRC2,UQCRH,UQCRHL,UQCRB,UQCRQ,UQCR10,UQCR11,COX1,COX2,COX3,COX4I2,COX4I1,COX5A,COX5B,COX6A1,COX6A2,COX6B1,COX6B2,COX6C,COX7A1,COX7A2,COX7A2L,COX7B,COX7B2,COX7C,COX8C,COX8A,COX10,COX11,COX14,COX15,COX16,COX17,COX18,COX19,COX20,COA1,COA3,COA4,COA5,COA6,COA7,ATP5F1A,ATP5F1B,ATP5F1C,ATP5F1D,ATP5F1E,ATP5PO,ATP6,ATP5PB,ATP5MC1,ATP5MC2,ATP5MC3,ATP5PD,ATP5ME,ATP5MF,ATP5PF,ATP8,ATP5MG,CPT2,SLC25A29,SLC25A20,CPT1A,CPT1B,CPT1C",Thermogenesis,231

hsa04720,"GRIA1,GRIA2,ADCY1,ADCY8,PRKACA,PRKACB,PRKACG,PPP1R1A,PPP1CA,PPP1CB,PPP1CC,CAMK2A,CAMK2D,CAMK2B,CAMK2G,RAPGEF3,RAP1A,RAP1B,PPP3CA,PPP3CB,PPP3CC,PPP3R1,PPP3R2,GRIN1,GRIN2A,GRIN2B,GRIN2C,GRIN2D,CACNA1C,CALML3,CALM2,CALM3,CALM1,CALML6,CALML5,CALML4,CREBBP,EP300,ATF4,CAMK4,HRAS,KRAS,NRAS,ARAF,BRAF,RAF1,MAP2K1,MAP2K2,MAPK1,MAPK3,RPS6KA3,RPS6KA1,RPS6KA2,RPS6KA6,ITPR1,ITPR2,ITPR3,PRKCA,PRKCB,PRKCG,GRM1,GRM5,GNAQ,PLCB1,PLCB2,PLCB3,PLCB4",Long-term potentiation,67

hsa04721,"SLC32A1,SLC18A1,SLC18A2,SLC18A3,SLC17A6,SLC17A8,SLC17A7,SYT1,VAMP2,RAB3A,RIMS1,STX1A,STX2,STX3,STX1B,STXBP1,UNC13A,UNC13B,UNC13C,SNAP25,CPLX1,CPLX2,CPLX3,CPLX4,CACNA1A,CACNA1B,NSF,NAPA,DNM1,DNM3,DNM2,CLTA,CLTB,CLTC,CLTCL1,AP2A2,AP2A1,AP2B1,AP2M1,AP2S1,ATP6V1A,ATP6V1B1,ATP6V1B2,ATP6V1C2,ATP6V1C1,ATP6V1D,ATP6V1E2,ATP6V1E1,ATP6V1F,ATP6V1G1,ATP6V1G3,ATP6V1G2,ATP6V0E1,ATP6V0E2,TCIRG1,ATP6V0A2,ATP6V0A4,ATP6V0A1,ATP6V0D1,ATP6V0D2,ATP6V1H,ATP6V0C,ATP6V0B,SLC6A1,SLC6A2,SLC6A3,SLC6A4,SLC6A5,SLC6A9,SLC6A7,SLC6A11,SLC6A12,SLC6A13,SLC1A1,SLC1A2,SLC1A3,SLC1A6,SLC1A7",Synaptic vesicle cycle,78

hsa04722,"NGF,BDNF,NTF4,NTF3,NTRK1,NTRK2,NTRK3,SH2B2,SH2B1,SH2B3,GRB2,SOS1,SOS2,HRAS,KRAS,NRAS,RAF1,BRAF,MAP2K1,MAP2K2,MAPK1,MAPK3,RPS6KA3,RPS6KA1,RPS6KA2,RPS6KA6,RPS6KA5,ATF4,BCL2,KIDINS220,FRS2,CRK,CRKL,RAPGEF1,RAP1A,RAP1B,MAP3K3,MAP2K5,MAPK7,MAPK11,MAPK12,MAPK13,MAPK14,MAPKAPK2,SHC1,SHC2,SHC3,SHC4,GAB1,PIK3R1,PIK3R2,PIK3R3,PIK3CA,PIK3CD,PIK3CB,AKT1,AKT2,AKT3,NFKBIB,NFKBIA,NFKBIE,NFKB1,RELA,FOXO3,FASLG,BAD,GSK3B,PDPK1,IRS1,PLCG1,PLCG2,PRKCD,CALML3,CALM2,CALM3,CALM1,CALML6,CALML5,CALML4,CAMK2A,CAMK2D,CAMK2B,CAMK2G,CAMK4,ABL1,PTPN11,MATK,NGFR,ARHGDIB,ARHGDIA,ARHGDIG,RHOA,CDC42,RAC1,MAP3K1,MAP3K5,MAP2K7,MAPK8,MAPK10,MAPK9,JUN,TP53,BAX,TP73,TRAF6,ZNF274,PRDM4,MAGED1,BEX3,YWHAE,RIPK2,IRAK1,IRAK2,IRAK3,IRAK4,IKBKB,SORT1,PSEN1,PSEN2",Neurotrophin signaling pathway,119

hsa04723,"SLC17A6,SLC17A8,SLC17A7,GRIA1,GRIA2,GRIA3,GRIA4,CACNA1C,CACNA1D,CACNA1F,CACNA1S,GRM1,GRM5,GNAQ,PLCB1,PLCB2,PLCB3,PLCB4,ITPR1,ITPR2,ITPR3,NAPEPLD,PRKCA,PRKCB,PRKCG,DAGLA,DAGLB,PTGS2,ABHD6,FAAH,CNR1,GNAI1,GNAI3,GNAI2,GNAO1,GNB1,GNB2,GNB3,GNB4,GNB5,GNG2,GNG3,GNG4,GNG5,GNG7,GNG8,GNG10,GNG11,GNG12,GNG13,GNGT1,GNGT2,KCNJ3,KCNJ6,KCNJ9,KCNJ5,CACNA1A,CACNA1B,MAPK1,MAPK3,MAPK8,MAPK10,MAPK9,MAPK11,MAPK12,MAPK13,MAPK14,ADCY1,ADCY2,ADCY3,ADCY4,ADCY5,ADCY6,ADCY7,ADCY8,ADCY9,PRKACA,PRKACB,PRKACG,MGLL,ND1,ND2,ND3,ND4,ND4L,ND5,ND6,NDUFV1,NDUFV2,NDUFV3,NDUFA1,NDUFA2,NDUFA3,NDUFA4,NDUFA4L2,NDUFA5,NDUFA6,NDUFA7,NDUFA8,NDUFA9,NDUFA10,NDUFAB1,NDUFA11,NDUFA12,NDUFA13,NDUFB1,NDUFB2,NDUFB3,NDUFB4,NDUFB5,NDUFB6,NDUFB7,NDUFB8,NDUFB9,NDUFB10,NDUFB11,NDUFS1,NDUFS2,NDUFS3,NDUFS4,NDUFS5,NDUFS6,NDUFS7,NDUFS8,NDUFC1,NDUFC2,NDUFC2-KCTD14,SLC32A1,RIMS1,GABRA1,GABRA2,GABRA3,GABRA4,GABRA5,GABRA6,GABRB1,GABRB3,GABRB2,GABRG1,GABRG2,GABRG3,GABRD,GABRE,GABRQ,GABRP,GABRR1,GABRR2,GABRR3",Retrograde endocannabinoid signaling,148

hsa04724,"SLC38A1,SLC38A2,GLS2,GLS,SLC17A6,SLC17A8,SLC17A7,GRIK1,GRIK2,GRIK3,GRIK4,GRIK5,GRIA1,GRIA2,GRIA3,GRIA4,GRIN1,GRIN2A,GRIN2B,GRIN2C,GRIN2D,GRIN3A,GRIN3B,PPP3CA,PPP3CB,PPP3CC,PPP3R1,PPP3R2,DLG4,DLGAP1,SHANK3,SHANK1,SHANK2,TRPC1,GRM1,GRM5,HOMER1,HOMER2,HOMER3,GNAQ,PLCB1,PLCB2,PLCB3,PLCB4,PRKCA,PRKCB,PRKCG,ITPR1,ITPR2,ITPR3,PLA2G4E,PLA2G4A,JMJD7-PLA2G4B,PLA2G4B,PLA2G4C,PLA2G4D,PLA2G4F,PLD1,PLD2,MAPK1,MAPK3,GNAS,ADCY1,ADCY2,ADCY3,ADCY4,ADCY5,ADCY6,ADCY7,ADCY8,ADCY9,PRKACA,PRKACB,PRKACG,GRM2,GRM3,GRM4,GRM6,GRM7,GRM8,GNAI1,GNAI3,GNAI2,GNAO1,GNB1,GNB2,GNB3,GNB4,GNB5,GNG2,GNG3,GNG4,GNG5,GNG7,GNG8,GNG10,GNG11,GNG12,GNG13,GNGT1,GNGT2,GRK2,GRK3,KCNJ3,CACNA1A,SLC1A2,SLC1A7,SLC1A1,SLC1A6,CACNA1C,CACNA1D,SLC1A3,GLUL,SLC38A3",Glutamatergic synapse,114

hsa04725,"CHAT,ACHE,SLC18A3,CHRM1,CHRM3,CHRM5,GNAQ,GNA11,PLCB1,PLCB2,PLCB3,PLCB4,ITPR1,ITPR2,ITPR3,PRKCA,PRKCB,PRKCG,KCNQ1,KCNQ2,KCNQ3,KCNQ4,KCNQ5,KCNJ2,KCNJ12,KCNJ4,KCNJ14,CHRM2,CHRM4,GNAI1,GNAI3,GNAI2,GNAO1,GNB1,GNB2,GNB3,GNB4,GNB5,GNG2,GNG3,GNG4,GNG5,GNG7,GNG8,GNG10,GNG11,GNG12,GNG13,GNGT1,GNGT2,KCNJ3,KCNJ6,PIK3CG,PIK3R5,PIK3R6,HRAS,KRAS,NRAS,MAP2K1,MAPK1,MAPK3,FOS,CHRNA7,CHRNA4,CHRNB2,CHRNA3,CHRNB4,CHRNA6,ADCY1,ADCY2,ADCY3,ADCY4,ADCY5,ADCY6,ADCY7,ADCY8,ADCY9,PRKACA,PRKACB,PRKACG,CREB1,ATF4,CREB3,CREB3L1,CREB3L2,CREB3L3,CREB3L4,CREB5,CAMK2A,CAMK2D,CAMK2B,CAMK2G,CAMK4,JAK2,FYN,PIK3CA,PIK3CD,PIK3CB,PIK3R1,PIK3R2,PIK3R3,AKT1,AKT2,AKT3,BCL2,CACNA1A,CACNA1B,CACNA1C,CACNA1D,CACNA1F,CACNA1S,SLC5A7",Cholinergic synapse,112

hsa04726,"TPH2,TPH1,DDC,SLC18A1,SLC18A2,CACNA1C,CACNA1D,CACNA1F,CACNA1S,HTR2A,HTR2B,HTR2C,GNAQ,PLCB1,PLCB2,PLCB3,PLCB4,ITPR1,ITPR2,ITPR3,PRKCA,PRKCB,PRKCG,MAPK1,MAPK3,PLA2G4E,PLA2G4A,JMJD7-PLA2G4B,PLA2G4B,PLA2G4C,PLA2G4D,PLA2G4F,CYP2C8,CYP2C9,CYP2C18,CYP2C19,CYP2D6,LOC107987478,CYP2D7,LOC107987479,CYP2J2,CYP4X1,ALOX5,ALOX12,ALOX12B,ALOX15,ALOX15B,PTGS1,PTGS2,HTR3A,HTR3E,HTR3D,HTR3C,HTR3B,HTR4,HTR6,HTR7,GNAS,ADCY5,PRKACA,PRKACB,PRKACG,KCNN2,KCND2,GABRB1,GABRB3,GABRB2,RAPGEF3,APP,HTR1A,HTR1B,HTR1D,HTR1E,HTR1F,HTR5A,GNAI1,GNAI3,GNAI2,GNAO1,GNB1,GNB2,GNB3,GNB4,GNB5,GNG2,GNG3,GNG4,GNG5,GNG7,GNG8,GNG10,GNG11,GNG12,GNG13,GNGT1,GNGT2,CASP3,DUSP1,HRAS,KRAS,NRAS,ARAF,BRAF,RAF1,MAP2K1,CACNA1A,CACNA1B,KCNJ3,KCNJ6,KCNJ9,KCNJ5,TRPC1,SLC6A4,MAOB,MAOA",Serotonergic synapse,115

hsa04727,"SLC38A1,SLC38A2,GLS2,GLS,GAD1,GAD2,SLC32A1,ABAT,GABRA1,GABRA2,GABRA3,GABRA4,GABRA5,GABRA6,GABRB1,GABRB3,GABRB2,GABRG1,GABRG2,GABRG3,GABRD,GABRE,GABRQ,GABRP,PRKACA,PRKACB,PRKACG,SRC,PRKCA,PRKCB,PRKCG,HAP1,GABARAP,GABARAPL1,GABARAPL2,NSF,TRAK2,PLCL1,GPHN,GABRR1,GABRR2,GABRR3,CACNA1A,CACNA1B,CACNA1C,CACNA1D,CACNA1F,CACNA1S,GABBR1,GABBR2,GNAI1,GNAI3,GNAI2,GNAO1,GNB1,GNB2,GNB3,GNB4,GNB5,GNG2,GNG3,GNG4,GNG5,GNG7,GNG8,GNG10,GNG11,GNG12,GNG13,GNGT1,GNGT2,ADCY1,ADCY2,ADCY3,ADCY4,ADCY5,ADCY6,ADCY7,ADCY8,ADCY9,KCNJ6,SLC6A1,SLC6A13,SLC6A11,SLC6A12,GLUL,SLC38A3,SLC38A5,SLC12A5",GABAergic synapse,89

hsa04728,"TH,DDC,SLC18A1,SLC18A2,DRD1,DRD5,CALY,GNAQ,PLCB1,PLCB2,PLCB3,PLCB4,ITPR1,ITPR2,ITPR3,CALML3,CALM2,CALM3,CALM1,CALML6,CALML5,CALML4,CAMK2A,CAMK2D,CAMK2B,CAMK2G,PPP3CA,PPP3CB,PPP3CC,PRKCA,PRKCB,PRKCG,FOS,GNAS,GNAL,ADCY5,PRKACA,PRKACB,PRKACG,CREB1,ATF2,ATF4,CREB3,CREB3L1,CREB3L2,CREB3L3,CREB3L4,CREB5,ATF6B,MAPK11,MAPK12,MAPK13,MAPK14,MAPK8,MAPK10,MAPK9,PPP1R1B,PPP1CA,PPP1CB,PPP1CC,SCN1A,CACNA1C,CACNA1D,CACNA1A,CACNA1B,KCNJ3,KCNJ6,KCNJ9,KCNJ5,DRD3,DRD4,GNAI1,GNAI3,GNAI2,GNAO1,GNB1,GNB2,GNB3,GNB4,GNB5,GNG2,GNG3,GNG4,GNG5,GNG7,GNG8,GNG10,GNG11,GNG12,GNG13,GNGT1,GNGT2,DRD2,PPP2CA,PPP2CB,PPP2R1B,PPP2R1A,PPP2R2A,PPP2R2B,PPP2R2C,PPP2R2D,PPP2R3B,PPP2R3C,PPP2R3A,PPP2R5B,PPP2R5C,PPP2R5D,PPP2R5E,PPP2R5A,AKT1,AKT2,AKT3,GSK3A,GSK3B,GRIN2A,GRIN2B,GRIA1,GRIA2,GRIA3,GRIA4,KIF5A,KIF5B,KIF5C,CLOCK,ARNTL,SLC6A3,MAOB,MAOA,COMT,LRTOMT,ARRB2",Dopaminergic synapse,131

hsa04730,"NOS1,GUCY1A2,GUCY1A1,GUCY1B1,PRKG1,PRKG2,PPP1R17,PPP2R1B,PPP2R1A,PPP2CA,PPP2CB,HRAS,KRAS,NRAS,ARAF,BRAF,RAF1,MAP2K1,MAP2K2,MAPK1,MAPK3,GRID2,GRM1,GNAI1,GNAI3,GNAI2,GNAO1,GNAZ,GNAS,GNA12,GNA13,PLA2G4E,PLA2G4A,JMJD7-PLA2G4B,PLA2G4B,PLA2G4C,PLA2G4D,PLA2G4F,PRKCA,PRKCB,PRKCG,GNAQ,GNA11,PLCB1,PLCB2,PLCB3,PLCB4,GRIA1,GRIA2,GRIA3,LYN,CACNA1A,ITPR1,ITPR2,ITPR3,RYR1,CRH,CRHR1,IGF1,IGF1R",Long-term depression,60

hsa04740,"OR2J3,OR14J1,OR10C1,OR2A2,OR52K2,OR5P2,OR5P3,OR8I2,OR2D3,OR52E2,OR52J3,OR51L1,OR51A7,OR51S1,OR51F2,OR52R1,OR4C46,OR4X2,OR52M1,OR1E1,OR1E2,OR1D2,OR1D5,OR1G1,OR1A1,OR1A2,OR2J2,OR2A4,OR2C1,OR2F1,OR2W1,OR3A1,OR3A2,OR3A3,OR5I1,OR5V1,OR6A2,OR7A17,OR7A5,OR10H1,OR10H2,OR10H3,OR10J1,OR11A1,OR12D2,OR12D3,OR51E2,OR52A1,OR51B4,OR51B2,OR4N4,OR5B3,OR9K2,OR4Q3,OR4M1,OR13G1,OR2L13,OR52E6,OR52E8,OR52E4,OR56A3,OR56A5,OR10A6,OR4X1,OR5D13,OR5D16,OR8H2,OR8H3,OR5T3,OR5T1,OR8K1,OR5M9,OR5M10,OR5M1,OR9G1,OR2AG1,OR6B3,OR1Q1,OR7D2,OR56B4,OR8U1,OR4C16,OR4C11,OR4S2,OR4C6,OR5D14,OR5L1,OR5D18,OR5AS1,OR8K5,OR5T2,OR8H1,OR8K3,OR8J1,OR5R1,OR5M3,OR5M8,OR5AR1,OR8B12,OR8G5,OR10G8,OR10G9,OR10S1,OR6T1,OR4D5,OR6Q1,OR9I1,OR9Q1,OR9Q2,OR1S2,OR1S1,OR10Q1,OR5B17,OR5B21,OR5A2,OR5A1,OR4D6,OR4D11,OR6C74,OR6C3,OR1L4,OR52B2,OR4C3,OR4S1,OR51F1,OR1C1,OR2B6,OR1J4,OR2M4,OR2L2,OR2K2,OR5L2,OR5K1,OR8G2P,OR8B8,OR10A3,OR7C2,OR7C1,OR4D1,OR2T1,OR2H1,OR4C13,OR4C12,OR51V1,OR8D1,OR8D2,OR8B4,OR9G4,OR10A4,OR6C6,OR2Z1,OR10H5,OR14A16,OR2V2,OR13C9,OR13D1,OR8D4,OR5F1,OR5AP2,OR52L1,OR2AG2,OR52B6,OR2AT4,OR10A2,OR6C2,OR6C4,OR8S1,OR6S1,OR6F1,OR2T3,OR10R2,OR2T29,OR6V1,OR2A12,OR2A1,OR1J1,OR1B1,OR13H1,OR56B1,OR52K1,OR52I1,OR51D1,OR52A5,OR51B6,OR2D2,OR52W1,OR56A4,OR56A1,OR10P1,OR10AD1,OR10A7,OR4K14,OR4L1,OR11H6,OR4D2,OR7D4,OR7G1,OR1M1,OR1I1,OR10H4,OR2M5,OR2M3,OR2T12,OR14C36,OR2T34,OR2T10,OR2T4,OR2T11,OR10J5,OR2B11,OR10T2,OR10X1,OR10Z1,OR6K6,OR6N1,OR9A4,OR2Y1,OR9A2,OR2A14,OR6B1,OR2F2,OR13C5,OR13C8,OR13C3,OR13C4,OR13F1,OR1L8,OR1N2,OR1N1,OR52B4,OR52I2,OR10A5,OR51M1,OR51Q1,OR51I1,OR51I2,OR52D1,OR52H1,OR52N4,OR52N5,OR52N2,OR5AK2,OR5B12,OR5AN1,OR4D10,OR4D9,OR10V1,OR6X1,OR6M1,OR10G4,OR10G7,OR8A1,OR6C1,OR6C75,OR6C76,OR6C70,OR4N2,OR4K2,OR4K13,OR4K17,OR4N5,OR11G2,OR11H4,OR5AU1,OR4M2,OR4F6,OR4F15,OR7G2,OR7G3,OR7A10,OR10K2,OR10K1,OR6Y1,OR6K3,OR11L1,OR2L8,OR2AK2,OR2L3,OR2M2,OR2T33,OR2M7,OR2G6,OR2A25,OR13J1,OR13C2,OR1L6,OR5C1,OR1K1,OR2A5,OR2A7,OR51T1,OR51A4,OR51A2,OR2T2,OR2T5,OR14I1,OR5K2,OR2A42,OR1F1,OR2S2,OR13A1,OR2H2,OR2C3,OR2B2,OR4B1,OR5M11,OR2T6,OR51E1,OR8G1,OR10G3,OR10G2,OR4F4,OR4F3,OR4E2,OR1L3,OR1L1,OR1J2,OR51B5,OR10AG1,OR5J2,OR10J3,OR6B2,OR5H6,OR5H2,OR4K5,OR51G1,OR52N1,OR4F5,OR4K1,OR5AC2,OR11H1,OR4F17,OR4K15,OR8J3,OR51G2,OR4P4,OR4C15,OR4A5,OR4A16,OR4A15,OR2AE1,OR4F16,OR6N2,OR6K2,OR2G3,OR2G2,OR2W3,OR2T8,OR8U8,OR2B3,OR6C65,OR6C68,OR7E24,OR4C45,OR4A47,OR2T27,OR4F21,OR5W2,OR6P1,OR8U9,OR9G9,OR5H1,OR8B3,OR8B2,OR5B2,OR11H12,OR5K3,OR5K4,OR10W1,OR4F29,OR2AP1,OR2V1,OR2L5,LOC102723532,OR5H14,LOC105369274,OR4E1,OR5H15,OR1E3,OR1F12,OR10G6,OR12D1,OR52Z1,OR4K3,OR4Q2,OR10J4,OR10AC1,OR51H1,OR2T35,OR2W5,OR2J1,OR1D4,OR52E1,OR4C5,OR51J1,OR5AL1,LOC107987545,OR6J1,OR2AJ1,LOC112268384,OR10D3,OR14K1,OR14A2,OR52E5,OR11H7,OR11H2,OR2T7,GNAL,GNB1,GNG7,GNG13,ADCY3,CNGA2,CNGA4,CNGB1,ANO2,GUCY2D,CNGA3,NCALD,PRKG1,PRKG2,SLC8A1,SLC8A2,SLC8A3,SLC24A4,GRK2,GRK3,ARRB1,ARRB2,RGS2,PRKACA,PRKACB,PRKACG,CAMK2A,CAMK2D,CAMK2B,CAMK2G,CALML3,CALM2,CALM3,CALM1,CALML6,CALML5,CALML4,PDE1A,PDE1B,PDE1C,PDE2A",Olfactory transduction,448

hsa04742,"SCNN1A,SCNN1B,SCNN1G,ENTPD2,TAS1R2,TAS1R3,GNAT3,GNB3,GNG13,ADCY4,ADCY6,ADCY8,PRKACA,PRKACB,PRKACG,ITPR3,TRPM5,SCN2A,SCN3A,SCN9A,CALHM1,P2RX2,P2RX3,P2RY1,P2RY4,CHRM3,TAS1R1,GRM1,GRM4,TAS2R39,TAS2R40,TAS2R41,TAS2R43,TAS2R31,TAS2R45,TAS2R46,TAS2R19,TAS2R20,TAS2R50,TAS2R60,TAS2R42,TAS2R3,TAS2R4,TAS2R16,TAS2R1,TAS2R9,TAS2R8,TAS2R7,TAS2R13,TAS2R10,TAS2R14,TAS2R5,TAS2R38,TAS2R30,PDE1A,PDE1B,PDE1C,PKD2L1,PKD1L3,KCNK5,HCN4,CACNA1A,CACNA1C,GABRA1,GABRA2,GABRA3,GABRA4,GABRA5,GABRA6,GABBR1,GABBR2,HTR1A,HTR1B,HTR1D,HTR1E,HTR1F,HTR3A,HTR3E,HTR3D,HTR3C,HTR3B,ASIC2,PLCB2",Taste transduction,83

hsa04744,"RHO,GRK7,GRK1,RCVRN,SAG,GNAT2,GNAT1,GNB1,GNGT1,RGS9,PDE6A,PDE6B,PDE6G,GUCY2D,GUCY2F,GUCA1A,GUCA1B,GUCA1C,SLC24A1,CALML3,CALM2,CALM3,CALM1,CALML6,CALML5,CALML4,CNGA1,CNGB1",Phototransduction,28

hsa04750,"KNG1,BDKRB1,BDKRB2,HTR2A,HTR2B,HTR2C,HRH1,P2RY2,GNAQ,PLA2G4E,PLA2G4A,JMJD7-PLA2G4B,PLA2G4B,PLA2G4C,PLA2G4D,PLA2G4F,PLA2G6,ALOX12,PLCB1,PLCB2,PLCB3,PLCB4,ITPR1,ITPR2,ITPR3,PRKCE,PPP1CA,PPP1CB,PPP1CC,TRPA1,TRPV1,ASIC1,ASIC2,ASIC3,ASIC4,ASIC5,CALML3,CALM2,CALM3,CALM1,CALML6,CALML5,CALML4,CAMK2A,CAMK2D,CAMK2B,CAMK2G,IL1B,IL1R1,IL1RAP,MAP2K3,MAP2K6,MAPK11,MAPK12,MAPK13,MAPK14,MAPK8,MAPK10,MAPK9,NGF,NTRK1,PLCG1,PLCG2,PIK3CA,PIK3CD,PIK3CB,PIK3R1,PIK3R2,PIK3R3,PRKCD,SRC,TRPM8,PTGER2,PTGER4,GNAS,ADCY1,ADCY2,ADCY3,ADCY4,ADCY5,ADCY6,ADCY7,ADCY8,ADCY9,PRKACA,PRKACB,PRKACG,TRPV4,F2RL1,CYP2J2,CYP4A11,CYP4A22,PRKCA,PRKCB,PRKCG,PRKCH,PRKCQ,IGF1,TRPV2,TRPV3",Inflammatory mediator regulation of TRP channels,100

hsa04810,"LPAR1,LPAR2,LPAR4,LPAR5,CXCL12,CXCR4,F2,F2R,INS,EGF,FGF1,FGF2,FGF3,FGF4,FGF17,FGF6,FGF7,FGF8,FGF9,FGF10,FGF16,FGF5,FGF18,FGF20,FGF22,FGF19,FGF21,FGF23,PDGFA,PDGFB,PDGFC,PDGFD,INSRR,EGFR,FGFR1,FGFR2,FGFR3,FGFR4,PDGFRA,PDGFRB,FN1,ITGA1,ITGA2,ITGA2B,ITGA3,ITGA4,ITGA5,ITGA6,ITGA7,ITGA8,ITGA9,ITGA10,ITGA11,ITGAV,ITGAL,ITGAM,ITGAX,ITGAD,ITGAE,ITGB1,ITGB2,ITGB3,ITGB4,ITGB5,ITGB6,ITGB7,ITGB8,KNG1,BDKRB1,BDKRB2,CHRM1,CHRM2,CHRM3,CHRM4,CHRM5,GNA12,GNA13,GNG12,FGD1,FGD3,PTK2,BCAR1,CRK,CRKL,DOCK1,SRC,SOS1,SOS2,HRAS,KRAS,NRAS,RRAS,RRAS2,MRAS,ARHGEF6,PIK3CA,PIK3CD,PIK3CB,PIK3R1,PIK3R2,PIK3R3,VAV3,VAV1,VAV2,TIAM1,ARAF,BRAF,RAF1,MOS,MAP2K1,MAP2K2,MAPK1,MAPK3,ARHGEF1,ARHGEF12,ARHGAP35,RHOA,RAC1,RAC2,RAC3,CDC42,PAK1,PAK2,PAK3,PAK4,PAK5,PAK6,BUB1B-PAK6,ARHGEF7,GIT1,ROCK1,ROCK2,MYLK,MYLK2,MYLK3,MYLK4,PPP1CA,PPP1CB,PPP1CC,PPP1R12A,PPP1R12B,PPP1R12C,MYL2,MYL5,MYL7,MYL9,MYL10,MYL12B,MYL12A,MYLPF,DIAPH1,DIAPH2,SLC9A1,PIP5K1C,PIP5K1A,PIP5K1B,PIP4K2C,PIP4K2A,PIP4K2B,PIKFYVE,LIMK1,LIMK2,DIAPH3,BAIAP2,ENAH,WAS,WASL,WASF2,CYFIP1,CYFIP2,NCKAP1,NCKAP1L,ABI2,BRK1,WASF1,ARPC5,ARPC5L,ARPC4,ARPC3,ARPC1B,ARPC1A,ARPC2,ACTB,ACTG1,PFN3,PFN1,PFN2,PFN4,PXN,EZR,RDX,MSN,TMSB4X,TMSB4Y,CFL1,CFL2,SSH1,SSH3,SSH2,VCL,IQGAP1,IQGAP2,IQGAP3,GSN,SCIN,ACTN1,ACTN4,APC,APC2,ARHGEF4,SPATA13,MYH9,MYH10,MYH14",Regulation of actin cytoskeleton,214

hsa04910,"INS,INSR,IRS1,IRS2,IRS4,PIK3R1,PIK3R2,PIK3R3,PIK3CA,PIK3CD,PIK3CB,PDPK1,AKT1,AKT2,AKT3,GSK3B,GYS2,GYS1,PPP1CA,PPP1CB,PPP1CC,PPP1R3A,PPP1R3C,PPP1R3D,PPP1R3B,PPP1R3E,PPP1R3F,PHKG1,PHKG2,PHKB,PHKA2,PHKA1,CALML3,CALM2,CALM3,CALM1,CALML6,CALML5,CALML4,PYGL,PYGM,PYGB,PDE3B,PRKACA,PRKACB,PRKACG,PRKAR1A,PRKAR2A,PRKAR2B,PRKAR1B,LIPE,PRKCZ,PRKCI,SLC2A4,FLOT2,FLOT1,SH2B2,SORBS1,CBL,CBLB,CRK,CRKL,RAPGEF1,RHOQ,EXOC7,TRIP10,SREBF1,ACACA,ACACB,FASN,PKLR,HK3,HK1,HK2,HKDC1,GCK,PRKAA1,PRKAA2,PRKAB1,PRKAB2,PRKAG1,PRKAG3,PRKAG2,FOXO1,PPARGC1A,G6PC,G6PC2,G6PC3,FBP1,FBP2,PCK1,PCK2,MTOR,RPTOR,RPS6KB1,RPS6KB2,RPS6,EIF4EBP1,EIF4E,EIF4E2,EIF4E1B,TSC1,TSC2,RHEB,BAD,SHC1,SHC2,SHC3,SHC4,GRB2,SOS1,SOS2,HRAS,KRAS,NRAS,ARAF,BRAF,RAF1,MAP2K1,MAP2K2,MAPK1,MAPK3,MKNK1,MKNK2,ELK1,SOCS1,SOCS2,SOCS3,SOCS4,PTPN1,PTPRF,MAPK8,MAPK10,MAPK9,IKBKB,INPPL1,INPP5K",Insulin signaling pathway,137

hsa04911,"SLC2A1,SLC2A2,GCK,TRPM4,ATP1A1,ATP1A2,ATP1A3,ATP1A4,ATP1B4,ATP1B1,ATP1B2,ATP1B3,FXYD2,ABCC8,KCNJ11,CACNA1C,CACNA1D,CACNA1F,CACNA1S,CAMK2A,CAMK2D,CAMK2B,CAMK2G,KCNMA1,KCNU1,KCNMB1,KCNMB2,KCNMB3,KCNMB4,KCNN1,KCNN2,KCNN3,KCNN4,GCG,GLP1R,GIP,GPR119,ADCYAP1,ADCYAP1R1,GNAS,ADCY1,ADCY2,ADCY3,ADCY4,ADCY5,ADCY6,ADCY7,ADCY8,ADCY9,PRKACA,PRKACB,PRKACG,CREB1,ATF2,ATF4,CREB3,CREB3L1,CREB3L2,CREB3L3,CREB3L4,CREB5,ATF6B,PDX1,INS,RAPGEF4,RIMS2,PCLO,RAB3A,CCK,CCKAR,CHRM3,FFAR1,GNAQ,GNA11,PLCB1,PLCB2,PLCB3,PLCB4,ITPR3,RYR2,VAMP2,STX1A,SNAP25,PRKCA,PRKCB,PRKCG",Insulin secretion,86

hsa04912,"GNRH1,GNRH2,GNRHR,GNAS,ADCY1,ADCY2,ADCY3,ADCY4,ADCY5,ADCY6,ADCY7,ADCY8,ADCY9,PRKACA,PRKACB,PRKACG,ATF4,GNAQ,GNA11,PLCB1,PLCB2,PLCB3,PLCB4,ITPR1,ITPR2,ITPR3,CALML3,CALM2,CALM3,CALM1,CALML6,CALML5,CALML4,CAMK2A,CAMK2D,CAMK2B,CAMK2G,PRKCA,PRKCB,PRKCD,CACNA1C,CACNA1D,CACNA1F,CACNA1S,MAP3K1,MAP3K2,MAP3K3,MAP3K4,MAP2K3,MAP2K6,MAPK11,MAPK12,MAPK13,MAPK14,MAPK7,PLA2G4E,PLA2G4A,JMJD7-PLA2G4B,PLA2G4B,PLA2G4C,PLA2G4D,PLA2G4F,PLD1,PLD2,SRC,CDC42,MAP2K4,MAP2K7,MAPK8,MAPK10,MAPK9,JUN,LHB,CGA,FSHB,PTK2B,MMP2,MMP14,HBEGF,EGFR,GRB2,SOS1,SOS2,HRAS,KRAS,NRAS,RAF1,MAP2K1,MAP2K2,MAPK1,MAPK3,ELK1,EGR1",GnRH signaling pathway,93

hsa04913,"CGA,LHB,LHCGR,GNAS,ADCY1,ADCY2,ADCY3,ADCY4,ADCY5,ADCY6,ADCY7,ADCY8,ADCY9,PRKACA,PRKACB,PRKACG,PLA2G4E,PLA2G4A,JMJD7-PLA2G4B,PLA2G4B,PLA2G4C,PLA2G4D,PLA2G4F,ALOX5,CYP2J2,PTGS2,INS,INSR,IGF1,IGF1R,LDLR,SCARB1,STAR,CYP11A1,CYP17A1,HSD17B1,HSD17B2,AKR1C3,HSD17B7,HSD3B1,HSD3B2,FSHB,FSHR,CYP19A1,CYP1B1,CYP1A1,BMP15,BMP6,ACOT2",Ovarian steroidogenesis,49

hsa04914,"PGR,PIK3R1,PIK3R2,PIK3R3,PIK3CA,PIK3CD,PIK3CB,MAPK8,MAPK10,MAPK9,MAPK11,MAPK12,MAPK13,MAPK14,GNAI1,GNAI3,GNAI2,ADCY1,ADCY2,ADCY3,ADCY4,ADCY5,ADCY6,ADCY7,ADCY8,ADCY9,PRKACA,PRKACB,PRKACG,AURKA,CPEB1,CPEB2,CPEB3,CPEB4,SPDYA,SPDYC,SPDYE2B,SPDYE18,SPDYE11,SPDYE16,SPDYE17,SPDYE1,SPDYE4,SPDYE3,SPDYE2,SPDYE5,SPDYE6,CDK2,CDK1,CCNA2,CCNA1,MOS,HSP90AA1,HSP90AB1,MAP2K1,MAPK1,MAPK3,RPS6KA3,RPS6KA1,RPS6KA2,RPS6KA6,PKMYT1,CCNB1,CCNB2,CCNB3,STK10,PLK1,CDC25A,CDC25B,CDC25C,INS,IGF1,IGF1R,AKT1,AKT2,AKT3,PDE3B,KRAS,ARAF,BRAF,RAF1,BUB1,MAD1L1,MAD2L1,MAD2L2,FZR1,ANAPC1,ANAPC2,CDC27,ANAPC4,ANAPC5,CDC16,ANAPC7,CDC23,ANAPC10,ANAPC11,CDC26,ANAPC13,KIF22",Progesterone-mediated oocyte maturation,99

hsa04915,"ESR1,ESR2,HSP90AA1,HSP90AB1,HSP90B1,FKBP4,FKBP5,HSPA8,HSPA1A,HSPA2,HSPA1L,HSPA1B,HSPA6,NCOA1,NCOA2,NCOA3,BCL2,EBAG9,KRT9,KRT10,KRT12,KRT13,KRT14,KRT15,KRT16,KRT17,KRT18,KRT19,KRT31,KRT32,KRT33A,KRT33B,KRT34,KRT35,KRT40,KRT39,KRT23,KRT25,KRT28,KRT24,KRT27,KRT20,KRT38,KRT37,KRT36,LOC100653049,KRT26,CTSD,TFF1,PGR,TGFA,RARA,FOS,JUN,SP1,GPER1,GNAS,SRC,MMP2,MMP9,HBEGF,ADCY1,ADCY2,ADCY3,ADCY4,ADCY5,ADCY6,ADCY7,ADCY8,ADCY9,PRKACA,PRKACB,PRKACG,CREB1,ATF2,ATF4,CREB3,CREB3L1,CREB3L2,CREB3L3,CREB3L4,CREB5,ATF6B,EGFR,SHC1,SHC2,SHC3,SHC4,GRB2,SOS1,SOS2,HRAS,KRAS,NRAS,RAF1,MAP2K1,MAP2K2,MAPK1,MAPK3,PIK3CA,PIK3CD,PIK3CB,PIK3R1,PIK3R2,PIK3R3,AKT1,AKT2,AKT3,GABBR1,GABBR2,GNAI1,GNAI3,GNAI2,GNAO1,KCNJ3,KCNJ6,KCNJ9,KCNJ5,POMC,OPRM1,PRKCD,GNAQ,PLCB1,PLCB2,PLCB3,PLCB4,ITPR1,ITPR2,ITPR3,CALML3,CALM2,CALM3,CALM1,CALML6,CALML5,CALML4,NOS3,GRM1",Estrogen signaling pathway,138

hsa04916,"POMC,ASIP,MC1R,GNAS,ADCY1,ADCY2,ADCY3,ADCY4,ADCY5,ADCY6,ADCY7,ADCY8,ADCY9,PRKACA,PRKACB,PRKACG,CREB1,CREB3,CREB3L1,CREB3L2,CREB3L3,CREB3L4,CREBBP,EP300,MITF,WNT1,WNT2,WNT2B,WNT3,WNT3A,WNT4,WNT5A,WNT5B,WNT6,WNT7A,WNT7B,WNT8A,WNT8B,WNT9A,WNT9B,WNT10B,WNT10A,WNT11,WNT16,FZD1,FZD7,FZD2,FZD3,FZD4,FZD5,FZD8,FZD6,FZD10,FZD9,GNAO1,GNAQ,DVL3,DVL2,DVL1,GSK3B,CTNNB1,TCF7,TCF7L1,TCF7L2,LEF1,KITLG,KIT,HRAS,KRAS,NRAS,RAF1,MAP2K1,MAP2K2,MAPK1,MAPK3,TYR,TYRP1,DCT,EDN1,EDNRB,GNAI1,GNAI3,GNAI2,PLCB1,PLCB2,PLCB3,PLCB4,CALML3,CALM2,CALM3,CALM1,CALML6,CALML5,CALML4,CAMK2A,CAMK2D,CAMK2B,CAMK2G,PRKCA,PRKCB,PRKCG",Melanogenesis,101

hsa04917,"PRL,PRLR,JAK2,SHC1,SHC2,SHC3,SHC4,SRC,PIK3CA,PIK3CD,PIK3CB,PIK3R1,PIK3R2,PIK3R3,AKT1,AKT2,AKT3,FOXO3,GALT,GRB2,SOS1,SOS2,HRAS,KRAS,NRAS,RAF1,MAP2K1,MAP2K2,MAPK1,MAPK3,FOS,MAPK8,MAPK10,MAPK9,MAPK11,MAPK12,MAPK13,MAPK14,CCND1,GSK3B,CISH,SOCS1,SOCS2,SOCS3,SOCS4,SOCS5,SOCS7,SOCS6,STAT1,STAT3,STAT5A,STAT5B,TNFSF11,TNFRSF11A,NFKB1,RELA,ELF5,CSN2,CGA,LHB,LHCGR,CYP17A1,ESR1,ESR2,IRF1,TH,SLC2A2,GCK,CCND2,INS",Prolactin signaling pathway,70

hsa04918,"TSHB,CGA,TSHR,GNAS,ADCY1,ADCY2,ADCY3,ADCY4,ADCY5,ADCY6,ADCY7,ADCY8,ADCY9,PRKACA,PRKACB,PRKACG,GNAQ,PLCB1,PLCB2,PLCB3,PLCB4,PRKCA,PRKCB,PRKCG,TTF1,TTF2,PAX8,CREB1,ATF2,ATF4,CREB3,CREB3L1,CREB3L2,CREB3L3,CREB3L4,CREB5,ATF6B,TG,HSPA5,HSP90B1,CANX,PDIA4,ASGR1,ASGR2,ITPR1,ITPR2,ITPR3,SLC5A5,SLC26A4,TPO,ATP1A1,ATP1A2,ATP1A3,ATP1A4,ATP1B4,ATP1B1,ATP1B2,ATP1B3,FXYD2,IYD,DUOXA2,GPX6,GPX7,GPX2,GPX3,GPX1,GPX5,GPX8,GSR,ALB,TTR,SERPINA7,LRP2,DUOX2",Thyroid hormone synthesis,74

hsa04919,"PRKACA,PRKACB,PRKACG,ITGAV,ITGB3,HRAS,KRAS,NRAS,RAF1,MAP2K1,MAP2K2,MAPK1,MAPK3,STAT1,TP53,ESR1,THRB,NCOR1,SIN3A,HDAC1,HDAC2,HDAC3,THRA,RXRA,RXRB,RXRG,KAT2B,KAT2A,NCOA1,NCOA2,NCOA3,CREBBP,EP300,MED4,MED12L,MED12,MED13L,MED13,MED14,MED16,MED17,MED24,MED27,MED30,MED1,CCND1,GATA4,RCAN1,HIF1A,MYC,MYH7,MYH6,ATP2A1,ATP2A3,ATP2A2,PLN,WNT4,CTNNB1,NOTCH1,NOTCH2,NOTCH3,NOTCH4,BMP4,PLCB1,PLCB2,PLCB3,PLCB4,PLCD1,PLCD3,PLCD4,PLCE1,PLCG1,PLCG2,PLCZ1,PRKCA,PRKCB,PRKCG,SLC9A1,ATP1A1,ATP1A2,ATP1A3,ATP1A4,ATP1B4,ATP1B1,ATP1B2,ATP1B3,FXYD2,SLC16A2,SLC16A10,SRC,PIK3CA,PIK3CD,PIK3CB,PIK3R1,PIK3R2,PIK3R3,PDPK1,AKT1,AKT2,AKT3,GSK3B,MDM2,TSC2,RHEB,MTOR,TBC1D4,PFKFB2,BAD,FOXO1,CASP9,RCAN2,SLC2A1,SLCO1C1,DIO1,DIO2,DIO3,ACTB,ACTG1,PFKP",Thyroid hormone signaling pathway,119

hsa04920,"TNF,TNFRSF1A,TRADD,TNFRSF1B,TRAF2,MTOR,MAPK8,MAPK10,MAPK9,CHUK,IKBKB,IKBKG,NFKBIA,NFKBIB,NFKBIE,NFKB1,RELA,SOCS3,IRS1,IRS2,IRS4,AKT1,AKT2,AKT3,CD36,ACSL6,ACSL4,ACSL1,ACSL5,ACSL3,ACSBG1,ACSBG2,PRKCQ,LEP,LEPR,JAK2,STAT3,POMC,PRKAA1,PRKAA2,PRKAB1,PRKAB2,PRKAG1,PRKAG3,PRKAG2,AGRP,NPY,PPARGC1A,PCK1,PCK2,G6PC,G6PC2,G6PC3,PTPN11,PPARA,RXRA,RXRB,RXRG,ADIPOQ,ADIPOR1,ADIPOR2,STK11,CAMKK2,ACACB,CPT1A,CPT1B,CPT1C,SLC2A1,SLC2A4",Adipocytokine signaling pathway,69

hsa04921,"OXT,OXTR,GNAQ,HRAS,KRAS,NRAS,RAF1,MAP2K1,MAP2K2,MAPK1,MAPK3,PLA2G4E,PLA2G4A,JMJD7-PLA2G4B,PLA2G4B,PLA2G4C,PLA2G4D,PLA2G4F,PTGS2,MAP2K5,MAPK7,JUN,FOS,MEF2C,CCND1,ELK1,RYR1,RYR2,RYR3,CD38,TRPM2,KCNJ2,KCNJ12,KCNJ4,KCNJ14,PLCB1,PLCB2,PLCB3,PLCB4,PRKCA,PRKCB,PRKCG,EEF2K,EEF2,CACNA1C,CACNA1D,CACNA1F,CACNA1S,CACNB1,CACNB2,CACNB3,CACNB4,CACNA2D1,CACNA2D2,CACNA2D3,CACNA2D4,CACNG1,CACNG2,CACNG3,CACNG4,CACNG5,CACNG6,CACNG7,CACNG8,ITPR1,ITPR2,ITPR3,CALML3,CALM2,CALM3,CALM1,CALML6,CALML5,CALML4,PPP3CA,PPP3CB,PPP3CC,PPP3R1,PPP3R2,NFATC1,NFATC2,NFATC3,NFATC4,RGS2,RCAN1,CAMKK2,PRKAA1,PRKAA2,PRKAB1,PRKAB2,PRKAG1,PRKAG3,PRKAG2,CAMK1D,CAMK1G,CAMK1,CAMK2A,CAMK2D,CAMK2B,CAMK2G,CAMK4,NOS3,GUCY1A2,GUCY1A1,GUCY1B1,NPPA,NPR1,NPR2,MYLK,MYLK2,MYLK3,MYLK4,MYL6B,MYL6,MYL9,ACTB,ACTG1,RHOA,ROCK1,ROCK2,PPP1CA,PPP1CB,PPP1CC,PPP1R12A,PPP1R12B,PPP1R12C,GNAS,ADCY1,ADCY2,ADCY3,ADCY4,ADCY5,ADCY6,ADCY7,ADCY8,ADCY9,PRKACA,PRKACB,PRKACG,GNAI1,GNAI3,GNAI2,GNAO1,PIK3CG,PIK3R5,PIK3R6,SRC,KCNJ3,KCNJ6,KCNJ9,KCNJ5,EGFR,CDKN1A",Oxytocin signaling pathway,153

hsa04922,"AKT1,AKT2,AKT3,PDE3B,GCG,GCGR,GNAS,ADCY2,PRKACA,PRKACB,PRKACG,CREB1,ATF2,ATF4,CREB3,CREB3L1,CREB3L2,CREB3L3,CREB3L4,CREB5,PPP4R3A,PPP4R3B,PPP4C,CRTC2,SIK2,CREBBP,EP300,PPARGC1A,SIK1B,SIK1,GNAQ,PLCB1,PLCB2,PLCB3,PLCB4,ITPR1,ITPR2,ITPR3,PPP3CA,PPP3CB,PPP3CC,PPP3R1,PPP3R2,CAMK2A,CAMK2D,CAMK2B,CAMK2G,FOXO1,SIRT1,PPARA,PRMT1,G6PC,G6PC2,G6PC3,PCK1,PCK2,CPT1A,CPT1B,CPT1C,GYS2,GYS1,PHKB,PHKA2,PHKA1,PHKG1,PHKG2,CALML3,CALM2,CALM3,CALM1,CALML6,CALML5,CALML4,PYGL,PYGM,PYGB,PFKFB1,PRKAA1,PRKAA2,PRKAB1,PRKAB2,PRKAG1,PRKAG3,PRKAG2,ACACA,ACACB,SLC2A1,SLC2A2,GCK,FBP1,FBP2,PFKM,PFKP,PFKL,PGAM1,PGAM2,PGAM4,PKM,PDHA2,PDHA1,PDHB,LDHAL6A,LDHAL6B,LDHA,LDHB,LDHC",Glucagon signaling pathway,106

hsa04923,"TSHB,CGA,TSHR,ADRB1,ADRB2,ADRB3,GNAS,ADCY1,ADCY2,ADCY3,ADCY4,ADCY5,ADCY6,ADCY7,ADCY8,ADCY9,PRKACA,PRKACB,PRKACG,NPPA,NPR1,PRKG1,PRKG2,PLIN1,LIPE,PNPLA2,ABHD5,MGLL,FABP4,AQP7,INS,INSR,IRS1,IRS2,IRS4,PIK3CA,PIK3CD,PIK3CB,PIK3R1,PIK3R2,PIK3R3,AKT1,AKT2,AKT3,PDE3B,PLAAT3,PTGS1,PTGS2,ADORA1,PTGER3,NPY,NPY1R,GNAI1,GNAI3,GNAI2",Regulation of lipolysis in adipocytes,55

hsa04924,"ADRB1,ADRB2,ADRB3,ADCYAP1,ADCYAP1R1,AQP1,PTGER2,PTGER4,GNAS,ADCY5,ADCY6,PRKACA,PRKACB,PRKACG,CREB1,KCNMA1,PDE1A,PDE1B,PDE1C,PDE3A,PDE3B,REN,ADORA1,GNAI1,GNAI3,GNAI2,ORAI1,CLCA1,CLCA2,CLCA4,CACNA1C,CACNA1D,CACNA1F,CACNA1S,KCNJ2,EDN1,EDN2,EDN3,EDNRA,AGTR1,GNAQ,PLCB1,PLCB2,PLCB3,PLCB4,ITPR1,ITPR2,ITPR3,CALML3,CALM2,CALM3,CALM1,CALML6,CALML5,CALML4,PPP3CA,PPP3CB,PPP3CC,PPP3R1,PPP3R2,GUCY1A2,GUCY1A1,GUCY1B1,NPPA,NPR1,PRKG2,CTSB,AGT,ACE",Renin secretion,69

hsa04925,"AGT,AGTR1,GNAQ,GNA11,PLCB1,PLCB2,PLCB3,PLCB4,ITPR1,ITPR2,ITPR3,PRKCA,PRKCB,PRKCG,PRKCE,PRKD1,PRKD3,PRKD2,CREB1,ATF2,ATF4,CREB3,CREB3L1,CREB3L2,CREB3L3,CREB3L4,CREB5,ATF6B,ATF1,DAGLA,DAGLB,KCNK3,KCNK9,KCNJ5,ATP1A1,ATP1A2,ATP1A3,ATP1A4,ATP1B4,ATP1B1,ATP1B2,ATP1B3,CACNA1G,CACNA1H,CACNA1I,CACNA1C,CACNA1D,CACNA1F,CACNA1S,ORAI1,CALML3,CALM2,CALM3,CALM1,CALML6,CALML5,CALML4,CAMK1D,CAMK1G,CAMK1,CAMK2A,CAMK2D,CAMK2B,CAMK2G,CAMK4,ATP2B1,ATP2B3,ATP2B4,ATP2B2,LIPE,LDLR,SCARB1,POMC,MC2R,GNAS,ADCY1,ADCY2,ADCY3,ADCY4,ADCY5,ADCY6,ADCY7,ADCY8,ADCY9,PRKACA,PRKACB,PRKACG,CYP11B2,STAR,NR4A2,NR4A1,CYP11A1,HSD3B1,HSD3B2,CYP21A2,NPPA,NPR1,PDE2A",Aldosterone synthesis and secretion,98

hsa04926,"RLN1,RLN2,RXFP1,GNAO1,GNAI1,GNAI3,GNAI2,GNAS,GNB1,GNB2,GNB3,GNB4,GNB5,GNG2,GNG3,GNG4,GNG5,GNG7,GNG8,GNG10,GNG11,GNG12,GNG13,GNGT1,GNGT2,ADCY5,PRKACA,PRKACB,PRKACG,CREB1,ATF2,ATF4,CREB3,CREB3L1,CREB3L2,CREB3L3,CREB3L4,CREB5,ATF6B,VEGFA,VEGFB,VEGFC,VEGFD,NFKBIA,NFKB1,RELA,NOS2,PIK3CA,PIK3CD,PIK3CB,PIK3R1,PIK3R2,PIK3R3,PRKCZ,RAF1,MAP2K1,MAP2K2,MAPK1,MAPK3,MMP2,MMP9,AKT1,AKT2,AKT3,NOS3,NOS1,EDN1,EDNRB,MMP1,MMP13,TGFB1,TGFBR1,TGFBR2,SMAD2,SMAD3,ACTA2,COL1A1,COL1A2,COL3A1,COL4A2,COL4A4,COL4A6,COL4A1,COL4A5,COL4A3,INSL3,RXFP2,RLN3,RXFP3,ADCY1,ADCY2,ADCY3,ADCY4,ADCY6,ADCY7,ADCY8,ADCY9,PLCB1,PLCB2,PLCB3,PLCB4,PRKCA,MAP2K4,MAP2K7,MAPK11,MAPK12,MAPK13,MAPK14,MAPK8,MAPK10,MAPK9,JUN,FOS,HRAS,KRAS,NRAS,SRC,SHC1,SHC2,SHC3,SHC4,SOS1,SOS2,ARRB1,ARRB2,EGFR,GRB2,INSL5,RXFP4,GNA15",Relaxin signaling pathway,130

hsa04927,"POMC,MC2R,MRAP,GNAS,ADCY1,ADCY2,ADCY3,ADCY4,ADCY5,ADCY6,ADCY7,ADCY8,ADCY9,PDE8B,PDE8A,PRKACA,PRKACB,PRKACG,NR0B1,NR5A1,NR4A1,SP1,PBX1,CREB1,ATF2,ATF4,CREB3,CREB3L1,CREB3L2,CREB3L3,CREB3L4,CREB5,ATF6B,CYP11B1,CYP17A1,STAR,NCEH1,AGT,AGTR1,GNAQ,GNA11,PLCB1,PLCB2,PLCB3,PLCB4,ITPR1,ITPR2,ITPR3,KCNK2,CACNA1G,CACNA1H,CACNA1I,CACNA1C,CACNA1D,CACNA1F,CACNA1S,ORAI1,KCNA4,KCNK3,LDLR,SCARB1,CYP11A1,HSD3B1,HSD3B2,CYP21A2",Cortisol synthesis and secretion,65

hsa04928,"CASR,GNAI1,GNAI3,GNAI2,ADCY1,ADCY2,ADCY3,ADCY4,ADCY5,ADCY6,ADCY7,ADCY8,ADCY9,GNAQ,GNA11,PLCB1,PLCB2,PLCB3,PLCB4,ITPR1,ITPR2,ITPR3,GCM1,GCM2,MAFB,GATA3,SP1,VDR,RXRA,RXRB,RXRG,FGF23,FGFR1,KL,MAPK1,MAPK3,EGR1,PTH,PTHLH,PTH1R,ARRB1,ARRB2,ARAF,BRAF,RAF1,MAP2K1,PRKCA,PRKCB,PRKCG,TRPV5,SLC34A2,SLC34A1,SLC34A3,SLC9A3R1,GNAS,PDE4A,PDE4B,PDE4C,PDE4D,PRKACA,PRKACB,PRKACG,CYP27B1,CYP24A1,MEF2A,MEF2C,MEF2D,SOST,NR4A2,NACA,JUND,BGLAP,LRP5,LRP6,CREB1,ATF2,CREB3,CREB3L1,CREB3L2,CREB3L3,CREB3L4,ATF4,CREB5,ATF6B,TNFSF11,BCL2,CDKN1A,FOS,RUNX2,MMP13,GNA12,GNA13,ARHGEF1,ARHGEF11,AKAP13,RHOA,PLD1,PLD2,MMP14,MMP15,MMP16,MMP17,MMP24,MMP25,HBEGF,EGFR","Parathyroid hormone synthesis, secretion and action",106

hsa04930,"INS,INSR,IRS1,IRS2,IRS4,PIK3R1,PIK3R2,PIK3R3,PIK3CA,PIK3CD,PIK3CB,SLC2A4,ADIPOQ,MAPK1,MAPK3,MTOR,PRKCZ,SOCS1,SOCS2,SOCS3,SOCS4,IKBKB,MAPK8,MAPK10,MAPK9,TNF,PRKCD,PRKCE,PDX1,MAFA,SLC2A2,HK3,HK1,HK2,HKDC1,GCK,PKM,PKLR,KCNJ11,ABCC8,CACNA1C,CACNA1D,CACNA1A,CACNA1B,CACNA1E,CACNA1G",Type II diabetes mellitus,46

hsa04931,"INS,RPS6KA3,RPS6KA1,RPS6KA2,RPS6KA6,PPP1CA,PPP1CB,PPP1CC,PPP1R3A,PPP1R3C,PPP1R3D,PPP1R3B,PPP1R3E,INSR,IRS1,PTPN1,PTPN11,PTPRF,PIK3CA,PIK3CD,PIK3CB,PIK3R1,PIK3R2,PIK3R3,MTOR,RPS6KB1,RPS6KB2,PRKCB,IL6,STAT3,SOCS3,GSK3B,GYS2,GYS1,TNF,TNFRSF1A,MAPK8,MAPK10,MAPK9,IKBKB,NFKBIA,NFKB1,RELA,NOS3,PRKCZ,TBC1D4,SLC2A4,PRKAA1,PRKAA2,PRKAB1,PRKAB2,PRKAG1,PRKAG3,PRKAG2,ACACB,CPT1B,AGT,SLC27A1,SLC27A4,SLC27A2,SLC27A3,SLC27A5,SLC27A6,CD36,PRKCQ,PRKCD,PTPA,SREBF1,IRS2,TRIB3,PYGL,PYGM,PYGB,FOXO1,PCK1,PCK2,G6PC,G6PC2,G6PC3,SLC2A2,NR1H3,NR1H2,PPARGC1B,MLX,MLXIP,MLXIPL,CPT1A,PRKCE,PPARGC1A,PPARA,PDPK1,AKT1,AKT2,AKT3,PTEN,OGA,OGT,SLC2A1,GFPT2,GFPT1,CRTC2,CREB1,CREB3,CREB3L1,CREB3L2,CREB3L3,CREB3L4,CREB5",Insulin resistance,108

hsa04932,"IL6,IL6R,SOCS3,TNF,TNFRSF1A,NFKB1,RELA,INS,INSR,IRS1,IRS2,PIK3CA,PIK3CD,PIK3CB,PIK3R1,PIK3R2,PIK3R3,AKT1,AKT2,AKT3,GSK3A,GSK3B,NR1H3,RXRA,SREBF1,MLX,MLXIP,MLXIPL,PKLR,LEP,LEPR,ADIPOQ,ADIPOR1,ADIPOR2,PRKAA1,PRKAA2,PRKAB1,PRKAB2,PRKAG1,PRKAG3,PRKAG2,PPARA,CDC42,RAC1,MAP3K11,MAPK8,MAPK10,MAPK9,ITCH,ERN1,TRAF2,MAP3K5,JUN,IL1A,IL1B,IKBKB,XBP1,CEBPA,CYP2E1,FASLG,CXCL8,TGFB1,EIF2AK3,EIF2S1,ATF4,DDIT3,BCL2L11,BAX,FAS,CASP8,BID,CYCS,CASP3,CASP7,NDUFV1,NDUFV2,NDUFV3,NDUFA1,NDUFA2,NDUFA3,NDUFA4,NDUFA4L2,NDUFA5,NDUFA6,NDUFA7,NDUFA8,NDUFA9,NDUFA10,NDUFAB1,NDUFA11,NDUFA12,NDUFA13,NDUFB1,NDUFB2,NDUFB3,NDUFB4,NDUFB5,NDUFB6,NDUFB7,NDUFB8,NDUFB9,NDUFB10,NDUFB11,NDUFS1,NDUFS2,NDUFS3,NDUFS4,NDUFS5,NDUFS6,NDUFS7,NDUFS8,NDUFC1,NDUFC2,NDUFC2-KCTD14,SDHA,SDHB,SDHC,SDHD,UQCRFS1,CYTB,CYC1,UQCRC1,UQCRC2,UQCRH,UQCRHL,UQCRB,UQCRQ,UQCR10,UQCR11,COX3,COX1,COX2,COX4I2,COX4I1,COX5A,COX5B,COX6A1,COX6A2,COX6B1,COX6B2,COX6C,COX7A1,COX7A2,COX7A2L,COX7B,COX7B2,COX7C,COX8C,COX8A",Non-alcoholic fatty liver disease (NAFLD),149

hsa04933,"TGFB2,TGFB3,TGFB1,TGFBR1,TGFBR2,SMAD2,SMAD3,SMAD4,CDKN1B,FN1,COL1A1,COL1A2,COL3A1,COL4A2,COL4A4,COL4A6,COL4A1,COL4A5,COL4A3,AGT,AGTR1,AGER,NOX1,CYBB,NOX4,PLCD1,PLCD3,PLCD4,PLCB1,PLCB2,PLCB3,PLCB4,PLCG1,PLCG2,PLCE1,PRKCA,PRKCB,PRKCD,PRKCE,PRKCZ,MAPK1,MAPK3,JUN,VEGFA,VEGFB,VEGFC,VEGFD,CCL2,SERPINE1,SELE,VCAM1,ICAM1,MMP2,IL1A,IL1B,IL6,CXCL8,TNF,F3,EDN1,THBD,MAPK11,MAPK12,MAPK13,MAPK14,RELA,NFKB1,MAPK8,MAPK10,MAPK9,DIAPH1,RAC1,CDC42,HRAS,KRAS,NRAS,PIK3CA,PIK3CD,PIK3CB,PIK3R1,PIK3R2,PIK3R3,AKT1,AKT2,AKT3,NOS3,FOXO1,BCL2,BAX,CASP3,JAK2,STAT3,PIM1,NFATC1,STAT1,STAT5A,STAT5B,CCND1,CDK4,EGR1",AGE-RAGE signaling pathway in diabetic complications,100

hsa04934,"CRH,CRHR1,CRHR2,GNAS,ADCY1,ADCY2,ADCY3,ADCY4,ADCY5,ADCY6,ADCY7,ADCY8,ADCY9,PRKACA,PRKACB,PRKACG,CREB1,ATF2,ATF4,CREB3,CREB3L1,CREB3L2,CREB3L3,CREB3L4,CREB5,ATF6B,MEN1,ASH2L,RBBP5,WDR5,WDR5B,KMT2A,KMT2D,CDKN2C,CDKN2B,CDKN2A,CDK4,CDK6,CCND1,RB1,E2F1,E2F2,E2F3,RASD1,GNAI1,GNAI3,GNAI2,AHR,AIPL1,AIP,ARNT,CDKN1B,CDK2,CCNE1,CCNE2,CDKN1A,CACNA1C,CACNA1D,CACNA1F,CACNA1S,CAMK2A,CAMK2D,CAMK2B,CAMK2G,RAP1A,RAP1B,BRAF,MAP2K1,MAP2K2,MAPK1,MAPK3,NR4A1,EGFR,USP8,POMC,MC2R,MRAP,PDE8B,PDE8A,PDE11A,NR5A1,SP1,PBX1,CYP11B1,CYP17A1,STAR,NCEH1,ARMC5,AGT,AGTR1,GNAQ,GNA11,PLCB1,PLCB2,PLCB3,PLCB4,ITPR1,ITPR2,ITPR3,KCNK2,CACNA1G,CACNA1H,CACNA1I,ORAI1,KCNA4,KCNK3,LDLR,SCARB1,CYP11A1,HSD3B1,HSD3B2,CYP21A2,FH,WNT1,WNT2,WNT2B,WNT3,WNT3A,WNT4,WNT5A,WNT5B,WNT6,WNT7A,WNT7B,WNT8A,WNT8B,WNT9A,WNT9B,WNT10B,WNT10A,WNT11,WNT16,FZD1,FZD7,FZD2,FZD3,FZD4,FZD5,FZD8,FZD6,FZD10,FZD9,DVL3,DVL2,DVL1,GSK3B,AXIN1,AXIN2,CTNNB1,APC,APC2,TCF7,TCF7L1,TCF7L2,LEF1",Cushing syndrome,155

hsa04940,"INS,GAD1,GAD2,PTPRN,PTPRN2,CPE,HSPD1,ICA1,HLA-DMA,HLA-DMB,HLA-DOA,HLA-DOB,HLA-DPA1,HLA-DPB1,HLA-DQA1,HLA-DQA2,HLA-DQB1,HLA-DRA,HLA-DRB1,HLA-DRB3,HLA-DRB4,HLA-DRB5,CD80,CD86,CD28,IL12A,IL12B,IL2,IFNG,HLA-A,HLA-B,HLA-C,HLA-F,HLA-G,HLA-E,FASLG,FAS,PRF1,GZMB,LTA,TNF,IL1A,IL1B",Type I diabetes mellitus,43

hsa04950,"HHEX,MNX1,ONECUT1,PDX1,NR5A2,NEUROG3,NKX2-2,NKX6-1,PAX6,PAX4,NEUROD1,RFX6,HES1,HNF1B,FOXA2,MAFA,HNF4A,HNF1A,HNF4G,FOXA3,PKLR,SLC2A2,INS,IAPP,GCK,BHLHA15",Maturity onset diabetes of the young,26

hsa04960,"HSD11B2,NR3C2,SCNN1A,SCNN1B,SCNN1G,SGK1,KCNJ1,SLC9A3R2,NEDD4L,SFN,KRAS,ATP1A1,ATP1A2,ATP1A3,ATP1A4,ATP1B4,ATP1B1,ATP1B2,ATP1B3,FXYD2,FXYD4,INS,IGF1,INSR,IRS1,PIK3R1,PIK3R2,PIK3R3,PIK3CA,PIK3CD,PIK3CB,PDPK1,PRKCA,PRKCB,PRKCG,MAPK1,MAPK3",Aldosterone-regulated sodium reabsorption,37

hsa04961,"PTH,PTH1R,GNAS,ADCY6,ADCY9,PRKACA,PRKACB,PRKACG,VDR,ESR1,TRPV5,KL,DNM1,DNM3,DNM2,AP2A2,AP2A1,AP2B1,AP2M1,AP2S1,CLTA,CLTB,CLTC,CLTCL1,RAB11A,CALB1,ATP1A1,ATP1A2,ATP1A3,ATP1A4,ATP1B4,ATP1B1,ATP1B2,ATP1B3,FXYD2,SLC8A1,SLC8A2,SLC8A3,KLK2,KLK1,BDKRB2,GNAQ,PLCB1,PLCB2,PLCB3,PLCB4,PRKCA,PRKCB,PRKCG,ATP2B1",Endocrine and other factor-regulated calcium reabsorption,50

hsa04962,"AVP,AVPR2,GNAS,ADCY6,ADCY3,ADCY9,PRKACA,PRKACB,PRKACG,ARHGDIB,ARHGDIA,ARHGDIG,CREB1,CREB3,CREB3L1,CREB3L2,CREB3L3,CREB3L4,CREB5,AQP2,RAB11A,RAB11B,DYNC1H1,DYNC2H1,DYNC1I1,DYNC1I2,DYNC1LI2,DYNC1LI1,DYNC2LI1,DYNLL1,DYNLL2,DCTN1,DCTN2,DCTN4,DCTN5,DCTN6,VAMP2,NSF,STX4,RAB5A,RAB5B,RAB5C,AQP4,AQP3",Vasopressin-regulated water reabsorption,44

hsa04964,"SLC9A3,CA4,AQP1,CA2,SLC4A4,ATP1A1,ATP1A2,ATP1A3,ATP1A4,ATP1B4,ATP1B1,ATP1B2,ATP1B3,FXYD2,SLC38A3,GLS2,GLS,GLUD2,GLUD1,SLC25A10,MDH1,PCK1,PCK2",Proximal tubule bicarbonate reclamation,23

hsa04966,"CA2,ATP6V1A,ATP6V1B1,ATP6V1B2,ATP6V1G1,ATP6V1G3,ATP6V1G2,ATP6V1C2,ATP6V1C1,ATP6V1D,ATP6V1E2,ATP6V1E1,ATP6V1F,ATP6V0E1,ATP6V0E2,TCIRG1,ATP6V0A2,ATP6V0A4,ATP6V0A1,ATP6V0C,ATP6V0D1,ATP6V0D2,ATP4A,ATP4B,SLC4A1,SLC12A7,CLCNKB",Collecting duct acid secretion,27

hsa04970,"ADRB1,ADRB2,ADRB3,GNAS,ADCY1,ADCY2,ADCY3,ADCY4,ADCY5,ADCY6,ADCY7,ADCY8,ADCY9,PRKACA,PRKACB,PRKACG,VAMP2,ADRA1A,ADRA1B,ADRA1D,CHRM3,GNAQ,PLCB1,PLCB2,PLCB3,PLCB4,PRKCA,PRKCB,PRKCG,ITPR1,ITPR2,ITPR3,RYR3,CALML3,CALM2,CALM3,CALM1,CALML6,CALML5,CALML4,NOS1,GUCY1A2,GUCY1A1,GUCY1B1,PRKG1,PRKG2,CD38,BST1,BEST2,AQP5,ATP1A1,ATP1A2,ATP1A3,ATP1A4,ATP1B4,ATP1B1,ATP1B2,ATP1B3,FXYD2,ATP2B1,ATP2B3,ATP2B4,ATP2B2,SLC12A2,KCNN4,KCNMA1,SLC4A2,SLC9A1,TRPV6,MUC5B,MUC7,PRH2,PRH1,PRB1,PRB2,DMBT1,CST1,CST2,CST3,CST4,CST5,HTN1,HTN3,STATH,LYZ,LPO,CAMP,AMY1A,AMY1B,AMY1C",Salivary secretion,90

hsa04971,"CHRM3,GAST,CCKBR,GNAQ,PLCB1,PLCB2,PLCB3,PLCB4,PRKCA,PRKCB,PRKCG,EZR,ITPR1,ITPR2,ITPR3,CALML3,CALM2,CALM3,CALM1,CALML6,CALML5,CALML4,CAMK2A,CAMK2D,CAMK2B,CAMK2G,MYLK,MYLK2,MYLK3,MYLK4,HRH2,GNAS,ADCY1,ADCY2,ADCY3,ADCY4,ADCY5,ADCY6,ADCY7,ADCY8,ADCY9,SST,SSTR2,GNAI1,GNAI3,GNAI2,PRKACA,PRKACB,PRKACG,ATP4A,ATP4B,KCNE2,KCNQ1,KCNJ1,KCNJ2,KCNJ10,KCNJ15,KCNJ16,CFTR,KCNK2,KCNK10,ATP1A1,ATP1A2,ATP1A3,ATP1A4,ATP1B4,ATP1B1,ATP1B2,ATP1B3,SLC9A1,SLC9A4,CA2,SLC26A7,SLC4A2,ACTB",Gastric acid secretion,75

hsa04972,"CHRM3,CCK,CCKAR,GNAQ,PLCB1,PLCB2,PLCB3,PLCB4,PRKCA,PRKCB,PRKCG,RAB3D,RAB8A,RAB11A,RAB27B,RAP1A,RAP1B,RHOA,RAC1,PRSS3,PRSS2,PRSS1,CTRL,CELA2A,CELA2B,CELA3A,CELA3B,CPA1,CPA2,CPA3,CPB1,CPB2,PNLIP,PNLIPRP1,PNLIPRP2,CEL,PLA2G10,PLA2G2D,PLA2G2E,PLA2G3,PLA2G2F,PLA2G12A,PLA2G12B,PLA2G1B,PLA2G5,PLA2G2A,PLA2G2C,ITPR1,ITPR2,ITPR3,RYR2,CD38,BST1,TPCN2,ATP2A1,ATP2A3,ATP2A2,CLCA1,CLCA2,CLCA4,TRPC1,SLC12A2,ATP1A1,ATP1A2,ATP1A3,ATP1A4,ATP1B4,ATP1B1,ATP1B2,ATP1B3,FXYD2,KCNQ1,KCNMA1,ATP2B1,ATP2B3,ATP2B4,ATP2B2,SLC9A1,SLC4A2,SCT,SCTR,GNAS,ADCY1,ADCY2,ADCY3,ADCY4,ADCY5,ADCY6,ADCY7,ADCY8,ADCY9,CFTR,SLC26A3,SLC4A4,CA2,CTRB1,AMY2A,AMY2B",Pancreatic secretion,98

hsa04973,"AMY1C,AMY2A,AMY1A,AMY2B,AMY1B,LCT,MGAM,MGAM2,SI,SLC5A1,ATP1A1,ATP1A2,ATP1A3,ATP1A4,ATP1B4,ATP1B1,ATP1B2,ATP1B3,FXYD2,SLC2A2,HK3,HK1,HK2,HKDC1,SLC37A4,G6PC,G6PC2,G6PC3,SLC2A5,PIK3R1,PIK3R2,PIK3R3,PIK3CA,PIK3CD,PIK3CB,AKT1,AKT2,AKT3,CACNA1D,PRKCB,TAS1R2,TAS1R3,GNAT3,PLCB2",Carbohydrate digestion and absorption,44

hsa04974,"PGA5,PGA4,PGA3,PRSS3,PRSS2,PRSS1,CTRL,CELA2A,CELA2B,CELA3A,CELA3B,CPA1,CPA2,CPA3,CPB1,CPB2,SLC9A3,SLC15A1,ATP1A1,ATP1A2,ATP1A3,ATP1A4,ATP1B4,ATP1B1,ATP1B2,ATP1B3,FXYD2,KCNN4,KCNK5,KCNE3,KCNQ1,KCNJ13,SLC38A2,MME,MEP1A,MEP1B,ACE2,PRCP,DPP4,XPNPEP2,SLC1A1,SLC1A5,SLC8A1,SLC8A2,SLC8A3,SLC6A19,SLC3A2,SLC7A8,SLC16A10,SLC3A1,SLC7A9,SLC7A7,ELN,COL1A1,COL1A2,COL2A1,COL3A1,COL4A2,COL4A4,COL4A6,COL4A1,COL4A5,COL4A3,COL5A1,COL5A2,COL5A3,COL11A1,COL24A1,COL27A1,COL11A2,COL6A1,COL6A2,COL6A3,COL6A6,COL6A5,COL7A1,COL9A1,COL9A2,COL9A3,COL10A1,COL12A1,COL13A1,COL14A1,COL15A1,COL17A1,COL18A1,COL21A1,COL22A1,CTRB1,SLC36A1",Protein digestion and absorption,90

hsa04975,"LIPF,PNLIP,PNLIPRP1,PNLIPRP2,CEL,PLA2G10,PLA2G2D,PLA2G2E,PLA2G3,PLA2G2F,PLA2G12A,PLA2G12B,PLA2G1B,PLA2G5,PLA2G2A,PLA2G2C,CLPS,CD36,GOT2,FABP2,FABP1,AGPAT1,AGPAT2,PLPP1,PLPP3,PLPP2,MOGAT2,MOGAT3,DGAT1,DGAT2,SCARB1,NPC1L1,ABCG5,ABCG8,APOA4,APOB,MTTP,APOA1,ABCA1,SLC27A4,ACAT2",Fat digestion and absorption,41

hsa04976,"EPHX1,SLCO1A2,SLCO1B3,SLCO1B1,SLCO1B7,SLC10A1,SLC22A7,SLC22A8,SLC22A1,HMGCR,SCARB1,LDLR,NCEH1,CYP7A1,NR1H4,RXRA,NR0B2,SULT2A1,SLC27A5,BAAT,CYP3A4,AQP9,AQP8,ABCG5,ABCG8,ABCB11,ABCC2,ABCG2,CA2,SLC4A2,ABCB1,ABCB4,SLC9A1,SLC4A5,ABCC3,ABCC4,SLC51A,SLC51B,KCNN2,ATP1A1,ATP1A2,ATP1A3,ATP1A4,ATP1B4,ATP1B1,ATP1B2,ATP1B3,FXYD2,SCT,SCTR,GNAS,ADCY1,ADCY2,ADCY3,ADCY4,ADCY5,ADCY6,ADCY7,ADCY8,ADCY9,PRKACA,PRKACB,PRKACG,CFTR,SLC4A4,AQP4,AQP1,SLC10A2,SLC5A1,SLC9A3,SLC2A1,UGT2B4",Bile secretion,72

hsa04977,"FOLH1,BTD,CBLIF,CUBN,LMBRD1,MMACHC,ABCC1,TCN2,SLC19A3,SLC19A2,SLC52A3,SLC5A6,SLC19A1,SCARB1,PNLIP,PLB1,RBP2,AWAT2,LRAT,APOA4,APOB,APOA1,SLC46A1,SLC23A1",Vitamin digestion and absorption,24

hsa04978,"VDR,TRPM6,TRPM7,TRPV6,SLC8A1,SLC8A2,SLC8A3,S100G,SLC26A3,SLC26A6,SLC26A9,CLCN2,SLC9A3,SLC5A1,SLC6A19,ATP1A1,ATP1A2,ATP1A3,ATP1A4,ATP1B4,ATP1B1,ATP1B2,ATP1B3,FXYD2,HMOX1,HMOX2,CYBRD1,SLC11A2,FTH1,FTL,SLC40A1,HEPH,TF,SLC39A4,SLC30A1,SLC31A1,STEAP1,STEAP2,MT1B,MT1E,MT1F,MT1G,MT1H,MT1M,MT1X,MT2A,MT1A,MT1HL1,ATOX1,SLC34A2,SLC46A1,ATP2B1,ATP7A",Mineral absorption,53

hsa04979,"ABCA1,APOA1,LPL,LCAT,LIPG,PLTP,CETP,LIPC,LPA,CD36,LDLR,NCEH1,SOAT2,SOAT1,APOC1,APOC2,APOH,ANGPTL3,ANGPTL4,ANGPTL8,SCARB1,SORT1,APOB,PCSK9,LDLRAP1,MYLIP,APOE,LRP1,LRP2,LRPAP1,LIPA,STARD3,VDAC1,VDAC2,VDAC3,TSPO,CYP27A1,VAPA,VAPB,NPC1,OSBPL5,NPC2,STAR,CYP7A1,ABCB11,ABCG5,ABCG8,APOA2,APOC3,APOA4",Cholesterol metabolism,50

hsa05010,"ADAM10,ADAM17,APP,NAE1,APBB1,GAPDH,BACE1,BACE2,RTN3,RTN4,PSENEN,PSEN1,PSEN2,NCSTN,APH1A,APH1B,IDE,MME,NDUFV1,NDUFV2,NDUFV3,NDUFA1,NDUFA2,NDUFA3,NDUFA4,NDUFA4L2,NDUFA5,NDUFA6,NDUFA7,NDUFA8,NDUFA9,NDUFA10,NDUFAB1,NDUFA11,NDUFA12,NDUFA13,NDUFB1,NDUFB2,NDUFB3,NDUFB4,NDUFB5,NDUFB6,NDUFB7,NDUFB8,NDUFB9,NDUFB10,NDUFB11,NDUFS1,NDUFS2,NDUFS3,NDUFS4,NDUFS5,NDUFS6,NDUFS7,NDUFS8,NDUFC1,NDUFC2,NDUFC2-KCTD14,SDHA,SDHB,SDHC,SDHD,UQCRFS1,CYTB,CYC1,UQCRC1,UQCRC2,UQCRH,UQCRHL,UQCRB,UQCRQ,UQCR10,UQCR11,COX3,COX1,COX2,COX4I2,COX4I1,COX5A,COX5B,COX6A1,COX6A2,COX6B1,COX6B2,COX6C,COX7A1,COX7A2,COX7A2L,COX7B,COX7B2,COX7C,COX8C,COX8A,ATP5F1A,ATP5F1B,ATP5F1C,ATP5F1D,ATP5F1E,ATP6,ATP5PB,ATP5MC1,ATP5MC2,ATP5MC3,ATP5PD,ATP5PO,ATP5PF,ATP8,HSD17B10,LPL,APOE,LRP1,FAS,TNFRSF1A,FADD,CASP8,BID,CALML3,CALM2,CALM3,CALM1,CALML6,CALML5,CALML4,PPP3CA,PPP3CB,PPP3CC,PPP3R1,PPP3R2,BAD,CYCS,APAF1,CASP9,CASP3,GNAQ,PLCB1,PLCB2,PLCB3,PLCB4,GRIN1,GRIN2A,GRIN2B,GRIN2C,GRIN2D,CACNA1C,CACNA1D,CACNA1F,CACNA1S,MAPK1,MAPK3,RYR3,ITPR1,ITPR2,ITPR3,ATP2A1,ATP2A3,ATP2A2,ATF6,ERN1,EIF2AK3,CASP12,NOS1,CAPN1,CAPN2,CDK5R1,CDK5,MAPT,GSK3B,CASP7,SNCA,TNF,IL1B",Alzheimer disease,171

hsa05012,"ADORA2A,GNAL,DRD2,GNAI1,GNAI3,GNAI2,ADCY5,PRKACA,PRKACB,PRKACG,DRD1,UBA1,UBA7,UBB,UBE2L3,UBE2L6,UBE2J2,UBE2J1,UBE2G2,UBE2G1,PRKN,SNCA,GPR37,SEPTIN5,SNCAIP,UCHL1,TH,SLC6A3,SLC18A1,SLC18A2,ND1,ND2,ND3,ND4,ND4L,ND5,ND6,NDUFV1,NDUFV2,NDUFV3,NDUFA1,NDUFA2,NDUFA3,NDUFA4,NDUFA4L2,NDUFA5,NDUFA6,NDUFA7,NDUFA8,NDUFA9,NDUFA10,NDUFAB1,NDUFA11,NDUFA12,NDUFA13,NDUFB1,NDUFB2,NDUFB3,NDUFB4,NDUFB5,NDUFB6,NDUFB7,NDUFB8,NDUFB9,NDUFB10,NDUFB11,NDUFS1,NDUFS2,NDUFS3,NDUFS4,NDUFS5,NDUFS6,NDUFS7,NDUFS8,NDUFC1,NDUFC2,NDUFC2-KCTD14,SDHA,SDHB,SDHC,SDHD,UQCRFS1,CYTB,CYC1,UQCRC1,UQCRC2,UQCRH,UQCRHL,UQCRB,UQCRQ,UQCR10,UQCR11,COX3,COX1,COX2,COX4I2,COX4I1,COX5A,COX5B,COX6A1,COX6A2,COX6B1,COX6B2,COX6C,COX7A1,COX7A2,COX7A2L,COX7B,COX7B2,COX7C,COX8C,COX8A,ATP5F1A,ATP5F1B,ATP5F1C,ATP5F1D,ATP5F1E,ATP6,ATP5PB,ATP5MC1,ATP5MC2,ATP5MC3,ATP5PD,ATP5PO,ATP5PF,ATP8,LRRK2,PINK1,PARK7,HTRA2,VDAC1,VDAC2,VDAC3,SLC25A4,SLC25A5,SLC25A6,SLC25A31,PPIF,CYCS,APAF1,CASP9,CASP3",Parkinson disease,142

hsa05014,"TP53,PPP3CA,PPP3CB,PPP3CC,PPP3R1,PPP3R2,CASP1,BID,BCL2,BCL2L1,BAX,BAD,APAF1,CYCS,CASP9,CASP3,SOD1,TOMM40,TOMM40L,DERL1,CASP12,MAP3K5,TNF,TNFRSF1A,TNFRSF1B,DAXX,MAP2K3,MAP2K6,MAPK11,MAPK12,MAPK13,MAPK14,NOS1,CCS,CAT,PRPH,NEFL,NEFM,NEFH,ALS2,RAB5A,RAC1,SLC1A2,GRIA1,GRIA2,GRIN1,GRIN2A,GRIN2B,GRIN2C,GRIN2D,GPX1",Amyotrophic lateral sclerosis (ALS),51

hsa05016,"CLTA,CLTB,CLTC,CLTCL1,HIP1,AP2A2,AP2A1,AP2B1,AP2M1,AP2S1,HTT,IFT57,CASP8,CASP3,GRM5,GNAQ,PLCB1,PLCB2,PLCB3,PLCB4,GRIN1,GRIN2B,ITPR1,DLG4,TGM2,REST,SIN3A,RCOR1,HDAC1,HDAC2,POLR2A,POLR2B,POLR2C,POLR2D,POLR2E,POLR2F,POLR2G,POLR2H,POLR2I,POLR2L,POLR2J,POLR2J3,POLR2J2,POLR2K,BDNF,DCTN1,DCTN2,DCTN4,HAP1,DNAH1,DNAH3,DNAH2,DNAH7,DNAH5,DNAH6,DNAH9,DNAH11,DNAH12,DNAH14,DNAH8,DNAH10,DNAH17,DNALI1,DNAI1,DNAI2,DNAL1,DNAL4,CREBBP,EP300,TAF4B,TAF4,TBPL2,TBPL1,TBP,SP1,CREB1,CREB3,CREB3L1,CREB3L2,CREB3L3,CREB3L4,CREB5,PPARGC1A,PPARG,TFAM,NRF1,SOD1,SOD2,UCP1,TP53,BAX,BBC3,NDUFV1,NDUFV2,NDUFV3,NDUFA1,NDUFA2,NDUFA3,NDUFA4,NDUFA4L2,NDUFA5,NDUFA6,NDUFA7,NDUFA8,NDUFA9,NDUFA10,NDUFAB1,NDUFA11,NDUFA12,NDUFA13,NDUFB1,NDUFB2,NDUFB3,NDUFB4,NDUFB5,NDUFB6,NDUFB7,NDUFB8,NDUFB9,NDUFB10,NDUFB11,NDUFS1,NDUFS2,NDUFS3,NDUFS4,NDUFS5,NDUFS6,NDUFS7,NDUFS8,NDUFC1,NDUFC2,NDUFC2-KCTD14,SDHA,SDHB,SDHC,SDHD,UQCRFS1,CYTB,CYC1,UQCRC1,UQCRC2,UQCRH,UQCRHL,UQCRB,UQCRQ,UQCR10,UQCR11,COX3,COX1,COX2,COX4I2,COX4I1,COX5A,COX5B,COX6A1,COX6A2,COX6B1,COX6B2,COX6C,COX7A1,COX7A2,COX7A2L,COX7B,COX7B2,COX7C,COX8C,COX8A,ATP5F1A,ATP5F1B,ATP5F1C,ATP5F1D,ATP5F1E,ATP6,ATP5PB,ATP5MC1,ATP5MC2,ATP5MC3,ATP5PD,ATP5PO,ATP5PF,ATP8,VDAC1,VDAC2,VDAC3,SLC25A4,SLC25A5,SLC25A6,SLC25A31,PPIF,CYCS,APAF1,CASP9,GPX1",Huntington disease,193

hsa05020,"PRNP,HSPA5,NCAM1,NCAM2,LAMC1,SOD1,STIP1,PRKACA,PRKACB,PRKACG,BAX,FYN,CASP12,C5,C6,C7,C8A,C8B,C8G,C9,C1QA,C1QB,C1QC,NOTCH1,MAP2K1,MAP2K2,MAPK1,MAPK3,ELK1,EGR1,CCL5,IL1A,IL1B,IL6,HSPA1A",Prion diseases,35

hsa05030,"TH,DDC,SLC18A1,SLC18A2,MAOB,MAOA,SLC6A3,DRD1,GNAS,GRM2,GRM3,GNAI1,GNAI3,GNAI2,DRD2,ADCY5,PRKACA,PRKACB,PRKACG,GPSM1,RGS9,CREB1,ATF2,ATF4,CREB3,CREB3L1,CREB3L2,CREB3L3,CREB3L4,CREB5,ATF6B,PDYN,BDNF,JUN,FOSB,NFKB1,RELA,CDK5,CDK5R1,GRIN1,GRIN2A,GRIN2B,GRIN2C,GRIN2D,GRIN3A,GRIN3B,DLG4,PPP1R1B,GRIA2",Cocaine addiction,49

hsa05031,"TH,DDC,SLC18A1,SLC18A2,MAOB,MAOA,SLC6A3,CACNA1C,CACNA1D,GRIN1,GRIN2A,GRIN2B,GRIN2C,GRIN2D,GRIN3A,GRIN3B,GRIA1,GRIA2,GRIA3,GRIA4,DRD1,PRKCA,PRKCB,PRKCG,STX1A,CAMK2A,CAMK2D,CAMK2B,CAMK2G,CALML3,CALM2,CALM3,CALM1,CALML6,CALML5,CALML4,CAMK4,GNAS,ADCY5,PRKACA,PRKACB,PRKACG,PPP1R1B,PPP1CA,PPP1CB,PPP1CC,CREB1,ATF2,ATF4,CREB3,CREB3L1,CREB3L2,CREB3L3,CREB3L4,CREB5,ATF6B,PDYN,ARC,FOS,PPP3CA,PPP3CB,PPP3CC,PPP3R1,PPP3R2,JUN,FOSB,SIRT1,HDAC1",Amphetamine addiction,68

hsa05032,"OPRM1,DRD1,ADCY1,ADCY2,ADCY3,ADCY4,ADCY5,ADCY6,ADCY7,ADCY8,ADCY9,SLC32A1,KCNJ3,KCNJ6,KCNJ9,KCNJ5,CACNA1A,CACNA1B,GABRA1,GABRA2,GABRA3,GABRA4,GABRA5,GABRA6,GABRB1,GABRB3,GABRB2,GABRG1,GABRG2,GABRG3,GABRD,GABRE,GABRQ,GABRP,GABRR1,GABRR2,GABRR3,GABBR1,GABBR2,GNAI1,GNAI3,GNAI2,GNAO1,GNB1,GNB2,GNB3,GNB4,GNB5,GNG2,GNG3,GNG4,GNG5,GNG7,GNG8,GNG10,GNG11,GNG12,GNG13,GNGT1,GNGT2,GNAS,GRK2,GRK3,GRK4,GRK5,GRK6,ARRB1,ARRB2,PRKCA,PRKCB,PRKCG,PRKACA,PRKACB,PRKACG,PDE1A,PDE1B,PDE1C,PDE2A,PDE3A,PDE3B,PDE4A,PDE4B,PDE4C,PDE4D,PDE7A,PDE7B,PDE8B,PDE8A,PDE10A,PDE11A,ADORA1",Morphine addiction,91

hsa05033,"SLC32A1,CHRNA6,CACNA1A,CACNA1B,CHRNA4,CHRNB2,GABRA1,GABRA2,GABRA3,GABRA4,GABRA5,GABRA6,GABRB1,GABRB3,GABRB2,GABRG1,GABRG2,GABRG3,GABRD,GABRE,GABRQ,GABRP,GABRR1,GABRR2,GABRR3,SLC17A6,SLC17A8,SLC17A7,CHRNA7,GRIN1,GRIN2A,GRIN2B,GRIN2C,GRIN2D,GRIN3A,GRIN3B,GRIA1,GRIA2,GRIA3,GRIA4",Nicotine addiction,40

hsa05034,"TH,DDC,SLC18A1,SLC18A2,MAOB,MAOA,SLC6A3,GRIN1,GRIN2A,GRIN2B,GRIN2C,GRIN2D,GRIN3A,GRIN3B,HDAC1,HDAC2,HDAC3,HDAC4,HDAC5,HDAC6,HDAC7,HDAC8,HDAC9,HDAC10,HDAC11,H2AX,H2AC20,H2AC12,H2AC1,H2AW,H2AB3,H2AC8,H2AC4,MACROH2A2,MACROH2A1,H2AC19,H2AJ,H2AB1,H2AC17,H2AC18,H2AC11,H2AC21,H2AZ2,H2AC7,H2AZ1,H2AC15,H2AC6,H2AC13,H2AC14,H2AC16,H2AB2,H2BC15,H2BC9,H2BC14,H2BW1,H2BC1,H2BC18,H2BC17,H2BC3,H2BC8,H2BC13,H2BC4,H2BC5,H2BC6,H2BC10,H2BC21,H2BC11,H2BC12,H2BU1,H2BC7,H2BW2,H3-5,H3-3B,H3C4,H3C3,H3C1,H3-3A,H3-4,H3C14,H3C15,H3C13,H3C6,H3C11,H3C8,H3C12,H3C10,H3C2,H3C7,H4-16,H4C15,H4C9,H4C4,H4C6,H4C12,H4C11,H4C3,H4C8,H4C2,H4C5,H4C13,H4C14,H4C1,H4C7,HAT1,DRD1,GNAS,DRD2,GNAI1,GNAI3,GNAI2,GNAO1,GNB1,GNB2,GNB3,GNB4,GNB5,GNG2,GNG3,GNG4,GNG5,GNG7,GNG8,GNG10,GNG11,GNG12,GNG13,GNGT1,GNGT2,ADORA2A,ADORA2B,ADCY5,CREB1,ATF2,ATF4,CREB3,CREB3L1,CREB3L2,CREB3L3,CREB3L4,CREB5,ATF6B,CRH,NPY,BDNF,NTRK2,SHC1,SHC2,SHC3,SHC4,GRB2,SOS1,SOS2,HRAS,KRAS,NRAS,ARAF,BRAF,RAF1,MAP2K1,MAPK1,MAPK3,PDYN,CALML3,CALM2,CALM3,CALM1,CALML6,CALML5,CALML4,CAMKK1,CAMKK2,CAMK4,FOSB,PPP1R1B,PPP1CA,PPP1CB,PPP1CC,PKIA,SLC29A1,PRKACA",Alcoholism,180

hsa05100,"CDH1,CTNNB1,CTNNA3,CTNNA1,CTNNA2,ARHGAP10,MET,GAB1,PIK3R1,PIK3R2,PIK3R3,PIK3CA,PIK3CD,PIK3CB,CRK,CRKL,DOCK1,CDC42,RAC1,WAS,WASL,WASF1,WASF2,ARPC1B,ARPC1A,ARPC2,ARPC3,ARPC4,ARPC5,ARPC5L,ACTB,ACTG1,SEPTIN1,SEPTIN2,SEPTIN9,SEPTIN12,SEPTIN3,SEPTIN6,SEPTIN11,SEPTIN8,CBL,SHC1,SHC2,SHC3,SHC4,CD2AP,DNM1,DNM3,DNM2,CLTA,CLTB,CLTC,CLTCL1,CAV1,CAV2,CAV3,CTTN,HCLS1,SRC,PTK2,BCAR1,PXN,FN1,ITGA5,ITGB1,ILK,MAD2L2,ARHGEF26,RHOG,ELMO1,ELMO2,ELMO3,RHOA,VCL",Bacterial invasion of epithelial cells,74

hsa05110,"ATP6V1A,ATP6V1B1,ATP6V1B2,ATP6V1C2,ATP6V1C1,ATP6V1D,ATP6V1E2,ATP6V1E1,ATP6V1F,ATP6V1G1,ATP6V1G3,ATP6V1G2,ATP6V0E1,ATP6V0E2,TCIRG1,ATP6V0A2,ATP6V0A4,ATP6V0A1,ATP6V0D1,ATP6V0D2,ATP6V1H,ATP6AP1,ATP6V0C,ATP6V0B,KDELR1,KDELR2,KDELR3,PDIA4,ERO1A,SEC61A1,SEC61A2,SEC61B,SEC61G,ARF1,GNAS,ADCY3,ADCY9,PRKACA,PRKACB,PRKACG,CFTR,KCNQ1,SLC12A2,ACTB,ACTG1,PLCG1,PLCG2,PRKCA,TJP1,TJP2",Vibrio cholerae infection,50

hsa05120,"PTPN11,MET,PLCG1,PLCG2,TJP1,F11R,JAM2,JAM3,IGSF5,ADAM17,HBEGF,EGFR,CXCL8,CXCR1,CXCR2,ADAM10,SRC,LYN,CSK,RAC1,CDC42,MAPK11,MAPK12,MAPK13,MAPK14,PAK1,MAP2K4,MAPK8,MAPK10,MAPK9,JUN,MAP3K14,CHUK,IKBKB,IKBKG,NFKBIA,NFKB1,RELA,NOD1,CXCL1,CXCL2,CXCL3,CCL5,PTPRZ1,GIT1,CASP3,ATP6V1A,ATP6V1B1,ATP6V1B2,ATP6V1C2,ATP6V1C1,ATP6V1D,ATP6V1E2,ATP6V1E1,ATP6V1F,ATP6V1G1,ATP6V1G3,ATP6V1G2,ATP6V0E1,ATP6V0E2,TCIRG1,ATP6V0A2,ATP6V0A4,ATP6V0A1,ATP6V0D1,ATP6V0D2,ATP6V1H,ATP6AP1,ATP6V0C,ATP6V0B",Epithelial cell signaling in Helicobacter pylori infection,70

hsa05130,"TLR5,CD14,TLR4,LY96,TUBA1B,TUBA4A,TUBA3C,TUBA1A,TUBA1C,TUBA8,TUBA3E,TUBA3D,TUBAL3,TUBB6,TUBB,TUBB1,TUBB2A,TUBB3,TUBB4A,TUBB8,TUBB2B,TUBB4B,ARHGEF2,RHOA,ROCK1,ROCK2,NCK1,NCK2,WAS,WASL,ARPC1B,ARPC1A,ARPC2,ARPC3,ARPC4,ARPC5,ARPC5L,ACTB,ACTG1,FYN,ABL1,CTTN,HCLS1,NCL,ITGB1,CDC42,OCLN,EZR,PRKCA,CDH1,CTNNB1,YWHAQ,KRT18,YWHAZ,CLDN1",Pathogenic Escherichia coli infection,55

hsa05131,"ITGA5,ITGB1,CD44,RHOG,ELMO1,ELMO2,ELMO3,DOCK1,RAC1,WASF1,WASF2,ARPC5,ARPC5L,ARPC4,ARPC3,ARPC1B,ARPC1A,ARPC2,SRC,CTTN,HCLS1,ABL1,CRK,CRKL,CDC42,VCL,DIAPH1,ROCK1,ROCK2,WAS,WASL,ACTB,ACTG1,PFN3,PFN1,PFN2,PFN4,ATG5,NOD1,NOD2,RIPK2,MAPK8,MAPK10,MAPK9,MAPK1,MAPK3,MAPK11,MAPK12,MAPK13,MAPK14,IKBKG,CHUK,IKBKB,NFKBIB,NFKBIA,NFKB1,RELA,CXCL8,U2AF1,U2AF1L5,U2AF1L4,UBE2D4,UBE2D1,UBE2D2,UBE2D3,BTRC,FBXW11,MAD2L2",Shigellosis,68

hsa05132,"CD14,TLR4,LBP,MYD88,TLR5,NLRC4,PYCARD,CASP1,RAC1,CDC42,RHOG,MAPK11,MAPK12,MAPK13,MAPK14,MAPK1,MAPK3,MAPK8,MAPK10,MAPK9,FOS,JUN,NFKB1,RELA,IL18,IL1B,IL1A,IL6,CXCL8,CCL3,CCL3L1,CCL3L3,CCL4,CCL4L2,CCL4L1,CXCL1,CXCL2,CXCL3,CSF2,WASF1,WASF2,WAS,WASL,ARPC5,ARPC5L,ARPC4,ARPC3,ARPC1B,ARPC1A,ARPC2,TJP1,ACTB,ACTG1,PKN1,PKN3,PKN2,ROCK1,ROCK2,PLEKHM2,KLC3,KLC1,KLC2,KLC4,RAB7A,RAB7B,RILP,DYNC1H1,DYNC2H1,DYNC1I1,DYNC1I2,DYNC1LI2,DYNC1LI1,PFN3,PFN1,PFN2,PFN4,FLNA,FLNC,FLNB,IFNG,IFNGR1,IFNGR2,NOS2",Salmonella infection,83

hsa05133,"ITGA5,ITGB1,ITGAM,ITGB2,CALML3,CALM2,CALM3,CALM1,CALML6,CALML5,CALML4,RHOA,CFL1,CFL2,CASP3,CASP7,CASP1,PYCARD,NLRP3,IL1B,C1QA,C1QB,C1QC,C1R,C1S,C2,C4A,C4B,C3,C5,SERPING1,C4BPA,C4BPB,LY96,TLR4,CD14,TIRAP,MYD88,IRAK4,IRAK1,TRAF6,NFKB1,RELA,TICAM2,TICAM1,IRF3,MAPK11,MAPK12,MAPK13,MAPK14,MAPK8,MAPK10,MAPK9,MAPK1,MAPK3,FOS,JUN,IL6,IL12A,IL12B,IL23A,TNF,IL10,IRF1,IRF8,SFTPA1,SFTPA2,GNAI1,GNAI3,GNAI2,CXCL8,CXCL5,CXCL6,NOD1,IL1A,NOS2",Pertussis,76

hsa05134,"HSPD1,C3,CR1,ITGAM,ITGB2,ARF1,RAB1A,RAB1B,SEC22B,SAR1A,SAR1B,VCP,APAF1,CYCS,CASP9,CASP3,CASP8,BNIP3,BCL2L13,NFKB2,NFKBIA,NFKB1,RELA,CLK4,CLK1,HBS1L,EEF1A1,EEF1A2,HSF1,HSPA8,HSPA1A,HSPA2,HSPA1L,HSPA1B,HSPA6,EEF1G,NAIP,CASP7,NLRC4,PYCARD,CASP1,IL18,IL1B,TLR5,TLR2,CD14,TLR4,MYD88,TNF,IL6,IL12A,IL12B,CXCL8,CXCL1,CXCL2,CXCL3",Legionellosis,56

hsa05135,"FN1,ITGA4,ITGA5,ITGB1,SRC,BCAR1,CRK,CRKL,DOCK1,RAC1,RAC2,RAC3,PIP5K1C,PIP5K1A,PIP5K1B,ARF6,WAS,WASL,ACTR2,ACTR3B,ACTR3C,ACTR3,ACTB,ACTG1,WIPF2,WIPF1,WIPF3,BAIAP2,WASF2,PTK2B,PTK2,PXN,GIT2,ARHGEF7,CDC42,ARHGEF12,ARHGEF1,ARHGEF28,RHOA,ROCK1,ROCK2,FYB1,SKAP2,NLRP3,NLRP4,PKN1,PKN3,PKN2,RPS6KA3,RPS6KA1,RPS6KA2,RPS6KA6,MEFV,LIMK1,PYCARD,CASP1,IL18,IL1B,TLR4,MYD88,IRAK4,IRAK1,TRAF6,TRAF2,TAB1,TAB2,MAP3K7,IKBKG,CHUK,IKBKB,NFKBIA,NFKB1,RELA,MAP2K1,MAP2K2,MAPK1,MAPK3,MAP2K3,MAP2K6,MAPK11,MAPK12,MAPK13,MAPK14,MAP2K4,MAP2K7,MAPK8,MAPK10,MAPK9,FOS,JUN,TICAM1,TBK1,IRF3,TNF,IL6,CXCL8,IFNB1,PIK3CA,PIK3CD,PIK3CB,PIK3R1,PIK3R2,PIK3R3,AKT1,AKT2,AKT3,GSK3B,CCL2,LCK,ZAP70,LAT,LCP2,VAV3,VAV1,VAV2,PLCG1,NFATC1,NFATC2,NFATC3,IL2,IL10",Yersinia infection,121

hsa05140,"TLR2,TLR4,MYD88,IRAK1,IRAK4,TRAF6,MAP3K7,TAB1,TAB2,NFKBIB,NFKBIA,NFKB1,RELA,IL1A,IL1B,IL12A,IL12B,TNF,IL4,NOS2,IL10,TGFB1,TGFB2,TGFB3,C3,CR1,ITGAM,ITGB2,LOC102723407,FCGR1A,FCGR2A,FCGR2C,FCGR3A,FCGR3B,ITGA4,ITGB1,PTGS2,PRKCB,NCF1,NCF2,NCF4,CYBB,CYBA,MAPK1,MAPK3,ELK1,FOS,JUN,MAPK11,MAPK12,MAPK13,MAPK14,IFNG,IFNGR1,IFNGR2,JAK1,JAK2,STAT1,HLA-DMA,HLA-DMB,HLA-DOA,HLA-DOB,HLA-DPA1,HLA-DPB1,HLA-DQA1,HLA-DQA2,HLA-DQB1,HLA-DRA,HLA-DRB1,HLA-DRB3,HLA-DRB4,HLA-DRB5,MARCKSL1,PTPN6",Leishmaniasis,74

hsa05142,"TLR4,TICAM1,TLR2,TLR6,TLR9,MYD88,IRAK1,IRAK4,TRAF6,MAP2K4,MAPK8,MAPK10,MAPK9,MAPK1,MAPK3,MAPK11,MAPK12,MAPK13,MAPK14,FOS,JUN,IKBKG,CHUK,IKBKB,NFKBIA,NFKB1,RELA,IFNB1,CCL5,CCL2,TNF,IL12A,IL12B,IL6,CXCL8,CCL3,CCL3L1,CCL3L3,IL1B,PPP2R1B,PPP2R1A,PPP2R2A,PPP2R2B,PPP2R2C,PPP2R2D,PPP2CA,PPP2CB,IFNG,IFNGR1,IFNGR2,TNFRSF1A,NOS2,C3,CALR,C1QA,C1QB,C1QC,IL10,TGFB1,TGFB2,TGFB3,ACE,KNG1,BDKRB2,GNAQ,GNA11,GNA14,GNA15,PLCB1,PLCB2,PLCB3,PLCB4,GNAI1,GNAI3,GNAI2,GNAO1,GNAS,GNAL,ADCY1,PIK3R1,PIK3R2,PIK3R3,PIK3CA,PIK3CD,PIK3CB,AKT1,AKT2,AKT3,TGFBR2,TGFBR1,SMAD2,SMAD3,SERPINE1,FASLG,FAS,FADD,CASP8,CFLAR,CD3D,CD3E,CD3G,CD247,IL2",Chagas disease (American trypanosomiasis),103

hsa05143,"LOC102723407,TLR9,MYD88,THOP1,KNG1,NPPA,APOA1,APOL1,HPR,HBA1,HBA2,HBB,IL10,IL12A,IL12B,IL18,IFNG,TNF,IL1B,IL6,FASLG,FAS,VCAM1,SELE,ICAM1,LAMA4,IDO1,IDO2,F2RL1,GNAQ,PLCB1,PLCB2,PLCB3,PLCB4,PRKCA,PRKCB,PRKCG",African trypanosomiasis,37

hsa05144,"SDC1,SDC2,LRP1,CD81,HGF,MET,ACKR1,GYPA,GYPB,GYPC,HBA1,HBA2,HBB,TLR9,MYD88,TLR2,TLR4,IL10,TGFB1,TGFB2,TGFB3,CXCL8,IL6,CCL2,CSF3,IL1B,TNF,IL12A,IL18,KLRB1,KLRK1,KLRC4-KLRK1,IFNG,CD36,CR1,PECAM1,VCAM1,THBS1,COMP,THBS2,THBS3,THBS4,SELE,ITGAL,ITGB2,ICAM1,CD40LG,CD40,SELP",Malaria,49

hsa05145,"IFNG,IFNGR1,IFNGR2,JAK1,JAK2,STAT1,CIITA,HLA-DMA,HLA-DMB,HLA-DOA,HLA-DOB,HLA-DPA1,HLA-DPB1,HLA-DQA1,HLA-DQA2,HLA-DQB1,HLA-DRA,HLA-DRB1,HLA-DRB3,HLA-DRB4,HLA-DRB5,IRGM,NOS2,SOCS1,MYD88,IRAK1,IRAK4,TRAF6,MAP3K7,TAB1,TAB2,MAPK1,MAPK3,MAPK8,MAPK10,MAPK9,MAP2K3,MAP2K6,MAPK11,MAPK12,MAPK13,MAPK14,CHUK,IKBKB,IKBKG,NFKBIA,NFKBIB,NFKB1,RELA,IL12A,IL12B,TNF,BCL2,BCL2L1,BIRC2,BIRC3,XIAP,BIRC7,BIRC8,LY96,TLR4,HSPA8,HSPA1A,HSPA2,HSPA1L,HSPA1B,HSPA6,TLR2,LAMA1,LAMA2,LAMA3,LAMA5,LAMA4,LAMB1,LAMB2,LAMB3,LAMB4,LAMC1,LAMC2,LAMC3,ITGA6,ITGB1,LDLR,TNFRSF1A,CASP8,CASP3,CYCS,CASP9,GNAI1,GNAI3,GNAI2,GNAO1,PIK3CG,PIK3R5,PIK3R6,PDPK1,AKT1,AKT2,AKT3,BAD,CD40LG,CD40,PPIF,CCR5,ALOX5,IL10,IL10RA,IL10RB,TYK2,STAT3,TGFB1,TGFB2,TGFB3",Toxoplasmosis,113

hsa05146,"IL1B,IL1R1,IL1R2,NFKB1,RELA,HSPB1,MUC2,COL1A1,COL1A2,COL4A2,COL4A4,COL4A6,COL4A1,COL4A5,COL4A3,FN1,LAMA1,LAMA2,LAMA3,LAMA5,LAMA4,LAMB1,LAMB2,LAMB3,LAMB4,LAMC1,LAMC2,LAMC3,CASP3,LOC102723407,GNAQ,GNA11,GNA14,GNA15,PLCB1,PLCB2,PLCB3,PLCB4,PRKCA,PRKCB,PRKCG,GNAS,GNAL,ADCY1,PRKACA,PRKACB,PRKACG,RAB5A,RAB5B,RAB5C,RAB7A,RAB7B,TLR2,TLR4,CD14,IL6,CSF2,CXCL8,TNF,IL12A,IL12B,IFNG,COL3A1,PTK2,VCL,ACTN1,ACTN4,ARG2,ARG1,NOS2,ITGB2,ITGAM,PIK3R1,PIK3R2,PIK3R3,PIK3CA,PIK3CD,PIK3CB,SERPINB3,SERPINB4,SERPINB6,SERPINB9,SERPINB10,SERPINB13,CTSG,IL10,TGFB1,TGFB2,TGFB3,C8A,C8B,C8G,C9,CXCL1,CD1D",Amoebiasis,95

hsa05150,"KRT9,KRT10,KRT12,KRT13,KRT14,KRT15,KRT16,KRT17,KRT18,KRT19,KRT31,KRT32,KRT33A,KRT33B,KRT34,KRT35,KRT40,KRT39,KRT23,KRT25,KRT28,KRT24,KRT27,KRT20,KRT38,KRT37,KRT36,LOC100653049,KRT26,FGG,C3,CFB,CFD,CFH,MBL2,MASP1,MASP2,LOC102723407,C1QA,C1QB,C1QC,C1R,C1S,C2,C4A,C4B,C5,C3AR1,C5AR1,FCGR1A,FCGR2A,FCGR2B,FCGR2C,FCGR3A,FCGR3B,FCAR,FPR3,FPR2,FPR1,PLG,CFI,SELPLG,SELP,ICAM1,ITGAL,ITGAM,ITGB2,DEFB1,DEFB4A,DEFB4B,DEFB103A,DEFB103B,DEFA1,DEFA3,DEFA4,DEFA5,DEFA6,DEFA1B,CAMP,DSG1,HLA-DMA,HLA-DMB,HLA-DOA,HLA-DOB,HLA-DPA1,HLA-DPB1,HLA-DQA1,HLA-DQA2,HLA-DQB1,HLA-DRA,HLA-DRB1,HLA-DRB3,HLA-DRB4,HLA-DRB5,PTAFR,IL10",Staphylococcus aureus infection,96

hsa05152,"TNF,TNFRSF1A,TRADD,FADD,CASP8,CASP10,CASP3,BID,BAX,CYCS,CASP9,APAF1,AKT1,AKT2,AKT3,BAD,BCL2,CAMK2A,CAMK2D,CAMK2B,CAMK2G,IFNG,IFNGR1,IFNGR2,JAK1,JAK2,STAT1,CIITA,RFX5,RFXANK,RFXAP,NFYA,NFYB,NFYC,CREB1,HLA-DMA,HLA-DMB,HLA-DOA,HLA-DOB,HLA-DPA1,HLA-DPB1,HLA-DQA1,HLA-DQA2,HLA-DQB1,HLA-DRA,HLA-DRB1,HLA-DRB3,HLA-DRB4,HLA-DRB5,CD74,CREBBP,EP300,IL10,IL10RA,IL10RB,CTSS,CLEC4E,FCER1G,CLEC7A,SRC,SYK,CARD9,MALT1,BCL10,NOD2,RIPK2,HSPA9,HSPD1,LBP,TLR2,TLR1,TLR6,TLR4,CD14,TIRAP,MYD88,IRAK4,IRAK1,IRAK2,TRAF6,NFKB1,RELA,MAPK11,MAPK12,MAPK13,MAPK14,MAPK1,MAPK3,MAPK8,MAPK10,MAPK9,NOS2,IL6,IL12A,IL12B,IL18,IL23A,IL1A,IL1B,CEBPB,CEBPG,TLR9,IFNA1,IFNA2,IFNA4,IFNA5,IFNA6,IFNA7,IFNA8,IFNA10,IFNA13,IFNA14,IFNA16,IFNA17,IFNA21,IFNB1,CLEC4M,CD209,ARHGEF12,RHOA,LSP1,PLK3,KSR1,RAF1,TGFB1,TGFB2,TGFB3,CYP27B1,VDR,CAMP,C3,CR1,ITGAX,ITGB2,ITGAM,PLA2R1,MRC1,MRC2,SPHK1,SPHK2,CALML3,CALM2,CALM3,CALM1,CALML6,CALML5,CALML4,PIK3C3,RAB5A,RAB5B,RAB5C,EEA1,RAB7A,CTSD,TCIRG1,ATP6V0A2,ATP6V0A4,ATP6V0A1,ATP6V0D1,ATP6V0D2,ATP6V1H,ATP6AP1,ATP6V0C,ATP6V0B,LAMP1,LAMP2,PPP3CA,PPP3CB,PPP3CC,PPP3R1,PPP3R2,CORO1A,LOC102723407,FCGR1A,FCGR2A,FCGR2B,FCGR2C,FCGR3A,FCGR3B",Tuberculosis,179

hsa05160,"LDLR,SCARB1,CD81,CLDN4,CLDN3,CLDN7,CLDN19,CLDN16,CLDN14,CLDN15,CLDN17,CLDN20,CLDN11,CLDN18,CLDN22,CLDN5,CLDN10,CLDN8,CLDN6,CLDN2,CLDN1,CLDN9,CLDN23,CLDN25,CLDN24,OCLN,OAS1,OAS2,OAS3,RNASEL,DDX58,MAVS,TRAF3,TBK1,IKBKE,IRF3,IRF7,IFNA1,IFNA2,IFNA4,IFNA5,IFNA6,IFNA7,IFNA8,IFNA10,IFNA13,IFNA14,IFNA16,IFNA17,IFNA21,IFNB1,IFNG,TLR3,TICAM1,TRAF6,RIPK1,CHUK,IKBKB,IKBKG,NFKBIA,NFKB1,RELA,TNF,CXCL10,EIF2AK1,EIF2AK2,EIF2AK3,EIF2AK4,IFIT1,IFIT1B,EIF3E,EIF2S1,STAT3,SOCS3,IFNAR1,IFNAR2,JAK1,TYK2,STAT1,STAT2,IRF9,MX1,RSAD2,PPP2R1B,PPP2R1A,PPP2R2A,PPP2R2B,PPP2R2C,PPP2R2D,PPP2CA,PPP2CB,PIAS1,EGF,EGFR,GRB2,SOS1,SOS2,HRAS,KRAS,NRAS,BRAF,RAF1,ARAF,YWHAZ,YWHAB,YWHAQ,YWHAE,YWHAH,YWHAG,MAP2K1,MAP2K2,MAPK1,MAPK3,PIK3R1,PIK3R2,PIK3R3,PIK3CA,PIK3CD,PIK3CB,AKT1,AKT2,AKT3,GSK3B,CTNNB1,BAD,CDKN1A,TP53,CDK2,CDK4,CDK6,RB1,E2F1,E2F2,E2F3,TNFRSF1A,TRADD,TRAF2,FASLG,FAS,FADD,CASP8,CASP3,CFLAR,BID,BAX,BAK1,CYCS,APAF1,CASP9,PSME3,RXRA,PPARA,NR1H3,CCND1,MYC",Hepatitis C,155

hsa05161,"HSPG2,SLC10A1,TGFB1,TGFB2,TGFB3,TGFBR1,TGFBR2,SMAD2,SMAD3,CDKN1A,MAPK8,MAPK10,MAPK9,MYC,EGR2,EGR3,CREBBP,EP300,FASLG,DDB1,DDB2,MAP3K1,YWHAZ,YWHAB,YWHAQ,MAP2K4,PIK3CA,PIK3CD,PIK3CB,PIK3R1,PIK3R2,PIK3R3,AKT1,AKT2,AKT3,CHUK,IKBKB,IKBKG,NFKBIA,NFKB1,RELA,MMP9,BCL2,BAD,FAS,FADD,CASP8,CASP10,BID,BAX,APAF1,CYCS,CASP9,CASP3,CASP12,BIRC5,TP53,CCNA2,CCNA1,CDK2,CCNE1,CCNE2,RB1,E2F1,E2F2,E2F3,ATP6AP1,PRKCA,PRKCB,PRKCG,NFATC1,NFATC2,NFATC3,NFATC4,TNF,JAK1,JAK2,JAK3,TYK2,STAT3,STAT1,STAT2,STAT4,STAT5A,STAT5B,STAT6,VDAC3,PTK2B,SRC,GRB2,SOS1,SOS2,HRAS,KRAS,NRAS,JUN,SMAD4,ATF2,CREB1,CREB3,CREB3L1,CREB3L2,CREB3L3,CREB3L4,ATF4,CREB5,ATF6B,CXCL8,PCNA,ARAF,BRAF,RAF1,MAP2K1,MAP2K2,MAPK1,MAPK3,FOS,ELK1,TLR2,MYD88,TIRAP,TLR4,IRAK4,IRAK1,TRAF6,TAB1,TAB2,MAP3K7,IL6,MAP2K3,MAP2K6,MAPK11,MAPK12,MAPK13,MAPK14,MAP2K7,TICAM2,TLR3,TICAM1,DDX58,IFIH1,MAVS,TRAF3,IKBKE,TBK1,DDX3X,IRF3,IRF7,IFNA1,IFNA2,IFNA4,IFNA5,IFNA6,IFNA7,IFNA8,IFNA10,IFNA13,IFNA14,IFNA16,IFNA17,IFNA21,IFNB1,IFNAR1",Hepatitis B,163

hsa05162,"CD46,MSN,SLAMF1,CLEC4M,CD209,DDX58,IFIH1,MAVS,TRAF3,IKBKE,TBK1,IRF3,CHUK,IKBKB,IKBKG,NFKBIB,NFKBIA,NFKB1,RELA,TLR7,TLR9,MYD88,IRAK1,IRAK4,IRF7,IFNA1,IFNA2,IFNA4,IFNA5,IFNA6,IFNA7,IFNA8,IFNA10,IFNA13,IFNA14,IFNA16,IFNA17,IFNA21,IFNB1,TLR2,TLR4,TRAF6,CSNK2A1,CSNK2A2,CSNK2A3,CSNK2B,TNFAIP3,MAP3K7,TAB2,MAPK8,MAPK10,MAPK9,JUN,FOS,IL1A,IL1B,IL6,IL12A,IL12B,IFNAR1,IFNAR2,RACK1,JAK1,TYK2,STAT1,STAT2,IRF9,OAS1,OAS2,OAS3,ADAR,MX1,EIF2AK1,EIF2AK2,EIF2AK3,EIF2AK4,RAB9A,RAB9B,HSPA8,HSPA1A,HSPA2,HSPA1L,HSPA1B,HSPA6,EIF3H,EIF2S1,RCHY1,TP53,TP73,BBC3,FASLG,FAS,TRADD,FADD,CASP8,BID,BAX,BAK1,BCL2,BCL2L1,BAD,CYCS,APAF1,CASP9,CASP3,CDKN1B,CCND1,CCND2,CCND3,CCNE1,CCNE2,CDK4,CDK6,CDK2,CD3D,CD3E,CD3G,CD28,IL2,IL2RA,IL2RB,IL2RG,PIK3R1,PIK3R2,PIK3R3,PIK3CA,PIK3CD,PIK3CB,CBLB,AKT1,AKT2,AKT3,GSK3B,JAK3,STAT3,STAT5A,STAT5B,FCGR2B",Measles,138

hsa05163,"PDGFRA,PIK3CA,PIK3CD,PIK3CB,PIK3R1,PIK3R2,PIK3R3,AKT1,AKT2,AKT3,TSC1,TSC2,RHEB,MTOR,EIF4EBP1,RPS6KB1,RPS6KB2,CHUK,IKBKB,IKBKG,NFKB1,RELA,EGFR,GRB2,SOS1,SOS2,HRAS,KRAS,NRAS,RAF1,MAP2K1,MAP2K2,MAPK1,MAPK3,ELK1,SP1,ITGAV,ITGB3,SRC,RAC1,RAC2,RAC3,RHOA,CGAS,STING1,TBK1,IRF3,IFNA1,IFNA2,IFNA4,IFNA5,IFNA6,IFNA7,IFNA8,IFNA10,IFNA13,IFNA14,IFNA16,IFNA17,IFNA21,IFNB1,TNF,TNFRSF1A,RIPK1,TRADD,TRAF5,TRAF2,IL1B,IL1R1,NFKBIA,CCL2,CCL5,CXCL8,IL6,GNB1,GNB2,GNB3,GNB4,GNB5,GNG2,GNG3,GNG4,GNG5,GNG7,GNG8,GNG10,GNG11,GNG12,GNG13,GNGT1,GNGT2,GNAQ,GNA11,PLCB1,PLCB2,PLCB3,PLCB4,ITPR1,ITPR2,ITPR3,CALML3,CALM2,CALM3,CALM1,CALML6,CALML5,CALML4,PPP3CA,PPP3CB,PPP3CC,PPP3R1,PPP3R2,NFATC1,NFATC2,NFATC3,NFATC4,PTGS2,GNAI1,GNAI3,GNAI2,GNAO1,ADCY1,ADCY2,ADCY3,ADCY4,ADCY5,ADCY6,ADCY7,ADCY8,ADCY9,PRKACA,PRKACB,PRKACG,CCL3,CCL3L1,CCL3L3,CCL4,CCL4L2,CCL4L1,CX3CL1,GNA12,GNA13,ARHGEF1,ARHGEF11,ARHGEF12,AKAP13,ROCK1,ROCK2,CTNNB1,PTGER1,PTGER2,PTGER3,PTGER4,GNAS,GSK3B,CCND1,MYC,IL6R,JAK1,STAT3,VEGFA,MAP2K6,MAPK11,MAPK12,MAPK13,MAPK14,CREB1,ATF2,ATF4,CREB3,CREB3L1,CREB3L2,CREB3L3,CREB3L4,CREB5,ATF6B,CXCL12,CXCR4,PRKCA,PRKCB,PRKCG,PTK2B,PTK2,BCAR1,CRK,CRKL,PXN,IL10RA,IL10RB,CXCR2,CCR1,CCR3,CCR5,TAP1,TAP2,TAPBP,PDIA3,CALR,HLA-A,HLA-B,HLA-C,HLA-F,HLA-G,HLA-E,B2M,CDKN2A,MDM2,TP53,CDKN1A,CDK4,CDK6,RB1,E2F1,E2F2,E2F3,FASLG,FAS,FADD,CASP8,BID,BAX,BAK1,CYCS,CASP9,CASP3",Human cytomegalovirus infection,225

hsa05164,"TPSD1,TPSAB1,TPSB2,PLG,PRSS3,PRSS2,PRSS1,TMPRSS2,TMPRSS4,TMPRSS11D,PRKCB,OAS1,OAS2,OAS3,RNASEL,DNAJB1,DNAJC3,EIF2AK2,EIF2S1,TRIM25,DDX58,IFIH1,NLRX1,MAVS,CHUK,IKBKB,IKBKG,NFKBIB,NFKBIA,NFKB1,RELA,CALCOCO2,TLR3,TICAM1,TRAF3,TBK1,IKBKE,IRF3,CREBBP,EP300,TLR4,TLR7,MYD88,IRAK4,IRF7,IL1A,IL1B,IL6,IL12A,IL12B,TNF,CXCL8,CCL2,CCL5,CXCL10,ICAM1,IFNA1,IFNA2,IFNA4,IFNA5,IFNA6,IFNA7,IFNA8,IFNA10,IFNA13,IFNA14,IFNA16,IFNA17,IFNA21,IFNB1,IFNAR1,IFNAR2,JAK1,TYK2,SOCS3,STAT1,STAT2,IRF9,MX1,ADAR,PML,IFNG,IFNGR1,IFNGR2,JAK2,CIITA,HLA-DMA,HLA-DMB,HLA-DOA,HLA-DOB,HLA-DPA1,HLA-DPB1,HLA-DQA1,HLA-DQA2,HLA-DQB1,HLA-DRA,HLA-DRB1,HLA-DRB3,HLA-DRB4,HLA-DRB5,NLRP3,PYCARD,CASP1,IL18,IL33,PIK3R1,PIK3R2,PIK3R3,PIK3CA,PIK3CD,PIK3CB,AKT1,AKT2,AKT3,CCND3,CDK4,CDK6,TNFSF10,TNFRSF10A,TNFRSF10B,FASLG,FAS,TNFRSF1A,TRADD,FADD,CASP8,BID,BAX,BAK1,VDAC1,SLC25A4,SLC25A5,SLC25A6,SLC25A31,CYCS,APAF1,CASP9,CASP3,CPSF4,PABPN1,PABPN1L,BCL2L2-PABPN1,NXF1,NXF2,NXF2B,NXF5,NXF3,NXT1,NXT2,HNRNPUL1,RAE1,NUP98,RSAD2,FDPS,PRKCA,RAF1,MAP2K1,MAP2K2,MAPK1,MAPK3,ACTB,ACTG1,RAB11A,RAB11B,XPO1,KPNA1,KPNA6,KPNA5,KPNA2,KPNA7",Influenza A,170

hsa05165,"WNT1,WNT2,WNT2B,WNT3,WNT3A,WNT4,WNT5A,WNT5B,WNT6,WNT7A,WNT7B,WNT8A,WNT8B,WNT9A,WNT9B,WNT10B,WNT10A,WNT11,WNT16,FZD1,FZD7,FZD2,FZD3,FZD4,FZD5,FZD8,FZD6,FZD10,FZD9,DVL3,DVL2,DVL1,GSK3B,AXIN1,AXIN2,APC,APC2,CTNNB1,CSNK1A1L,CSNK1A1,TERT,TCF7,TCF7L1,TCF7L2,UBE3A,TLR3,TICAM1,TRAF3,IKBKE,TBK1,IRF3,IFNB1,CREBBP,EP300,TADA3,TP53,MAGI1,PTEN,PARD3,PARD6A,PARD6G,PARD6B,PRKCI,PRKCZ,CDC42,SCRIB,DLG1,DLG2,DLG3,LLGL2,LLGL1,CRB3,PATJ,MPP5,VWF,THBS1,COMP,THBS2,THBS3,THBS4,FN1,COL1A1,COL1A2,COL2A1,COL4A2,COL4A4,COL4A6,COL4A1,COL4A5,COL4A3,COL6A1,COL6A2,COL6A3,COL6A6,COL6A5,COL9A1,COL9A2,COL9A3,LAMA1,LAMA2,LAMA3,LAMA5,LAMA4,LAMB1,LAMB2,LAMB3,LAMB4,LAMC1,LAMC2,LAMC3,CHAD,RELN,SPP1,VTN,TNC,TNN,TNR,TNXB,IBSP,ITGA1,ITGA2,ITGA2B,ITGA3,ITGA4,ITGA5,ITGA6,ITGA7,ITGA8,ITGA9,ITGA10,ITGA11,ITGAV,ITGB1,ITGB3,ITGB4,ITGB5,ITGB6,ITGB7,ITGB8,PTK2,PXN,TNF,TNFRSF1A,TRADD,FASLG,FAS,FADD,CASP8,CASP3,SLC9A3R1,PPP2CA,PPP2CB,PPP2R1B,PPP2R1A,PPP2R2A,PPP2R2B,PPP2R2C,PPP2R2D,PPP2R3B,PPP2R3C,PPP2R3A,PPP2R5B,PPP2R5C,PPP2R5D,PPP2R5E,PPP2R5A,TSC1,TSC2,RHEB,MTOR,EIF4EBP1,RPS6KB1,RPS6KB2,BAK1,BAD,PDGFRB,PIK3CA,PIK3CD,PIK3CB,PIK3R1,PIK3R2,PIK3R3,AKT1,AKT2,AKT3,FOXO1,CDKN1B,CDKN1A,CCND1,CCND2,CCND3,CDK4,CDK6,CCNE1,CCNE2,CDK2,RB1,RBL1,RBL2,PSMC1,E2F1,CCNA2,CCNA1,ATM,ATR,VEGFA,CHUK,IKBKB,IKBKG,NFKB1,RELA,MDM2,NFX1,HDAC1,HDAC2,CHD4,IRF1,TBPL2,TBPL1,TBP,HLA-A,HLA-B,HLA-C,HLA-F,HLA-G,HLA-E,BCAP31,ATP6V1A,ATP6V1B1,ATP6V1B2,ATP6V1C2,ATP6V1C1,ATP6V1D,ATP6V1E2,ATP6V1E1,ATP6V1F,ATP6V1G1,ATP6V1G3,ATP6V1G2,ATP6V1H,TCIRG1,ATP6V0A2,ATP6V0A4,ATP6V0A1,ATP6V0D1,ATP6V0D2,ATP6V0E1,ATP6V0E2,ATP6V0C,ATP6V0B,ATP6AP1,EGF,EGFR,GRB2,SOS1,SOS2,HRAS,KRAS,NRAS,RAF1,MAP2K1,MAP2K2,MAPK1,MAPK3,PTGS2,PTGER4,GNAS,PRKACA,PRKACB,PRKACG,BAX,CREB1,CREB3,CREB3L1,CREB3L2,CREB3L3,CREB3L4,CREB5,UBR4,TUBG2,TUBG1,PKM,IFNA1,IFNA2,IFNA4,IFNA5,IFNA6,IFNA7,IFNA8,IFNA10,IFNA13,IFNA14,IFNA16,IFNA17,IFNA21,IFNAR1,IFNAR2,JAK1,TYK2,STAT1,STAT2,IRF9,EIF2AK2,OASL,MX1,ISG15,JAG1,NOTCH1,NOTCH2,NOTCH3,NOTCH4,PSEN1,MFNG,LFNG,RFNG,MAML3,MAML2,MAML1,RBPJL,RBPJ,HES1,HES2,HES6,HES7,HES3,HES4,HES5,HEYL,HEY1,HEY2",Human papillomavirus infection,330

hsa05166,"SLC2A1,NRP1,MYC,TRRAP,KAT5,CCND2,ITGAL,ITGB2,ICAM1,HRAS,KRAS,NRAS,MAP2K1,MAP2K2,MAPK1,MAPK3,CD4,TLN1,TLN2,RANBP3,RAN,XPO1,CALR,CANX,PPP3CA,PPP3CB,PPP3CC,PPP3R1,PPP3R2,NFATC1,NFATC2,NFATC3,NFATC4,IL2RB,IL2RG,JAK1,JAK3,STAT5A,STAT5B,IL2,HLA-A,HLA-B,HLA-C,HLA-F,HLA-G,HLA-E,B2M,FDPS,VDAC1,SLC25A4,SLC25A5,SLC25A6,SLC25A31,VDAC2,VDAC3,TSPO,VAC14,RANBP1,MAD1L1,MAD2L1,BUB1B,BUB3,ANAPC1,ANAPC2,CDC27,ANAPC4,ANAPC5,CDC16,ANAPC7,CDC23,ANAPC10,ANAPC11,CDC26,CDC20,PTTG1,PTTG2,ESPL1,CCNB2,CDKN2B,CDKN2A,CDK4,CCND1,CCND3,CDKN1A,CCNA2,CCNA1,CCNE1,CCNE2,CDK2,RB1,E2F1,E2F2,E2F3,ATM,ATR,CHEK1,CHEK2,TP53,PIK3R1,PIK3R2,PIK3R3,PIK3CA,PIK3CD,PIK3CB,AKT1,AKT2,AKT3,DLG1,PTEN,TNF,TNFRSF1A,MAP3K1,MAP2K4,MAPK8,MAPK10,MAPK9,JUN,MMP7,IL1R1,IL1R2,CD3D,CD3E,CD3G,MAP3K3,CHUK,IKBKB,IKBKG,NFKBIA,NFKB1,RELA,GPS2,LTBR,CD40,TNFRSF13C,MAP3K14,RELB,NFKB2,ZFP36,IL2RA,IL15,IL15RA,IL6,CSF2,LTA,BCL2L1,XIAP,TGFB1,TGFB2,TGFB3,TGFBR1,TGFBR2,SMAD2,SMAD3,SMAD4,ADCY1,ADCY2,ADCY3,ADCY4,ADCY5,ADCY6,ADCY7,ADCY8,ADCY9,PRKACA,PRKACB,PRKACG,NFYB,HLA-DMA,HLA-DMB,HLA-DOA,HLA-DOB,HLA-DPA1,HLA-DPB1,HLA-DQA1,HLA-DQA2,HLA-DQB1,HLA-DRA,HLA-DRB1,HLA-DRB3,HLA-DRB4,HLA-DRB5,SRF,ELK4,ELK1,SPI1,ETS1,ETS2,TBPL2,TBPL1,TBP,FOS,EGR1,FOSL1,EGR2,CREB1,CREB3,CREB3L1,CREB3L2,CREB3L3,CREB3L4,ATF2,ATF4,CREB5,ATF6B,CRTC1,CRTC2,CRTC3,CREBBP,EP300,KAT2B,KAT2A,TCF3,POLB,LCK,BAX,CDKN2C,TERT,MSX1,MSX2",Human T-cell leukemia virus 1 infection,219

hsa05167,"C3,TLR3,TICAM1,TRAF3,IKBKE,TBK1,IRF7,IFNA1,IFNA2,IFNA4,IFNA5,IFNA6,IFNA7,IFNA8,IFNA10,IFNA13,IFNA14,IFNA16,IFNA17,IFNA21,IRF3,CREBBP,EP300,IFNB1,IFNAR1,IFNAR2,JAK1,TYK2,STAT1,STAT2,IRF9,EIF2AK2,MAPK11,MAPK12,MAPK13,MAPK14,MAPKAPK2,MAP2K6,ZFP36,IL6,CSF2,CCR3,CCR5,CCR1,CCR4,CCR8,FAS,FADD,CASP8,BID,BAX,BAK1,CYCS,CASP9,CASP3,TNFRSF1A,TRADD,TRAF2,CHUK,IKBKB,IKBKG,NFKBIA,NFKB1,RELA,TP53,CDKN1A,CDK4,CDK6,RB1,E2F1,E2F2,E2F3,HLA-A,HLA-B,HLA-C,HLA-F,HLA-G,HLA-E,IFNGR1,ICAM1,CD86,MICB,MICA,CLEC2B,UBB,BECN1,BECN2,ATG14,PIK3C3,ATG3,GABARAP,GABARAPL1,GABARAPL2,GNB1,GNB2,GNB3,GNB4,GNB5,GNG2,GNG3,GNG4,GNG5,GNG7,GNG8,GNG10,GNG11,GNG12,GNG13,GNGT1,GNGT2,HRAS,KRAS,NRAS,RAF1,MAP2K1,MAP2K2,MAPK1,MAPK3,HIF1A,JUN,FOS,PIK3CG,PIK3R5,PIK3R6,PIK3CA,PIK3CD,PIK3CB,PIK3R1,PIK3R2,PIK3R3,AKT1,AKT2,AKT3,MTOR,PREX1,RAC1,CD200R1L,CD200R1,MAPK8,MAPK10,MAPK9,SYK,SRC,HCK,LYN,MAP2K4,MAP2K7,PLCG2,PLCG1,ITPR1,ITPR2,ITPR3,CALML3,CALM2,CALM3,CALM1,CALML6,CALML5,CALML4,PPP3CA,PPP3CB,PPP3CC,PPP3R1,PPP3R2,NFATC1,NFATC2,NFATC3,NFATC4,CXCL8,PTGS2,CXCL1,CXCL2,CXCL3,RCAN1,FGF2,VEGFA,PDGFB,ANGPT2,IL6ST,JAK2,STAT3,GSK3B,CTNNB1,CREB1,CCND1,MYC",Kaposi sarcoma-associated herpesvirus infection,186

hsa05168,"LTA,TNFSF14,TNFRSF14,TRAF2,TRAF5,IKBKB,CHUK,IKBKG,NFKBIA,NFKB1,RELA,BIRC2,BIRC3,CCL2,NECTIN1,PILRA,PTPN11,ITGA5,ITGB3,SRC,SYK,CARD9,IRF3,IFNB1,TP53,C3,C5,CFP,TLR2,TLR9,MYD88,IRF7,TLR3,TICAM1,IRAK4,IRAK1,TRAF6,MAP3K7,TAB1,TAB2,IL1B,CCL5,IL6,TNF,IL12A,IL12B,DDX58,IFIH1,MAVS,TRAF3,IKBKE,TBK1,IFNA1,IFNA2,IFNA4,IFNA5,IFNA6,IFNA7,IFNA8,IFNA10,IFNA13,IFNA14,IFNA16,IFNA17,IFNA21,CGAS,STING1,PPP1CA,PPP1CB,PPP1CC,EIF2AK1,EIF2AK2,EIF2AK3,EIF2AK4,EIF2S1,EIF2B1,EIF2B2,EIF2B3,EIF2B4,EIF2B5,OAS1,OAS2,OAS3,RNASEL,HCFC2,HCFC1,POU2F1,POU2F2,POU2F3,IFNAR1,IFNAR2,SOCS3,JAK1,TYK2,STAT1,STAT2,IRF9,ZNF583,ZNF558,ZNF98,ZNF555,ZNF569,ZNF570,ZNF595,ZNF425,ZNF746,ZNF782,ZNF519,ZNF836,ZNF610,ZNF320,ZNF550,ZNF846,ZNF791,ZNF564,ZNF709,ZNF433,ZNF383,ZNF780B,ZNF100,ZNF540,ZNF596,ZNF169,ZNF431,ZNF675,ZNF627,ZNF584,ZNF25,ZFP30,ZNF510,ZNF620,ZNF718,ZNF549,ZNF324,ZNF473,ZNF658,ZNF337,ZNF285,ZNF777,ZNF544,ZNF311,ZNF547,ZIK1,ZNF615,ZNF763,ZFP82,ZNF875,ZNF256,ZNF443,ZNF267,ZNF211,ZNF234,ZNF273,ZNF460,ZNF268,ZNF275,ZNF257,ZIM3,ZNF554,ZNF689,ZNF354B,ZNF641,ZNF816,ZNF543,ZNF813,ZNF12,ZNF14,ZNF708,ZNF17,ZNF19,ZNF20,ZNF182,ZNF26,ZNF33A,ZNF33B,ZNF37A,ZNF41,ZNF43,ZNF45,ZNF79,ZNF84,ZNF221,ZNF85,ZNF91,ZNF99,ZNF222,ZNF124,ZNF132,ZNF133,ZNF135,ZNF136,ZNF140,ZNF141,ZNF154,ZNF155,ZNF157,ZNF175,ZNF177,ZNF180,ZNF184,ZNF189,ZNF195,ZNF205,ZNF208,ZNF214,ZNF223,ZNF224,ZNF225,ZNF226,ZNF227,ZNF112,ZNF229,ZNF230,ZNF426,ZNF343,ZNF557,ZNF419,ZNF665,ZNF552,ZNF669,ZNF212,ZNF671,ZNF613,ZNF442,ZNF556,ZNF606,ZNF614,ZNF430,ZNF34,ZNF436,ZNF611,ZNF93,ZNF282,ZNF528,ZNF333,ZNF527,ZNF559,ZNF347,ZNF577,ZNF607,ZNF514,ZNF382,ZNF587,ZNF566,ZNF551,ZNF616,ZNF766,ZNF468,ZNF160,ZNF799,ZNF700,ZNF439,ZNF486,ZNF479,ZNF682,ZNF765,ZNF300,ZNF461,ZNF585B,ZNF764,ZNF235,ZNF670,ZNF101,ZNF264,ZNF254,ZNF432,ZNF114,ZNF440,ZNF57,ZNF684,ZNF786,ZFP92,ZFP28,ZFP90,ZNF597,ZNF785,ZNF480,ZNF534,ZNF418,ZNF417,ZNF548,ZNF560,ZNF563,ZNF420,ZNF565,ZNF582,ZNF621,ZNF454,ZNF707,ZNF354C,ZNF546,ZFP69,ZNF680,ZNF517,ZNF404,ZNF284,ZNF677,ZNF860,ZFP57,ZNF530,ZNF713,ZNF429,ZNF233,ZNF699,ZNF568,ZNF773,ZNF790,ZNF470,ZNF324B,ZNF793,ZNF772,ZNF705A,ZNF506,ZNF716,ZNF571,ZNF589,ZNF44,ZNF853,ZSCAN32,ZNF331,ZNF823,ZNF416,ZNF334,ZNF701,ZNF415,ZNF302,ZNF253,ZNF248,ZNF286A,ZNF304,ZNF490,ZNF624,ZNF471,ZNF492,ZFP14,ZNF317,RBAK,ZNF77,ZNF250,ZNF350,ZNF667,ZNF674,ZNF649,ZNF354A,ZNF736,ZNF705D,ZNF878,ZFP37,ZNF3,ZNF7,ZNF8,ZNF10,ZNF484,ZFP1,ZNF567,ZNF792,ZNF829,ZNF90,ZNF81,ZNF30,ZNF251,ZNF688,ZNF2,ZNF23,ZNF561,ZNF730,ZNF599,ZNF783,ZNF398,ZNF283,ZNF879,ZNF662,ZNF562,ZNF630,ZNF200,ZNF778,ZNF623,ZNF727,ZNF679,ZNF768,ZFP69B,ZNF181,ZNF705E,ZNF729,ZNF850,ZNF605,ZNF559-ZNF177,ZNF891,ZNF714,ZNF676,ZNF721,ZNF585A,ZNF619,ZNF761,ZNF107,ZNF83,ZNF529,ZNF814,ZNF735,ZNF845,ZNF749,ZNF841,ZNF316,ZNF717,ZNF780A,ZNF806,ZNF286B,ZNF74,ZNF726,ZNF600,ZNF737,BST2,IFNG,IFNGR1,IFNGR2,JAK2,DAXX,SP100,PML,TAP1,TAP2,TAPBP,PDIA3,CALR,HLA-A,HLA-B,HLA-C,HLA-F,HLA-G,HLA-E,B2M,HLA-DMA,HLA-DMB,HLA-DOA,HLA-DOB,HLA-DPA1,HLA-DPB1,HLA-DQA1,HLA-DQA2,HLA-DQB1,HLA-DRA,HLA-DRB1,HLA-DRB3,HLA-DRB4,HLA-DRB5,CD74,PIK3CA,PIK3CD,PIK3CB,PIK3R1,PIK3R2,PIK3R3,AKT1,AKT2,AKT3,TSC1,TSC2,RHEB,MTOR,EIF4EBP1,SRPK1,SRSF1,SRSF2,SRSF8,SRSF3,SRSF4,SRSF5,SRSF6,SRSF7,SRSF9,NXF1,NXF2,NXF2B,NXF5,NXF3,ALYREF,FASLG,FAS,TNFRSF1A,TRADD,FADD,CASP8,BID,BAX,BAK1,BAD,BCL2,BCL2L1,CYCS,APAF1,CASP9,CASP3",Herpes simplex virus 1 infection,492

hsa05169,"CR2,CD19,HLA-DMA,HLA-DMB,HLA-DOA,HLA-DOB,HLA-DPA1,HLA-DPB1,HLA-DQA1,HLA-DQA2,HLA-DQB1,HLA-DRA,HLA-DRB1,HLA-DRB3,HLA-DRB4,HLA-DRB5,TLR2,MYD88,IRAK4,IRAK1,TRAF6,TAB1,TAB2,MAP3K7,CHUK,IKBKB,IKBKG,NFKBIA,NFKB1,RELA,TNF,IL6,DDX58,MAVS,TRAF3,TBK1,IKBKE,IRF7,IRF3,IFNA1,IFNA2,IFNA4,IFNA5,IFNA6,IFNA7,IFNA8,IFNA10,IFNA13,IFNA14,IFNA16,IFNA17,IFNA21,IFNB1,IFNAR1,IFNAR2,JAK1,TYK2,STAT1,STAT2,IRF9,EIF2AK2,OAS1,OAS2,OAS3,CXCL10,ISG15,FAS,FADD,CASP8,BID,BCL2L11,BCL2,BAX,BAK1,CYCS,APAF1,CASP9,CASP3,CD3E,CD3G,CD3D,CD247,PSMC2,PSMC1,PSMC4,PSMC6,PSMC3,PSMC5,PSMD2,PSMD1,PSMD3,PSMD12,PSMD11,PSMD6,PSMD7,PSMD13,PSMD4,PSMD14,PSMD8,ADRM1,SEM1,TAP1,TAP2,TAPBP,PDIA3,CALR,HLA-A,HLA-B,HLA-C,HLA-F,HLA-G,HLA-E,B2M,SNW1,SIN3A,HDAC1,HDAC2,NCOR2,SAP30,CIR1,RBPJL,RBPJ,HES1,FCER2,RUNX3,MYC,USP7,MDM2,TP53,CDKN1A,GADD45A,GADD45B,GADD45G,DDB2,POLK,TRAF5,PIK3CA,PIK3CD,PIK3CB,PIK3R1,PIK3R2,PIK3R3,AKT1,AKT2,AKT3,MAP3K14,RELB,NFKB2,TRADD,NFKBIB,NFKBIE,MAP2K4,MAP2K7,MAPK8,MAPK10,MAPK9,TRAF2,RIPK1,MAP2K3,MAP2K6,MAPK11,MAPK12,MAPK13,MAPK14,JUN,ENTPD3,ENTPD8,ENTPD1,CD40,CD44,ITGAL,CD58,ICAM1,VIM,TNFAIP3,JAK3,STAT3,LYN,SYK,NEDD4,RAC1,LOC102723407,BTK,BLNK,PLCG2,SKP2,CDKN1B,CCND1,CCND2,CCND3,CDK4,CDK6,CCNA2,CCNA1,CCNE1,CCNE2,CDK2,RB1,E2F1,E2F2,E2F3",Epstein-Barr virus infection,201

hsa05170,"CD4,BTRC,FBXW11,SKP1,CUL1,RBX1,CXCR4,CCR5,GNAI1,GNAI3,GNAI2,GNAO1,PIK3CA,PIK3CD,PIK3CB,PIK3R1,PIK3R2,PIK3R3,PTK2,PTK2B,PXN,CRK,CRKL,AKT1,AKT2,AKT3,MTOR,RPS6KB1,RPS6KB2,NFKB1,RELA,FOS,JUN,BAD,HRAS,KRAS,NRAS,RAF1,MAP2K1,MAP2K2,MAPK1,MAPK3,GNAQ,GNA11,PLCG1,PLCG2,ITPR1,ITPR2,ITPR3,CALML3,CALM2,CALM3,CALM1,CALML6,CALML5,CALML4,PPP3CA,PPP3CB,PPP3CC,PPP3R1,PPP3R2,NFATC1,NFATC2,NFATC3,NFATC4,PRKCA,PRKCB,PRKCG,GNB1,GNB2,GNB3,GNB4,GNB5,GNG2,GNG3,GNG4,GNG5,GNG7,GNG8,GNG10,GNG11,GNG12,GNG13,GNGT1,GNGT2,RAC1,RAC2,RAC3,PAK1,PAK2,PAK3,PAK4,PAK5,PAK6,BUB1B-PAK6,LIMK1,LIMK2,CFL1,CFL2,TNF,TNFRSF1B,BCL2L1,TLR2,TLR4,MYD88,IRAK4,IRAK1,TRAF6,TAB1,TAB2,MAP3K7,CHUK,IKBKB,IKBKG,NFKBIA,IFNA1,IFNA2,IFNA4,IFNA5,IFNA6,IFNA7,IFNA8,IFNA10,IFNA13,IFNA14,IFNA16,IFNA17,IFNA21,IFNB1,MAPK11,MAPK12,MAPK13,MAPK14,MAPK8,MAPK10,MAPK9,CGAS,STING1,TBK1,IRF3,APOBEC3H,APOBEC3F,APOBEC3A_B,APOBEC3D,APOBEC3A,APOBEC3C,APOBEC3G,APOBEC3B,SAMHD1,TRIM5,BST2,TNFRSF1A,RIPK1,TRADD,TRAF5,TRAF2,MAP2K3,MAP2K6,MAP2K7,FASLG,FAS,FADD,CASP8,BID,BAX,BAK1,BCL2,CYCS,CASP9,CASP3,CD3E,CD3G,CD3D,CD247,TAP1,TAP2,TAPBP,PDIA3,CALR,HLA-A,HLA-B,HLA-C,HLA-F,HLA-G,HLA-E,AP1B1,AP1G1,AP1G2,AP1M1,AP1M2,AP1S1,AP1S2,AP1S3,B2M,ELOB,ELOC,CUL5,RNF7,DCAF1,DDB1,CUL4B,CUL4A,ATM,ATR,CHEK1,CDC25C,WEE1,WEE2,CCNB1,CCNB2,CCNB3,CDK1",Human immunodeficiency virus 1 infection,212

hsa05200,"DCC,CASP3,CASP9,APPL1,CDH1,CTNNB1,CTNNA3,CTNNA1,CTNNA2,AXIN1,AXIN2,APC,APC2,GSK3B,TCF7,TCF7L1,TCF7L2,LEF1,BIRC5,MYC,CCND1,WNT1,WNT2,WNT2B,WNT3,WNT3A,WNT4,WNT5A,WNT5B,WNT6,WNT7A,WNT7B,WNT8A,WNT8B,WNT9A,WNT9B,WNT10B,WNT10A,WNT11,WNT16,FZD1,FZD7,FZD2,FZD3,FZD4,FZD5,FZD8,FZD6,FZD10,FZD9,LRP5,LRP6,DVL3,DVL2,DVL1,FRAT1,FRAT2,F2,AGT,F2R,F2RL3,LPAR1,LPAR2,LPAR3,LPAR4,LPAR5,LPAR6,AGTR1,GNA12,GNA13,ARHGEF12,ARHGEF11,ARHGEF1,PLEKHG5,RHOA,ROCK1,ROCK2,CXCL12,CXCR4,GNAI1,GNAI3,GNAI2,PTGER1,PTGER2,PTGER3,PTGER4,GNAS,ADCY1,ADCY2,ADCY3,ADCY4,ADCY5,ADCY6,ADCY7,ADCY8,ADCY9,PRKACA,PRKACB,PRKACG,GNB1,GNB2,GNB3,GNB4,GNB5,GNG2,GNG3,GNG4,GNG5,GNG7,GNG8,GNG10,GNG11,GNG12,GNG13,GNGT1,GNGT2,COL4A2,COL4A4,COL4A6,COL4A1,COL4A5,COL4A3,LAMA1,LAMA2,LAMA3,LAMA5,LAMA4,LAMB1,LAMB2,LAMB3,LAMB4,LAMC1,LAMC2,LAMC3,FN1,ITGA2,ITGA2B,ITGA3,ITGA6,ITGAV,ITGB1,PTK2,PIK3CA,PIK3CD,PIK3CB,PIK3R1,PIK3R2,PIK3R3,PTEN,NKX3-1,AKT1,AKT2,AKT3,CHUK,IKBKB,IKBKG,NFKBIA,NFKB1,NFKB2,RELA,PTGS2,NOS2,BCL2,BIRC2,BIRC3,XIAP,BIRC7,BIRC8,BCL2L1,TRAF1,TRAF2,TRAF3,TRAF4,TRAF5,TRAF6,MTOR,RPS6KB1,RPS6KB2,BAD,FOXO1,MDM2,TP53,CDKN1B,CDKN1A,BCR,ABL1,CRK,CRKL,CBL,STAT5A,STAT5B,PIM1,PIM2,KNG1,EDN1,BDKRB1,BDKRB2,EDNRA,EDNRB,GNAQ,GNA11,PLCB1,PLCB2,PLCB3,PLCB4,PRKCA,PRKCB,PRKCG,IL2,IL3,IL4,IL5,IL6,IL7,IL12A,IL12B,IL13,IL15,IL23A,EPO,IFNA1,IFNA2,IFNA4,IFNA5,IFNA6,IFNA7,IFNA8,IFNA10,IFNA13,IFNA14,IFNA16,IFNA17,IFNA21,IFNG,IL2RA,IL2RB,IL2RG,IL3RA,IL4R,CSF2RB,IL5RA,IL6R,IL6ST,IL7R,IL12RB1,IL12RB2,IL13RA1,IL15RA,IL23R,EPOR,IFNAR1,IFNAR2,IFNGR1,IFNGR2,EML4,ALK,RASGRP1,RASGRP2,RASGRP3,RASGRP4,JAK1,JAK2,JAK3,STAT3,STAT1,STAT2,STAT4,STAT6,VEGFA,VEGFB,PGF,VEGFC,VEGFD,TGFA,EGF,EGFR,ERBB2,PDGFA,PDGFB,PDGFRA,PDGFRB,IGF1,IGF2,IGF1R,KITLG,KIT,FLT3LG,FLT3,HGF,MET,FGF1,FGF2,FGF3,FGF4,FGF17,FGF6,FGF7,FGF8,FGF9,FGF10,FGF16,FGF5,FGF18,FGF20,FGF22,FGF19,FGF21,FGF23,FGFR1,FGFR2,FGFR3,FGFR4,GRB2,SOS1,SOS2,HRAS,KRAS,NRAS,ARAF,BRAF,RAF1,MAP2K1,MAP2K2,MAPK1,MAPK3,JUN,FOS,ETS1,MMP1,MMP2,MMP9,CXCL8,CDK4,RPS6KA5,RET,CCDC6,NCOA4,NTRK1,TPM3,TPR,TFG,RASSF1,RASSF5,STK4,DAPK1,DAPK3,DAPK2,PLCG1,PLCG2,ELK1,CALML3,CALM2,CALM3,CALM1,CALML6,CALML5,CALML4,CAMK2A,CAMK2D,CAMK2B,CAMK2G,RALGDS,RALA,RALB,RALBP1,CDC42,RAC1,RAC2,RAC3,MAPK8,MAPK10,MAPK9,PAX8,PPARG,RXRA,RXRB,RXRG,RARB,ESR1,ESR2,NCOA1,NCOA3,SP1,PPARD,JUP,ZBTB16,PML,RARA,RUNX1,RUNX1T1,SPI1,CEBPA,CSF2RA,CSF3R,CSF1R,CDKN2A,E2F1,E2F2,E2F3,MAX,ZBTB17,CDKN2B,CDK6,CCND2,CCND3,CKS1B,CKS2,SKP1,CUL1,RBX1,SKP2,CDK2,CCNE1,CCNE2,RB1,GADD45A,GADD45B,GADD45G,BAX,BAK1,DDB2,POLK,TERT,TERC,MITF,TGFB1,TGFB2,TGFB3,TGFBR1,TGFBR2,SMAD2,SMAD3,SMAD4,MECOM,CTBP1,CTBP2,HDAC1,HDAC2,MLH1,MSH2,MSH3,MSH6,BRCA2,RAD51,FASLG,FAS,FADD,CASP8,BID,PMAIP1,BBC3,BCL2L11,CYCS,APAF1,CASP7,KEAP1,NFE2L2,HMOX1,NQO1,GSTA5,GSTA2,GSTA4,GSTO2,GSTM4,GSTT2,GSTT1,GSTM3,MGST1,MGST3,GSTP1,GSTM1,GSTM5,MGST2,GSTA1,GSTM2,GSTA3,GSTO1,GSTT2B,TXNRD1,TXNRD3,TXNRD2,VHL,ELOC,ELOB,CUL2,EGLN1,EGLN3,EGLN2,FH,HIF1A,EPAS1,ARNT,ARNT2,CREBBP,EP300,SLC2A1,JAG1,JAG2,DLL3,DLL1,DLL4,NOTCH1,NOTCH2,NOTCH3,NOTCH4,HES1,HES5,HEYL,HEY1,HEY2,FLT4,SHH,PTCH1,SMO,KIF7,SUFU,GLI1,GLI2,GLI3,BMP2,BMP4,HHIP,PTCH2,AR,HSP90AA1,HSP90AB1,HSP90B1,KLK3,PLD1,CCNA1",Pathways in cancer,530

hsa05202,"RUNX1,CSF1R,MPO,CSF2,IL3,RUNX1T1,HDAC1,HDAC2,SIN3A,NCOR1,CEBPA,PER2,SPI1,CD14,ITGAM,FCGR1A,JUP,PML,RARA,CEBPE,BCL2A1,ZBTB16,MYC,DUSP6,TCF3,PBX1,WNT16,ETV6,ETV7,ELANE,GZMB,KMT2A,AFF1,CDK9,CCNT1,CCNT2,MLLT1,MLLT3,DOT1L,LMO2,PBX3,RUNX2,SMAD1,KLF3,MEF2C,HOXA9,HOXA10,JMJD1C,HMGA2,KDM6A,UTY,SUPT3H,PROM1,FLT3,BMP2K,IGF1R,CDKN1B,CDK14,MEIS1,HOXA11,SIX1,SIX4,EYA1,CDKN2C,HPGD,GRIA3,FUT8,TLX3,TLX1,BCL11B,LDB1,LYL1,HHEX,PTCRA,REL,CCND2,TRAF1,BCL2L1,CD86,CD40,BCL6,LOC102723407,MAF,ITGB7,NSD2,H3-5,H3-3B,H3C4,H3C3,H3C1,H3-3A,H3-4,H3C14,H3C15,H3C13,H3C6,H3C11,H3C8,H3C12,H3C10,H3C2,H3C7,PAX5,PAX8,PPARG,RXRA,RXRB,RXRG,PRCC,TFE3,CDKN1A,TMPRSS2,ERG,PLAU,PLAT,MMP3,MMP9,ZEB1,IL1R2,SPINT1,ETV1,ETV4,ETV5,SLC45A3,ELK4,DDX5,MYCN,MAX,MDM2,PTK2,TP53,BMI1,COMMD3-BMI1,SP1,ZBTB17,NTRK1,NGFR,MEN1,EWSR1,FLI1,IGF1,ID2,TGFBR2,IGFBP3,FEV,ATF1,ARNT2,ATM,MITF,WT1,PDGFA,IL2RB,BAIAP3,TSPAN7,MLF1,NR4A3,TAF15,FUS,DDIT3,CEBPB,IL6,NFKBIZ,NFKB1,RELA,CXCL8,FOXO1,FLT1,SS18,SSX1,SSX2,SSX2B,NUPR1,ASPSCR1,MET,GADD45A,GADD45B,GADD45G,BAX,BAK1,DDB2,POLK,DEFA3,BIRC3,PAX3,PAX7,CCNA1",Transcriptional misregulation in cancer,186

hsa05203,"SRC,HRAS,KRAS,NRAS,MAPK1,MAPK3,CREB1,ATF2,ATF4,CREB3,CREB3L1,CREB3L2,CREB3L3,CREB3L4,CREB5,ATF6B,DDB1,YWHAZ,YWHAB,YWHAQ,YWHAE,YWHAH,YWHAG,JAK1,STAT3,STAT5A,STAT5B,CASP3,HPN,PIK3CA,PIK3CD,PIK3CB,PIK3R1,PIK3R2,PIK3R3,RELA,REL,NFKB2,NFKB1,TP53,CCNE1,CCNE2,CDK2,EGR2,EGR3,VDAC3,LTBR,SP100,HNRNPK,CDKN1A,DDX3X,EIF2AK2,PRKACA,PRKACB,PRKACG,H2BC15,H2BC9,H2BC14,H2BW1,H2BC1,H2BC18,H2BC17,H2BC3,H2BC8,H2BC13,H2BC4,H2BC5,H2BC6,H2BC10,H2BC21,H2BC11,H2BC12,H2BU1,H2BC7,H2BW2,H4-16,H4C15,H4C9,H4C4,H4C6,H4C12,H4C11,H4C3,H4C8,H4C2,H4C5,H4C13,H4C14,H4C1,H4C7,BAX,GRB2,TRADD,CDK1,TBPL2,TBPL1,TBP,USP7,MDM2,RBPJL,RBPJ,SNW1,GTF2H1,GTF2H2,GTF2H2C_2,GTF2H2C,GTF2H3,GTF2H4,GTF2E1,GTF2E2,SND1,CREBBP,EP300,GTF2B,CCND1,CCND2,CCND3,CDK6,CCNA2,CCNA1,SKP2,MRPS18B,TRAF1,TRAF2,TRAF3,TRAF5,JAK3,LYN,SYK,ATP6V0D1,ATP6V0D2,UBE3A,DLG1,SCRIB,PXN,BAK1,IRF3,PSMC1,RB1,RBL1,RBL2,CDKN1B,UBR4,JUN,CHD4,HDAC1,HDAC2,HDAC3,HDAC4,HDAC5,HDAC6,HDAC7,HDAC8,HDAC9,HDAC10,HDAC11,PKM,IRF9,DNAJA3,KAT2B,KAT2A,GTF2A1L,GTF2A1,GTF2A2,SRF,IKBKG,NFKBIA,RASA2,CDC42,RAC1,RHOA,GSN,SCIN,ACTN1,ACTN4,CDK4,CDKN2A,CDKN2B,CHEK1,CDC20,MAD1L1,VAC14,RANBP1,POLB,IRF7,IL6ST,CCR5,CCR8,CCR3,CCR4,MAPKAPK2,CASP8,BAD,PMAIP1,C3,HLA-A,HLA-B,HLA-C,HLA-F,HLA-G,HLA-E",Viral carcinogenesis,201

hsa05204,"CYP1A1,CYP3A4,CYP3A5,CYP3A7,CYP3A7-CYP3A51P,CYP3A43,PTGS2,NAT2,NAT1,CYP1A2,SULT1A2,SULT1A1,SULT1A3,SULT1A4,CYP1B1,CYP2C8,CYP2C9,CYP2C18,CYP2C19,EPHX1,ARNT,GSTA5,GSTA2,GSTA4,GSTO2,GSTM4,GSTT2,GSTT1,GSTM3,MGST1,MGST3,GSTP1,GSTM1,GSTM5,MGST2,GSTA1,GSTM2,GSTA3,GSTO1,GSTT2B,GSTK1,HPGDS,SULT2A1,CHRNA7,CBR1,HSD11B1,CYP2A6,CYP2A13,UGT2A1,UGT2A3,UGT2B17,UGT2B11,UGT2B28,UGT1A6,UGT1A4,UGT1A1,UGT1A3,UGT2B10,UGT1A9,UGT2B7,UGT1A10,UGT1A8,UGT1A5,UGT2B15,UGT1A7,UGT2B4,UGT2A2,CYP2E1,ALDH3B1,ALDH3B2,ALDH1A3,ALDH3A1,ADH1A,ADH1B,ADH1C,ADH7,ADH4,ADH6,ADH5,KYAT3,KYAT1,AKR1C2",Chemical carcinogenesis,82

hsa05205,"CD44,SRC,CTTN,HCLS1,ERBB2,GRB2,VAV3,VAV1,VAV2,HRAS,KRAS,NRAS,RRAS,RRAS2,MRAS,BRAF,RAF1,ARAF,MAP2K1,MAP2K2,MAPK1,MAPK3,RAC1,IQGAP1,CDC42,ELK1,ESR1,CCND1,ACTB,ACTG1,FLNA,FLNC,FLNB,PAK1,TIAM1,ARHGEF1,RHOA,ROCK1,ROCK2,ANK1,ANK2,ANK3,GAB1,PIK3CA,PIK3CD,PIK3CB,PIK3R1,PIK3R2,PIK3R3,AKT1,AKT2,AKT3,SLC9A1,PPP1CA,PPP1CB,PPP1CC,PPP1R12A,PPP1R12B,PPP1R12C,ARHGEF12,PLCE1,ITPR1,ITPR2,ITPR3,CAMK2A,CAMK2D,CAMK2B,CAMK2G,NANOG,DDX5,DROSHA,STAT3,MIR21,TWIST1,TWIST2,MIR10A,MIR10B,HOXD10,DCN,IGF1,IGF1R,MTOR,PDPK1,RPS6KB1,RPS6KB2,EIF4B,RPS6,EGFR,CAV1,CAV2,CAV3,CD63,CDKN1A,CASP3,TGFB1,TLR2,TLR4,PDCD4,TNF,IL12B,ERBB3,ERBB4,MYC,CTNNB1,HIF1A,TFAP4,VEGFA,KDR,MET,CBL,TIMP3,THBS1,MMP2,MMP9,LUM,FASLG,FAS,MDM2,TP53,TGFB2,HPSE,HPSE2,SDC1,PLAU,PLAUR,ITGA2,ITGB1,VTN,ITGAV,ITGB3,FN1,ITGB5,HGF,WNT1,FZD1,FZD7,FZD2,FZD3,FZD4,FZD5,FZD8,FZD6,FZD10,FZD9,ITGA5,SDC2,EZR,RDX,MSN,SDC4,FGF2,FGFR1,FRS2,PTPN11,SOS1,SOS2,PLCG1,PLCG2,PRKCA,PRKCB,PRKCG,NUDT16L1,PXN,PTK2,HBEGF,GPC1,IGF2,GPC3,WNT2,WNT2B,WNT3,WNT3A,WNT4,WNT5A,WNT5B,WNT6,WNT7A,WNT7B,WNT8A,WNT8B,WNT9A,WNT9B,WNT10B,WNT10A,WNT11,WNT16,SHH,IHH,PTCH1,SMO,HSPG2,CTSL,PRKACA,PRKACB,PRKACG,MAPK11,MAPK12,MAPK13,MAPK14,HSPB2,PTPN6,SMAD2,COL21A1",Proteoglycans in cancer,203

hsa05206,"MIR17,MIR18A,MIR19A,MIR19B2,MIR19B1,MIR20A,MIR92A1,MIR92A2,PTEN,MIR221,MIR222,CDKN1B,MIR21,TIMP3,MIRLET7A1,MIRLET7A2,MIRLET7A3,MIRLET7B,MIRLET7D,MIRLET7E,MIRLET7F1,MIRLET7F2,MIRLET7G,MIRLET7I,MIRLET7C,KRAS,CDK6,CDC25A,CDC25B,CDC25C,HMGA2,MIR107,MIR15A,MIR15B,MIR16-1,MIR16-2,CCND1,CCND2,CCNE1,CCNE2,MIR126,CRK,CRKL,MIR1-1,MIR1-2,HDAC4,HDAC5,FOXP1,PIM1,MET,MIR29A,MIR29B1,MIR29B2,MIR29C,DNMT3A,DNMT3B,MIR133A1,MIR133A2,MIR133B,MCL1,BCL2L2,MIR145,EGFR,VEGFA,MIR200C,ZEB1,MIR141,TGFB2,MIR183,EZR,MIR125A,MIR125B1,MIR125B2,PDCD4,MIR143,MIR192,MIR194-1,MIR194-2,CREBBP,EP300,MIR215,MIR28,MIR30E,MIR30D,MIR30A,MIR30B,MIR30C1,MIR30C2,TPM1,SPRY2,BCL2,SERPINB5,MIR155,MIR203A,MIR203B,MIR205,MIR27A,MIR27B,ST14,CYP1B1,MIR31,MIR99A,MIR100,MTOR,RPTOR,CYP24A1,ERBB2,ERBB3,ABL1,TP63,ZEB2,FSCN1,MIR375,MIR602,RASSF1,MIR10B,HOXD10,DDIT4,BMF,MIR224,PAK4,MMP9,MIR363,MIR494,MIR615,MIR625,MIR25,MIR34A,MIR96,MIR10A,MIR23A,MIR23B,MIR23C,MIR122,SLC7A1,CCNG1,HMOX1,MIR150,MIR223,STMN1,MIR342,MIR423,STAT3,MYC,CASP3,HRAS,NRAS,MIR199B,MIR324,MIR483,MIR26A1,MIR26A2,MIR26B,MIR152,DNMT1,MIR135B,MIR135A2,MIR135A1,APC,APC2,IRS1,PIK3R1,PIK3R2,PIK3R3,MAPK7,PIK3CA,PIK3CD,PIK3CB,MIR200A,MIR200B,HNRNPK,RECK,MIR124-1,MIR124-3,MIR124-2,MIR137,MIR128-1,MIR128-2,E2F3,MIR7-1,MIR7-2,MIR7-3,IRS2,MIR326,NOTCH1,NOTCH2,NOTCH3,NOTCH4,BMI1,COMMD3-BMI1,MIR146A,MMP16,MIR181A2,MIR181A1,MIR181B1,MIR181B2,MIR181C,MIR181D,ATM,SOCS1,MIR373,MIR520A,MIR520G,MIR520H,MIR520B,MIR520E,CD44,MIR103A1,MIR103B2,MIR103A2,MIR103B1,DICER1,FZD3,ITGA5,RDX,RHOA,MIR193B,PLAU,BAK1,ZFPM2,ITGB3,UBE2I,MIR335,SOX4,TNC,TNN,TNR,TNXB,MIR206,MIR451A,ABCB1,MIR345,ABCC1,MIR214,BRCA1,MIR106B,CDKN1A,MIR210,EFNA1,EFNA2,EFNA3,EFNA4,EFNA5,THBS1,IGF2BP1,TRIM71,MIR34B,MIR34C,MIR9-1,MIR9-2,MIR9-3,NFKB1,MIR199A1,MIR199A2,IKBKB,PTGS2,MAPK1,MAPK3,PRKCA,PRKCB,PRKCG,GRB2,FGFR3,RPS6KA5,TP53,MDM2,MDM4,PDGFRA,PLCG1,PLCG2,MIR101-1,MIR101-2,RAF1,MIR195,MAP2K1,MAP2K2,SOS1,SOS2,SHC1,PDGFA,MIR129-1,MIR129-2,SHC4,PDGFB,PDGFRB,CDKN2A,E2F1,E2F2,MIR32,BCL2L11,MARCKS,BMPR2,MIR449A,HDAC1,HDAC2,EZH2,GLS2,GLS,SIRT1,WNT3,WNT3A,MIR330,MIR331,CDCA5,KIF23,MIR520C,ROCK1,PRKCE,SLC45A3,VIM",MicroRNAs in cancer,310

hsa05210,"GSK3B,AXIN1,AXIN2,CTNNB1,APC,APC2,TCF7,TCF7L1,TCF7L2,LEF1,BIRC5,MYC,CCND1,KRAS,PIK3CA,PIK3CD,PIK3CB,PIK3R1,PIK3R2,PIK3R3,AKT1,AKT2,AKT3,BAD,CASP9,ARAF,BRAF,RAF1,MAP2K1,MAP2K2,MAPK1,MAPK3,JUN,FOS,RALGDS,RALA,RALB,RAC1,RAC2,RAC3,RHOA,MAPK8,MAPK10,MAPK9,DCC,CASP3,APPL1,TGFB1,TGFB2,TGFB3,TGFBR1,TGFBR2,SMAD2,SMAD3,SMAD4,MLH1,MSH2,MSH3,MSH6,BAX,PMAIP1,BCL2L11,BBC3,BCL2,CYCS,TP53,CDKN1A,GADD45A,GADD45B,GADD45G,BAK1,DDB2,POLK,EGF,TGFA,EREG,AREG,EGFR,GRB2,SOS1,SOS2,HRAS,NRAS,MTOR,RPS6KB1,RPS6KB2",Colorectal cancer,86

hsa05211,"HIF1A,EPAS1,EGLN1,EGLN3,EGLN2,VHL,ELOC,ELOB,RBX1,CUL2,ARNT,ARNT2,CREBBP,EP300,SLC2A1,VEGFA,TGFB1,TGFB2,TGFB3,PDGFB,TGFA,HGF,MET,GAB1,PIK3CA,PIK3CD,PIK3CB,PIK3R1,PIK3R2,PIK3R3,AKT1,AKT2,AKT3,BAD,CRK,CRKL,RAPGEF1,RAP1A,RAP1B,PTPN11,GRB2,SOS1,SOS2,HRAS,KRAS,NRAS,ARAF,BRAF,RAF1,MAP2K1,MAP2K2,MAPK1,MAPK3,ETS1,JUN,RAC1,CDC42,PAK1,PAK2,PAK3,PAK4,PAK5,PAK6,BUB1B-PAK6,PRCC,TFE3,CDKN1A,FH,FLCN",Renal cell carcinoma,69

hsa05212,"KRAS,PIK3CA,PIK3CD,PIK3CB,PIK3R1,PIK3R2,PIK3R3,ARHGEF6,RAC1,RAC2,RAC3,NFKB1,RELA,AKT1,AKT2,AKT3,CHUK,IKBKB,IKBKG,BAD,BCL2L1,CASP9,ARAF,BRAF,RAF1,MAP2K1,MAPK1,MAPK3,MAPK8,MAPK10,MAPK9,RALGDS,RALA,RALB,RALBP1,CDC42,TGFA,EGF,EGFR,ERBB2,MTOR,RPS6KB1,RPS6KB2,JAK1,STAT3,STAT1,VEGFA,CDKN2A,CDK4,CDK6,CCND1,RB1,E2F1,E2F2,E2F3,TP53,CDKN1A,GADD45A,GADD45B,GADD45G,BAX,BAK1,DDB2,POLK,TGFB1,TGFB2,TGFB3,TGFBR1,TGFBR2,SMAD2,SMAD3,SMAD4,BRCA2,RAD51,PLD1",Pancreatic cancer,75

hsa05213,"EGF,EGFR,PIK3CA,PIK3CD,PIK3CB,PIK3R1,PIK3R2,PIK3R3,PTEN,PDPK1,ILK,AKT1,AKT2,AKT3,CASP9,BAD,FOXO3,GRB2,SOS1,SOS2,HRAS,KRAS,NRAS,ARAF,BRAF,RAF1,MAP2K1,MAP2K2,MAPK1,MAPK3,ELK1,MLH1,CDH1,CTNNB1,CTNNA3,CTNNA1,CTNNA2,AXIN1,AXIN2,APC,APC2,GSK3B,TCF7,TCF7L1,TCF7L2,LEF1,MYC,CCND1,TP53,CDKN1A,GADD45A,GADD45B,GADD45G,BAX,BAK1,DDB2,POLK,ERBB2",Endometrial cancer,58

hsa05214,"EGF,TGFA,EGFR,PDGFA,PDGFB,PDGFRA,PDGFRB,IGF1,IGF1R,PLCG1,PLCG2,CALML3,CALM2,CALM3,CALM1,CALML6,CALML5,CALML4,CAMK1D,CAMK1G,CAMK1,CAMK2A,CAMK2D,CAMK2B,CAMK2G,CAMK4,PRKCA,PRKCB,PRKCG,SHC1,SHC2,SHC3,SHC4,GRB2,SOS1,SOS2,HRAS,KRAS,NRAS,ARAF,BRAF,RAF1,MAP2K1,MAP2K2,MAPK1,MAPK3,PIK3CA,PIK3CD,PIK3CB,PIK3R1,PIK3R2,PIK3R3,AKT1,AKT2,AKT3,MTOR,PTEN,CDKN2A,MDM2,TP53,CDKN1A,CCND1,CDK4,CDK6,RB1,E2F1,E2F2,E2F3,GADD45A,GADD45B,GADD45G,BAX,BAK1,DDB2,POLK",Glioma,75

hsa05215,"CDKN1B,CDK2,CCNE1,CCNE2,RB1,E2F1,E2F2,E2F3,TMPRSS2,ERG,PLAU,PLAT,MMP3,MMP9,ZEB1,IL1R2,SPINT1,ETV5,INS,PDGFA,PDGFB,PDGFC,PDGFD,EGF,TGFA,IGF1,INSRR,PDGFRA,PDGFRB,FGFR1,FGFR2,EGFR,ERBB2,IGF1R,PIK3CA,PIK3CD,PIK3CB,PIK3R1,PIK3R2,PIK3R3,PTEN,PDPK1,NKX3-1,AKT1,AKT2,AKT3,CASP9,BAD,FOXO1,CDKN1A,MDM2,TP53,GSK3B,CREB1,ATF4,CREB3,CREB3L1,CREB3L2,CREB3L3,CREB3L4,CREB5,CTNNB1,CREBBP,EP300,TCF7,TCF7L1,TCF7L2,LEF1,CCND1,CHUK,IKBKB,IKBKG,NFKBIA,NFKB1,RELA,BCL2,MTOR,GRB2,SOS1,SOS2,HRAS,KRAS,NRAS,ARAF,BRAF,RAF1,MAP2K1,MAP2K2,MAPK1,MAPK3,SRD5A2,AR,HSP90AA1,HSP90AB1,HSP90B1,KLK3,GSTP1",Prostate cancer,97

hsa05216,"RET,CCDC6,NCOA4,NTRK1,TPM3,TPR,TFG,HRAS,NRAS,KRAS,BRAF,MAP2K1,MAP2K2,MAPK1,MAPK3,PAX8,PPARG,RXRA,RXRB,RXRG,TP53,CDKN1A,GADD45A,GADD45B,GADD45G,BAX,BAK1,DDB2,POLK,CDH1,CTNNB1,TCF7,TCF7L1,TCF7L2,LEF1,MYC,CCND1",Thyroid cancer,37

hsa05217,"TP53,CDKN1A,GADD45A,GADD45B,GADD45G,BAX,BAK1,DDB2,POLK,SHH,PTCH1,SMO,KIF7,SUFU,GLI1,GLI2,GLI3,BMP2,BMP4,HHIP,PTCH2,WNT1,WNT2,WNT2B,WNT3,WNT3A,WNT4,WNT5A,WNT5B,WNT6,WNT7A,WNT7B,WNT8A,WNT8B,WNT9A,WNT9B,WNT10B,WNT10A,WNT11,WNT16,FZD1,FZD7,FZD2,FZD3,FZD4,FZD5,FZD8,FZD6,FZD10,FZD9,DVL3,DVL2,DVL1,GSK3B,AXIN1,AXIN2,APC,APC2,CTNNB1,TCF7,TCF7L1,TCF7L2,LEF1",Basal cell carcinoma,63

hsa05218,"FGF1,FGF2,FGF3,FGF4,FGF17,FGF6,FGF7,FGF8,FGF9,FGF10,FGF16,FGF5,FGF18,FGF20,FGF22,FGF19,FGF21,FGF23,HGF,IGF1,PDGFA,PDGFB,PDGFC,PDGFD,EGF,FGFR1,MET,IGF1R,PDGFRA,PDGFRB,EGFR,HRAS,KRAS,NRAS,ARAF,BRAF,RAF1,MAP2K1,MAP2K2,MAPK1,MAPK3,CCND1,CDK4,PIK3CA,PIK3CD,PIK3CB,PIK3R1,PIK3R2,PIK3R3,AKT1,AKT2,AKT3,BAD,PTEN,CDKN2A,MDM2,TP53,CDKN1A,CDK6,RB1,E2F1,E2F2,E2F3,CDH1,GADD45A,GADD45B,GADD45G,BAX,BAK1,DDB2,POLK,MITF",Melanoma,72

hsa05219,"FGFR3,HRAS,KRAS,NRAS,ARAF,BRAF,RAF1,MAP2K1,MAP2K2,MAPK1,MAPK3,RPS6KA5,MYC,RASSF1,DAPK1,DAPK3,DAPK2,CDKN2A,MDM2,TP53,CDKN1A,CCND1,CDK4,RB1,E2F1,E2F2,E2F3,THBS1,HBEGF,MMP2,MMP9,UPK3A,SRC,EGF,ERBB2,EGFR,TYMP,VEGFA,MMP1,CXCL8,CDH1",Bladder cancer,41

hsa05220,"BCR,ABL1,CRK,CRKL,CBL,PIK3CA,PIK3CD,PIK3CB,PIK3R1,PIK3R2,PIK3R3,AKT1,AKT2,AKT3,BAD,BCL2L1,CHUK,IKBKB,IKBKG,NFKBIA,NFKB1,RELA,MDM2,TP53,CDKN1B,GRB2,GAB2,PTPN11,SOS1,SOS2,HRAS,KRAS,NRAS,RAF1,ARAF,BRAF,MAP2K1,MAP2K2,MAPK1,MAPK3,SHC1,SHC2,SHC3,SHC4,MYC,STAT5A,STAT5B,CDKN2A,CDKN1A,CCND1,CDK4,CDK6,RB1,E2F1,E2F2,E2F3,GADD45A,GADD45B,GADD45G,BAX,BAK1,DDB2,POLK,TGFB1,TGFB2,TGFB3,TGFBR1,TGFBR2,SMAD4,MECOM,RUNX1,CTBP1,CTBP2,HDAC1,HDAC2,SMAD3",Chronic myeloid leukemia,76

hsa05221,"KIT,FLT3,PIK3CA,PIK3CD,PIK3CB,PIK3R1,PIK3R2,PIK3R3,AKT1,AKT2,AKT3,CHUK,IKBKB,IKBKG,NFKB1,RELA,BAD,MTOR,EIF4EBP1,RPS6KB1,RPS6KB2,GRB2,SOS1,SOS2,HRAS,NRAS,KRAS,ARAF,BRAF,RAF1,MAP2K1,MAP2K2,MAPK1,MAPK3,STAT3,STAT5A,STAT5B,PIM1,PIM2,RUNX1,CSF1R,MPO,CSF2,IL3,RUNX1T1,PML,RARA,ZBTB16,CEBPA,PER2,SPI1,CD14,ITGAM,FCGR1A,CEBPE,BCL2A1,MYC,DUSP6,JUP,TCF7,TCF7L1,TCF7L2,LEF1,CCND1,PPARD,CCNA1",Acute myeloid leukemia,66

hsa05222,"FHIT,RARB,RXRA,RXRB,RXRG,TP53,CDKN1A,GADD45A,GADD45B,GADD45G,BAX,BAK1,DDB2,POLK,BCL2,CYCS,APAF1,CASP9,CASP3,MYC,MAX,ZBTB17,CDKN2B,CDK4,CDK6,CCND1,CKS1B,CKS2,SKP2,CDKN1B,CDK2,CCNE1,CCNE2,RB1,E2F1,E2F2,E2F3,COL4A2,COL4A4,COL4A6,COL4A1,COL4A5,COL4A3,LAMA1,LAMA2,LAMA3,LAMA5,LAMA4,LAMB1,LAMB2,LAMB3,LAMB4,LAMC1,LAMC2,LAMC3,FN1,ITGA2,ITGA2B,ITGA3,ITGA6,ITGAV,ITGB1,PTK2,PIK3CA,PIK3CD,PIK3CB,PIK3R1,PIK3R2,PIK3R3,PTEN,AKT1,AKT2,AKT3,CHUK,IKBKB,IKBKG,NFKBIA,NFKB1,RELA,BIRC2,BIRC3,XIAP,BIRC7,BIRC8,BCL2L1,TRAF1,TRAF2,TRAF3,TRAF4,TRAF5,TRAF6,PTGS2,NOS2",Small cell lung cancer,93

hsa05223,"FHIT,RARB,RXRA,RXRB,RXRG,CDKN2A,CDK4,CDK6,CCND1,RB1,E2F1,E2F2,E2F3,KRAS,RASSF1,RASSF5,STK4,PIK3CA,PIK3CD,PIK3CB,PIK3R1,PIK3R2,PIK3R3,PDPK1,AKT1,AKT2,AKT3,BAD,CASP9,FOXO3,EGF,TGFA,EGFR,ERBB2,GRB2,SOS1,SOS2,HRAS,NRAS,ARAF,BRAF,RAF1,MAP2K1,MAP2K2,MAPK1,MAPK3,PLCG1,PLCG2,PRKCA,PRKCB,PRKCG,TP53,CDKN1A,GADD45A,GADD45B,GADD45G,BAX,BAK1,DDB2,POLK,EML4,ALK,JAK3,STAT3,STAT5A,STAT5B",Non-small cell lung cancer,66

hsa05224,"ESR1,ESR2,NCOA1,NCOA3,FOS,JUN,SP1,CCND1,MYC,PGR,WNT1,WNT4,TNFSF11,ERBB2,FGF1,FGF2,FGF3,FGF4,FGF17,FGF6,FGF7,FGF8,FGF9,FGF10,FGF16,FGF5,FGF18,FGF20,FGF22,FGF19,FGF21,FGF23,FGFR1,IGF1,IGF1R,EGF,EGFR,KIT,SHC1,SHC2,SHC3,SHC4,GRB2,SOS1,SOS2,HRAS,KRAS,NRAS,ARAF,BRAF,RAF1,MAP2K1,MAP2K2,MAPK1,MAPK3,PIK3CA,PIK3CD,PIK3CB,PIK3R1,PIK3R2,PIK3R3,PTEN,AKT1,AKT2,AKT3,MTOR,RPS6KB1,RPS6KB2,JAG1,JAG2,DLL3,DLL1,DLL4,NOTCH1,NOTCH2,NOTCH3,NOTCH4,HES1,HES5,HEYL,HEY1,HEY2,FLT4,CDKN1A,NFKB2,WNT2,WNT2B,WNT3,WNT3A,WNT5A,WNT5B,WNT6,WNT7A,WNT7B,WNT8A,WNT8B,WNT9A,WNT9B,WNT10B,WNT10A,WNT11,WNT16,FZD1,FZD7,FZD2,FZD3,FZD4,FZD5,FZD8,FZD6,FZD10,FZD9,LRP5,LRP6,DVL3,DVL2,DVL1,FRAT1,FRAT2,GSK3B,AXIN1,AXIN2,APC,APC2,CTNNB1,CSNK1A1L,CSNK1A1,TCF7,TCF7L1,TCF7L2,LEF1,TP53,GADD45A,GADD45B,GADD45G,BAX,BAK1,DDB2,POLK,CDK4,CDK6,RB1,E2F1,E2F2,E2F3,BRCA1,BRCA2",Breast cancer,147

hsa05225,"TGFA,EGFR,IGF2,IGF1R,PLCG1,PLCG2,PRKCA,PRKCB,PRKCG,ELK1,SHC1,SHC2,SHC3,SHC4,GRB2,SOS1,SOS2,HRAS,KRAS,NRAS,ARAF,BRAF,RAF1,MAP2K1,MAP2K2,MAPK1,MAPK3,PIK3CA,PIK3CD,PIK3CB,PIK3R1,PIK3R2,PIK3R3,PTEN,AKT1,AKT2,AKT3,MTOR,RPS6KB1,RPS6KB2,BAD,BCL2L1,MYC,RB1,E2F1,E2F2,E2F3,TERT,TERC,TP53,CDKN1A,GADD45A,GADD45B,GADD45G,BAX,BAK1,DDB2,POLK,CDKN2A,CDK4,CDK6,CCND1,TGFB1,TGFB2,TGFB3,TGFBR1,TGFBR2,SMAD2,SMAD3,SMAD4,WNT1,WNT2,WNT2B,WNT3,WNT3A,WNT4,WNT5A,WNT5B,WNT6,WNT7A,WNT7B,WNT8A,WNT8B,WNT9A,WNT9B,WNT10B,WNT10A,WNT11,WNT16,FZD1,FZD7,FZD2,FZD3,FZD4,FZD5,FZD8,FZD6,FZD10,FZD9,LRP5,LRP6,DVL3,DVL2,DVL1,FRAT1,FRAT2,GSK3B,AXIN1,AXIN2,APC,APC2,CTNNB1,CSNK1A1L,CSNK1A1,TCF7,TCF7L1,TCF7L2,LEF1,KEAP1,NFE2L2,HMOX1,NQO1,GSTA5,GSTA2,GSTA4,GSTO2,GSTM4,GSTT2,GSTT1,GSTM3,MGST1,MGST3,GSTP1,GSTM1,GSTM5,MGST2,GSTA1,GSTM2,GSTA3,GSTO1,GSTT2B,TXNRD1,TXNRD3,TXNRD2,HGF,MET,GAB1,ACTB,ACTG1,ACTL6A,ACTL6B,SMARCD1,SMARCD2,SMARCD3,PHF10,DPF1,DPF3,ARID1B,ARID1A,SMARCE1,SMARCB1,SMARCC1,SMARCC2,SMARCA2,SMARCA4,ARID2,PBRM1,BRD7",Hepatocellular carcinoma,168

hsa05226,"CDX2,MUC2,CDH17,REG4,ABCB1,SHH,TP53,CDKN1A,GADD45A,GADD45B,GADD45G,BAX,BAK1,DDB2,POLK,RARB,RXRA,RXRB,RXRG,WNT1,WNT2,WNT2B,WNT3,WNT3A,WNT4,WNT5A,WNT5B,WNT6,WNT7A,WNT7B,WNT8A,WNT8B,WNT9A,WNT9B,WNT10B,WNT10A,WNT11,WNT16,FZD1,FZD7,FZD2,FZD3,FZD4,FZD5,FZD8,FZD6,FZD10,FZD9,LRP5,LRP6,DVL3,DVL2,DVL1,FRAT1,FRAT2,GSK3B,AXIN1,AXIN2,APC,APC2,CTNNB1,CSNK1A1L,CSNK1A1,TCF7,TCF7L1,TCF7L2,LEF1,MYC,CCND1,TERT,TERC,EGF,EGFR,ERBB2,SHC1,SHC2,SHC3,SHC4,GRB2,SOS1,SOS2,HRAS,KRAS,NRAS,ARAF,BRAF,RAF1,MAP2K1,MAP2K2,MAPK1,MAPK3,PIK3CA,PIK3CD,PIK3CB,PIK3R1,PIK3R2,PIK3R3,AKT1,AKT2,AKT3,MTOR,RPS6KB1,RPS6KB2,BCL2,MLH1,CDKN1B,CDK2,CCNE1,CCNE2,RB1,E2F1,E2F2,E2F3,TGFB1,TGFB2,TGFB3,TGFBR1,TGFBR2,SMAD2,SMAD3,SMAD4,CDKN2B,CDH1,CTNNA3,CTNNA1,CTNNA2,JUP,HGF,MET,FGF1,FGF2,FGF3,FGF4,FGF17,FGF6,FGF7,FGF8,FGF9,FGF10,FGF16,FGF5,FGF18,FGF20,FGF22,FGF19,FGF21,FGF23,FGFR2,GAB1",Gastric cancer,149

hsa05230,"SLC2A1,SLC2A2,GCK,PKM,PDHA2,PDHA1,PDHB,PDK1,TP53,SLC1A5,SIRT3,SLC16A3,SIRT6,MYC,HIF1A,KIT,MET,RET,EGFR,ERBB2,NTRK1,NTRK3,PDGFRA,PDGFRB,FGFR1,FGFR2,FGFR3,FLT3,HRAS,KRAS,NRAS,RAF1,MAP2K1,MAP2K2,MAPK1,MAPK3,GLS2,GLS,G6PD,HK3,HK1,HK2,HKDC1,PFKM,PFKP,PFKL,PGAM1,PGAM2,PGAM4,LDHAL6A,LDHAL6B,LDHA,LDHB,LDHC,TIGAR,PTEN,PIK3CA,PIK3CD,PIK3CB,PIK3R1,PIK3R2,PIK3R3,AKT1,AKT2,AKT3,MTOR,SLC7A5,IDH1,SCO2",Central carbon metabolism in cancer,69

hsa05231,"EGF,PDGFA,PDGFB,PDGFC,PDGFD,EGFR,PDGFRA,PDGFRB,GRB2,SOS1,SOS2,HRAS,KRAS,NRAS,RAF1,MAP2K1,MAP2K2,MAPK1,MAPK3,RALGDS,MAPK8,MAPK10,MAPK9,PLA2G4E,PLA2G4A,JMJD7-PLA2G4B,PLA2G4B,PLA2G4C,PLA2G4D,PLA2G4F,PIK3CA,PIK3CD,PIK3CB,PIK3R1,PIK3R2,PIK3R3,PDPK1,AKT1,AKT2,AKT3,TSC1,TSC2,RHEB,MTOR,RPS6KB1,RPS6KB2,EIF4EBP1,PIP5K1C,PIP5K1A,PIP5K1B,WAS,WASL,RAC1,RAC2,RAC3,WASF1,WASF2,WASF3,SP1,PLD1,PLD2,SLC5A7,SLC44A1,SLC44A4,SLC44A5,SLC44A2,SLC44A3,SLC22A1,SLC22A2,SLC22A3,SLC22A5,SLC22A4,CHKA,CHKB,HIF1A,JUN,FOS,PCYT1B,PCYT1A,CHPT1,PLCG1,PLPP1,PLPP3,PLPP2,DGKZ,DGKD,DGKI,DGKA,DGKE,DGKB,DGKH,DGKG,DGKQ,DGKK,PRKCA,PRKCB,PRKCG,LYPLA1,GPCPD1",Choline metabolism in cancer,99

hsa05235,"HIF1A,EGF,EGFR,HRAS,KRAS,NRAS,RAF1,MAP2K1,MAP2K2,MAPK1,MAPK3,FOS,JUN,EML4,ALK,PIK3R1,PIK3R2,PIK3R3,PIK3CA,PIK3CD,PIK3CB,PTEN,AKT1,AKT2,AKT3,MTOR,RPS6KB1,RPS6KB2,CHUK,IKBKB,IKBKG,NFKBIA,NFKBIB,NFKBIE,NFKB1,RELA,IFNG,IFNGR1,IFNGR2,JAK1,JAK2,STAT1,STAT3,TLR2,TLR4,TLR9,TIRAP,MYD88,TRAF6,NFATC1,NFATC2,NFATC3,TICAM1,TICAM2,CD274,PDCD1,PTPN6,PTPN11,BATF3,BATF,BATF2,CSNK2A1,CSNK2A2,CSNK2A3,CSNK2B,CD4,LCK,CD3E,CD3G,CD247,CD3D,ZAP70,MAP3K3,MAP2K3,MAP2K6,MAPK11,MAPK12,MAPK13,MAPK14,LAT,PLCG1,PPP3CA,PPP3CB,PPP3CC,PPP3R1,PPP3R2,RASGRP1,CD28,PRKCQ",PD-L1 expression and PD-1 checkpoint pathway in cancer,89

hsa05310,"HLA-DMA,HLA-DMB,HLA-DOA,HLA-DOB,HLA-DPA1,HLA-DPB1,HLA-DQA1,HLA-DQA2,HLA-DQB1,HLA-DRA,HLA-DRB1,HLA-DRB3,HLA-DRB4,HLA-DRB5,IL4,CD40LG,CD40,LOC102723407,FCER1A,MS4A2,FCER1G,IL9,IL10,IL13,IL5,CCL11,TNF,IL3,PRG2,RNASE3,EPX",Asthma,31

hsa05320,"IFNA1,IFNA2,IFNA4,IFNA5,IFNA6,IFNA7,IFNA8,IFNA10,IFNA13,IFNA14,IFNA16,IFNA17,IFNA21,CTLA4,TPO,TG,HLA-DMA,HLA-DMB,HLA-DOA,HLA-DOB,HLA-DPA1,HLA-DPB1,HLA-DQA1,HLA-DQA2,HLA-DQB1,HLA-DRA,HLA-DRB1,HLA-DRB3,HLA-DRB4,HLA-DRB5,CD80,CD86,CD28,IL2,HLA-A,HLA-B,HLA-C,HLA-F,HLA-G,HLA-E,FASLG,FAS,PRF1,GZMB,CD40LG,CD40,IL4,IL5,IL10,LOC102723407,TSHR,CGA,TSHB",Autoimmune thyroid disease,53

hsa05321,"TLR2,TLR4,TLR5,NFKB1,RELA,NOD2,HLA-DMA,HLA-DMB,HLA-DOA,HLA-DOB,HLA-DPA1,HLA-DPB1,HLA-DQA1,HLA-DQA2,HLA-DQB1,HLA-DRA,HLA-DRB1,HLA-DRB3,HLA-DRB4,HLA-DRB5,IFNG,IFNGR1,IFNGR2,STAT1,TBX21,IL4,IL5,IL12A,IL12B,IL12RB2,IL12RB1,STAT4,IL2,IL18,IL18R1,IL18RAP,JUN,TNF,IL6,IL1A,IL1B,TGFB1,TGFB2,TGFB3,SMAD2,SMAD3,STAT3,IL21,IL21R,IL23A,IL23R,RORC,RORA,FOXP3,IL17A,IL17F,IL22,IL4R,IL2RG,STAT6,IL10,IL13,MAF,NFATC1,GATA3",Inflammatory bowel disease (IBD),65

hsa05322,"C1QA,C1QB,C1QC,C2,C4A,C4B,H2AX,H2AC20,H2AC12,H2AC1,H2AW,H2AB3,H2AC8,H2AC4,MACROH2A2,MACROH2A1,H2AC19,H2AJ,H2AB1,H2AC17,H2AC18,H2AC11,H2AC21,H2AZ2,H2AC7,H2AZ1,H2AC15,H2AC6,H2AC13,H2AC14,H2AC16,H2AB2,H2BC15,H2BC9,H2BC14,H2BW1,H2BC1,H2BC18,H2BC17,H2BC3,H2BC8,H2BC13,H2BC4,H2BC5,H2BC6,H2BC10,H2BC21,H2BC11,H2BC12,H2BU1,H2BC7,H2BW2,H3-5,H3-3B,H3C4,H3C3,H3C1,H3-3A,H3-4,H3C14,H3C15,H3C13,H3C6,H3C11,H3C8,H3C12,H3C10,H3C2,H3C7,H4-16,H4C15,H4C9,H4C4,H4C6,H4C12,H4C11,H4C3,H4C8,H4C2,H4C5,H4C13,H4C14,H4C1,H4C7,SNRPB,SNRPD1,SNRPD3,GRIN2A,GRIN2B,TRIM21,RO60,SSB,ACTN1,ACTN4,HLA-DMA,HLA-DMB,HLA-DOA,HLA-DOB,HLA-DPA1,HLA-DPB1,HLA-DQA1,HLA-DQA2,HLA-DQB1,HLA-DRA,HLA-DRB1,HLA-DRB3,HLA-DRB4,HLA-DRB5,CD80,CD86,CD28,CD40LG,CD40,TNF,IFNG,IL10,LOC102723407,C1R,C1S,C3,C5,C6,C7,C8A,C8B,C8G,C9,CTSG,ELANE,FCGR1A,FCGR2A,FCGR3A,FCGR3B",Systemic lupus erythematosus,133

hsa05323,"CD80,CD86,CD28,CTLA4,HLA-DMA,HLA-DMB,HLA-DOA,HLA-DOB,HLA-DPA1,HLA-DPB1,HLA-DQA1,HLA-DQA2,HLA-DQB1,HLA-DRA,HLA-DRB1,HLA-DRB3,HLA-DRB4,HLA-DRB5,ITGAL,ITGB2,ICAM1,IL15,TNFSF13,TNFSF13B,LOC102723407,LTB,TNF,IL1A,IL1B,IL6,IL11,IL18,TLR2,TLR4,JUN,FOS,TGFB1,TGFB2,TGFB3,IL23A,IL17A,PTH,CSF1,TNFSF11,TNFRSF11A,ATP6V1A,ATP6V1B1,ATP6V1B2,ATP6V1C2,ATP6V1C1,ATP6V1D,ATP6V1E2,ATP6V1E1,ATP6V1F,ATP6V1G1,ATP6V1G3,ATP6V1G2,ATP6V0E1,ATP6V0E2,TCIRG1,ATP6V0A2,ATP6V0A4,ATP6V0A1,ATP6V0D1,ATP6V0D2,ATP6V1H,ATP6AP1,ATP6V0C,ATP6V0B,CTSK,ACP5,MMP1,MMP3,CTSL,CSF2,CCL5,CCL2,CCL3,CCL3L1,CCL3L3,CCL20,CXCL5,CXCL6,CXCL8,CXCL12,VEGFA,FLT1,ANGPT1,TEK,IFNG,CXCL1",Rheumatoid arthritis,91

hsa05330,"HLA-A,HLA-B,HLA-C,HLA-F,HLA-G,HLA-E,CD80,CD86,CD28,IL12A,IL12B,FASLG,FAS,PRF1,GZMB,HLA-DMA,HLA-DMB,HLA-DOA,HLA-DOB,HLA-DPA1,HLA-DPB1,HLA-DQA1,HLA-DQA2,HLA-DQB1,HLA-DRA,HLA-DRB1,HLA-DRB3,HLA-DRB4,HLA-DRB5,IL2,CD40LG,CD40,IFNG,TNF,IL4,IL5,IL10,LOC102723407",Allograft rejection,38

hsa05332,"IL6,IL1A,IL1B,TNF,HLA-DMA,HLA-DMB,HLA-DOA,HLA-DOB,HLA-DPA1,HLA-DPB1,HLA-DQA1,HLA-DQA2,HLA-DQB1,HLA-DRA,HLA-DRB1,HLA-DRB3,HLA-DRB4,HLA-DRB5,CD80,CD86,CD28,IL2,HLA-A,HLA-B,HLA-C,HLA-F,HLA-G,HLA-E,FASLG,FAS,PRF1,GZMB,IFNG,KLRD1,KIR2DL1,KIR2DL2,KIR2DL3,KIR3DL1,KIR3DL2,KLRC1,KIR2DL5A",Graft-versus-host disease,41

hsa05340,"ADA,IL7R,IL2RG,DCLRE1C,RAG1,RAG2,CD3D,CD3E,PTPRC,CD4,CD8A,CD8B,AIRE,TAP1,TAP2,LCK,ZAP70,RFX5,RFXAP,RFXANK,CIITA,ORAI1,LOC102723407,CD79A,BLNK,BTK,IKBKG,CD40,CD40LG,UNG,AICDA,ICOS,TNFRSF13C,CD19,TNFRSF13B,IGLL1,JAK3",Primary immunodeficiency,37

hsa05410,"ITGA1,ITGA2,ITGA2B,ITGA3,ITGA4,ITGA5,ITGA6,ITGA7,ITGA8,ITGA9,ITGA10,ITGA11,ITGAV,ITGB1,ITGB3,ITGB4,ITGB5,ITGB6,ITGB7,ITGB8,SGCD,SGCG,SGCA,SGCB,LAMA1,LAMA2,DAG1,DES,DMD,ACTB,ACTG1,TTN,TNNT2,TNNC1,TNNI3,ACTC1,TPM1,TPM2,TPM3,TPM4,MYBPC3,MYL3,MYL2,MYH7,MYH6,EMD,LMNA,CACNA1C,CACNA1D,CACNA1F,CACNA1S,CACNB1,CACNB2,CACNB3,CACNB4,CACNA2D1,CACNA2D2,CACNA2D3,CACNA2D4,CACNG1,CACNG2,CACNG3,CACNG4,CACNG5,CACNG6,CACNG7,CACNG8,RYR2,ATP2A1,ATP2A3,ATP2A2,PRKAA1,PRKAA2,PRKAB1,PRKAB2,PRKAG1,PRKAG3,PRKAG2,ACE,IGF1,TGFB1,TGFB2,TGFB3,TNF,IL6,EDN1,AGT,SLC8A1,SLC8A2,SLC8A3",Hypertrophic cardiomyopathy (HCM),90

hsa05412,"ITGA1,ITGA2,ITGA2B,ITGA3,ITGA4,ITGA5,ITGA6,ITGA7,ITGA8,ITGA9,ITGA10,ITGA11,ITGAV,ITGB1,ITGB3,ITGB4,ITGB5,ITGB6,ITGB7,ITGB8,SGCD,SGCG,SGCA,SGCB,LAMA1,LAMA2,DAG1,DMD,DES,ACTB,ACTG1,EMD,LMNA,CTNNB1,TCF7,TCF7L1,TCF7L2,LEF1,CACNA1C,CACNA1D,CACNA1F,CACNA1S,CACNB1,CACNB2,CACNB3,CACNB4,CACNA2D1,CACNA2D2,CACNA2D3,CACNA2D4,CACNG1,CACNG2,CACNG3,CACNG4,CACNG5,CACNG6,CACNG7,CACNG8,RYR2,ATP2A1,ATP2A3,ATP2A2,SLC8A1,SLC8A2,SLC8A3,CDH2,JUP,CTNNA3,CTNNA1,CTNNA2,ACTN3,ACTN2,DSC2,PKP2,DSP,DSG2,GJA1",Arrhythmogenic right ventricular cardiomyopathy (ARVC),77

hsa05414,"ITGA1,ITGA2,ITGA2B,ITGA3,ITGA4,ITGA5,ITGA6,ITGA7,ITGA8,ITGA9,ITGA10,ITGA11,ITGAV,ITGB1,ITGB3,ITGB4,ITGB5,ITGB6,ITGB7,ITGB8,SGCD,SGCG,SGCA,SGCB,LAMA1,LAMA2,DAG1,DES,DMD,ACTB,ACTG1,TTN,TNNT2,TNNC1,TNNI3,ACTC1,TPM1,TPM2,TPM3,TPM4,MYBPC3,MYL3,MYL2,MYH7,MYH6,EMD,LMNA,LOC102723407,ADRB1,GNAS,ADCY1,ADCY2,ADCY3,ADCY4,ADCY5,ADCY6,ADCY7,ADCY8,ADCY9,PRKACA,PRKACB,PRKACG,CACNA1C,CACNA1D,CACNA1F,CACNA1S,CACNB1,CACNB2,CACNB3,CACNB4,CACNA2D1,CACNA2D2,CACNA2D3,CACNA2D4,CACNG1,CACNG2,CACNG3,CACNG4,CACNG5,CACNG6,CACNG7,CACNG8,RYR2,ATP2A1,ATP2A3,ATP2A2,PLN,SLC8A1,SLC8A2,SLC8A3,IGF1,TGFB1,TGFB2,TGFB3,TNF,AGT",Dilated cardiomyopathy (DCM),96

hsa05416,"CXADR,CD55,FYN,CAV1,ABL1,ABL2,RAC1,RAC2,RAC3,SGCD,SGCG,SGCA,SGCB,LAMA1,LAMA2,DAG1,DMD,ACTB,ACTG1,EIF4G3,EIF4G1,EIF4G2,CCND1,CASP8,BID,CYCS,CASP9,CASP3,MYH7,MYH6,CD40LG,CD40,HLA-DMA,HLA-DMB,HLA-DOA,HLA-DOB,HLA-DPA1,HLA-DPB1,HLA-DQA1,HLA-DQA2,HLA-DQB1,HLA-DRA,HLA-DRB1,HLA-DRB3,HLA-DRB4,HLA-DRB5,HLA-A,HLA-B,HLA-C,HLA-F,HLA-G,HLA-E,CD80,CD86,CD28,PRF1,ITGAL,ITGB2,ICAM1,LOC102723407",Viral myocarditis,60

hsa05418,"HSP90AA1,HSP90AB1,HSP90B1,NOS3,CAV1,CAV2,CAV3,CALML3,CALM2,CALM3,CALM1,CALML6,CALML5,CALML4,GPC1,SDC1,SDC2,SDC4,TRPV4,KEAP1,NFE2L2,HMOX1,GSTA5,GSTA2,GSTA4,GSTO2,GSTM4,GSTT2,GSTT1,GSTM3,MGST1,MGST3,GSTP1,GSTM1,GSTM5,MGST2,GSTA1,GSTM2,GSTA3,GSTO1,GSTT2B,NQO1,SQSTM1,TXN,TXN2,NPPC,ASS1,THBD,PLAT,CDH5,KDR,CTNNB1,PRKAA1,PRKAA2,PECAM1,PIK3CA,PIK3CD,PIK3CB,PIK3R1,PIK3R2,PIK3R3,AKT1,AKT2,AKT3,MAP2K5,MAPK7,MEF2A,MEF2C,KLF2,ITGA2B,ITGAV,ITGB3,SRC,PTK2,ARHGEF2,RHOA,ACTB,ACTG1,DUSP1,MIR10A,TNFRSF1A,IL1R1,IL1R2,MAP3K7,IKBKG,CHUK,IKBKB,NFKB1,RELA,MAP3K5,MAP2K4,MAP2K7,MAPK8,MAPK10,MAPK9,MAP2K6,MAPK11,MAPK12,MAPK13,MAPK14,FOS,JUN,MMP2,MMP9,CTSL,CCL2,VCAM1,ICAM1,SELE,TNF,IL1A,IL1B,IFNG,PDGFA,PDGFB,EDN1,VEGFA,BMP4,BMPR2,ACVR2A,ACVR2B,ACVR1,BMPR1A,BMPR1B,NOX1,CYBA,NCF1,NCF2,RAC1,RAC2,RAC3,PRKCZ,PIAS4,TP53,SUMO3,SUMO2,SUMO1,SUMO4,BCL2",Fluid shear stress and atherosclerosis,139
